# Enhanced crystallinity of tetrahalopyridyl (THP) derivatized compounds†

**DOI:** 10.1039/d6sc01377e

**Published:** 2026-04-20

**Authors:** Callum S. Begg, Viktoriya G. Dragomanova, Dmitry S. Yufit, Toby J. Blundell, Steven L. Cobb, Mark A. Fox, Matthew O. Kitching, William D. G. Brittain

**Affiliations:** a Department of Chemistry, Durham University South Road Durham DH1 3LE UK m.a.fox@durham.ac.uk matthew.o.kitching@durham.ac.uk william.d.brittain@durham.ac.uk

## Abstract

Promoting the formation of ordered crystalline material is a fundamental challenge in the fields of organic synthesis, crystal engineering and wider material science. Traditional approaches typically employ strong, unidirectional intermolecular interactions as the core principles for tecton and synthon design. In contrast, the interactions between complex biomolecules, such as proteins, take advantage of the cooperativity of multiple, weak, polyaxial non-covalent interactions (NCIs), working in concert, to generate strongly associated superstructures. Such design principles have yet to be successfully applied to small molecule crystal engineering. Here we show that the tetrahalopyridyl (THP) unit fulfils these tectonic criteria. Firstly, vast and varied THP based NCIs are identified within the Cambridge Structural Database (CSD). The diversity of NCIs is then validated through manual interrogation of a model library and quantified through quantum topological analyses using Bader's atoms in molecules (QTAIM), non-covalent interactions-reduced density gradient (NCI-RDG) and natural bond orbital (NBO) approaches. Furthermore, the critical importance of F⋯F interactions is revealed through analysis of 17 pairs of interactions in a diverse library of 12 related scaffolds. The utility of the approach is then shown across a wide variety of substrates including promoting natural product crystallinity and for application in absolute structural determination.

## Abstract 2

Derivatization of compounds with tetrahalopyridyl (THP) groups is presented as a broadly applicable route to increase their crystallinity.

## Graphical abstract

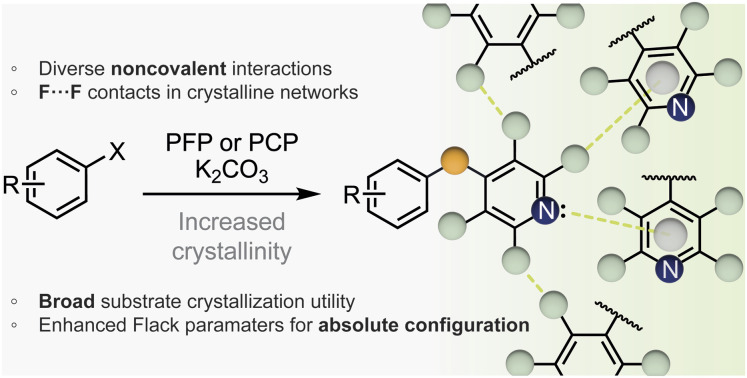

## Introduction

The ability to promote crystallinity is highly prized. Being able to influence the solid-state properties of molecules has vast implications for the fields of organic synthesis,^1–5^ crystal engineering,^6–8^ materials science^9–12^ as well as process development.^13^ However, the ability to predict whether a substance will be crystalline or not is at present extremely challenging. This has led to a central dogma within crystal engineering that a successful strategy to increase the degree of crystallinity of a compound is to introduce a single, strong, unidirectional supramolecular synthon.

**Fig. 1:**
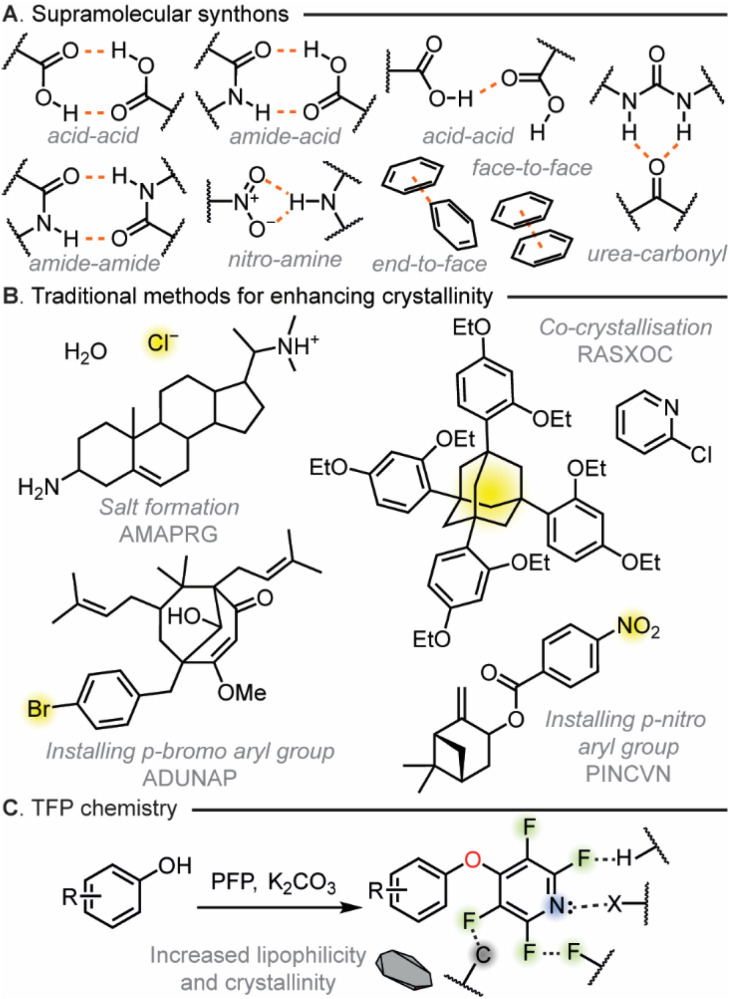
(A) Supramolecular synthons used in crystal engineering. (B) Traditional methods used to enhance material crystallinity. (C) Increased lipophilicity and crystallinity of TFP derivatised compounds compared to the starting material.

The utilization of synthons in crystal engineering was pioneered by Desiraju in 1995.^14^ This concept invoked structural units within supermolecules, formed through known or conceivable synthetic operations involving non-covalent interactions (NCIs), building large, structured arrays of molecules to underpin crystal engineering.^15,16^ These synthons can take many forms (Fig. 1A), including hydrogen bonds, halogen bonds and π⋯π interactions.^17–23^ The building blocks that can be introduced to display a particular synthon are referred to as tectons and derivatization with tectons has become a cornerstone of crystal engineering as well as taking a wider role in the control of molecular conformation and binding.^24–30^ Traditional strategies to enhance crystallinity of compounds have followed the synthon approach with salt formation,^31–33^ co-crystallization,^34–37^ and derivatization with a tecton (Fig. 1B) being mainstays in the field.^38^ All these approaches predominately rely on the invocation of a strong, unidirectional NCI within the solid state. However, this approach to stabilization is in sharp contrast to how biological processes utilize NCIs. All processes that are critical to life utilize NCIs to give exquisite selectivity within protein folding,^39,40^ DNA structure,^41–44^ and cell membrane interactions.^45–47^ Within nature it is the case that instead of a single synthon being used a wide variety of simultaneous cooperative synthons are deployed for stability. It should be noted that a range of non-covalent interactions have been explored by others to influence the crystallinity of aromatic molecules including halogen bonding thus we believed that a simple to install tecton approach that could utilise many NCIs may offer significant value.^48–53^

Taking inspiration from biology, we hypothesized that a tecton – which instead of invoking a single strong synthon – could invoke multiple cooperative polyaxial NCIs which is an unprecedented design concept to be applied to crystal engineering. To make this a practical concept we devised several tectonic design criteria: the tecton had to be able to be easily installed under mild conditions and needed to have broad applicability to a range of functionality traditionally found in organic molecules. From previous work utilizing them for synthetic methodology purposes we believed that 2,3,5,6-tetrahalopyridyl (THP) groups could meet our tecton design criteria.^54–57^ The high density of functionality within the THP groups would potentially allow it to partake in many NCIs including, N⋯H hydrogen bonding, X⋯X halogen bonding, X⋯H hydrogen bonding and π⋯π interactions.^58–60^ In addition, we have previously shown that tetrafluoropyridyl (TFP) derivatization can be readily accomplished utilising S_*N*_Ar chemistry under mild reaction conditions in up to quantitative yield without the requirement for purification.^54^ Subsequently as the incorporation of heavy atoms can be beneficial for absolute structure determination, we also wished to investigate the use of the tetrachloropyridyl (TCP) group which has not been previously studied in our group to see if this THP could also meet our tectonic criteria.

To fully investigate the THP groups as a new tecton to enhance crystallinity we designed a study to identify the types of NCIs possible, quantify their stabilization capability within the solid state and then apply them to a range of common synthetic applications. To do this we targeted a combined approach of utilizing synthetic methodology development, computational calculations and manual interrogation of single crystal X-ray diffraction (SCXRD) data (Fig. 1C). This would help us reveal the capability of the THP group's ability to influence the solid state.

## Results and discussion

**Fig. 2:**
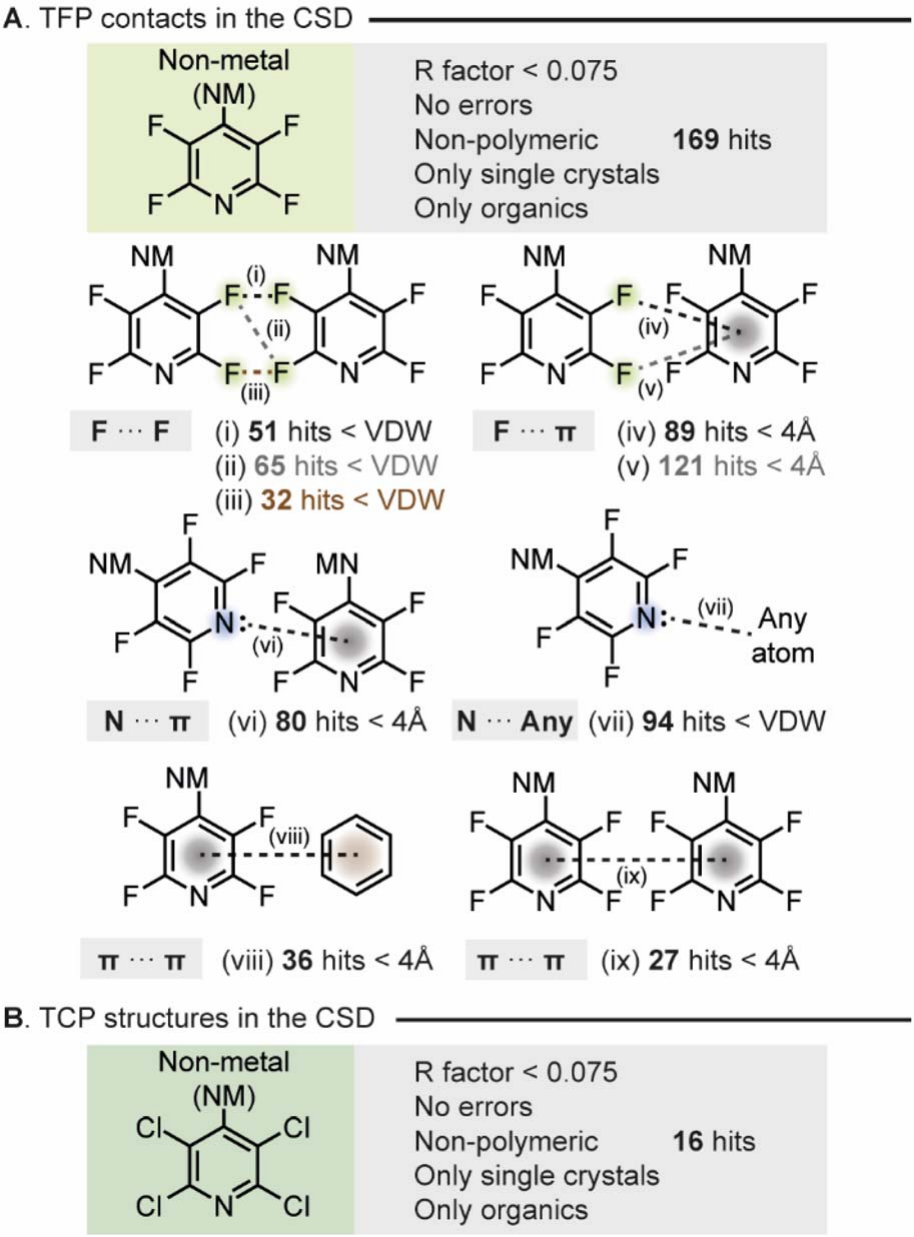
(A) TFP contacts made in the CSD. (i), (ii) and (iii) depict F⋯F contacts (<van der Waals radii); (iv) and (v) show F⋯π interactions (<4 Å); (vi) shows N⋯π-interactions (<4 Å); (vii) shows N···any atom contacts (<van der Waals radii); (viii) shows π⋯π interactions to a non-TFP ring (<4 Å); and (ix) shows π⋯π interactions to another TFP ring (<4 Å) (B) TCP derivitized structures in the CSD.

Initially, we wished to investigate the propensity of THP groups to engage in NCIs. To do this we decided to probe the identities and frequencies of NCIs attributed to THP groups within the Cambridge Structural Database (CSD). We triaged the multitude of interactions made by THP the groups in any structure that had been deposited and incorporated a TFP or a TCP group with a non-metal atom substituted on the 4-position of the pyridine ring. The search of TFP functionalities returned 169 hits. Firstly, we searched for the dominant interactions made between TFP groups in the crystal structures from the generated list. This investigation identified multiple F⋯F halogen bonded contacts (<van der Waals radii), F⋯π interactions (<4 Å), N⋯π-interactions (<4 Å), π⋯π interactions (<4 Å) and N⋯H interactions (<van der Waals radii) which could act to stabilize the structure in the solid state (Fig. 2A). Notably, approximately 57% of crystals contained an F⋯F contact from one TFP group to another TFP group. The next most prevalent interaction made by the TFP group was that of the nitrogen lone pair to other atoms within the crystal structure. Hence modulation of the availability of the pyridyl N-lone pair will alter crystallinity and solid-state packing for TFP substituted compounds. What is clear is that various direct interactions between TFP groups effectively stabilize the derivatized compounds in the solid state, thereby enhancing crystallinity.

Secondly, the search for TCP derivatized compounds yielded considerably fewer results (Fig. 2B), with 9 out of 16 hits corresponding to pentachloropyridine. Consequently, the analysis conducted for TFP derivatized compounds could not be performed on such a small dataset of TCP compounds. Nonetheless, we postulated that owing to the heightened propensity of chlorine to engage in halogen bonding,^20^ along with its ability to form a comparable myriad of intermolecular interactions, this functionality would likely exhibit a similar effect to TFP in augmenting material crystallinity.

**Fig. 3:**
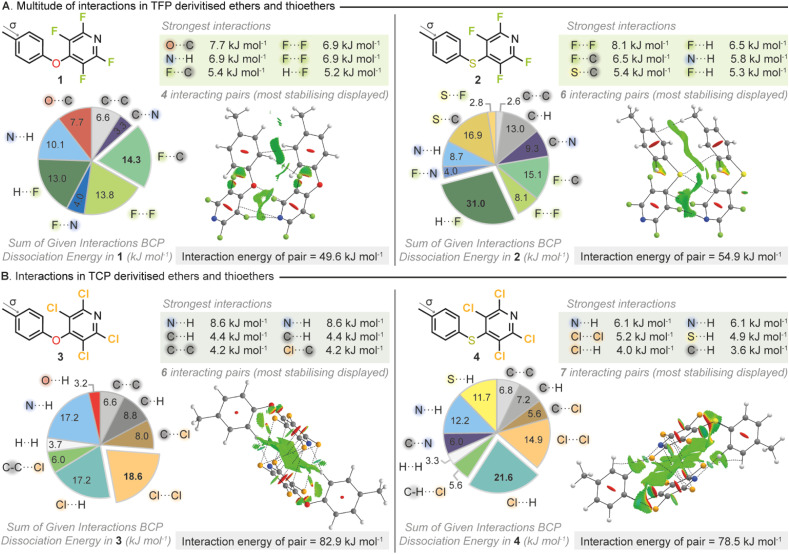
(A) Stabilizing interactions in THP derivatized ethers 1 and 2 calculated by QTAIM *in silico* analysis and visualized with BCP paths (dashed lines) and NCI-RDG contours (green favourable interactions, red unfavourable interactions). The molecular pair with the greatest stabilization energy is displayed for each structure. (B) Stabilizing interactions in THP derivatised thioethers 3 and 4 similarly analysed by QTAIM and NCI-RDG.

To gain quantitative insight into the number and strength of interactions that had been identified from the CSD search we next sought to apply computational approaches. We therefore went about synthesizing a set of model THP derivatives. A set of *para*-methyl variants (1–4) were selected as an appropriate test set and were synthesized utilizing the appropriate perhalopyridine and nucleophile. All materials were found to be highly crystalline and suitable for SCXRD. Utilization of computational approaches have been successfully employed to study NCIs both in solution and in the solid state.^61–63^ Therefore, high-level *ab initio* wavefunction computations were then carried out on all possible interacting pairs within the crystal structures of 1–4 with the overall interaction energy per pair determined (Fig. 3).

Each intermolecular interaction within a pair was explored using three distinct methods. (a) Quantum topological analyses using Bader's atoms in molecules (QTAIM) which reveal bond critical points (BCP) and their interaction energies in each interaction where present (b) non-covalent interactions-reduced density gradient (NCI-RDG) analyses show attractive interactions as contours (in green in figures) containing electrostatic/dispersive components and (c) natural bond orbital (NBO) analyses compute energies of weak charge transfer between orbitals.^64,65^ This suite of approaches allows for a full picture of the types and strengths of interactions present within the solid state to be gleaned.

It was immediately obvious from our calculations that the THP moiety can participate in many types of stabilizing interaction in the solid state. The breakdown of the intermolecular interaction energies (Fig. 3) demonstrated that the type of interactions that were stabilizing the crystals of the test set included hydrogen bonds, π–π interactions and halogen bonds with many different atoms taking part in NCIs. Whilst fluorine incorporation has been traditionally utilized to influence electrostatics,^66–70^ and has been used as a tool to invoke intramolecular interactions,^71,72^ it has been less well studied in its ability to enhance crystallinity as a derivatization tool. Surprisingly, further interrogation of the BCP dissociation energies for the TFP containing compounds (1–2) revealed that the fluorines were playing a critical role in stabilization of the solid state. Fluorine···fluorine interactions have been somewhat controversial and uncommonly reported especially given that the electrostatic terms are usually not attractive due to the absence of a σ-hole in a fluorine atom.^48^ However, several studies have identified or demonstrated that F⋯F interactions can have significant stabilizing properties due to their favorable dispersion and polarization terms which are not simply a byproduct of crystal packing.^73–76^ Within compound 1 the two largest contributions to the stabilizing intermolecular interactions identified, both involved fluorine, C⋯F (14.3 kJ mol^−1^) and F⋯F (13.8 kJ mol^−1^). In compound 2 where the connecting atom between the two rings was switched from oxygen to sulfur the single strongest intermolecular interaction identified was an F⋯F (8.1 kJ mol^−1^).

In the analogous TCP containing compounds 3 and 4 it was observed that again the THP moiety was participating in a range of stabilizing intermolecular interactions. In both TCP compounds an N⋯H hydrogen bond was identified as the single strongest interaction (3 = 8.6 kJ mol^−1^, 4 = 6.1 kJ mol^−1^). However, the sums of BCP dissociation energies across all interacting pairs within the crystal structures highlighted that the presence of a halogen was crucial in stabilizing the crystalline state. Across both compounds, 3 and 4, interactions involving chlorine were overall the most stabilizing with Cl⋯Cl (18.6 kJ mol^−1^) and Cl⋯H (21.6 kJ mol^−1^) being the strongest respectively. It should be noted that the overall stabilization energies were calculated to be higher for the two TCP containing compounds compared to the TFP analogues. This increased overall stabilization was mirrored in the compounds' respective melting points with compound 3 seeing its melting point (compared to *p*-cresol) increase from 34–35 °C to 106–107 °C following derivatization.^77^ Compound 4 behaved similarly with its melting point (compared to *p*-thiocresol) increasing from 41–43 °C to 113–114 °C after TCP installation.^78^ The applied wavefunction calculations to the test set had demonstrated that the THP moiety was participating in multiple different types of polyaxial intermolecular interactions. Therefore, the THP group could fulfil the tectonic properties we had originally envisaged in our design principles.

**Fig. 4:**
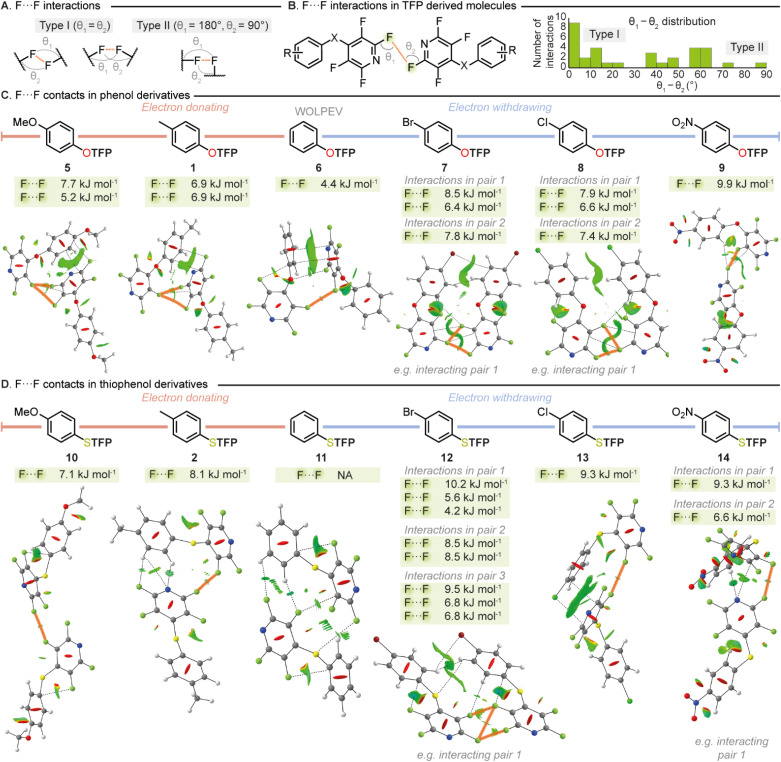
(A) Type I *versus* type II F⋯F contacts (B) F⋯F contact angle distribution within TFP derivatized molecules (C) F⋯F contacts identified in TFP derivatized phenols (D) F⋯F contacts identified in TFP derivatized thiophenols.

F⋯F interactions had been identified as being critical to the stabilization occurring within the crystal structures of the TFP compounds studied. Therefore, we decided to further expand our investigation into these intriguing intermolecular interactions. We conducted manual analysis of all TFP containing compounds disclosed in this report to measure the distances and geometry of all F⋯F interactions. It is well established that X⋯X halogen bonding can be characterized as either type I or type II (Fig. 4A). Type I interactions exhibit more van der Waals-like geometry with similar C–X⋯X bond angles for both X atoms whilst type II interactions exist through contacts made between electrophilic regions on one X atom with the nucleophilic regions of another and exist with *θ*_1_ − *θ*_2_ = 90°. Manual inspection of F⋯F contacts across our complete library of TFP derivatized aromatics revealed that these interactions prevail at an average distance of 2.85 ± 0.07 Å, with type I being the main interaction observed (37 F⋯F contacts: type I = 16 (43%), type II = 1 (3%)) (Fig. 4B). This distribution is consistent with the overall distribution of the types of F⋯F contacts present within the CSD.

To quantify the strengths of F⋯F interactions and to further explore the electronic influences on these critical stabilizing contacts we designed an electronically diverse *para*-substituted library (1, 2, 5–14) suitable for *ab initio* wavefunction computations. Compound substituents were selected to encompass a range of Hammett parameters, and two sets were chosen to probe the difference in the heteroatom between the two rings. Compounds (1, 2, 5–14) were readily synthesized from their respective nucleophile and pentafluoropyridine (PFP) and SCXRD data obtained as a basis for analysis. QTAIM, NCI-RDG and NBO analyses were conducted across the library on all individual interacting pairs (Fig. 4C and D).

It was revealed from the calculations that F⋯F contacts play a crucial role in stabilizing the crystal structures of both the phenol and thiophenol derived molecules regardless of electronic character. From strongly donating to strongly withdrawing phenolic substituents, F⋯F interactions were present and key to stabilization, giving further evidence that the proximity of the fluorine atoms in the solid state is not simply an artifact of packing but a key stabilizing feature. Intriguingly it was found that the presence of either an electron withdrawing or electron donating group significantly strengthened the F⋯F contacts within the crystalline state. Indeed, the only compound that was found not to possess a stabilizing F⋯F interaction by QTAIM analysis was the unsubstituted sulfur containing compound 11. However, a small favorable NCI-RDG contour of one F⋯F interaction was present in 11 and a weak donor–acceptor at the same F⋯F interaction was determined at 0.3 kJ mol^−1^ from the NBO analysis. This is in sharp contrast to when an electron donating substituent (*e.g.*10, F⋯F, 7.1 kJ mol^−1^) or electron withdrawing substituent (*e.g.*14, total F⋯F, 15.9 kJ mol^−1^) is introduced which greatly strengthens these interactions. This phenomenon was observed across both oxygen (Fig. 4C) and sulfur containing compounds (Fig. 4D). It should be noted that we also conducted studies into intramolecular interactions with several compounds displaying favorable halogen–carbon interactions by QTAIM and NBO analyses (see SI) further demonstrating the key role of halogen incorporation to the THP synthon.

From these exploratory studies the THP moiety had been successfully identified and quantified to exhibit the tectonic behavior required to meet our core design principles. The THP group was being seen to participate in multi-directional, multi-modal interactions and the solid state was being stabilised by the sum of many contacts. Thus, we set about exploring the derivatization of a range of scaffolds to optimize installation conditions and to probe the limits of synthetic applicability.

**Fig. 5:**
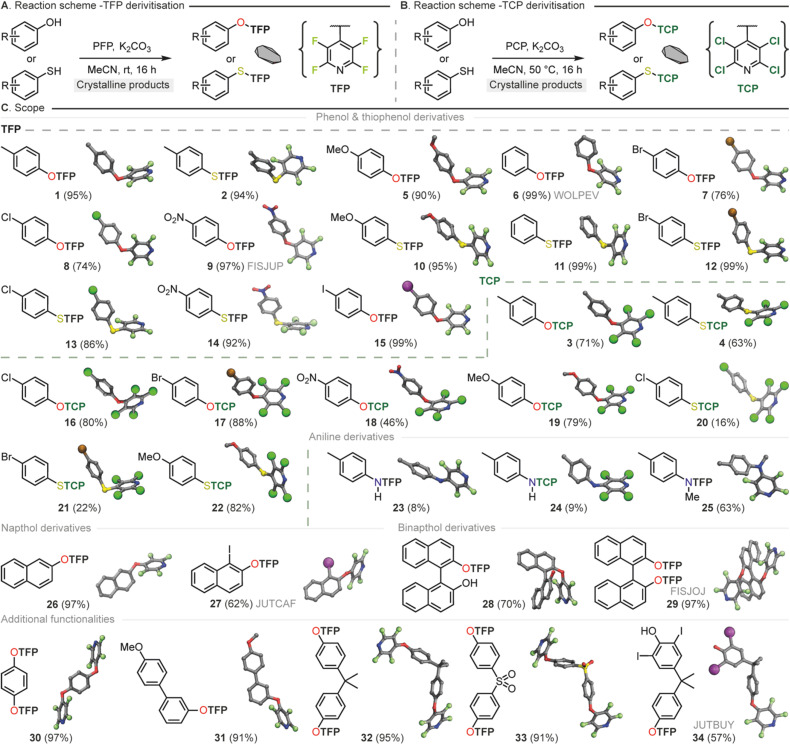
(A) Scheme for the synthesis of TFP compounds through reaction of PFP with phenol and thiophenol analogues. (B) Scheme for the synthesis of TCP compounds through reaction of PCP with phenol and thiophenol analogs. (C) Library of PFP compounds with crystal structure renders (yield%).

Within our research group, general conditions for the installation of TFP groups had been developed.^54,55^ By mixing the compound for derivatization *e.g.* phenol, thiophenol or aniline with a small excess of pentafluoropyridine (PFP) in the presence of base (*e.g.* K_2_CO_3_) at room temperature the TFP appended compound can be isolated in high yields (up to 99%) without the need for purification (Fig. 5A). Due to the low boiling nature of PFP it can be simply removed by rotary evaporation making it especially attractive for either high throughput applications or when amounts of material to be crystallized may be limited.

In the case of TCP installation, it was found that due to the less reactive nature of pentachloropyridine (PCP) in contrast to PFP in S_*N*_Ar processes that a slightly elevated temperature (50 °C) helped to maximise yield. These general conditions worked well across a broad range of phenol and thiophenol substrates (Fig. 5B). The TCP derivatives were observed to have higher melting points that their TFP equivalents but did require purification by column chromatography to obtain material sufficiently pure for single crystal analysis. TCP derivatizations are therefore recommended in cases where increasing the likelihood of crystallinity is paramount above other concerns, or in specialist applications where heavy atoms are required.

In both cases (TFP/TCP) derivatization of primary anilines (see SI) to generate mono THP containing secondary amines was found to be challenging (23 and 24). The characteristics of the nucleophile in these cases did not seem well suited for our approach and a mixture of unreacted starting material and undesired overreaction was observed. In these cases, we were able to obtain sufficient material for SCXRD following purification, It should be noted that Pennington and co-workers have previously disclosed the synthesis of a selection of TFP amines however this required significant excess amine that may be an issue in the case of crystallization of small amounts of material.^79^

In general, the TFP and TCP groups were found to be readily installed across a wide variety of nucleophiles of varying electronic and steric character (1–34). With this knowledge in hand we targeted the use of the THP synthon for two widely applicable crystal engineering and synthetic chemistry applications. Those being, utilization as a crystal promoting tag to render non-crystalline material suitable for single crystal analysis and the use of the methodology in natural product absolute structure determination.

**Fig. 6:**
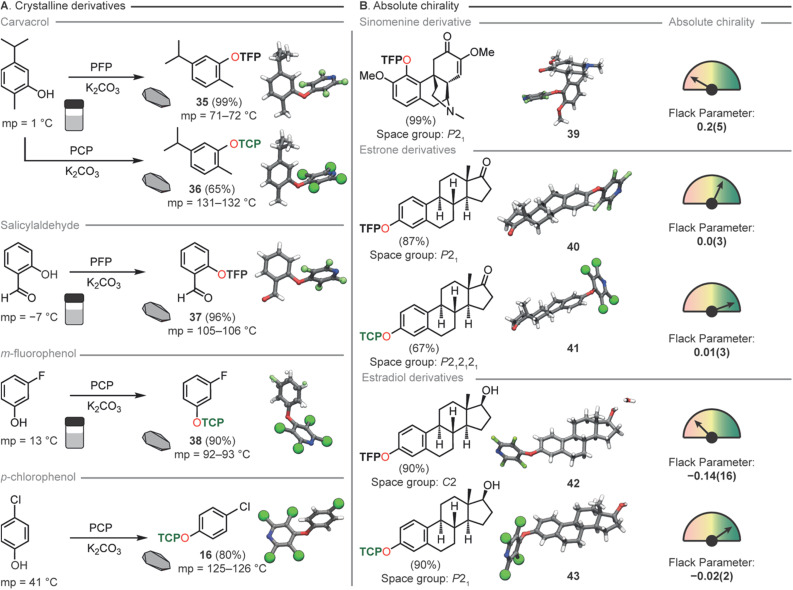
(A) Increasing the melting point of compounds through TFP and TCP incorporation. (B) Determination of absolute chirality through TFP and TCP derivatization of natural products.

By taking the liquid monoterpenoid phenolic natural product carvacrol^80^ and exposing it to our developed conditions TFP and TCP groups were readily installed, generating 35 and 36 in 99% and 65% yields respectively. Both 35 and 36 had a greatly increased melting point (71–72 °C and 131–132 °C), compared to the parent phenol (1 °C) (Fig. 6A) rendering the compound suitable for generation of single crystals at room temperature. This significant change in melting point was also demonstrated for salicylaldehyde (37), *m*-fluorophenol (38) and *p*-chlorophenol (16). Furthermore, the incorporation of the TCP group resulted in a greater elevation of the melting point compared to TFP analogues, matching the observations from our *ab initio* computations.

Due to the localization of electron density onto the halogen atoms substituted on the THP ring we hypothesized that this synthon could also act as an easy-to-install handle for either connectivity analysis or absolute determination of chirality for natural product identification (Fig. 6B). TFP was successfully installed in 99% yield into sinomenine (39), an alkaloid natural product used in traditional medicine for the treatment of rheumatic and arthritic diseases.^81^ This derivatization rendered the material highly crystalline and a suitable sample for SCXRD was easily obtained. The resulting crystal structure whilst adequate for connectivity analysis gave a Flack parameter which was not sufficient for absolute structure determination. This led us to generate two analogues of estrone to probe the influence of the halogen in the THP moiety for absolute structure determination. TFP (40) and TCP (41) were installed onto estrone in 87% and 67% yield respectively. SCXRD analysis of the two analogues showed in this case that both THP groups gave sufficient Flack parameters (0.0(3), 40: 0.01(3), 41) for absolute chirality determination. However, as expected, the TCP-functionalised analogue exhibited superior Flack and Hooft parameters, as well as a more favorable Parson's quotient compared to the TFP-functionalized molecule. To demonstrate that further chemical reactions can be conducted on THP appended molecules we next reduced compounds 40 and 41 to the corresponding estradiol derivatives 42 and 43 both in 90% yields. We had previously disclosed this transformation for 40 however we did not obtain SCXRD data and thus were unable to comment on the diastereoselectivity of the reduction.^54^ Again, the same trends in SCXRD data were observed with the TCP analogue exhibiting the superior Flack and Hooft parameters and Parson's quotient. From these data we were able to use the TFP group to tentatively assign the stereochemistry as (*S*) at the hydroxyl carbon of 40 with a Flack parameter of (−0.14(16)) whilst chirality of 41 could be unequivocally determined to be (*S*) at the hydroxyl carbon with a Flack parameter of (0.02(2)).

From these natural product derivatization studies, the TFP and TCP groups are perhaps best employed for slightly different scenarios. Where absolute structure determination is key the TCP group gives better SCXRD data. The TCP group also raises the melting points of materials higher than for TFP compounds thus if either of these factors are critical to the desired application then TCP was concluded to be the better THP choice. If connectivity is only required than TFP can be superior due to its general ease of installation and lack of purification requirement. We envisage that TFP tagging can be used as a scouting technology for identification of materials in high throughput applications whilst TCP will be used for singular application where high crystallinity for absolute structure determination is the desired goal.

## Conclusions

We have successfully identified, quantified and applied the THP tecton to give a versatile tool for crystal engineering and synthetic chemistry. The THP group represents a new approach to crystallization of small molecules where many multi-directional, multi-modal NCIs are employed to stabilize the solid state. This approach mirrors that of biological systems in invoking a range of interactions in contrast to the traditional synthon-based approach for crystal formation. Through exploration of the CSD and analysis of our own library of compounds, we have identified key interactions that are dominated by hydrogen bonding and X⋯X halogen bonding interactions. Consequently, any modulation of these contacts has profound effects on the crystalline properties observed. We have elucidated that these solid-state interactions are highly influenced by the electronic properties of aryl rings adjacent to the site of THP installation, the ability of the THP pyridyl nitrogen to partake in hydrogen bonding interactions and the presence of any other hydrogen bond donors. The THP group has proven to be easily installable and can effectively convert liquids into crystalline solids. Moreover, TCP groups serve as a valuable tool for determining the absolute chirality of compounds whilst TFP groups act as an easy-to-deploy tool for high throughput screening applications.

## Author contributions

C. S. B. conducted crystallographic studies, data analysis, wrote sections of the manuscript and the SI. V. G. D. synthesized several compounds and contributed to the SI. D. S. Y. and T. J. B. conducted crystallographic studies and analysis. M. A. F. performed all *ab initio* computations and analysed the data. M. O. K. co-conceived/co-supervised the study. S. L. C. co-conceived the study. W. D. G. B. conducted the synthetic aspects, co-conceived/co-supervised the study, and wrote sections of the manuscript and SI. All co-authors were involved in data analysis, contributed ideas and contributed towards the preparation of the manuscript.

## Conflicts of interest

There are no conflicts to declare.

## Supplementary Material

SC-017-D6SC01377E-s001

SC-017-D6SC01377E-s002

SC-017-D6SC01377E-s003

## Data Availability

CCDC 2392089–2392126 (1–5, 7, 8, 10–26, 28, 30–33 and 35–43) contain the supplementary crystallographic data for this paper.^82*a*–*al*^ All data supporting this study are available within the article and in the supplementary information (SI). Supplementary information: general experimental details, synthetic methods, computational calculations, crystallographic data, characterization data and spectroscopic data. See DOI: https://doi.org/10.1039/d6sc01377e.
