# Supplementary material for: Enhanced crystallinity of tetrahalopyridyl (THP) derivatized compounds
Source: Chem Sci. 2026 Apr 20;17(22):10948–57. doi: 10.1039/d6sc01377e (PMC13115132; doi:10.1039/d6sc01377e)
Supplement: SC-017-D6SC01377E-s001 [file SC-017-D6SC01377E-s001.pdf]

Supplementary Information (SI) for Chemical Science.
This journal is © The Royal Society of Chemistry 2026

Supporting Information

Enhanced Crystallinity of Tetrahalopyridyl (THP) Derivatized Compounds

Callum S. Begg,[a] Viktoriya G. Dragomanova,[a] Dmitry S. Yufit,[a] Toby J. Blundell,[a] Steven L. Cobb,[a] Mark A. Fox,*[a] Matthew O. Kitching,*[a] and William D. G. Brittain*[a]

[a] Department of Chemistry, Durham University, South Road, Durham, DH1 3LE, United Kingdom

*Corresponding Author emails: m.a.fox@durham.ac.uk, matthew.o.kitching@durham.ac.uk, william.d.brittain@durham.ac.uk

Contents

General Experimental....S-2

General Procedures....S-3

Characterization Data....S-4

NMR Data for Synthesized Compounds....S-29

CSD Search Parameters....S-145

Crystallographic Information....S-151

Computational Data....S-194

References....S-256

General Experimental

All starting materials and reagents were purchased from commercial sources and used as received. All reactions were conducted under an atmosphere of air. ^{1}H NMR spectra were recorded at 400 or 600 MHz using Bruker Avance III or Varian VNMRS-600 spectrometers respectively. $^{13}C\{^{1}H\}$ NMR spectra were recorded at 100 or 151 MHz using a Bruker Avance III or Varian VNMRS-600 respectively. $^{19}F\{^{1}H\}$ NMR spectra were recorded at 376 MHz using a Bruker Avance III spectrometer. All coupling constants are reported in Hertz (Hz). In cases where it was required, 2D NMR techniques were used to confirm compound identity. Chemical shifts are reported in ppm and are referenced to residual solvent peaks; $CHCl_3$ (^{1}H 7.26 ppm, ^{13}C 77.0 ppm), CH_3CN (^{1}H 1.94 ppm, ^{13}C 1.89 ppm) or DMSO (^{1}H 2.50 ppm, ^{13}C 39.5 ppm). Mass spectra were collected either using ESI-LC or GCMS. ESI-LC in MeCN were collected using a Waters TQD mass spectrometer with a Acquity UPLC BEH C18 1.7 µm (2.1 mm x 50 mm). ESI-LC was collected using water containing formic acid (0.1% v/v) and MeCN mixture in a 95:5 to 5:95 gradient over 5 min. GCMS experiments were carried out on a Shimadzu QP2010-Ultra with a Rxi-5Sil MS (0.15µm x 10m x 0.15 mm). Helium was employed as the carrier gas (0.41 mL/min). EI is carried at 70 eV and the working mass range is 35 – 650 au for all GCMS experiments. ASAP samples were run isothermally at 350 °C vaporizing the sample to enable atmospheric pressure chemical ionization. Melting points are uncorrected and were carried out in triplicate, and an average of the values taken and reported as a range using a Stuart SMP10 or Gallenkamp MDP 350 melting point apparatus. Compounds **6**, **27**, **29** and **34** have been previously reported with their corresponding crystal structures.[1][2] For the sake of completeness, the characterization data for all compounds are given in full here.

General Procedure for the Synthesis of Tetrafluoropyridyl Ethers

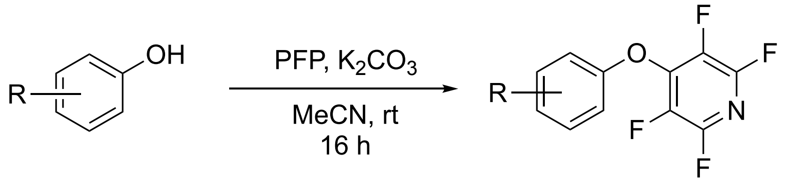

To a stirred solution of phenol (1.00 equiv.) in acetonitrile (10 mL) was added pentafluoropyridine (1.05 equiv.) and potassium carbonate (1.05 equiv.). The reaction mixture was stirred at room temperature for 16 h. After this time the reaction mixture was filtered and concentrated under reduced pressure. The resulting residue was passed through a short silica plug (100% EtOAc), concentrated and then purified directly by flash column chromatography if required.

General Procedure for the Synthesis of Tetrafluoropyridyl Thioethers

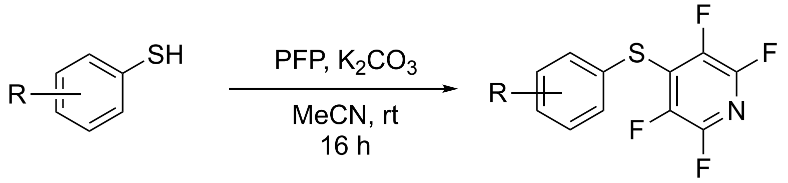

To a stirred solution of thiophenol (1.00 equiv.) in acetonitrile (10 mL) was added pentafluoropyridine (1.05 equiv.) and potassium carbonate (1.05 equiv.). The reaction mixture was stirred at room temperature for 4 h. After this time the resulting solution was filtered and concentrated under reduced pressure. The resulting residue was passed through a short silica plug (100% EtOAc), concentrated, and then purified directly by flash column chromatography if required.

General Procedure for the Synthesis of Tetrachloropyridyl Ethers

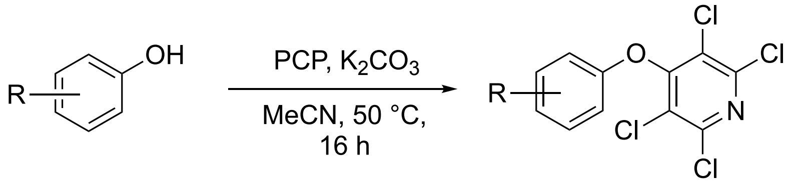

To a stirred solution of phenol (1.00 equiv.) in acetonitrile (10 mL) was added pentachloropyridine (1.00 equiv.) and potassium carbonate (1.05 equiv.). The reaction mixture was stirred at 50 °C for 16

h. After this time the resulting solution was filtered and concentrated under reduced pressure the recovered residue was then directly subjected to flash column chromatography.

General Procedure for the Synthesis of Tetrachloropyridyl Thioethers

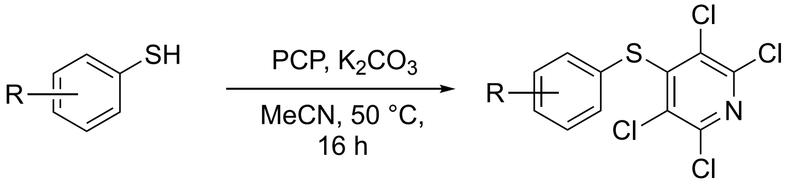

To a stirred solution of thiophenol (1.00 equiv.) in acetonitrile (10 mL) was added pentachloropyridine (1.00 equiv.) and potassium carbonate (1.05 equiv.). The reaction mixture was stirred at 50 °C for 16 h. After this time the resulting solution was filtered and concentrated under reduced pressure the recovered residue was then directly subjected to flash column chromatography.

Synthesis of 2,3,5,6-tetrafluoro-4-(*p*-tolyloxy)pyridine (1)

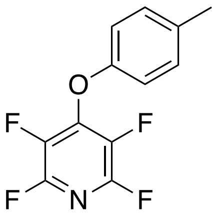

The title compound was synthesized according to the general procedure for the synthesis of tetrafluoropyridyl ethers from 4.63 mmol of the corresponding phenol as a clear crystalline solid (1.13 g) in 95% yield.

^{1}H NMR (400 MHz, CDCl$_3$) δ 7.25 – 7.11 (m, 2H, Ar*H*), 6.98 (app. d, *J* = 8.6, 2H, Ar*H*), 2.38 (s, 3H, C*H$_3$*).

^{19}F{^{1}H} NMR (376 MHz, CDCl$_3$) δ −88.46 – −89.71 (m), −154.17 – −155.60 (m).

^{13}C{^{1}H} NMR (176 MHz, CDCl$_3$) δ 153.78, 145.04 – 144.57 (m), 143.86 – 143.04 (m), 137.16 – 136.45 (m), 135.55 – 135.17 (m), 134.90, 130.38, 116.56, 20.66.

HRMS ESI$^-$ Calculated for [M−H]$^-$ $C_{12}H_6NOF_4^-$ = 256.0386. Found = 256.0376.

MP 52 – 53 °C.

Synthesis of 2,3,5,6-tetrafluoro-4-(*p*-tolylthio)pyridine (2)

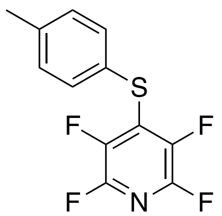

The title compound was synthesized according to the general procedure for the synthesis of tetrafluoropyridyl thioethers from 0.80 mmol of the corresponding thiophenol as a white solid (0.204 g) in 94% yield.

^{1}H NMR (400 MHz, $CDCl_3$) δ 7.45 (d, J = 8.0, 2H, ArH), 7.20 (d, J = 8.0, 2H, ArH), 2.40 (s, 3H, CH_3).

^{19}F{^{1}H} NMR (376 MHz, $CDCl_3$) δ −90.63 – −91.02 (m), −136.92 – −137.13 (m).

^{13}C{^{1}H} NMR (101 MHz, $CDCl_3$) δ 144.96 – 144.41 (m), 142.53 – 141.84 (m), 140.12, 139.86 – 139.24 (m), 133.44, 131.95 – 131.29 (m), 130.41, 125.34, 21.26.

HRMS AP$^+$ Calculated for $[M+H]^+$ $C_{12}H_8NF_4S^+$ = 274.0314. Found= 274.0310.

MP 39 – 40 °C.

Synthesis of 2,3,5,6-tetrachloro-4-(*p*-tolyloxy)pyridine (3)

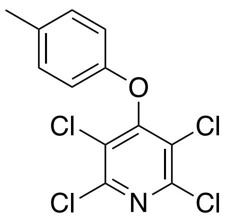

The title compound was synthesized according to the general procedure for the synthesis of tetrachloropyridyl ethers from 1.85 mmol of the corresponding phenol. Purified by flash column chromatography 100% hexanes to 90% hexanes 10% EtOAc. This gave the product as a white crystalline solid (0.424 g) in 71% yield.

^{1}H NMR (400 MHz, $CDCl_3$) δ 7.18 – 7.13 (m, 2H, ArH), 6.76 (d, J = 8.6, 2H, ArH), 2.35 (s, 3H, CH_3).

^{13}C{^{1}H} NMR (101 MHz, $CDCl_3$) δ 157.61, 153.24, 147.18, 133.63, 130.47, 125.63, 115.24, 20.65.

HRMS ESI$^+$ Calculated for $[M+H]^+$ $C_{12}H_8NOCl_4^+$ = 321.9360. Found= 321.9357.

MP 106 – 107 °C.

Synthesis of 2,3,5,6-tetrachloro-4-(*p*-tolylthio)pyridine (4)

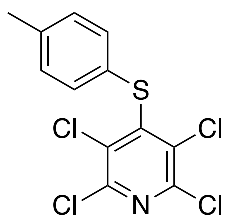

The title compound was synthesized according to the general procedure for the synthesis of tetrachloropyridyl ethers from 1.61 mmol of the corresponding thiophenol. Purified by flash column chromatography 100% hexanes to 90% hexanes 10% EtOAc. This gave the product as a white crystalline solid (0.382 g) in 63% yield.

^{1}H NMR (400 MHz, $CDCl_3$) δ 7.21 (d, J = 8.1, 2H, ArH), 7.15 (d, J = 8.1, 2H, ArH), 2.36 (s, 3H, CH_3).

^{13}C{^{1}H} NMR (101 MHz, $CDCl_3$) δ 148.35, 146.54, 138.76, 133.70, 131.17, 130.36, 128.32, 21.21.

HRMS ESI$^+$ Calculated for $[M+H]^+$ $C_{12}H_8NSCl_4^+$ = 337.9132. Found = 337.9135.

MP 113 – 114 °C.

Synthesis of 2,3,5,6-tetrafluoro-4-(4′-methoxyphenoxy)pyridine (5)

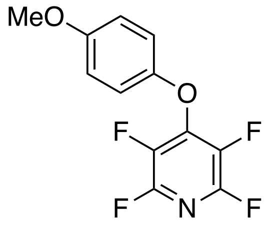

The title compound was synthesized according to the general procedure for the synthesis of tetrafluoropyridyl ethers from 1.61 mmol of the corresponding phenol as a white solid (0.394 g) in 90% yield.

^{1}H NMR (400 MHz, $CDCl_3$) δ 7.05 (d, J = 9.2, 2H, ArH), 6.91 (d, J = 9.2, 2H, ArH), 3.83 (s, 3H, CH_3).

^{19}F{^{1}H} NMR (376 MHz, $CDCl_3$) δ −88.94 – −89.22 (m), −154.98 – −155.18 (m).

^{13}C{^{1}H} NMR (101 MHz, $CDCl_3$) δ 156.97, 149.70, 145.72 – 144.91 (m), 143.27 – 142.63 (m), 137.59 – 136.86 (m), 135.04 – 134.27 (m), 118.23, 114.89, 55.69.

HRMS ESI$^-$ Calculated for $[M-H]^-$ $C_{12}H_6NO_2F_4^-$ = 272.0327. Found = 272.0335.

MP 76 – 78 °C.

Synthesis of 2,3,5,6-tetrafluoro-4-phenoxypyridine (6)

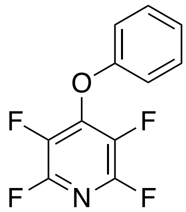

The title compound was synthesized according to the general procedure for the synthesis of tetrafluoropyridyl ethers from 1.06 mmol of the corresponding phenol as a white solid (0.257 g) in 99% yield.

^{1}H NMR (400 MHz, $CDCl_3$) δ 7.47 – 7.37 (m, 2H, Ar*H*), 7.31 – 7.21 (m, 1H, Ar*H*), 7.09 (d, *J* = 8.1 Hz, 2H, Ar*H*).

$^{19}F\{^{1}H\}$ NMR (376 MHz, $CDCl_3$) δ −88.58 – −88.84 (m), −154.23 – −154.44 (m).

$^{13}C\{^{1}H\}$ NMR (101 MHz, $CDCl_3$) δ 155.82, 145.61 – 145.20 (m), 144.60 – 144.26 (m), 143.18 – 142.78 (m), 137.76 – 137.25 (m), 135.13 – 134.63 (m), 130.05, 125.17, 116.68.

HRMS AP$^+$ Calculated for $[M+H]^+$ $C_{11}H_6NF_4O^+$ = 244.0386. Found = 244.0378.

MP 32 – 33 °C.

Synthesis of 2,3,5,6-tetrafluoro-4-(4′-bromophenoxy)pyridine (7)

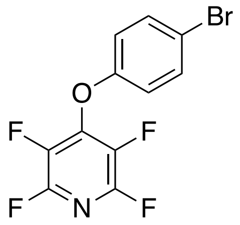

The title compound was synthesized according to the general procedure for the synthesis of tetrafluoropyridyl ethers from 4.62 mmol of 4-bromophenol, pentafluoropyridine (4.86 mmol) and K_2CO_3 (4.86 mmol). The solution was passed through a short silica plug (100% EtOAc) to give the desired product as a clear crystalline solid (1.12 g) in 76% yield.

^{1}H NMR (400 MHz, $CDCl_3$) δ 7.53 (d, *J* = 9.0, 2H, Ar*H*), 6.98 (d, *J* = 9.0, 2H, Ar*H*).

$^{19}F\{^{1}H\}$ NMR (376 MHz, $CDCl_3$) δ −87.90 – −88.43 (m), −153.93 – −154.20 (m).

$^{13}C\{^{1}H\}$ NMR (101 MHz, $CDCl_3$) δ 154.81, 145.68 – 145.08 (m), 144.25 – 143.67 (m), 143.20 – 142.60 (m), 137.72 – 137.04 (m), 135.05 – 134.38 (m), 133.05, 118.46, 117.98.

HRMS ESI$^-$ Calculated for $[M-H]^-$ $C_{11}H_3NOF_4Br^-$ = 319.9334. Found = 319.9312.

MP 61 – 62 °C.

Synthesis of 2,3,5,6-tetrafluoro-4-(4′-chlorophenoxy)- pyridine (8)

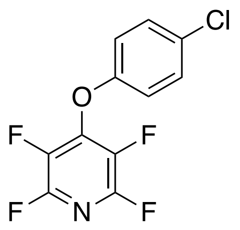

The title compound was synthesized according to the general procedure for the synthesis of tetrafluoropyridyl ethers from 3.89 mmol of 4-chlorophenol, pentafluoropyridine (4.09 mmol) and K_2CO_3 (4.09 mmol). The solution was passed through a short silica plug (100% EtOAc) to give the desired product as a clear crystalline solid (0.795 g) in 74% yield.

^{1}H NMR (400 MHz, $CDCl_3$) δ 7.38 (d, *J* = 9.1, 2H, Ar*H*), 7.04 (d, *J* = 9.1, 2H, Ar*H*).

^{19}F{^{1}H} NMR (376 MHz, $CDCl_3$) δ −87.99 – −88.29 (m), −154.04 – −154.24 (m).

^{13}C{^{1}H} NMR (101 MHz, $CDCl_3$) δ 154.25, 145.65 – 145.09 (m), 144.29 – 143.84 (m), 143.19 – 142.70 (m), 137.63 – 137.09 (m), 135.05 – 134.49 (m), 130.54, 130.08, 118.09.

HRMS ESI$^-$ Calculated for [M−H]$^-$ $C_{11}H_3NOF_4Cl^-$ = 275.9839. Found = 275.9820.

MP 31 – 32 °C.

Synthesis of 2,3,5,6-tetrafluoro-4-(4′-nitrophenoxy)pyridine (9)

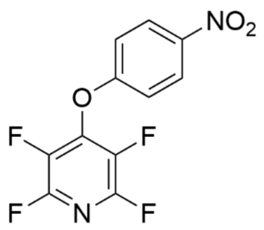

The title compound was synthesized according to the general procedure for the synthesis of tetrafluoropyridyl ethers from 3.60 mmol of the corresponding phenol as a white solid (1.01 g) in 97% yield.

^{1}H NMR (400 MHz, $CDCl_3$) δ 8.33 (d, *J* = 9.2, 2H, Ar*H*), 7.20 (d, *J* = 9.2, 2H, Ar*H*).

^{19}F{^{1}H} NMR (376 MHz, $CDCl_3$) δ −85.75 – −87.34 (m), −152.80 – −154.91 (m).

^{13}C{^{1}H} NMR (176 MHz, $CDCl_3$) δ 159.59, 145.01 – 144.75 (m), 144.66, 143.63 – 143.35 (m), 142.76 – 142.51 (m), 137.08 – 136.64 (m), 135.54 – 135.19 (m), 126.19, 116.69.

HRMS ESI$^-$ Calculated for [M−H]$^-$ $C_{11}H_4N_2O_3F_4^-$ = 287.0080. Found = 287.0091.

MP 84 – 85 °C.

Synthesis of 2,3,5,6-tetrafluoro-4-((4′-methoxyphenyl)thio)pyridine (10)

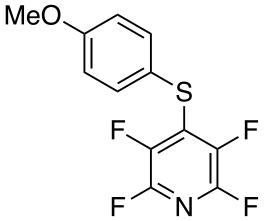

The title compound was synthesized according to the general procedure for the synthesis of tetrafluoropyridyl thioethers from 1.43 mmol of the corresponding thiophenol as a white crystalline solid (0.393 g) in 95% yield.

^{1}H NMR (400 MHz, $CDCl_3$) δ 7.54 (d, *J* = 8.9, 2H, Ar*H*), 6.92 (d, *J* = 8.9, 2H, Ar*H*), 3.85 (s, 3H, CH_3).

^{19}F{^{1}H} (376 MHz, $CDCl_3$) δ −90.94 – −91.11 (m), −137.83 – −138.01 (m).

^{13}C{^{1}H} NMR (101 MHz, $CDCl_3$) δ 161.12, 145.01 – 144.31 (m), 142.58 – 141.61 (m), 139.66 – 139.05 (m), 136.01, 132.84 – 131.81 (m), 118.71, 115.17, 55.45.

HRMS AP$^+$ Calculated for $[M+H]^+$ $C_{12}H_8NOF_4S^+$ = 290.0247. Found = 290.0263.

MP 34 – 36 °C.

Synthesis of 2,3,5,6-tetrafluoro-4-(phenylthio)pyridine (11)

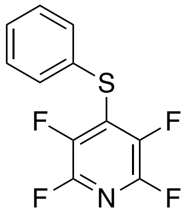

The title compound was synthesized according to the general procedure for the synthesis of tetrafluoropyridyl thioethers from 0.90 mmol of the corresponding phenol as a clear oil (0.232 g) in 99% yield. Crystals suitable for single crystal x-ray diffraction were obtained by cooling the material in a fridge.

^{1}H NMR (400 MHz, $CDCl_3$) δ 7.61 – 7.48 (m, 2H, Ar*H*), 7.46 – 7.36 (m, 3H, Ar*H*).

^{19}F{^{1}H} NMR (376 MHz, $CDCl_3$) δ −90.35 – −90.63 (m), −136.35 – −136.54 (m).

^{13}C{^{1}H} NMR (101 MHz, $CDCl_3$) δ 145.00 – 144.52 (m), 142.54 – 142.05 (m), 139.97 – 139.45 (m), 132.95, 131.20 – 130.76 (m), 129.66, 129.50, 129.18.

HRMS AP$^+$ Calculated for $[M+H]^+$ $C_{11}H_6NF_4S^+$ = 260.0157. Found = 260.0148.

MP 30 – 31 °C.

Synthesis of 2,3,5,6-tetrafluoro-4-((4′-bromophenyl)thio)pyridine (12)

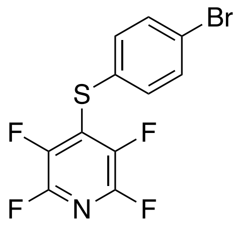

The title compound was synthesized according to the general procedure for the synthesis of tetrafluoropyridyl thioethers from 26.5 mmol of the corresponding thiophenol as pale yellow crystals (8.89 g) in 99% yield.

^{1}H NMR (400 MHz, $CDCl_3$) δ 7.50 (d, *J* = 8.5, 2H, Ar*H*), 7.38 (d, *J* = 8.5, 2H, Ar*H*).

^{19}F{^{1}H} NMR (376 MHz, $CDCl_3$) δ −89.85 – −90.02 (m), −136.10 – −136.28 (m).

^{13}C{^{1}H} NMR (101 MHz, $CDCl_3$) δ 144.98 – 144.54 (m), 142.52 – 142.01 (m), 139.93 – 139.47 (m), 134.42, 132.87, 132.23, 130.52 – 129.98 (m), 129.36, 128.22, 124.17.

ASAP MS: R_t = 0.65 min; *m/z* (AI$^+$): 339.9 ([M(^{81}Br)+H]$^+$, 99%), 337.9 ([M(^{79}Br)+H]$^+$, 100%).

HRMS AP$^+$ Calculated for [M+H]$^+$ $C_{11}H_5BrF_4NS^+$ = 337.9262. Found = 337.9273.

R_f = 0.48 (0.5:9.5, EtOAc:Hexanes).

MP 55 – 56 °C.

Synthesis of 2,3,5,6-tetrafluoro-4-((4′-chlorophenyl)thio)pyridine (13)

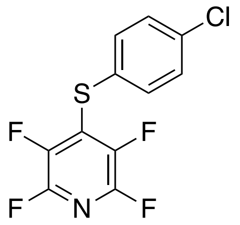

The title compound was synthesized according to the general procedure for the synthesis of tetrafluoropyridyl thioethers from 3.46 mmol of the corresponding thiophenol as pale yellow crystals (0.87 g) in 86% yield.

^{1}H NMR (400 MHz, $CDCl_3$) δ 7.49 (d, *J* = 8.7, 2H, Ar*H*), 7.38 (d, *J* = 8.7, 2H, Ar*H*).

^{19}F{^{1}H} NMR (376 MHz, $CDCl_3$) δ −89.91 – −90.08 (m), −136.24 – −136.42 (m).

^{13}C{^{1}H} NMR (101 MHz, $CDCl_3$) δ 145.03 – 144.49 (m), 142.49 – 142.02 (m), 139.94 – 139.39 (m), 136.09, 134.34, 130.69 – 130.22 (m), 129.91, 129.30 (d, *J* = 2.1), 127.49 (t, *J* = 2.1).

ASAP MS: R_t = 0.69 min; *m/z* (AI$^+$): 295.9 ([M(^{37}Cl)+H]$^+$, 45%), 293.9 ([M(^{35}Cl)+H]$^+$, 100%).

HRMS AP$^+$ Calculated for [M+H]$^+$ $C_{11}H_5ClF_4NS^+$ = 293.9767. Found = 293.9754.

MP 52 – 53 °C.

Synthesis of 2,3,5,6-tetrafluoro-4-((4′-nitrophenyl)thio)pyridine (14)

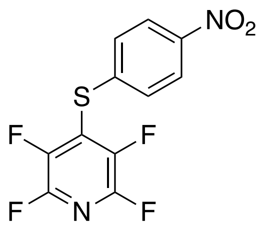

The title compound was synthesized according to the general procedure for the synthesis of tetrafluoropyridyl thioethers from 3.23 mmol of the corresponding thiophenol as bright yellow crystals (0.91 g) in 92% yield.

^{1}H NMR (400 MHz, $CDCl_3$) δ 8.22 (d, *J* = 9.0, 2H, Ar*H*), 7.54 (d, *J* = 9.0, 2H, Ar*H*).

^{19}F{^{1}H} NMR (376 MHz, $CDCl_3$) δ −88.30 – −88.52 (m), −134.07 – −134.26 (m).

^{13}C{^{1}H} NMR (101 MHz, $CDCl_3$) δ 147.65, 145.16 – 144.75 (m), 143.00 – 142.26 (m), 140.46 – 139.93 (m), 138.65, 130.93, 127.78 – 127.26 (m), 126.37, 124.64.

ASAP MS: R_t = 0.60 min; *m/z* (AI^+): 305.0 ($[M+H]^+$, 100%).

HRMS AP^+ Calculated for $[M+H]^+$ $C_{11}H_5F_4N_2O_2S^+$ = 305.0008. Found = 304.9999.

MP 96 – 97 °C.

Synthesis of 2,3,5,6-tetrafluoro-4-(4′-iodophenoxy)pyridine (15)

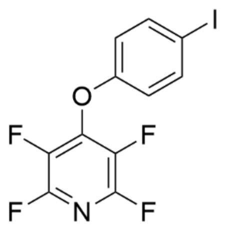

The title compound was synthesized according to the general procedure for the synthesis of tetrafluoropyridyl ethers from 2.27 mmol of the corresponding phenol as a white solid (0.835 g) in 99% yield.

^{1}H NMR (400 MHz, $CDCl_3$) δ 7.71 (d, *J* = 9.0, 2H, Ar*H*), 6.86 (d, *J* = 9.0, 2H, Ar*H*).

^{19}F{^{1}H} NMR (376 MHz, $CDCl_3$) δ −87.54 – −88.84 (m), −153.20 – −154.50 (m).

^{13}C{^{1}H} NMR (176 MHz, $CDCl_3$) δ 155.62, 144.98 – 144.70 (m), 143.95 – 143.66 (m), 143.57 – 143.31 (m), 138.99, 136.99 – 136.64 (m), 135.46 – 135.15 (m), 118.74, 88.39.

HRMS ESI^- Calculated for $[M-H]^-$ $C_{11}H_3NOF_4I^-$ = 367.9196. Found = 367.9203.

MP 86 – 87 °C.

Synthesis of 2,3,5,6-tetrachloro-4-(4′-chlorophenoxy)pyridine (16)

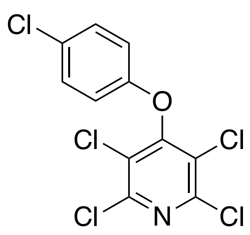

The title compound was synthesized according to the general procedure for the synthesis of tetrachloropyridyl ethers from 1.56 mmol of the corresponding phenol. Purified by flash column chromatography 100% hexanes to 90% hexanes 10% EtOAc. This gave the product as a white crystalline solid (0.426 g) in 80% yield.

^{1}H NMR (400 MHz, $CDCl_3$) δ 7.33 (d, *J* = 9.1, 2H, Ar*H*), 6.82 (d, *J* = 9.1, 2H, Ar*H*).

^{13}C{^{1}H} NMR (101 MHz, $CDCl_3$) δ 156.99, 153.70, 147.36, 130.07, 129.33, 125.48, 116.80.

HRMS ESI^{-} Calculated for $[M-H]^{-}$ $C_{11}H_3NOCl_5^{-}$ = 339.8657. Found = 339.8656.

MP 125 – 126 °C.

Synthesis of 2,3,5,6-tetrachloro-4-(4′-bromophenoxy)pyridine (17)

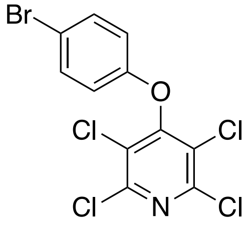

The title compound was synthesized according to the general procedure for the synthesis of tetrachloropyridyl ethers from 1.16 mmol of the corresponding phenol. Purified by flash column chromatography 100% hexanes to 90% hexanes 10% EtOAc. This gave the product as a white crystalline solid (0.391 g) in 88% yield.

^{1}H NMR (400 MHz, $CDCl_3$) δ 7.48 (d, *J* = 9.1, 2H, Ar*H*), 6.76 (d, *J* = 9.1, 2H, Ar*H*).

^{13}C{^{1}H} NMR (101 MHz, $CDCl_3$) δ 156.90, 154.24, 147.36, 133.03, 125.47, 117.22, 116.71.

HRMS AP^{+} Calculated for $[M+H]^{+}$ $C_{11}H_5NOCl_4Br^{+}$ = 385.8309. Found = 385.8300.

MP 137 °C.

Synthesis of 2,3,5,6-tetrachloro-4-(4′-nitrophenoxy)pyridine (18)

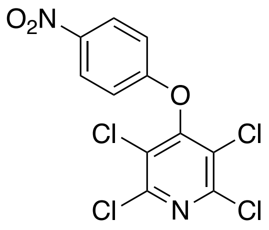

The title compound was synthesized according to the general procedure for the synthesis of tetrachloropyridyl ethers from 1.44 mmol of the corresponding phenol. Purified by flash column chromatography 100% hexanes to 90% hexanes 10% EtOAc. This gave the product as a white crystalline solid (0.426 g) in 46% yield.

^{1}H NMR (400 MHz, $CDCl_3$) δ 8.29 (d, J = 9.3, 2H, ArH), 7.00 (d, J = 9.3, 2H, ArH). *Note a small amount of an unknown impurity was observed in the spectra*

$^{13}C\{^{1}H\}$ NMR (101 MHz, $CDCl_3$) δ 159.19, 155.96, 147.60, 144.07, 126.34, 125.28, 115.78.

HRMS ESI$^-$ Calculated for $[M-H]^-$ $C_{11}H_3N_2O_3Cl_4^-$ = 350.8898. Found = 350.8902.

MP 163 – 164 °C.

Synthesis of 2,3,5,6-tetrachloro-4-(4′-methoxyphenoxy)pyridine (19)

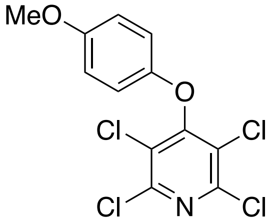

The title compound was synthesized according to the general procedure for the synthesis of tetrachloropyridyl ethers from 1.61 mmol of the corresponding phenol. Purified by flash column chromatography 100% hexanes to 90% hexanes 10% EtOAc. This gave the product as a white crystalline solid (0.431 g) in 79% yield.

^{1}H NMR (400 MHz, $CDCl_3$) δ 6.88 (d, J = 9.3, 2H, ArH), 6.81 (d, J = 9.3, 2H, ArH), 3.81 (s, 3H, CH_3).

$^{13}C\{^{1}H\}$ NMR (101 MHz, $CDCl_3$) δ 157.88, 156.03, 149.29, 147.21, 125.52, 116.59, 114.95, 55.70.

HRMS ESI$^+$ Calculated for $[M+H]^+$ $C_{12}H_8NO_2Cl_4^+$ = 337.9309. Found = 337.9317.

MP 138 – 139 °C.

Synthesis of 2,3,5,6-tetrachloro-4-((4′-chlorophenyl)thio)pyridine (20)

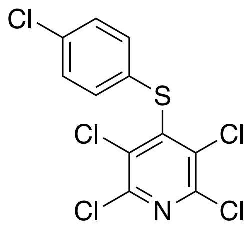

The title compound was synthesized according to the general procedure for the synthesis of tetrachloropyridyl ethers from 1.38 mmol of the corresponding thiophenol. Purified by flash column chromatography 100% hexanes to 90% hexanes 10% EtOAc. This gave the product as a white crystalline solid (0.080 g) in 16% yield.

^{1}H NMR (400 MHz, $CDCl_3$) δ 7.33 (d, *J* = 8.7, 2H, Ar*H*), 7.23 (d, *J* = 8.7, 2H, Ar*H*).

^{13}C{^{1}H} NMR (101 MHz, $CDCl_3$) δ 147.25, 146.76, 134.62, 133.85, 131.95, 130.40, 129.84.

HRMS AP$^+$ Calculated for $[M+H]^+$ $C_{11}H_5NSCl_5^+$ = 357.8585. Found = 357.8588.

MP 131 – 132 °C.

Synthesis of 2,3,5,6-tetrachloro-4-((4′-bromophenyl)thio)pyridine (21)

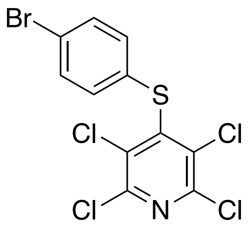

The title compound was synthesized according to the general procedure for the synthesis of tetrachloropyridyl ethers from 1.06 mmol of the corresponding thiophenol. Purified by flash column chromatography 100% hexanes to 90% hexanes 10% EtOAc. This gave the product as a white crystalline solid (0.092 g) in 22% yield.

^{1}H NMR (400 MHz, $CDCl_3$) δ 7.48 (d, *J* = 8.6, 2H, Ar*H*), 7.16 (d, *J* = 8.6, 2H, Ar*H*).

^{13}C{^{1}H} NMR (101 MHz, $CDCl_3$) δ 147.09, 146.77, 133.89, 132.78, 132.04, 131.11, 122.58.

HRMS AP$^+$ Calculated for $[M+H]^+$ $C_{11}H_5NSCl_4Br^+$ = 401.8080. Found = 401.8090.

MP 147 – 148 °C.

Synthesis of 2,3,5,6-tetrachloro-4-((4′-methoxyphenyl)thio)pyridine (22)

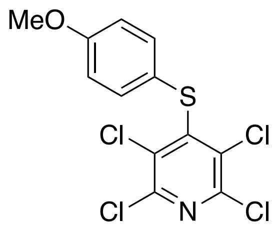

The title compound was synthesized according to the general procedure for the synthesis of tetrachloropyridyl ethers from 1.43 mmol of the corresponding thiophenol. Purified by flash column chromatography 100%

hexanes to 90% hexanes 10% EtOAc. This gave the product as a white crystalline solid (0.416 g) in 82% yield.

^{1}H NMR (400 MHz, $CDCl_3$) δ 7.33 (d, *J* = 8.9, 2H, Ar*H*), 6.87 (d, *J* = 8.9, 2H, Ar*H*), 3.82 (s, 3H, CH_3).

^{13}C{^{1}H} NMR (101 MHz, $CDCl_3$) δ 160.28, 148.94, 146.51, 134.05, 133.28, 121.95, 115.15, 55.43.

HRMS ESI$^+$ Calculated for $[M+H]^+$ $C_{12}H_8NOSCl_4^+$ = 353.9081. Found = 353.9089.

MP 128 – 129 °C.

Synthesis of 2,3,5,6-tetrafluoro-*N*-(*p*-tolyl)pyridin-4-amine (23)

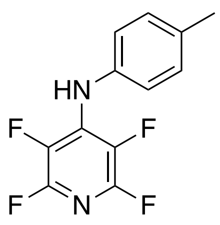

To a solution of 4-methylaniline (0.243 g, 2.27 mmol) in MeCN (3 mL) was added pentafluoropyridine (0.320 g, 1.89 mmol) and K_2CO_3 (0.506 g, 4.54 mmol). The resulting solution was heated to reflux overnight. The solution was then filtered and concentrated under reduced pressure. The recovered material was purified by flash column chromatography (100% hexanes to 90% hexanes 10% EtOAc. This gave the desired product as a brown crystalline solid (0.038 g) in 8% yield.

^{1}H NMR (400 MHz, $CDCl_3$) δ 7.19 (d, *J* = 8.0, 2H, Ar*H*), 7.05 (d, *J* = 8.0, 2H, Ar*H*), 6.30 (brs, 1H, N*H*), 2.39 (s, 3H, CH_3).

^{19}F{^{1}H} NMR (376 MHz, $CDCl_3$) δ −92.68 – −92.91 (m), −156.49 – −156.70 (m).

^{13}C{^{1}H} NMR (101 MHz, $CDCl_3$) δ 145.73 – 145.12 (m), 143.45 – 142.67 (m), 135.71, 135.51, 134.98 – 134.38 (m), 133.91 – 133.13 (m), 131.45 – 130.69 (m), 129.67, 122.46 (t, *J* = 1.8), 20.95.

HRMS ESI$^-$ Calculated for $[M-H]^-$ $C_{12}H_7N_2F_4^-$ = 255.0545. Found = 255.0547.

MP 98 – 99 °C.

Synthesis of 2,3,5,6-tetrachloro-*N*-(*p*-tolyl)pyridine-4-amine (24)

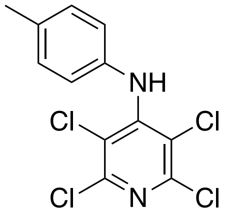

To a solution of 4-methylaniline (0.200 g, 1.87 mmol) and pentachloropyridine (0.373 g, 1.48 mmol) in MeCN (3 Ml) in a sealed tube was added Cs_2CO_3 (0.609 g, 1.87 mmol) and the tube heated to 100 °C for 16h. After this time the reaction mixture was filtered and concentrated under reduced pressure. The residue was then purified by flash column chromatography 100% hexanes to 100% EtOAc. This gave the product as a white crystalline solid (0.052 g) in 9% yield.

^{1}H NMR (400 MHz, $CDCl_3$) δ 7.16 (d, *J* = 8.1, 2H, Ar*H*), 6.90 (d, *J* = 8.1, 2H, Ar*H*), 6.69 (brs, 1H, N*H*), 2.38 (s, 3H, C*H*$_3$).

^{13}C{^{1}H} NMR (101 MHz, $CDCl_3$) δ 147.62, 146.66, 136.45, 135.32, 129.58, 122.64, 118.17, 20.96.

HRMS ESI$^+$ Calculated for $[M+H]^+$ $C_{12}H_9N_2Cl_4^+$ = 320.9520. Found = 320.9521.

MP 129 – 130 °C.

Synthesis of 2,3,5,6-tetrafluoro-*N*-methyl-*N*-(*p*-tolyl)pyridine-4-amine (25)

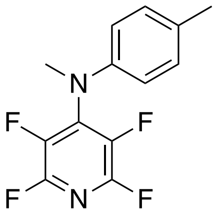

To a pressure tube was added *N*-methyl-*p*-toluidine (0.30 g, 2.48 mmol), pentafluoropyridine (0.440 g, 2.60 mmol) and Cs_2CO_3 (0.889 g, 2.73 mmol) and MeCN (2 mL). The reaction mixture was heated at 120 °C for 16 h. After this time the reaction mixture was filtered, and the filtrate washed with MeCN (20 mL). The solution was then concentrated to give the product as a yellow crystalline solid (0.421 g) in 63% yield.

^{1}H NMR (400 MHz, $CDCl_3$) δ 7.16 (d, *J* = 8.2, 2H, Ar*H*), 6.95 (d, *J* = 8.2, 2H, Ar*H*), 3.51 (t, *J* = 2.0, 3H, NC*H*$_3$), 2.36 (s, 3H, ArC*H*$_3$).

^{19}F{^{1}H} NMR (376 MHz, $CDCl_3$) δ −92.02 – −92.26 (m), −148.52 – −148.73 (m).

^{13}C{^{1}H} NMR (101 MHz, $CDCl_3$) δ 146.17 – 145.68 (m), 143.68, 143.60 – 143.33 (m), 138.31 – 137.81 (m), 135.77 – 135.22 (m), 133.43, 129.93, 119.21, 40.76 (t, *J* = 4.4), 20.71.

HRMS ESI$^-$ Calculated for $[M+H]^+$ $C_{13}H_{11}N_2F_4^+$ = 271.0858. Found = 271.0829.

Synthesis of 2,3,5,6-tetrafluoro-4-(naphthalen-2′-yloxy)pyridine (26)

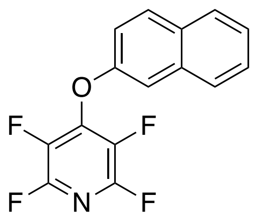

The title compound was synthesized according to the general procedure for the synthesis of tetrafluoropyridyl ethers from 3.47 mmol of the corresponding naphthol as a white crystalline solid (0.987 g) in 97% yield.

^{1}H NMR (400 MHz, $CDCl_3$) δ 7.94 – 7.87 (m, 2H, Ar*H*), 7.78 (App d, *J* = 8.1, 1H, Ar*H*), 7.59 – 7.48 (m, 2H, Ar*H*), 7.39 – 7.32 (m, 2H, Ar*H*).

^{19}F{^{1}H} NMR (376 MHz, $CDCl_3$) δ −88.30 – −88.51 (m), −153.96 – −154.17 (m).

^{13}C{^{1}H} NMR (176 MHz, $CDCl_3$) δ 153.53, 145.03 – 144.78 (m), 144.41 (dt, *J* = 10.7, 5.5 Hz), 143.66 – 143.39 (m), 137.18 – 136.88 (m), 135.68 – 135.41 (m), 133.68, 130.86, 130.46, 127.87, 127.30, 127.24, 125.72, 117.20, 112.21.

IR ν_{max} (ATR)/cm^{-1} 1647, 1628, 1480, 1208, 1153, 1124, 1061, 976, 817, 747, 472.

HRMS ESI$^-$ Calculated for $[M{-}H]^-$ $C_{15}H_6NOF_4^-$ = 292.0386. Found = 292.0376.

MP 94 – 95 °C.

Synthesis of 2,3,5,6-tetrafluoro-4-[(1′-iodonaphthalen-2′-yl)oxy]pyridine (27)

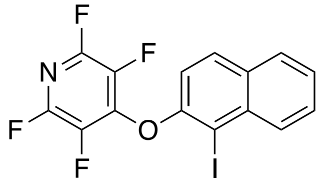

To a stirred solution of TFP ether (1.00 equiv.) in TFA (2 mL) was added NIS (1.10 equiv.) and the resulting solution stirred for 1 h at rt. After this time the reaction mixture was concentrated under reduced pressure. The resulting residue was taken up in EtOAc (30 mL) and washed with 1 M sodium thiosulfate solution. The organic fraction was then dried over $MgSO_4$ and concentrated under reduced pressure. The residue was purified by flash column chromatography.

Iodination was carried out as detailed in the general procedure for iodination. Following flash column chromatography, the recovered solid was recrystallized from hexanes. The reaction was carried out on 0.68 mmol of the corresponding tetrafluoropyridyl phenol this gave the product as a white crystalline solid in a 62% (0.174 g) yield.

^{1}H NMR (400 MHz, $CDCl_3$) δ 8.27 – 8.21 (m, 1H, Ar*H*), 7.90 – 7.83 (m, 2H, Ar*H*), 7.71 – 7.65 (m, 1H, Ar*H*), 7.61 – 7.54 (m, 1H, Ar*H*), 7.18 (d, *J* = 8.9, 1H, Ar*H*).

$^{19}F\{^{1}H\}$ NMR (376 MHz, $CDCl_3$) δ −88.40 – −88.61 (m), −155.17 – −155.37 (m).

$^{13}C\{^{1}H\}$ NMR (101 MHz, $CDCl_3$) δ 152.64, 145.69 – 145.17 (m), 144.50 – 144.01 (m), 143.28 – 142.77 (m), 137.72 – 137.25 (m), 136.09, 135.34, 135.10 – 134.64 (m), 132.45, 129.12, 127.69, 125.68, 121.75, 111.14, 94.38.

IR ν_{max} (ATR)/cm^{-1} 1642, 1473, 1203, 984, 961, 815, 762, 521.

HRMS ESI^{-} Calculated for $[M{-}H]^{-}$ $C_{15}H_5NOF_4{}^{127}I^{-}$ = 417.9352. Found = 417.9363.

MP 121 – 123 °C.

Synthesis of 2′-((2,3,5,6-tetrafluoropyridin-4′′-yl)oxy)-[1,1′-binaphthalen]-2-ol (28)

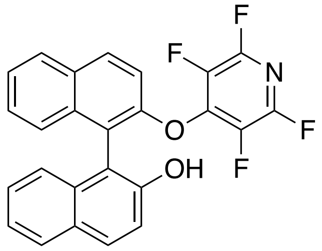

The title compound was synthesized according to the general procedure for the synthesis of tetrafluoropyridyl ethers from 1.75 mmol of *rac*-BINOL, pentafluoropyridine (1.57 mmol) and K_2CO_3 (1.75 mmol). The recovered material was purified using flash column chromatography (100% hexanes to 50% hexanes 50% toluene) as a clear crystalline solid (0.479 g) in 70% yield.

^{1}H NMR (400 MHz, $CDCl_3$) δ 8.14 (d, *J* = 9.0 , 1H, Ar*H*), 8.04 (d, *J* = 8.3, 1H, Ar*H*), 7.86 (d, *J* = 8.9, 1H, Ar*H*), 7.81 (d, *J* = 7.9, 1H, Ar*H*), 7.65 – 7.53 (m, 2H, Ar*H*), 7.46 – 7.41 (m, 1H, Ar*H*), 7.40 – 7.32 (m, 2H, Ar*H*), 7.30 – 7.27 (m, 1H, Ar*H*), 7.25 (d, *J* = 8.9, 1H, Ar*H*), 7.03 (d, *J* = 8.3 Hz, 1H, Ar*H*), 4.90 (brs, 1H, O*H*).

$^{19}F\{^{1}H\}$ NMR (376 MHz, $CDCl_3$) δ −90.13 – −90.34 (m), −154.35 – −154.55 (m).

$^{13}C\{^{1}H\}$ NMR (101 MHz, $CDCl_3$) δ 152.84, 151.81, 144.90 – 144.28 (m), 142.42 – 141.88 (m), 136.54 – 135.98 (m), 133.94 – 133.40 (m), 132.94, 131.79, 131.64, 131.01, 128.62, 128.50, 128.15, 127.06, 126.52, 125.64, 123.95, 123.89, 119.10, 118.94, 117.36, 112.14.

HRMS ESI^{-} Calculated for $[M{-}H]^{-}$ $C_{25}H_{12}NO_2F_4{}^{-}$ = 434.0804. Found = 434.0783.

MP 154 – 155 °C.

Synthesis of (*S*)-2,2′-bis((2′,3′,5′,6′-tetrafluoropyridin-4″-yl)oxy)-1,1′-binaphthalene (29)

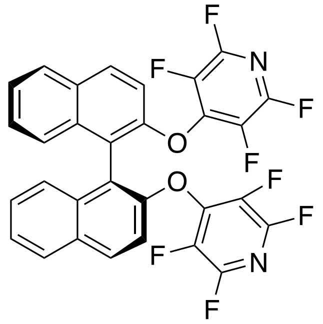

The title compound was synthesized according to the general procedure for the synthesis of tetrafluoropyridyl ethers from 0.70 mmol of (*S*)-BINOL, pentafluoropyridine (1.47 mmol) and K_2CO_3 (1.47 mmol). The solution was then passed through a short silica plug (100% EtOAc) to give the desired product as a clear crystalline solid (0.397 g) in 97% yield.

^{1}H NMR (400 MHz, $CDCl_3$) δ 8.02 (d, *J* = 9.0, 2H, Ar*H*), 7.93 (d, *J* = 8.3, 2H, Ar*H*), 7.50 (m, 4H, Ar*H*), 7.40 – 7.34 (m, 2H, Ar*H*), 7.23 (d, *J* = 8.3, 2H, Ar*H*).

^{19}F{^{1}H} NMR (376 MHz, $CDCl_3$) δ −89.74 – −89.96 (m), −154.03 – −154.25 (m).

^{13}C{^{1}H} NMR (101 MHz, $CDCl_3$) δ 152.25, 144.88 – 144.19 (m), 142.58 – 141.89 (m), 136.70 – 136.02 (m), 134.13 – 133.40 (m), 132.90, 131.28, 130.86, 128.30, 127.79, 126.26, 124.99, 119.36, 117.93.

HRMS ESI$^-$ Calculated for $[M-H]^-$ $C_{30}H_{11}N_2O_2F_8^-$ = 583.0692. Found = 583.0606.

MP 210 – 211 °C.

Synthesis of 1,4-bis((2,3,5,6-tetrafluoropyridin-4′-yl)oxy)benzene (30)

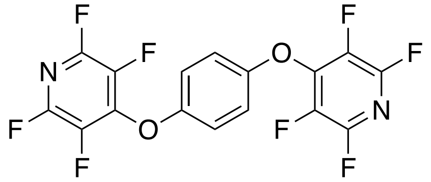

The title compound was synthesized according to the general procedure for the synthesis of tetrafluoropyridyl ethers from 2.50 mmol of hydroquinone, pentafluoropyridine (5.50 mmol) and K_2CO_3 (5.50 mmol) as a white crystalline solid (0.987 g) in 97% yield.

^{1}H NMR (400 MHz, $CDCl_3$) δ 7.13 (s, 4H, Ar*H*).

^{19}F{^{1}H} NMR (376 MHz, $CDCl_3$) δ −88.07 – −88.29 (m), −154.19 – −154.45 (m).

^{13}C{^{1}H} NMR (101 MHz, $CDCl_3$) δ 152.65, 145.71 – 145.09 (m), 144.54 – 143.95 (m), 143.23 – 142.68 (m), 137.70 – 136.74 (m), 134.95 – 134.35 (m), 118.48.

HRMS ESI$^-$ Calculated for $[M-H]^-$ $C_{16}H_3N_2O_2F_8^-$ = 407.0067. Found = 407.0075.

MP 128 – 129 °C.

Synthesis of 2,3,5,6-tetrafluoro-4-({4″-methoxy-[1′,1″-biphenyl]-3′-yl}oxy)pyridine (31)

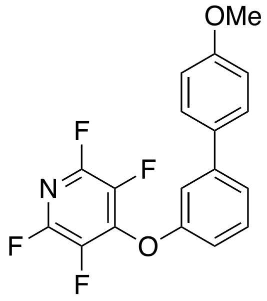

A solution of tetrafluoropyridyl-iodo-phenol (1.00 equiv.) and corresponding boronic acid were dissolved in 1,4-dioxane (10 mL) and the solution degassed by bubbling nitrogen for 1 h. At the same time a solution of K_2CO_3 (3.00 equiv.) in water was also degassed by bubbling nitrogen for 1 h. After this time, $Pd(PPh_3)_4$ (5 mol%) was added to the dioxane solution and the resulting mixture degassed for a further 10 min. The K_2CO_3 water solution was then transferred *via* syringe to the dioxane solution and the resulting mixture was heated at reflux for 12 h. The reaction mixture was then cooled and concentrated under reduced pressure. The resulting residue was taken up in EtOAc (50 mL) and washed with H_2O (25 mL), the organic layer was dried over $MgSO_4$, filtered and concentrated. The recovered residue was then subjected to flash column chromatography (100% hexanes to 90% hexanes 20% EtOAc).

The title compound was synthesized according to the general procedure for the for Suzuki-Miyaura cross-coupling from 0.27 mmol of the corresponding tetrafluoropyridyl phenol iodide as a white crystalline solid (0.085 g) in 91% yield.

^{1}H NMR (400 MHz, $CDCl_3$) δ 7.52 (d, *J* = 8.9, 2H, Ar*H*), 7.48 – 7.40 (m, 2H, Ar*H*), 7.28 – 7.23 (m, 1H, Ar*H*), 7.06 – 6.96 (m, 3H, Ar*H*), 3.88 (s, 3H, CH_3).

^{19}F{^{1}H} NMR (376 MHz, $CDCl_3$) δ −88.41 – −88.64 (m), −154.04 – −154.24 (m).

^{13}C{^{1}H} NMR (176 MHz, $CDCl_3$) δ 159.70, 156.19, 144.99 – 144.76 (m), 144.51 – 144.31 (m), 143.60 – 143.37 (m), 143.25, 137.08 – 136.80 (m), 135.57 – 135.31 (m), 132.18, 130.20, 128.21, 123.50, 114.99, 114.53, 114.34, 55.36.

IR ν_{max} (ATR)/cm^{-1} 3034, 2844, 1603, 1581, 1462, 1282, 966, 837, 782.

HRMS ESI$^-$ Calculated for $[M-H]^-$ $C_{18}H_{10}NO_2F_4^-$ = 348.0648. Found = 348.0631.

MP 98 – 100 °C.

Synthesis of 4,4'-((propane-2,2-diylbis(4,1-phenylene))bis(oxy))bis(2,3,5,6-tetrafluoropyridine) (32)

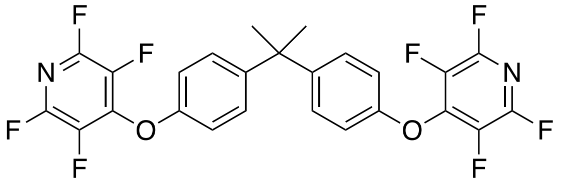

The title compound was synthesized according to the general procedure for the synthesis of tetrafluoropyridyl ethers from 13.2 mmol of bisphenol A, pentafluoropyridine (27.6 mmol) and K_2CO_3 (27.6 mmol). The recovered material was purified using flash column chromatography (100% hexanes to 100% toluene) as a clear crystalline solid (6.60 g) in 95% yield.

^{1}H NMR (400 MHz, $CDCl_3$) δ 7.25 (d, *J* = 8.9, 4H, Ar*H*), 7.00 (d, *J* = 8.9, 4H, Ar*H*), 1.71 (s, 6H, C*H₃*).

^{19}F{^{1}H} NMR (376 MHz, $CDCl_3$) δ −88.21 – −89.29 (m), −153.66 – −155.02 (m).

^{13}C{^{1}H} NMR (101 MHz, $CDCl_3$) δ 153.87, 147.24, 145.65 – 145.11 (m), 144.74 – 144.29 (m), 137.83 – 137.20 (m), 135.19 – 134.63 (m), 128.32, 116.27, 42.39, 30.89.

HRMS ESI$^-$ Calculated for $[M{-}H]^-$ $C_{25}H_{13}N_2O_2F_8^-$ = 525.0849. Found = 525.0851.

MP 86 – 88 °C.

Synthesis of 4,4’-((sulfonylbis(4,1-phenylene))bis(oxy))bis(2,3,5,6-tetrafluoropyridine) (33)

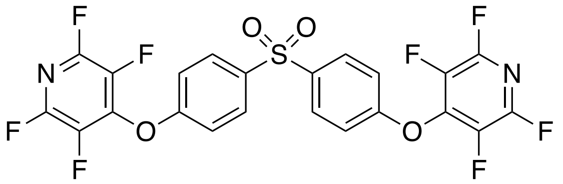

The title compound was synthesized according to the general procedure for the synthesis of tetrafluoropyridyl ethers from 0.4 mmol of 4,4’-sulfonyldiphenol, pentafluoropyridine (0.88 mmol) and K_2CO_3 (0.88 mmol). This gave the desired product as a clear crystalline solid (0.199 g) in 91% yield.

^{1}H NMR (400 MHz, $CDCl_3$) δ 8.00 (d, *J* = 8.9, 4H, Ar*H*), 7.18 (d, *J* = 8.9, 4H, Ar*H*).

^{19}F{^{1}H} NMR (376 MHz, $CDCl_3$) δ −86.87 – −87.08 (m), −152.92 – −153.13 (m).

^{13}C{^{1}H} NMR (101 MHz, $CDCl_3$) δ 158.89, 145.68 – 145.03 (m), 143.23 – 142.40 (m), 137.84, 137.70 – 137.05 (m), 135.24 – 134.47 (m), 130.32, 117.06.

HRMS ESI$^-$ Calculated for $[M-H]^-$ $C_{22}H_7N_2O_4F_8S^-$ = 546.9999. Found = 546.9976.

MP 181 – 182 °C.

Synthesis of 2,6-diiodo-4-(2′-{4″-[(2‴,3‴,5‴,6‴-tetrafluoropyridin-4″-yl)oxy]phenyl}propan-2′-yl)phenol (34)

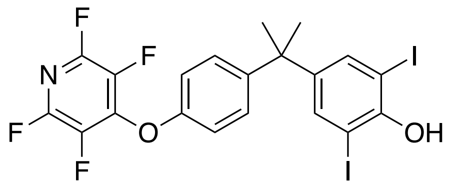

To a solution of 2-iodo-4-(2-{4-[(2,3,5,6-tetrafluoropyridin-4-yl)oxy]phenyl}propan-2-yl)phenol (0.600 g 1.19 mmol) in TFA (1 mL) was added NIS (0.295 g, 1.31 mmol) and the resulting reaction mixture stirred at room temperature for 3 h. After this time the reaction mixture was concentrated under reduced pressure and then directly to flash column chromatography (100% hexanes to 100% toluene). This gave the target compound as a colorless crystalline solid (0.425 g) in a 57% yield.

^{1}H NMR (400 MHz, $CDCl_3$) δ 7.50 (s, 2H, Ar*H*), 7.22 (d, *J* = 9.0, 2H, Ar*H*), 7.00 (d, *J* = 9.0, 2H, Ar*H*), 5.66 (brs, 1H, O*H*), 1.64 (s, 6H, CH_3).

^{19}F{^{1}H} NMR (376 MHz, $CDCl_3$) δ −88.50 – −88.71 (m), −154.09 – −154.30 (m).

^{13}C{^{1}H} NMR (176 MHz, $CDCl_3$) δ 153.93, 151.66, 146.51, 146.40, 144.96 – 144.73 (m), 144.56 – 144.37 (m), 143.58 – 143.35 (m), 137.64, 137.03 – 136.77 (m), 135.53 – 135.28 (m), 128.19, 116.40, 82.11, 41.75, 30.81.

IR ν_{max} (ATR)/cm^{-1} 1643, 1591, 1489, 1248, 1076, 740.

HRMS ESI$^-$ Calculated for $[M-H]^-$ $C_{20}H_{12}NO_2F_4I_2^-$ = 627.8894. Found = 627.8912.

MP 141 – 143 °C.

Synthesis of 2,3,5,6-tetrafluoro-4-(4′-isopropyl-2′-methylphenoxy)pyridine (35)

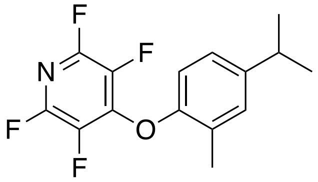

The title compound was synthesized according to the general procedure for the synthesis of tetrafluoropyridyl ethers from 1.33 mmol of carvacrol, pentafluoropyridine (1.40 mmol) and K_2CO_3 (1.40 mmol). The solution was filtered and concentrated to give the desired product as a clear crystalline solid (0.392 g) in 99% yield.

^{1}H NMR (400 MHz, $CDCl_3$) δ 7.21 (d, *J* = 7.8, 1H, Ar*H*), 7.04 (dd, *J* = 7.8, 1.6, 1H, Ar*H*), 6.72 (s, 1H, Ar*H*), 2.87 (hept, *J* = 6.9, 1H, ArC*H*(CH_3)$_2$), 2.34 (s, 3H, ArC*H*$_3$), 1.23 (d, *J* = 6.9, 6H, CH(C*H*$_3$)$_2$).

^{19}F{^{1}H} NMR (376 MHz, $CDCl_3$) δ −89.10 – −89.41 (m), −155.90 – −156.15 (m).

^{13}C{^{1}H} NMR (101 MHz, $CDCl_3$) δ 154.13, 148.75, 145.67 – 145.10 (m), 143.30 – 142.76 (m), 137.32 – 136.74 (m), 134.76 – 134.12 (m), 131.53, 125.18, 123.33, 114.23, 33.71, 23.87, 15.48.

HRMS ESI$^-$ Calculated for $[M-H]^-$ $C_{15}H_{12}NOF_4^-$ = 298.0855. Found = 298.0845.

MP 71 – 72 °C.

Synthesis of 2,3,5,6-tetrachloro-4-(4′-isopropyl-2′-methylphenoxy)pyridine (36)

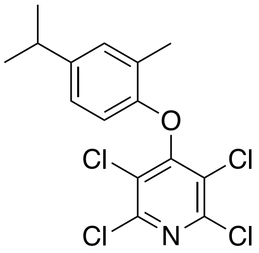

The title compound was synthesized according to the general procedure for the synthesis of tetrachloropyridyl ethers from 0.67 mmol of the corresponding phenol. Purified by flash column chromatography 100% hexanes to 90% hexanes 10% EtOAc. This gave the product as a clear crystalline solid (0.157 g) in 65% yield.

^{1}H NMR (400 MHz, $CDCl_3$) δ 7.22 (d, *J* = 7.7, 1H, Ar*H*), 6.96 (dd, *J* = 7.7, 1.5, 1H, Ar*H*), 6.19 (d, *J* = 1.5, 1H, Ar*H*), 2.78 (hept, *J* = 6.9, 1H, ArC*H*(CH_3)$_2$), 2.39 (s, 3H, ArCH_3), 1.18 (d, *J* = 6.9, 6H, CH(C*H*$_3$)$_2$).

^{13}C{^{1}H} NMR (101 MHz, $CDCl_3$) δ 158.11, 153.41, 148.50, 147.16, 131.63, 125.23, 124.43, 121.57, 111.47, 33.72, 23.92, 15.80.

HRMS ESI$^+$ Calculated for $[M+H]^+$ $C_{15}H_{14}NOCl_4^+$ = 363.9830. Found = 363.9832

MP 131 – 132 °C.

Synthesis of 2-((perfluoropyridin-4′-yl)oxy)benzaldehyde (37)

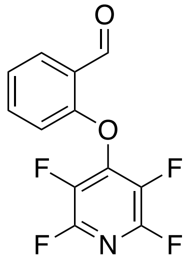

The title compound was synthesized according to the general procedure for the synthesis of tetrafluoropyridyl ethers from 1.64 mmol of salicylaldehyde, pentafluoropyridine (1.72 mmol) and K_2CO_3 (1.72 mmol). The solution was filtered and concentrated to give the desired product as a white crystalline solid (0.426 g) in 96% yield.

^{1}H NMR (400 MHz, $CDCl_3$) δ 10.51 (d, *J* = 0.6, 1H, ArC(O)*H*), 8.00 (dd, *J* = 7.7, 1.8, 1H, Ar*H*), 7.63 (ddd, *J* = 8.3, 7.4, 1.8, 1H, Ar*H*), 7.44 – 7.35 (m, 1H, Ar*H*), 7.01 – 6.94 (m, 1H, Ar*H*).

^{19}F{^{1}H} NMR (376 MHz, $CDCl_3$) δ −87.54 – −87.83 (m), −154.20 – −154.42 (m).

^{13}C{^{1}H} NMR (101 MHz, $CDCl_3$) δ 187.70, 145.59 – 145.18 (m), 143.89 – 143.56 (m), 143.18 – 142.75 (m), 137.56 – 137.08 (m), 135.86, 134.93 – 134.44 (m), 130.29, 126.04, 125.72, 116.43.

HRMS ESI$^+$ Calculated for $[M+H]^+$ $C_{12}H_6NO_2F_4^+$ = 272.0335. Found = 272.0324.

MP 105 – 106 °C.

Synthesis of 2,3,5,6-tetrachloro-4-(3′-fluorophenoxy)pyridine (38)

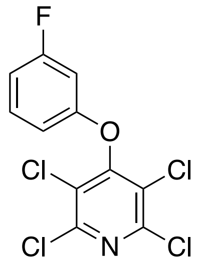

The title compound was synthesized according to the general procedure for the synthesis of tetrachloropyridyl ethers from 1.79 mmol of the corresponding phenol. Purified by flash column chromatography 100% hexanes to 90% hexanes 10% EtOAc. This gave the product as a brown crystalline solid (0.525 g) in 90% yield.

^{1}H NMR (400 MHz, $CDCl_3$) δ 7.38 – 7.26 (m, 1H, Ar*H*), 6.96 – 6.85 (m, 1H, Ar*H*), 6.73 – 6.58 (m, 2H, Ar*H*).

^{19}F{^{1}H} NMR (376 MHz, $CDCl_3$) δ −109.64 (s).

^{13}C{^{1}H} NMR (101 MHz, $CDCl_3$) δ 163.54 (d, *J* = 248.6), 156.79, 155.96 (d, *J* = 10.8), 147.36, 130.95 (d, *J* = 9.6), 125.53, 111.18 (d, *J* = 21.2), 110.91 (d, *J* = 3.3), 103.93 (d, *J* = 25.9).

HRMS ESI$^+$ Calculated for $[M+H]^+$ $C_{11}H_5NOFCl_4^+$ = 325.9109. Found = 325.9121.

MP 92 – 93 °C.

Synthesis of (4b*R*,8a*S*,9*S*)-3,7-dimethoxy-11-methyl-4-((2′,3′,5′,6′-tetrafluoropyridin-4′-yl)oxy)-9,10-dihydro-5*H*-9,4b-(epiminoethano)phenanthren-6(8a*H*)-one (39)

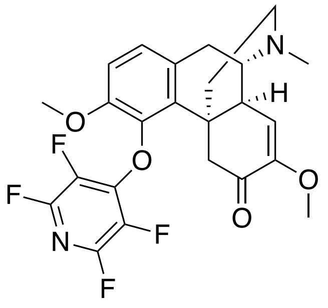

The title compound was synthesized according to the general procedure for the synthesis of tetrafluoropyridyl ethers from 0.054 mmol of sinomenine hydrochloride, pentafluoropyridine (0.060 mmol) and K_2CO_3 (0.119 mmol). The solution was then passed through a short silica plug (100% EtOAc) to give the desired product as a clear crystalline solid (0.026 g) in 99% yield.

^{1}H NMR (700 MHz, $CDCl_3$) δ 6.89 (d, *J* = 8.4, 1H), 6.70 (d, *J* = 8.4, 1H), 5.42 (s, 1H), 3.61 (d, *J* = 16.1, 1H), 3.58 (s, 1H), 3.44 (s, 3H), 3.23 (brs, 1H), 3.08 (brs, 1H), 3.02 (d, *J* = 18.2, 1H), 2.78 (brd, *J* = 17.3, 1H), 2.60 (brs, 1H), 2.51 (d, *J* = 16.2, 1H), 2.44 (s, 2H), 2.06 (brt, *J* = 11.4, 1H), 2.00 – 1.93 (m, 1H), 1.77 (d, *J* = 12.6, 1H).

^{19}F{^{1}H} NMR (376 MHz, $CDCl_3$) δ −90.17 – −90.42 (m), −91.14 – −91.41 (m), −156.65 – −157.08 (m), −160.69 – −160.96 (m).

^{13}C{^{1}H} NMR (101 MHz, $CDCl_3$) δ 191.98, 152.67, 142.76, 130.31, 128.67, 125.44, 114.69, 110.78, 56.36, 55.97, 54.97, 50.34, 46.74, 45.81, 42.69, 41.03, 36.90, 24.29, 22.87. Note: not all fluoropyridyl carbons could be observed due to their intensity.

HRMS ESI$^+$ Calculated for $[M-H]^+$ $C_{24}H_{23}N_2O_4F_4^+$ = 479.1594. Found = 479.1592.

MP 146 – 147 °C.

Synthesis of (1S,10R,11S,15S)-15-methyl-5-[(2′,3′,5′,6′-tetrafluoropyridin-4′-yl)oxy]tetracyclo[8.7.0.0^{2},^{7}.0^{11},^{15}]heptadeca-2(7),3,5-trien-14-one (40)

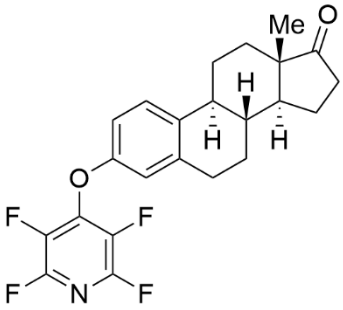

The title compound was synthesized according to the general procedure for the synthesis of tetrafluoropyridyl ethers from 1.85 mmol of estrone with the following modification. Due to the solubility of estrone in MeCN, 1 mL of DMF was added to the reaction mixture and the reaction mixture heated at 70 °C for 2 h. The reaction mixture was concentrated under reduced pressure and the residue purified by flash column chromatography (100% hexanes to 90% hexanes 10% EtOAc). This gave the title compound as a white crystalline solid (0.676 g) in 87% yield.

^{1}H NMR (400 MHz, $CDCl_3$) δ 7.30 (d, *J* = 8.6, 1H), 6.86 (dd, *J* = 8.6, 2.7, 1H), 6.80 (d, *J* = 2.7, 1H), 3.06 – 2.86 (m, 2H), 2.61 – 2.49 (m, 1H), 2.46 – 2.39 (m, 1H), 2.35 – 2.26 (m, 1H), 2.24 – 1.97 (m, 4H), 1.73 – 1.42 (m, 7H), 0.95 (s, 3H).

^{19}F{^{1}H} NMR (376 MHz, $CDCl_3$) δ −88.67 – −88.96 (m), −154.29 – −154.48 (m).

^{13}C{^{1}H} NMR (176 MHz, $CDCl_3$) δ 220.51, 153.85, 144.95 – 144.70 (m), 144.68 – 144.48 (m), 143.56 – 143.33 (m), 138.76, 137.08 – 136.83 (m), 136.73, 135.59 – 135.33 (m), 126.87, 116.52, 113.85, 50.38, 47.89, 44.00, 37.98, 35.80, 31.50, 29.45, 26.24, 25.77, 21.55, 13.81.

HRMS ESI$^-$ Calculated for $[M-H]^-$ $C_{23}H_{20}NO_2F_4^-$ = 418.1430. Found = 418.1432.

MP 153 – 154 °C.

Synthesis of (8*R*,9*S*,13*S*,14*S*)-13-methyl-3-((2′,3′,5′,6′-tetrafluoropyridin-4′-yl)oxy)-6,7,8,9,11,12,13,14,15,16-decahydro-17*H*-cyclopenta[*a*]phenanthren-17-one (41)

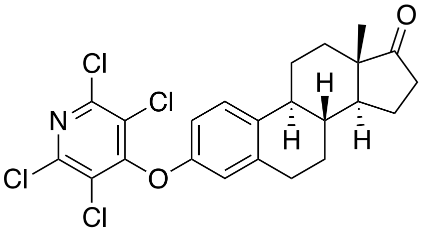

The title compound was synthesized according to the general procedure for the synthesis of tetrachloropyridyl ethers from 0.37 mmol of the corresponding phenol. Purified by flash

column chromatography 100% hexanes to 50% hexanes 50% EtOAc. This gave the product as a white crystalline solid (0.120 g) in 67% yield.

^{1}H NMR (400 MHz, $CDCl_3$) δ 7.26 (d, *J* = 8.5, 1H), 6.64 (dd, *J* = 8.5, 2.8, 1H), 6.57 (d, *J* = 2.8, 1H), 2.94 – 2.86 (m, 2H), 2.53 (dd, *J* = 18.9, 8.5, 1H), 2.44 – 2.37 (m, 1H), 2.29 (td, *J* = 10.7, 4.4, 1H), 2.23 – 1.95 (m, 4H), 1.72 – 1.41 (m, 6H), 0.94 (s, 3H).

^{13}C{^{1}H} NMR (101 MHz, $CDCl_3$) δ 157.50, 153.26, 147.16, 138.80, 135.59, 126.93, 125.71, 115.24, 112.68, 50.43, 47.95, 44.02, 38.05, 35.86, 31.55, 29.54, 26.32, 25.79, 21.60, 13.86.

HRMS ESI$^+$ Calculated for $[M+H]^+$ $C_{23}H_{22}NO_2Cl_4^+$ = 484.0405. Found = 484.0409.

MP 211 – 213 °C.

Synthesis of (1S,10R,11S,15S)-15-methyl-5-[(2′,3′,5′,6′-tetrafluoropyridin-4′-yl)oxy]tetracyclo[8.7.0.0^{2},^{7}.0^{11},^{15}]heptadeca-2(7),3,5-trien-14-ol (42)

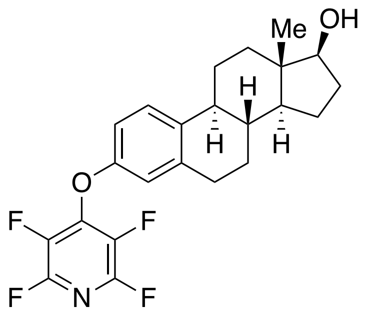

To a solution of TFP protected **40** (0.10 g, 0.24 mmol) in THF (10 mL) was added sodium borohydride (0.023 g, 0.60 mmol) and the reaction mixture stirred at rt for 24 h. The reaction mixture was quenched by the addition of water (5 mL) and then concentrated under reduced pressure. The recovered residue was taken up in EtOAc (30 mL) and washed with water (30 mL) and then brine (30 mL). The organic fraction was dried over $MgSO_4$, filtered and concentrated under reduced pressure. This gave the title compound as a white crystalline solid (0.091 g) in 90% yield.

^{1}H NMR (600 MHz, $CDCl_3$) δ 7.26 (d, *J* = 8.6, 1H, Ar*H*), 6.80 (dd, *J* = 8.6, 2.8, 1H, Ar*H*), 6.74 (d, *J* = 2.8, 1H, Ar*H*), 3.73 (t, *J* = 8.5 Hz, 1H, C*H*), 2.89 – 2.79 (m, 2H, C*H*$_2$), 2.34 – 2.27 (m, 1H, C*H*), 2.25 – 2.17 (m, 1H, C*H*), 2.16 – 2.08 (m, 1H, C*H*), 1.96 (dt, *J* = 12.6, 3.4, 2H, C*H*$_2$), 1.92 – 1.86 (m, 1H, C*H*), 1.75 – 1.65 (m, 1H, C*H*), 1.55 – 1.16 (m, 8H), 0.78 (s, 3H, C*H*$_3$).

^{19}F{^{1}H} NMR (376 MHz, $CDCl_3$) δ −88.82 – −89.09 (m), −154.29 – −154.55 (m).

$^{13}C\{^1H\}$ NMR (151 MHz, $CDCl_3$) δ 153.72, 145.09 – 144.80 (m), 144.79 – 144.51 (m), 143.50 – 143.16 (m), 138.98, 137.35, 137.26 – 136.94 (m), 135.52 – 135.17 (m), 126.84, 116.47, 113.68, 81.78, 50.02, 43.98, 43.18, 38.45, 36.61, 30.55, 29.58, 26.93, 26.17, 23.09, 11.02.

HRMS ESI⁻ Calculated for $[M-H]^-$ $C_{23}H_{22}NO_2F_4^-$= 420.1587. Found = 420.1585.

MP 99 – 101 °C.

Synthesis of (8*R*,9*S*,13*S*,14*S*,17*S*)-13-methyl-3-((2′,3′,5′,6′-tetrachloropyridin-4′-yl)oxy)-7,8,9,11,12,13,14,15,16,17-decahydro-6*H*-cyclopenta[*a*]phenanthren-17-ol (43)

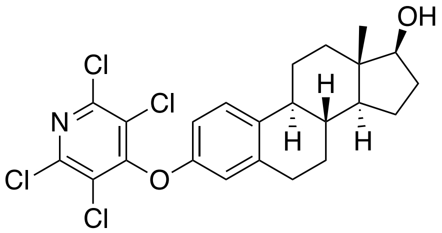

To a solution of compound **41** (0.023 g, 0.048 mmol) in THF (3 ml) was added $NaBH_4$ (0.005 g, 0.12 mmol) and the resulting mixture stirred at RT for 5 h. After this time the reaction mixture was quenched by the addition of water (5 mL). The resulting solution was concentrated under reduced pressure before being diluted with EtOAc (30 mL). This was then washed with water (50 mL) and brine (50 mL). The organic fraction was dried over $NaSO_4$, filtered, and then concentrated under reduced pressure to give the desired product as a white crystalline solid (0.021 g, 90%).

^1H NMR (400 MHz, $CDCl_3$) δ 7.25 (d, *J* = 8.5, 1H), 6.62 (dd, *J* = 8.5, 2.8, 1H), 6.56 (d, *J* = 2.8, 1H), 3.76 (t, *J* = 8.5, 1H), 2.94 – 2.78 (m, 2H), 2.37 – 2.28 (m, 1H), 2.27 – 2.10 (m, 2H), 1.98 (dt, *J* = 12.5, 3.5, 1H), 1.94 – 1.87 (m, 1H), 1.79 – 1.65 (m, 1H), 1.58 – 1.18 (m, 8H), 0.81 (s, 3H).

$^{13}C\{^1H\}$ NMR (101 MHz, $CDCl_3$) δ 157.57, 153.13, 147.14, 139.03, 136.20, 126.90, 125.73, 115.21, 112.50, 81.84, 50.07, 44.00, 43.24, 38.53, 36.67, 30.60, 29.68, 27.02, 26.20, 23.14, 11.08.

HRMS ESI⁺ Calculated for $[M+H]^+$ $C_{23}H_{24}NO_2Cl_4^+$ = 486.0561. Found = 486.0551.

MP 204 – 206 °C.

NMR Data for Synthesized Compounds

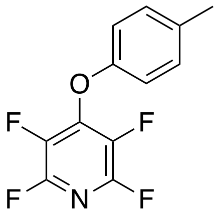

1

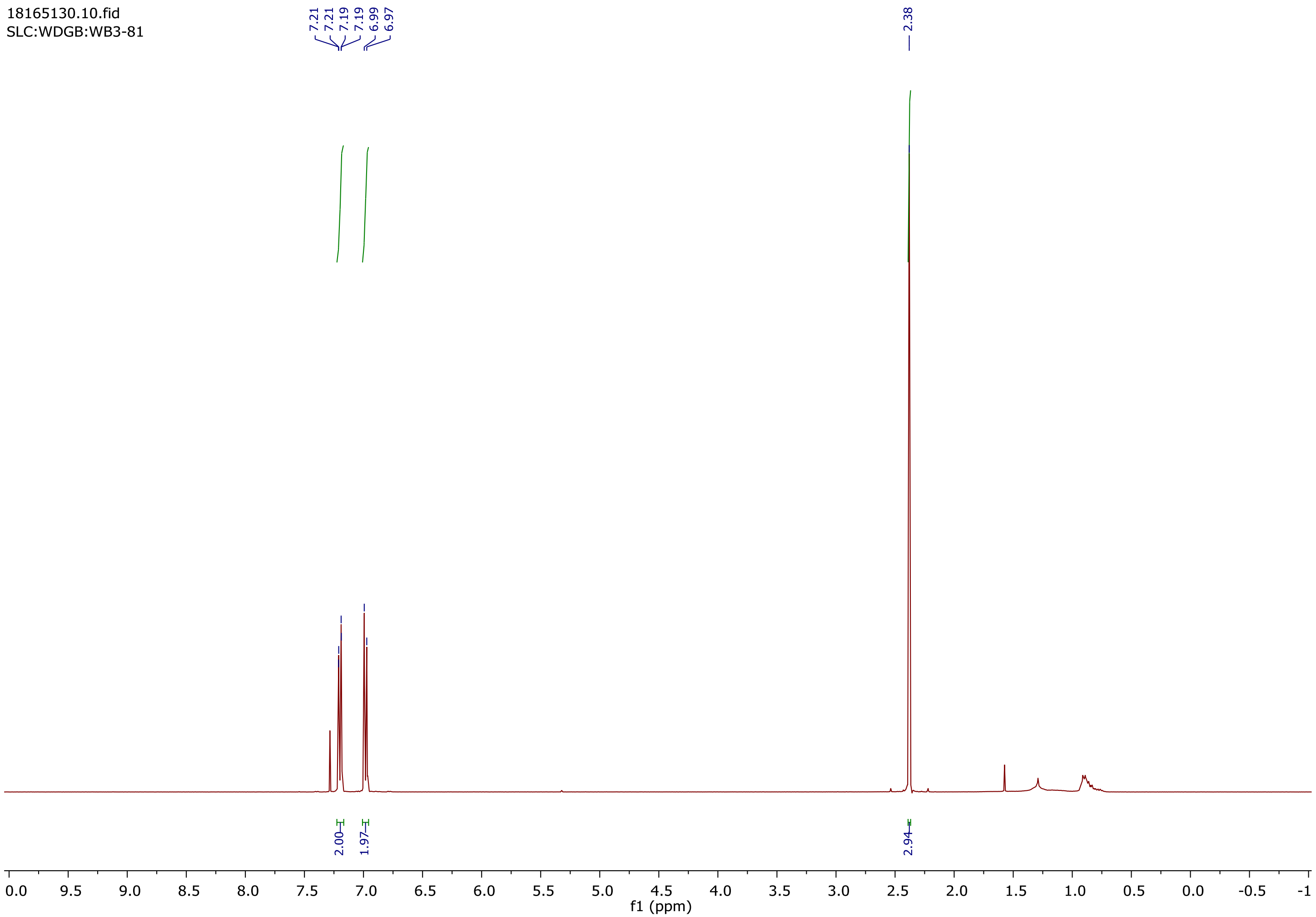

Figure S1. ^{1}H NMR spectrum of **1** recorded at 400 MHz in $CDCl_3$. Peaks at 7.26 ppm and 1.56 ppm correspond to $CHCl_3$ (in $CDCl_3$) and water respectively.

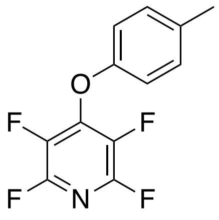

1

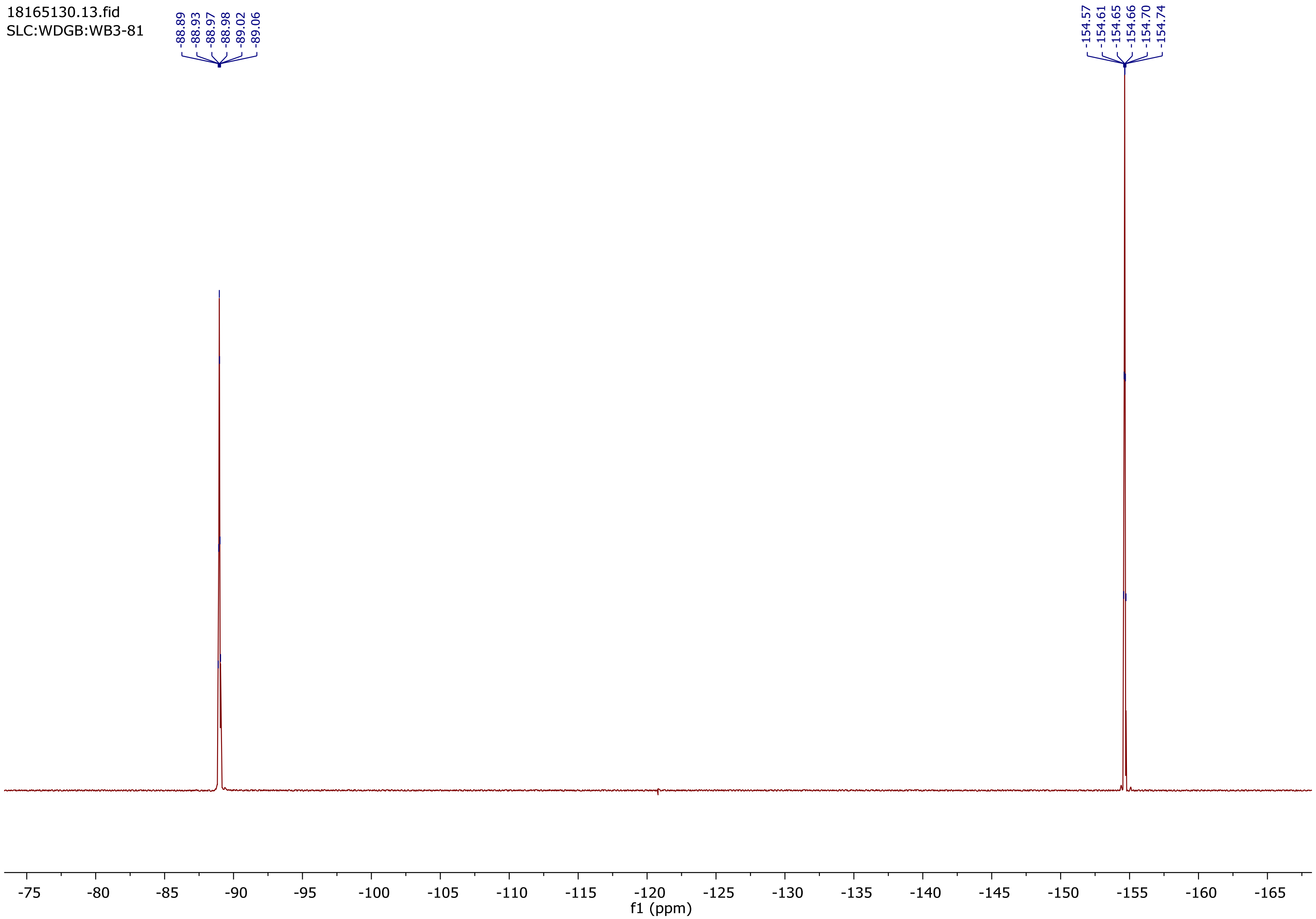

Figure S2. $^{19}F\{^{1}H\}$ NMR spectrum of **1** recorded at 376 MHz in $CDCl_3$.

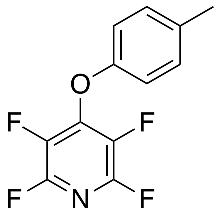

1

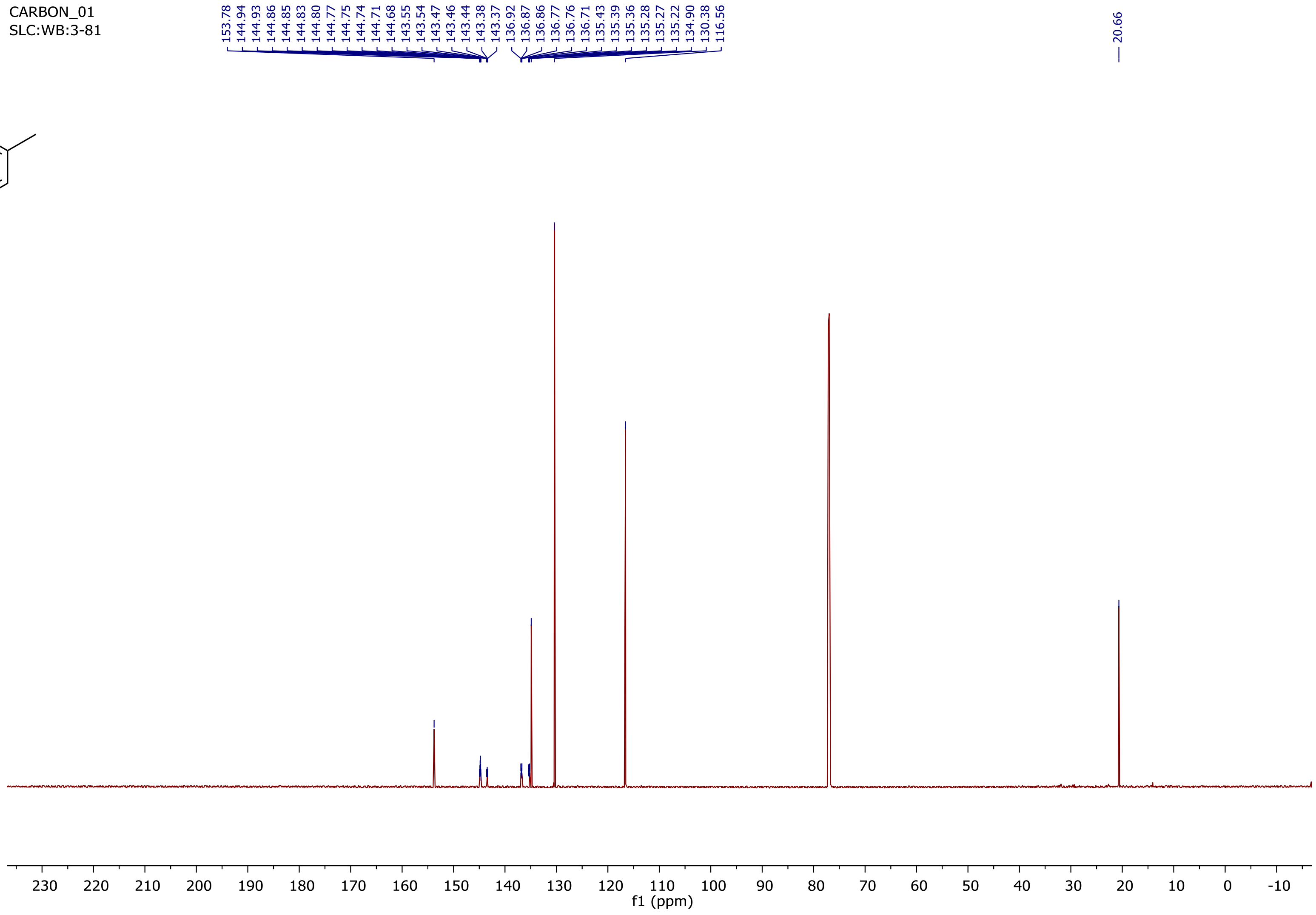

Figure S3. $^{13}C\{^{1}H\}$ NMR spectrum of **1** recorded at 176 MHz in $CDCl_3$.

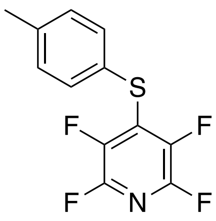

2

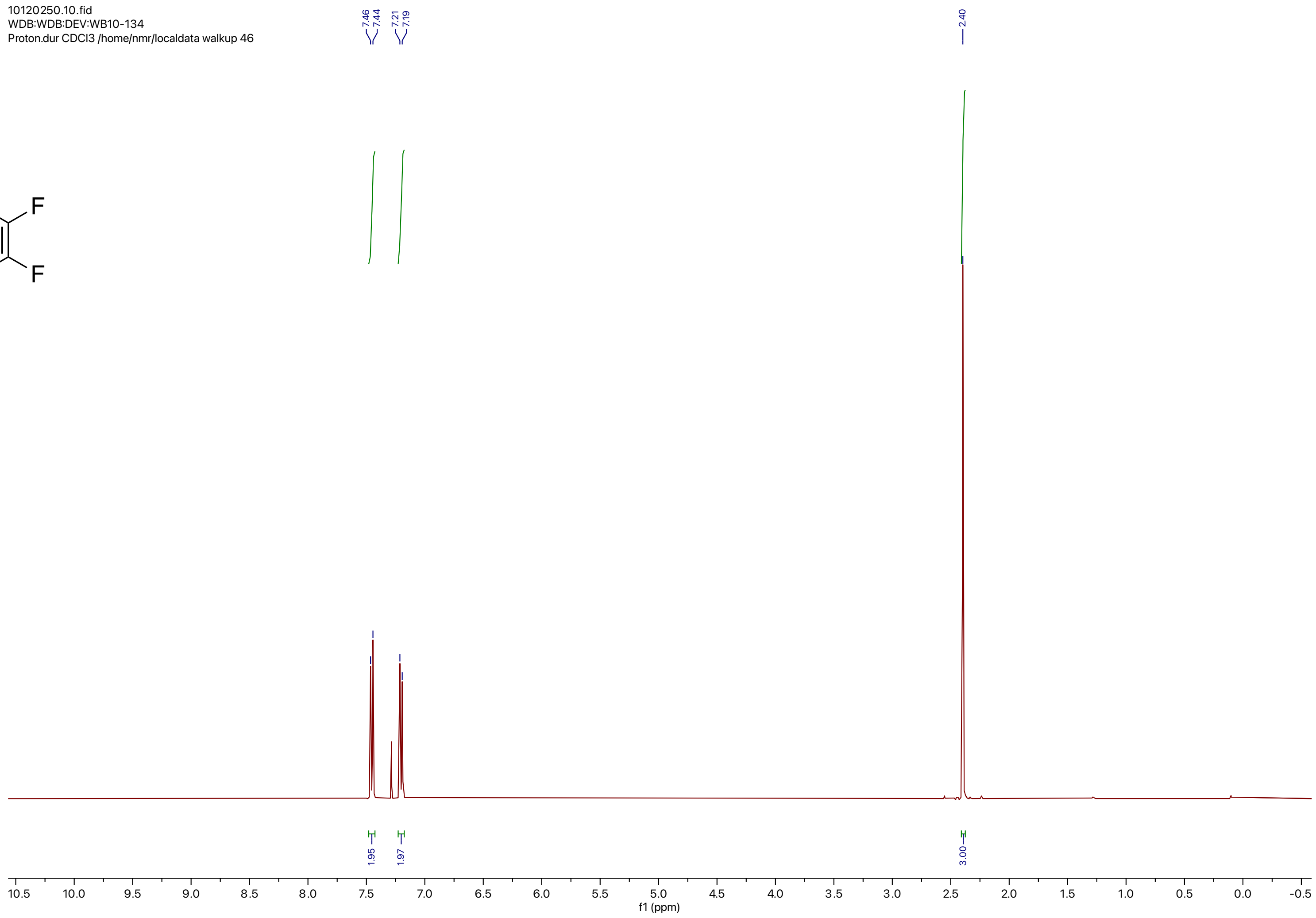

Figure S4. ^{1}H NMR spectrum of **2** recorded at 400 MHz in $CDCl_3$.

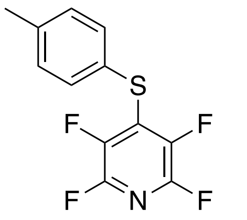

2

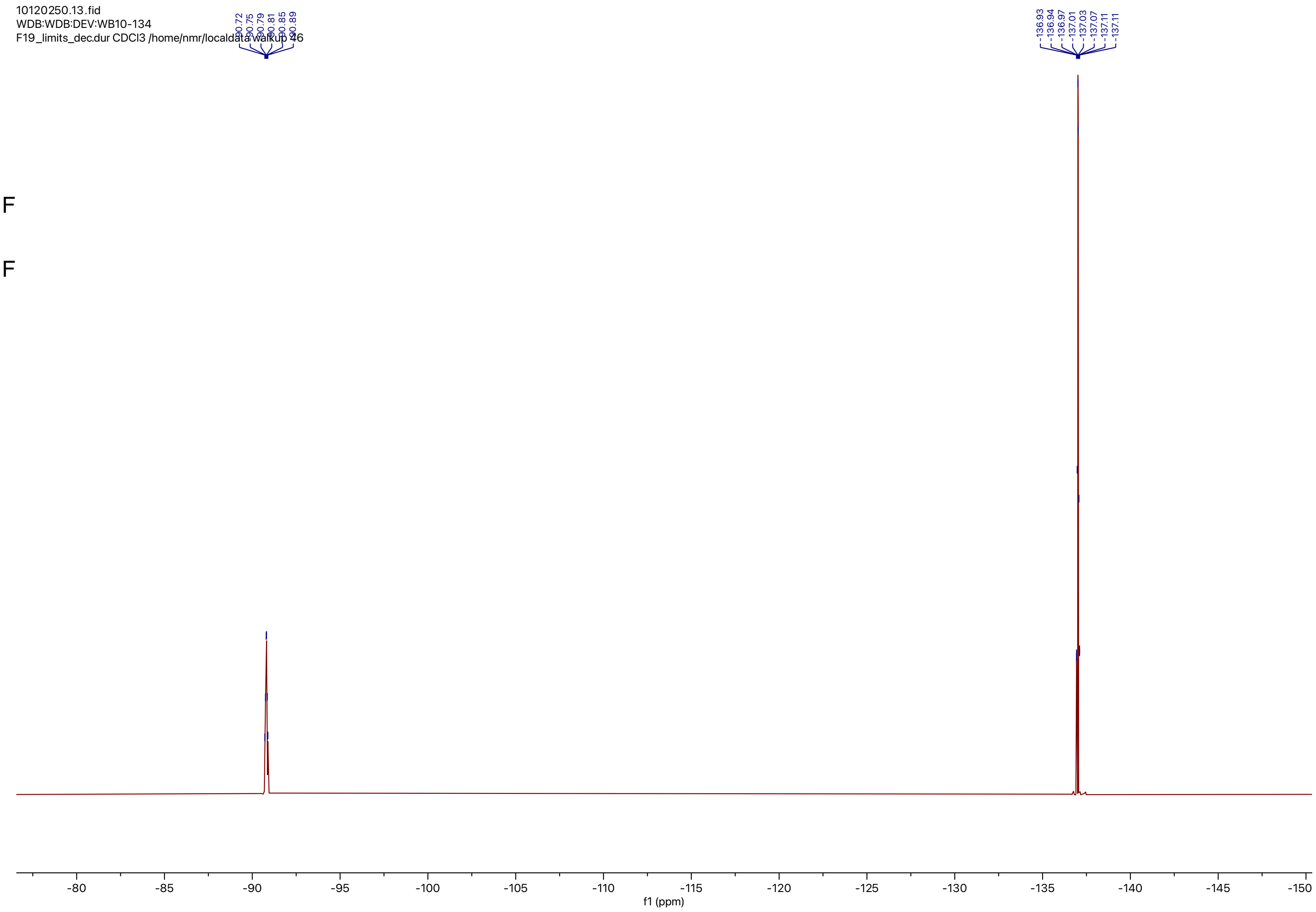

Figure S5. $^{19}F\{^{1}H\}$ NMR spectrum of **2** recorded at 376 MHz in $CDCl_3$.

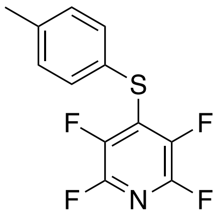

2

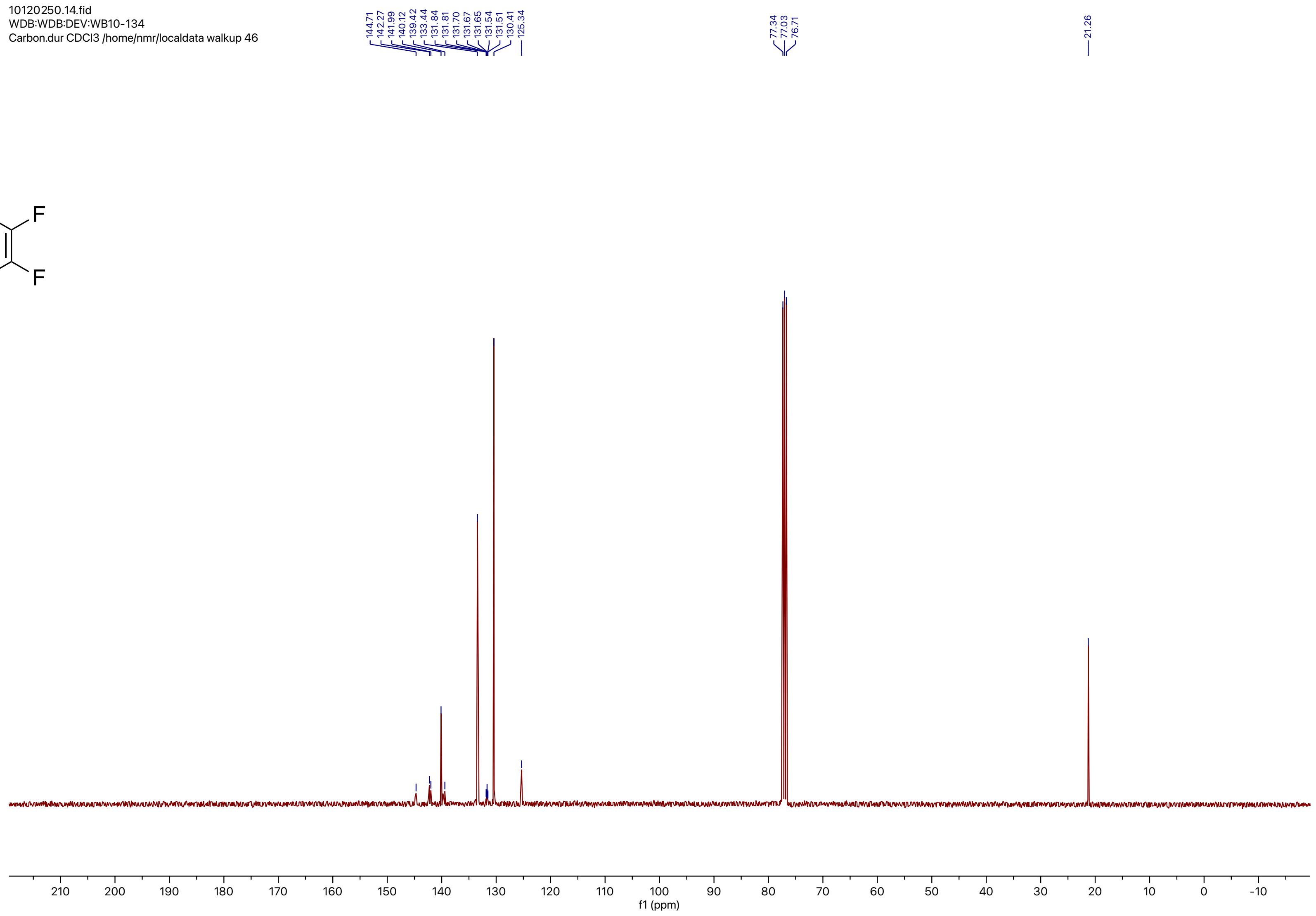

Figure S6. ${}^{13}C\{{}^{1}H\}$ NMR spectrum of **2** recorded at 101 MHz in $CDCl_3$.

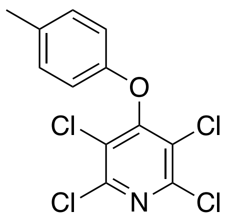

3

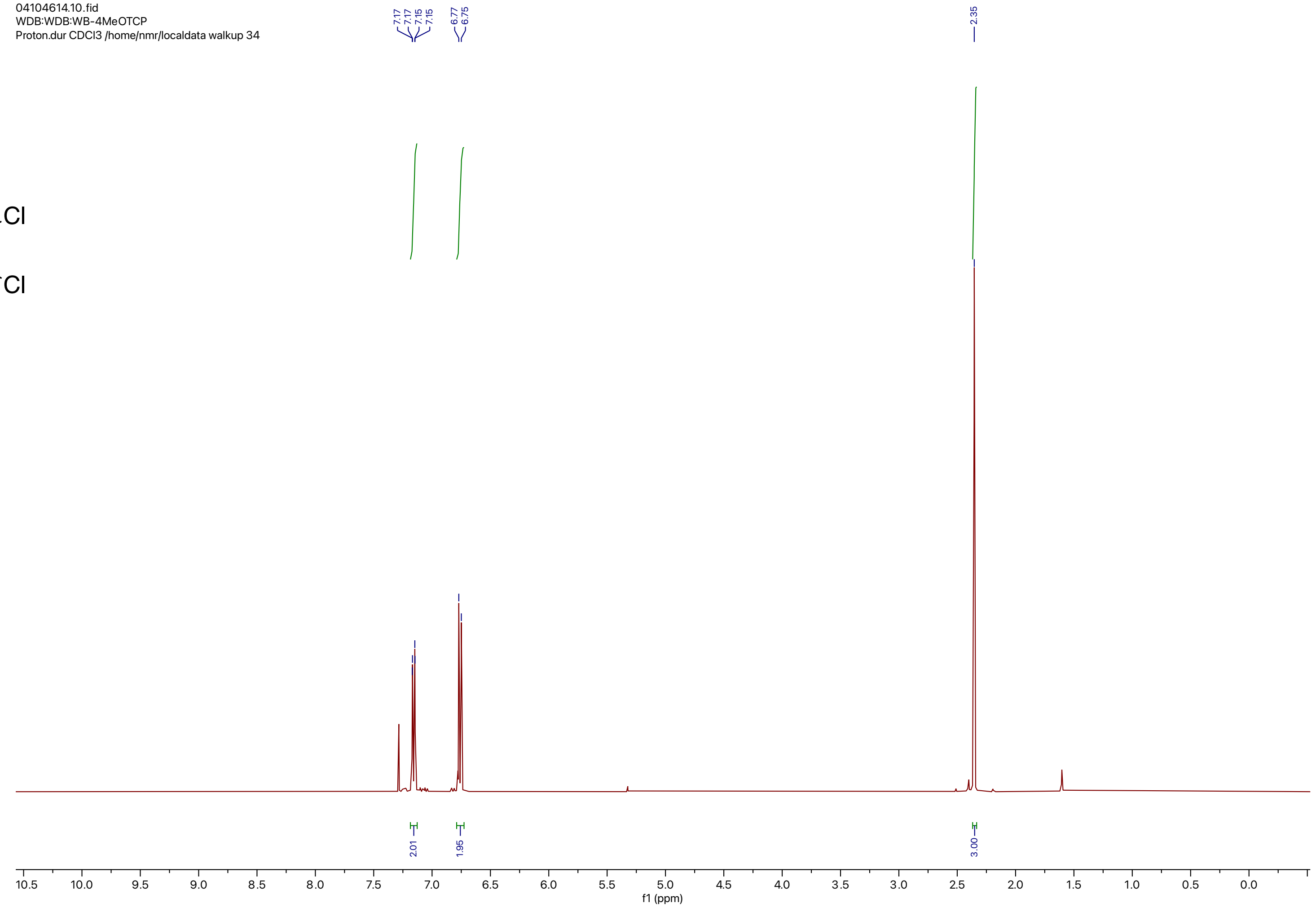

Figure S7. ^{1}H NMR spectrum of **3** in $CDCl_3$.

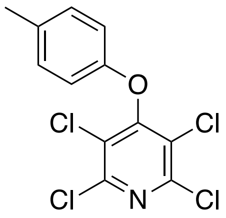

3

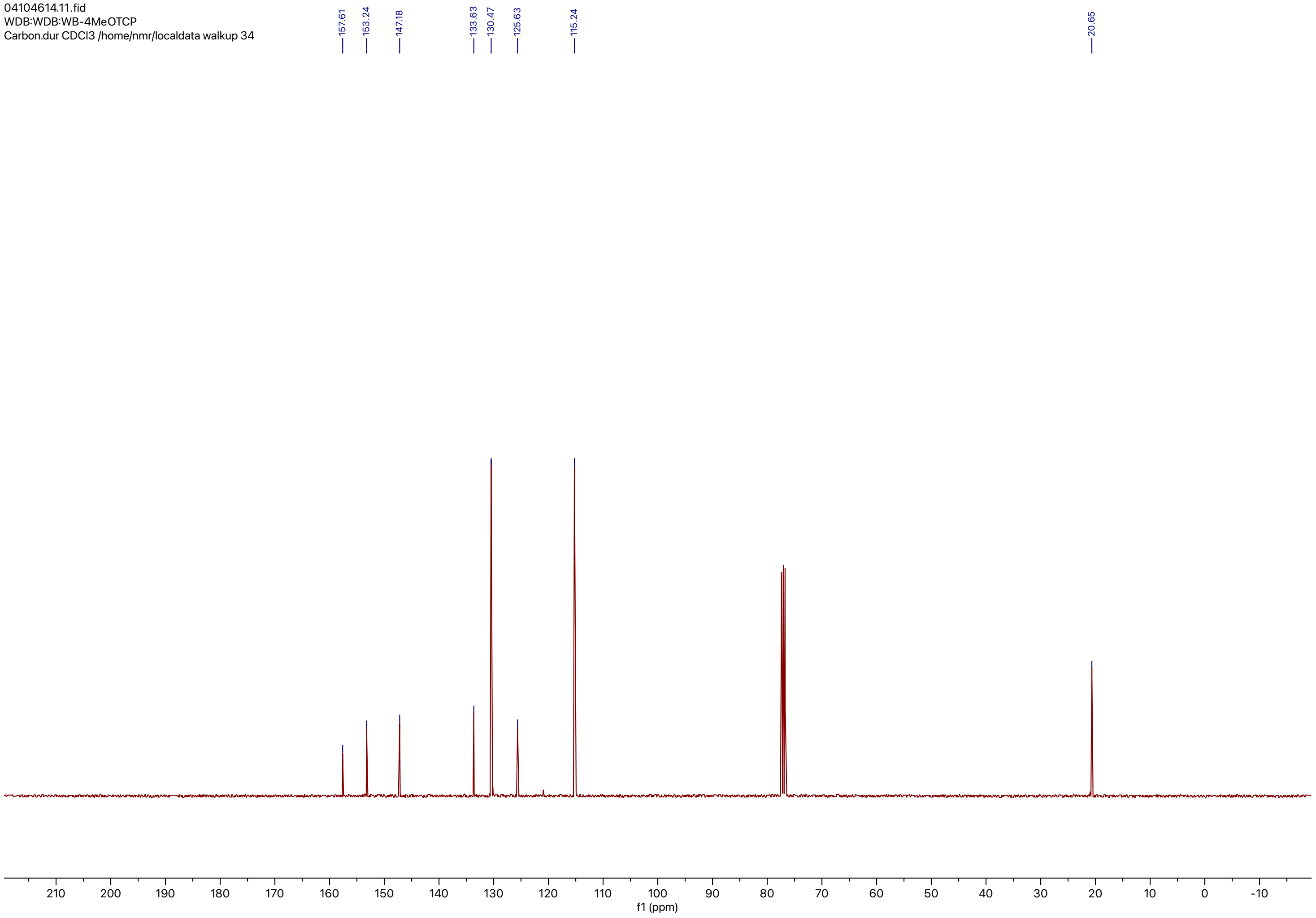

Figure S8. $^{13}C\{^{1}H\}$ NMR spectrum of **3** recorded at 101 MHz in $CDCl_3$.

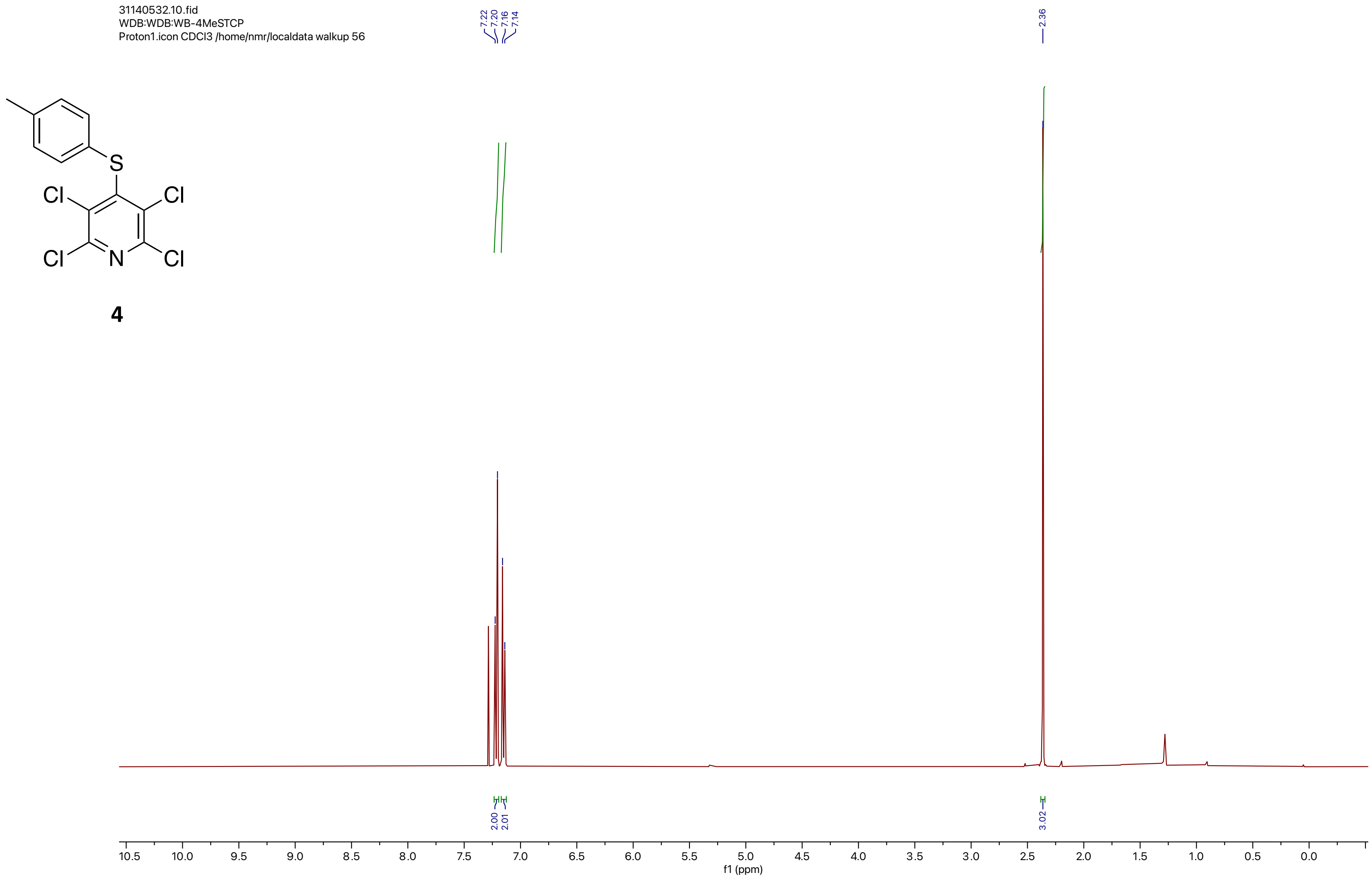

Figure S9. ^{1}H NMR spectrum of **4** recorded at 400 MHz in $CDCl_3$.

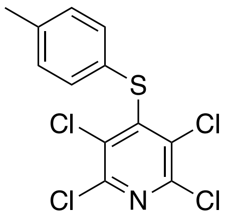

4

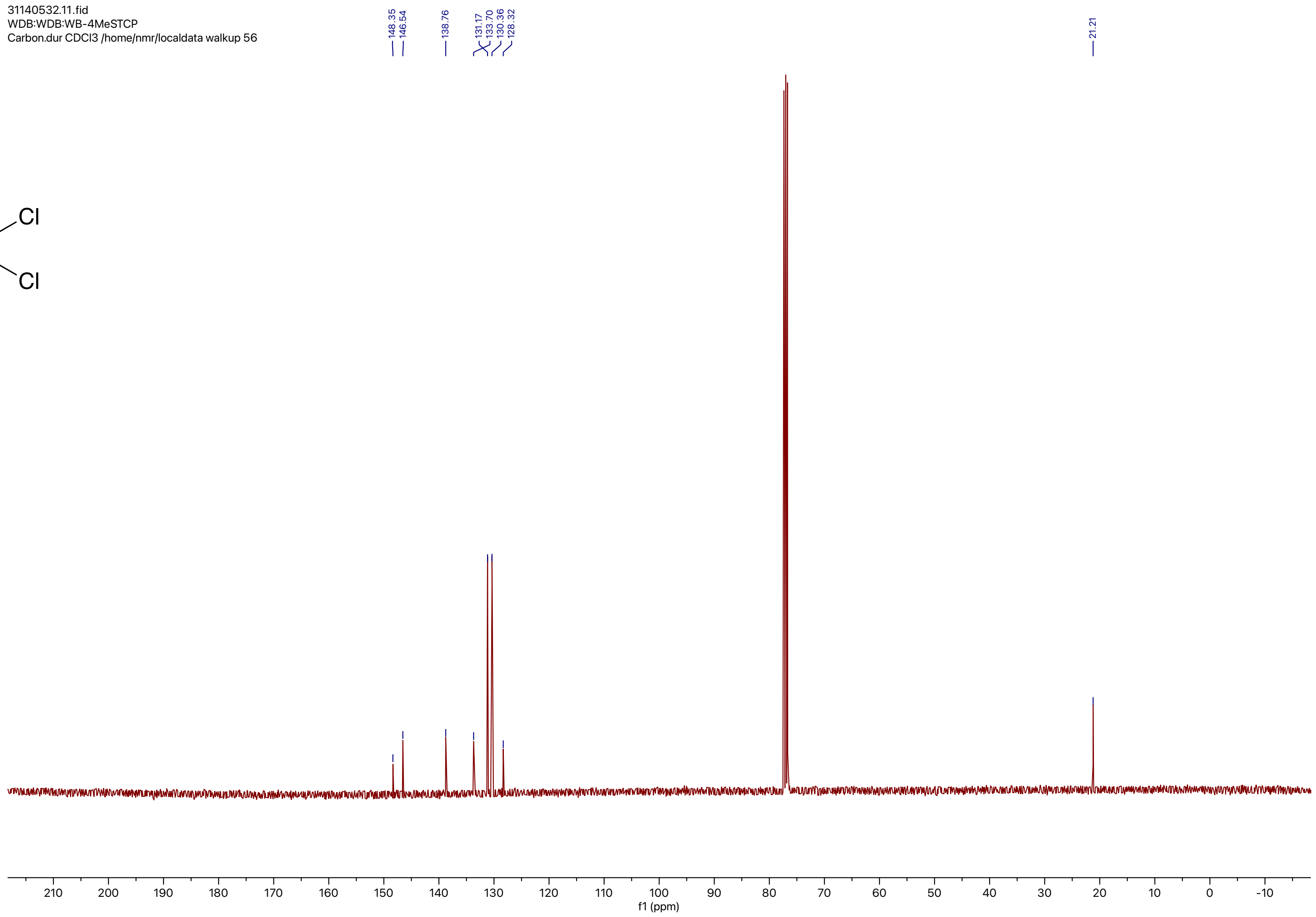

Figure S10. $^{13}C\{^{1}H\}$ NMR spectrum of **4** recorded at 101 MHz in $CDCl_3$.

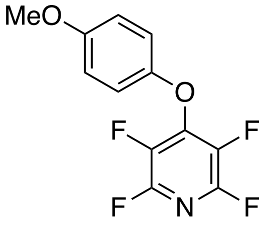

5

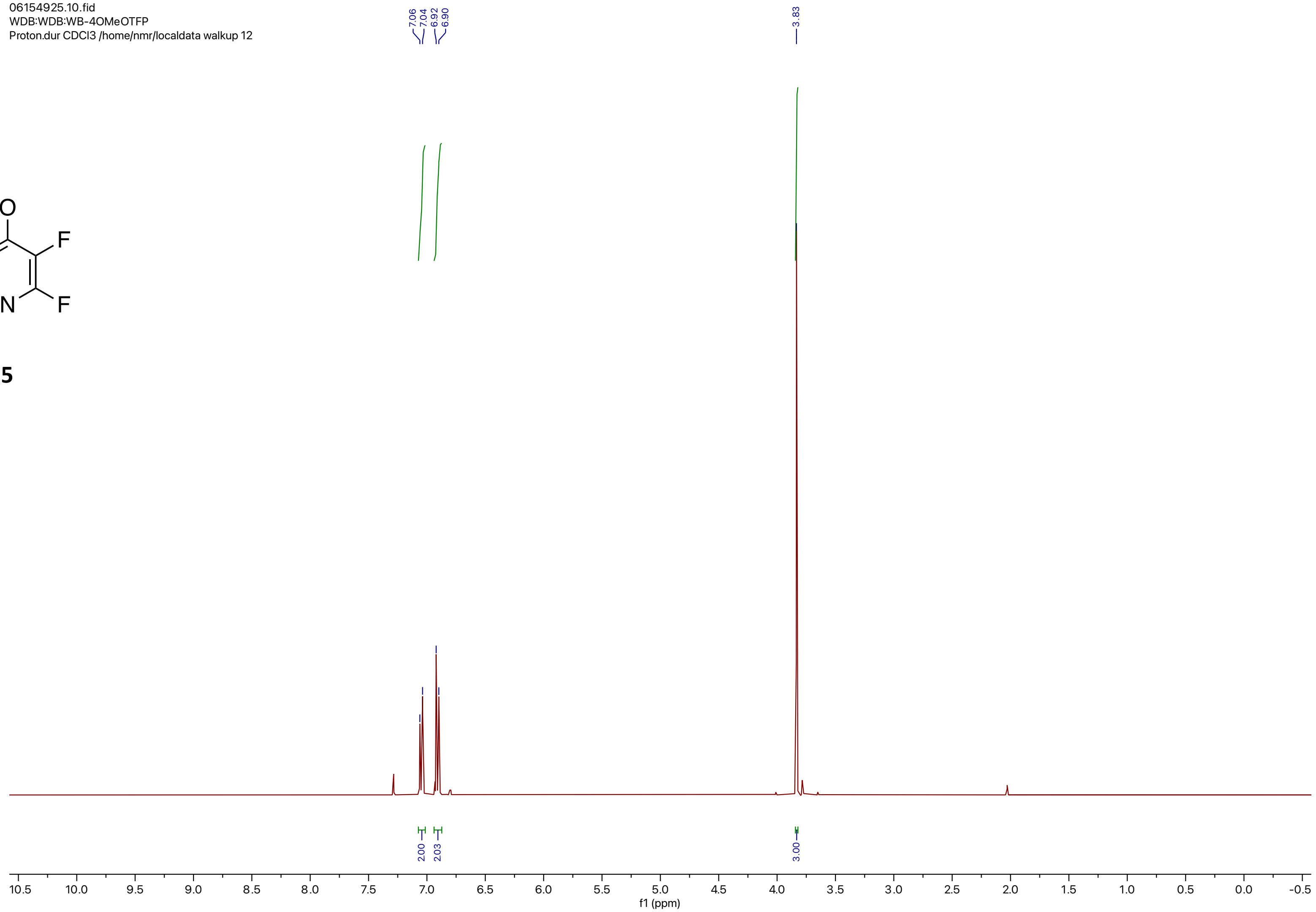

Figure S11. ^{1}H NMR spectrum of **5** recorded at 400 MHz in $CDCl_3$.

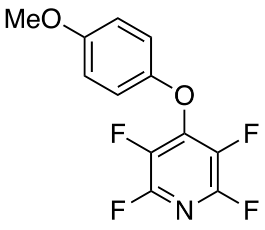

5

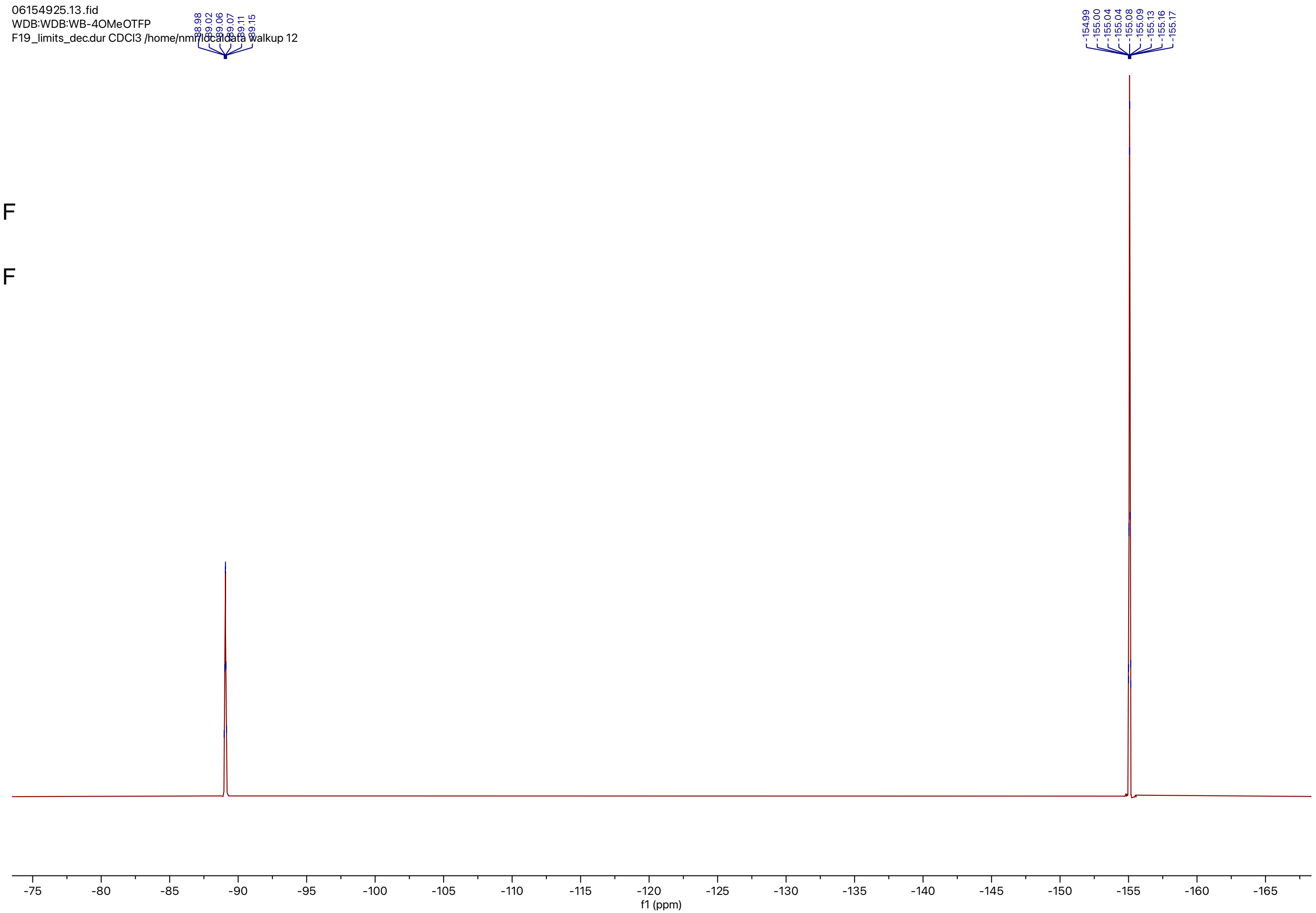

Figure S12. $^{19}F\{^{1}H\}$ NMR spectrum of **5** recorded at 376 MHz in $CDCl_3$.

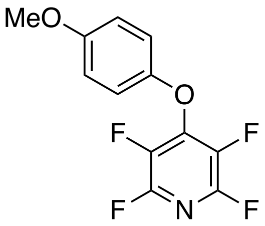

5

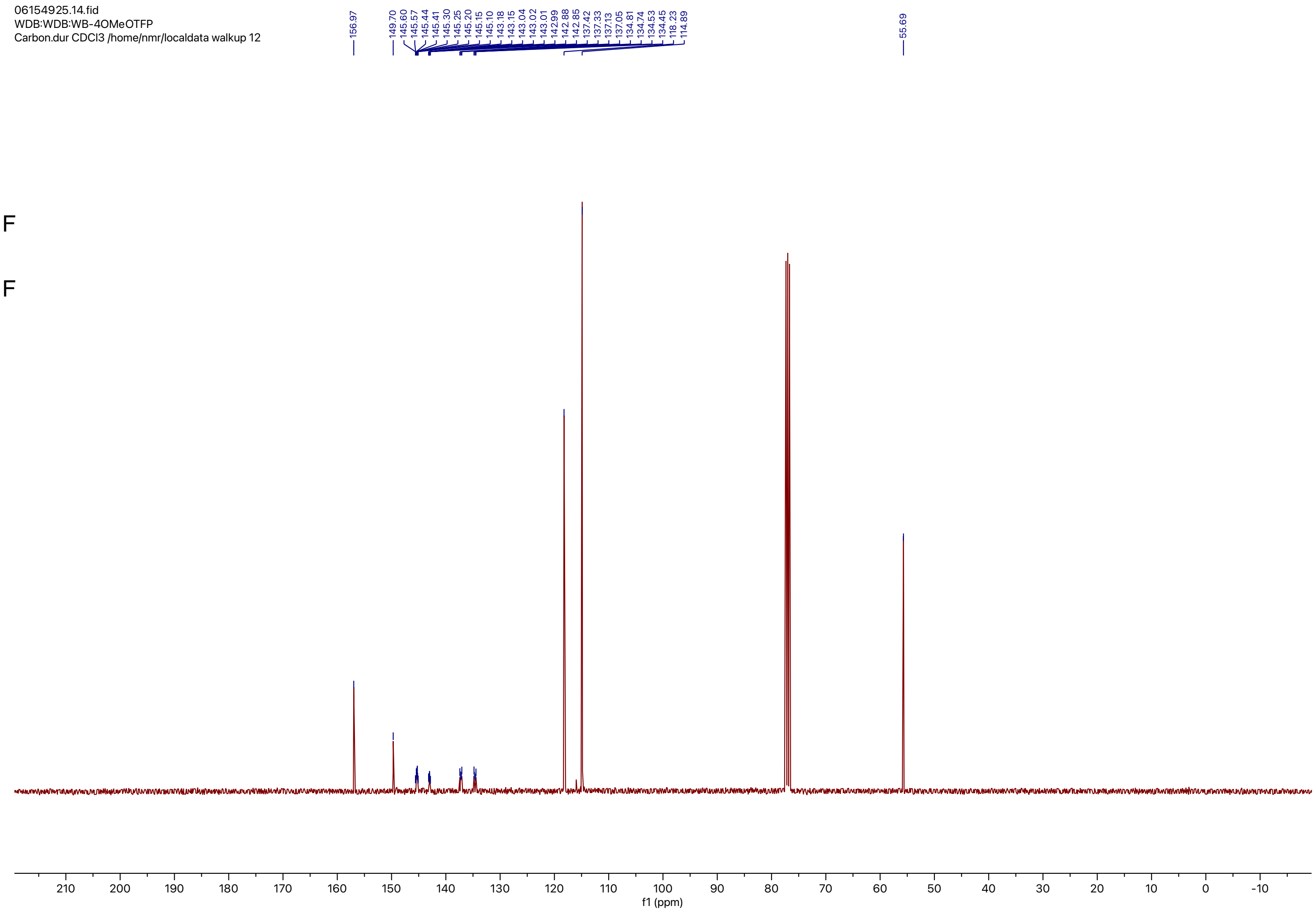

Figure S13. $^{13}C\{^{1}H\}$ NMR spectrum of **5** recorded at 101 MHz in $CDCl_3$.

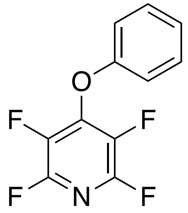

6

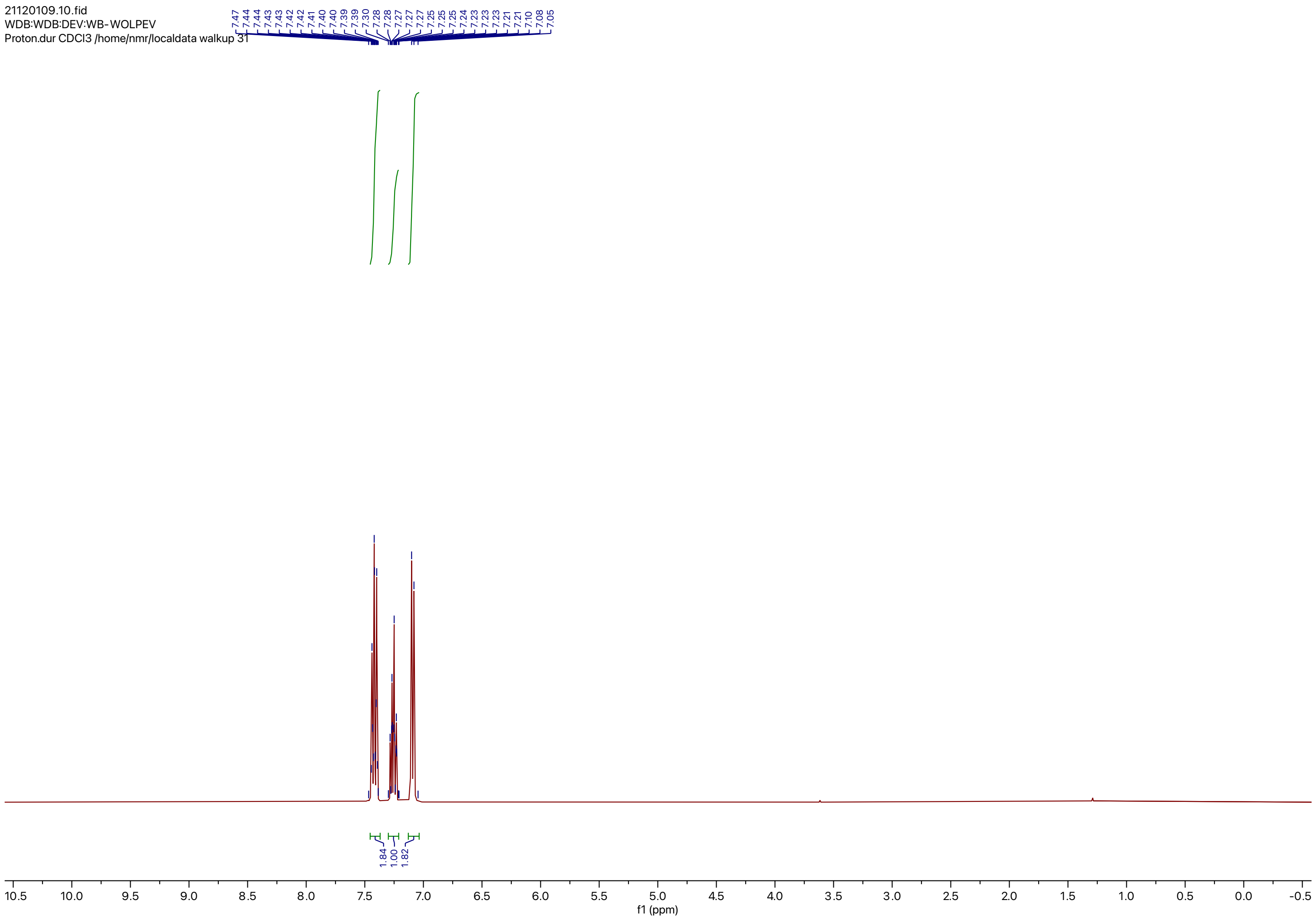

Figure S14. ^{1}H NMR spectrum of **6** recorded at 400nMHz in $CDCl_3$.

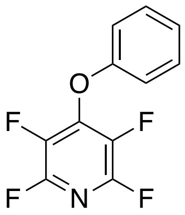

6

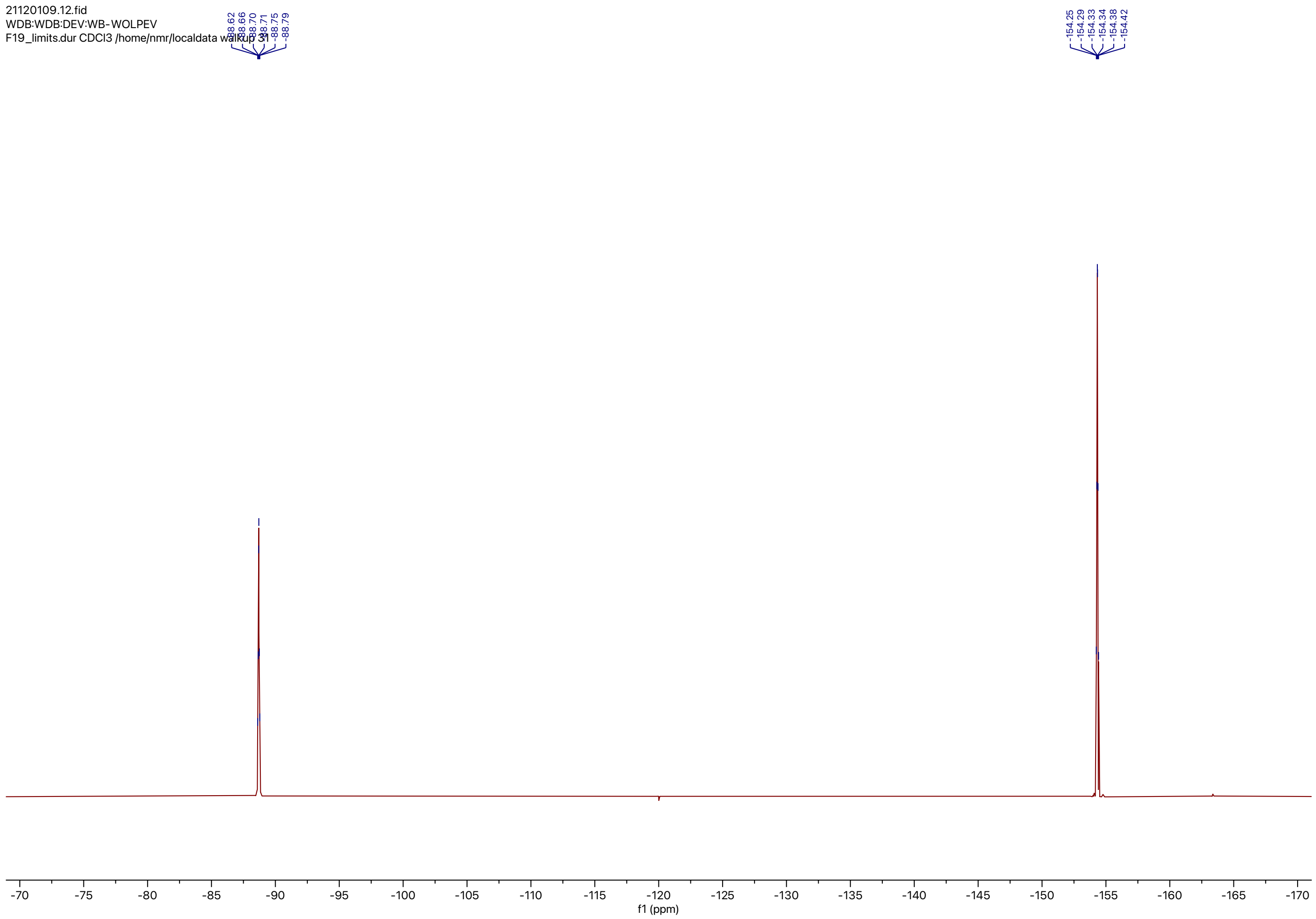

Figure S15. $^{19}F\{^{1}H\}$ NMR spectrum of **5** recorded at 376 MHz in $CDCl_3$.

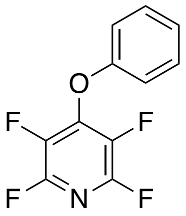

6

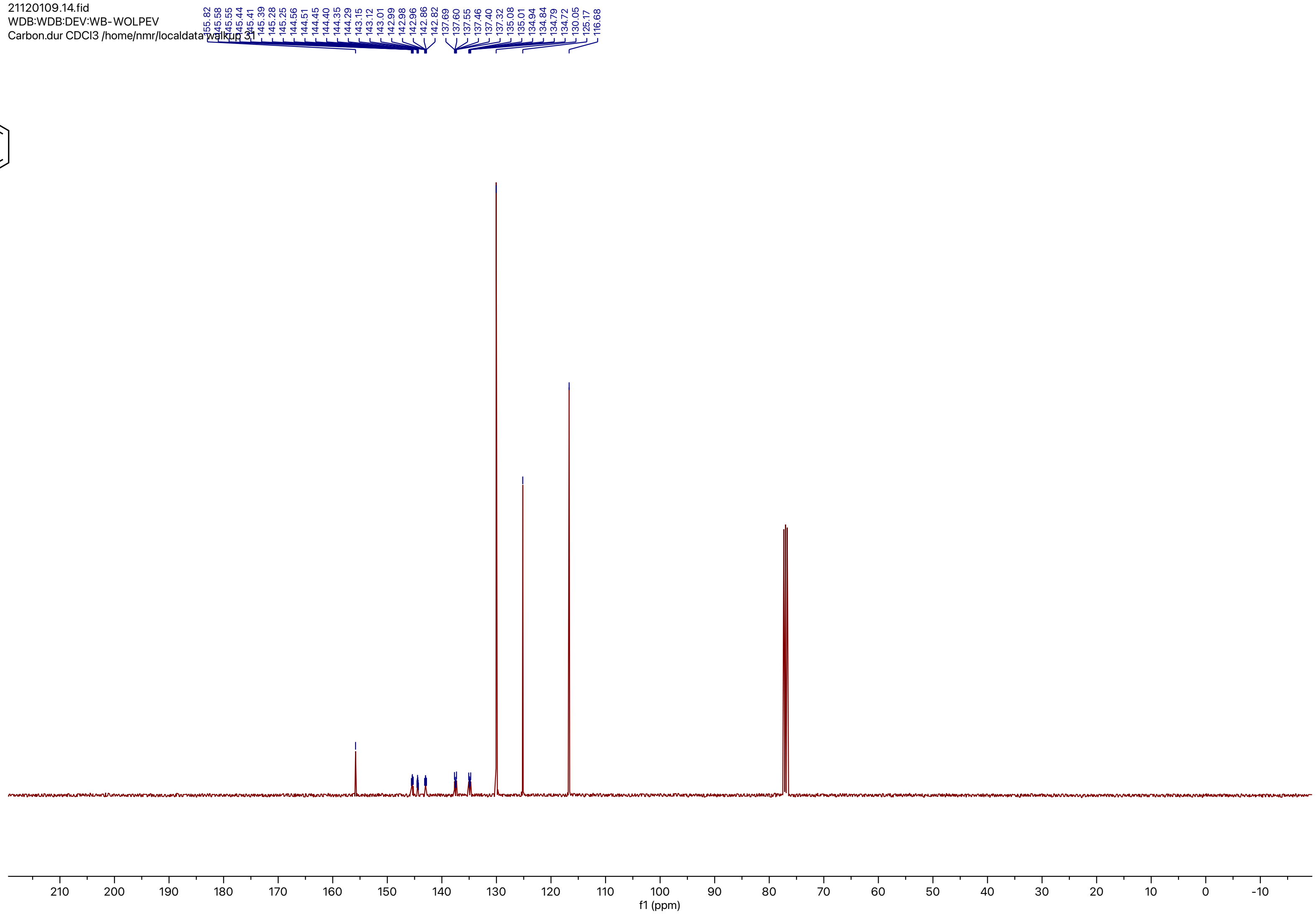

Figure S16. $^{13}C\{^{1}H\}$ NMR spectrum of **6** recorded at 101 MHz in $CDCl_3$.

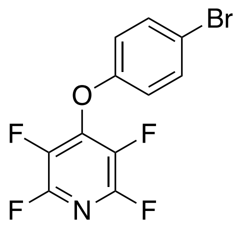

7

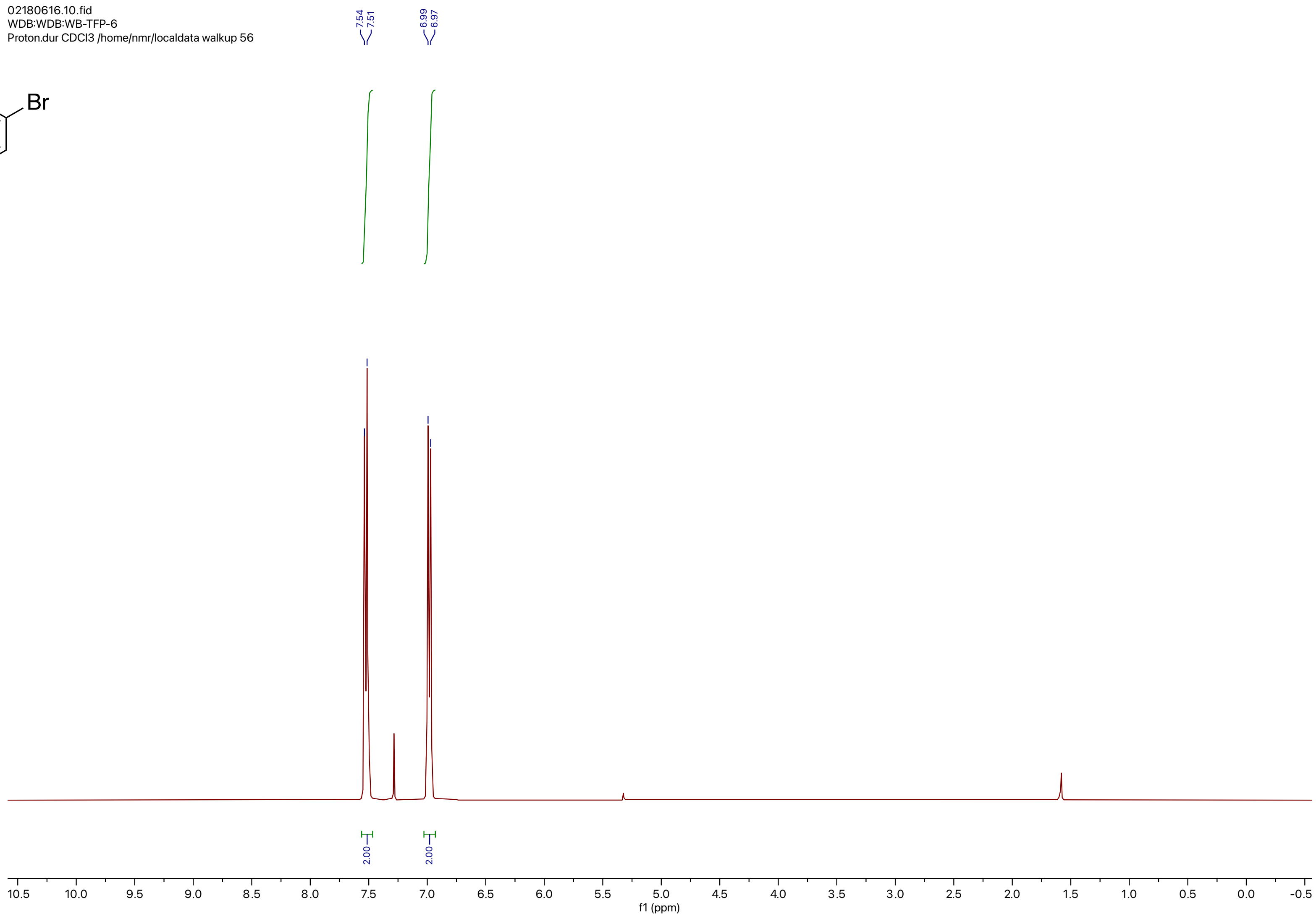

Figure S17. ^{1}H NMR spectrum of **7** recorded at 400 MHz in $CDCl_3$.

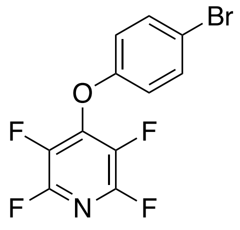

7

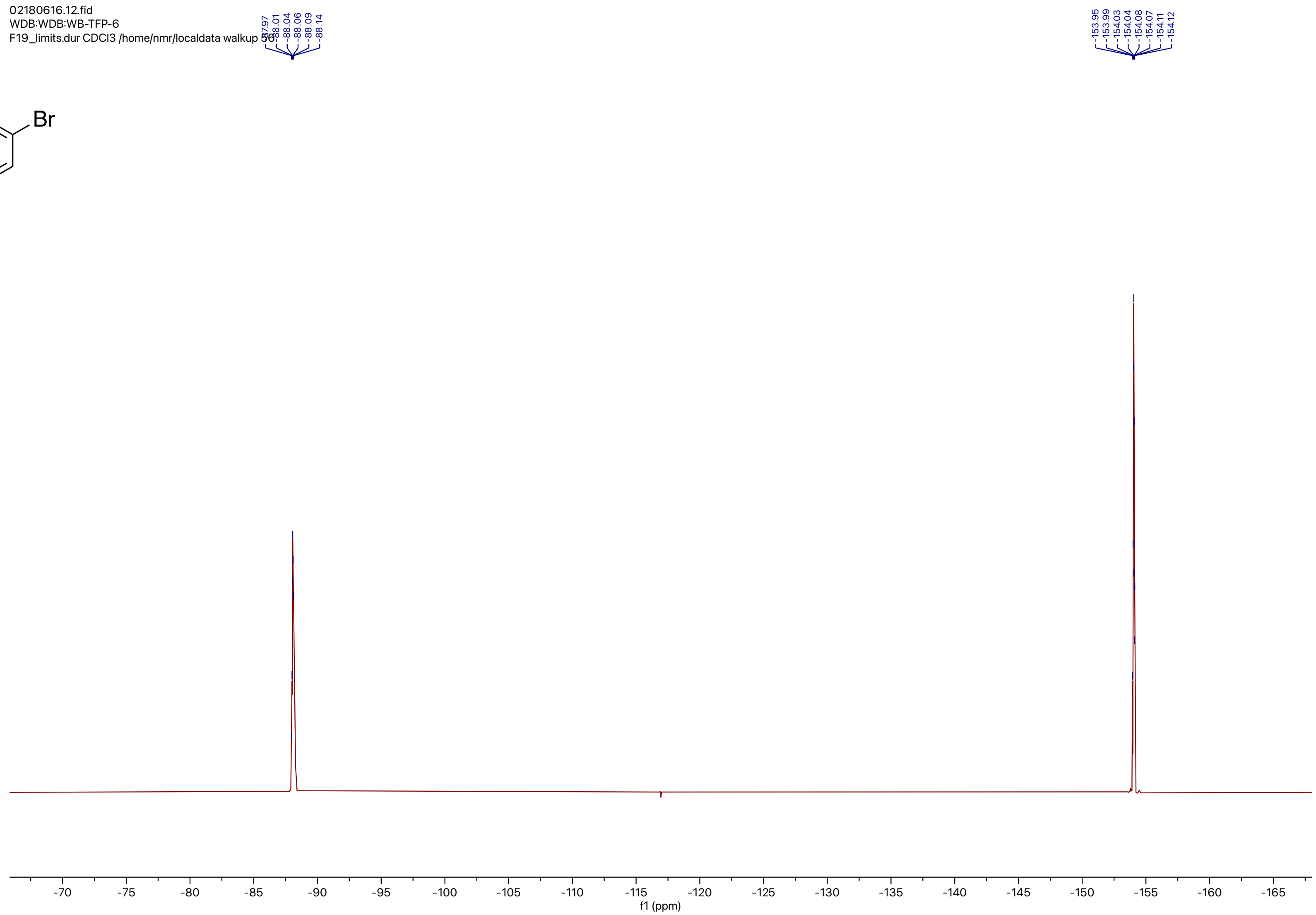

Figure S18. $^{19}F\{^{1}H\}$ NMR spectrum of **7** recorded at 376 MHz in $CDCl_3$.

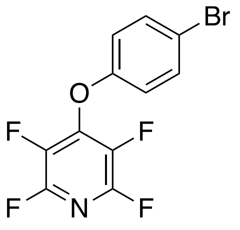

7

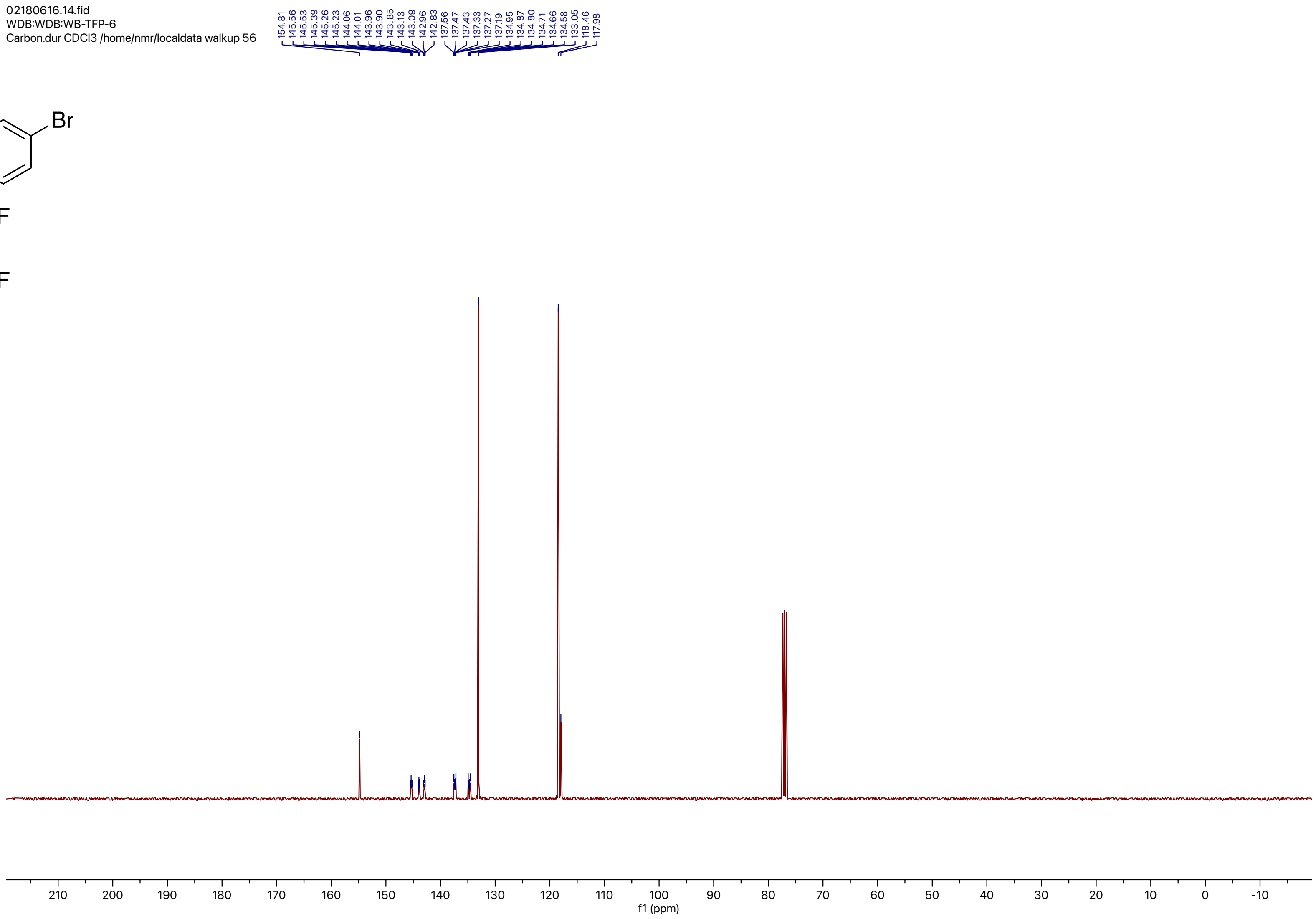

Figure S19. $^{13}C\{^{1}H\}$ NMR spectrum of **7** recorded at 101 MHz in $CDCl_3$.

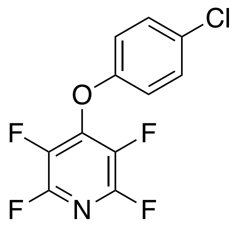

8

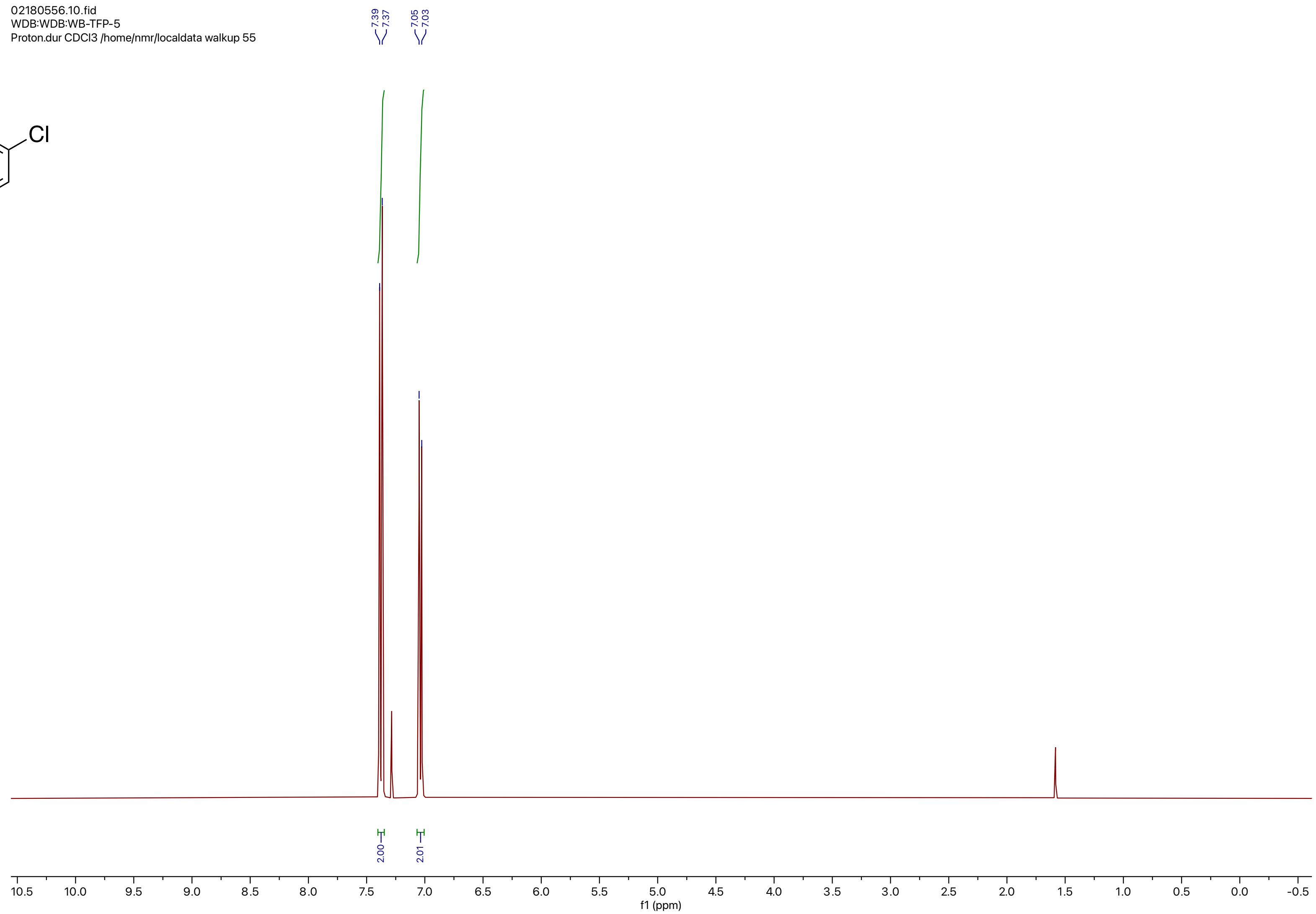

Figure S20. ^{1}H NMR spectrum of **8** recorded at 400 MHz in $CDCl_3$.

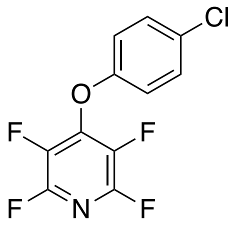

8

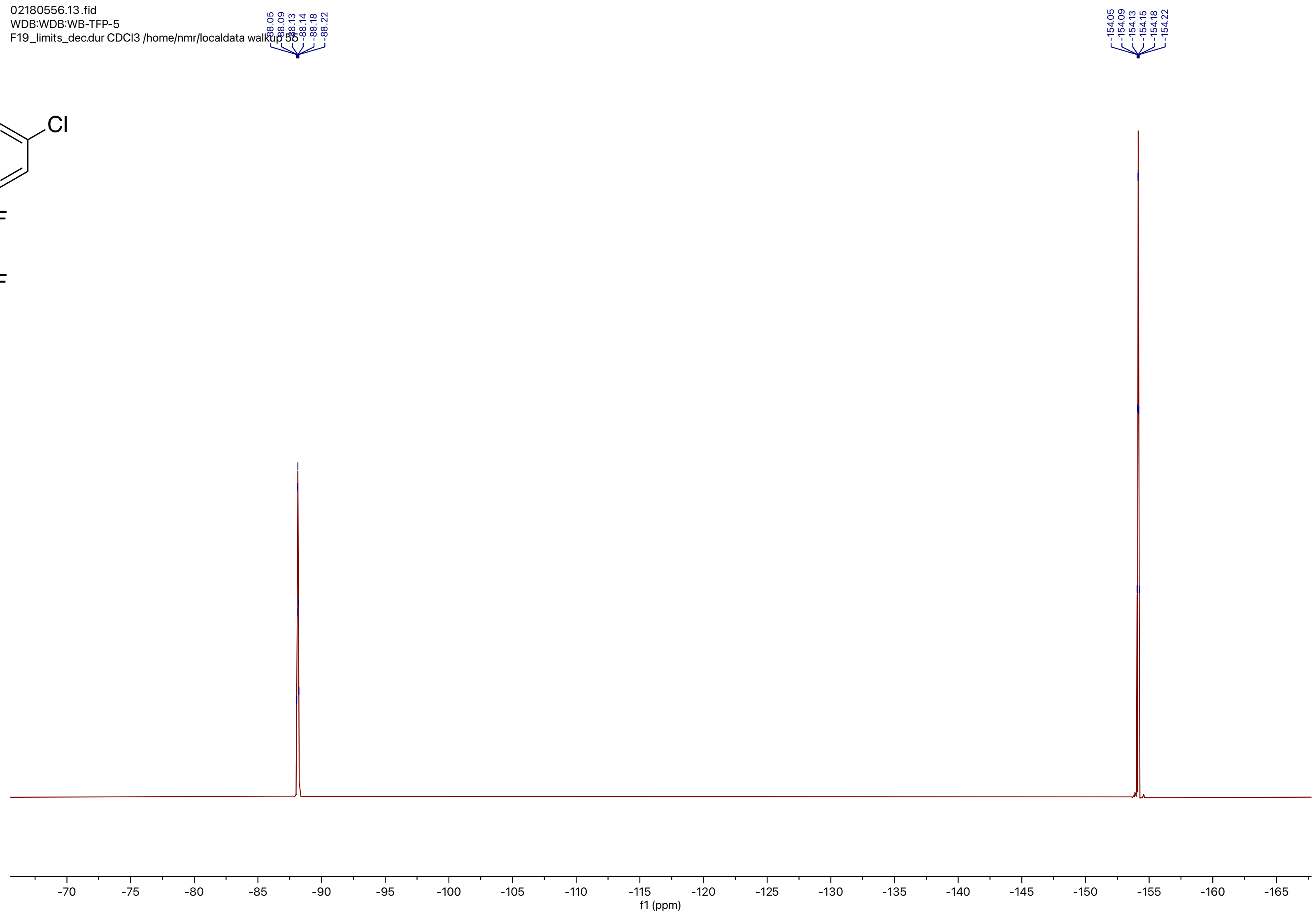

Figure S21. $^{19}F\{^1H\}$ NMR spectrum of **8** recorded at 376 MHz in $CDCl_3$.

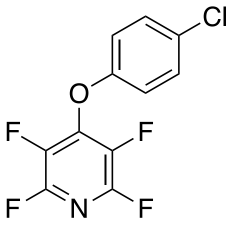

8

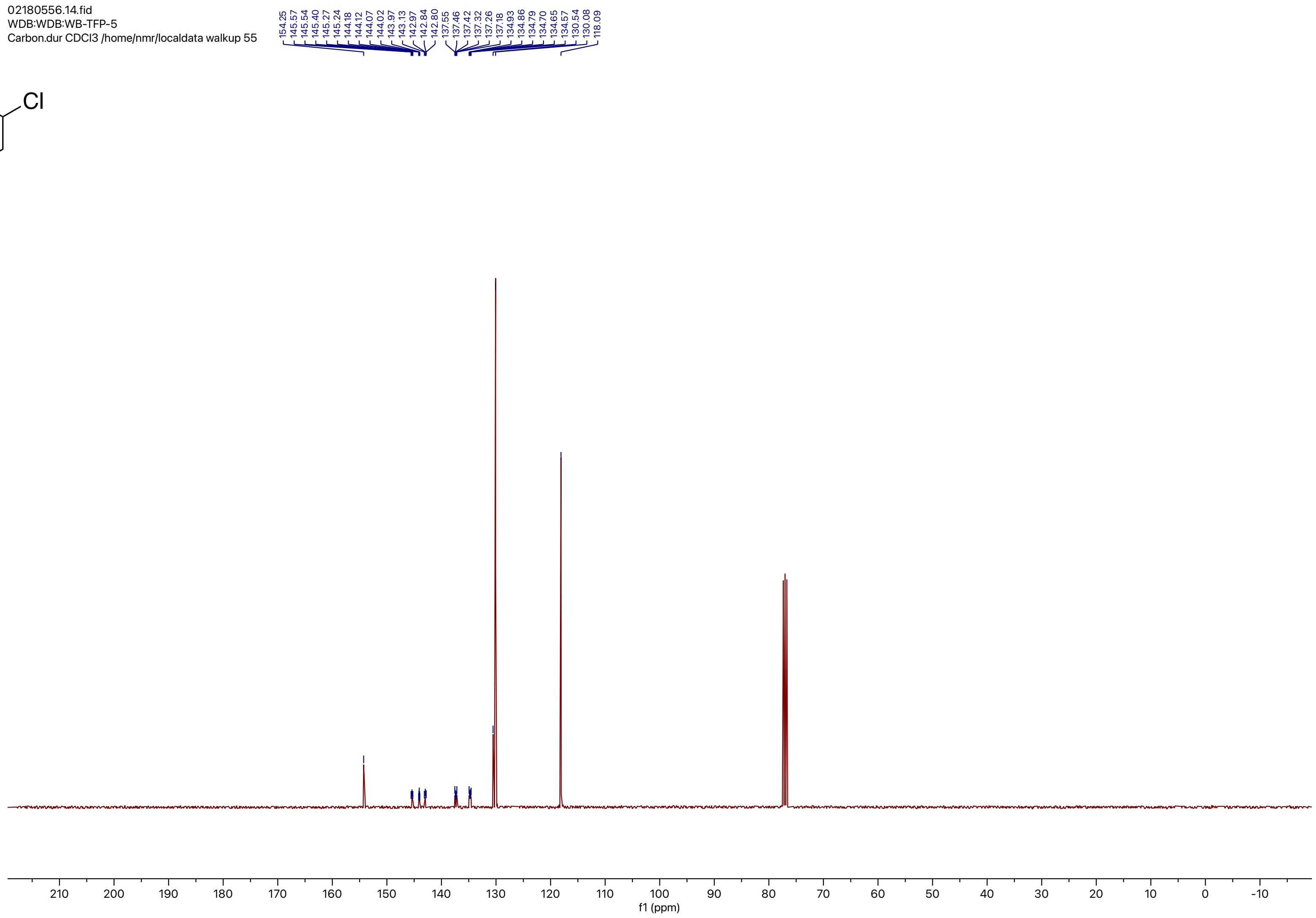

Figure S22. $^{13}C\{^{1}H\}$ NMR spectrum of **8** recorded at 101 MHz in $CDCl_3$.

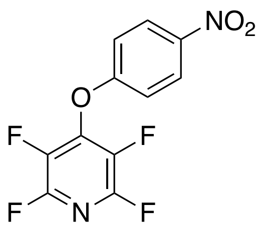

9

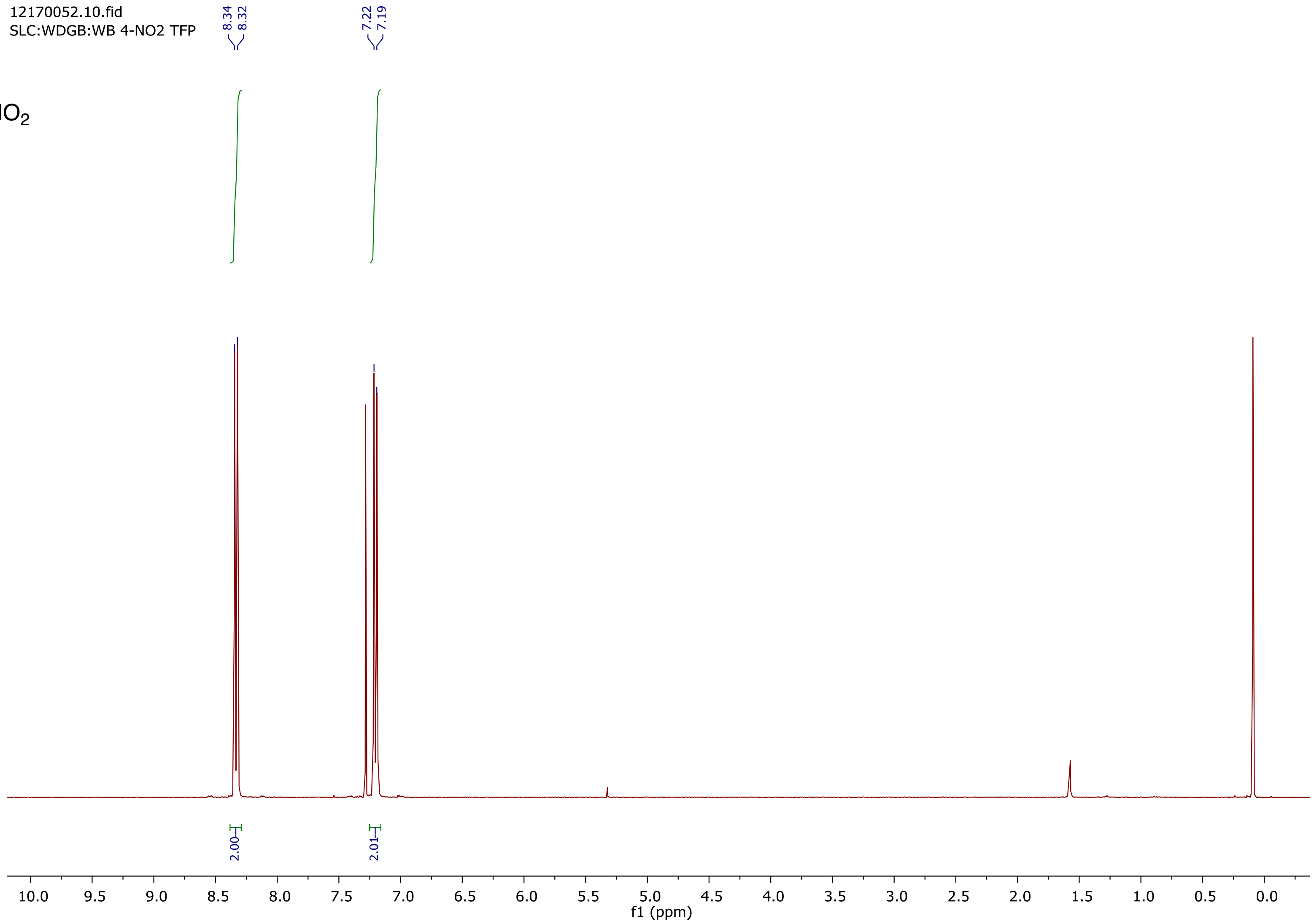

Figure S23. ^{1}H NMR spectrum of **9** recorded at 400 MHz in $CDCl_3$.

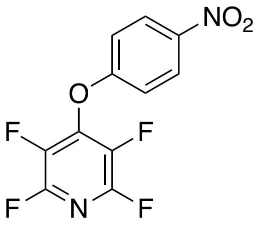

9

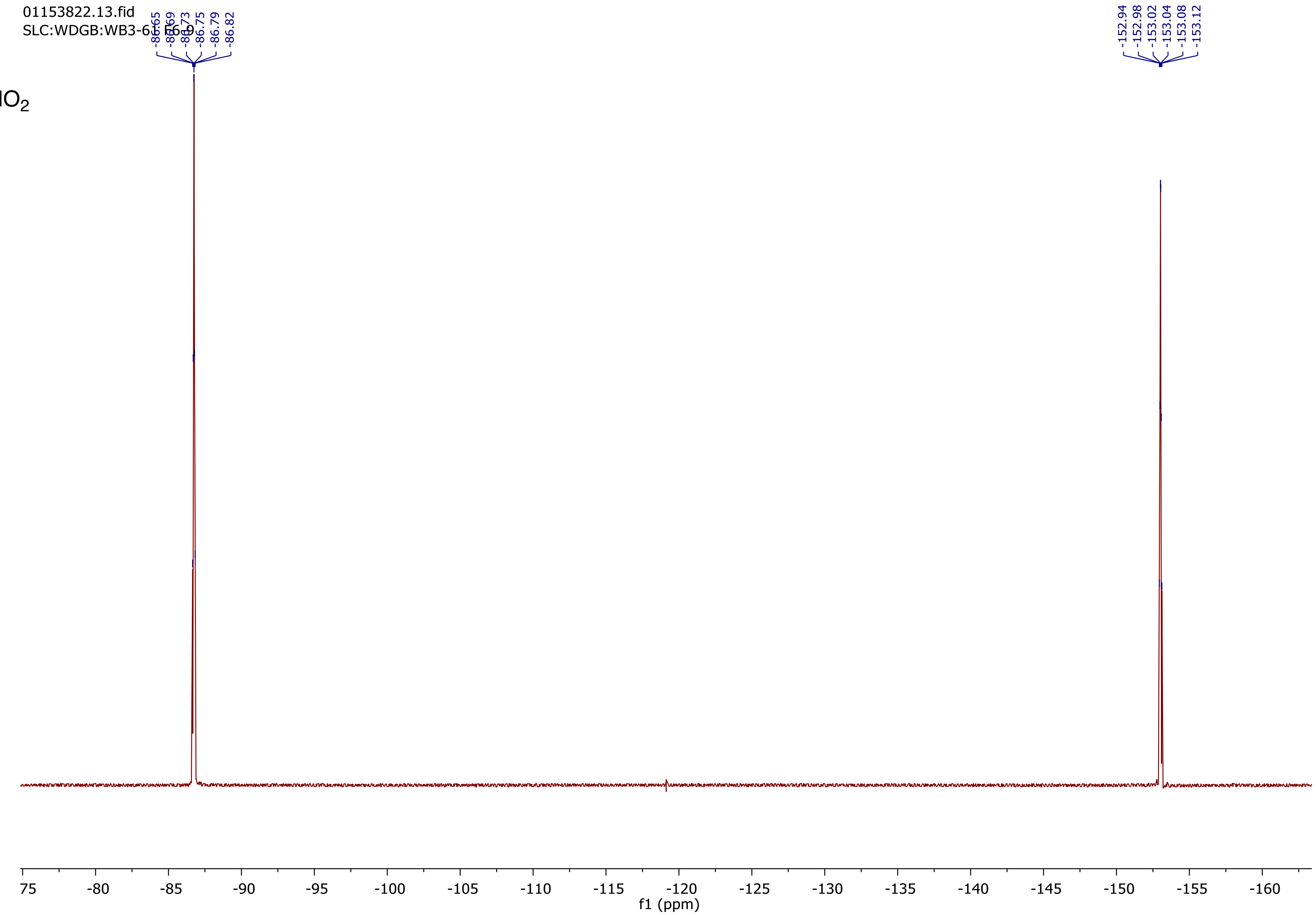

Figure S24. $^{19}F\{^1H\}$ NMR spectrum of **9** recorded at 376 MHz in $CDCl_3$.

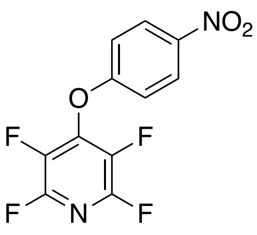

9

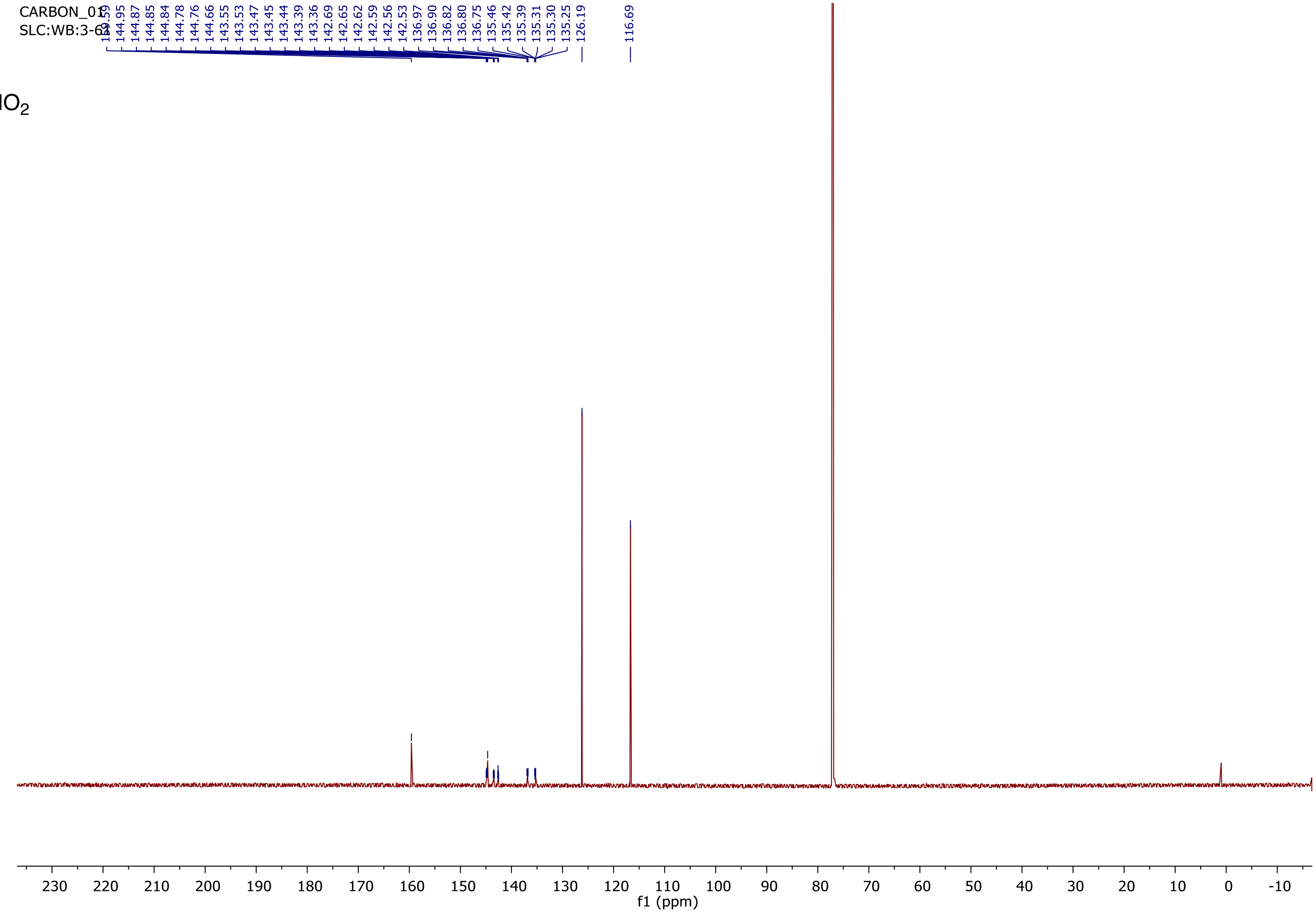

Figure S25. $^{13}C\{^1H\}$ NMR spectrum of **9** recorded at 101 MHz in $CDCl_3$.

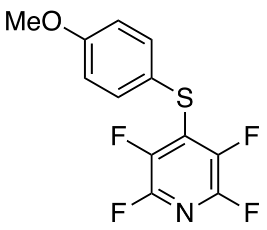

10

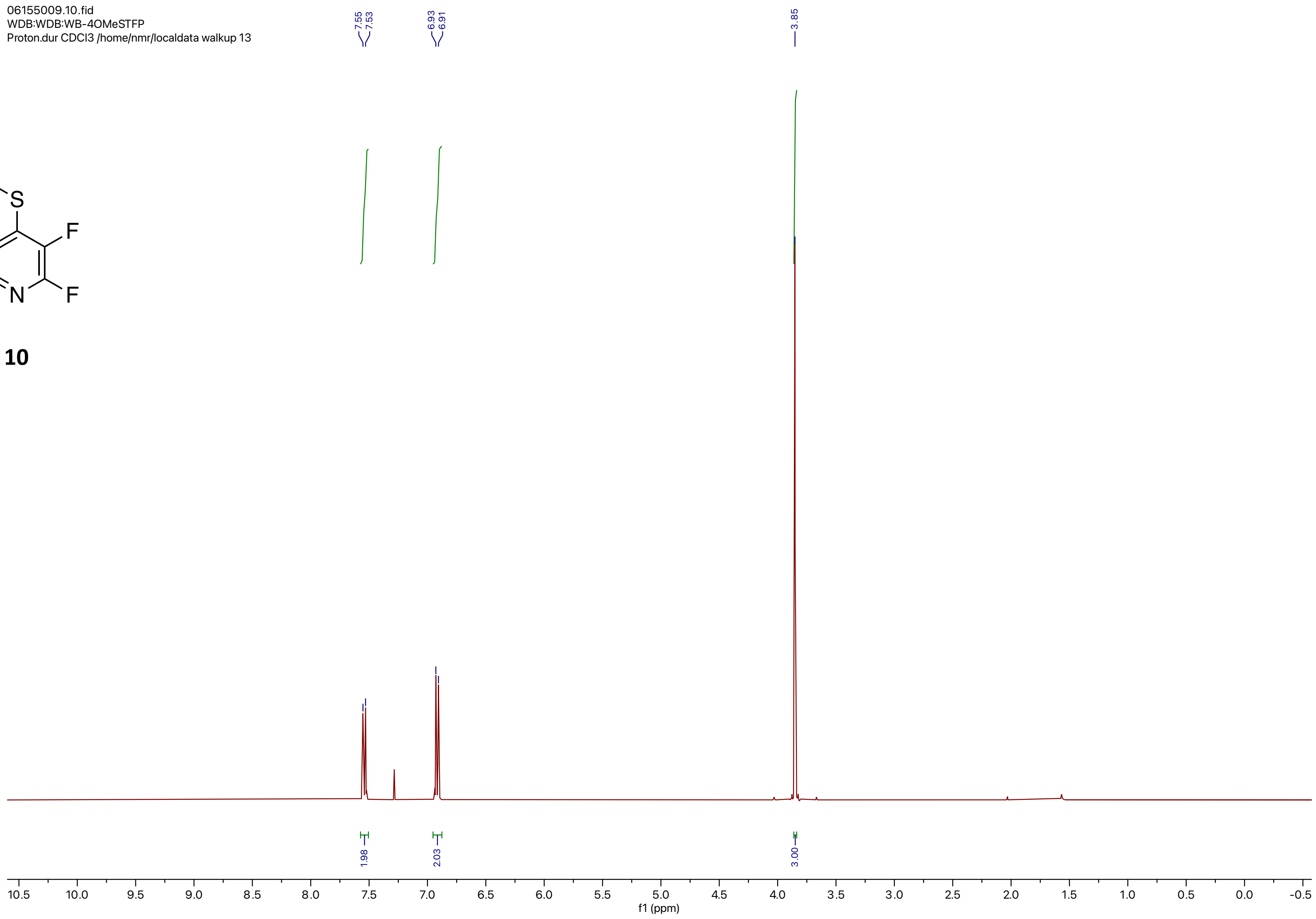

Figure S26. ^{1}H NMR spectrum of **10** recorded at 400 MHz in $CDCl_3$.

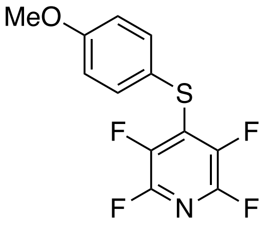

10

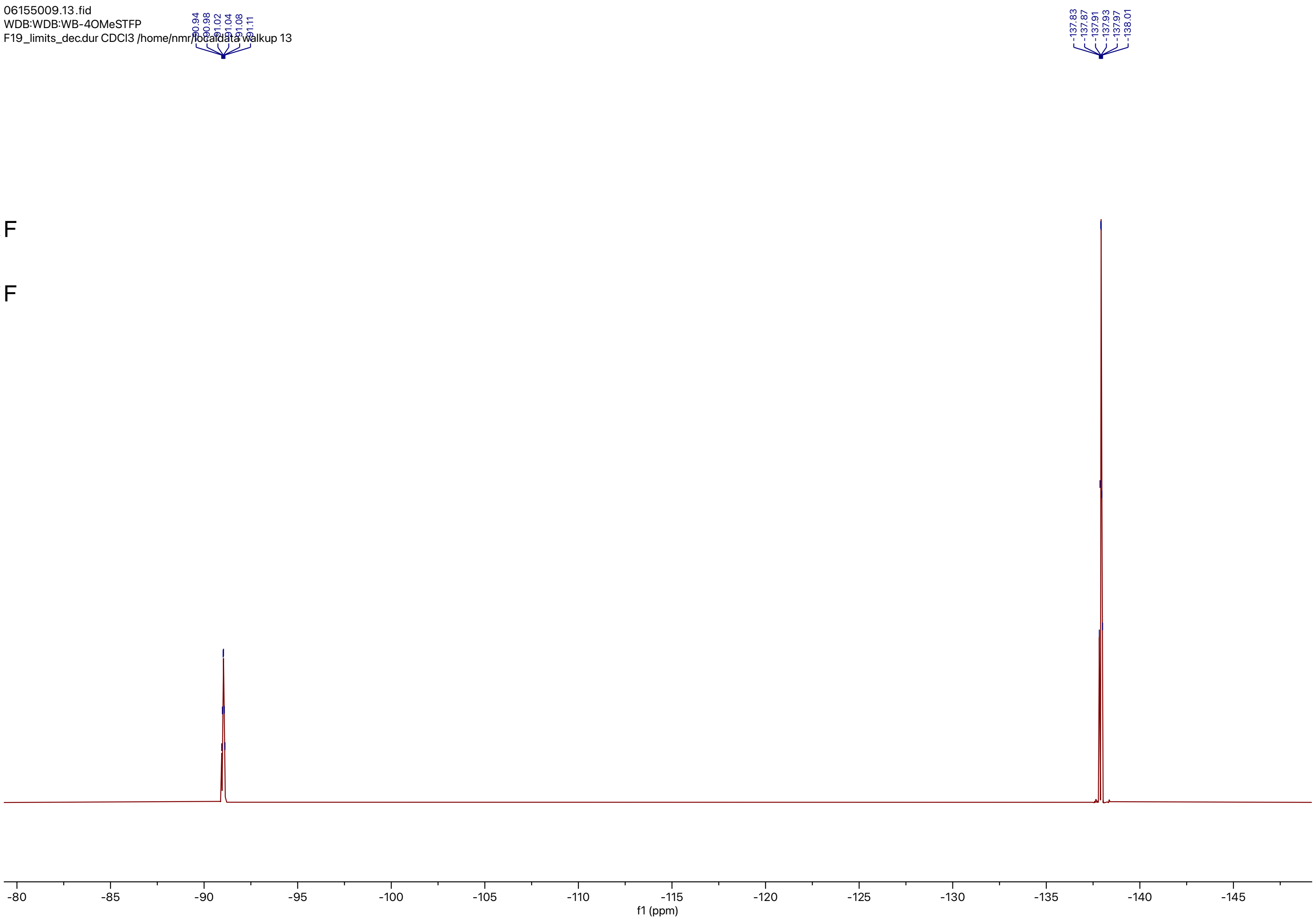

Figure S27. $^{19}F\{^{1}H\}$ NMR spectrum of **10** recorded at 376MHz in $CDCl_3$.

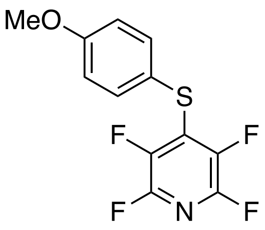

10

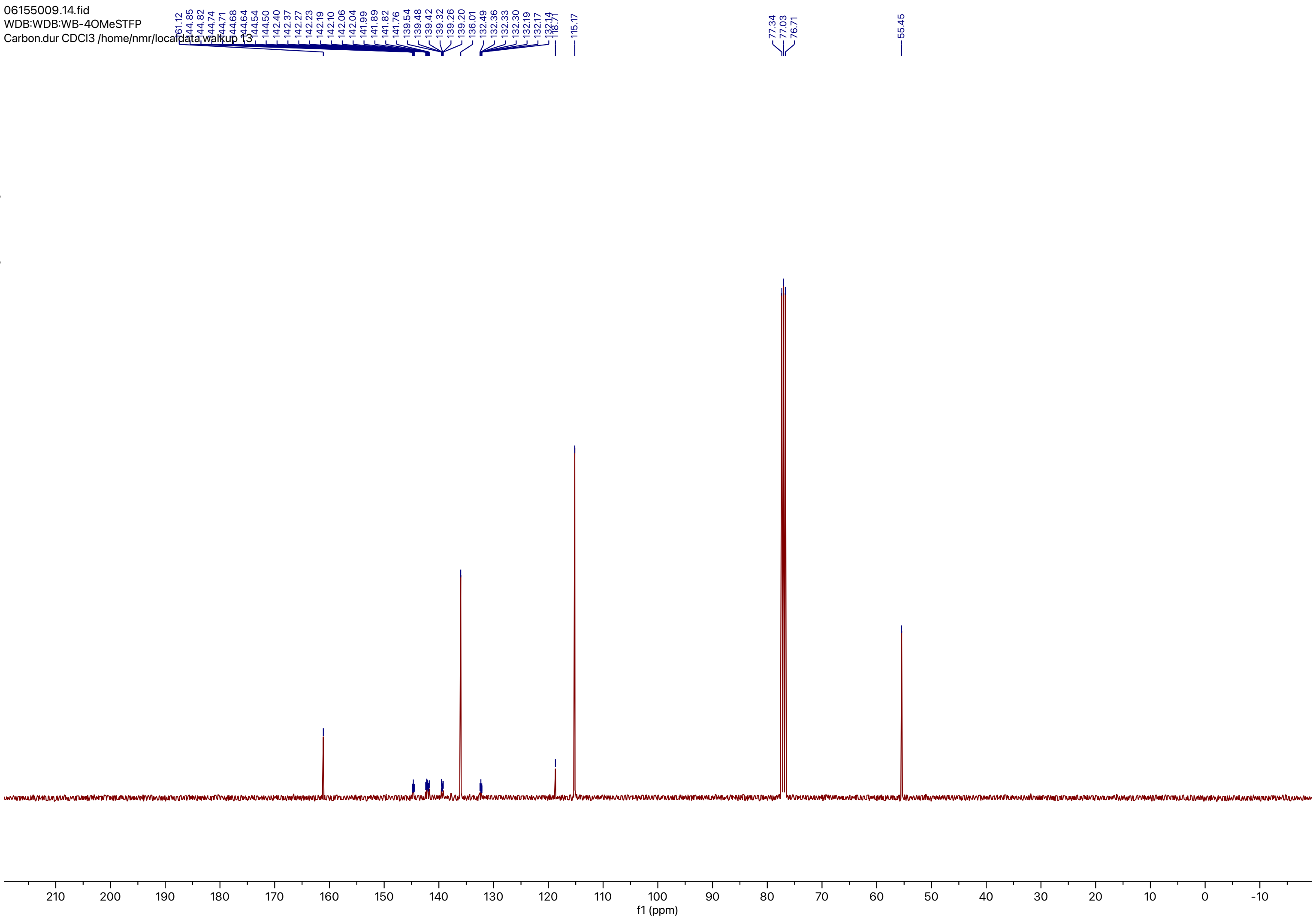

Figure S28. $^{13}C\{^{1}H\}$ NMR spectrum of **10** recorded at 101 MHz in $CDCl_3$.

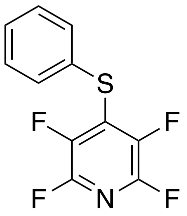

11

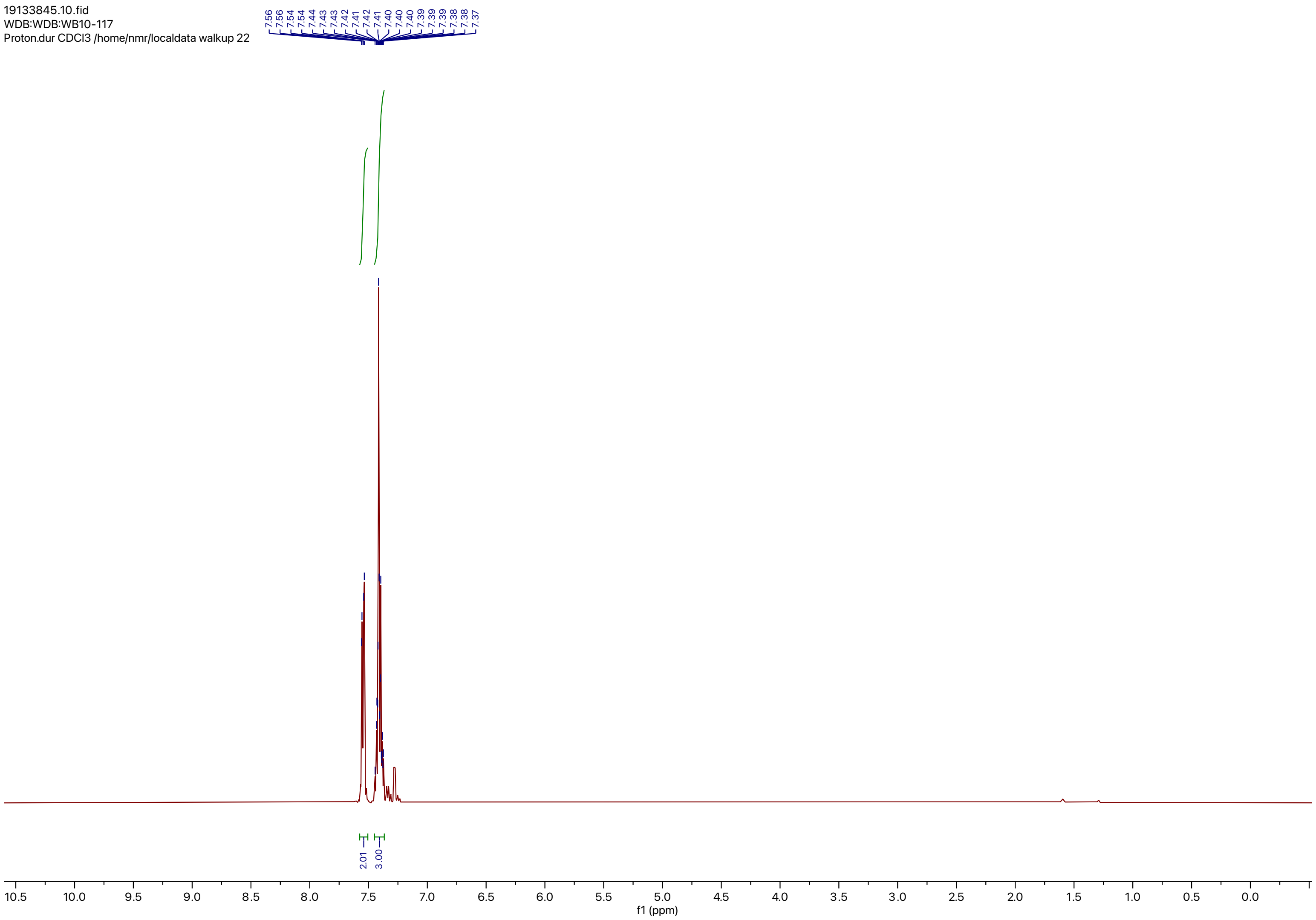

Figure S29. ^{1}H NMR spectrum of **11** recorded at 400 MHz in $CDCl_3$.

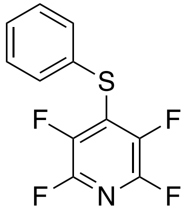

11

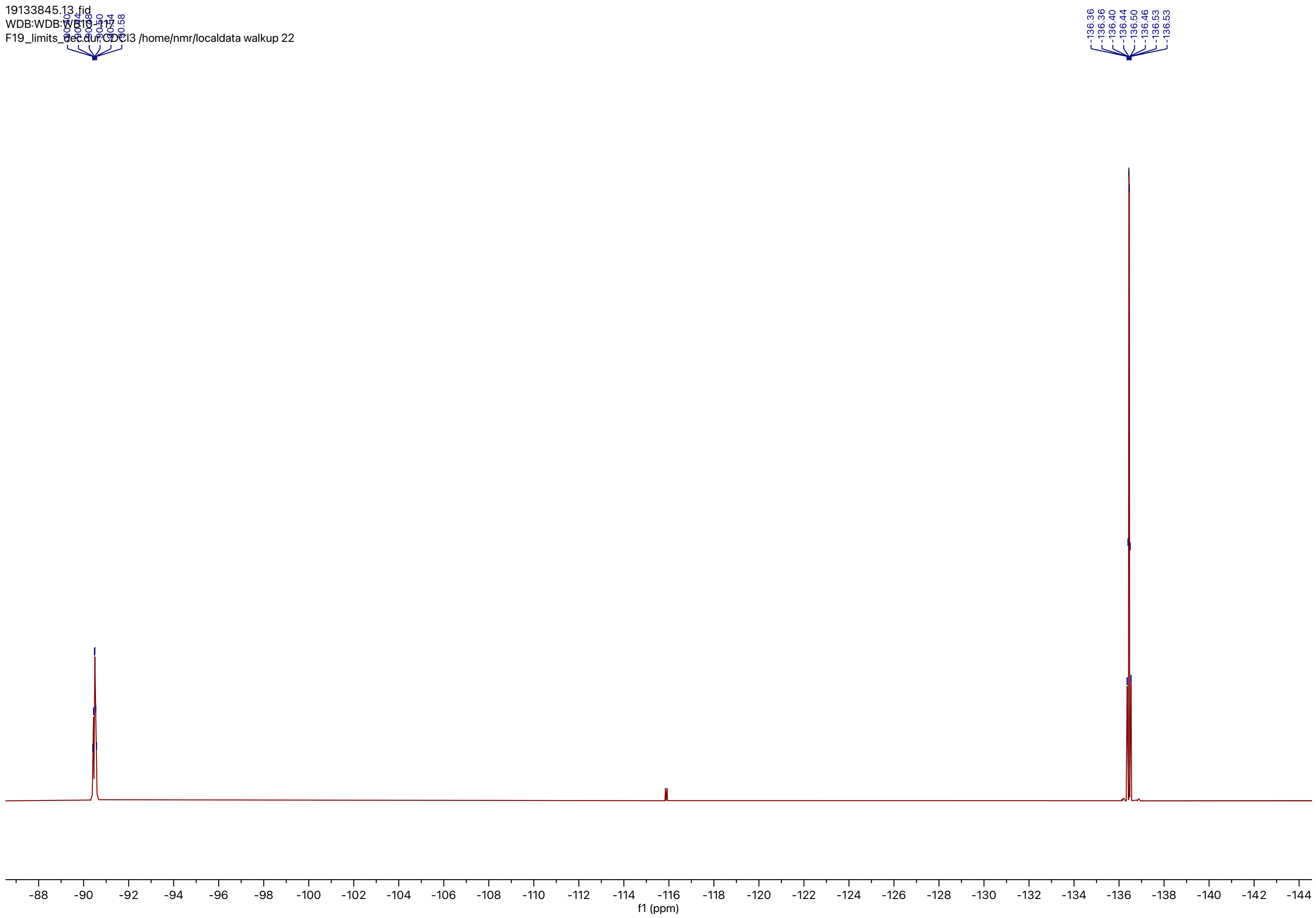

Figure S30. $^{19}F\{^{1}H\}$ NMR spectrum of **11** recorded at 376 MHz in $CDCl_3$.

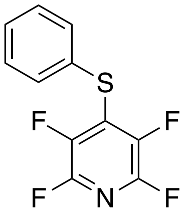

11

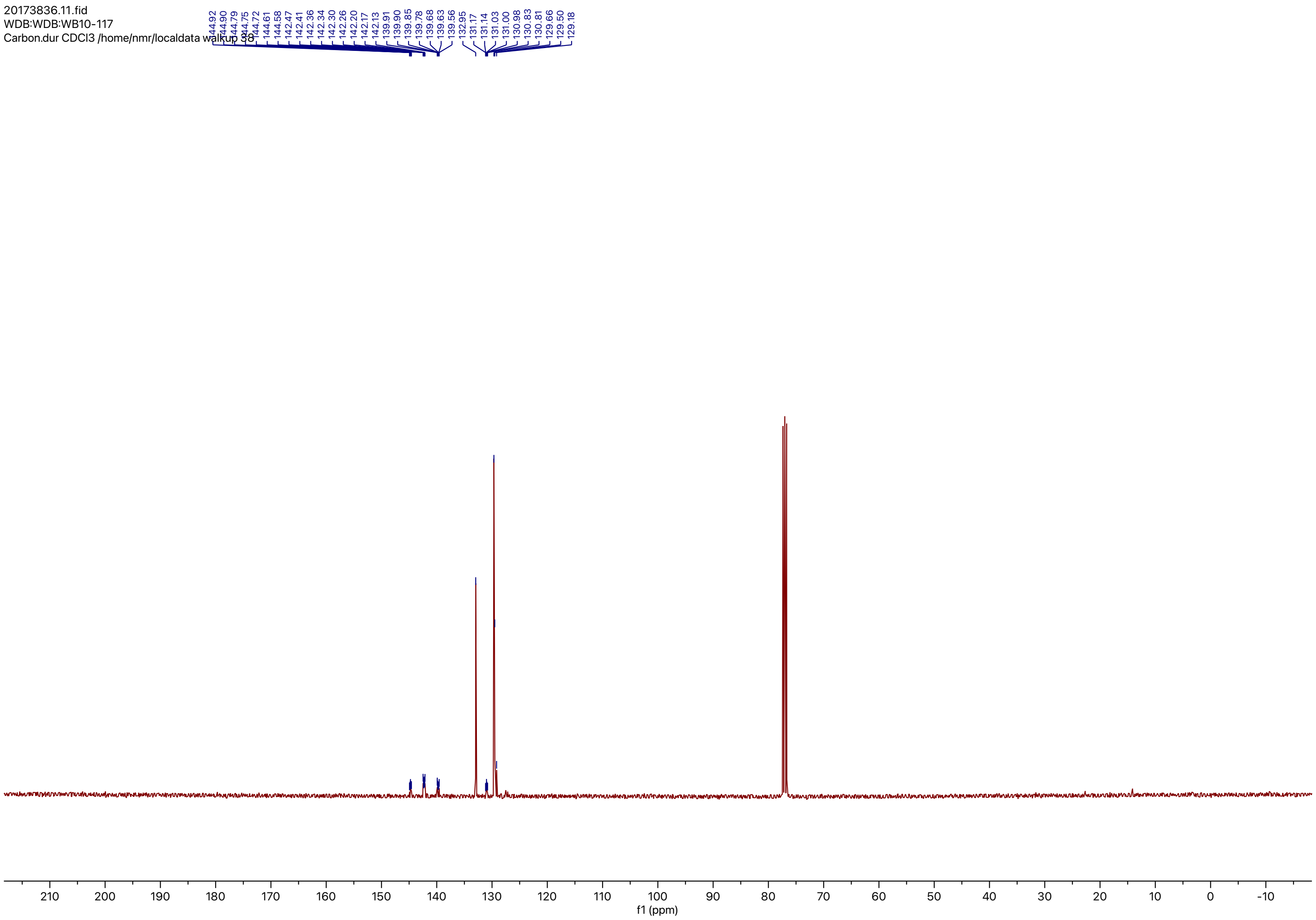

Figure S31. $^{13}C\{^{1}H\}$ NMR spectrum of **11** recorded at 101 MHz in $CDCl_3$.

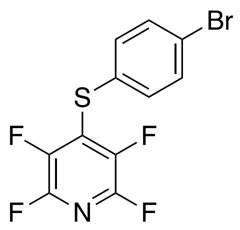

12

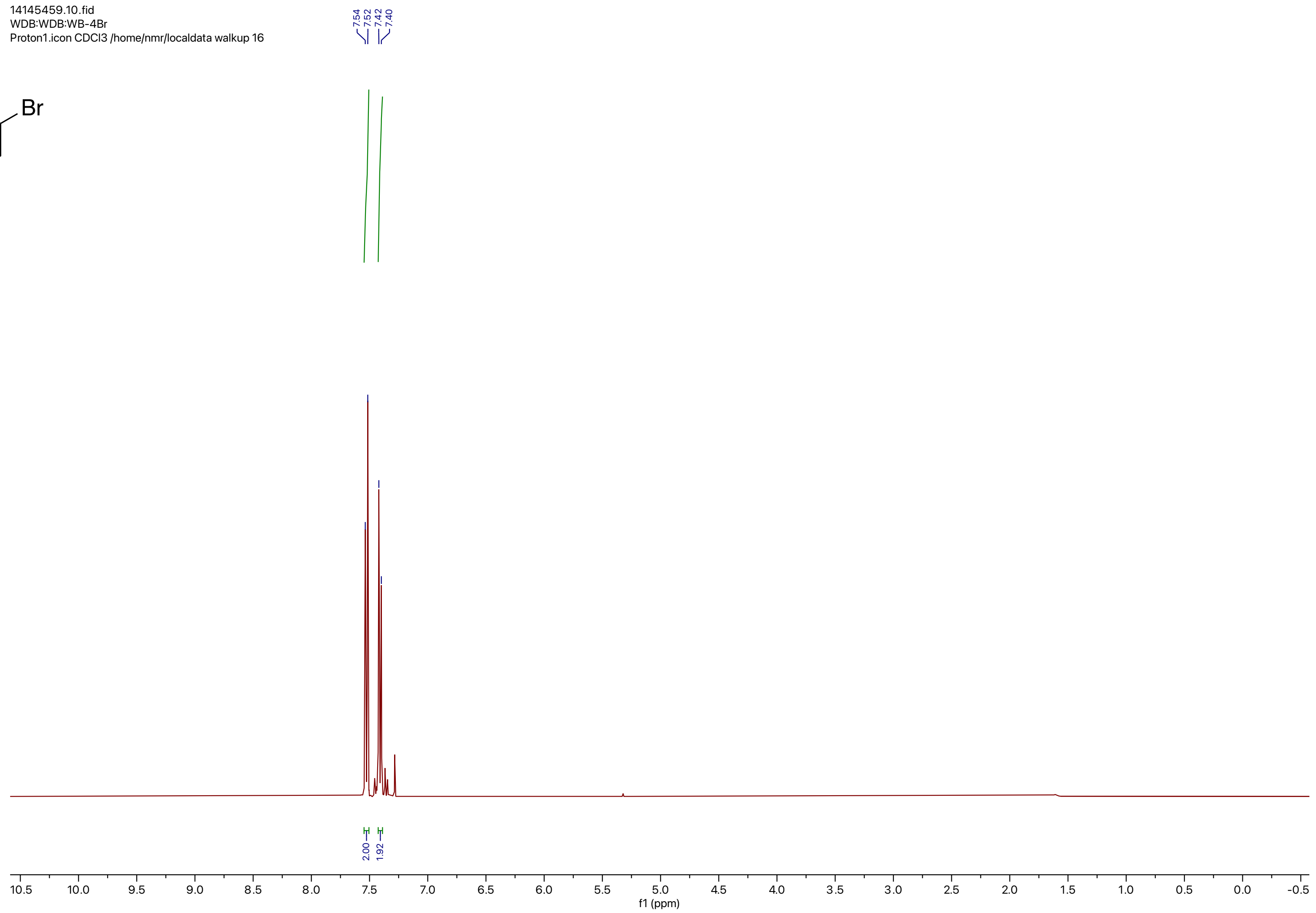

Figure S32. ^{1}H NMR spectrum of **12** recorded at 400 MHz in $CDCl_3$.

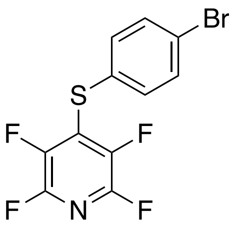

12

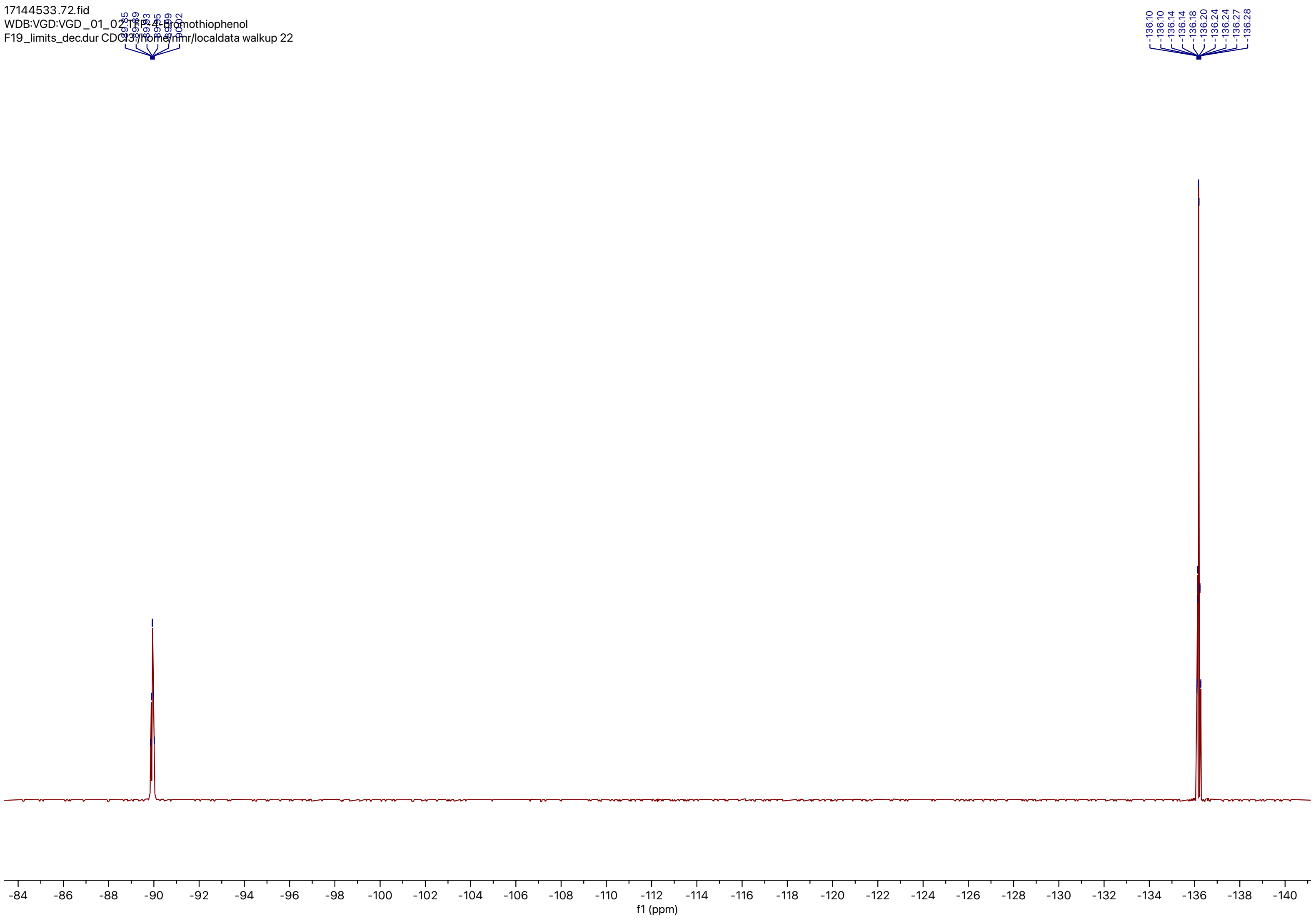

Figure S33. $^{19}F\{^{1}H\}$ NMR spectrum of **12** recorded at 376 MHz in $CDCl_3$.

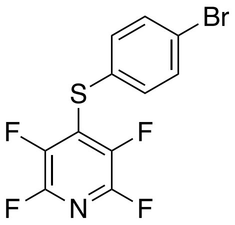

12

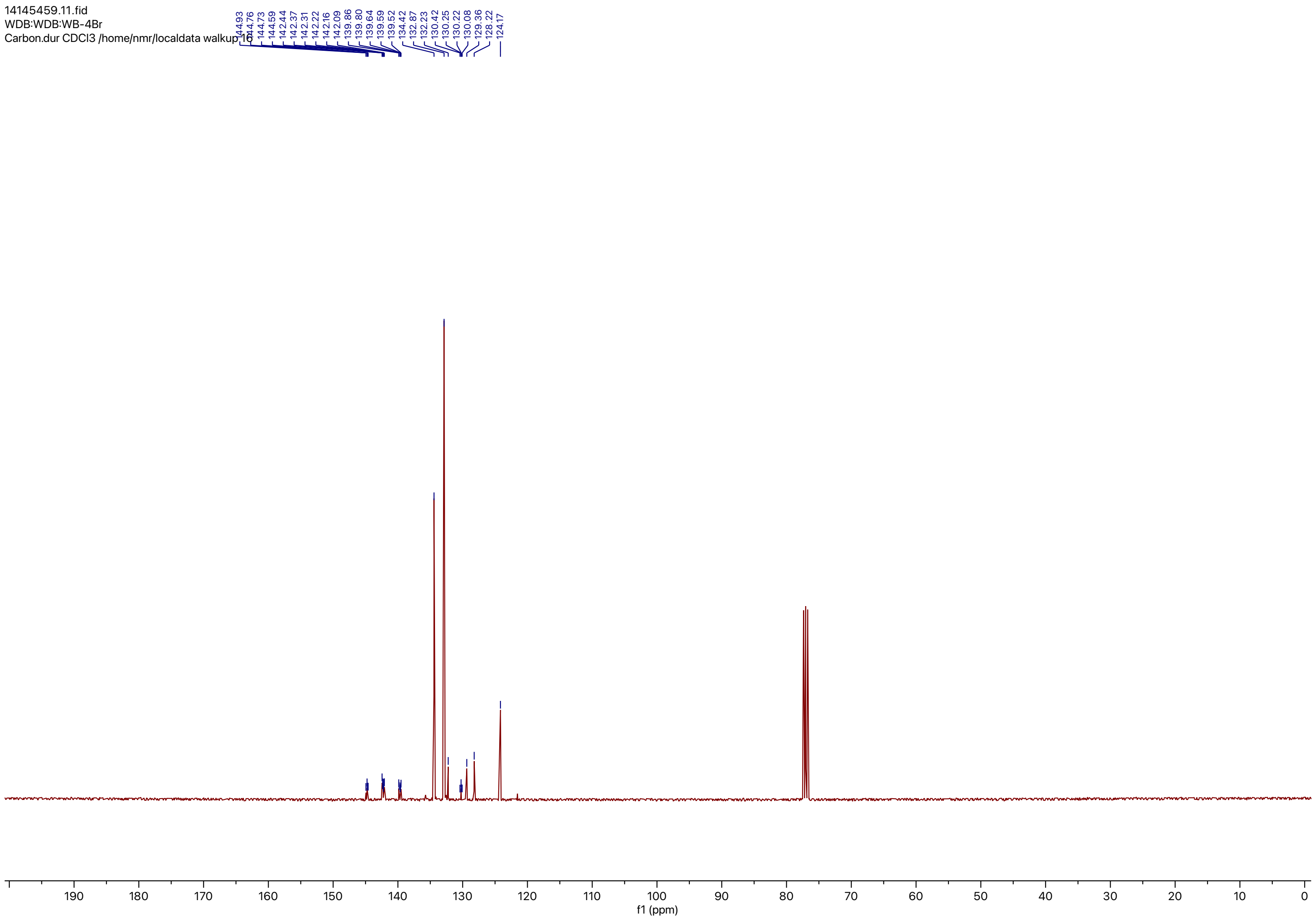

Figure S34. $^{13}C\{^{1}H\}$ NMR spectrum of **12** recorded at 101 MHz in $CDCl_3$.

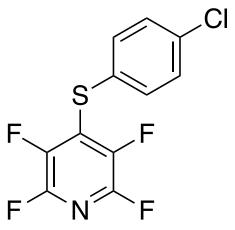

13

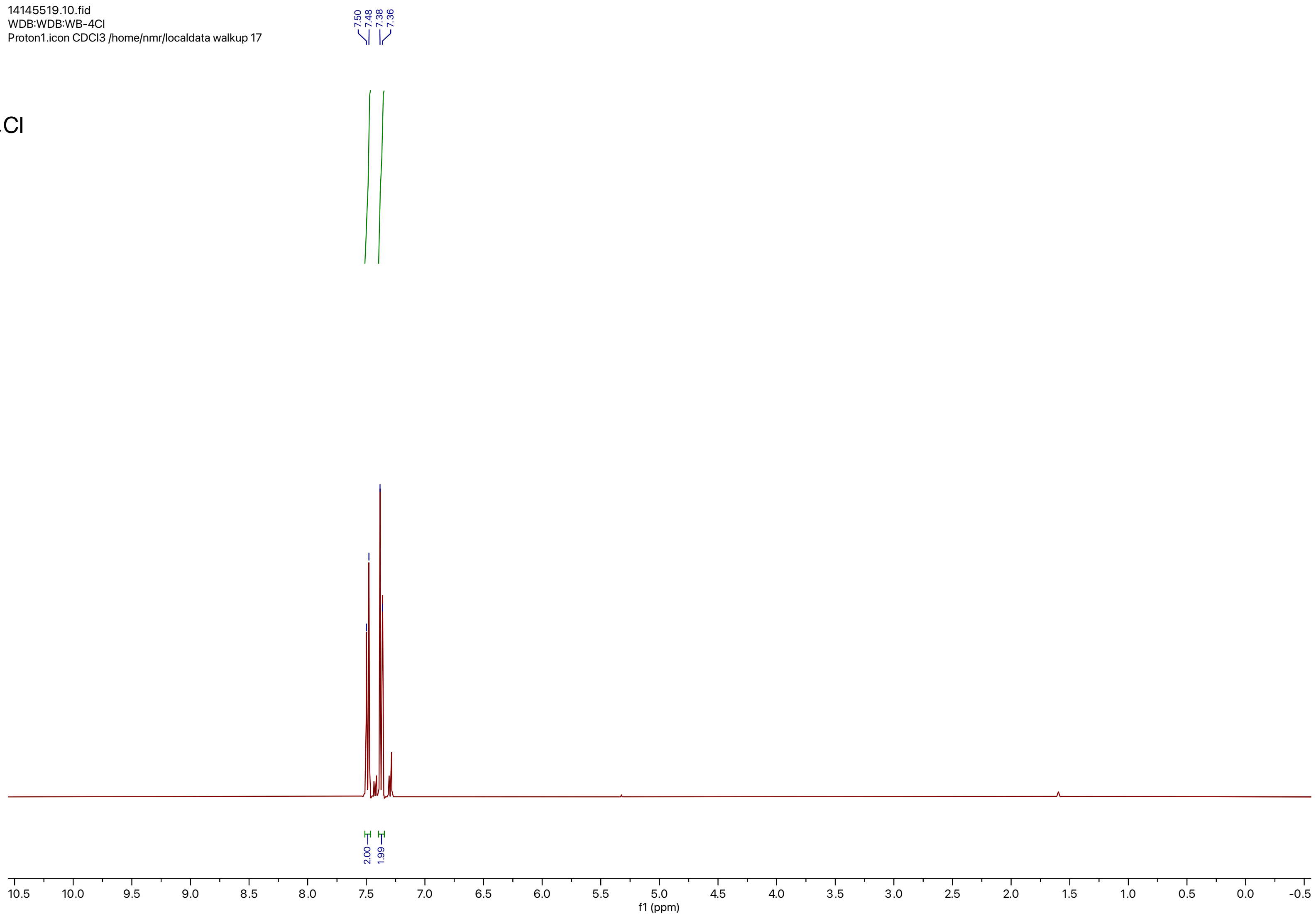

Figure S35. ^{1}H NMR spectrum of **13** recorded at 400 MHz in $CDCl_3$.

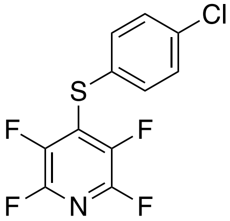

13

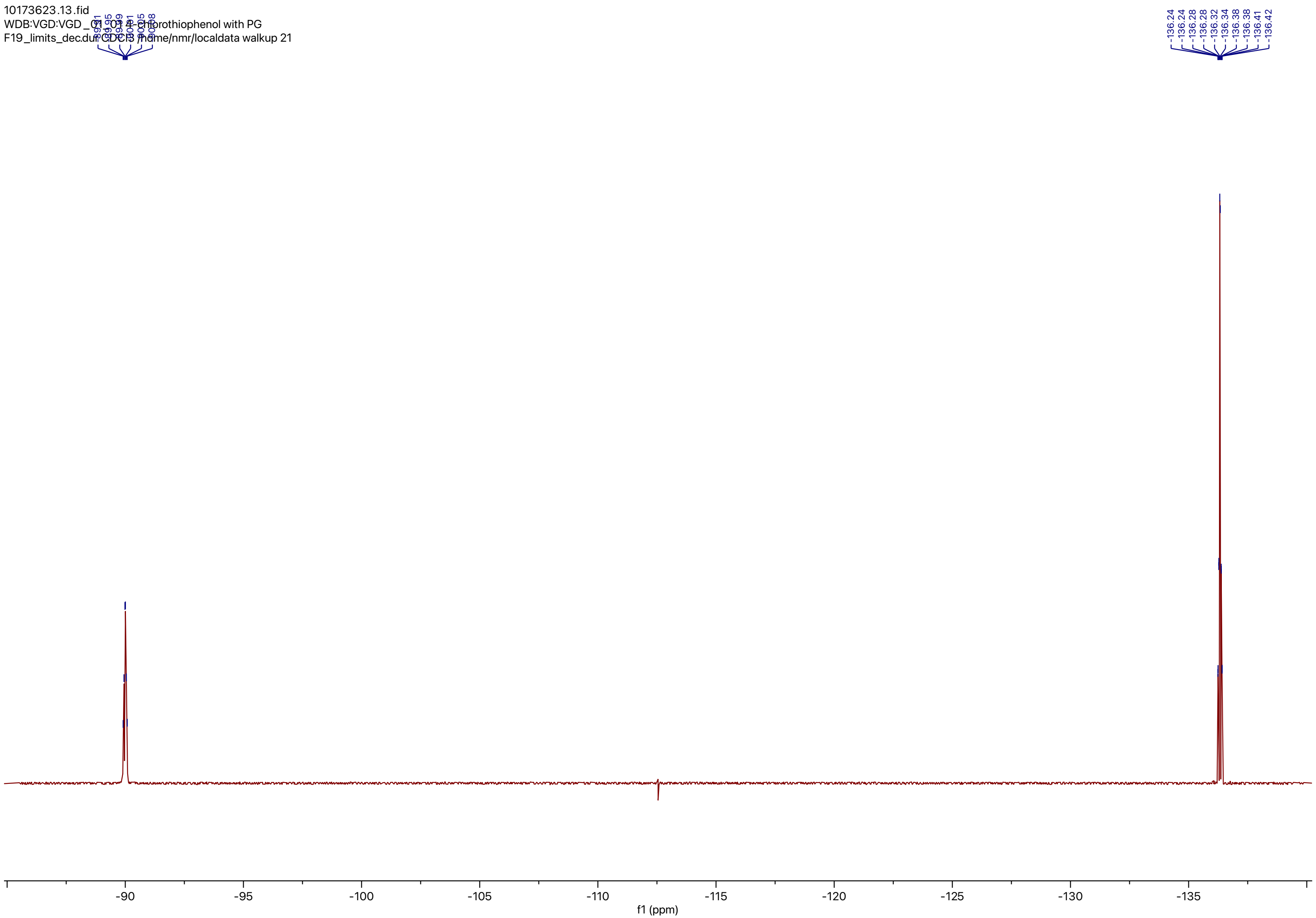

Figure S36. $^{19}F\{^1H\}$ NMR spectrum of **13** recorded at 376 MHz in $CDCl_3$.

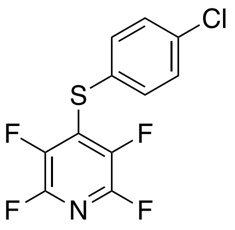

13

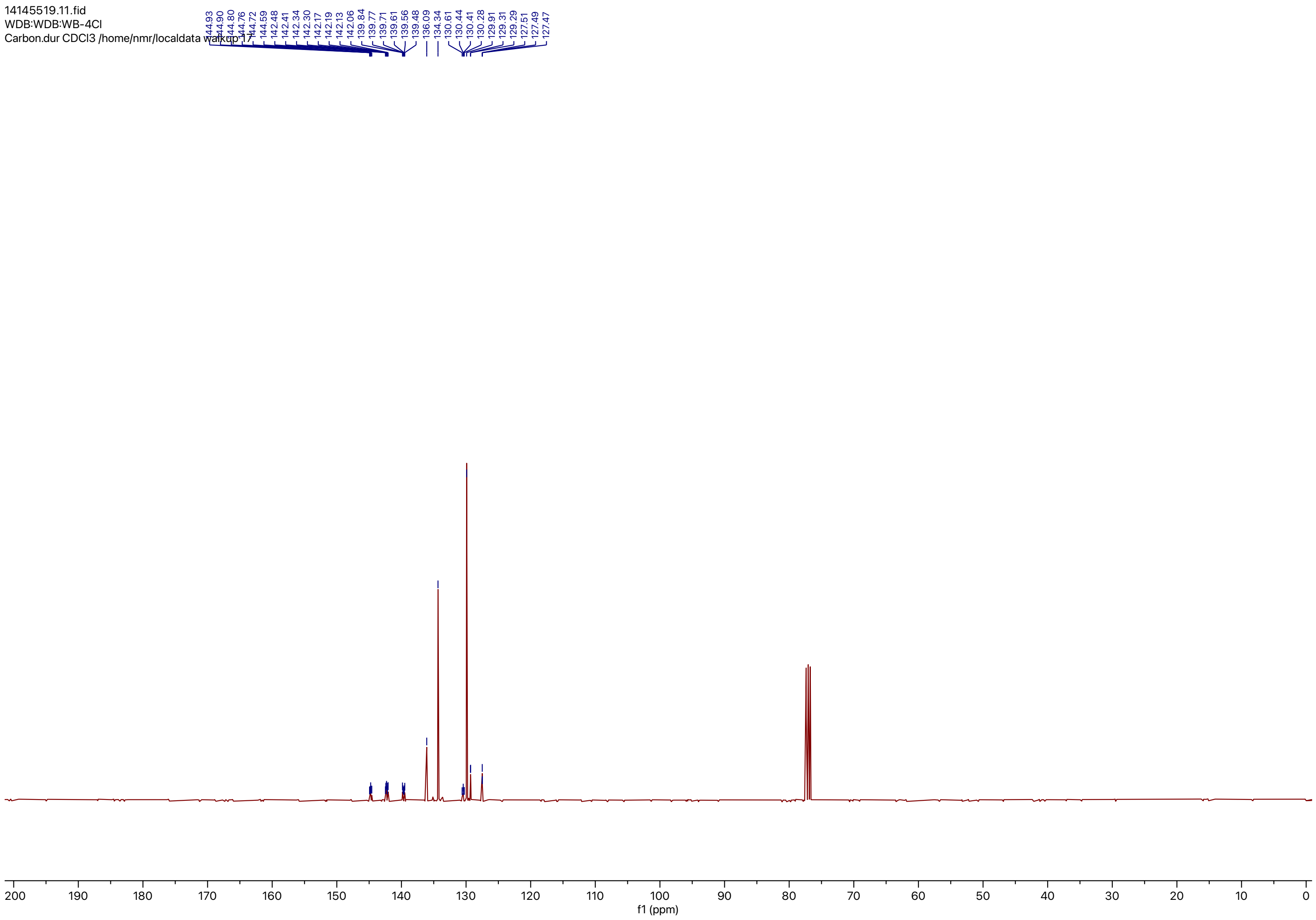

Figure S37. $^{13}C\{^{1}H\}$ NMR spectrum of **13** recorded at 101 MHz in $CDCl_3$.

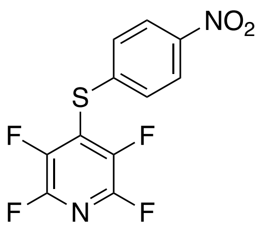

14

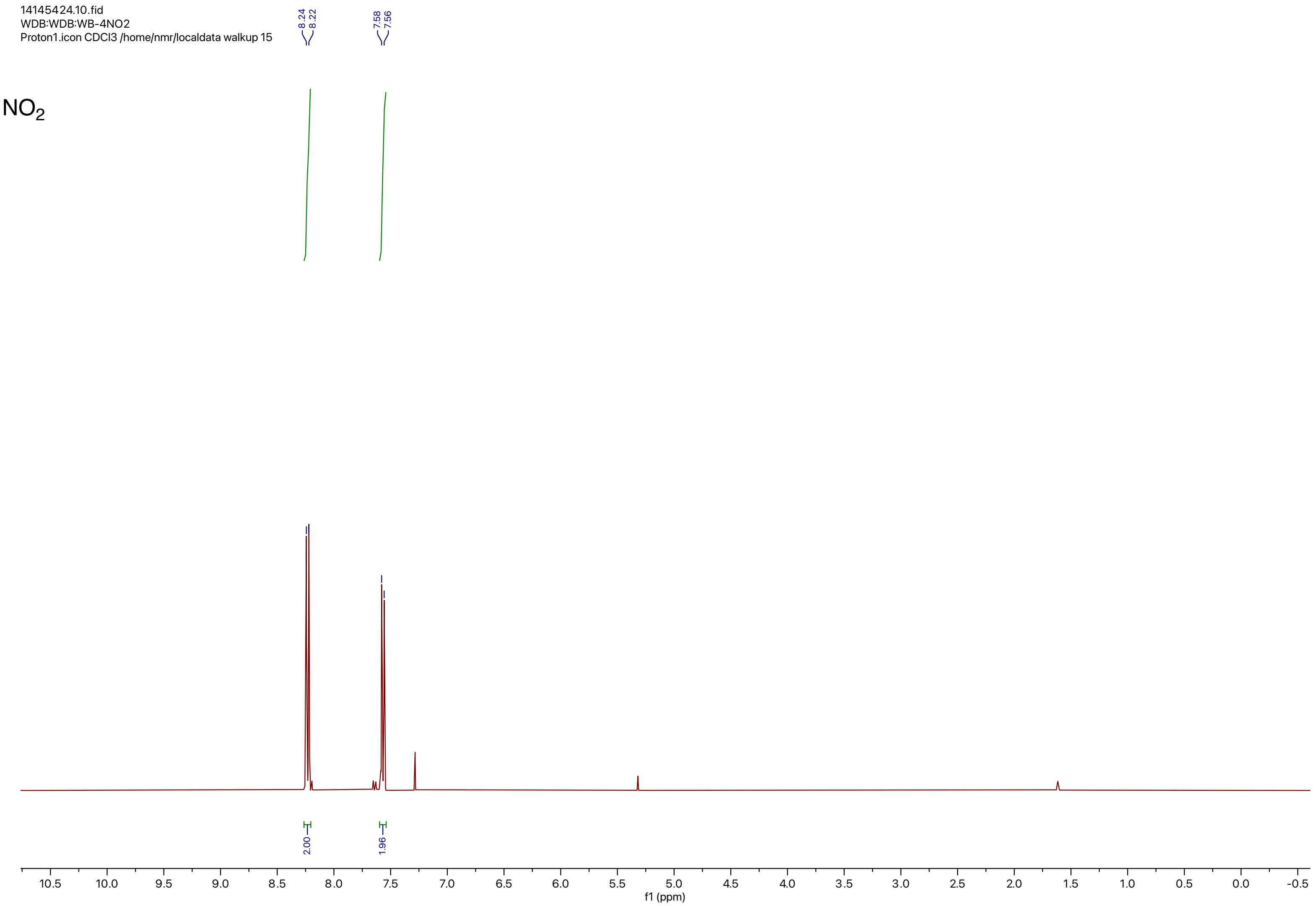

Figure S38. ^{1}H NMR spectrum of **14** recorded at 400 MHz in $CDCl_3$.

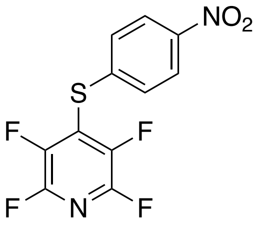

14

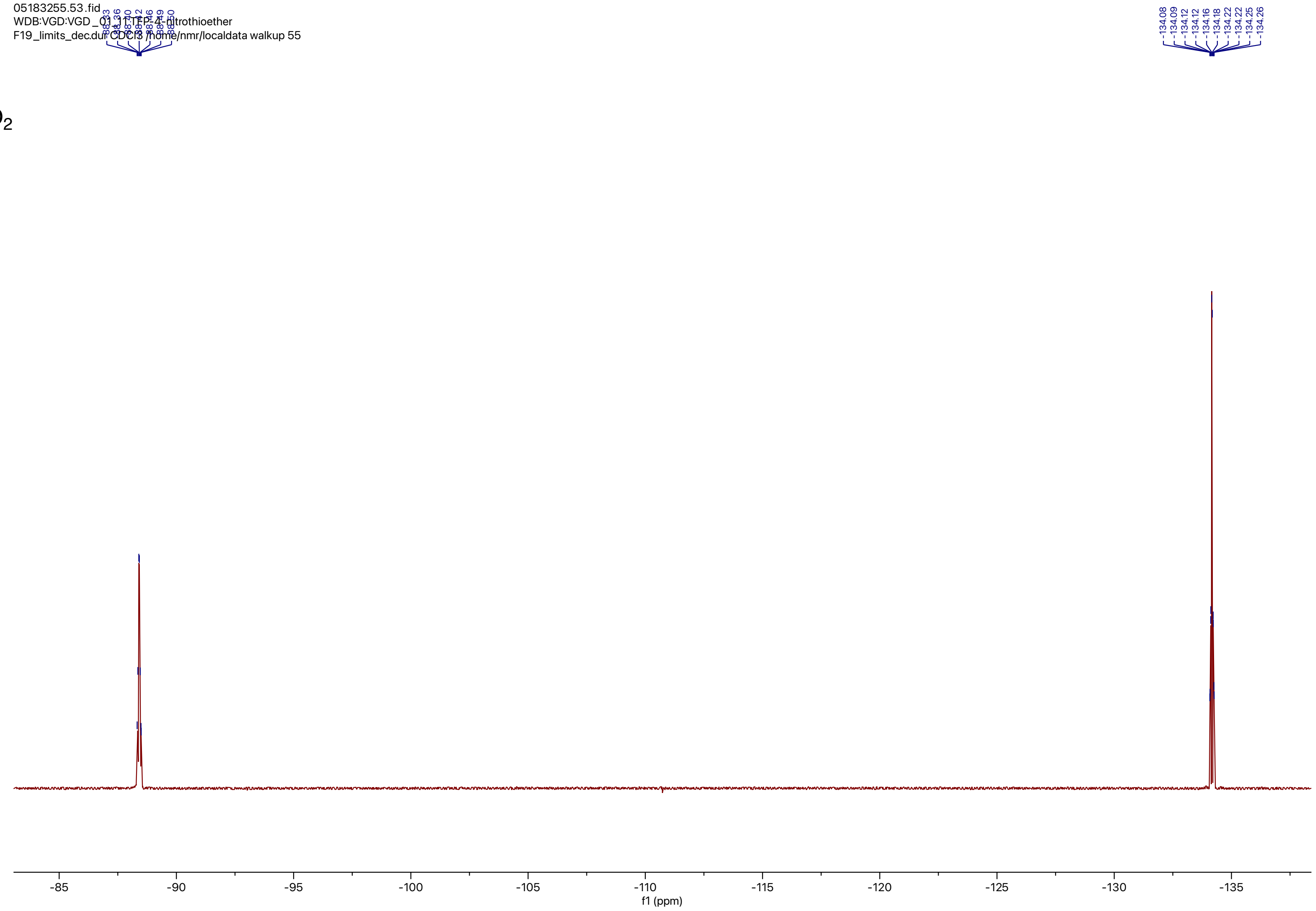

Figure S39. $^{19}F\{^{1}H\}$ NMR spectrum of **14** recorded at 376 MHz in $CDCl_3$.

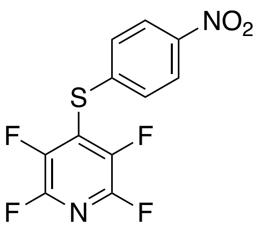

14

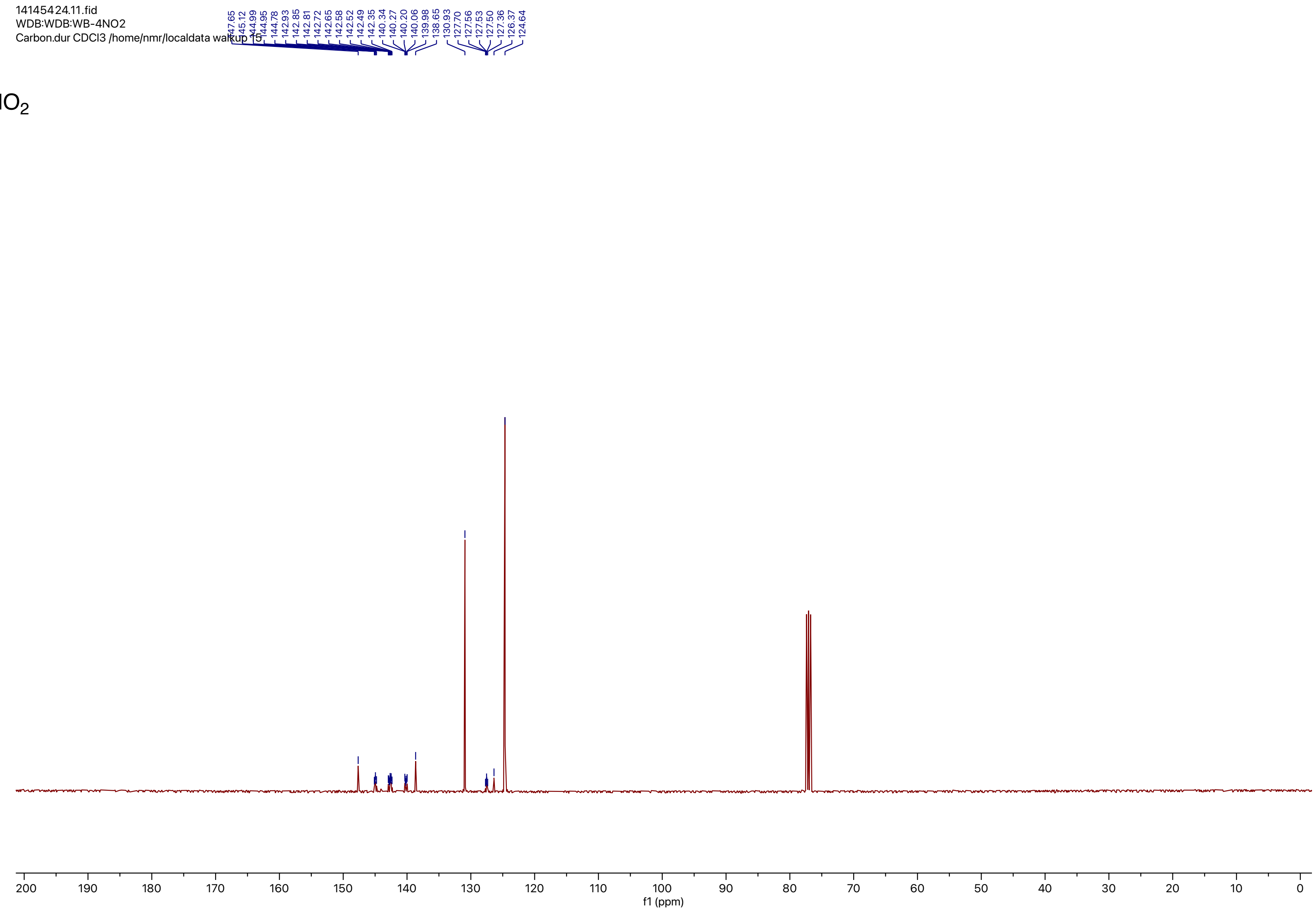

Figure S40. $^{13}C\{^{1}H\}$ NMR spectrum of **14** recorded at 101 MHz in $CDCl_3$.

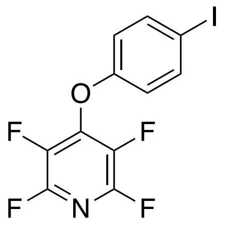

15

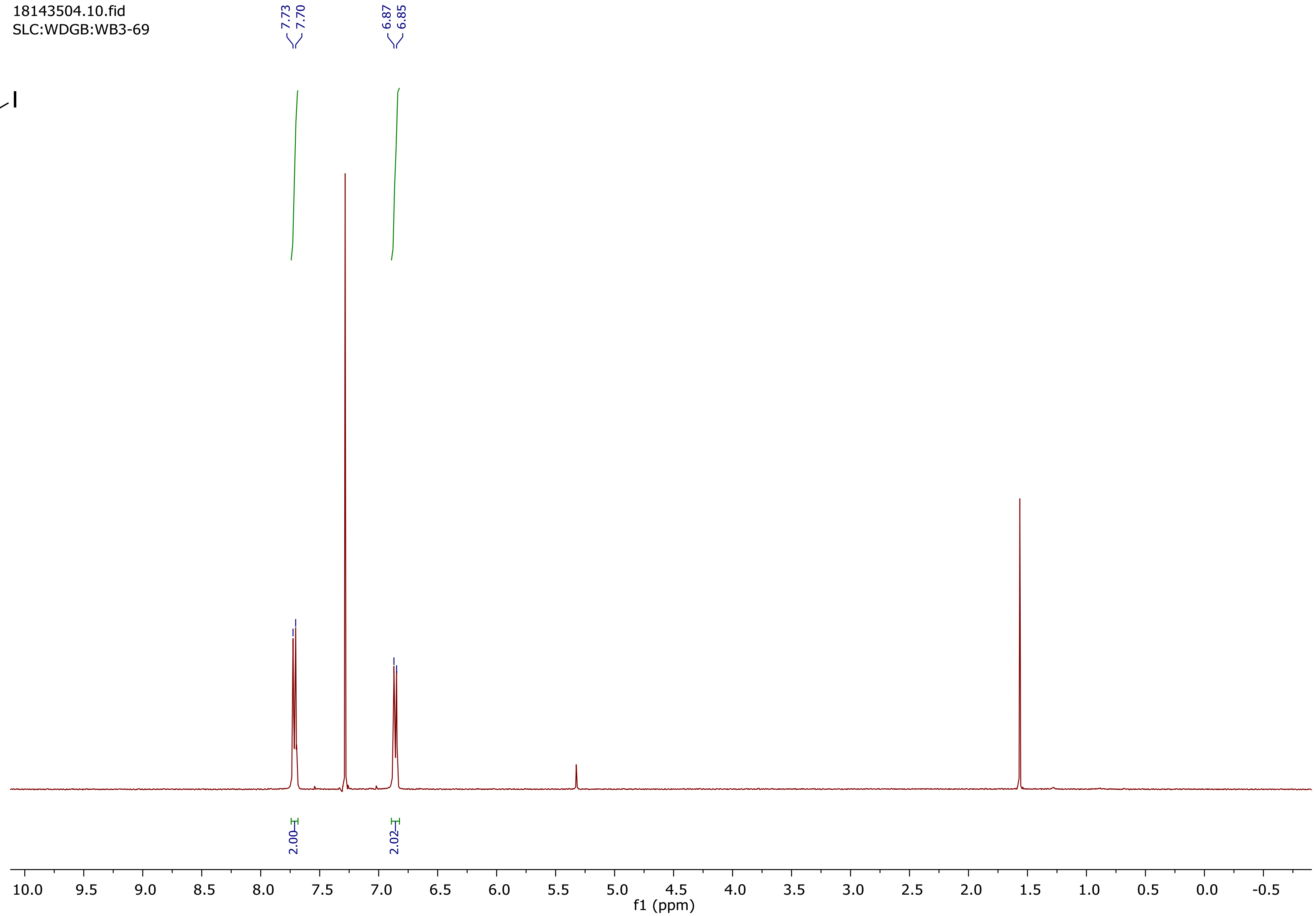

Figure S41. ^{1}H NMR spectrum of **15** recorded at 400 MHz in $CDCl_3$.

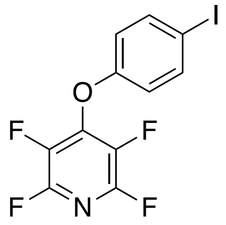

15

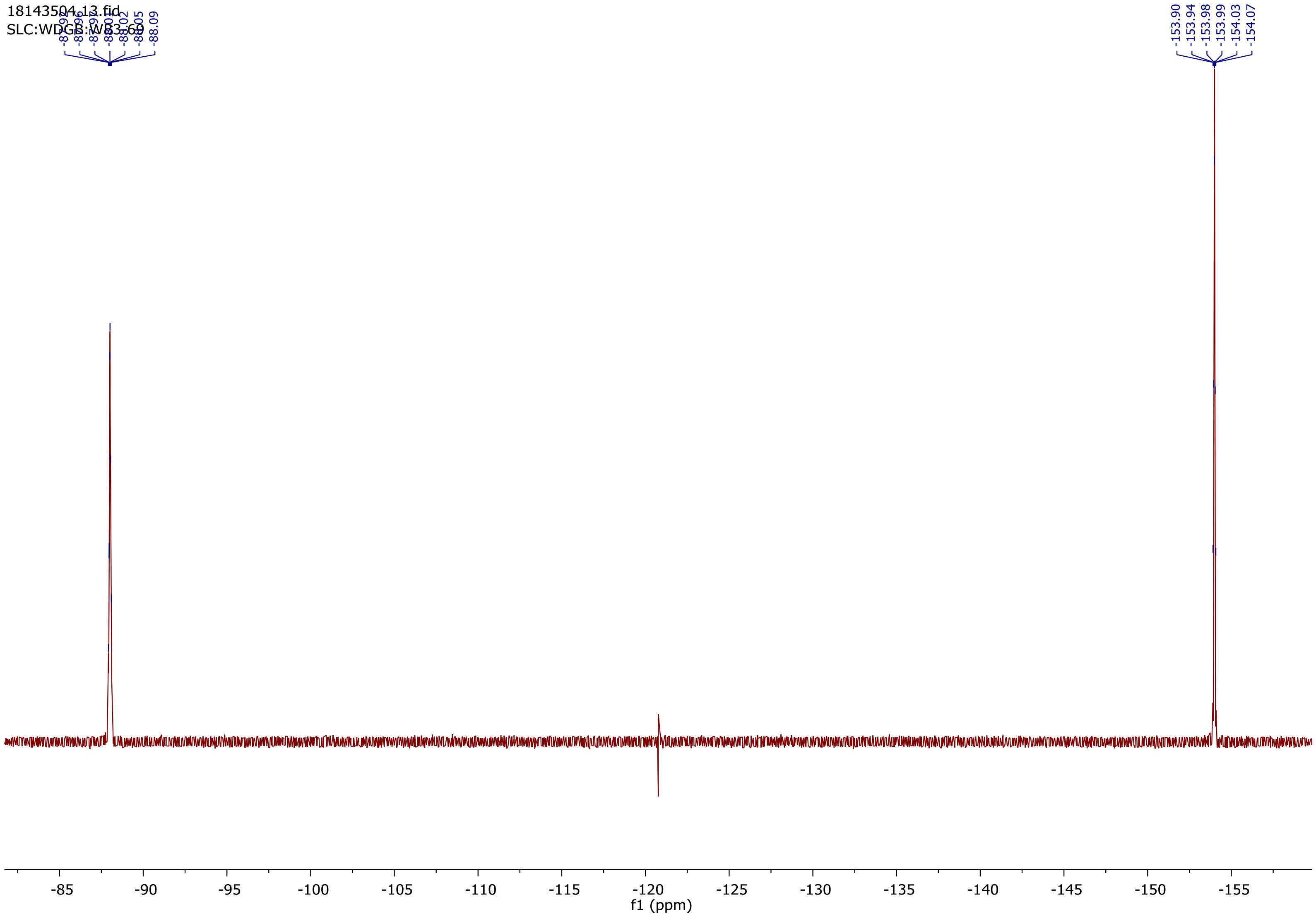

Figure S42. $^{19}F\{^1H\}$ NMR spectrum of **15** recorded at 376 MHz in $CDCl_3$.

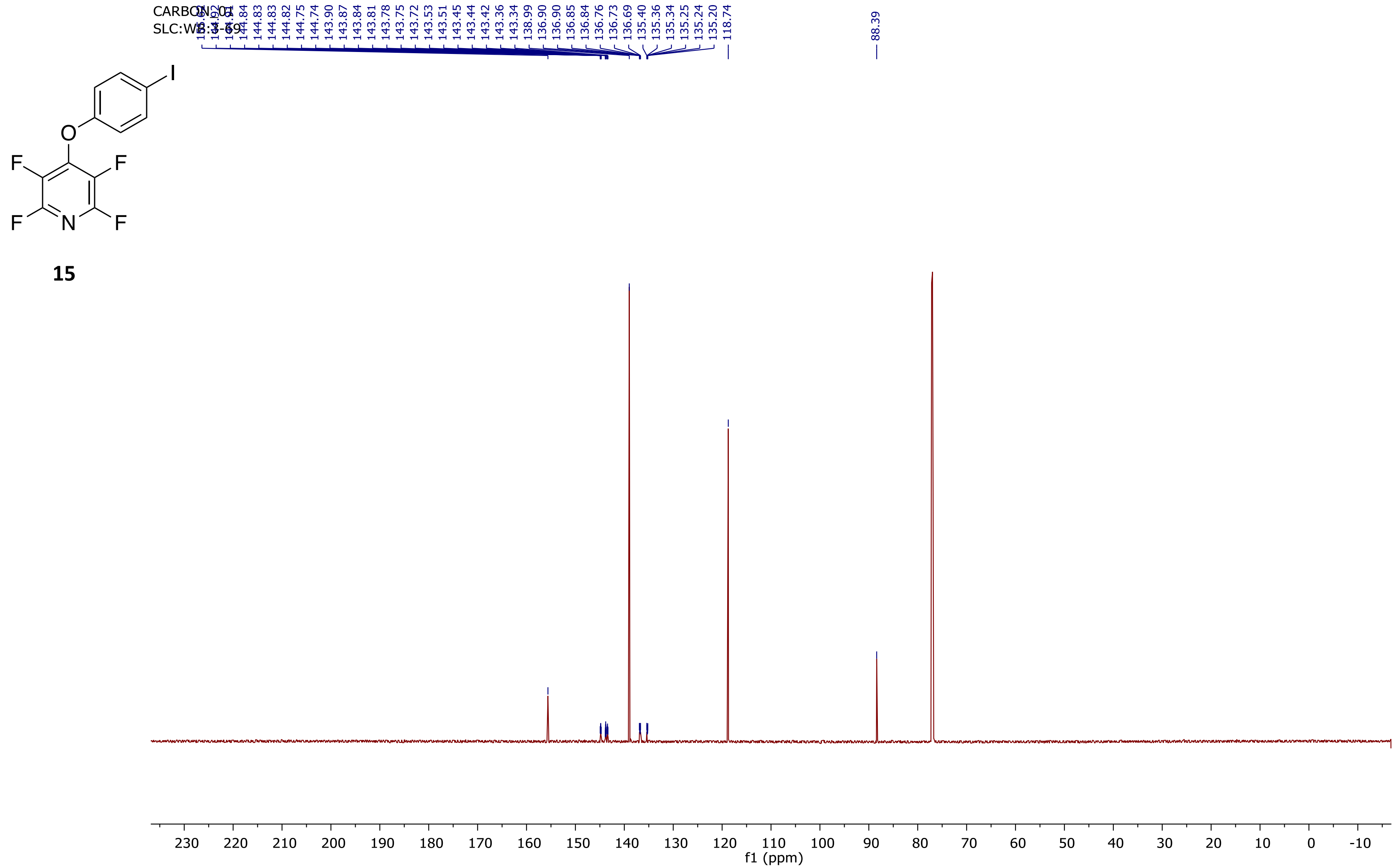

Figure S43. $^{13}C\{^{1}H\}$ NMR spectrum of **15** ecorded at 101 MHz **i**n $CDCl_3$.

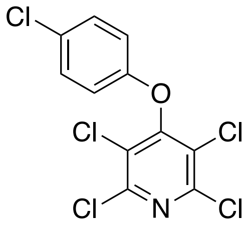

16

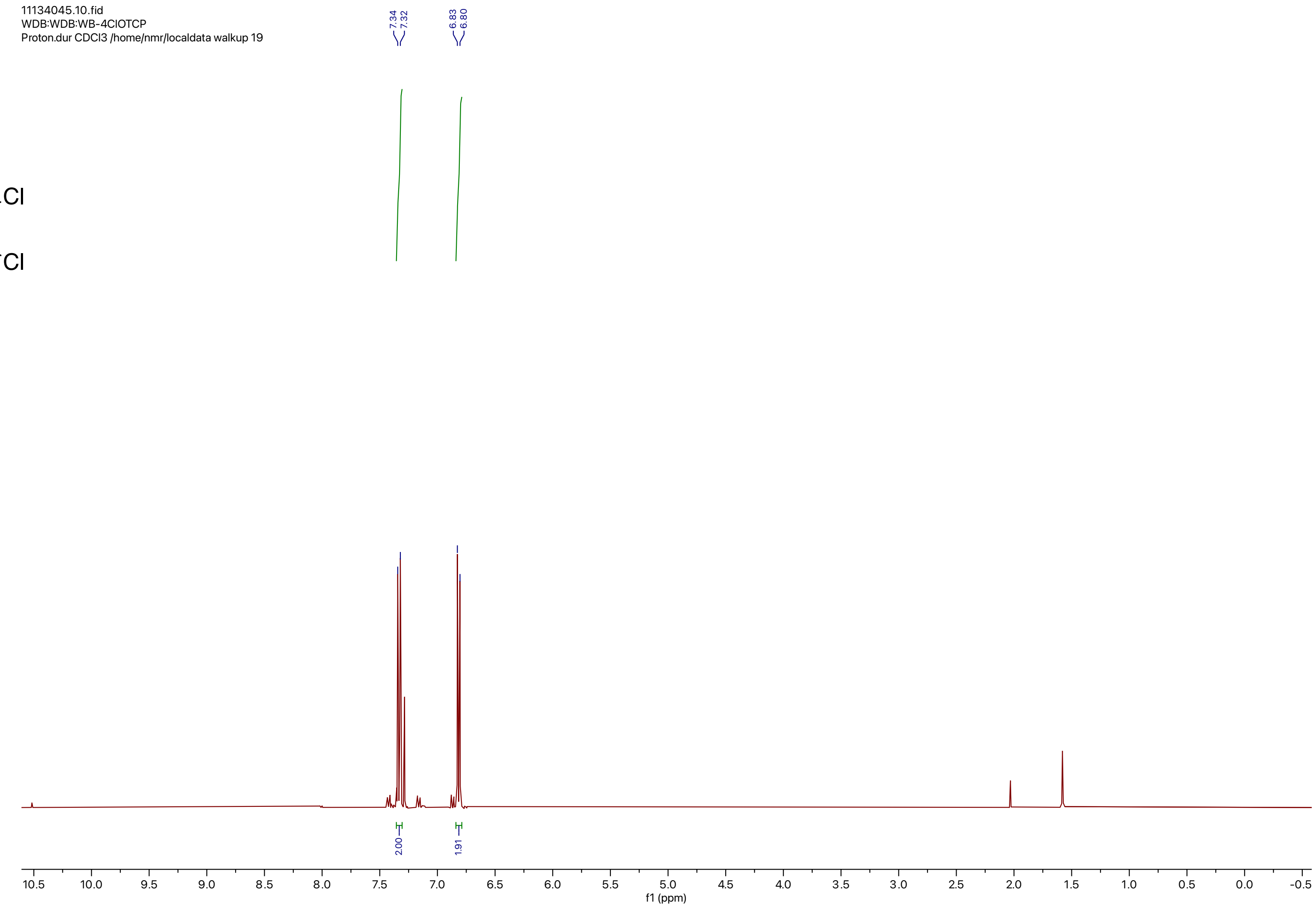

Figure S44. ^{1}H NMR spectrum of **16** recorded at 400 MHz in $CDCl_3$.

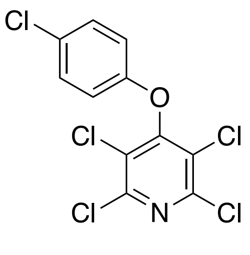

16

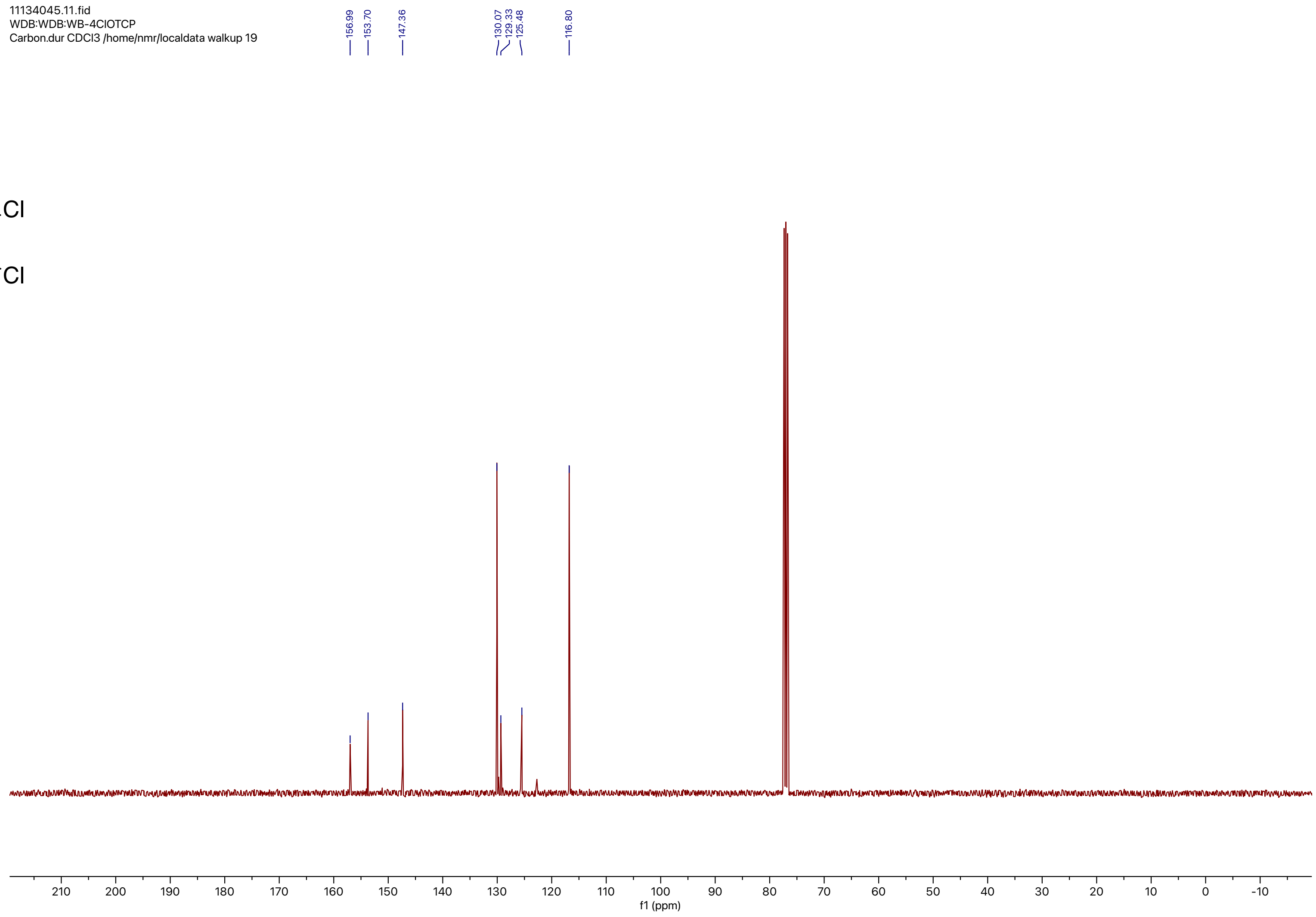

Figure S45. $^{13}C\{^1H\}$ NMR spectrum of **16** recorded at 101 MHz in $CDCl_3$.

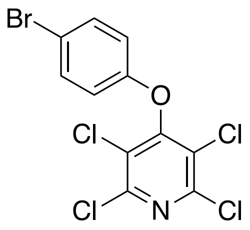

17

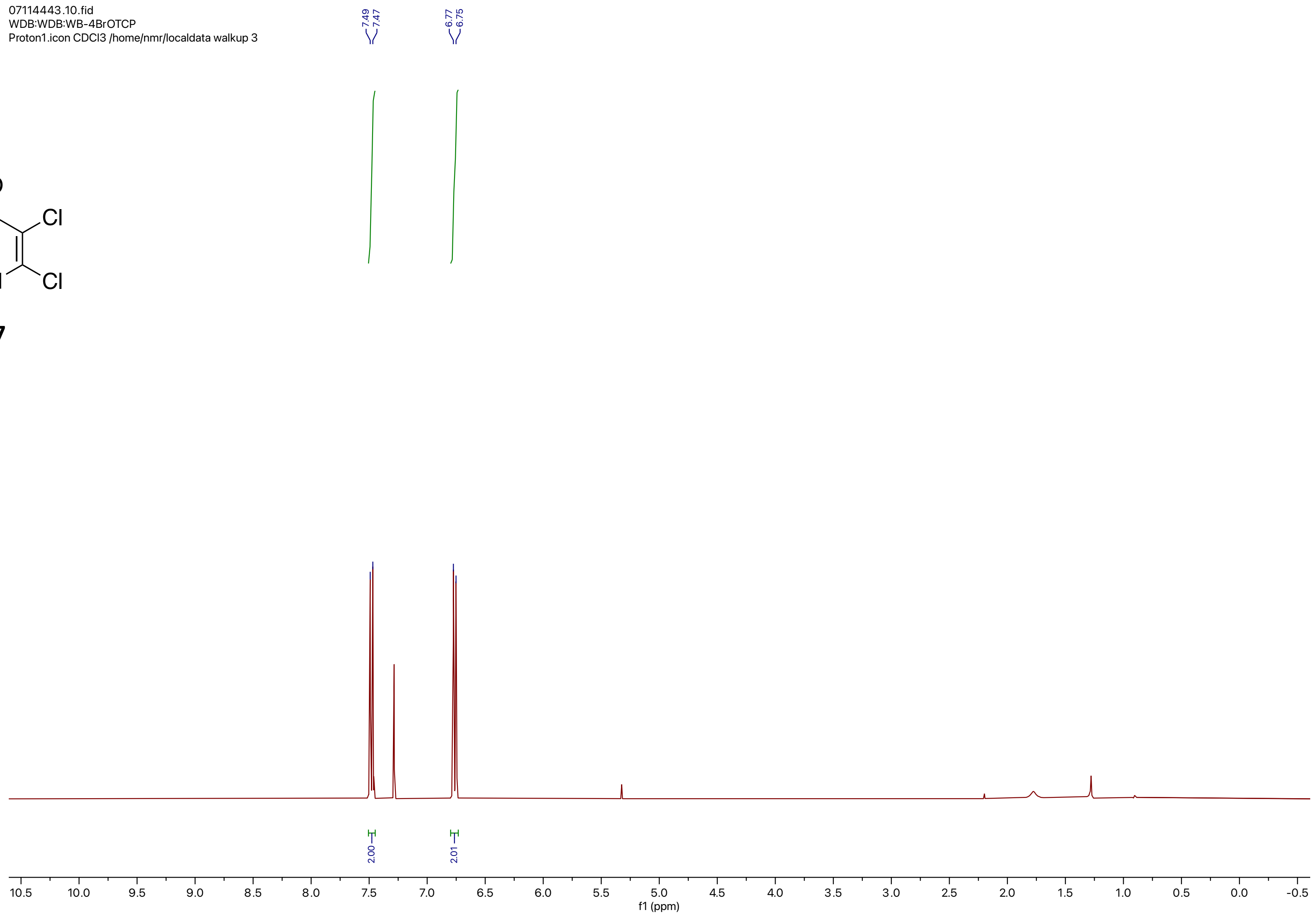

Figure S46. ^{1}H NMR spectrum of **17** recorded at 400 MHz in $CDCl_3$.

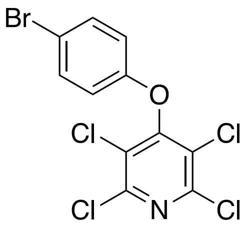

17

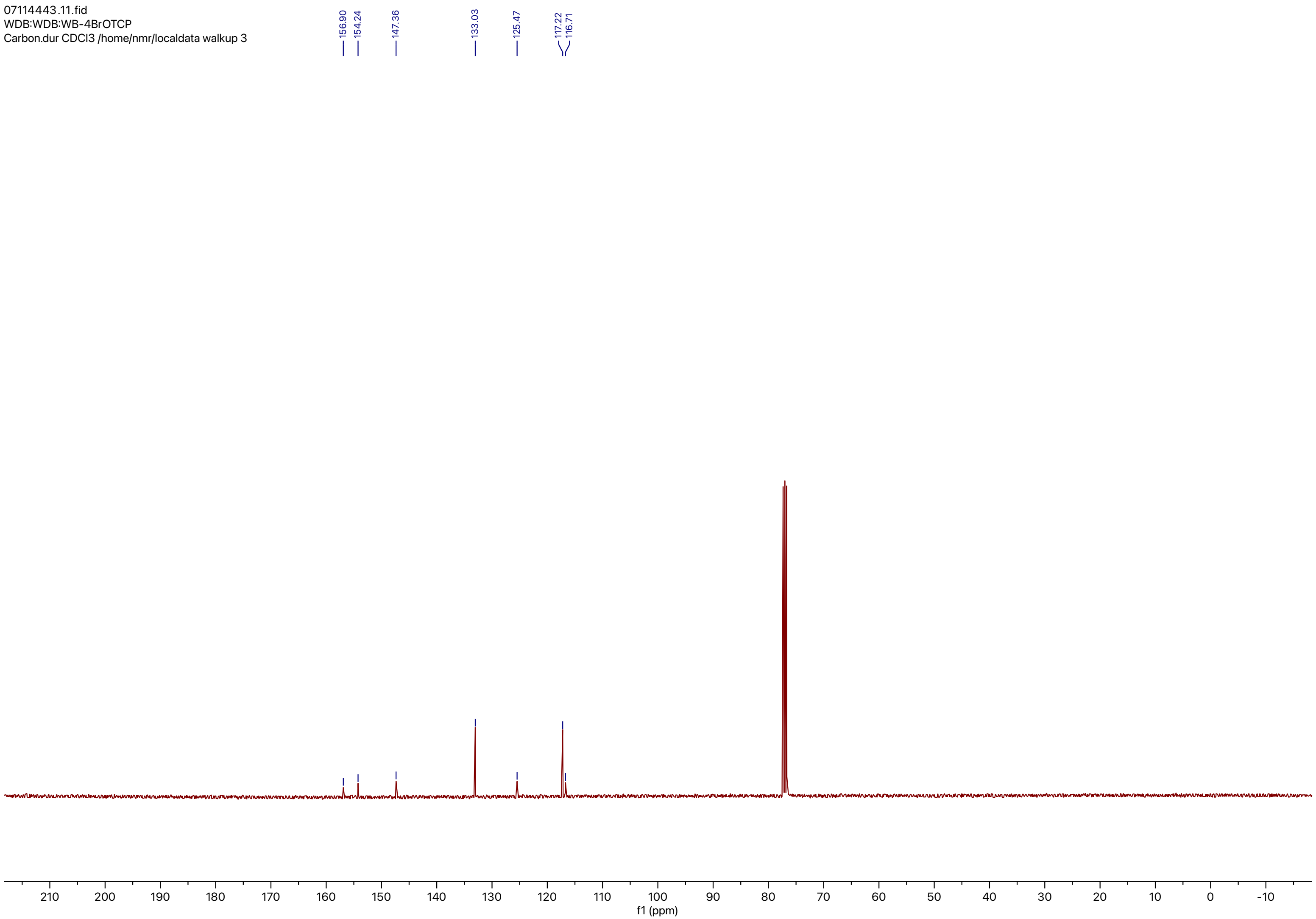

Figure S47. $^{13}C\{^1H\}$ NMR spectrum of **17** recorded at 101 MHz in $CDCl_3$.

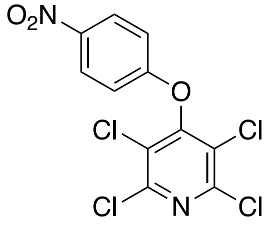

18

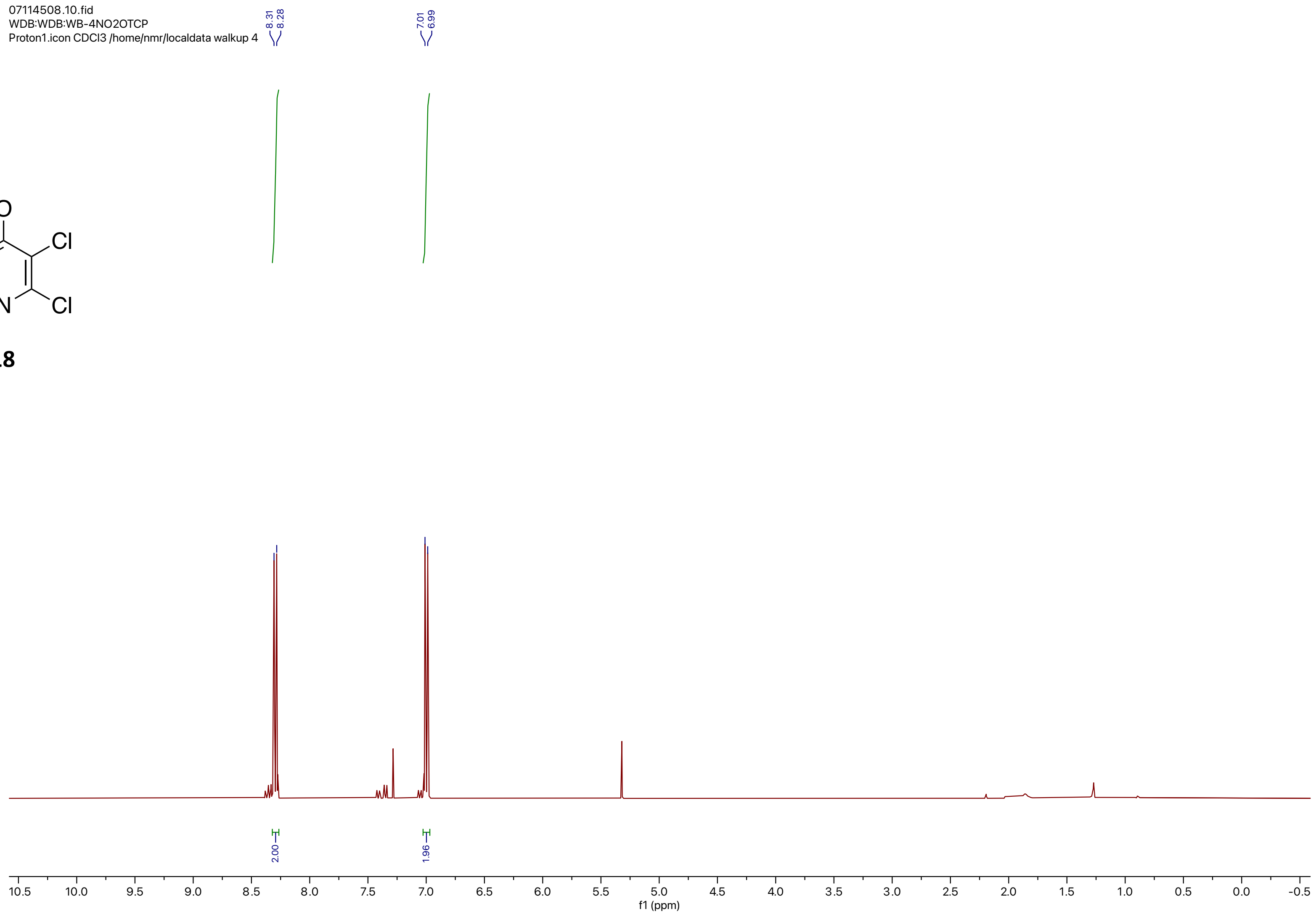

Figure S48. ^{1}H NMR spectrum of **18** recorded at 400 MHz in $CDCl_3$.

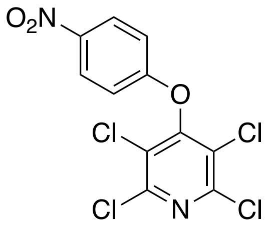

18

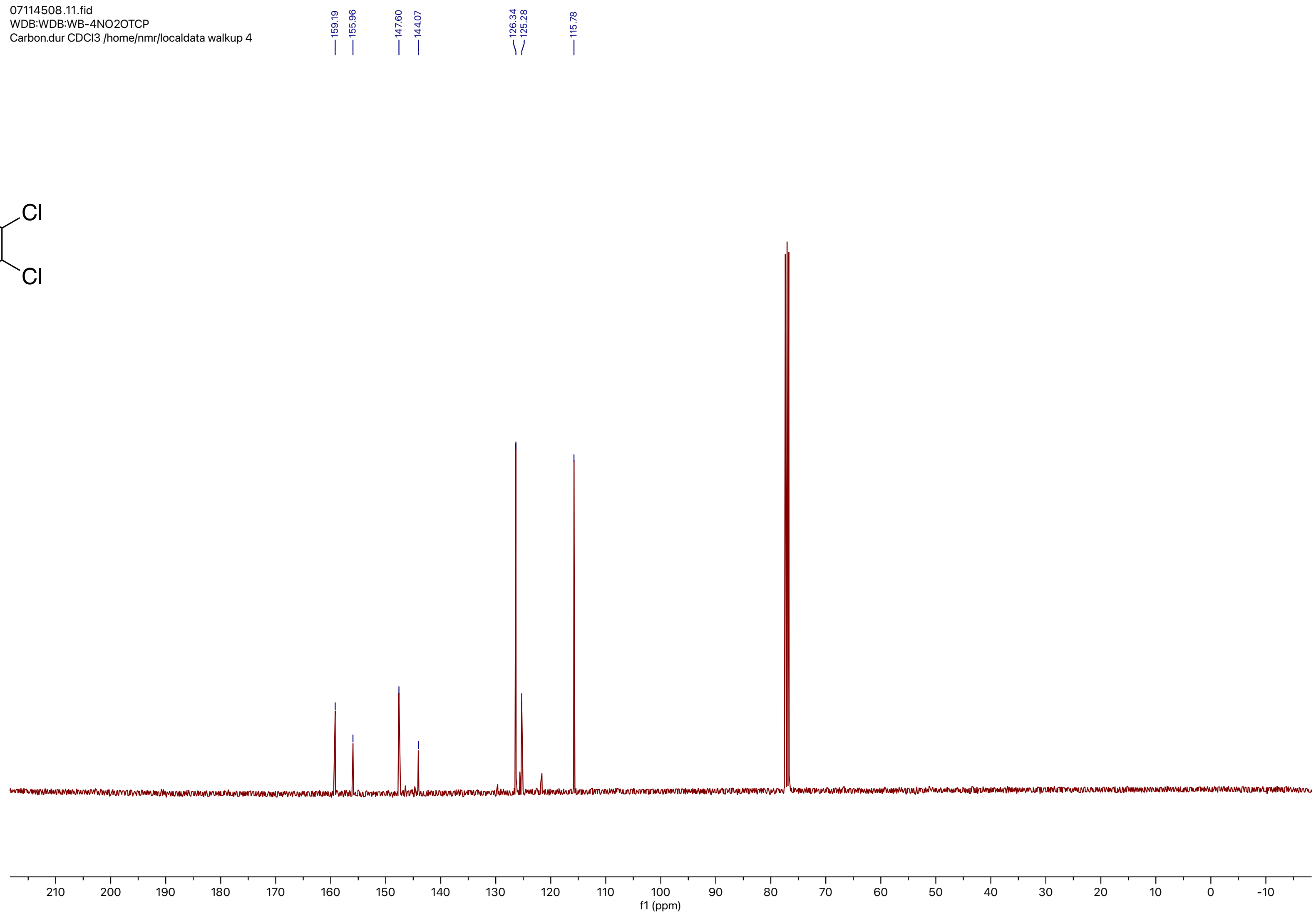

Figure S49. $^{13}C\{^1H\}$ NMR spectrum of **18** recorded at 101 MHz in $CDCl_3$.

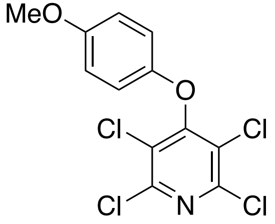

19

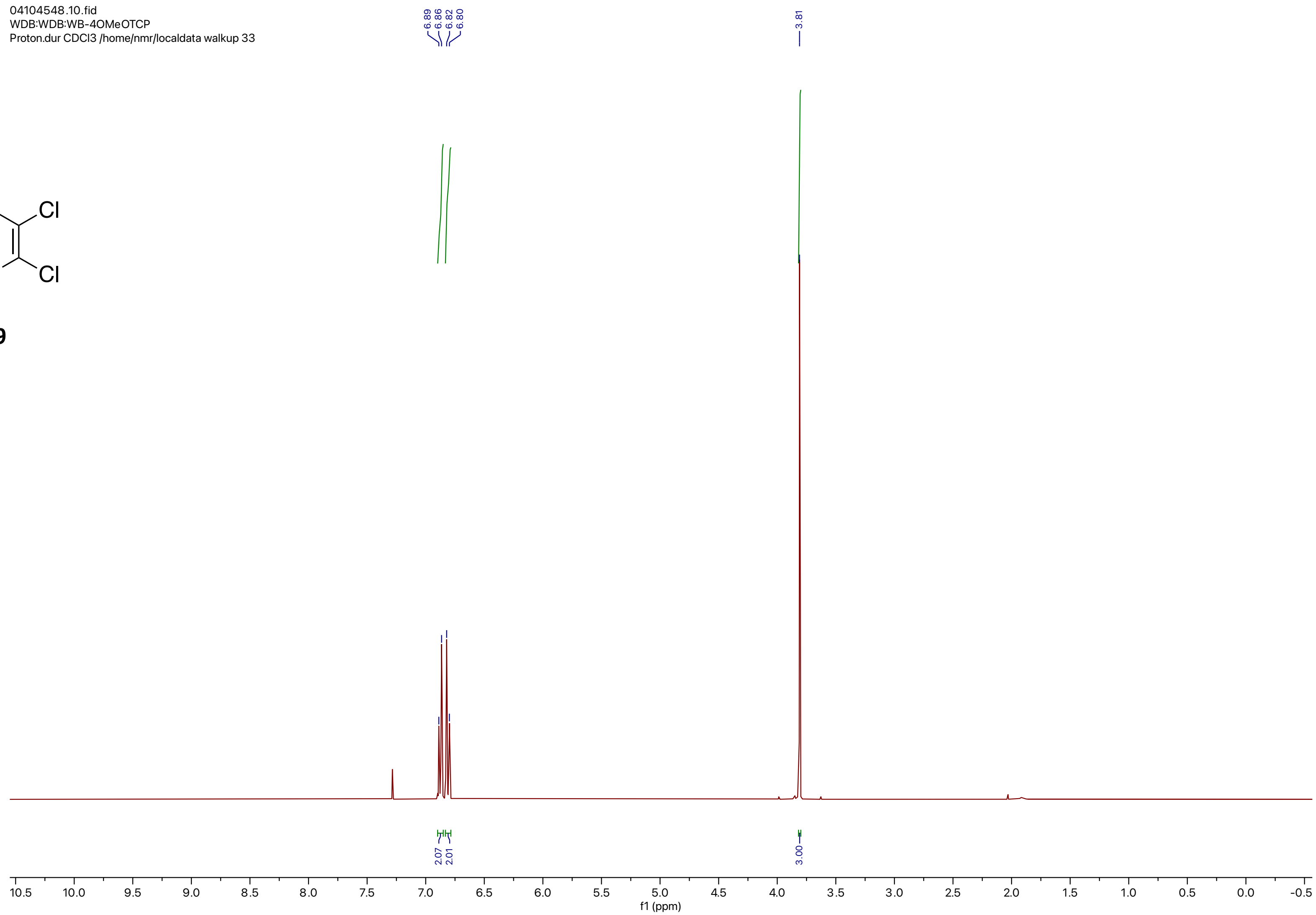

Figure S50. ^{1}H NMR spectrum of **19** recorded at 400 MHz in $CDCl_3$.

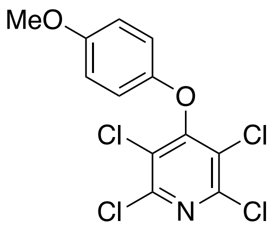

19

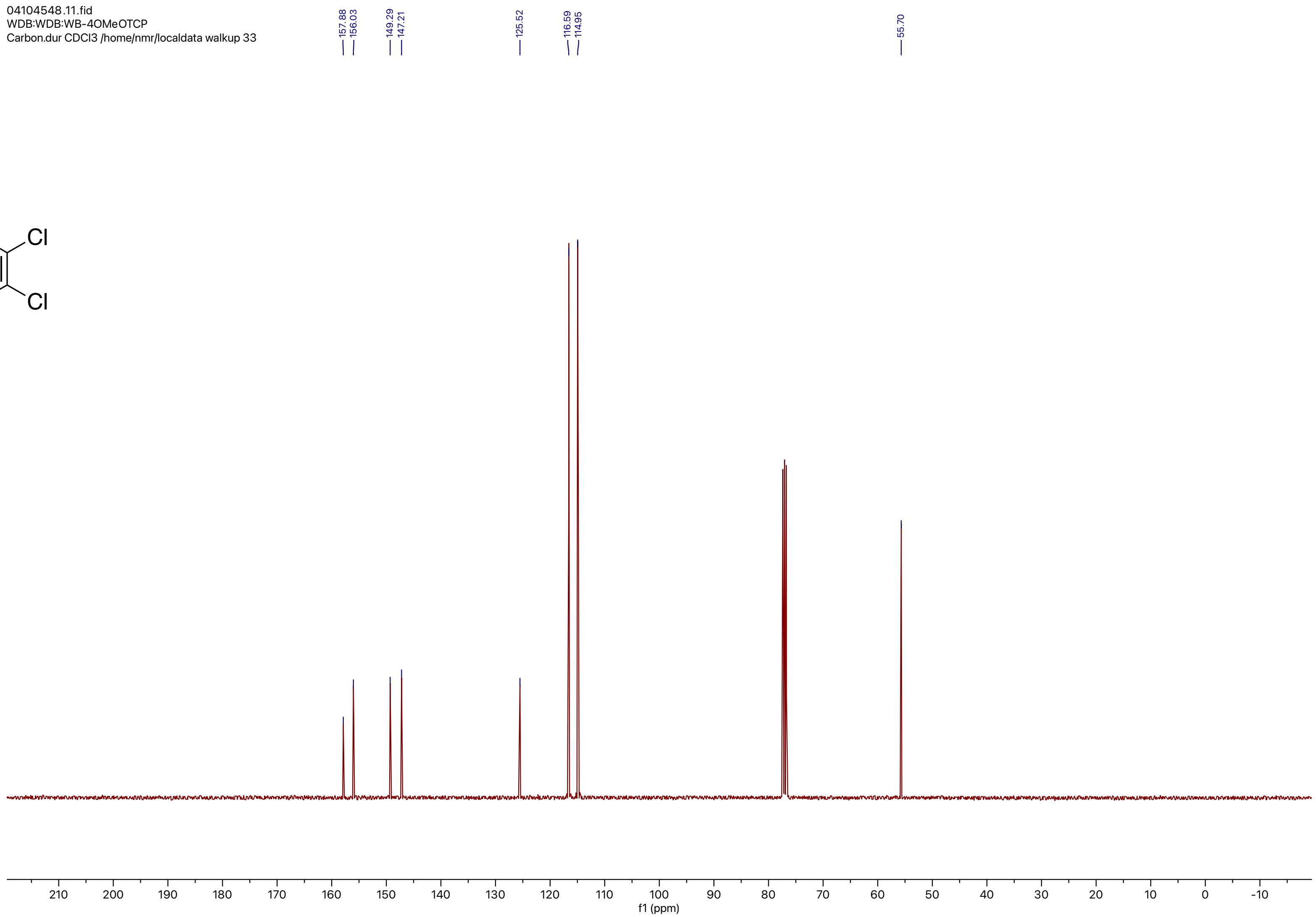

Figure S51. $^{13}C\{^{1}H\}$ NMR spectrum of **19** recorded at 101 MHz in $CDCl_3$.

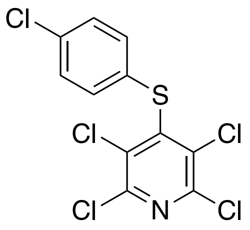

20

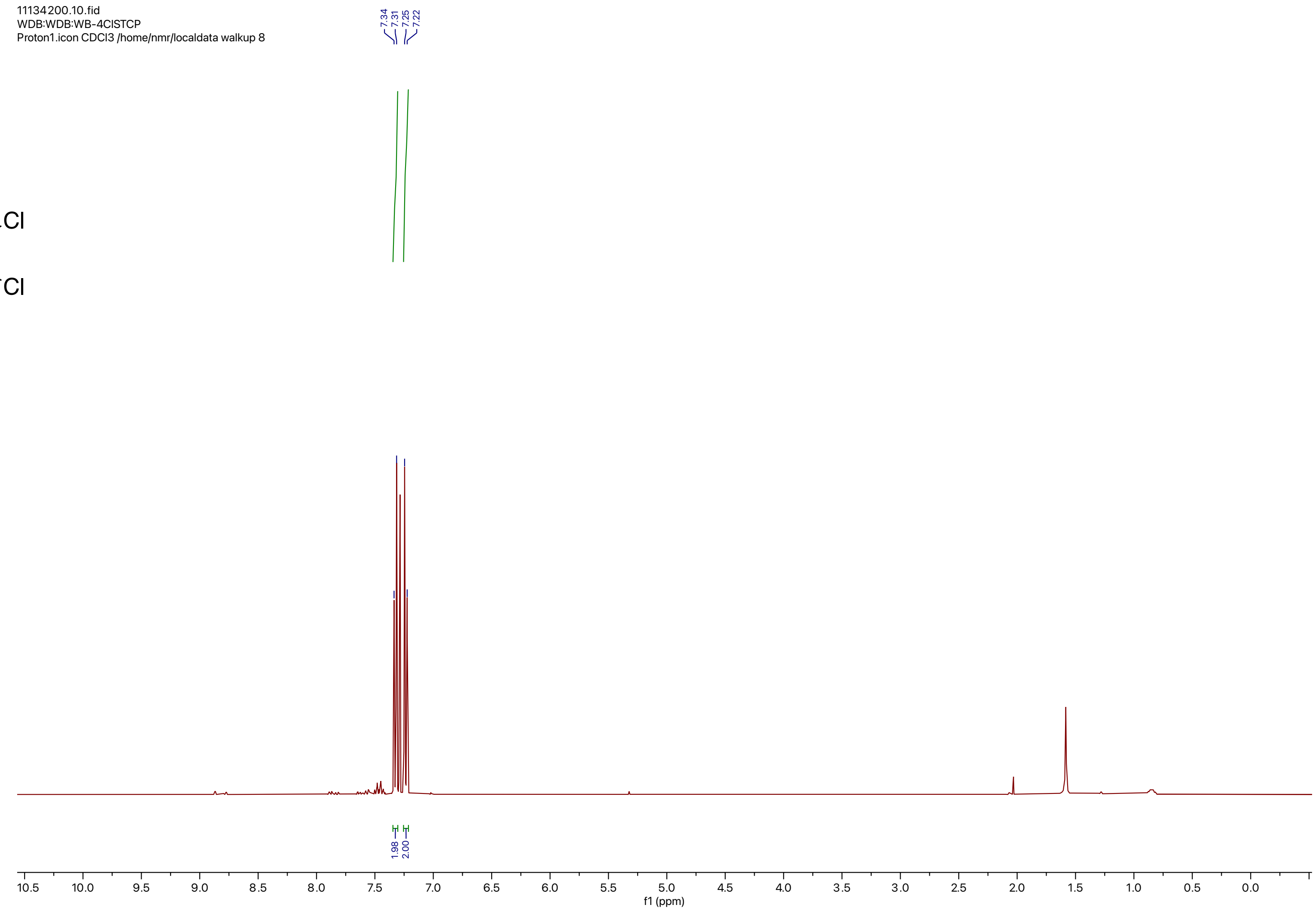

Figure S52. ^{1}H NMR spectrum of **20** recorded at 400 MHz in $CDCl_3$.

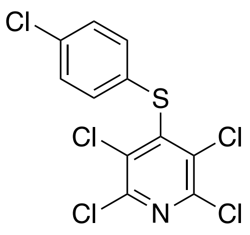

20

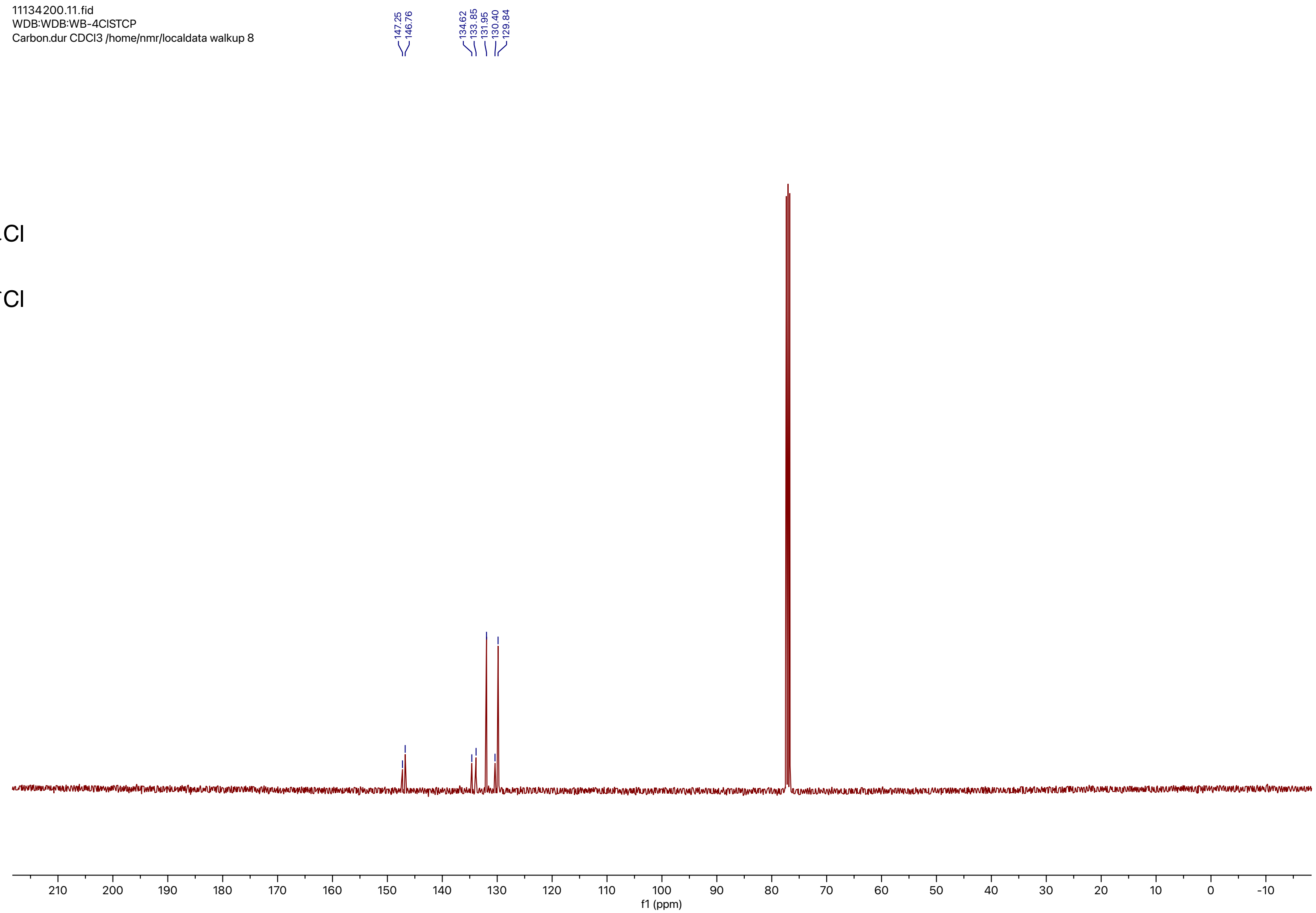

Figure S53. $^{13}C\{^{1}H\}$ NMR spectrum of **20** recorded at 101 MHz in $CDCl_3$.

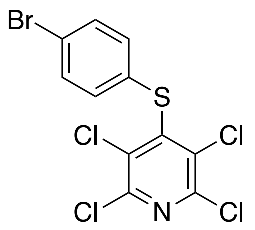

21

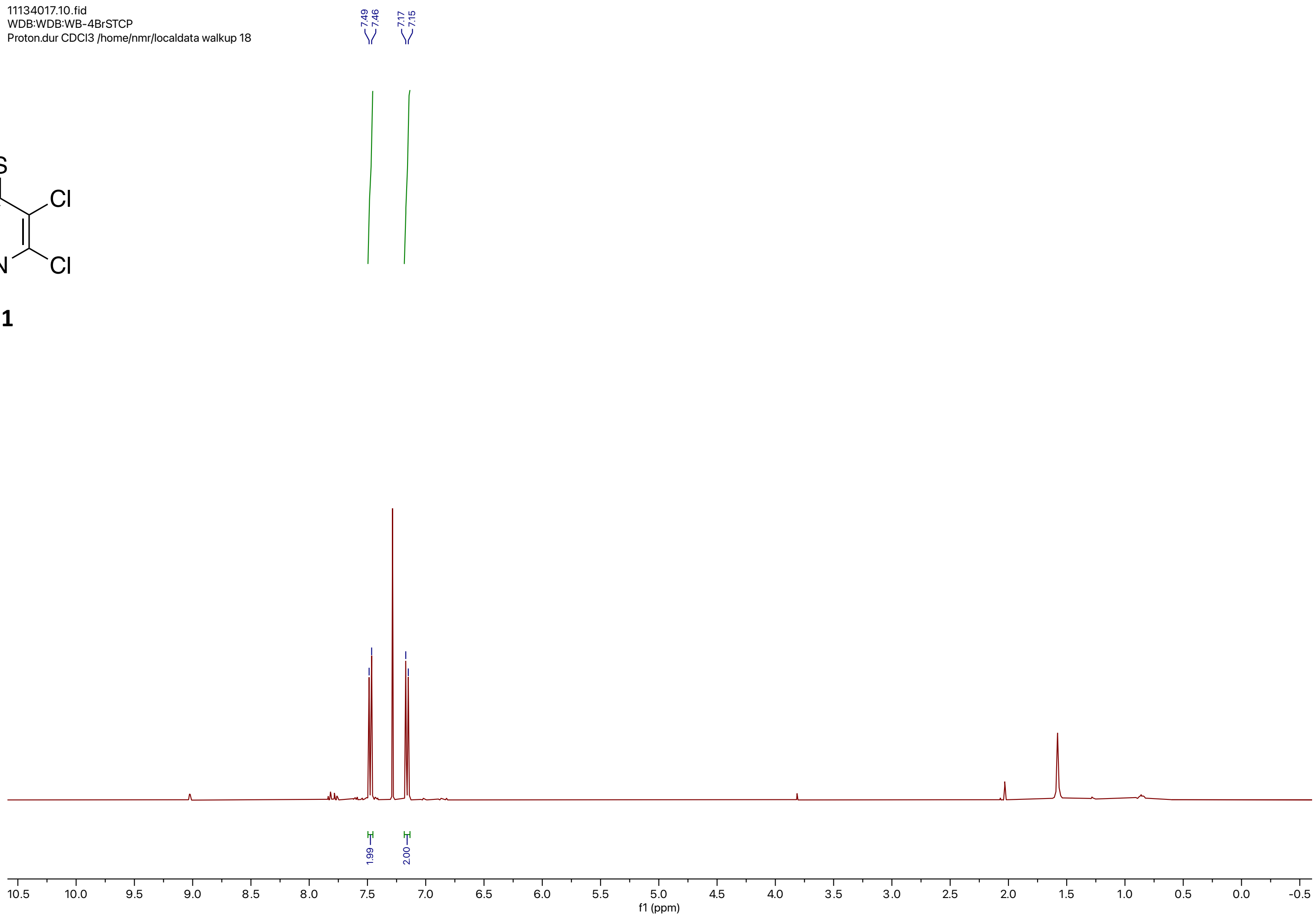

Figure S54. ^{1}H NMR spectrum of **21** recorded at 400MHz in $CDCl_3$.

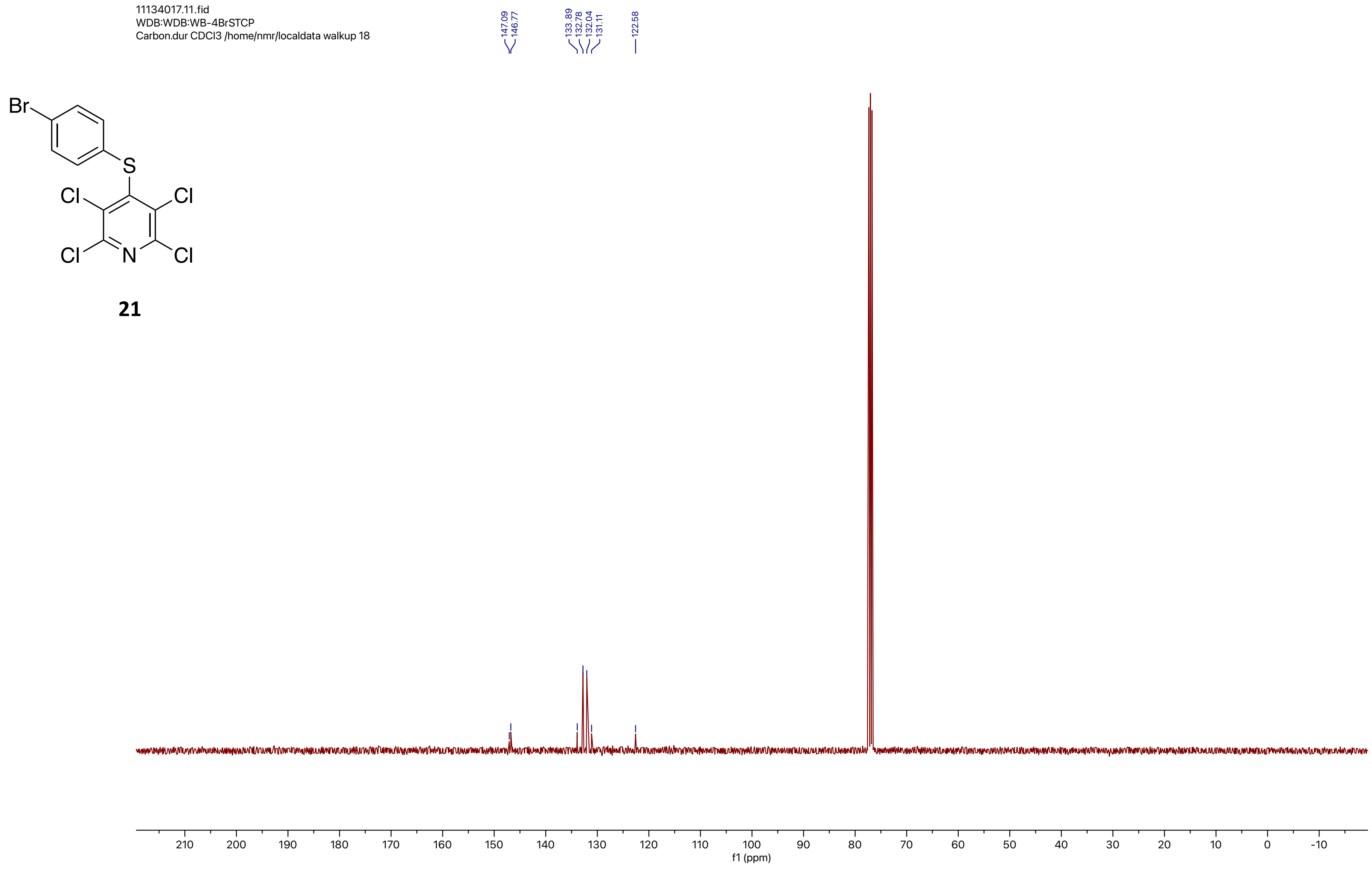

Figure S55. $^{13}C\{^{1}H\}$ NMR spectrum of **21** recorded at 101 MHz in $CDCl_3$.

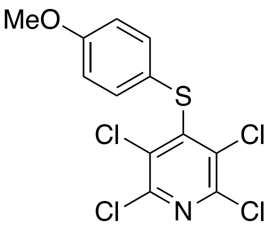

22

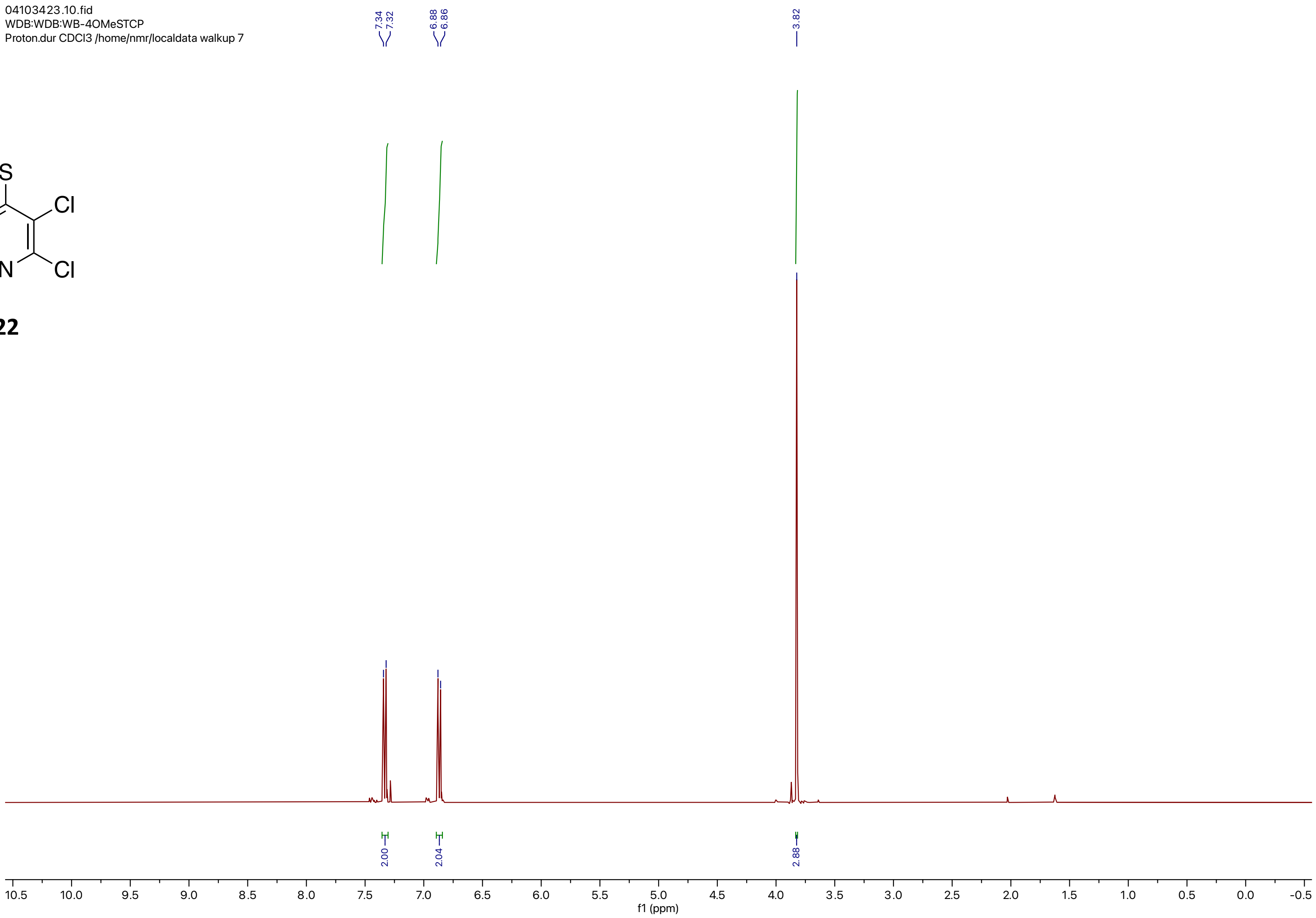

Figure S56. ^{1}H NMR spectrum of **22** recorded at 400 MHz in $CDCl_3$.

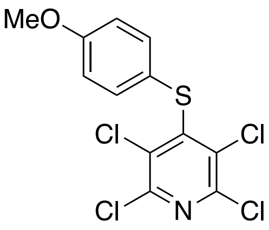

22

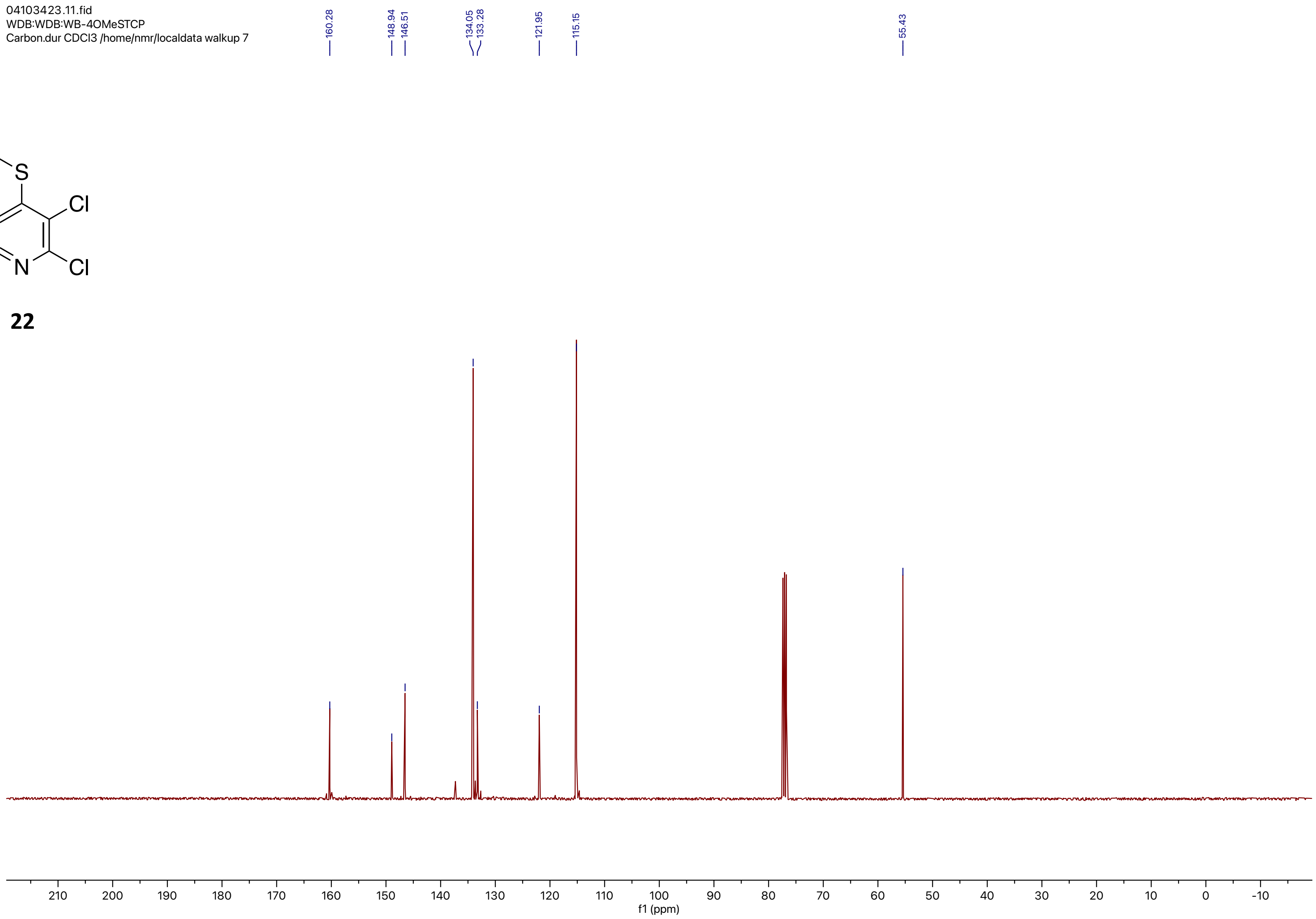

Figure S57. $^{13}C\{^{1}H\}$ NMR spectrum of **22** recorded at 101 MHz in $CDCl_3$.

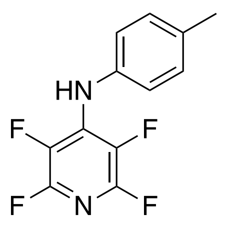

23

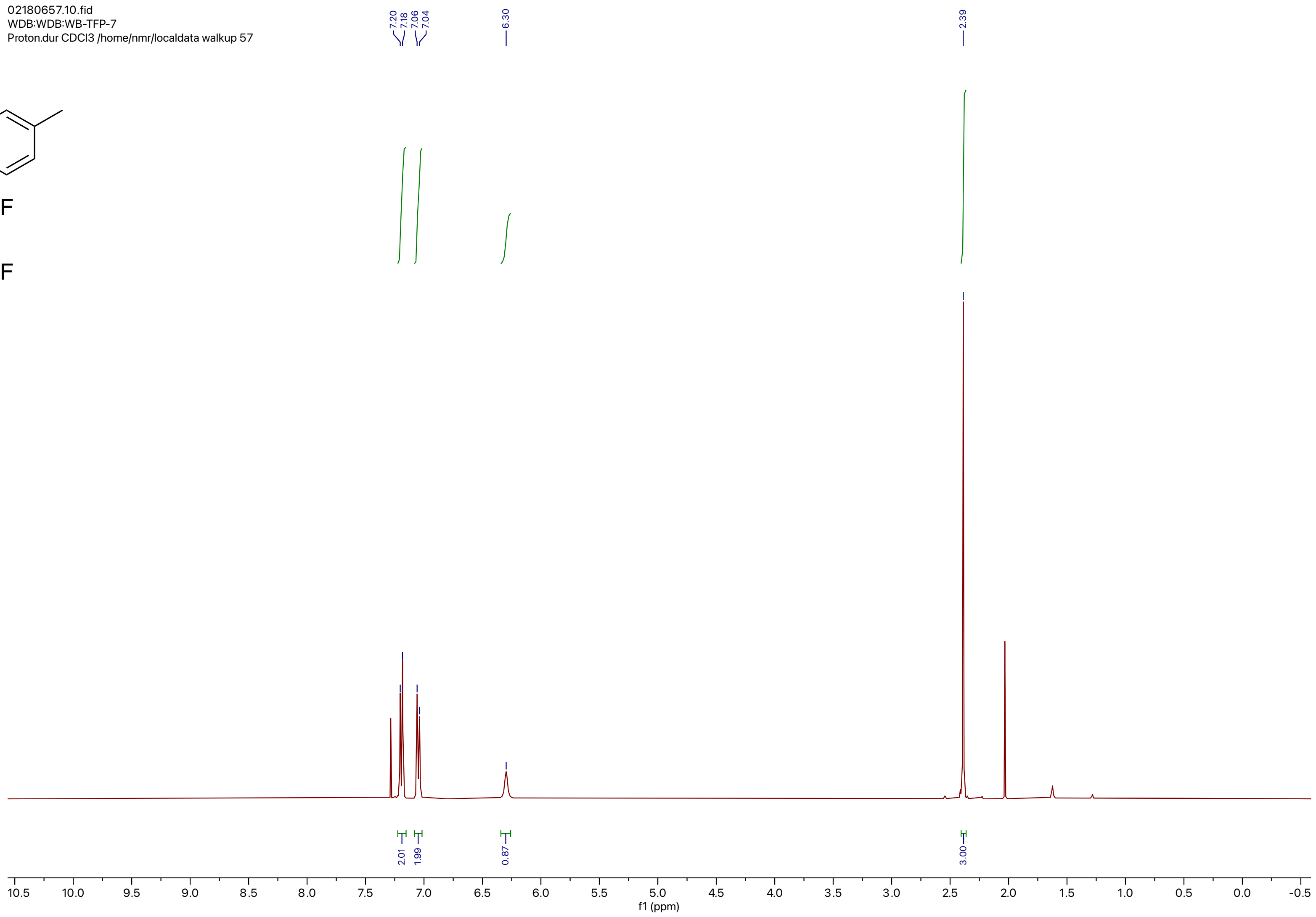

Figure S58. ^{1}H NMR spectrum of **23** recorded at 400 MHz in $CDCl_3$.

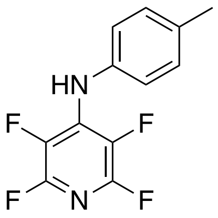

23

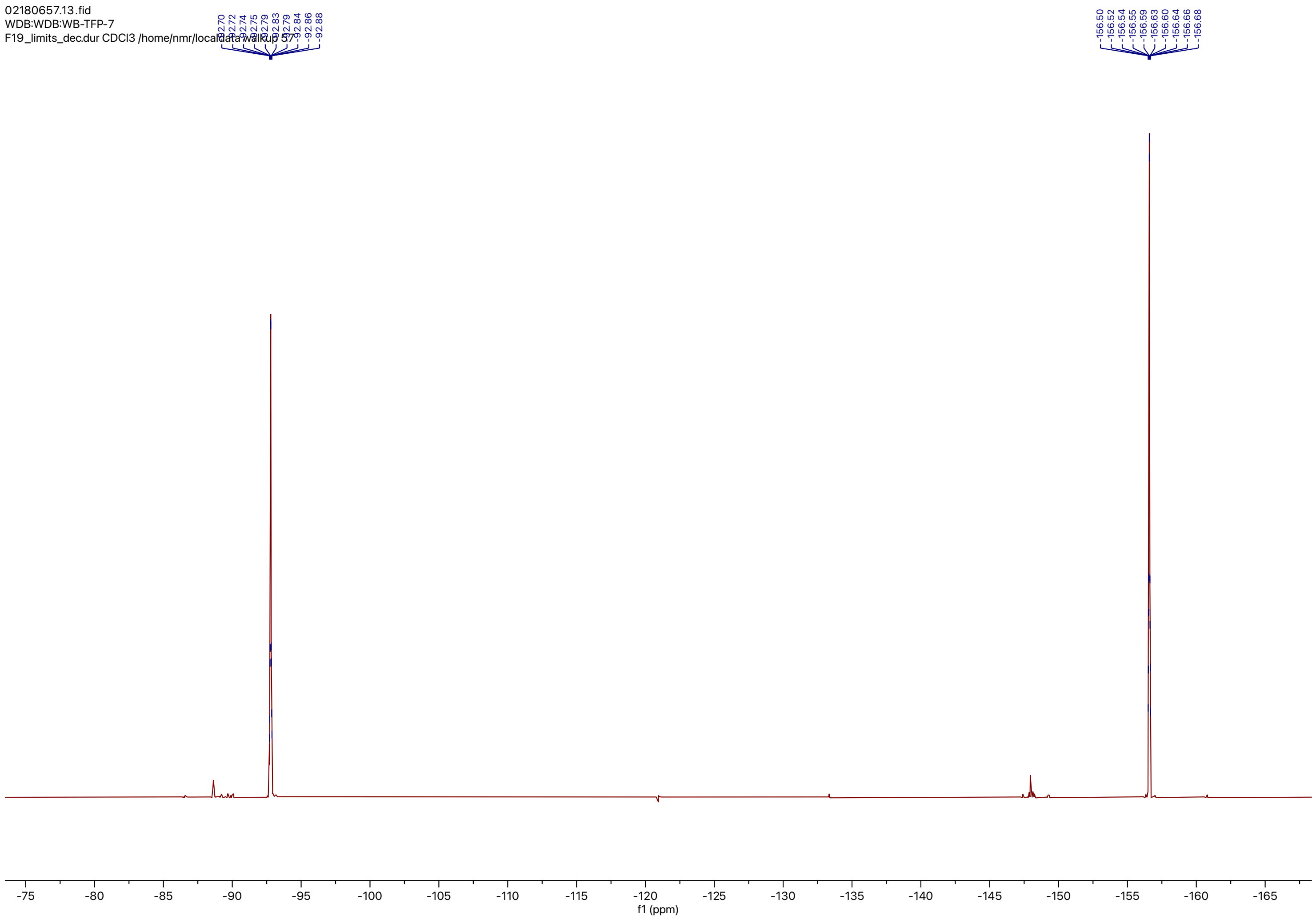

Figure S59. $^{19}F\{^1H\}$ NMR spectrum of **23** recorded at 376 MHz in $CDCl_3$.

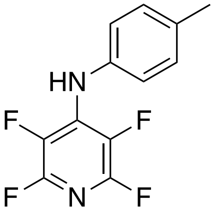

23

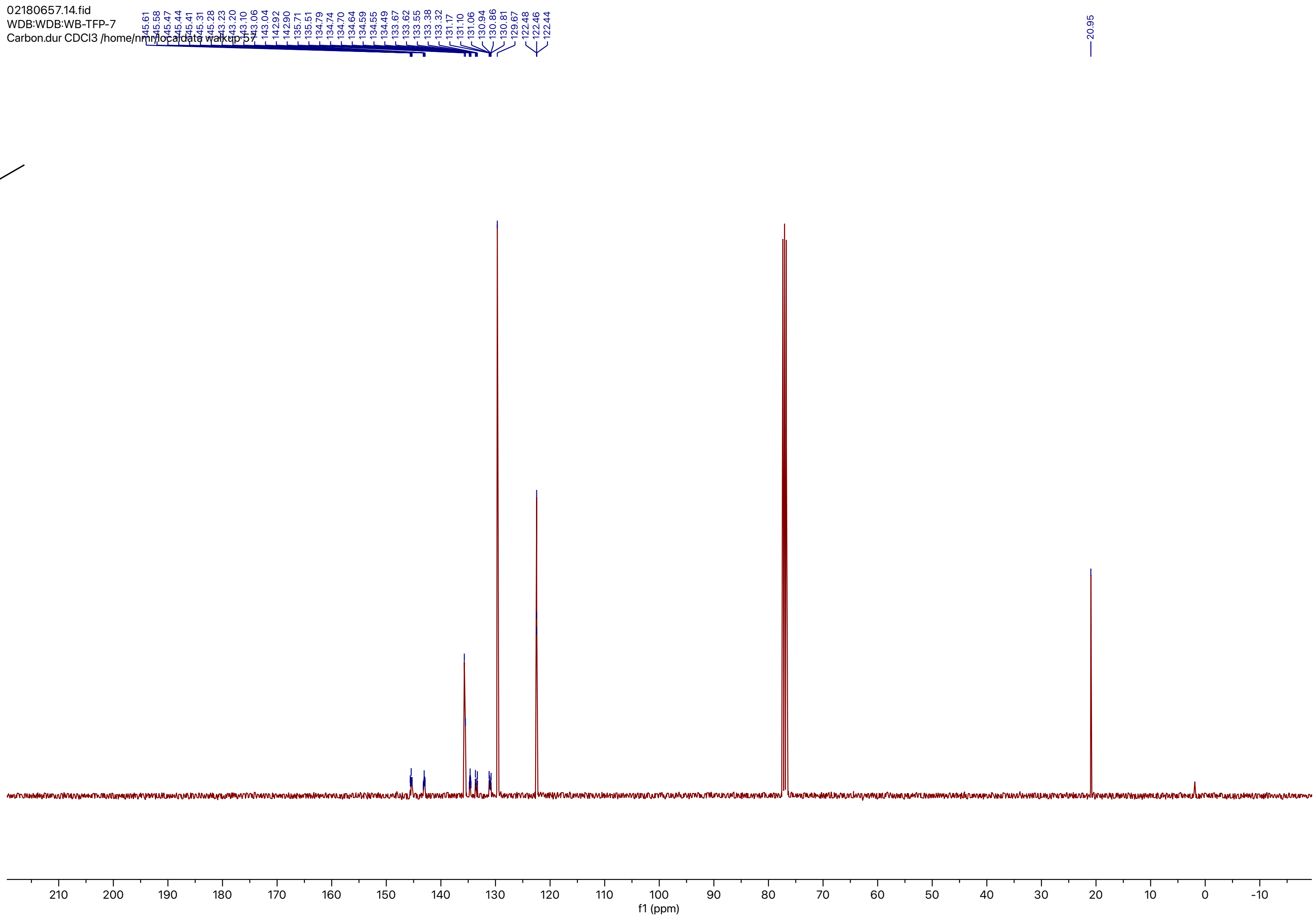

Figure S60. $^{13}C\{^{1}H\}$ NMR spectrum of **23** recorded at 101 MHz in $CDCl_3$.

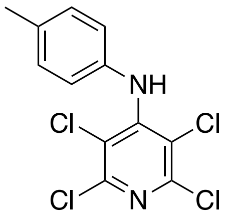

24

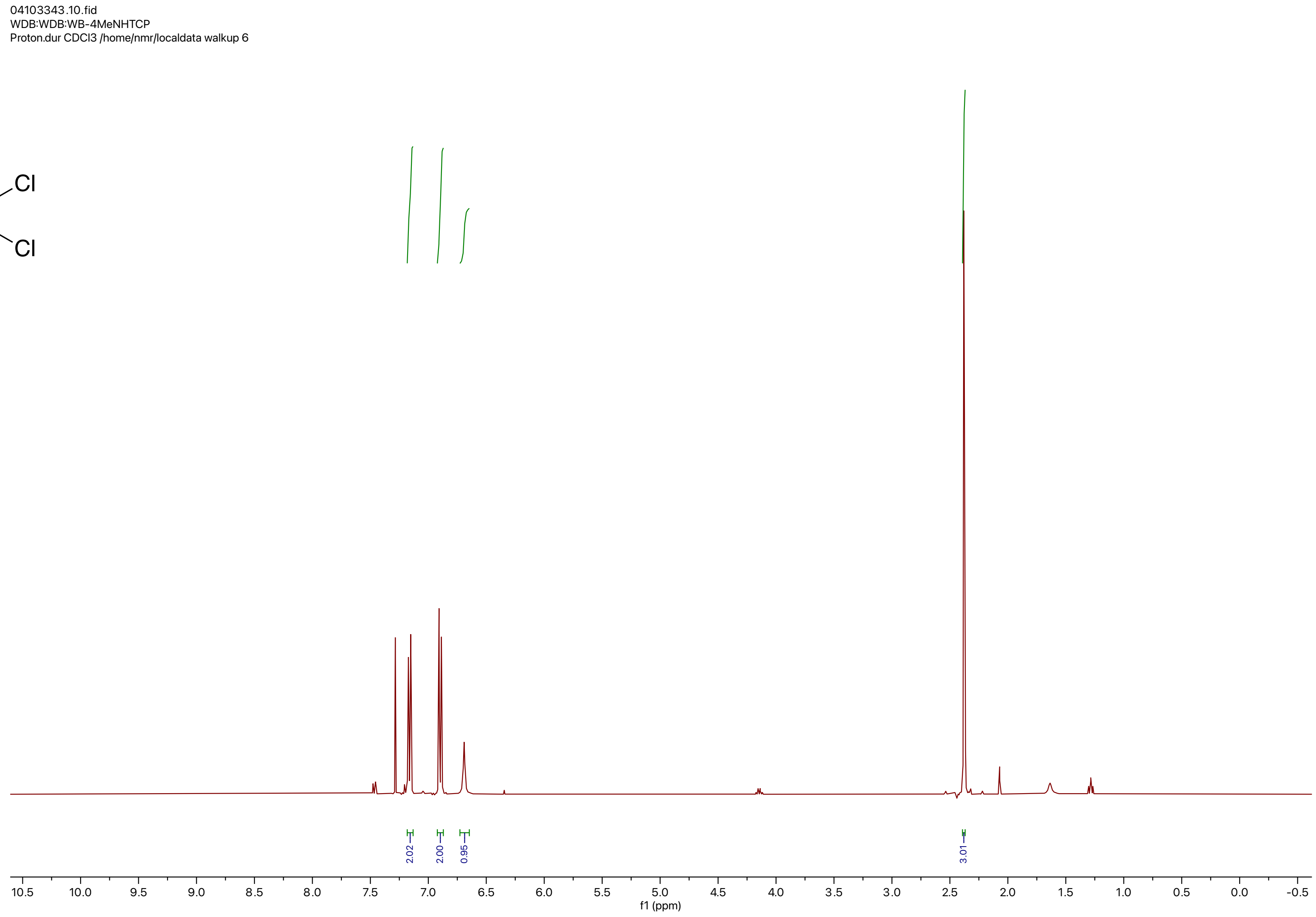

Figure S61. ^{1}H NMR spectrum of **24** recorded at 400 MHz in $CDCl_3$.

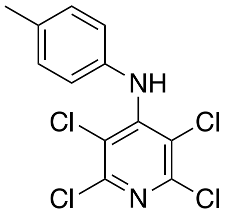

24

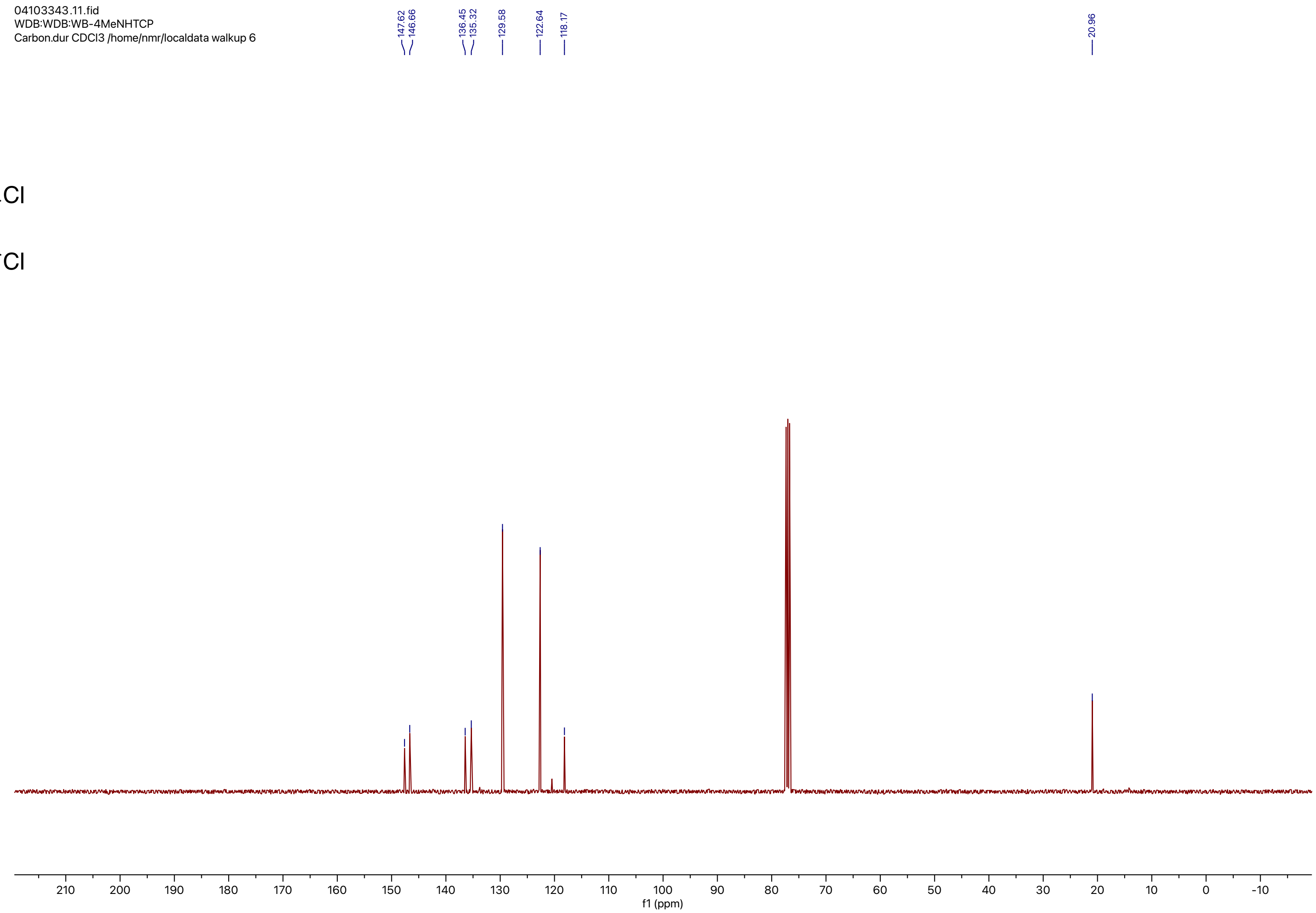

Figure S62. $^{13}C\{^{1}H\}$ NMR spectrum of **24** recorded at 101 MHz in $CDCl_3$.

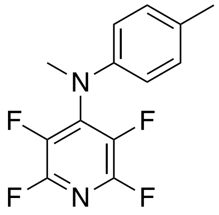

25

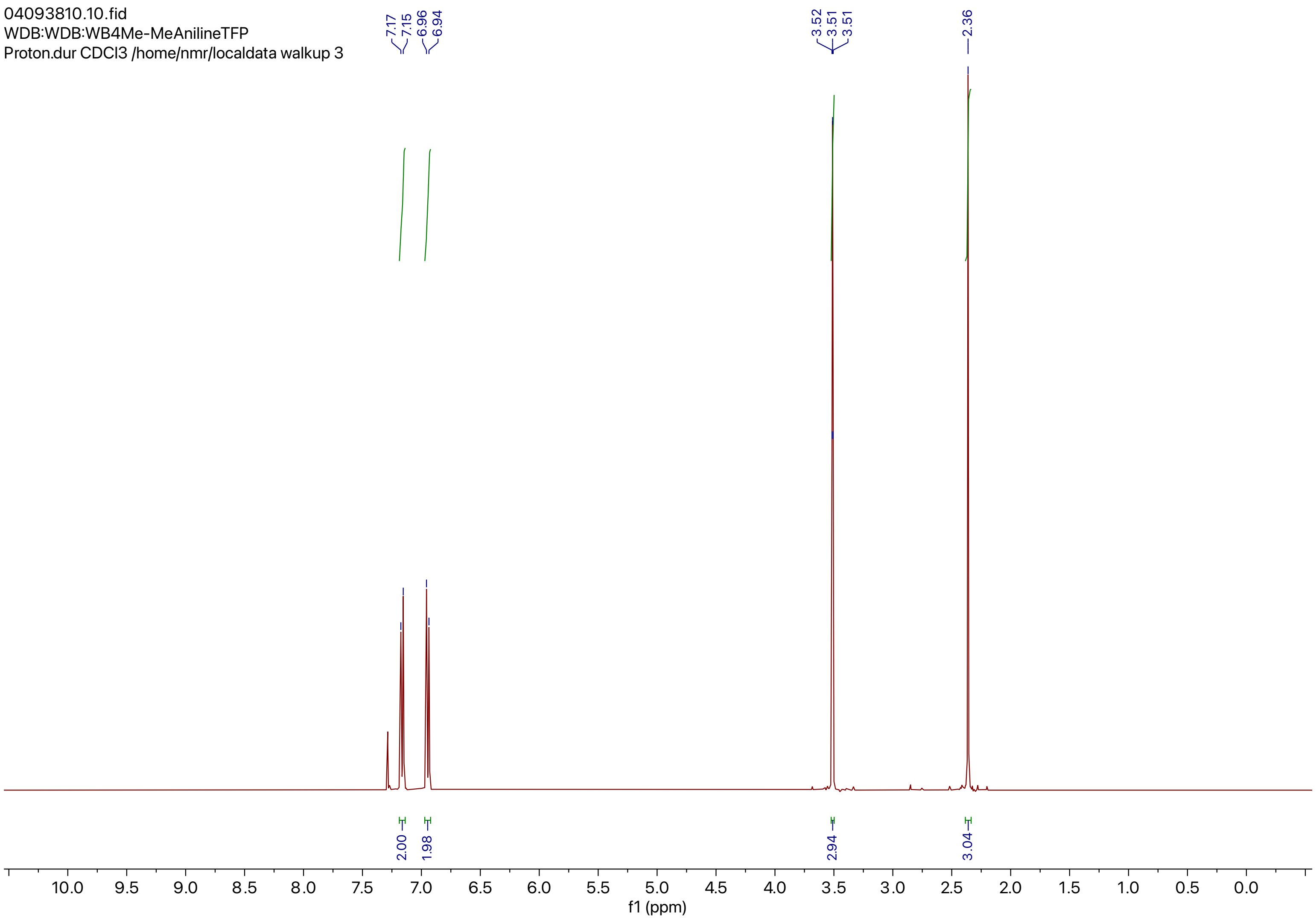

Figure S63. ^{1}H NMR spectrum of **25** recorded at 400 MHz in $CDCl_3$.

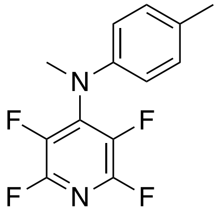

25

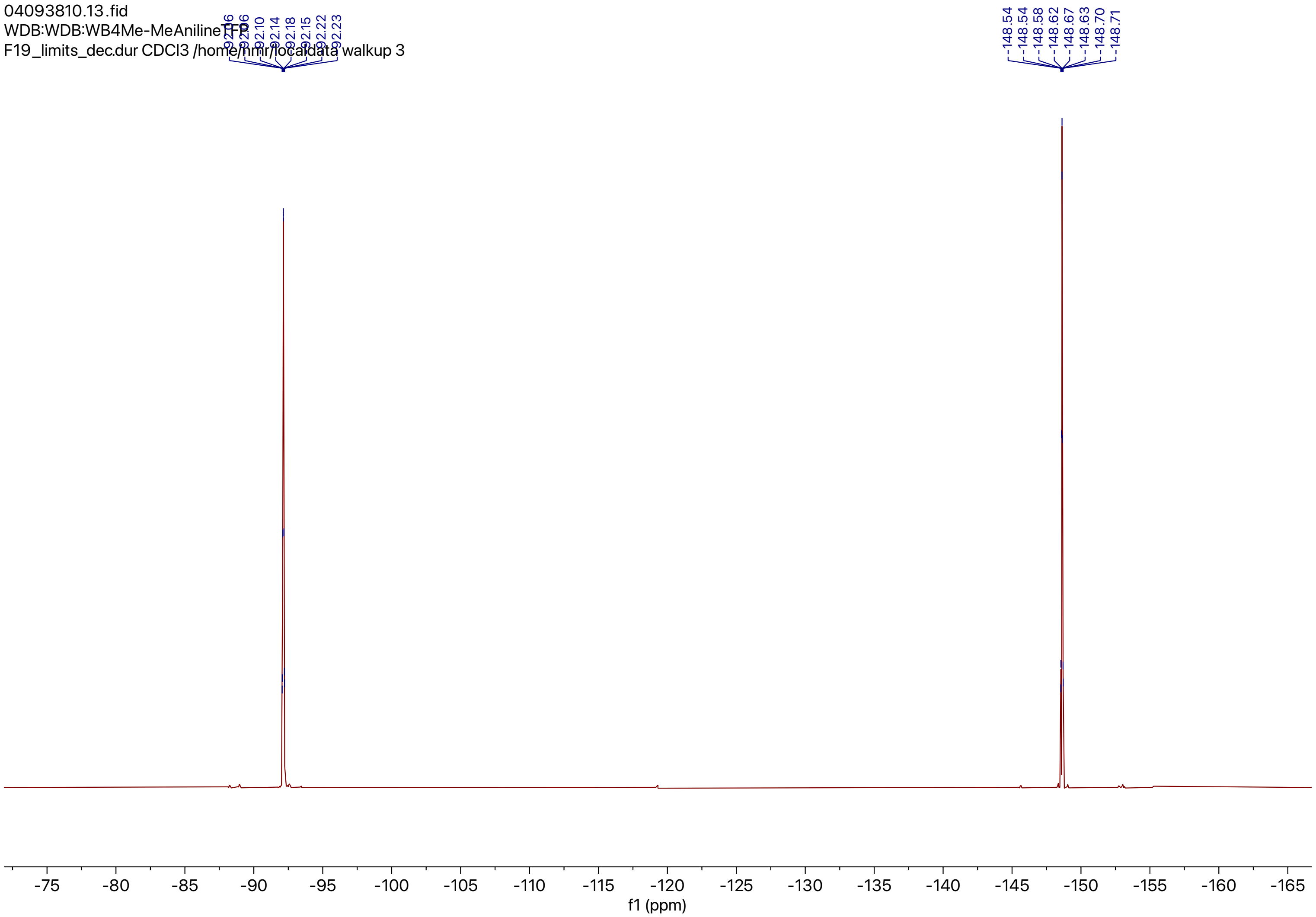

Figure S64. $^{19}F\{^{1}H\}$ NMR spectrum of **25** recorded at 376 MHz in $CDCl_3$.

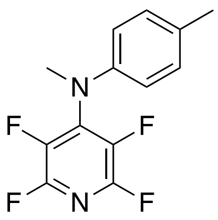

25

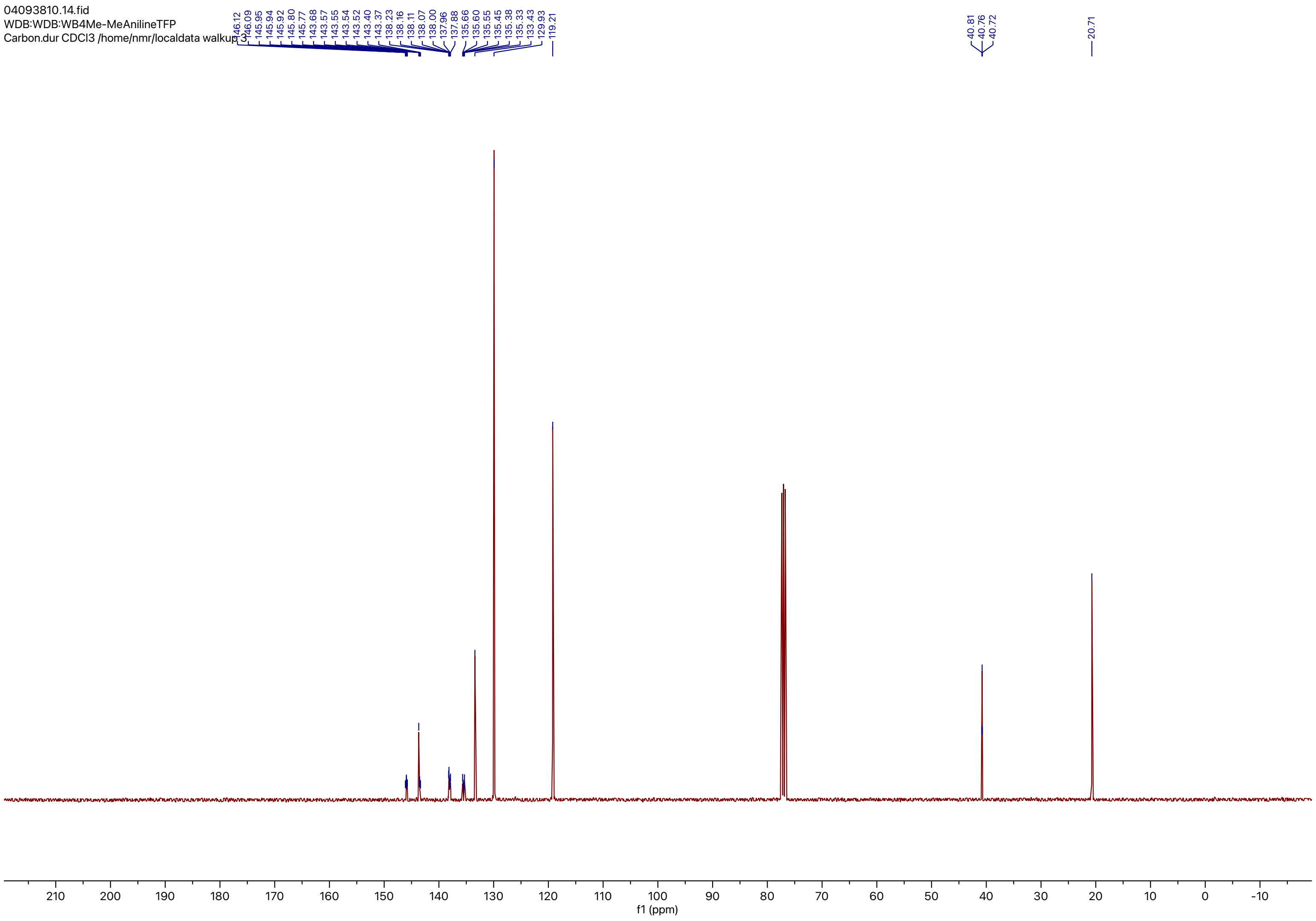

Figure S65. $^{13}C\{^{1}H\}$ NMR spectrum of **25** recorded at 101 MHz in $CDCl_3$.

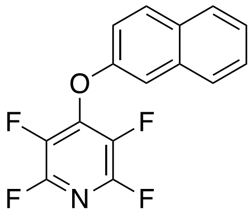

26

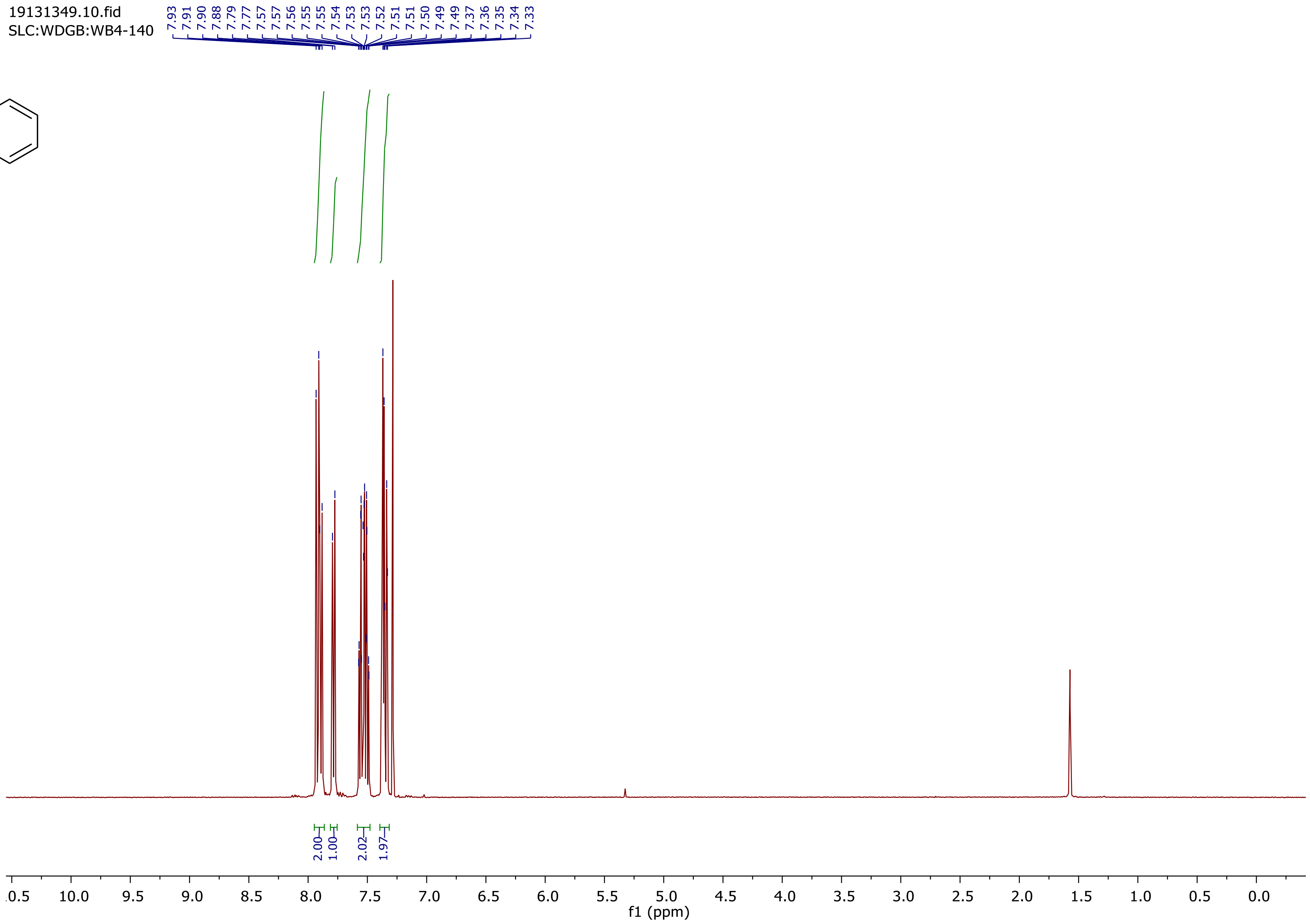

Figure S66. ^{1}H NMR spectrum of **26** recorded at 400 MHz in $CDCl_3$.

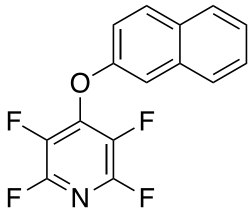

26

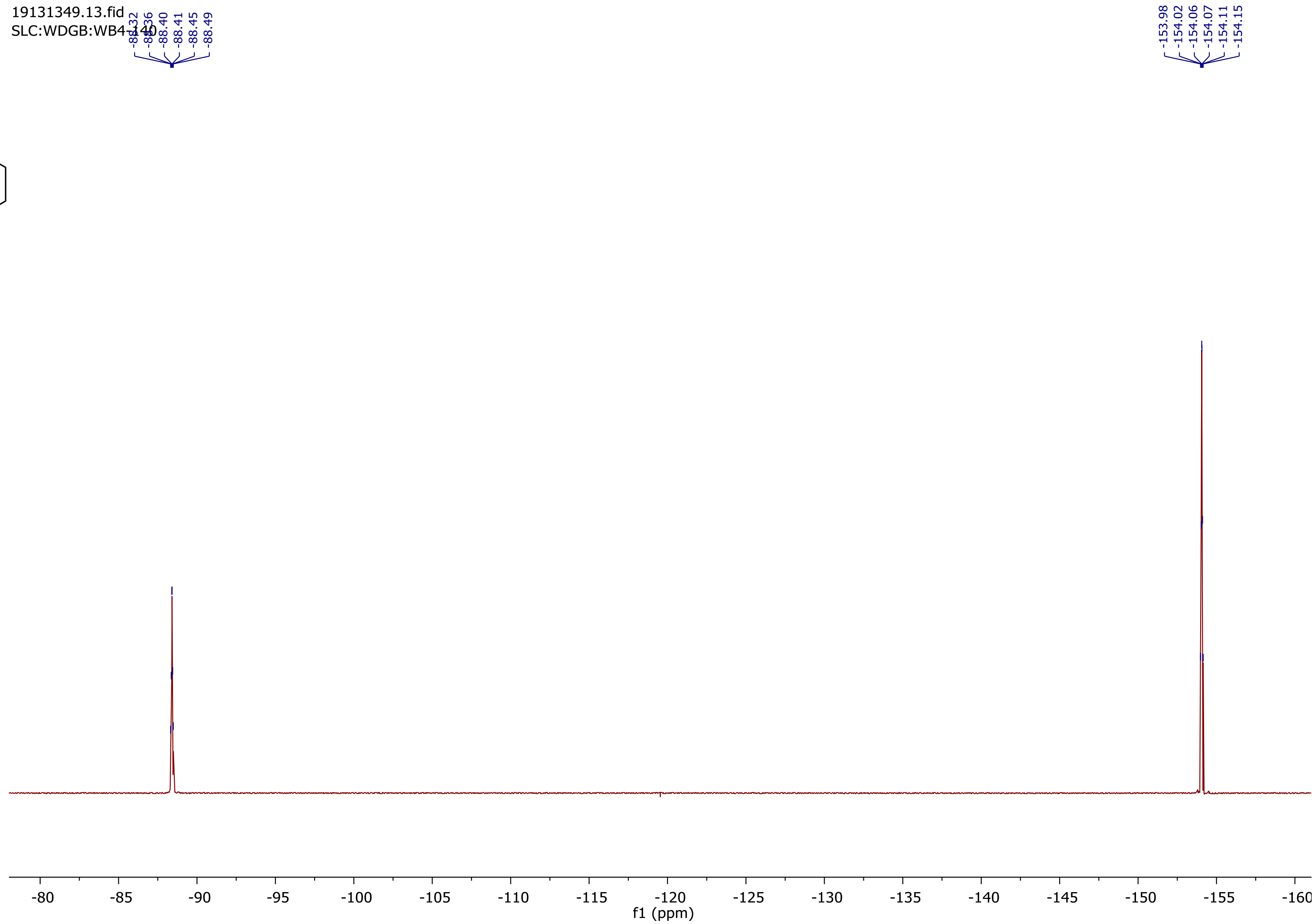

Figure S67. $^{19}F\{^{1}H\}$ NMR spectrum of **26** recorded at 376 MHz in $CDCl_3$.

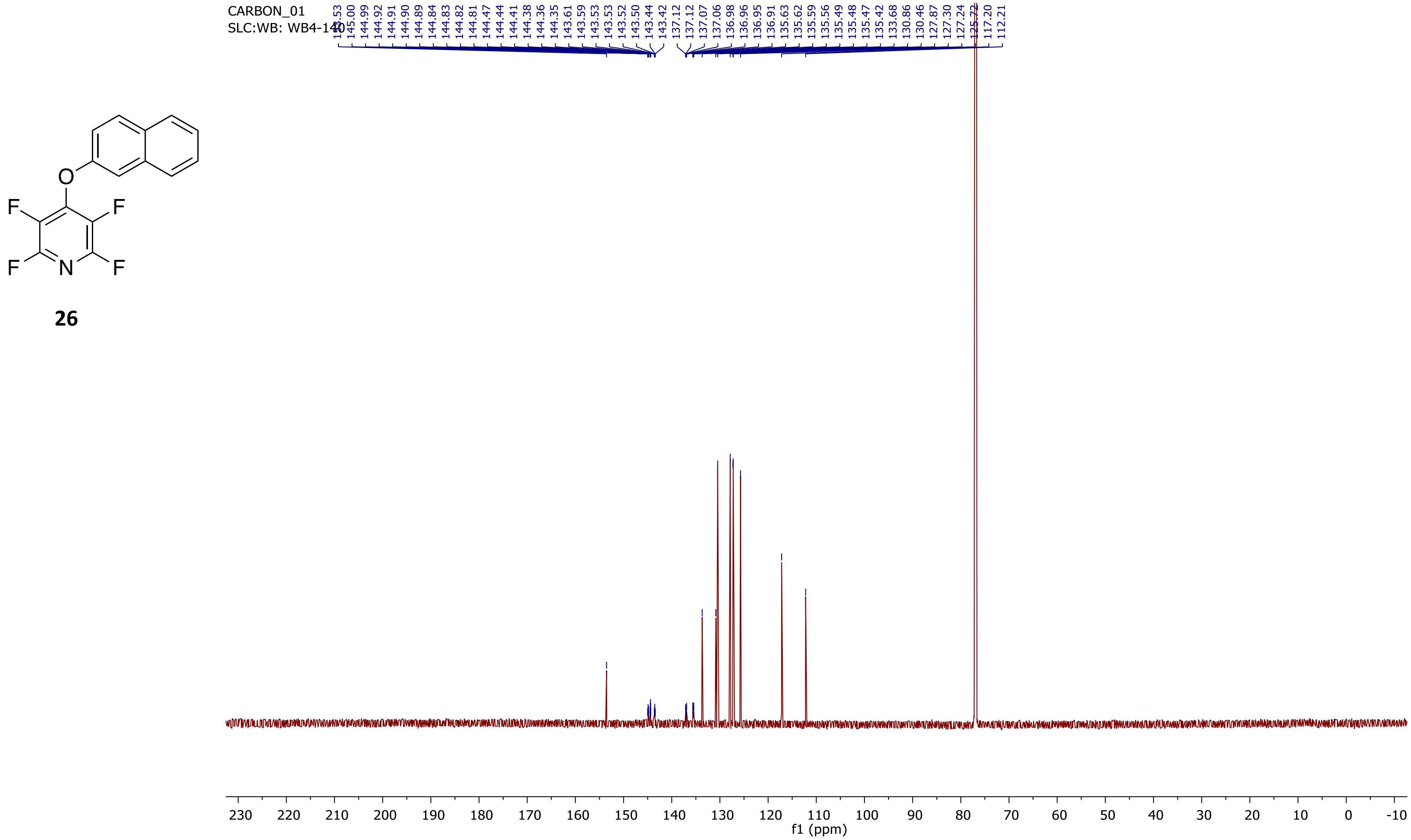

Figure S68. $^{13}C\{^1H\}$ NMR spectrum of **26** recorded at 101 MHz in $CDCl_3$.

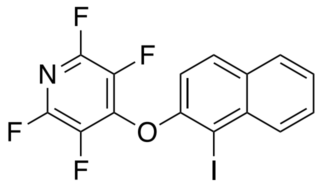

27

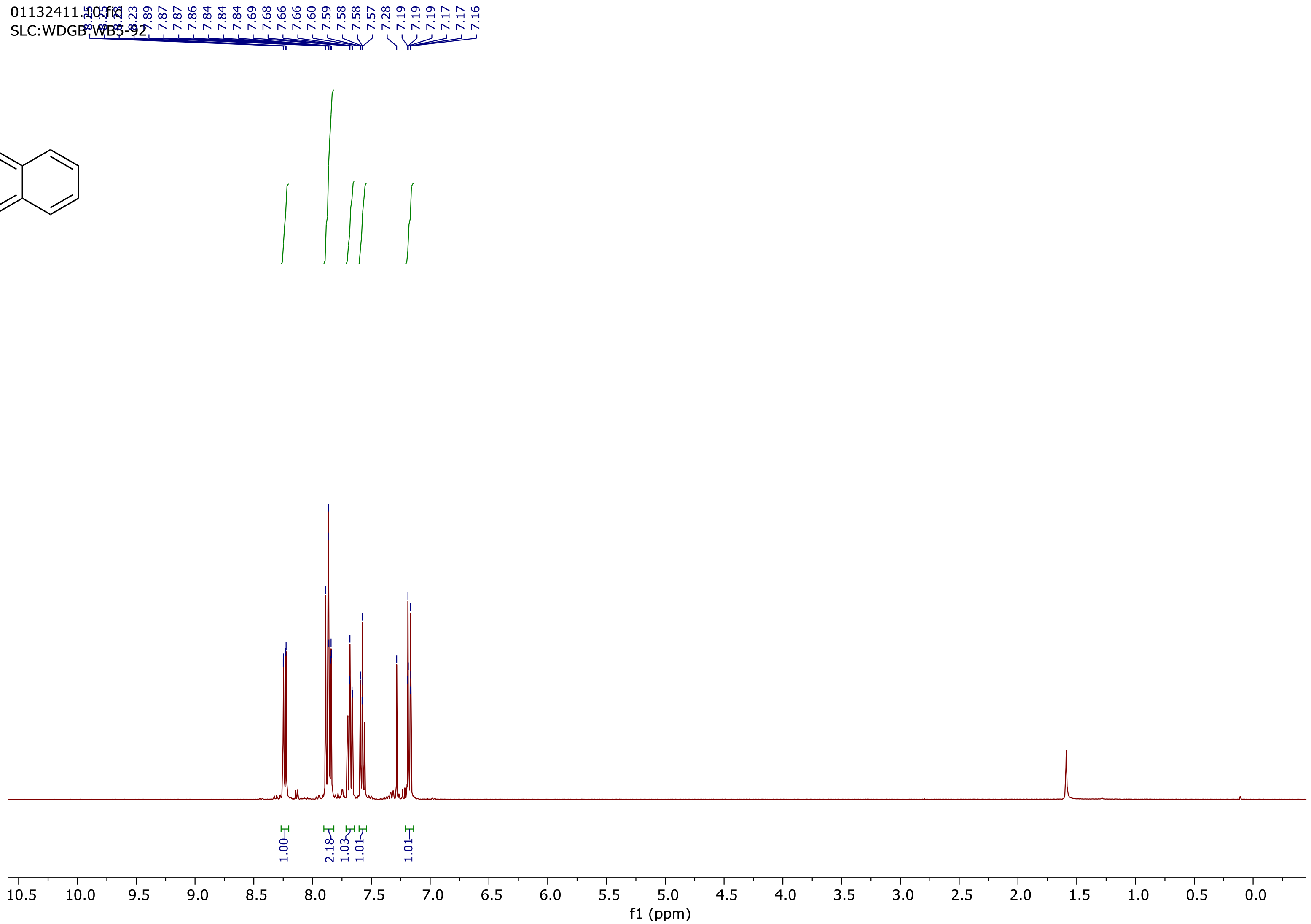

Figure S69. ^{1}H NMR spectrum of **27** recorded at 400 MHz in $CDCl_3$.

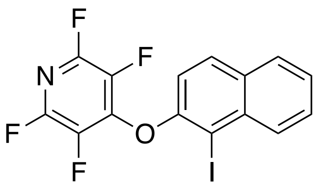

27

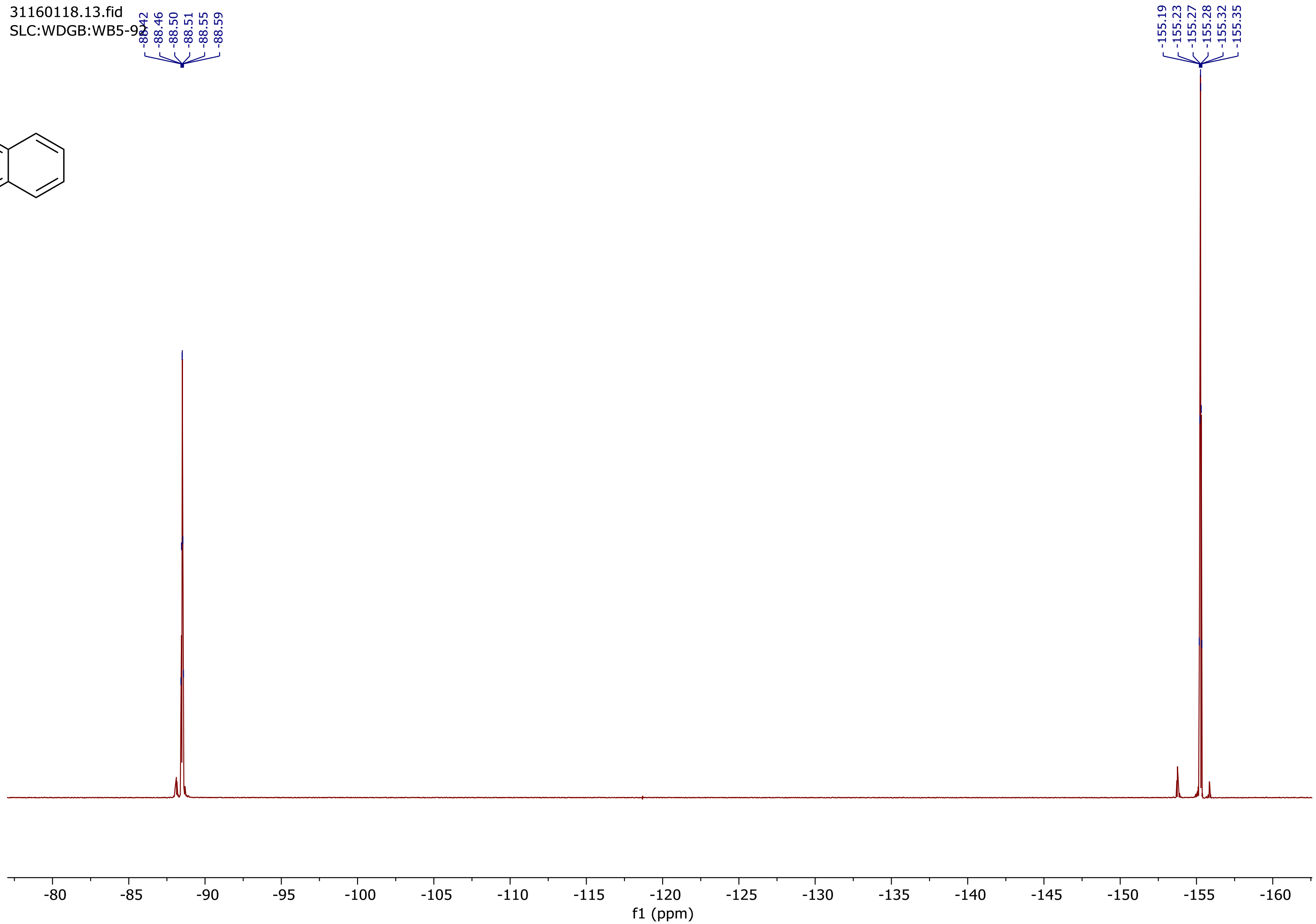

Figure S70. $^{19}F\{^{1}H\}$ NMR spectrum of **27** recorded at 376 MHz in $CDCl_3$.

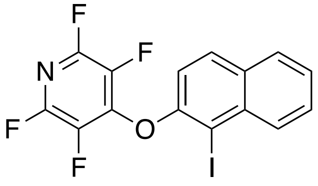

27

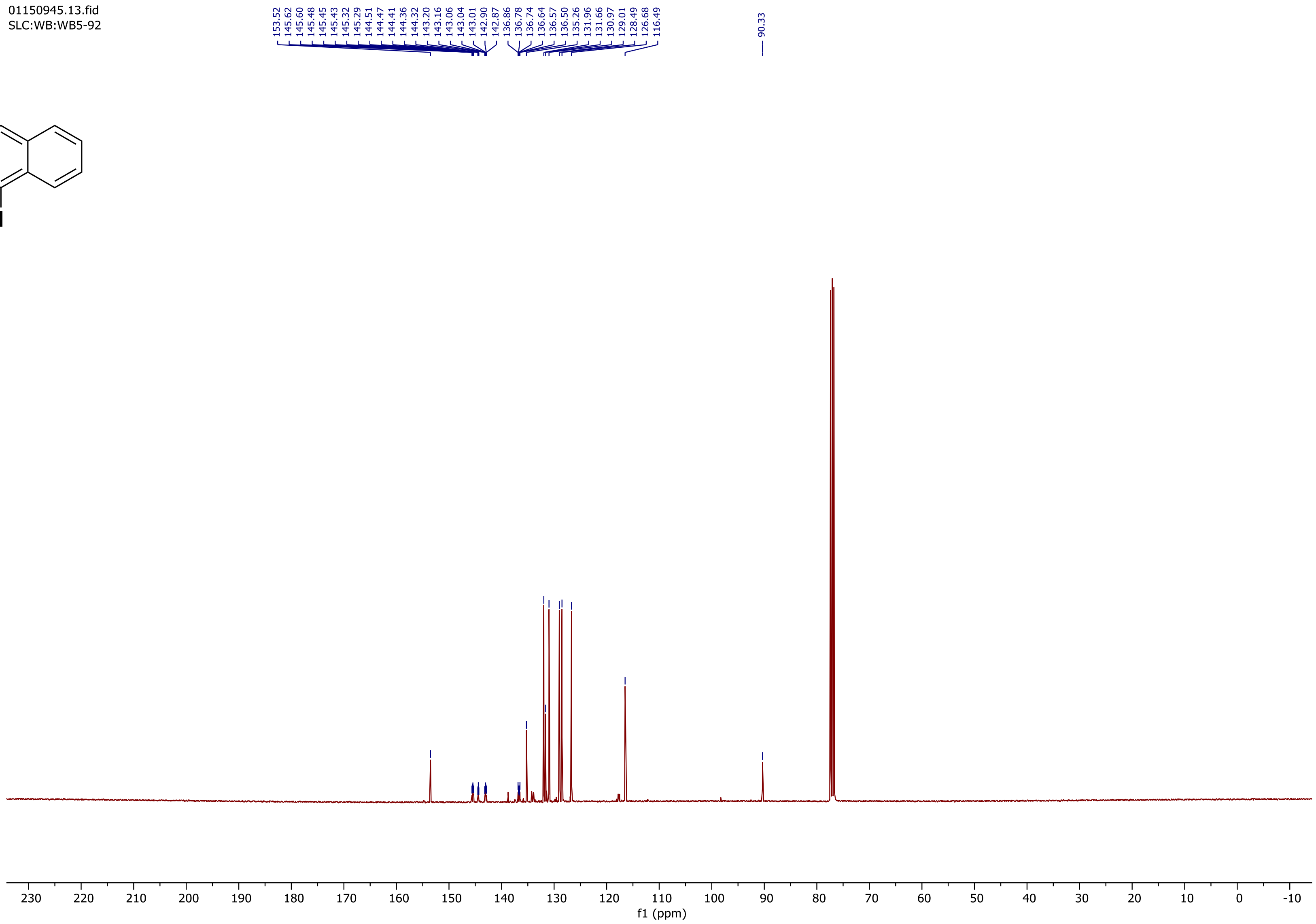

Figure S71. $^{13}C\{^{1}H\}$ NMR spectrum of **27** recorded at 101 MHz in $CDCl_3$.

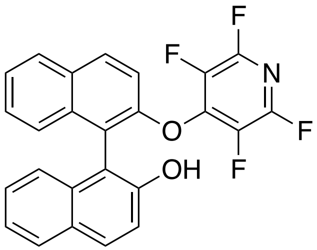

28

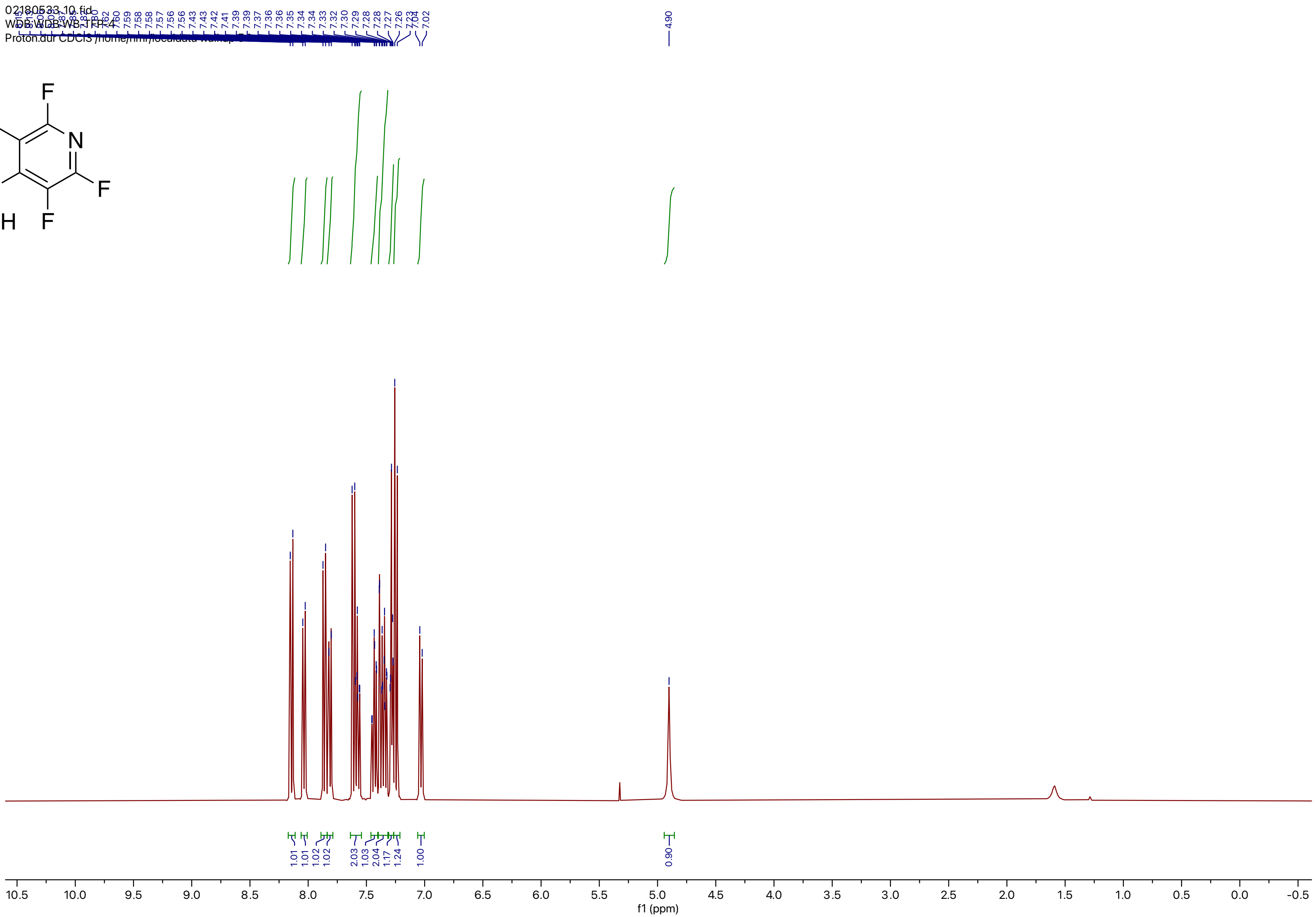

Figure S72. ^{1}H NMR spectrum of **28** recorded at 400 MHz in $CDCl_3$.

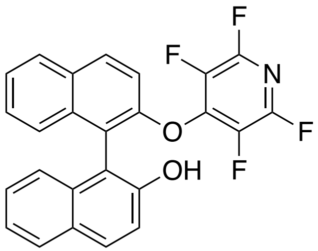

28

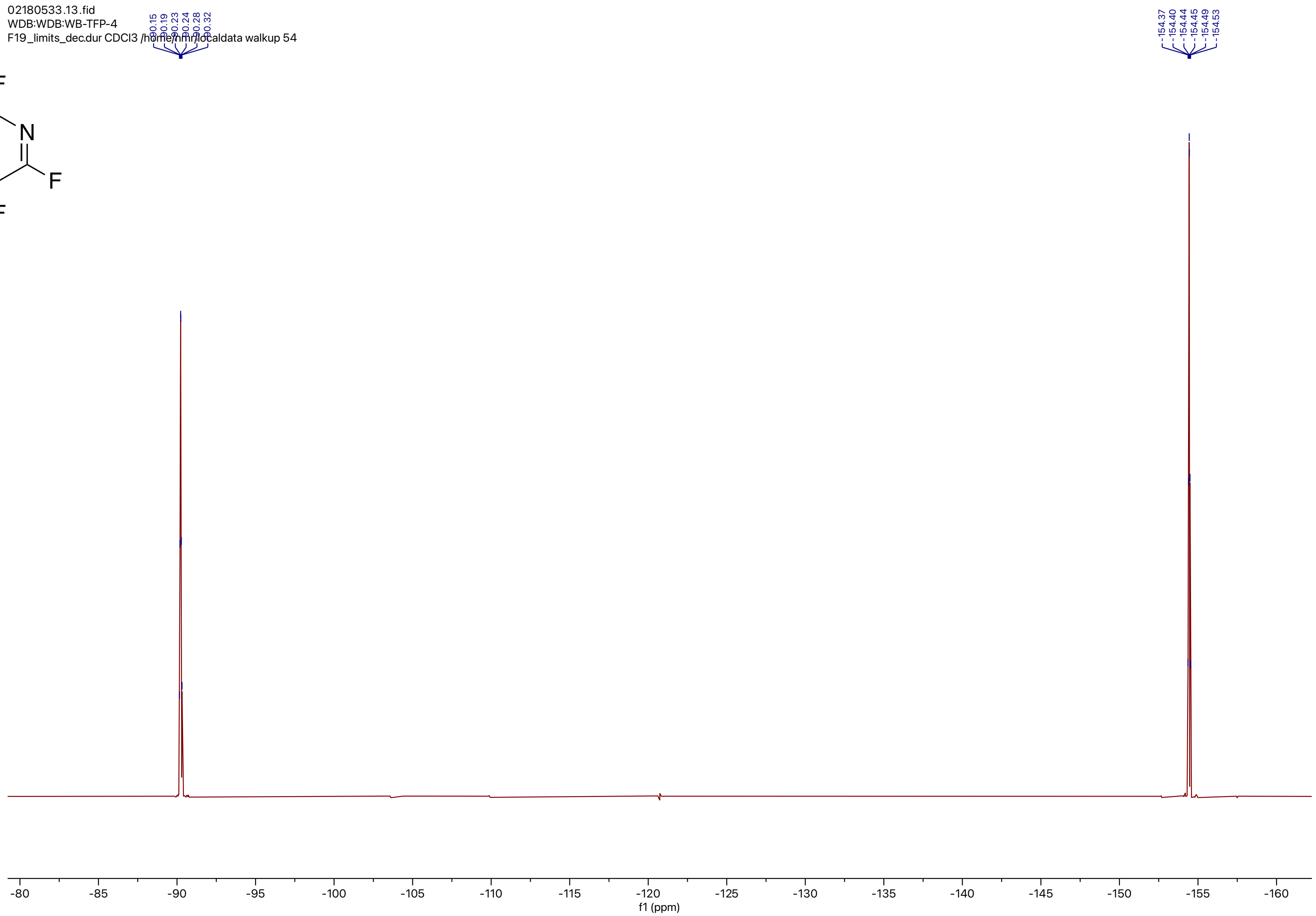

Figure S73. $^{19}F\{^{1}H\}$ NMR spectrum of **28** recorded at 376 MHz in $CDCl_3$.

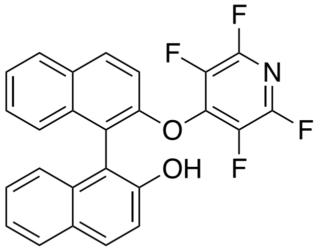

28

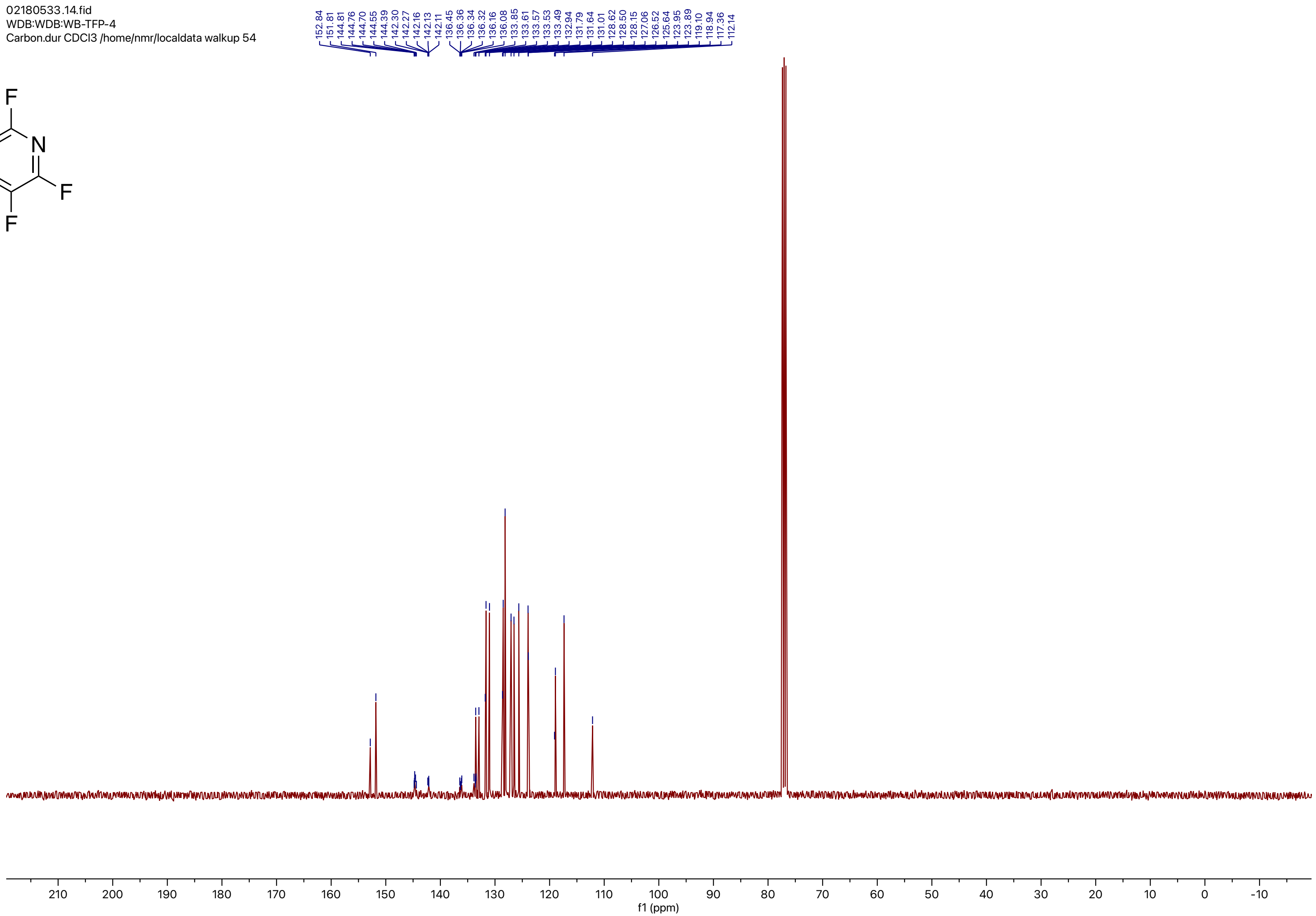

Figure S74. $^{13}C\{^{1}H\}$ NMR spectrum of **28** recorded at 101 MHz in $CDCl_3$.

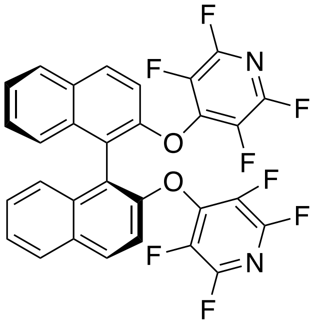

29

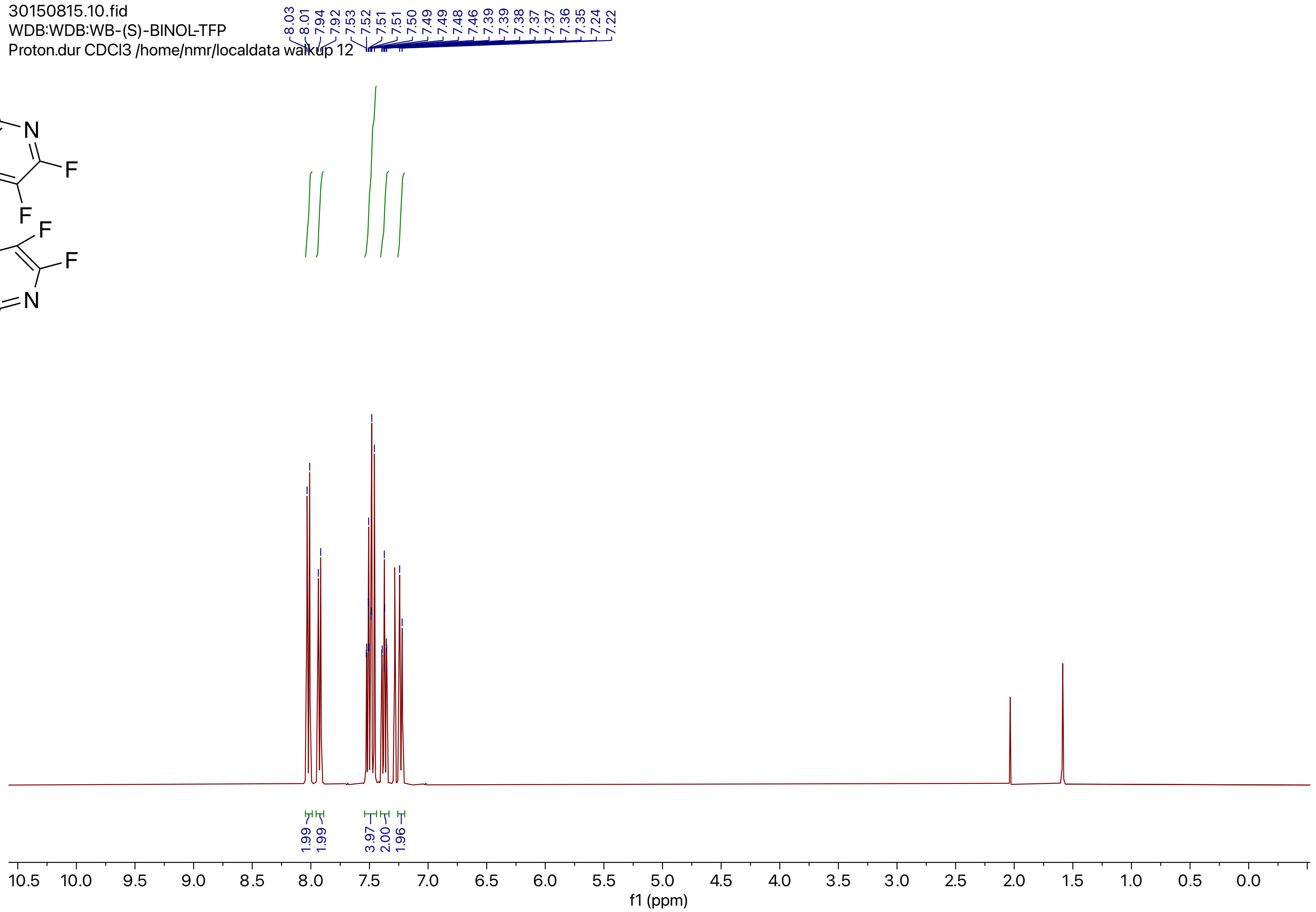

Figure S75. ^{1}H NMR spectrum of **29** recorded at 400 MHz in $CDCl_3$.

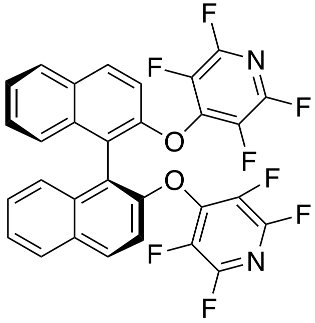

29

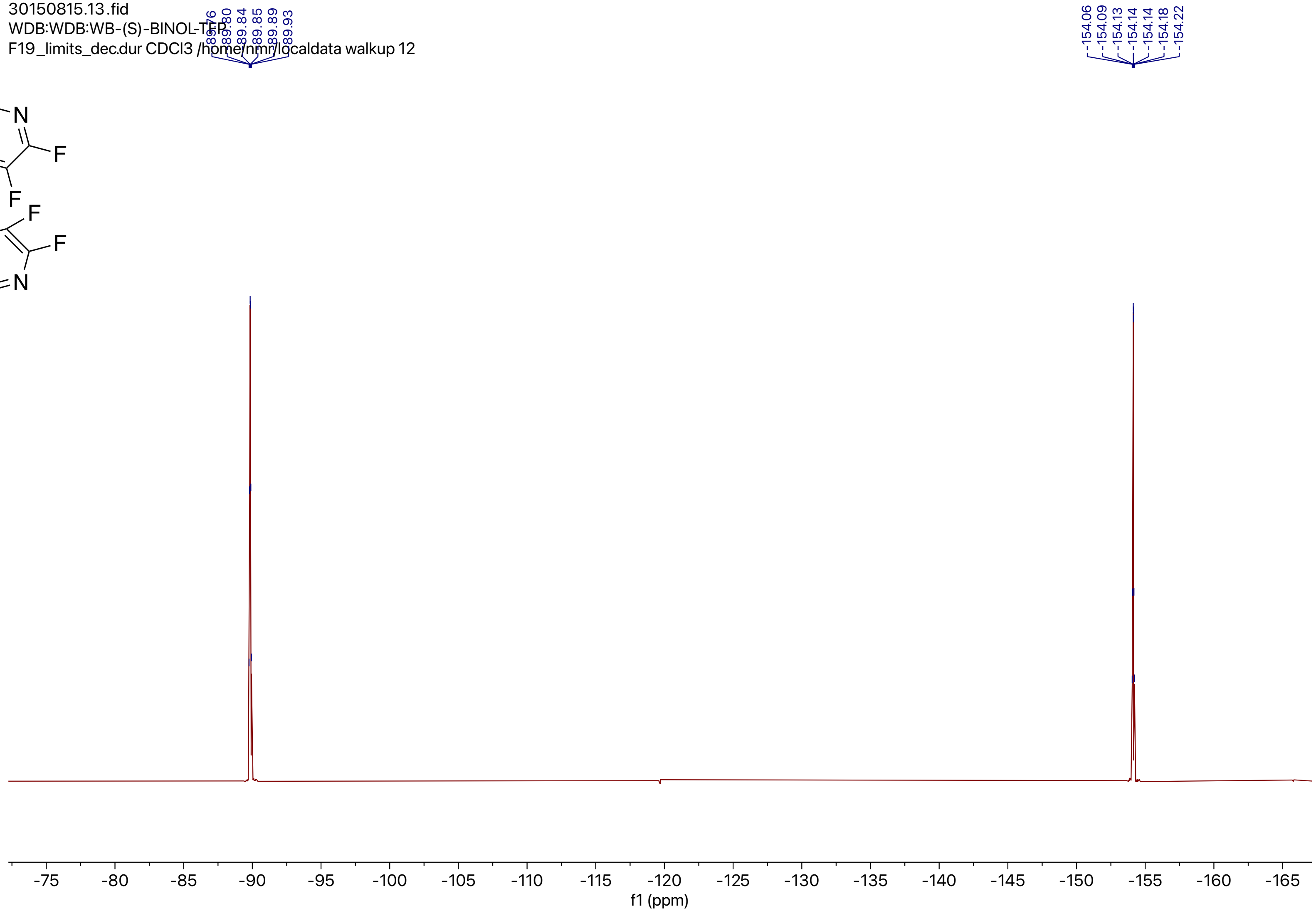

Figure S76. $^{19}F\{^{1}H\}$ NMR spectrum of **29** recorded at 376 MHz in $CDCl_3$.

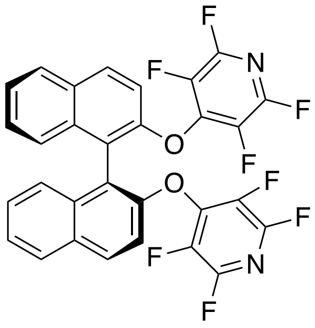

29

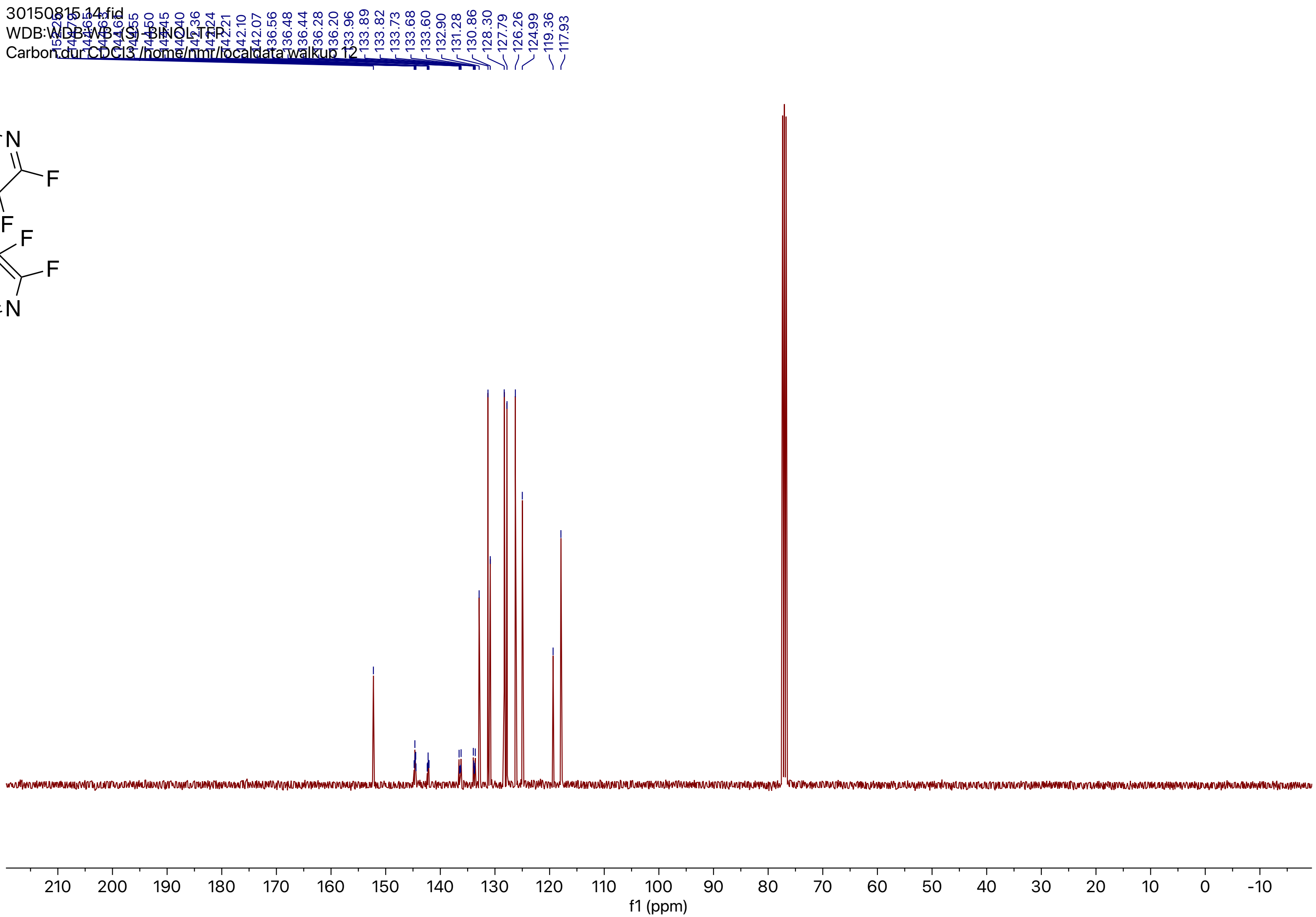

Figure S77. $^{13}C\{^{1}H\}$ NMR spectrum of **29** recorded at 101 MHz in $CDCl_3$.

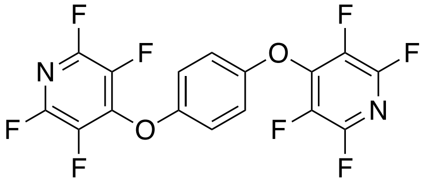

30

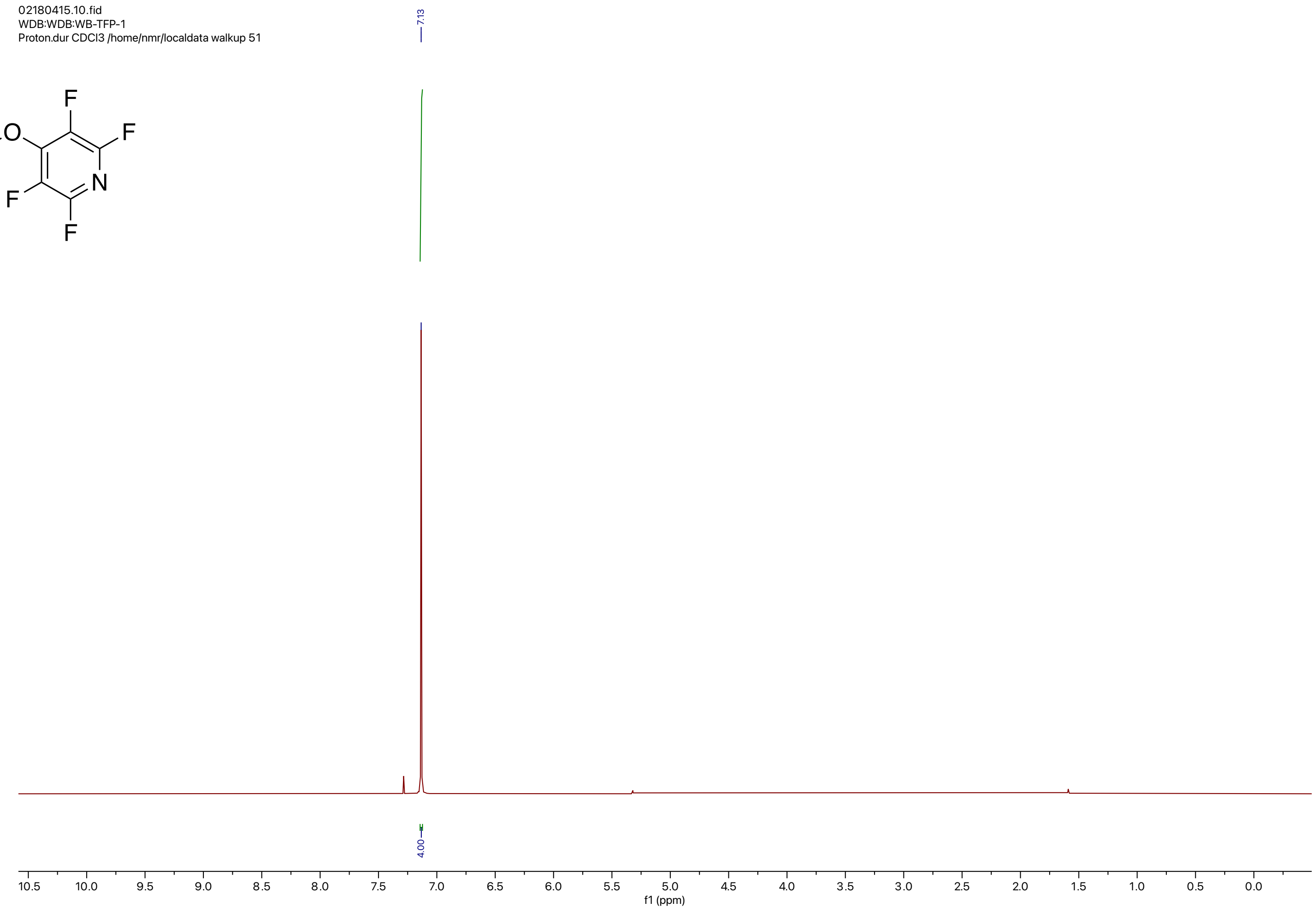

Figure S78. ^{1}H NMR spectrum of **30** recorded at 400 MHz in $CDCl_3$.

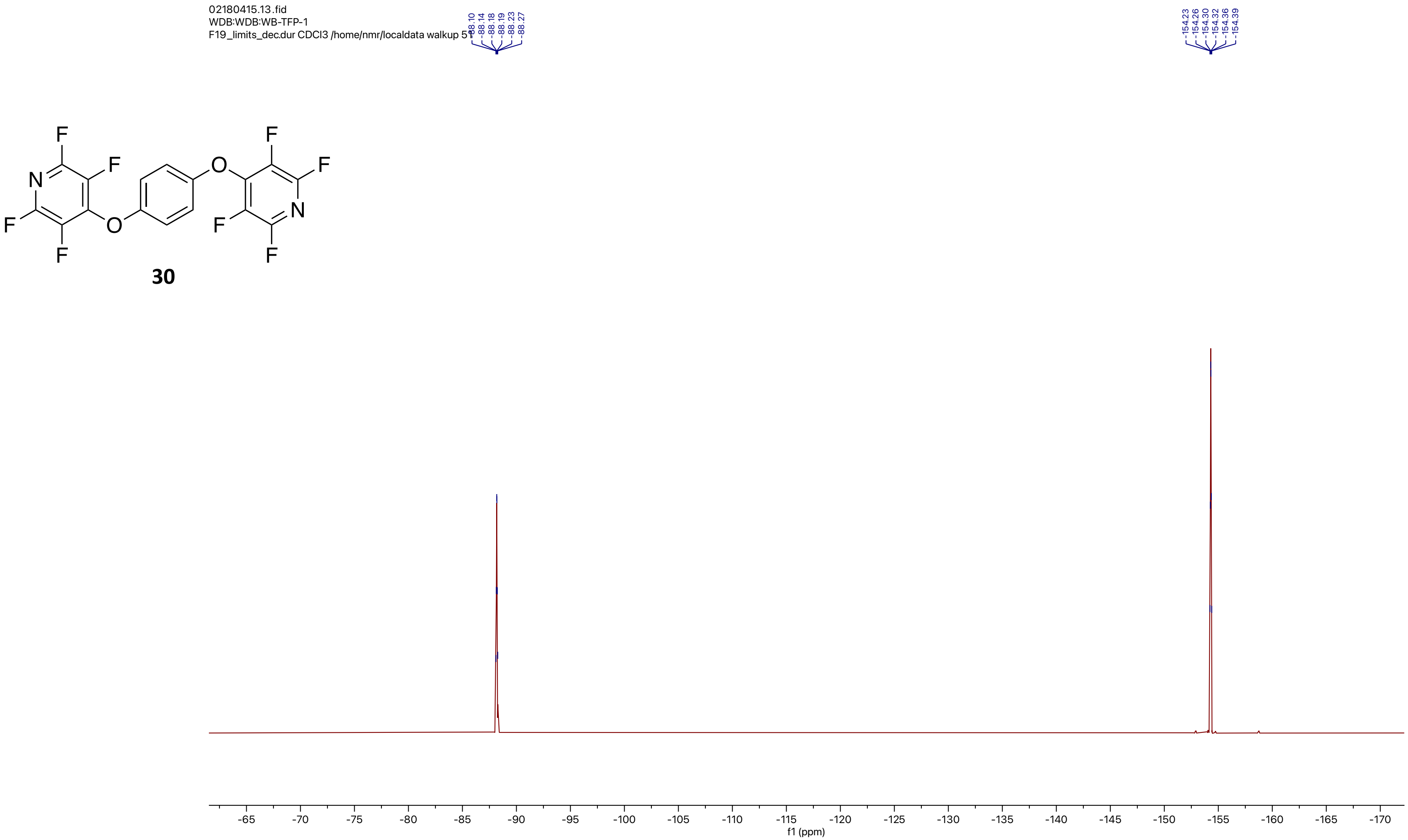

Figure S79. $^{19}F\{^1H\}$ NMR spectrum of **30** recorded at 376 MHz in $CDCl_3$.

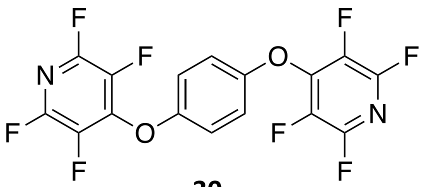

30

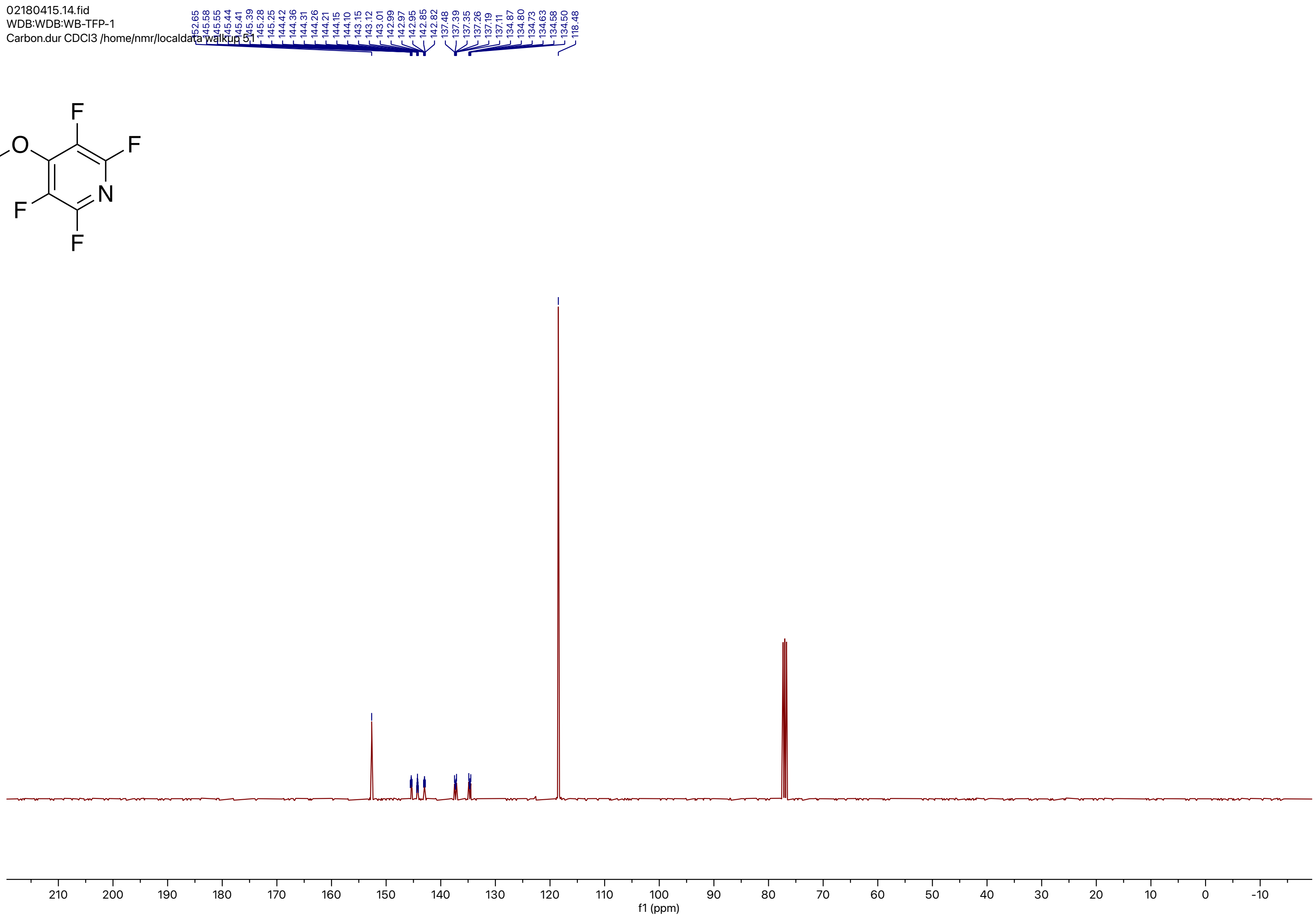

Figure S80. $^{13}C\{^{1}H\}$ NMR spectrum of **30** recorded at 101 MHz in $CDCl_3$.

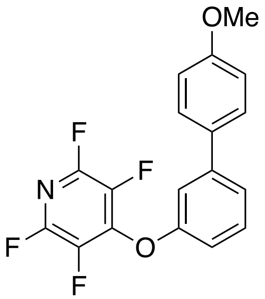

31

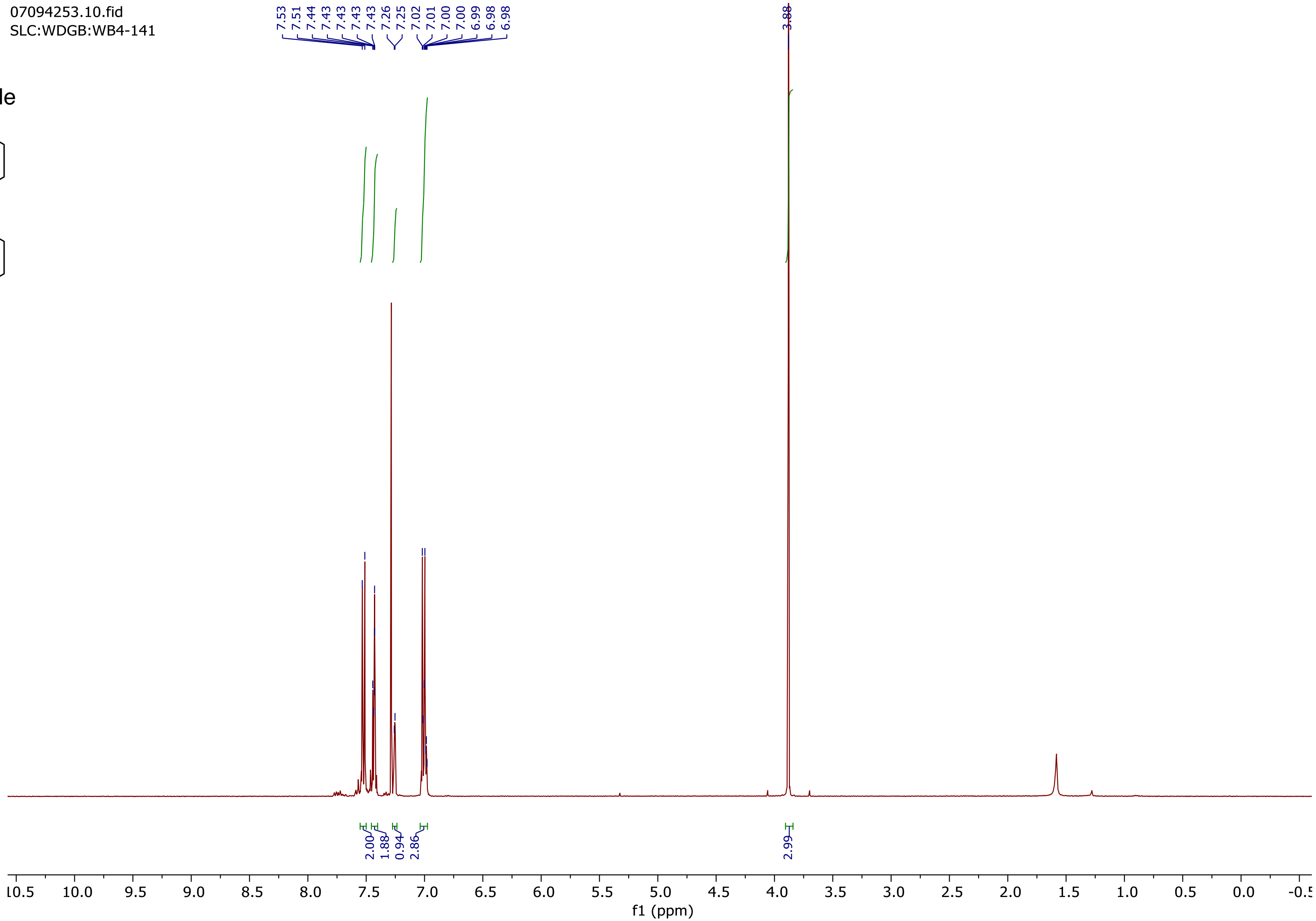

Figure S81. ^{1}H NMR spectrum of **31** recorded at 400 MHz in $CDCl_3$.

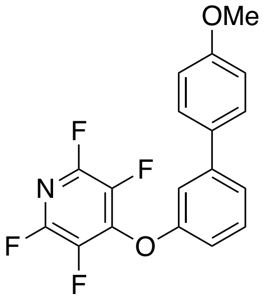

31

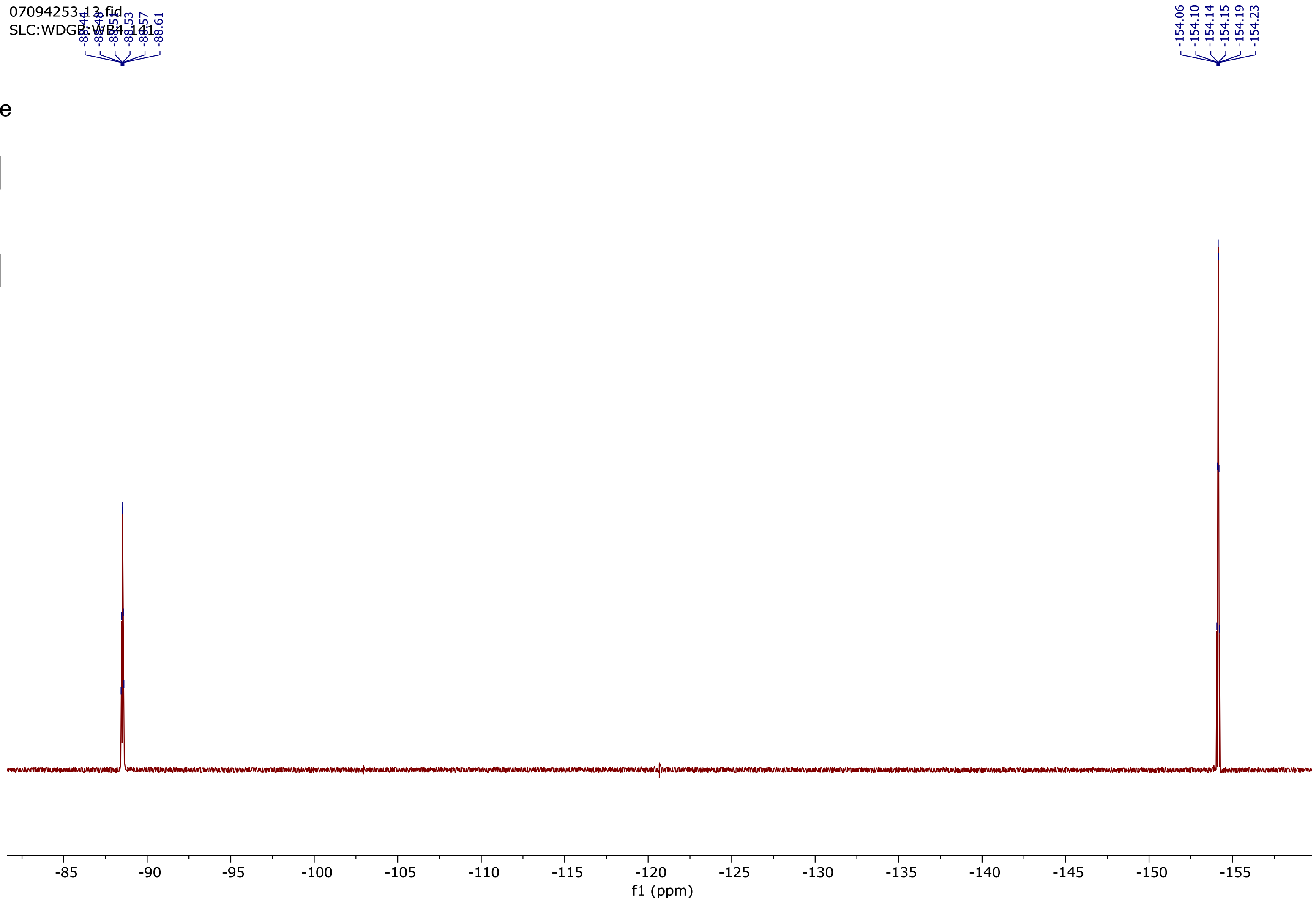

Figure S82. $^{19}F\{^{1}H\}$ NMR spectrum of **30** recorded at 376 MHz in $CDCl_3$.

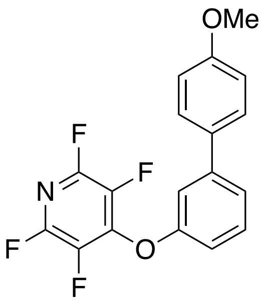

31

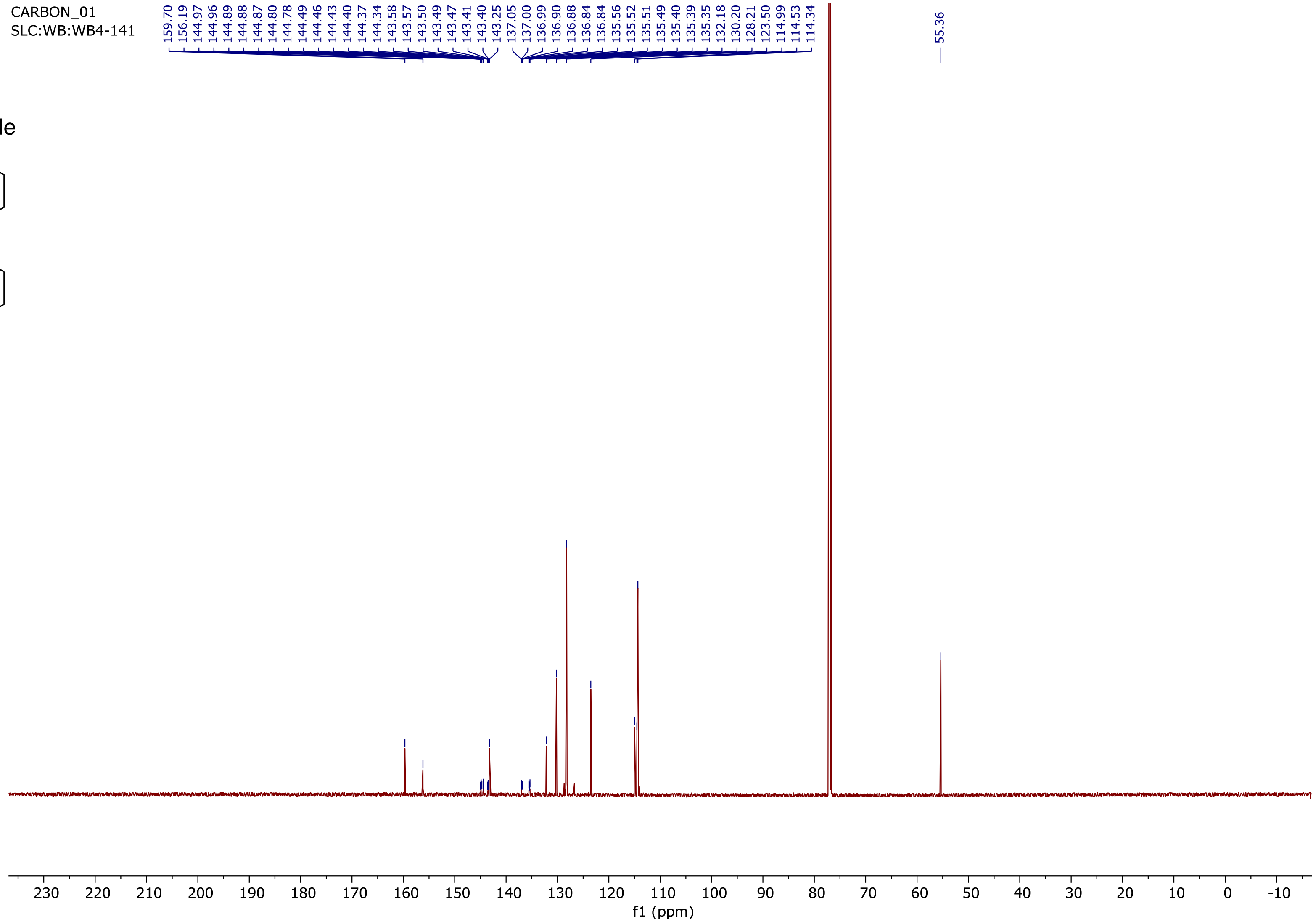

Figure S83. $^{13}C\{^{1}H\}$ NMR spectrum of **31** recorded at 176 MHz in $CDCl_3$.

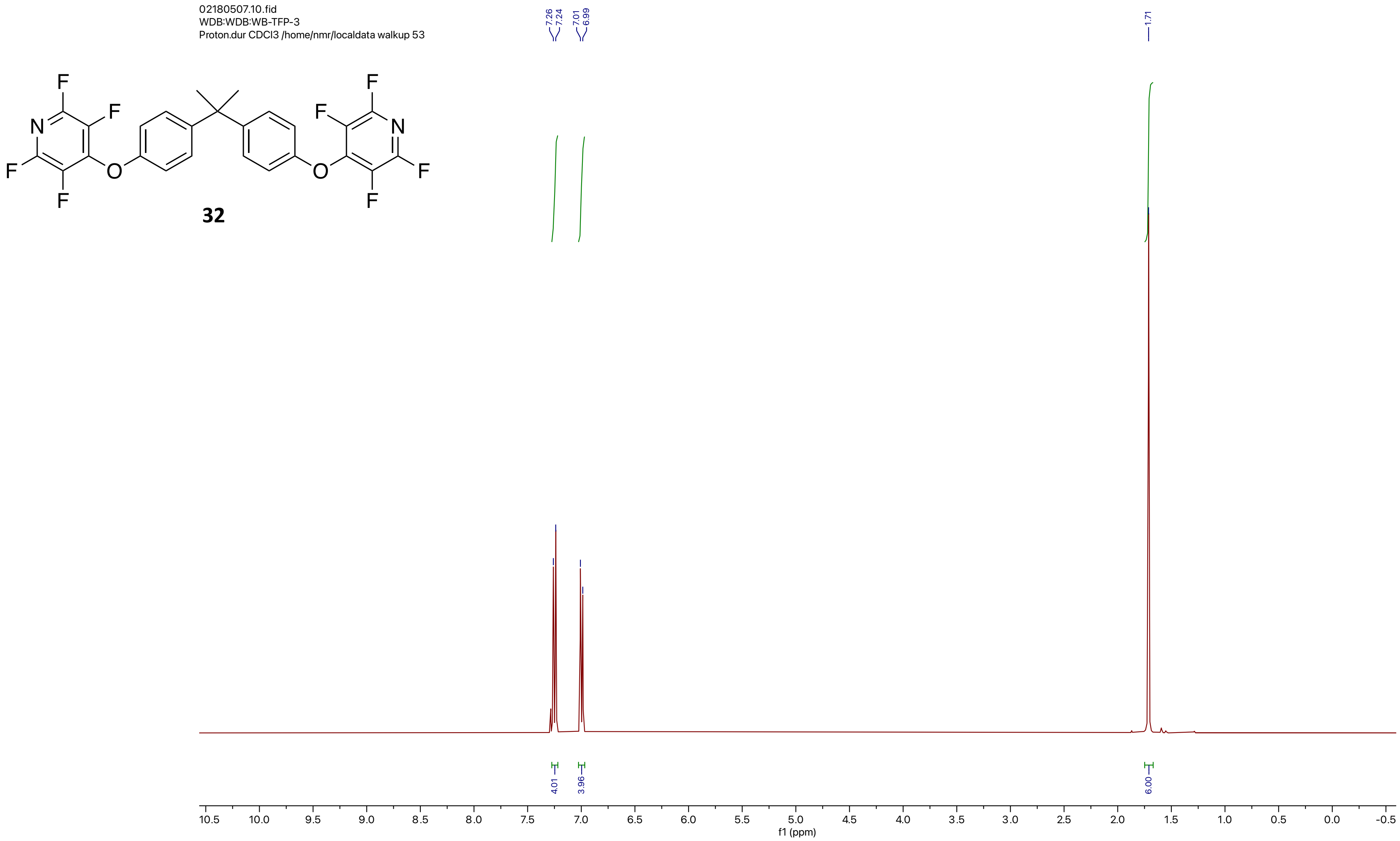

Figure S84. ^{1}H NMR spectrum of **32** recorded at 400 MHz in $CDCl_3$.

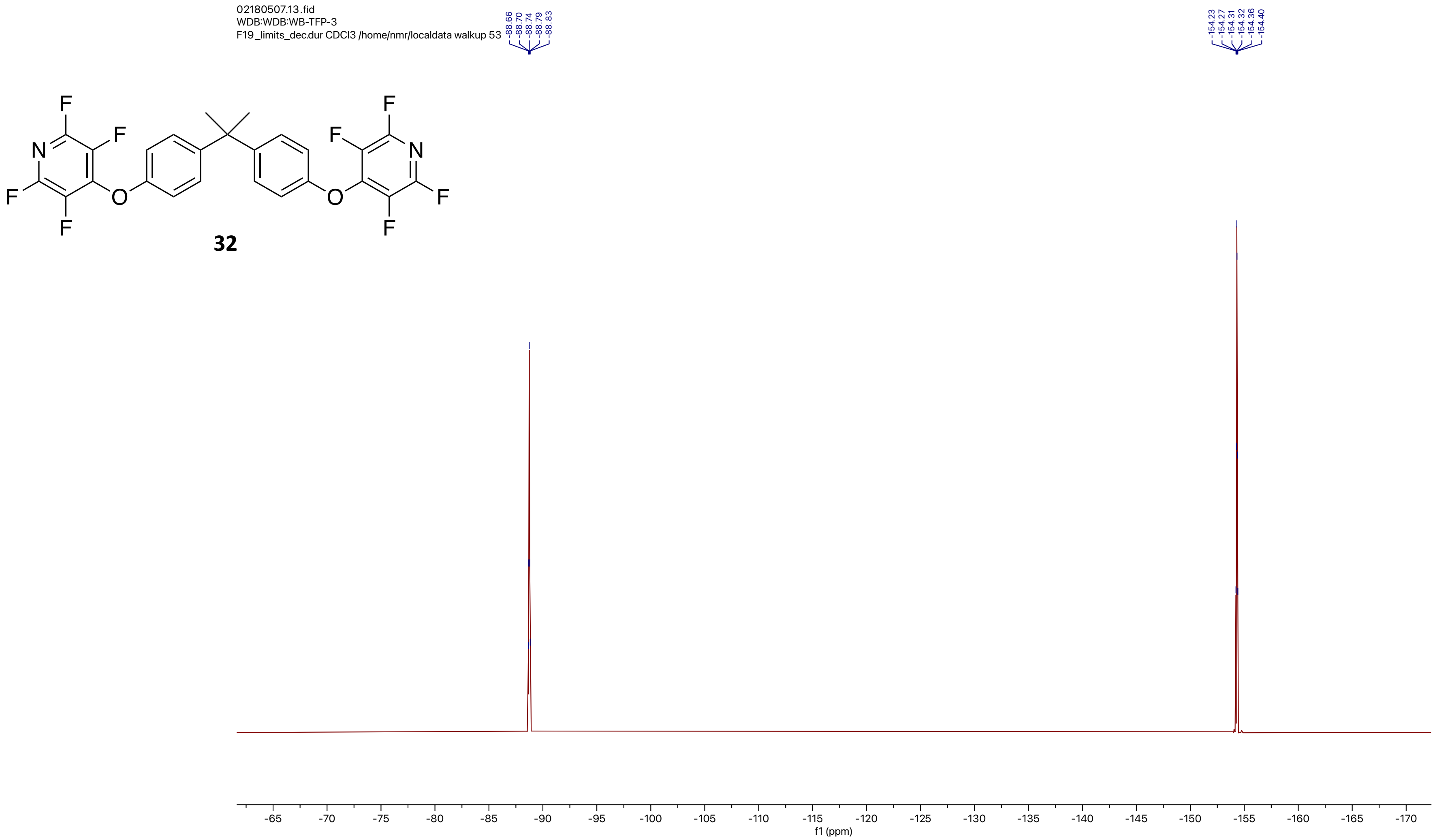

Figure S85. $^{19}F\{^{1}H\}$ NMR spectrum of **32** recorded at 376 MHz in $CDCl_3$.

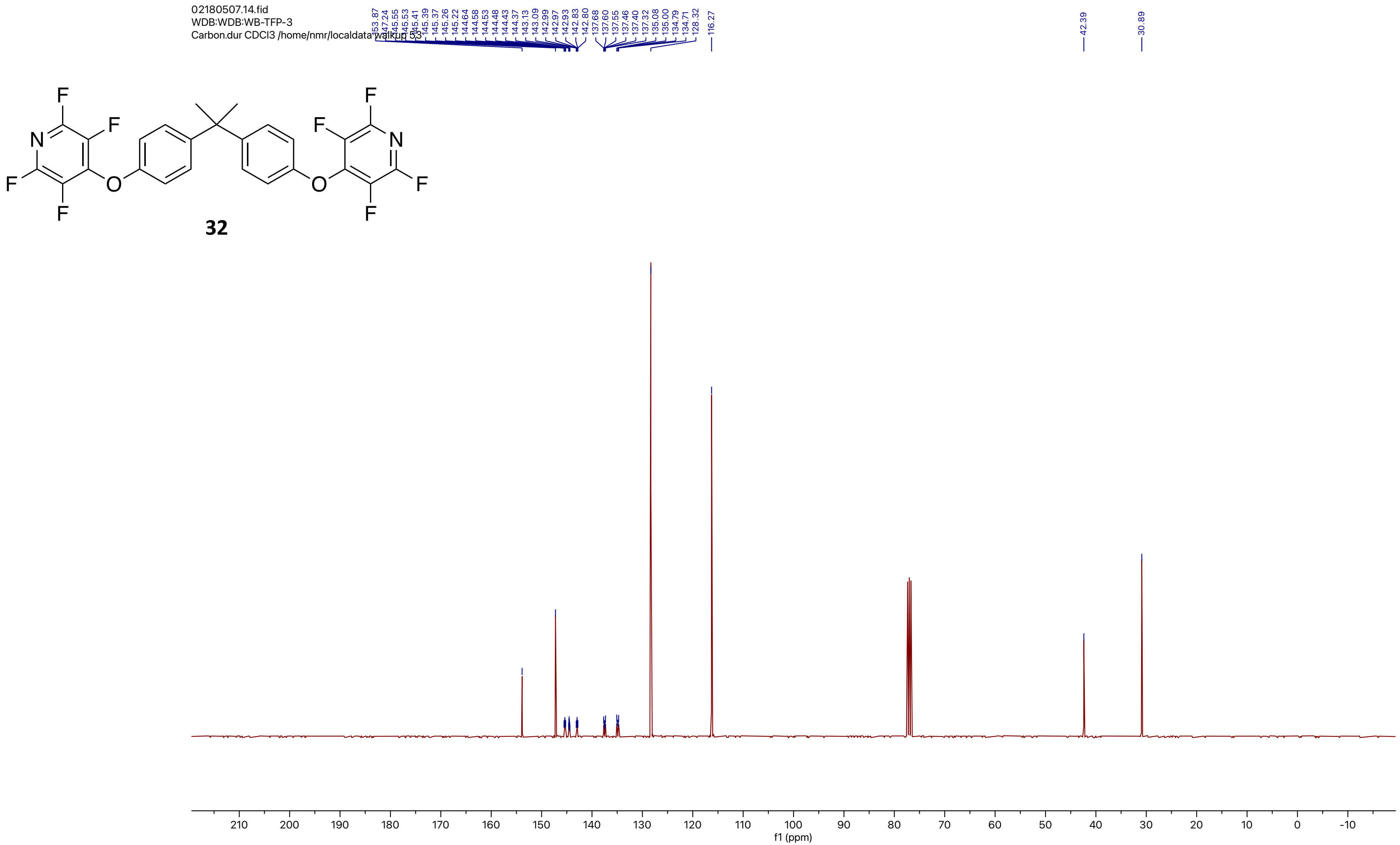

Figure S86. $^{13}C\{^{1}H\}$ NMR spectrum of **32** recorded at 101 MHz in $CDCl_3$.

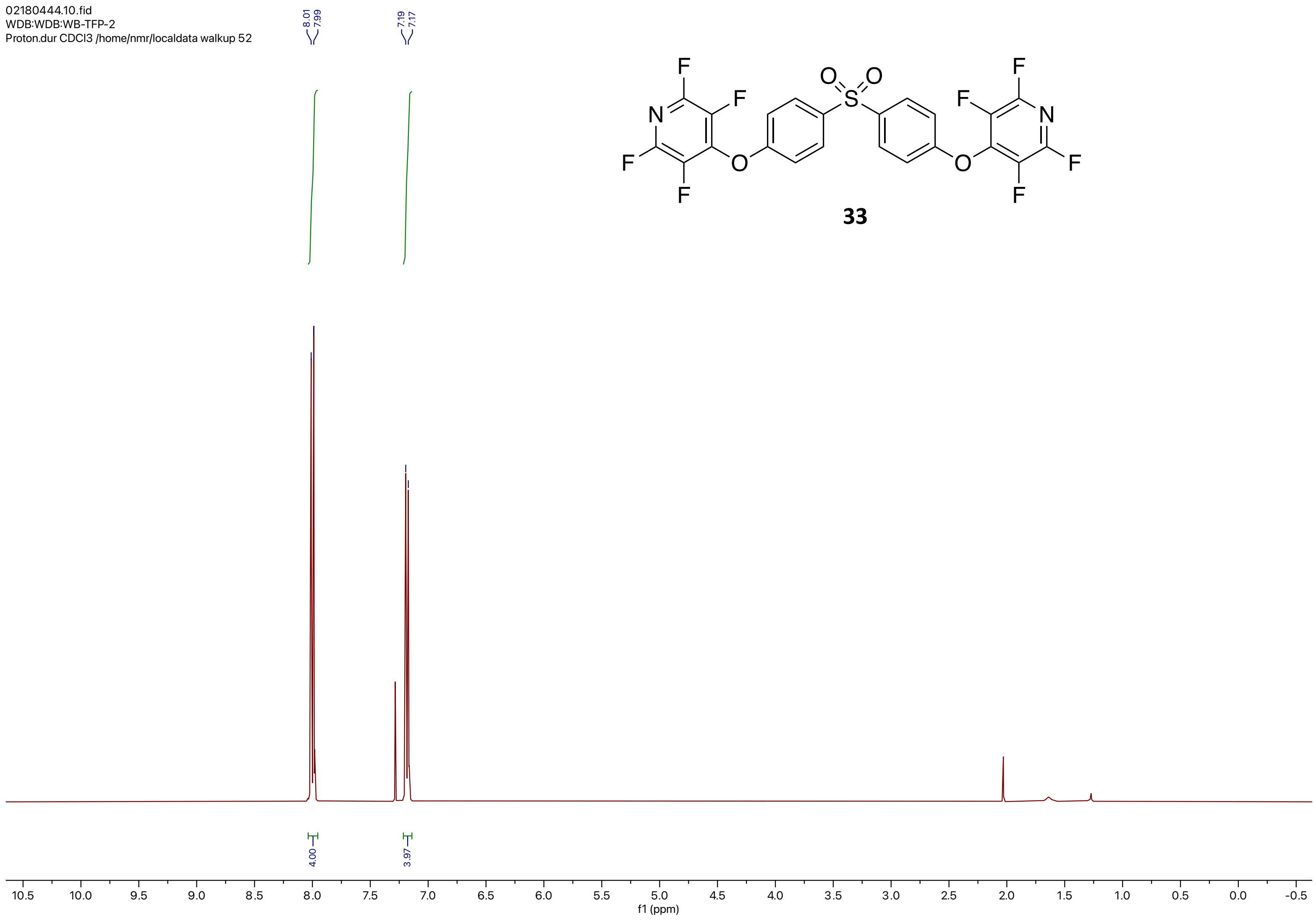

33

Figure S87. ^{1}H NMR spectrum of **33** recorded at 400 MHz in $CDCl_3$.

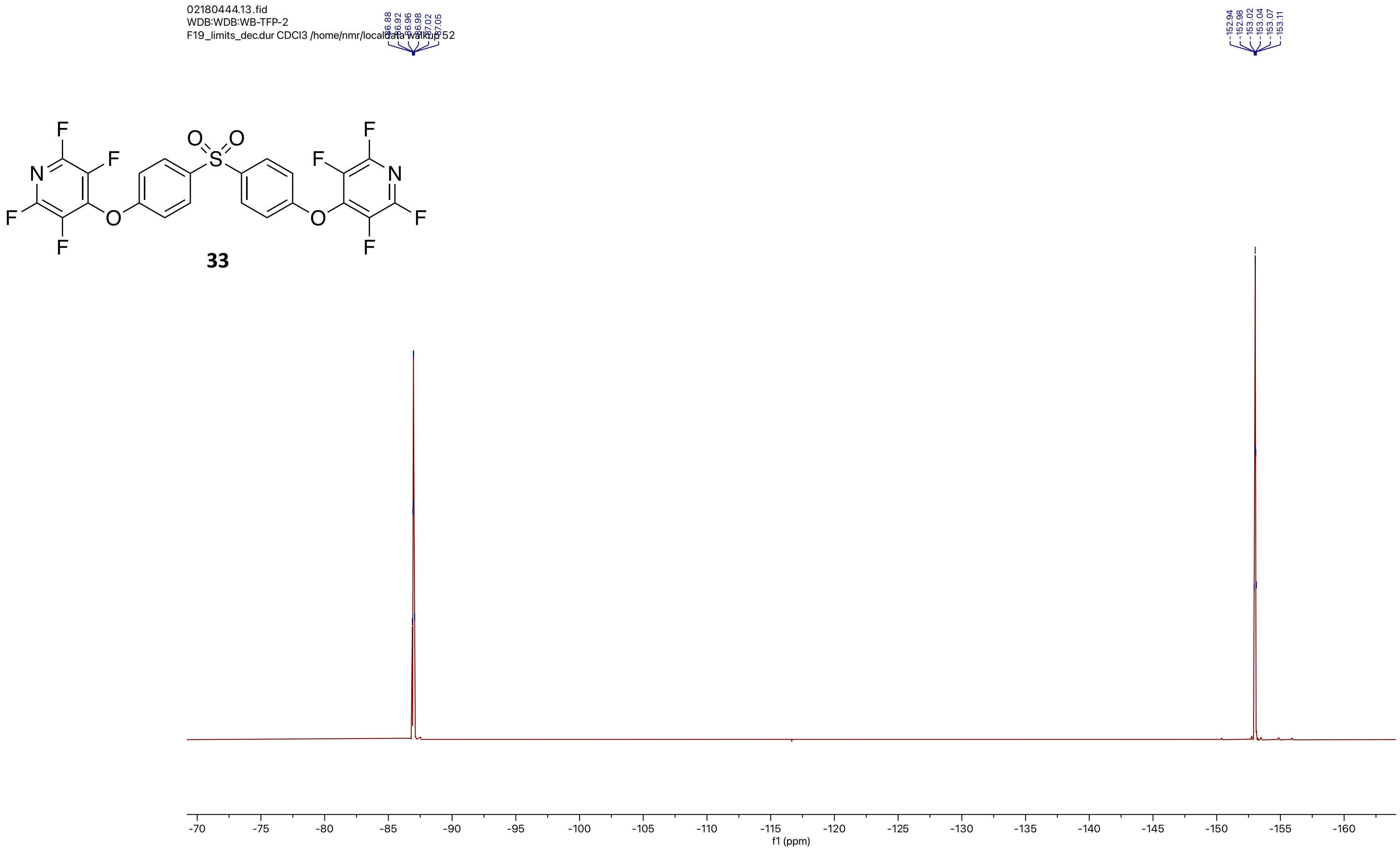

Figure S88. $^{19}F\{^{1}H\}$ NMR spectrum of **33** recorded at 376 MHz in $CDCl_3$.

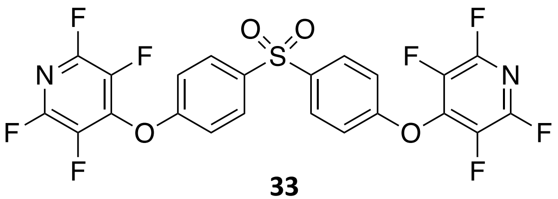

33

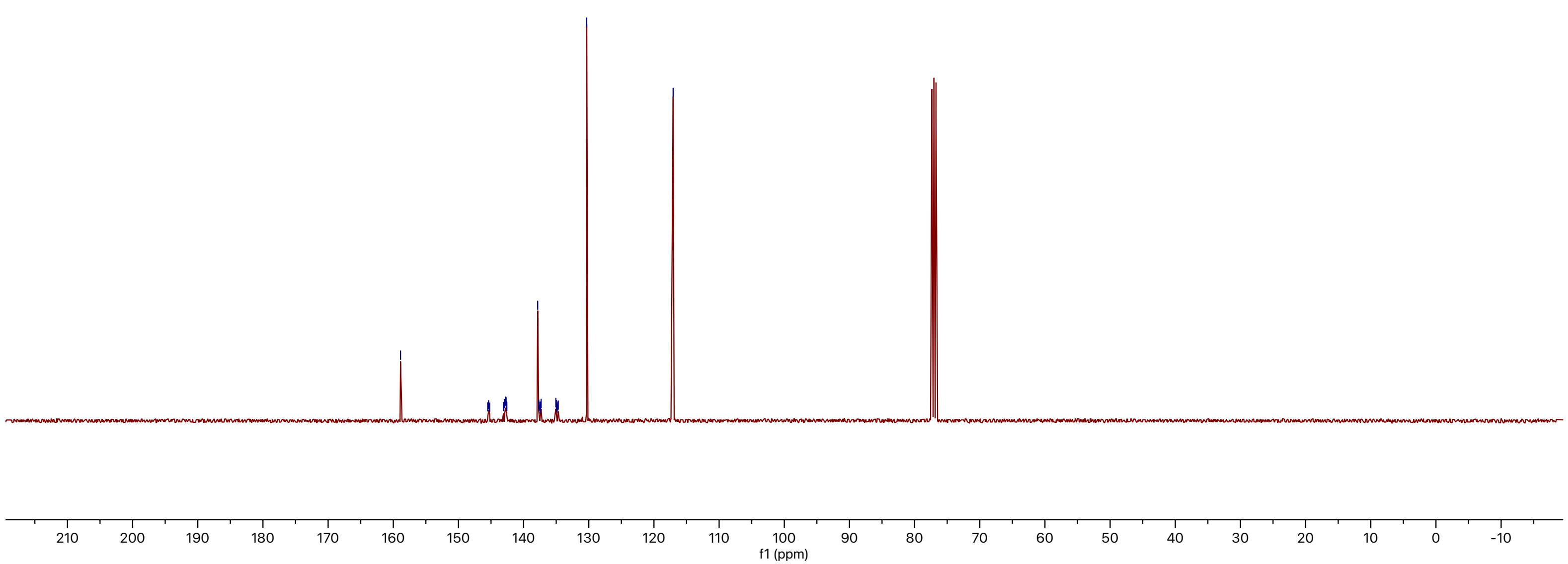

Figure S89. $^{13}C\{^{1}H\}$ NMR spectrum of **33** recorded at 101 MHz in $CDCl_3$.

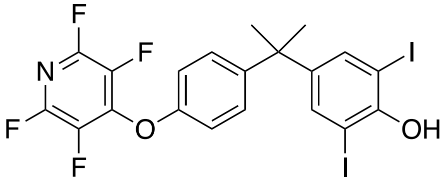

34

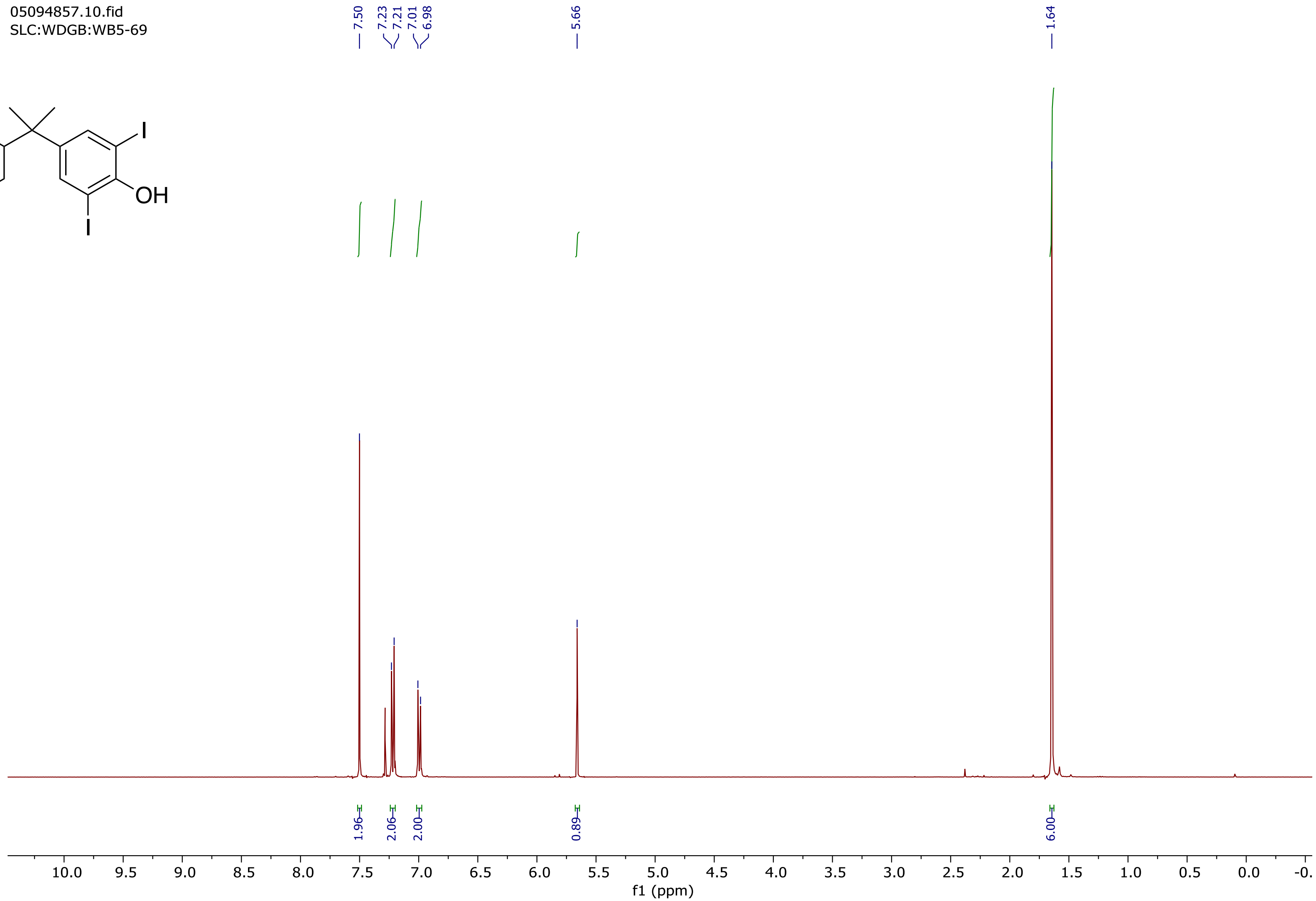

Figure S90. ^{1}H NMR spectrum of **34** recorded at 400 MHz in $CDCl_3$.

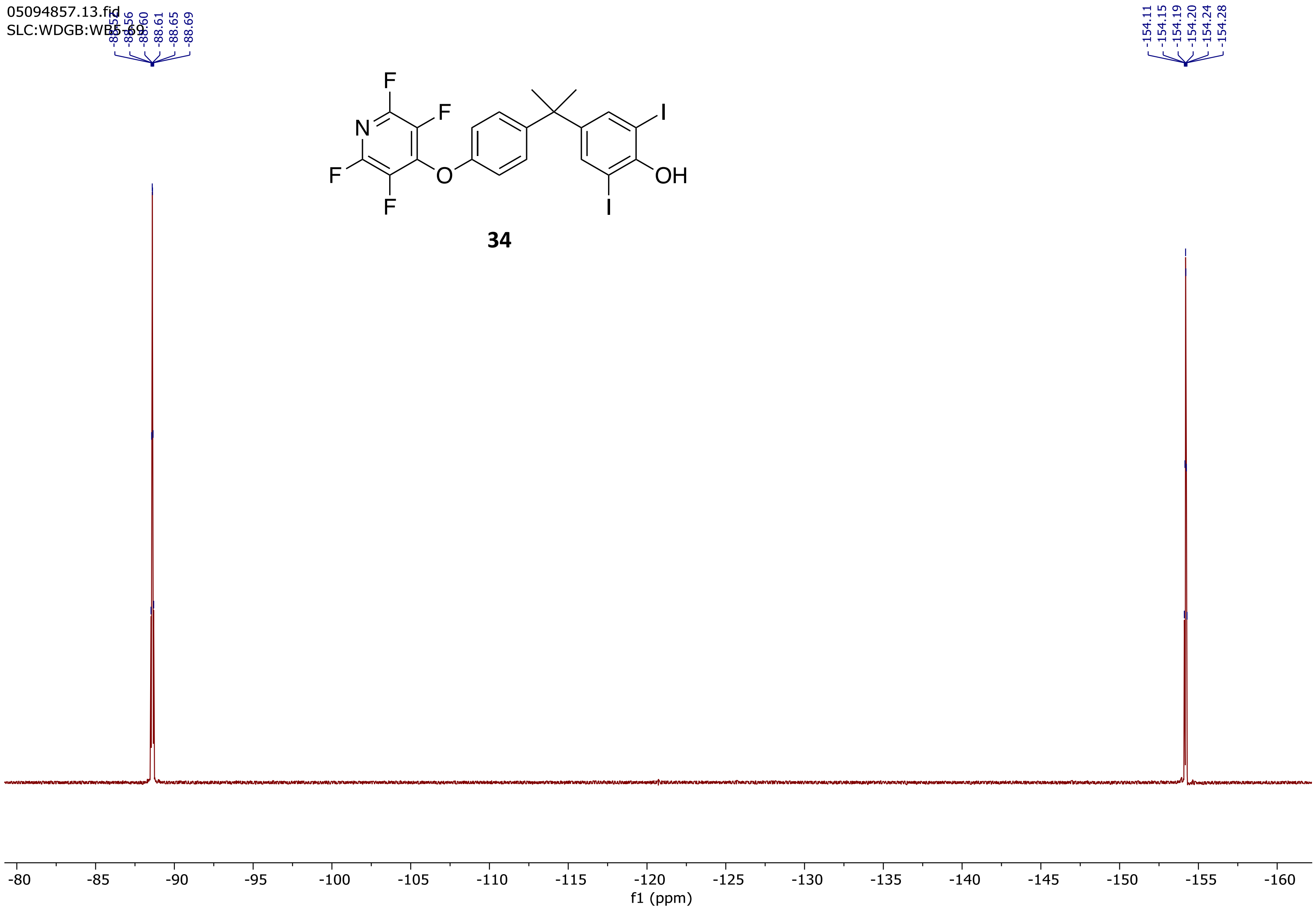

Figure S91. $^{19}F\{^1H\}$ NMR spectrum of **34** recorded at 376 MHz in $CDCl_3$.

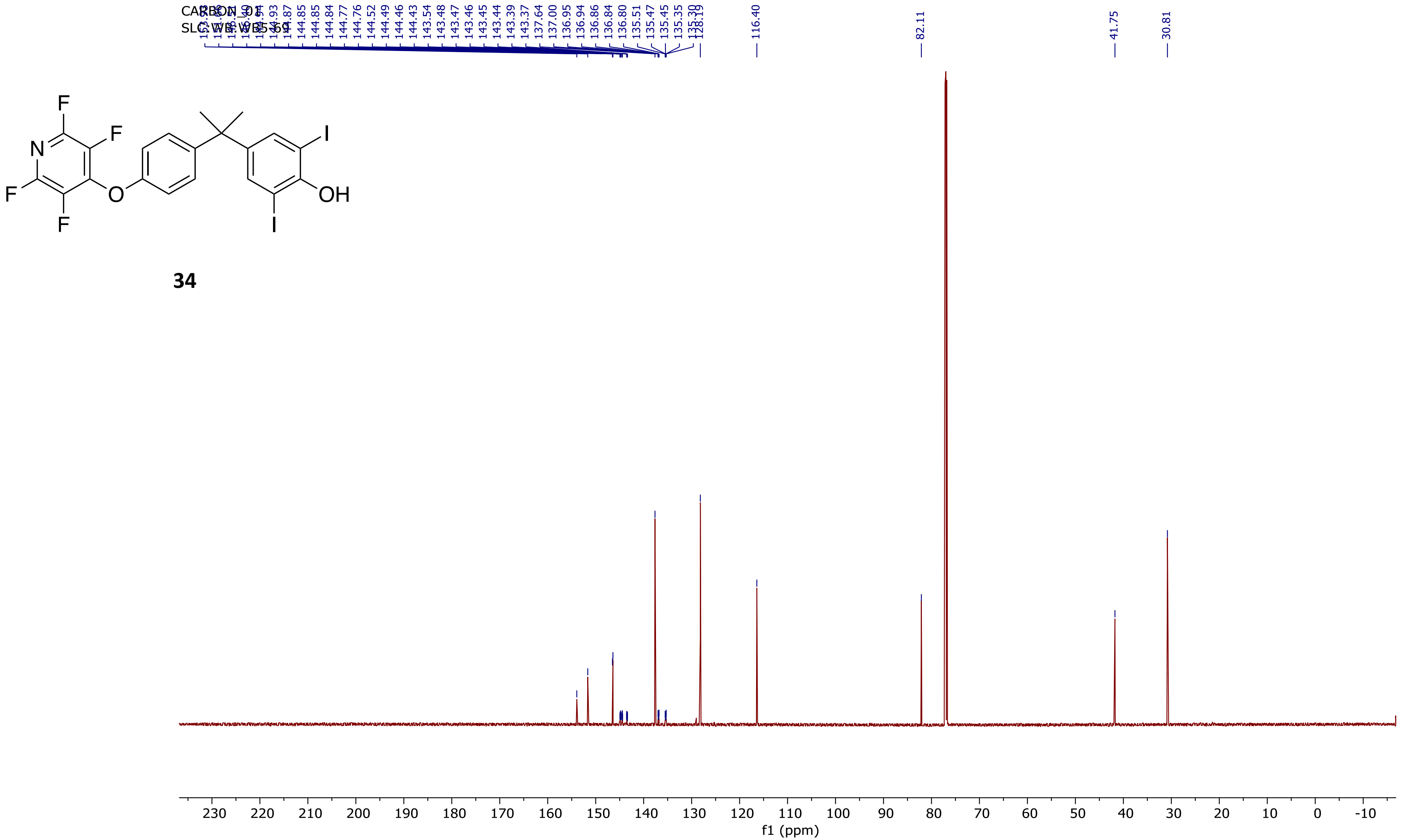

34

Figure S92. $^{13}C\{^{1}H\}$ NMR spectrum of **34** recorded at 176 MHz in $CDCl_3$.

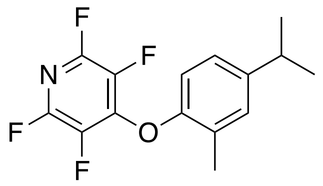

35

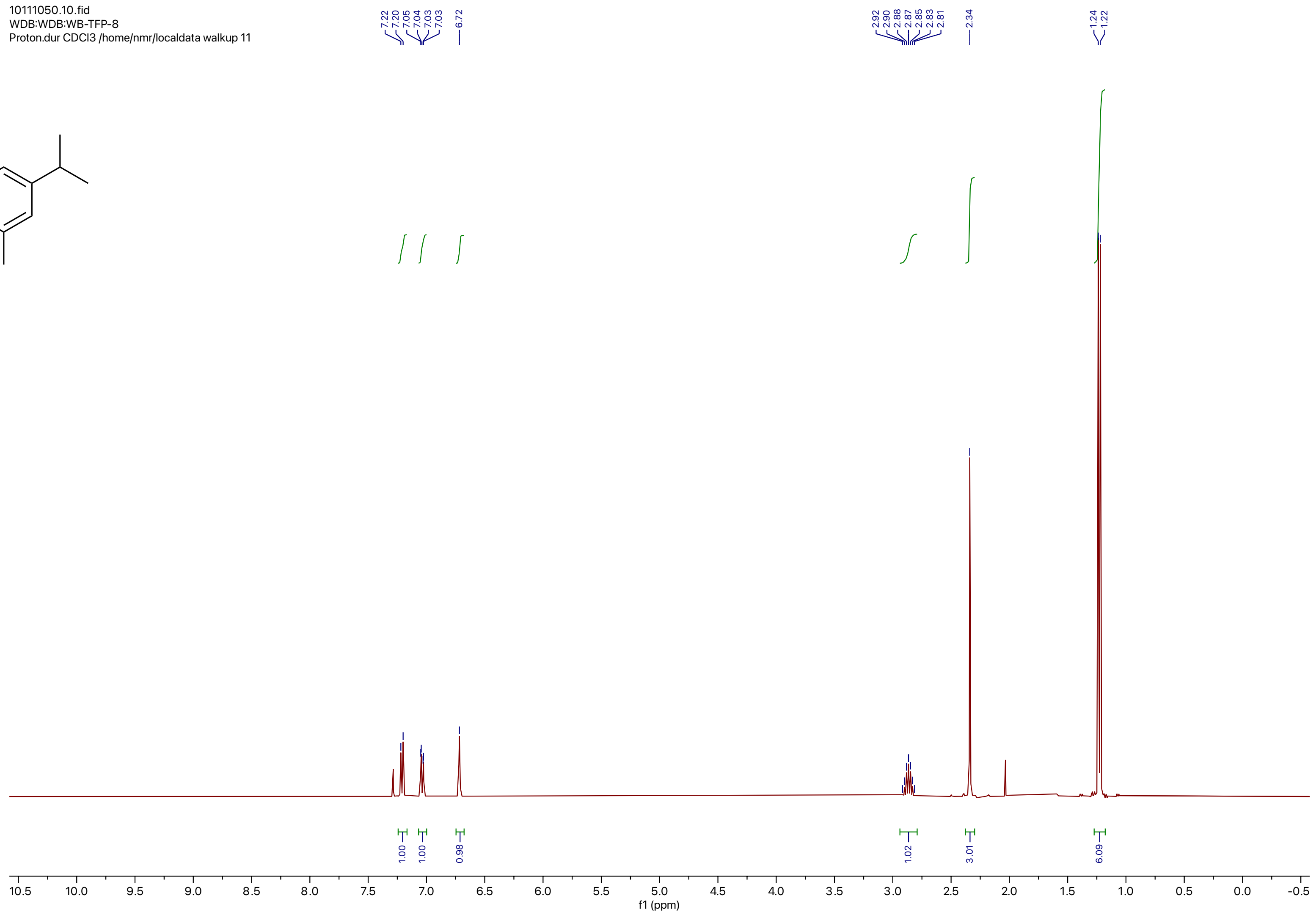

Figure S93. ^{1}H NMR spectrum of **35** recorded at 400 MHz in $CDCl_3$.

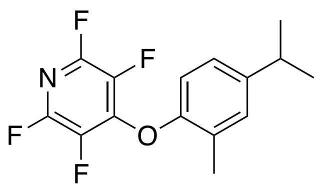

35

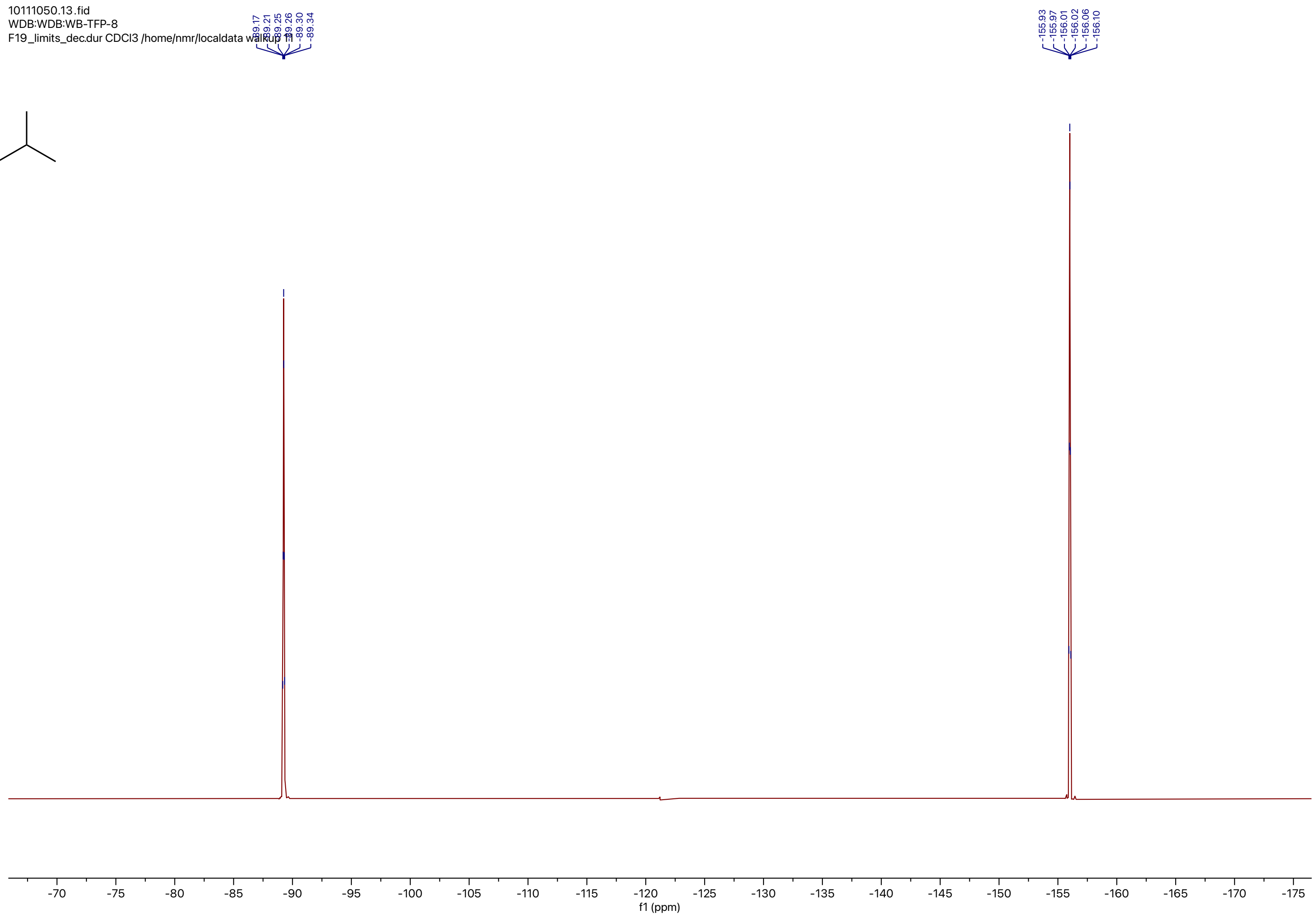

Figure S94. $^{19}F\{^{1}H\}$ NMR spectrum of **35** recorded at 376 MHz in $CDCl_3$.

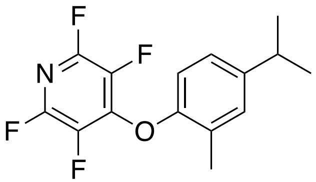

35

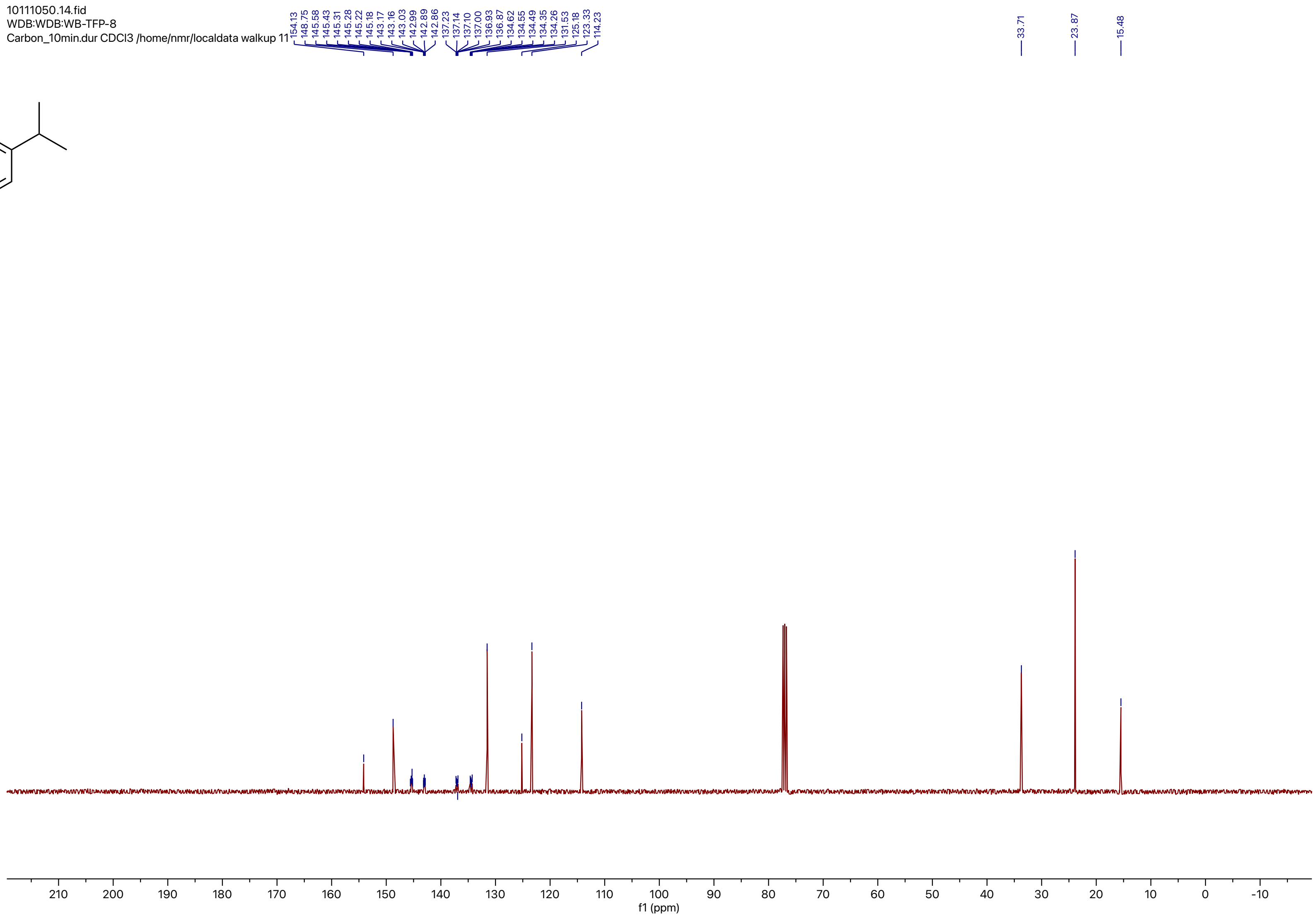

Figure S95. $^{13}C\{^{1}H\}$ NMR spectrum of **35** recorded at 101 MHz in $CDCl_3$.

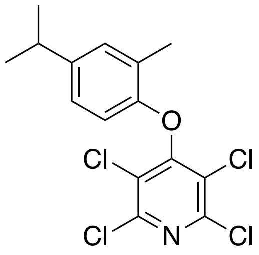

36

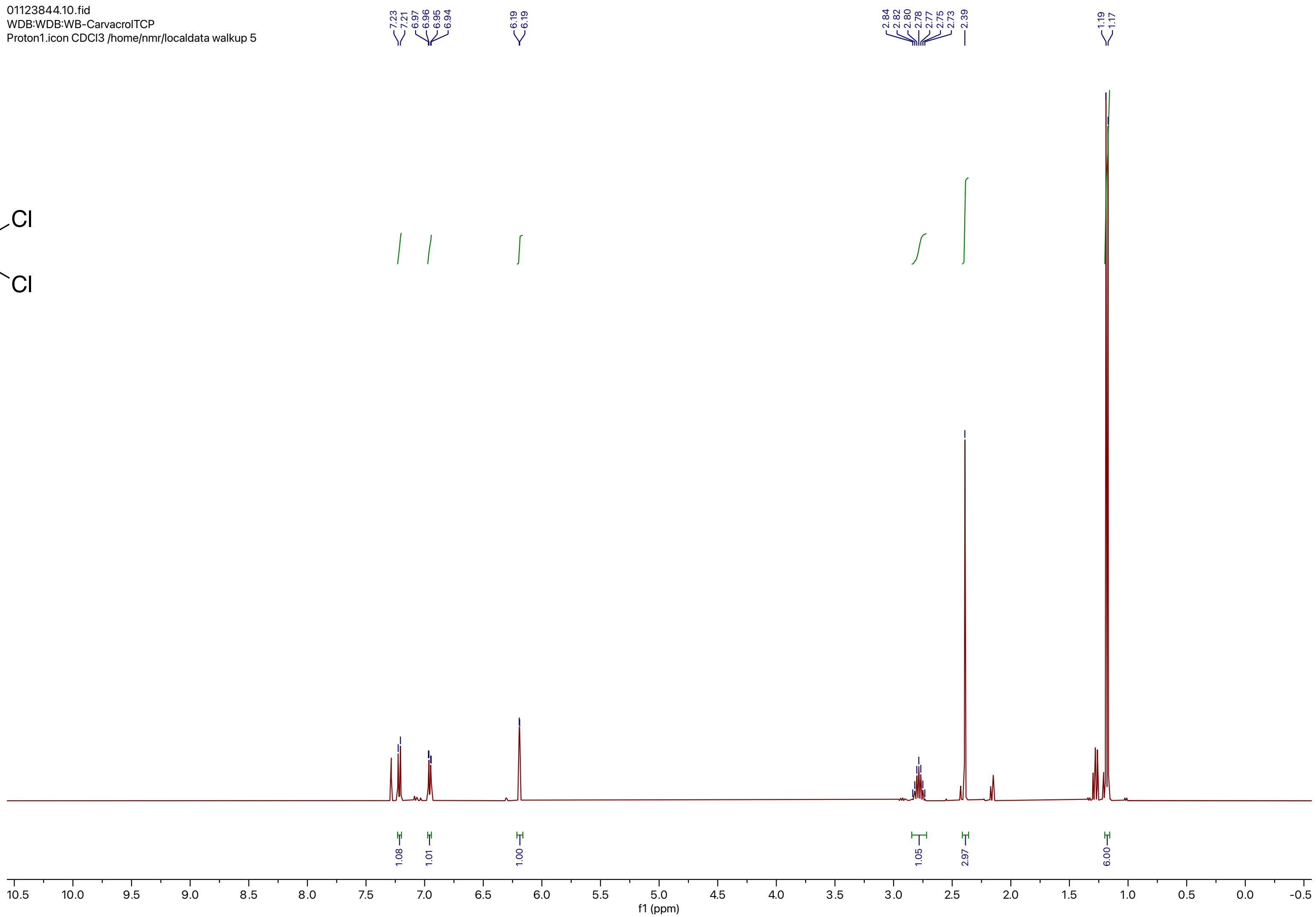

Figure S96. ^{1}H NMR spectrum of **36** recorded at 400 MHz in $CDCl_3$.

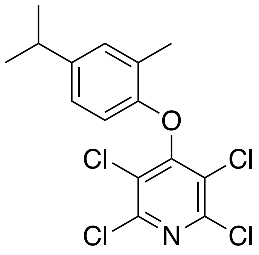

36

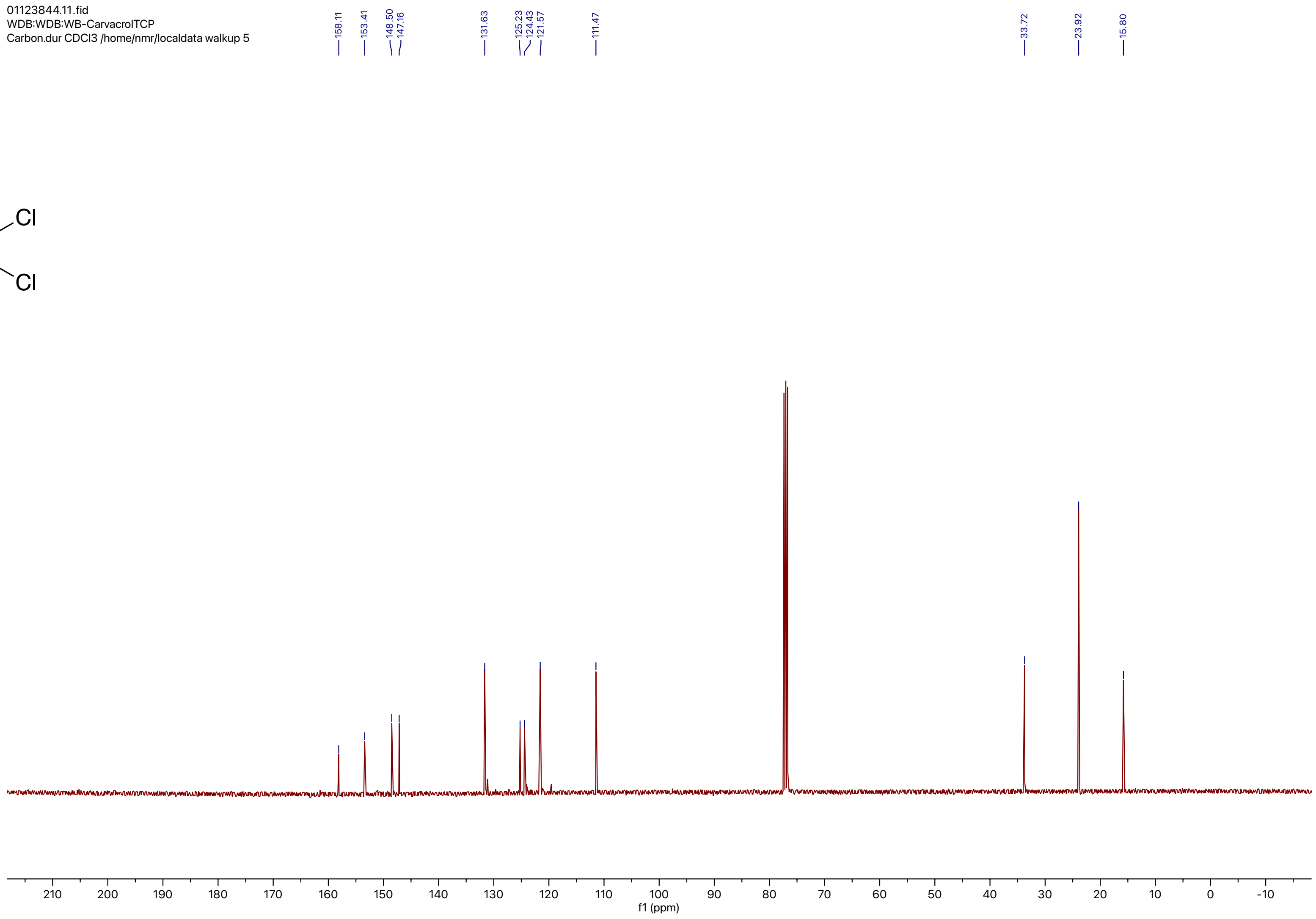

Figure S97. $^{13}C\{^{1}H\}$ NMR spectrum of **36** recorded at 101 MHz in $CDCl_3$.

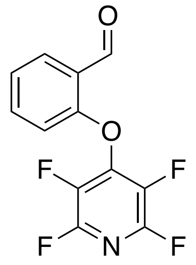

37

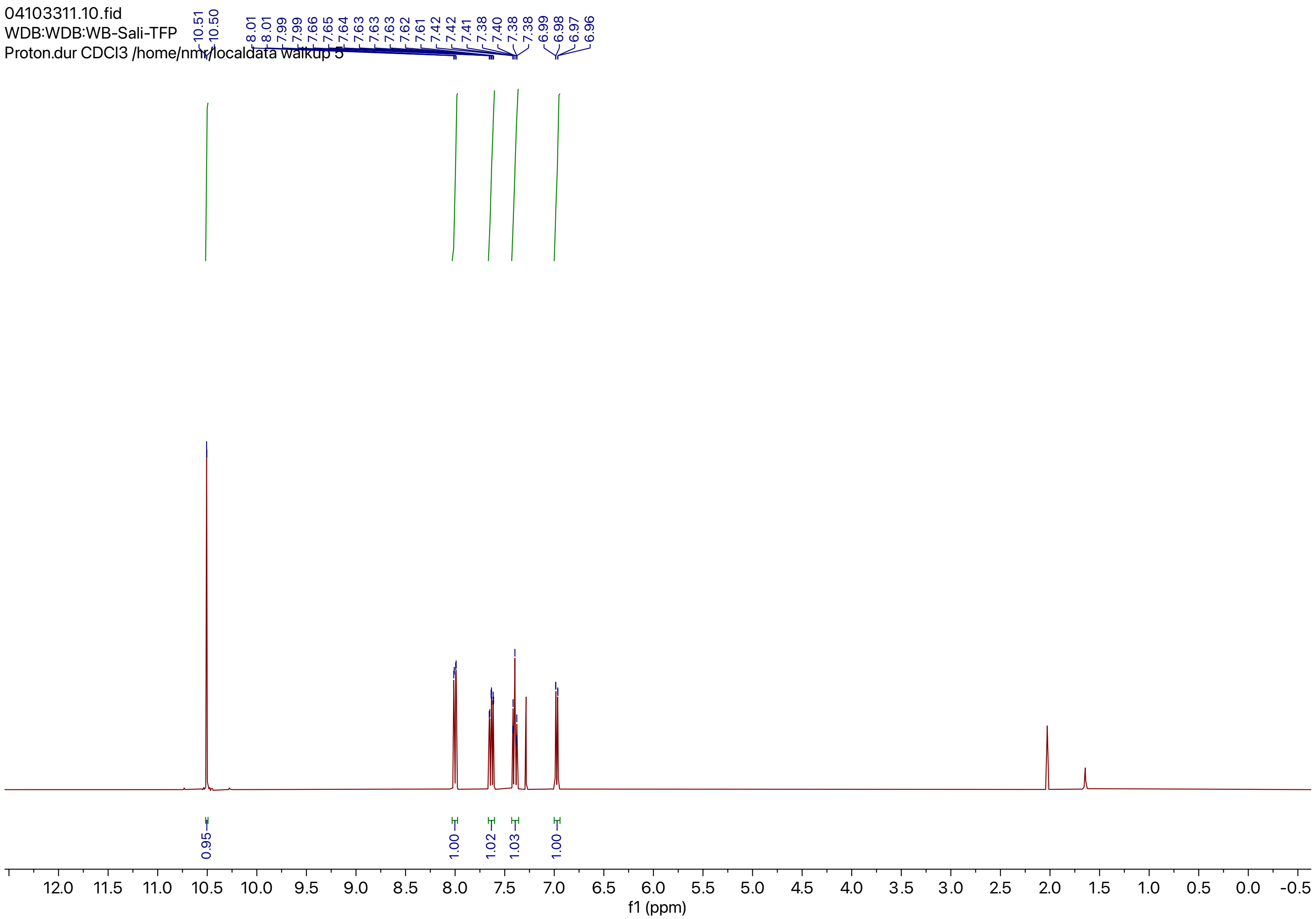

Figure S98. ^{1}H NMR spectrum of **37** recorded at 400 MHz in $CDCl_3$.

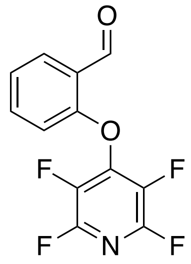

37

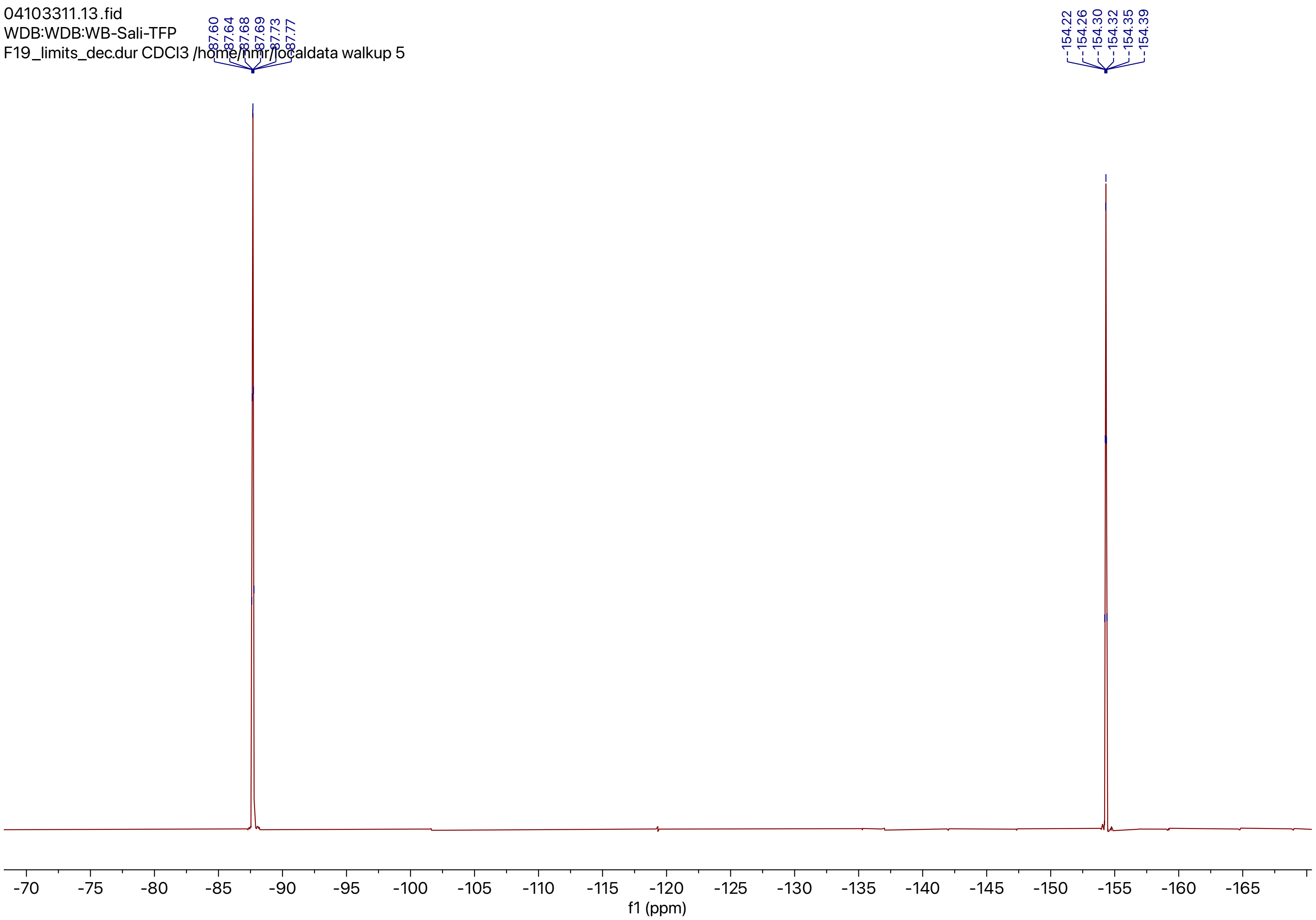

Figure S99. $^{19}F\{^1H\}$ NMR spectrum of **37** recorded at 376 MHz in $CDCl_3$.

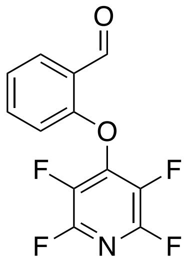

37

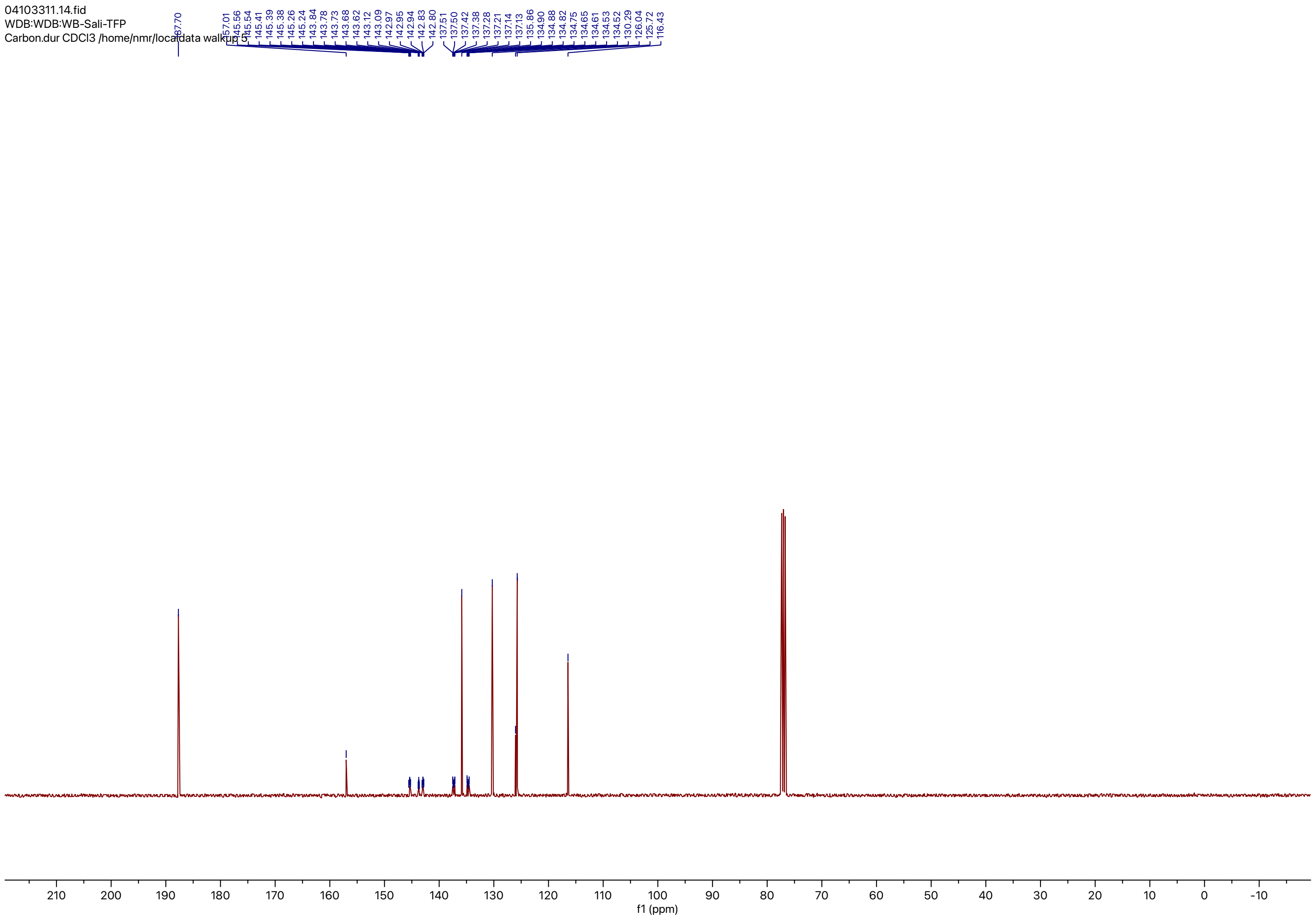

Figure S100. $^{13}C\{^{1}H\}$ NMR spectrum of **37** recorded at 101 MHz in $CDCl_3$.

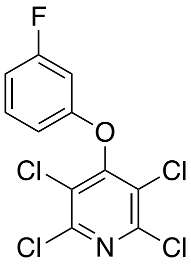

38

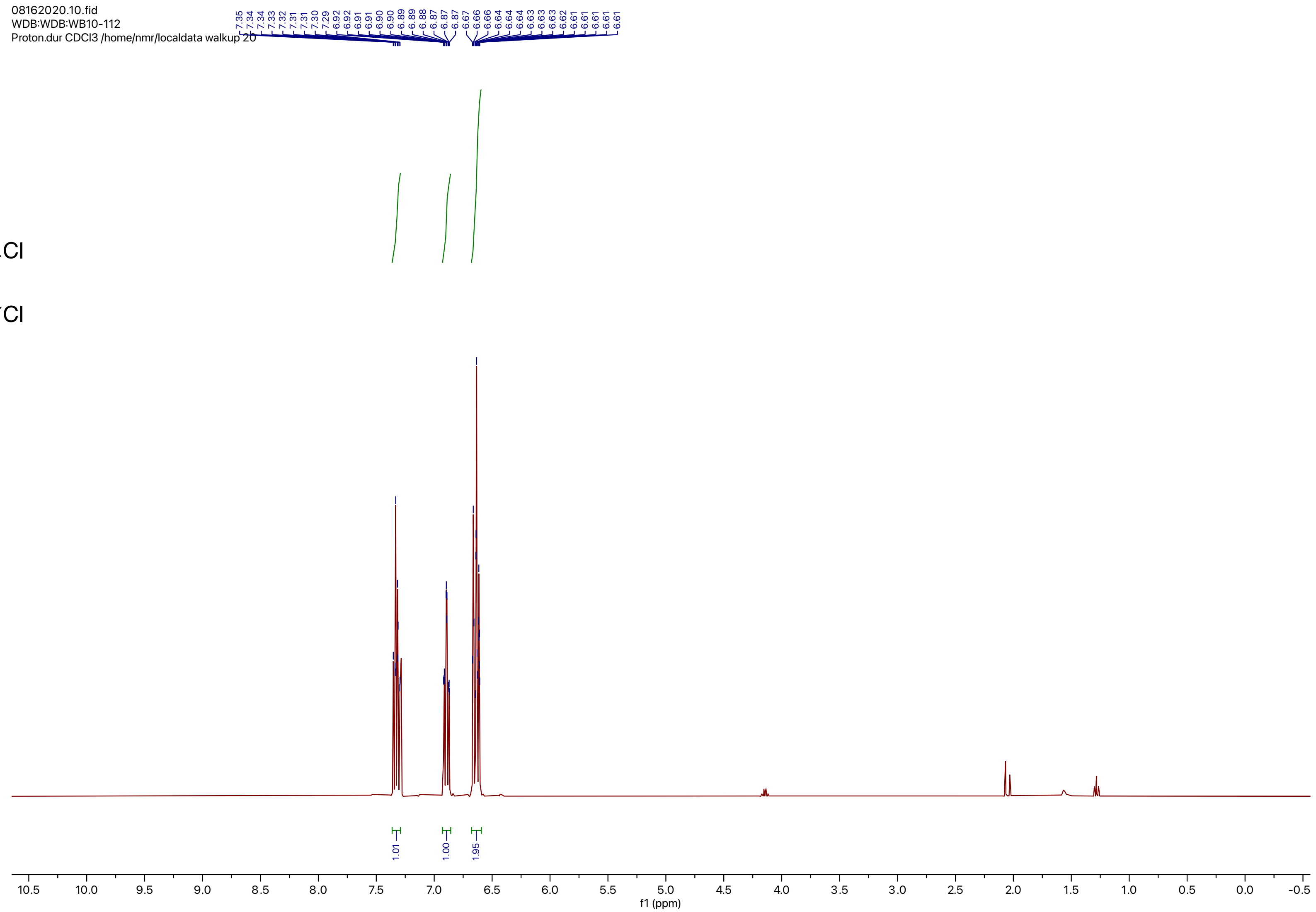

Figure S101. ^{1}H NMR spectrum of **38** recorded at 400 MHz in $CDCl_3$.

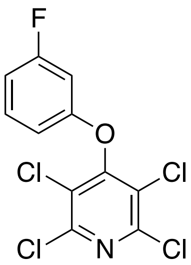

38

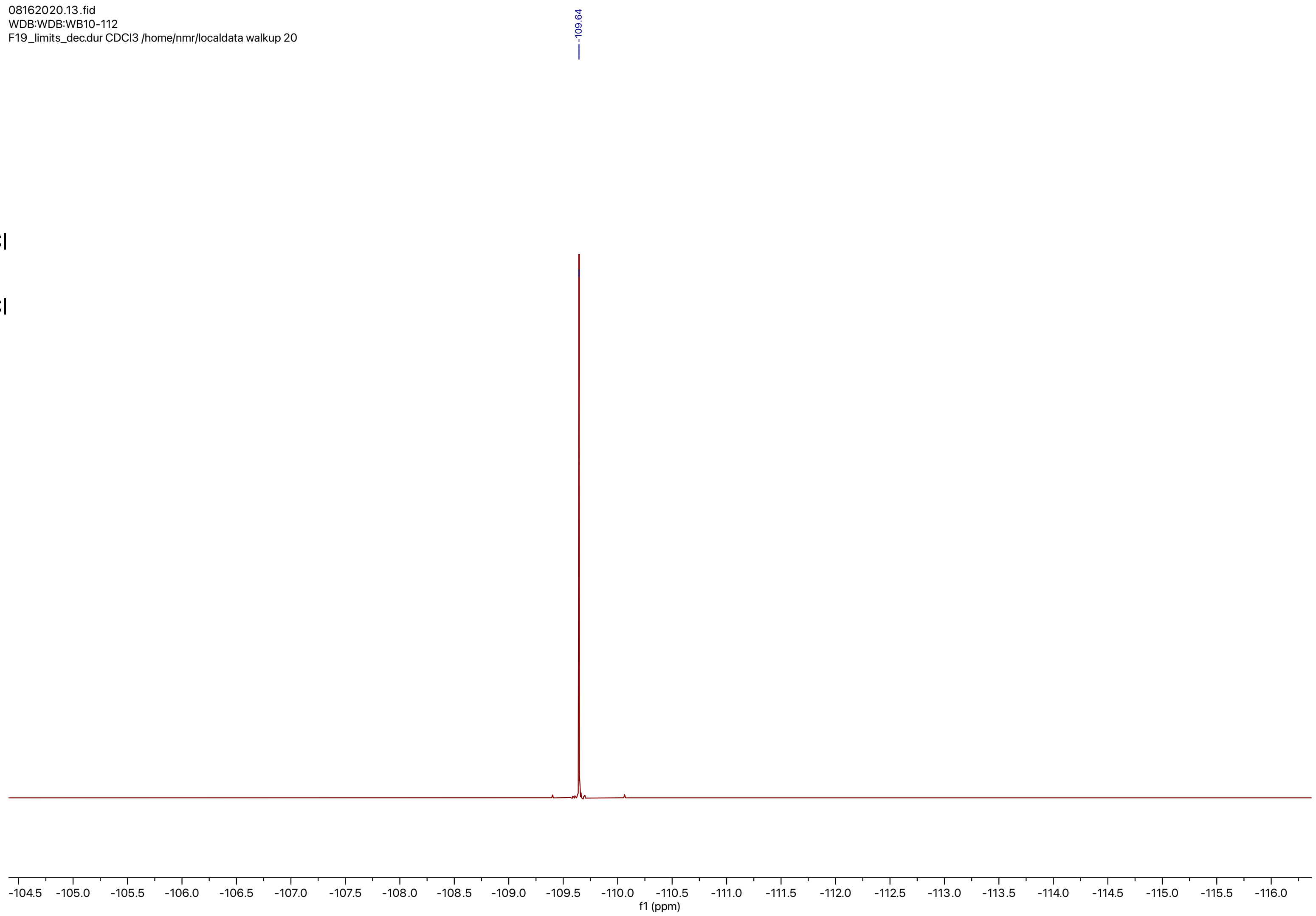

Figure S102. $^{19}F\{^{1}H\}$ NMR spectrum of **38** recorded at 376 MHz in $CDCl_3$.

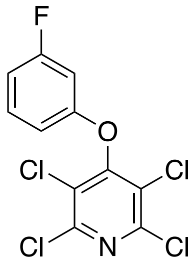

38

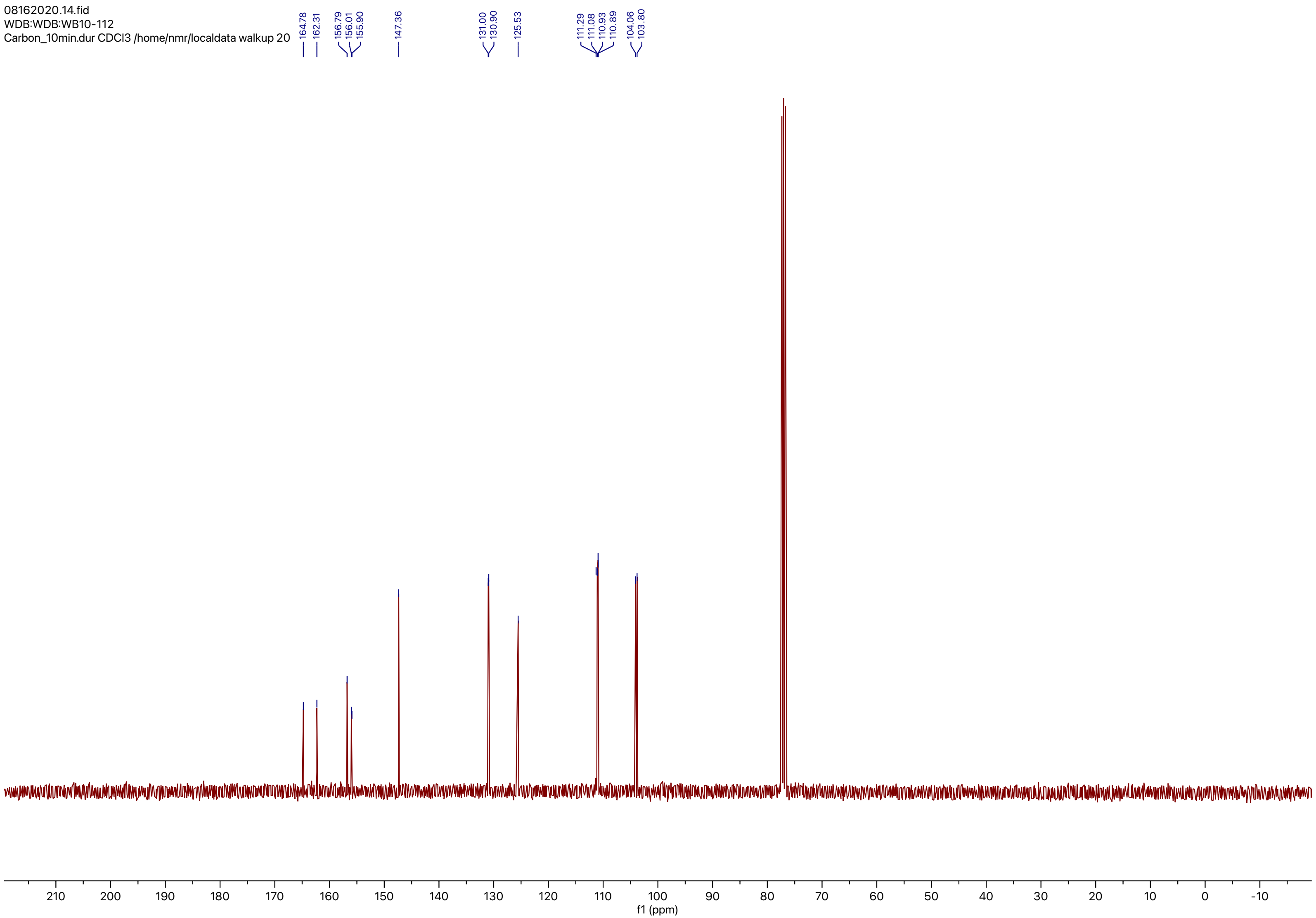

Figure S103. $^{13}C\{^{1}H\}$ NMR spectrum of **38** recorded at 101 MHz in $CDCl_3$.

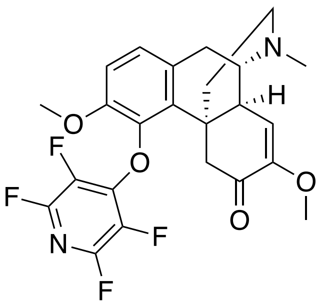

39

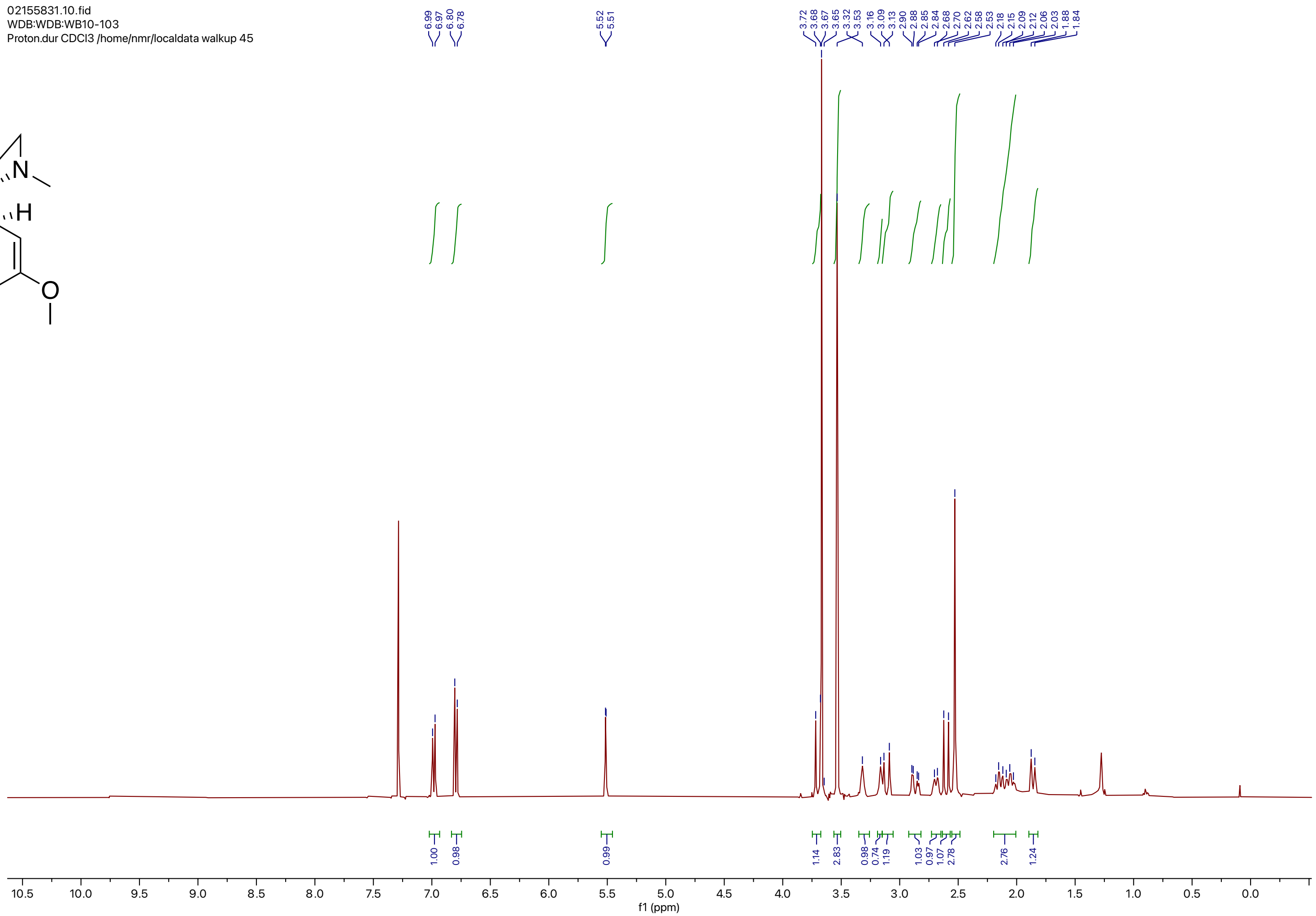

Figure S104. ^{1}H NMR spectrum of **39** recorded at 700 MHz in $CDCl_3$.

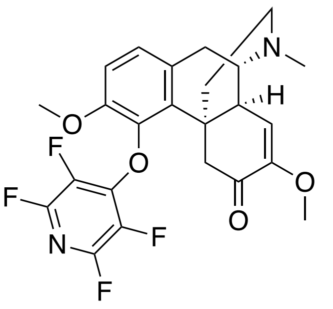

39

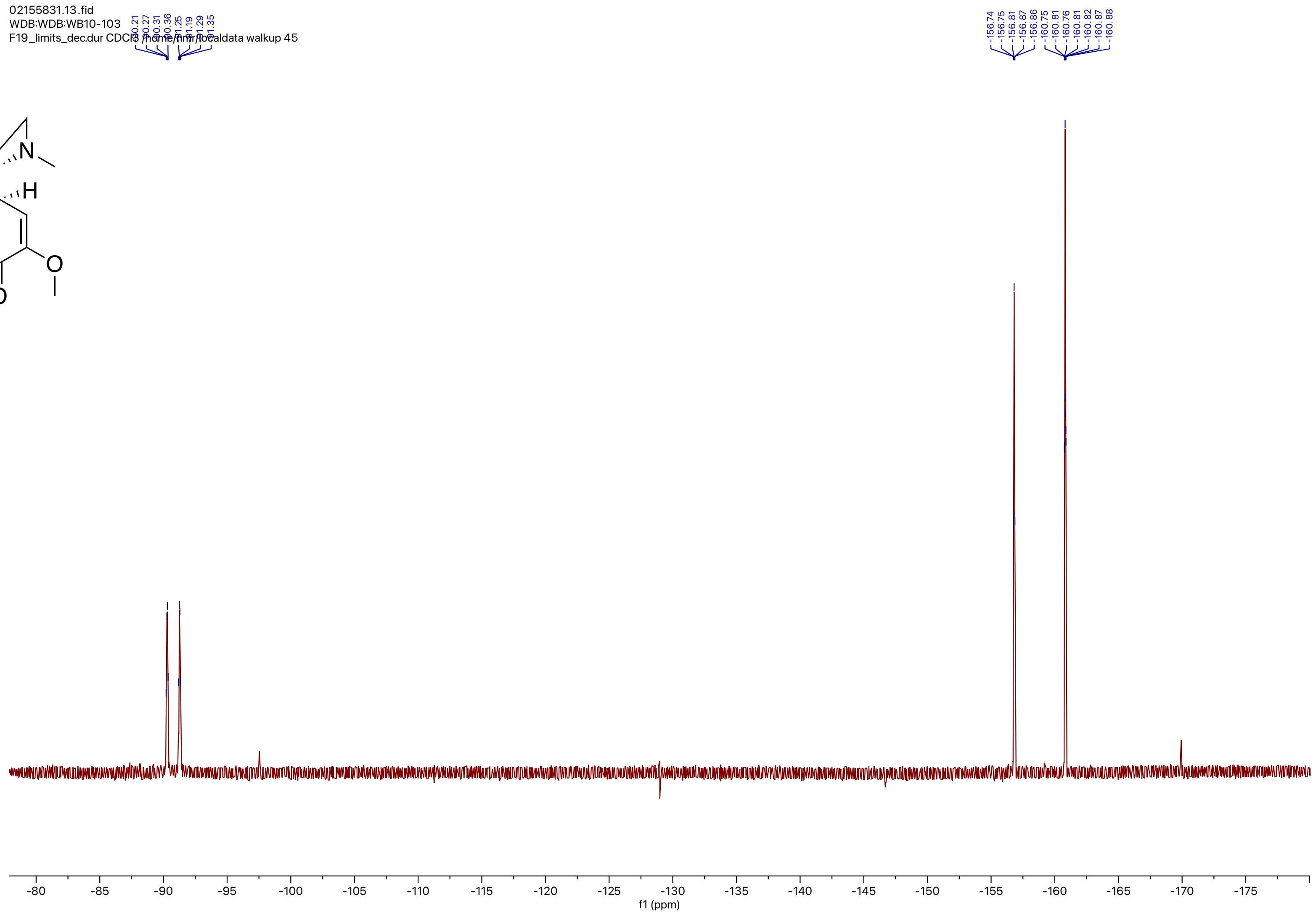

Figure S105. $^{19}F\{^{1}H\}$ NMR spectrum of **39** recorded at 376 MHz in $CDCl_3$.

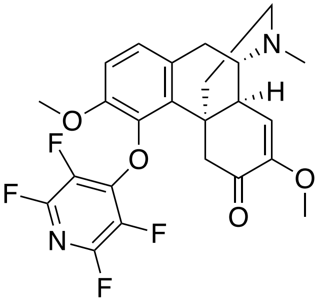

39

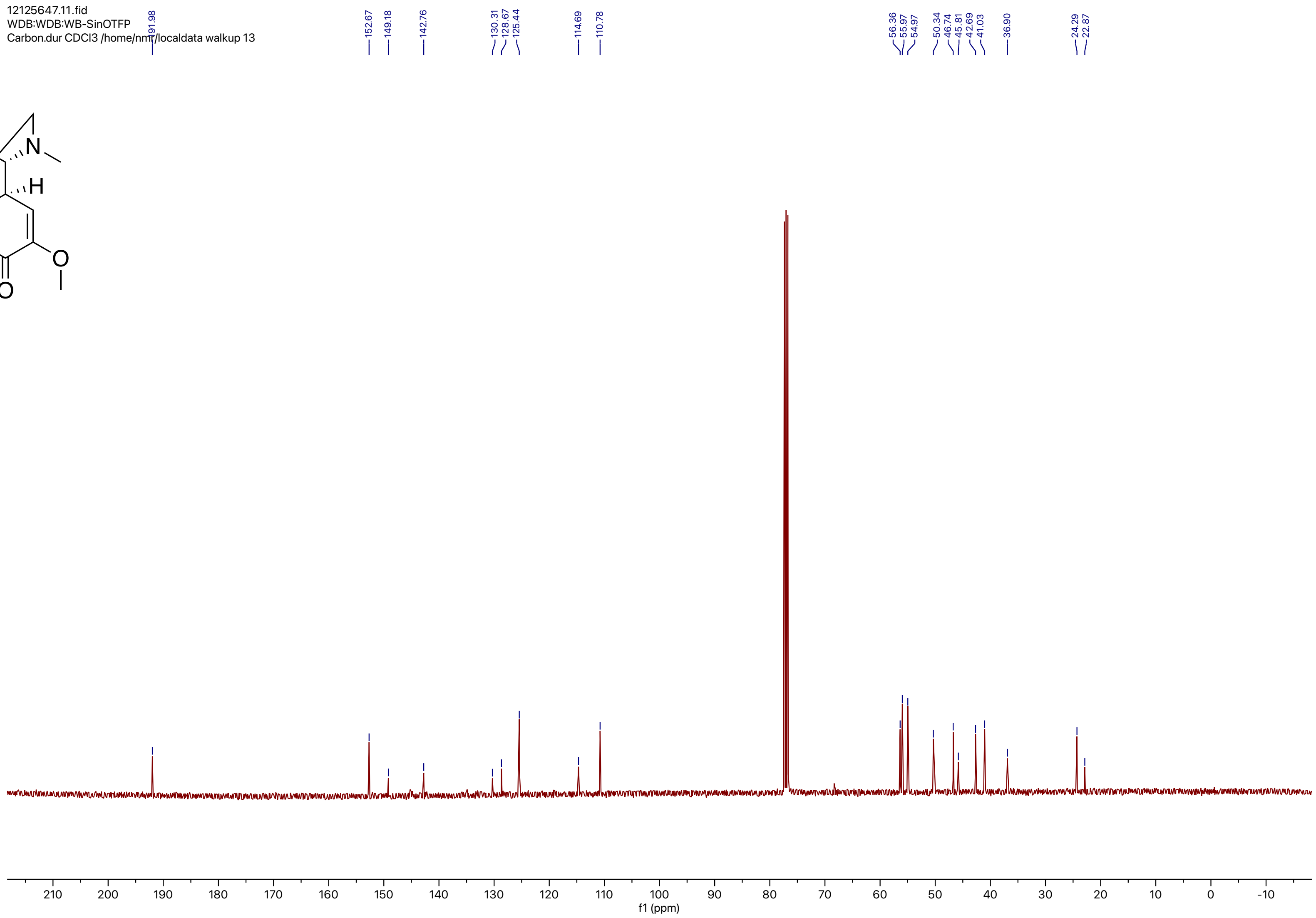

Figure S106. $^{13}C\{^{1}H\}$ NMR spectrum of **39** recorded at 101 MHz in $CDCl_3$.

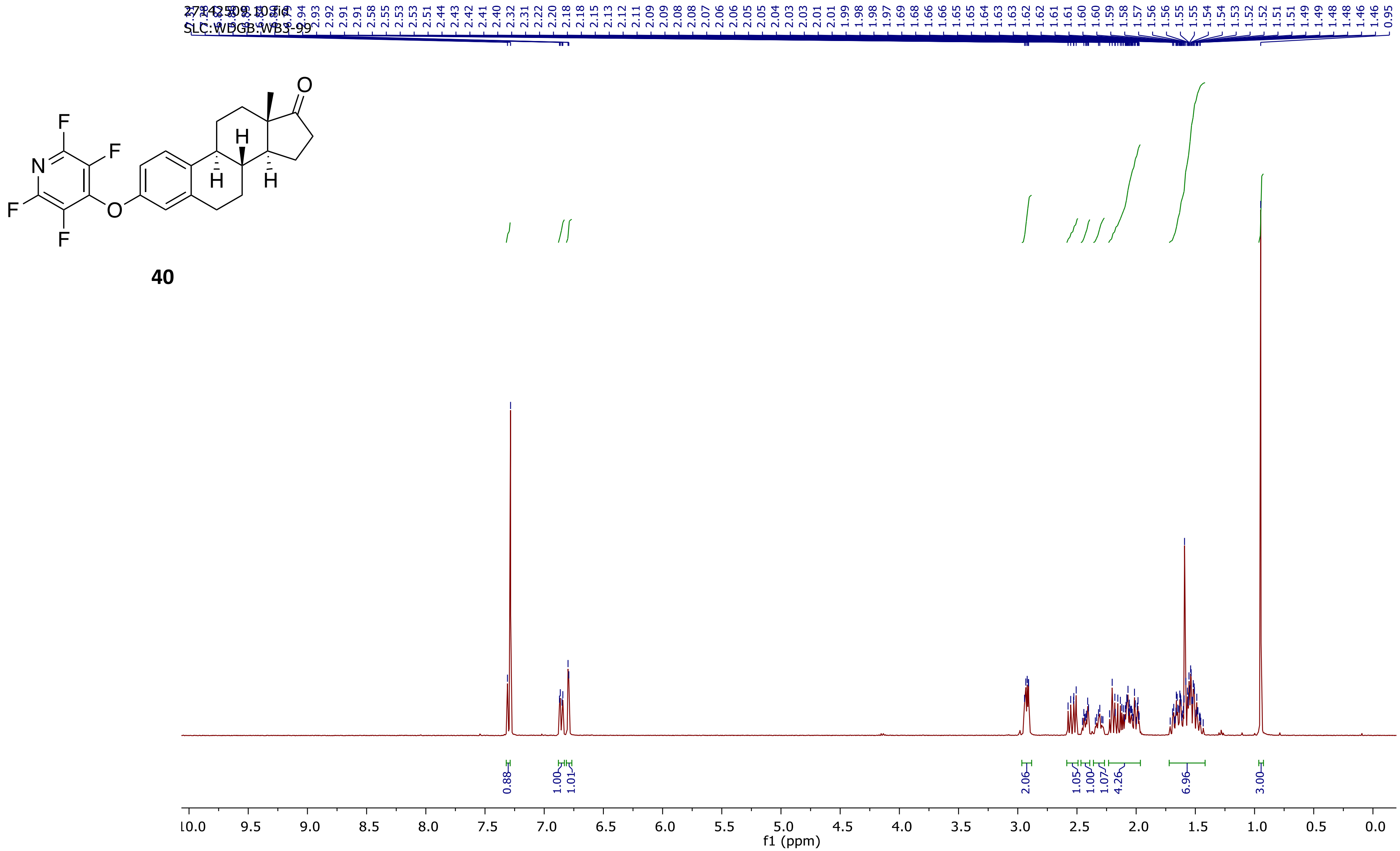

Figure S107. ^{1}H NMR spectrum of **40** recorded at 400 MHz in $CDCl_3$.

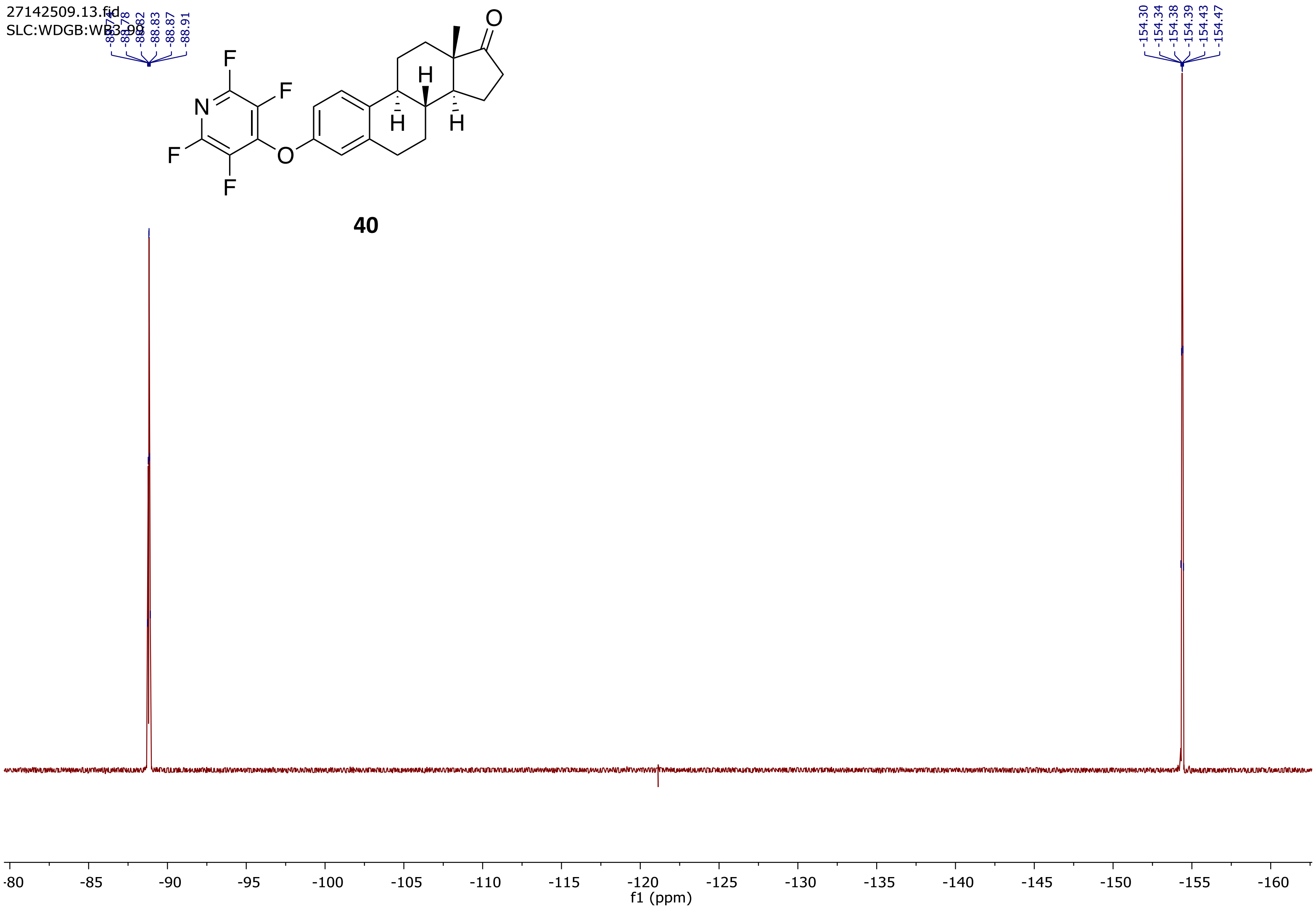

Figure S108. $^{19}F\{^{1}H\}$ NMR spectrum of **40** recorded at 376 MHz in $CDCl_3$.

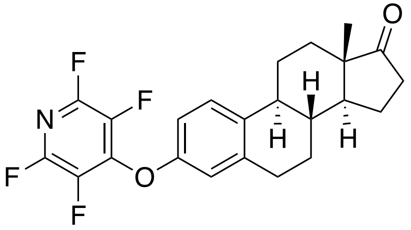

40

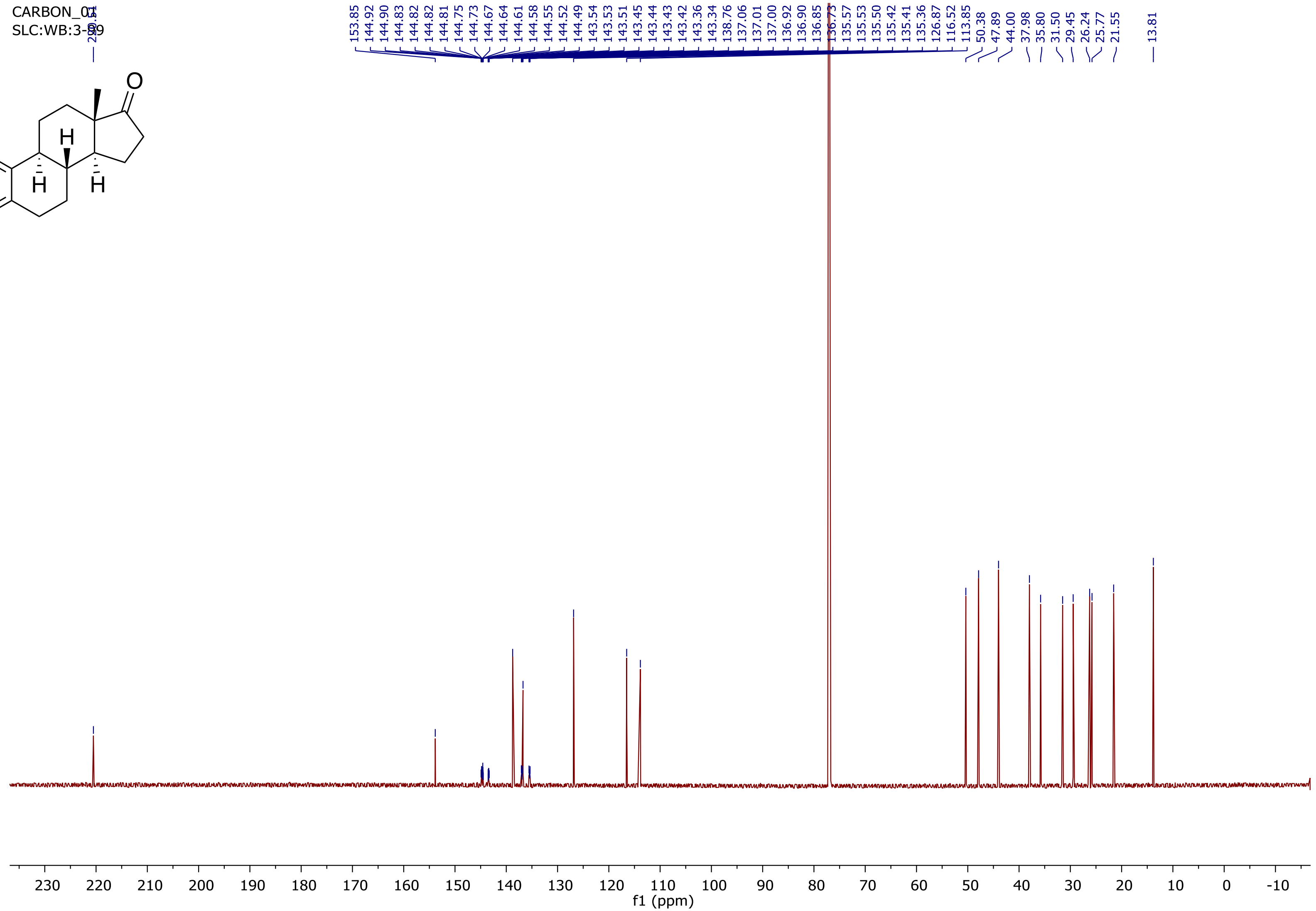

Figure S109. $^{13}C\{^{1}H\}$ NMR spectrum of **40** recorded at 176 MHz in $CDCl_3$.

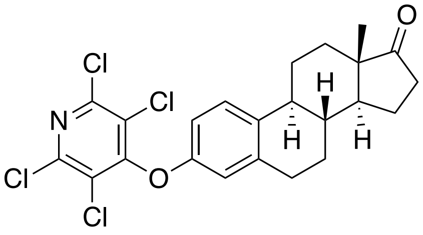

41

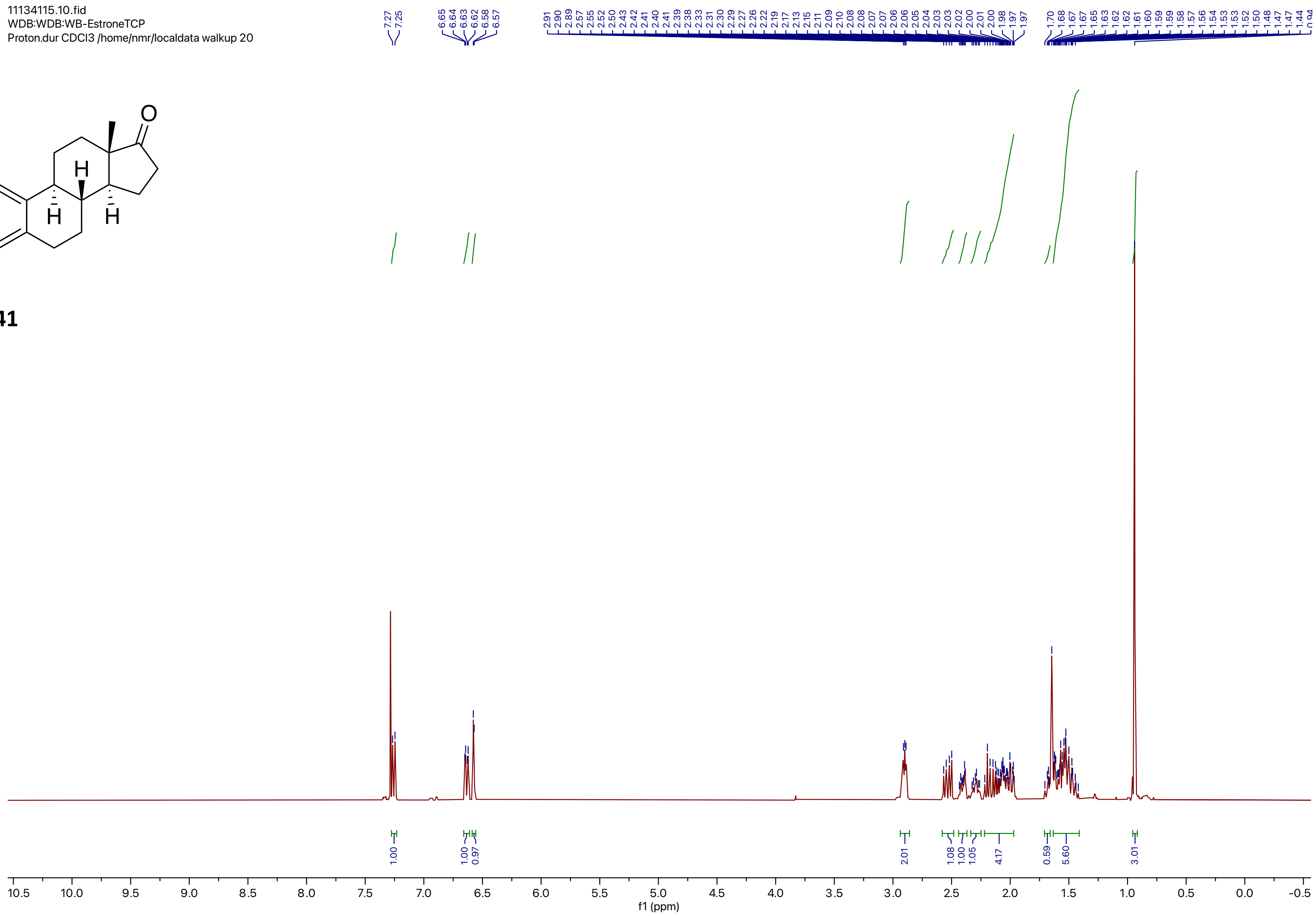

Figure S110. ^{1}H NMR spectrum of **41** recorded at 400 MHz in $CDCl_3$.

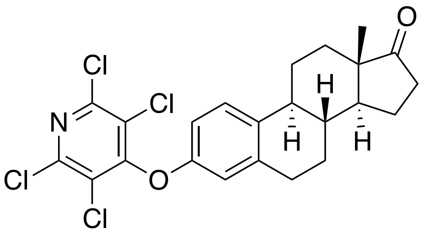

41

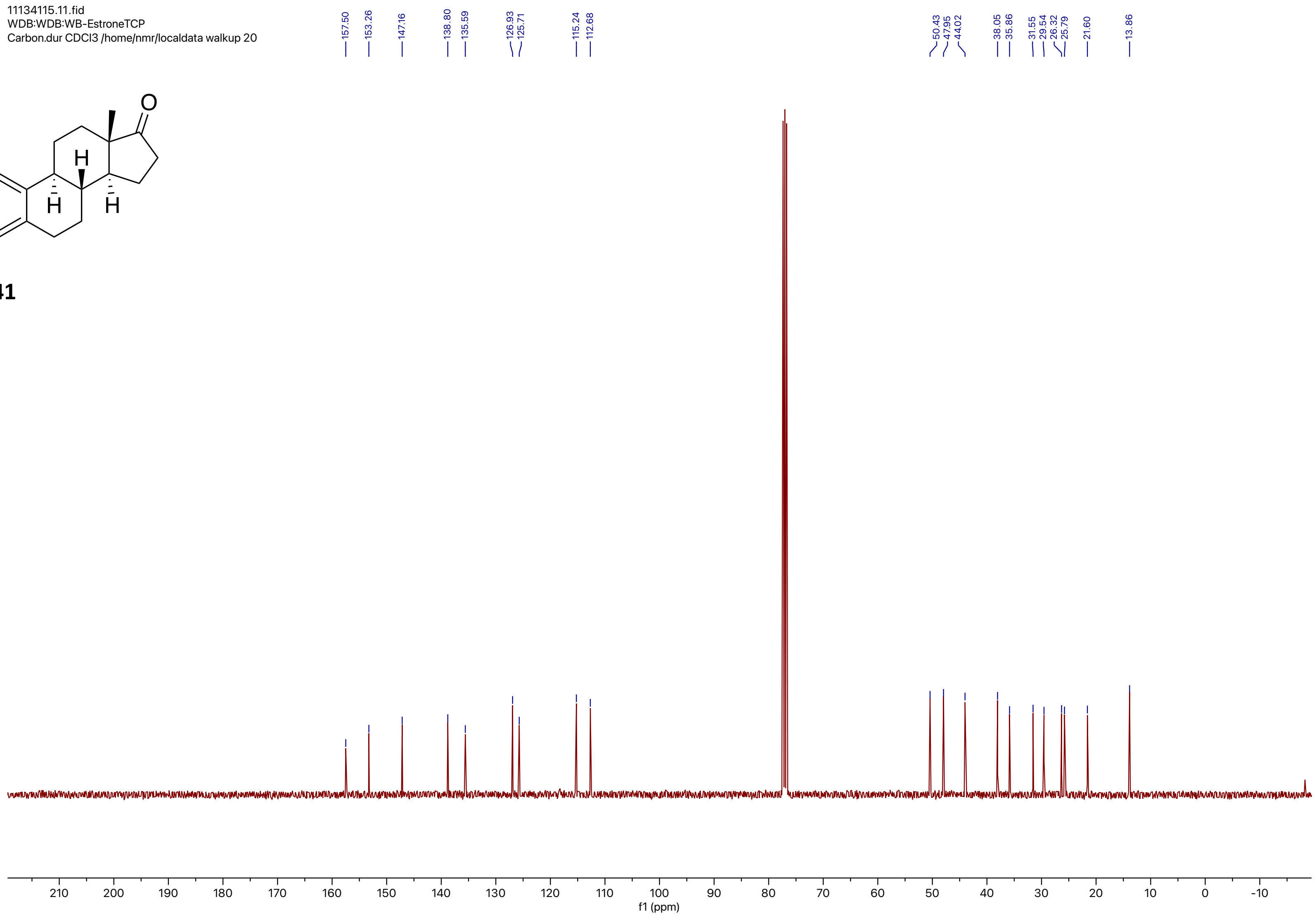

Figure S111. $^{13}C\{^{1}H\}$ NMR spectrum of **41** recorded at 101 MHz in $CDCl_3$.

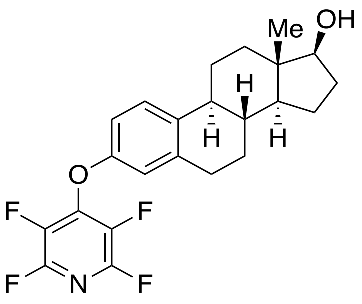

42

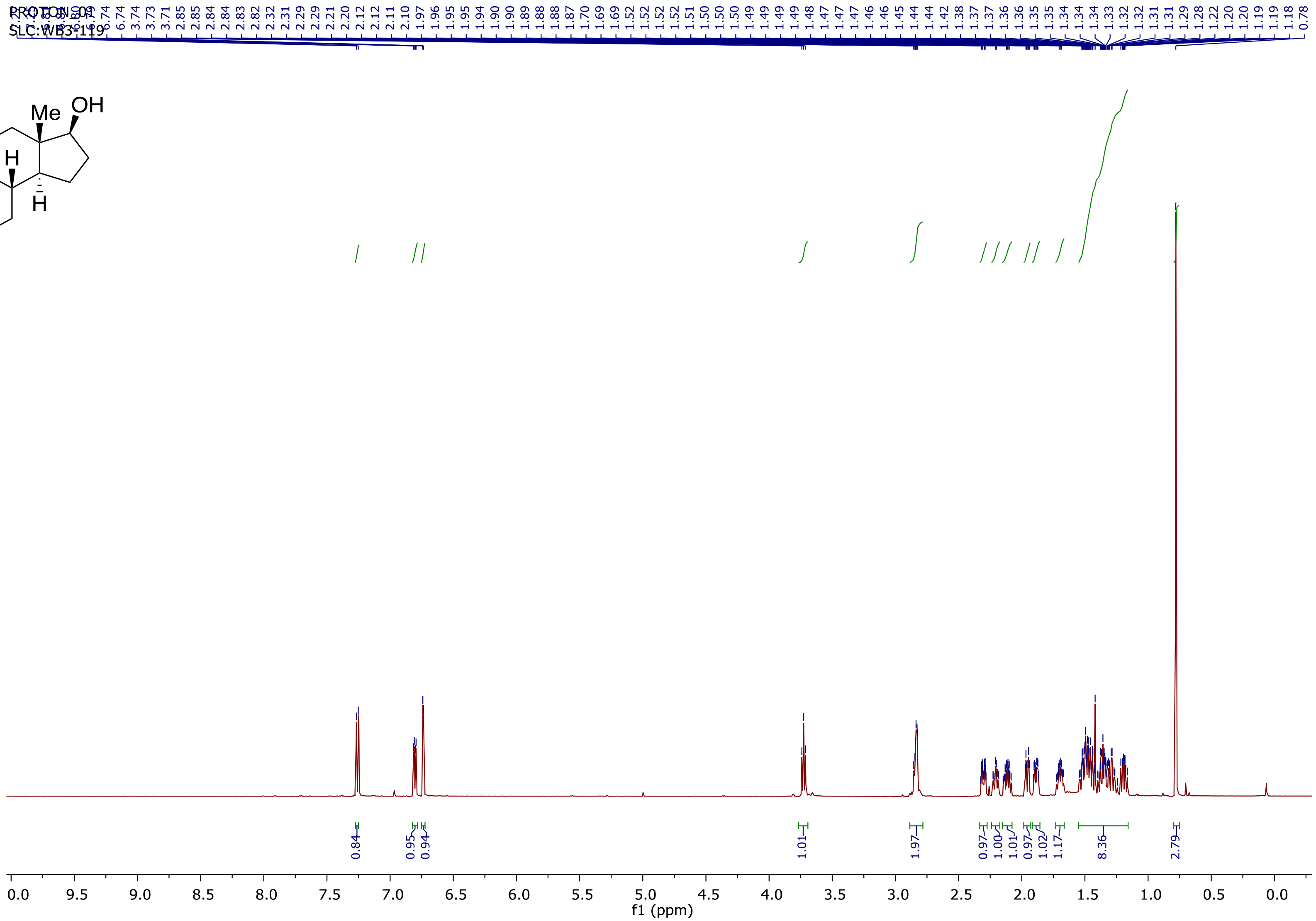

Figure S112. ^{1}H NMR spectrum of **42** recorded at 600 MHz in $CDCl_3$.

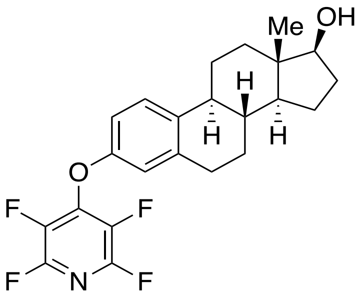

42

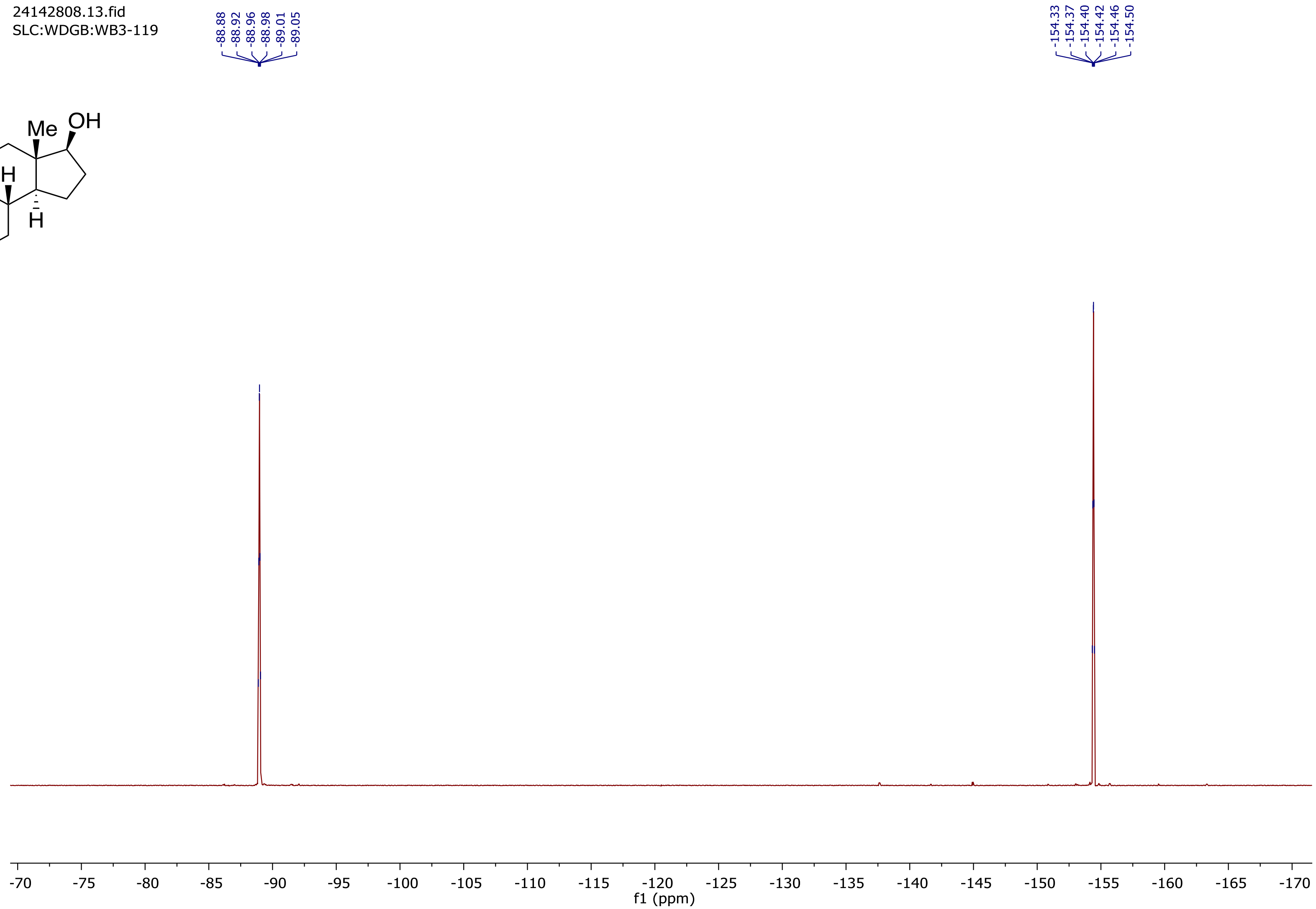

Figure S113. $^{19}F\{^{1}H\}$ NMR spectrum of **42** recorded at 376 MHz in $CDCl_3$.

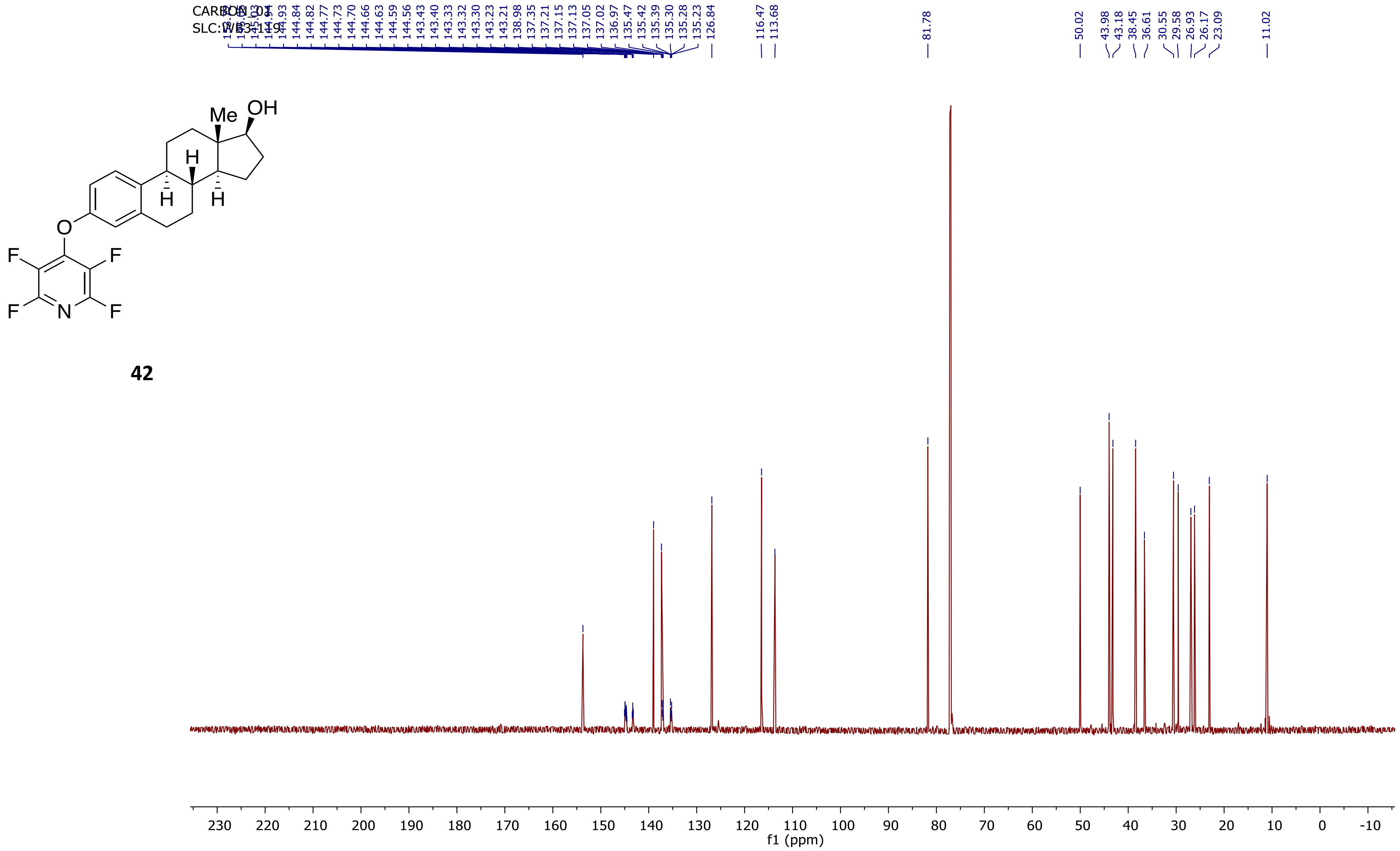

Figure S114. $^{13}C\{^{1}H\}$ NMR spectrum of **42** recorded at 151 MHz in $CDCl_3$.

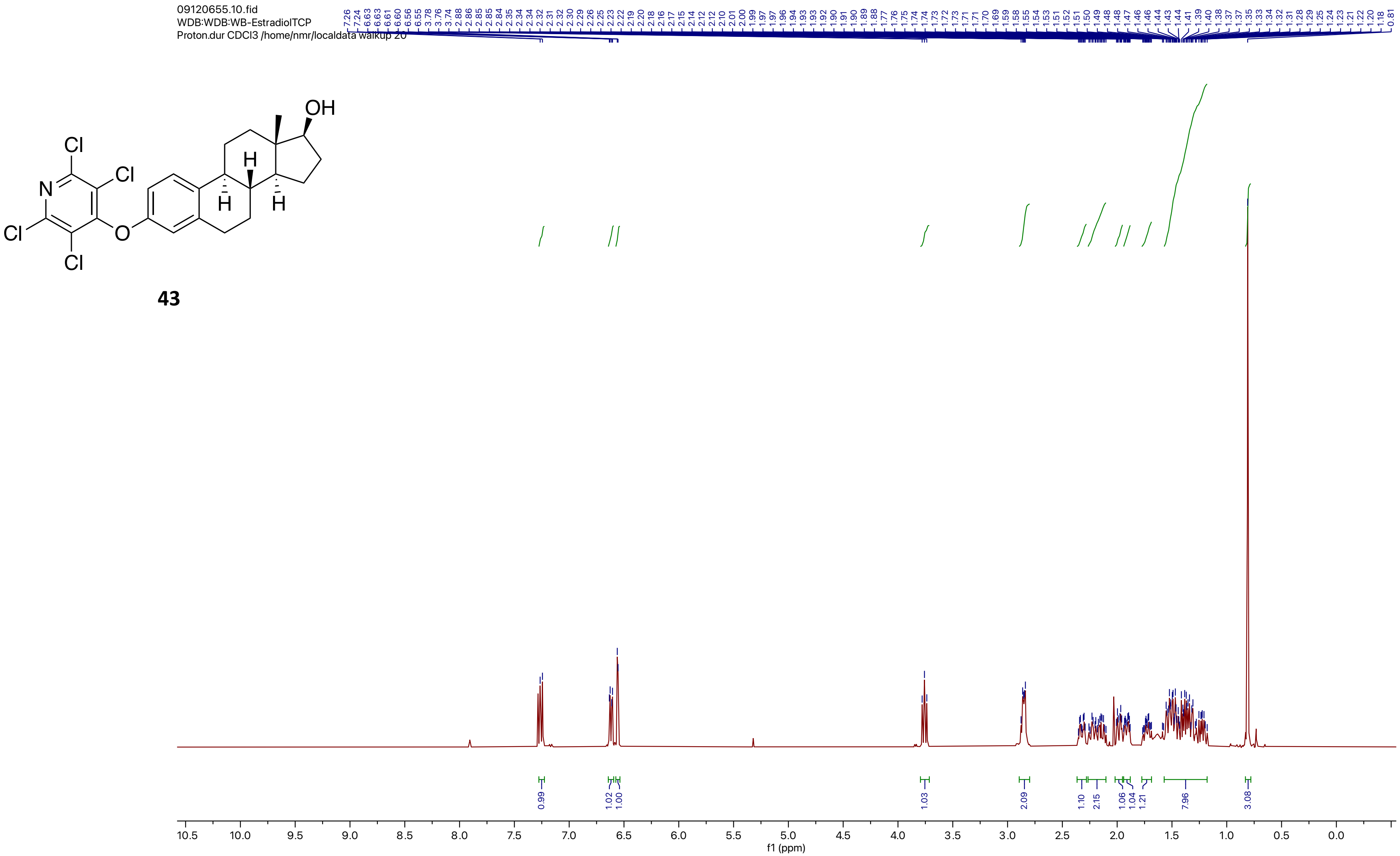

Figure S115. ^{1}H NMR spectrum of **43** recorded at 400 MHz in $CDCl_3$.

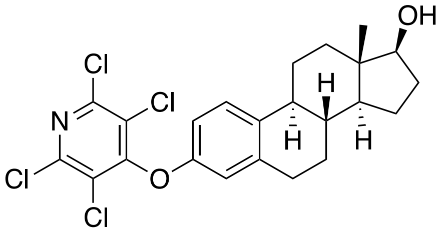

43

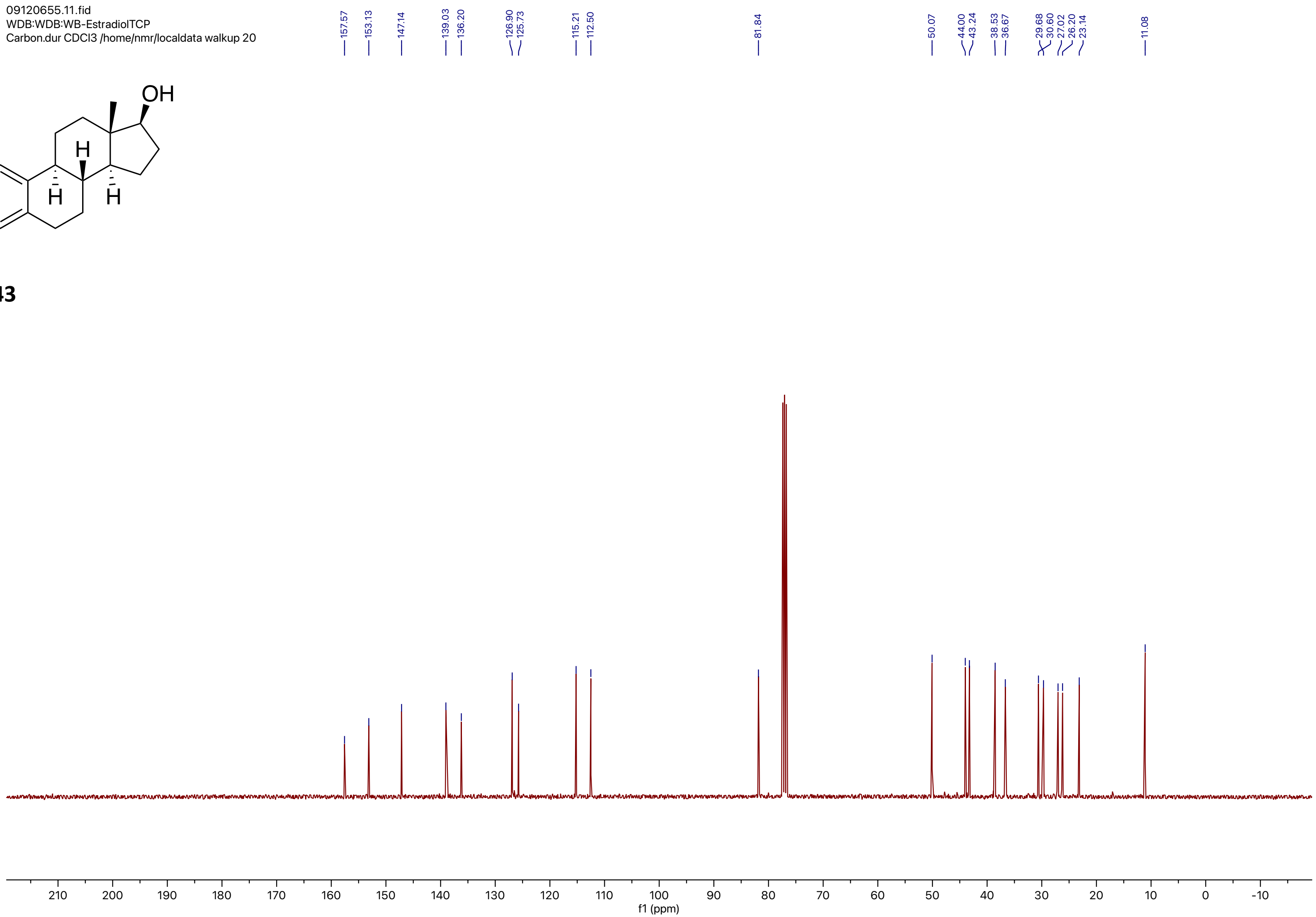

Figure S116. $^{13}C\{^{1}H\}$ NMR spectrum of **43** recorded at 101 MHz in $CDCl_3$.

CSD Search Parameters

CSD version 5.44 (April 2023) was used for the search. Queries were generated using *Conquest*. The search parameters dictated that the crystal structures must be organic, not polymers, single crystals only, R1 < 0.075, no errors and no disorder allowed.

The draw tool was used to search for TFP and TCP containing compounds. The following representations were the structures used in to identify hit compounds:

TFP;

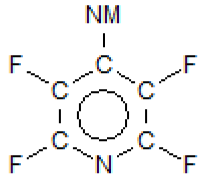

TCP;

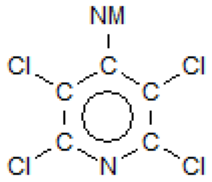

The TFP search returned 169 results and the TCP search returned 16 hits.

Lists of the refcodes for the crystal structures can be found below.

Table S1. TFP derivatized organic compounds in the CSD.

NAME	Publication Year	R-factor	Space Group. Symbol	Z Prime
AFUPUN	2008	3.93	*P*-1	3
AHECEY	2020	4.59	$P2_1$/n	1
AMOCOV	2011	2.14	*P*na2_1	1
APASUG	2011	3.05	$I4_1$cd	1
APILES	2012	1.95	$P2_1$/c	1
APIQAT	2012	4.19	$P2_1$/c	1
APIQEX	2012	3.76	$P2_1$/c	1
AQAKEK	2016	3.68	*P*-1	2
BECFUM	2012	3.4	$P2_1$/c	2
BECGAT	2012	3.61	$P2_1$/c	2
BEJPAK	2017	6.88	*F*dd2	0.5
BELLUD	2022	4.22	$P2_1$/n	1
CALVIV	2002	5.51	*I*2	2
COZXAT	2020	3.52	$P2_1$	1
DATLIV	2012	5.88	*P*nma	0.5
DATLIV01	2013	2.02	*P*nma	0.5
DIXVUB	1985	5.6	$P2_1$/c	1
FAJVER	1987	4.4	*P*nma	0.5
FAQYOP	2022	1.73	*P*-1	1
FEDJAD	2022	6.76	*P*-1	1
FIHJOZ	2023	4.25	$P2_1$/n	1
FIPZIP	2014	3.84	$P2_1$	2
FISJOJ	2019	3.84	$P2_1$/c	1
FISJUP	2019	6.62	$P2_1$/c	2
FOFYIL	2019	2.69	$P4_12_12$	0.5
FOFYOR	2019	1.88	$P4_12_12$	0.5
FOFYUX	2019	1.37	*C*2/c	1.5
FOFZAE	2019	2.05	*C*2/c	0.5
FOFZEI	2019	4.16	*P*-1	1
FOFZIM	2019	1.37	$P2_1$/n	1
FOFZOS	2019	1.9	$P2_12_12_1$	2
FOFZUY	2019	3.76	$P2_12_12_1$	2
FOGBAH	2019	2.89	$P2_12_12_1$	1
FOGDOX	2019	1.6	$P2_12_12_1$	1
GEQDUF	2022	4.47	*P*-1	1
GOFCEL	2014	5.27	$P2_1$/c	1
GOFCIP	2014	4.09	$P2_12_12$	1
GUGRUY	2020	3.26	*C*c	1
GUGRUY01	2022	6.42	*C*c	1
GUGSEJ	2020	4.2	$P2_1$/n	1
GUGSUZ	2020	4.79	*C*2/c	1
HICBAW	1995	2.5	*P*-1	2
HILJAQ	2019	2.85	*P*-1	1
HIQYEL	1999	4.6	*P*-1	1

HUBWIK	2000	5.74	$P2_1/c$	1
HUXWIJ	2020	3.97	$P2_1/c$	1
ITUCUY	2021	3.07	$I2/a$	1
IWENEF	2016	2.45	$P2_1/n$	1
IWEPAD	2016	3.07	$Pna2_1$	1
JAFLIO	2016	2.93	P-1	1
JAFXEW	2016	2.42	$P2_1/c$	1
JAFXUM	2016	3.63	$P2_1/c$	1
JIFVAY	2018	3.39	$C2/c$	0.5
JIFVEC	2018	3.8	$C2/c$	0.5
JUTBUY	2020	2.94	$P2_1/c$	1
JUTCAF	2020	1.63	$P2_1$	1
KAMTUO	2004	3.51	$Pbca$	0.5
KECCED	2017	4.39	$I4_1/acd$	0.5
KECNOY	2017	4.39	$P2_1/c$	1
KECNUE	2017	3.96	$C2/c$	2
KEDWEW	2006	4.53	$P2_1/c$	1
KEDWIA	2006	3.18	$P2_1/c$	1
KIKVUV	1991	4.2	$P2_1/n$	0.5
LAHBEC	2004	6.67	$P2_1/c$	0.5
LAHBUS	2004	3.65	$P2_12_12_1$	1
LAKDOU	2020	4.31	P-1	1
LUJYEU	2001	5.77	$Pbca$	1
MOJFUP	2019	4.47	P-1	2
MOJGAW	2019	3.81	$P2_1/n$	1
MOYJAN	2015	3.53	$P2_1/n$	1
NALJAP	2019	4.56	$C2/c$	0.5
NEMHAO	2001	5.2	$C2/c$	1
NEMHES	2001	5.27	$P112_1/b$	4
NEPTUB	2022	3	P-1	1
NIXMIR	2008	3.94	$P2_1/c$	1
NUJQEQ	2015	5.12	$C2/c$	1
NUJQIU	2015	3.87	$P2_1/n$	1
NUMVUO	2011	2.89	$P2_1$	1
NUTPAW	2020	3.38	$P2_12_12_1$	1
NUZPOO	2010	7.37	P-1	1
ODAWIC	2016	4.9	P-1	2
OFOLIG	2012	4.79	$P2_1/n$	1
OMUMAN	2021	4. 4_1	P-1	1
OXUTOT	2021	5. 2_1	P-1	0.5
PAJCAG	2012	5.32	$P2_1/n$	1
PALPOI	2005	4.61	P-1	2
PALPUO	2005	6.54	$Pnma$	0.5
PALQAV	2005	4.7	$P2_1/n$	1
PALQEZ	2005	3.72	P-1	1
PAXCUQ	2022	4. 2_1	$P1$	1
PUBGOI	1998	6.55	$P2_1/n$	2
PUXYUE	2016	3.99	$P2_1$	4

RARFUO	2017	3.89	$P2_1/c$	1
RARGOJ	2017	3.73	$P2_1$	2
RITNOY02	2013	2.95	$P2_1/n$	2
RUSXEL	2020	4.31	$P2_1/c$	2
RUSXIP	2020	4.22	*P*bca	1
RUXKIE	2002	5.68	$P2_1/c$	1
SEYTIB	2013	3.63	*P*-1	2
SEYTOH	2013	4.9	$P2_1/c$	1
SIXYIH	1991	4.9	*P*-1	1
SULTAV	2009	2.86	*P*-1	1
TACWIE	2003	5.17	$P2_1/c$	2
TAPRAD	1992	4.8	$P2_1/n$	1
TIDJID	2023	6.93	$P2_1/c$	1
TUHJIR	2015	6.33	$P2_1/n$	1
TUHJOX	2015	4.48	*P*bca	0.5
TUHJOX01	2015	5	*P*bca	0.5
TUHJOX02	2020	3.34	*P*bca	0.5
TUMWAC	2020	4.01	*P*-1	2
UDUXEY	2009	3.46	$P2_1/n$	1
UPULOH	2011	4.88	*P*-1	2
UPULUN	2011	5.52	*P*bca	1
URAFIF	2021	4.25	*P*bca	2
VEPXOG	2018	4.16	$P2_1/c$	1
VOCPEL	2019	3.91	*P*-1	1
WASBAU	2005	4.16	*C*2/c	0.5
WEXMUK	2018	6.46	$P2_1/c$	1
WOLPEV	2019	2.84	$P2_12_12_1$	1
WOLPIZ	2019	3.05	$P2_1/n$	1
WOLPOF	2019	3.06	$P2_12_12_1$	1
WOLPUL	2019	3.05	$P2_1$	1
WOLQAS	2019	3.57	*P*bcn	0.5
XABBAH	2020	3.94	*P*bca	1
XABBEL	2020	5.85	*P*-1	1
XABBOV	2020	4.43	$P2_1/c$	1
XABCEM	2020	6. 4_1	$Pna2_1$	1
XABCOW	2020	4.47	$P2_1/c$	2
XEKWAN	2012	3.65	*P*-1	2
XELBIB	2012	6.48	*P*-1	3
XUTLOQ	2020	3.52	$Pna2_1$	1
XUTLUW	2020	3.45	$P2_1/n$	1
XUTMAD	2020	3.23	*P*-1	2
XUTMAD01	2020	5.12	*C*2/c	0.5
XUTMIL	2020	3.33	$P32_12$	1
XUTMOR	2020	3.47	*P*-1	1
XUTMUX	2020	3.28	*P*-1	2
XUTNAE	2020	2.66	$C222_1$	0.5
XUTNIM	2020	3.8	*P*-1	2
XUTNOS	2020	3.18	$P2_1/n$	1

XUTPEK	2020	4.71	$P2_1/c$	1
XUZWUN	2020	6.55	$P2_1/c$	1
XUZXAU	2020	6.85	$P2_1$	2
XUZXEY	2020	5.51	$P2_1/c$	1
XUZXIC	2020	3.67	$Fdd2$	1
XUZXOI	2020	4.12	$P2_1/c$	1
XUZXUO	2020	6	$P2_1/c$	2
XUZYAV	2020	4.96	P-1	1
XUZYEZ	2020	6.56	$P2_1/c$	1
XUZYID	2020	6.2	P-1	1
XUZYOJ	2020	3.57	$P2_1/n$	1
XUZYOJ01	2020	7.26	$P2_1/n$	1
XUZZAW	2020	3.97	$P2_1/n$	1
XUZZEA	2020	7.15	$P2_1/c$	1
XUZZIE	2020	6.56	$P2_1/c$	1
XUZZOK	2020	3.43	$P2_1/n$	1
XUZZUQ	2020	3.56	$P2_1/c$	1
YAKXUH	2021	3.57	$P4_12_12$	0.5
YAKZET	2021	3.36	$Pbcn$	0.5
YAVFOR	2005	5.81	P-1	1
YAVFUX	2005	6.74	$P2_1/n$	1
YAYZUX	2022	1.99	$P2_12_12_1$	1
YIMNIT	2013	2.37	$Pcab$	2
ZADVAB	1995	2.61	$I2/a$	1
ZADVAB01	2016	1.33	$C2/c$	1
ZECGIZ	2012	5.56	$C2/c$	1
ZENZAX	2022	2.83	$P2_12_12_1$	1
ZESBIK	2013	3.58	P-1	1
ZUFTEB	2014	3.89	$Pca2_1$	2

Table S2. TCP derivatized organic compounds in the CSD.

NAME	Publication Year	R-factor	Space Group. Symbol	Z Prime
NIDMUM	2023	3.94	$P2_1$/c	0.5
DEXXAI	2018	3.43	Pna2_1	1
EDEGIE	2006	2.07	P-1	1
MESQOT	2018	3.16	$P2_1$/c	1
OMUMER	2021	4.27	$P2_1$/n	1
PCLPYR	1973	5.9	$P4_12_12$	0.5
PCLPYR03	2015	3.69	Pc	1
PCLPYR04	2015	4.9	Pc	1
PCLPYR05	2015	4.74	Pc	1
PCLPYR06	2015	5.25	Pc	1
PCLPYR07	2015	3.88	Pc	1
QIVSIA	2019	4.99	$P2_1$/c	1
RAVVEP	1997	4.64	$P2_1$/c	1
TCHYPY	1972	6.5	Pbca	1
TESHUX	2017	4.4	P-1	1
XUGXUU	2015	2.78	$P2_1$/n	1

Crystallography

Single Crystal X-ray Diffraction Experiments

X-ray single crystal data was collected using λMoKα radiation (λ = 0.71073 Å) or λCuKα (λ = 1.5406 Å) on an *Agilent* XCalibur (Sapphire-3 CCD detector, fine-focus sealed tube, graphite monochromator) or *Bruker* D8 Venture (Photon100 CMOS detector or Photon III MM C7/C14 CPAD detector, IμSmicrosource, focusing mirrors). Diffractometers are equipped with a *Cryostream* (Oxford Cryosystems) open-flow nitrogen cryostats at a temperature of 120.0 K. All structures were solved by direct methods and refined by full-matrix least squares on F2 for all data using Olex2[3] and SHELXTL[4][5] software. All non-disordered non-hydrogen atoms were refined anisotropically, the hydrogen atoms were placed in the calculated positions and refined in riding mode unless otherwise specified. Crystallographic data and related CIFs for the structures **1**-**5**, **7**,**8**, **10**-**26**, **28**, **30**-**33** and **35**-**43** have been deposited with the joint Cambridge Crystallographic Data Centre and Fachinformationszentrum Karlsruhe Access Structures service and are available free of charge with the following deposition numbers: CCDC-2392089–2392126.

Crystal Renders

Renders of the crystal structures depicted in figures were made in *VMD version 1.9.3*.

Crystallographic Data

Crystal structure: **1**

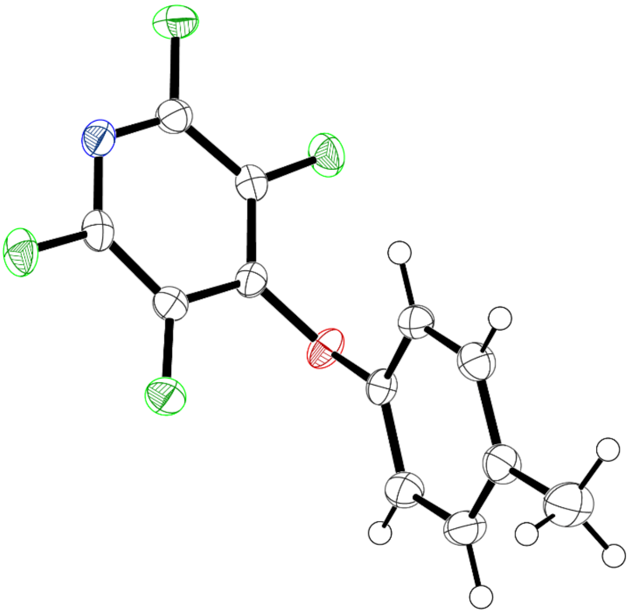

Empirical formula	$C_{12}H_7F_4NO$
Formula weight	257.19
Temperature/K	120.00
Crystal system	orthorhombic
Space group	$P2_12_12_1$
a/Å	5.0569(3)
b/Å	10.1934(6)
c/Å	21.1522(13)
α/°	90
β/°	90
γ/°	90
Volume/Å^3	1090.33(11)
Z	4
ρ_{calc}/cm^3	1.567
μ/mm^{-1}	0.147
F(000)	520.0
Crystal size/mm^3	0.45 × 0.19 × 0.15
Radiation	MoKα (λ = 0.71073)
2Θ range for data collection/°	4.436 to 59.98
Index ranges	-7 ≤ h ≤ 7, -14 ≤ k ≤ 14, -29 ≤ l ≤ 29
Reflections collected	18566
Independent reflections	3168 [R_{int} = 0.0343, R_{sigma} = 0.0245]
Data/restraints/parameters	3168/0/164
Goodness-of-fit on F^2	1.024
Final R indexes [I>=2σ (I)]	R1 = 0.0355, wR2 = 0.0971
Final R indexes [all data]	R1 = 0.0404, wR2 = 0.1007
Largest diff. peak/hole / e Å^{-3}	0.19/-0.16
Flack parameter	-0.05(19)
CCDC Number	2392089

Crystal structure: **2**

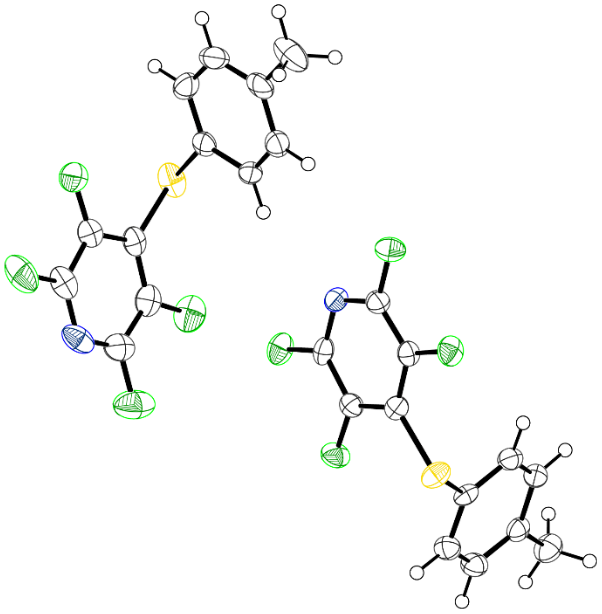

Empirical formula	$C_{12}H_7F_4NS$
Formula weight	273.25
Temperature/K	120.00
Crystal system	monoclinic
Space group	$P2_1$
a/Å	12.8721(9)
b/Å	4.9297(3)
c/Å	18.1417(12)
α/°	90
β/°	99.559(2)
γ/°	90
Volume/Å^3	1135.21(13)
Z	4
ρ_{calc}/cm^3	1.599
μ/mm^{-1}	0.317
F(000)	552.0
Crystal size/mm^3	0.154 × 0.09 × 0.023
Radiation	Mo Kα (λ = 0.71073)
2Θ range for data collection/°	4.232 to 61.048
Index ranges	-18 ≤ h ≤ 18, -7 ≤ k ≤ 7, -25 ≤ l ≤ 25
Reflections collected	25222
Independent reflections	6905 [R_{int} = 0.0618, R_{sigma} = 0.0740]
Data/restraints/parameters	6905/1/327
Goodness-of-fit on F^2	1.011
Final R indexes [I>=2σ (I)]	R1 = 0.0504, wR2 = 0.0905
Final R indexes [all data]	R1 = 0.0885, wR2 = 0.1020
Largest diff. peak/hole / e Å^{-3}	0.30/-0.29
Flack parameter	0.01(5)
CCDC Number	2392090

Crystal structure: **3**

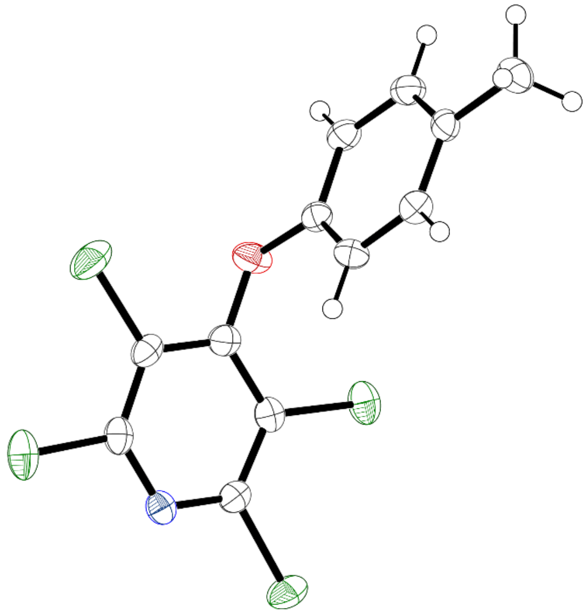

Empirical formula	$C_{12}H_7Cl_4NO$
Formula weight	322.99
Temperature/K	120.00
Crystal system	triclinic
Space group	P-1
a/Å	7.0232(8)
b/Å	8.9076(10)
c/Å	11.1467(13)
α/°	98.465(4)
β/°	104.067(4)
γ/°	98.377(4)
Volume/Å^3	656.98(13)
Z	2
ρ_{calc}/cm^3	1.633
μ/mm^{-1}	0.885
F(000)	324.0
Crystal size/mm^3	0.14 × 0.11 × 0.07
Radiation	MoKα (λ = 0.71073)
2Θ range for data collection/°	4.708 to 59.996
Index ranges	-9 ≤ h ≤ 9, -12 ≤ k ≤ 12, -15 ≤ l ≤ 15
Reflections collected	10998
Independent reflections	3818 [R_{int} = 0.0388, R_{sigma} = 0.0516]
Data/restraints/parameters	3818/0/164
Goodness-of-fit on F^2	1.042
Final R indexes [I>=2σ (I)]	R_1 = 0.0463, wR_2 = 0.0862
Final R indexes [all data]	R_1 = 0.0737, wR_2 = 0.0953
Largest diff. peak/hole / e Å^{-3}	0.31/-0.37
CCDC Number	2392091

Crystal structure: **4**

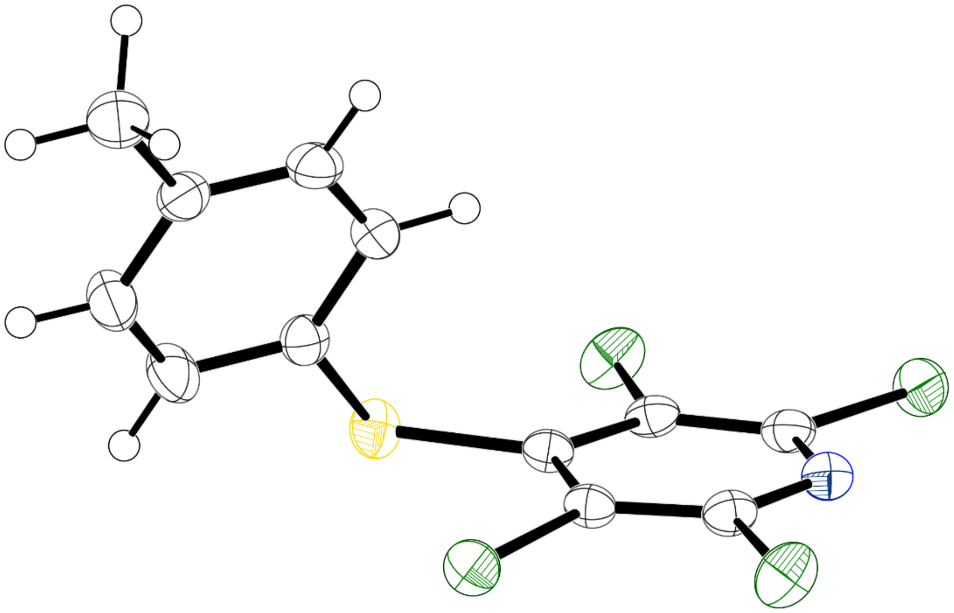

Empirical formula	$C_{12}H_7Cl_4NS$
Formula weight	339.05
Temperature/K	120.00
Crystal system	triclinic
Space group	P-1
a/Å	7.5828(4)
b/Å	8.5095(5)
c/Å	11.1016(6)
α/°	99.980(2)
β/°	99.362(2)
γ/°	100.354(2)
Volume/Å^3	679.98(7)
Z	2
ρ_{calc}/cm^3	1.656
μ/mm^{-1}	1.002
F(000)	340.0
Crystal size/mm^3	0.17 × 0.15 × 0.09
Radiation	MoKα (λ = 0.71073)
2Θ range for data collection/°	4.976 to 59.998
Index ranges	-10 ≤ h ≤ 10, -11 ≤ k ≤ 11, -15 ≤ l ≤ 15
Reflections collected	11754
Independent reflections	3956 [R_{int} = 0.0309, R_{sigma} = 0.0372]
Data/restraints/parameters	3956/0/164
Goodness-of-fit on F^2	1.105
Final R indexes [I>=2σ (I)]	R_1 = 0.0466, wR_2 = 0.0994
Final R indexes [all data]	R_1 = 0.0593, wR_2 = 0.1049
Largest diff. peak/hole / e Å^{-3}	0.85/-0.63
CCDC Number	2392092

Crystal structure: **5**

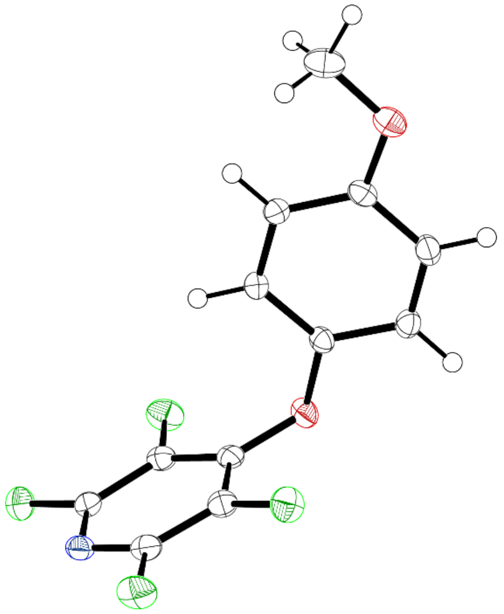

Empirical formula	$C_{12}H_7F_4NO_2$
Formula weight	273.19
Temperature/K	120.00
Crystal system	orthorhombic
Space group	$P2_12_12_1$
a/Å	5.1717(3)
b/Å	10.3813(6)
c/Å	20.4426(12)
α/°	90
β/°	90
γ/°	90
Volume/Å^3	1097.54(11)
Z	4
ρ_{calc}/cm^3	1.653
μ/mm^{-1}	0.158
F(000)	552.0
Crystal size/mm^3	0.243 × 0.177 × 0.064
Radiation	Mo Kα (λ = 0.71073)
2Θ range for data collection/°	4.4 to 67.634
Index ranges	-8 ≤ h ≤ 8, -16 ≤ k ≤ 16, -32 ≤ l ≤ 31
Reflections collected	39872
Independent reflections	4407 [R_{int} = 0.0498, R_{sigma} = 0.0298]
Data/restraints/parameters	4407/0/173
Goodness-of-fit on F^2	1.113
Final R indexes [I>=2σ (I)]	R_1 = 0.0463, wR_2 = 0.0950
Final R indexes [all data]	R_1 = 0.0562, wR_2 = 0.0989
Largest diff. peak/hole / e Å^{-3}	0.36/-0.24
Flack parameter	-0.5(2)
CCDC Number	2392093

Crystal structure: **7**

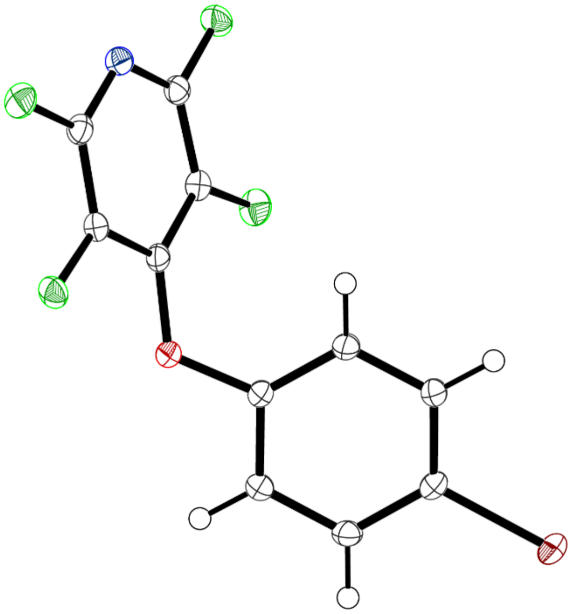

Empirical formula	$C_{11}H_4BrF_4NO$
Formula weight	322.06
Temperature/K	120.00
Crystal system	monoclinic
Space group	$P2_1/n$
a/Å	13.3478(4)
b/Å	5.8419(2)
c/Å	14.8721(5)
α/°	90
β/°	113.5380(10)
γ/°	90
Volume/Å^3	1063.18(6)
Z	4
ρ_{calc}/cm^3	2.012
μ/mm^{-1}	3.908
F(000)	624.0
Crystal size/mm^3	0.394 × 0.131 × 0.07
Radiation	Mo Kα (λ = 0.71073)
2Θ range for data collection/°	5.286 to 74.684
Index ranges	-22 ≤ h ≤ 22, -9 ≤ k ≤ 9, -25 ≤ l ≤ 24
Reflections collected	40342
Independent reflections	5123 [R_{int} = 0.0582, R_{sigma} = 0.0405]
Data/restraints/parameters	5123/0/163
Goodness-of-fit on F^2	1.041
Final R indexes [I>=2σ (I)]	R1 = 0.0316, wR2 = 0.0645
Final R indexes [all data]	R1 = 0.0535, wR2 = 0.0709
Largest diff. peak/hole / e Å^{-3}	0.66/-0.64
CCDC Number	2392094

Crystal structure: **8**

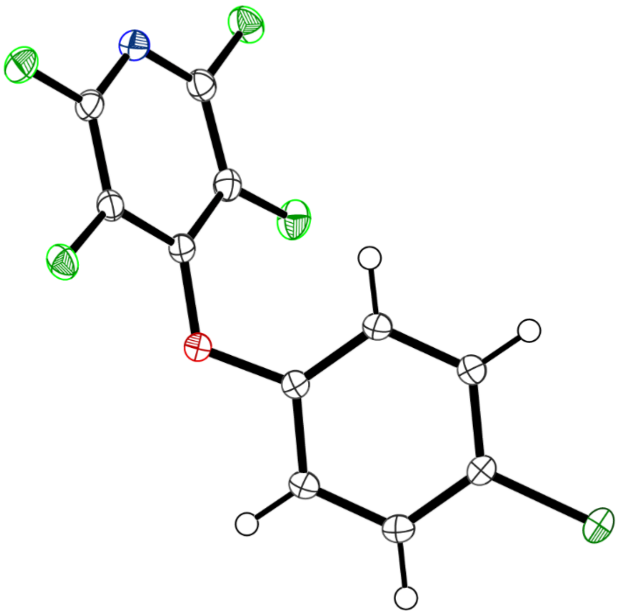

Empirical formula	$C_{11}H_4ClF_4NO$
Formula weight	277.60
Temperature/K	120.00
Crystal system	monoclinic
Space group	$P2_1/n$
a/Å	13.0866(3)
b/Å	5.84450(10)
c/Å	14.7452(4)
α/°	90
β/°	112.0060(10)
γ/°	90
Volume/Å^3	1045.62(4)
Z	4
ρ_{calc}/cm^3	1.763
μ/mm^{-1}	0.407
F(000)	552.0
Crystal size/mm^3	0.216 × 0.158 × 0.147
Radiation	Mo Kα (λ = 0.71073)
2Θ range for data collection/°	5.258 to 74.69
Index ranges	-21 ≤ h ≤ 21, -9 ≤ k ≤ 9, -24 ≤ l ≤ 25
Reflections collected	47725
Independent reflections	5171 [R_{int} = 0.0266, R_{sigma} = 0.0160]
Data/restraints/parameters	5171/0/163
Goodness-of-fit on F^2	1.043
Final R indexes [I>=2σ (I)]	R1 = 0.0335, wR2 = 0.0879
Final R indexes [all data]	R1 = 0.0397, wR2 = 0.0914
Largest diff. peak/hole / e Å^{-3}	0.55/-0.34
CCDC Number	2392095

Crystal structure: **9** (FISJUP)[1b]

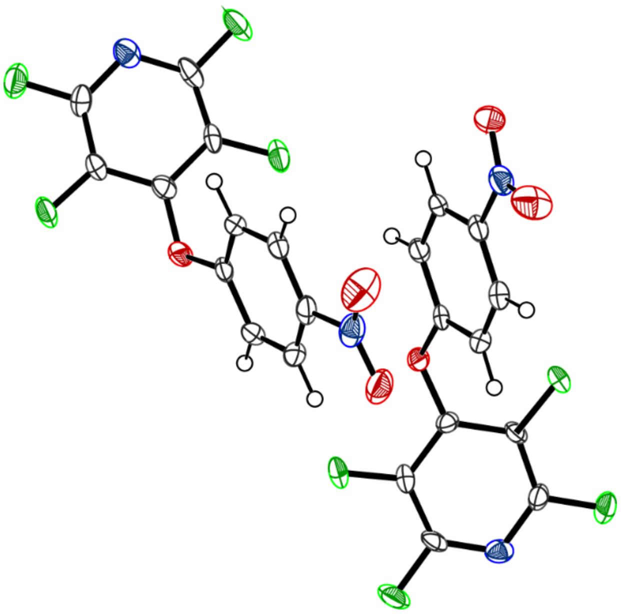

Empirical formula	$C_{11}H_4F_4N_2O_3$
Formula weight	288.16
Temperature/K	120.0
Crystal system	monoclinic
Space group	$P2_1/c$
a/Å	7.4591(17)
b/Å	10.786(3)
c/Å	26.797(6)
α/°	90
β/°	91.012(6)
γ/°	90
Volume/Å^3	2155.6(9)
Z	8
ρ_{calc}/cm^3	1.776
μ/mm^{-1}	0.175
F(000)	1152.0
Crystal size/mm^3	0.42 × 0.05 × 0.04
Radiation	MoKα (λ = 0.71073)
2Θ range for data collection/°	4.07 to 55.992
Index ranges	-9 ≤ h ≤ 9, -14 ≤ k ≤ 14, -35 ≤ l ≤ 35
Reflections collected	26864
Independent reflections	5207 [R_{int} = 0.1252, R_{sigma} = 0.1388]
Data/restraints/parameters	5207/0/361
Goodness-of-fit on F^2	1.010
Final R indexes [I>=2σ (I)]	R_1 = 0.0662, wR_2 = 0.1120
Final R indexes [all data]	R_1 = 0.1654, wR_2 = 0.1393
Largest diff. peak/hole / e Å^{-3}	0.28/-0.36

Crystal structure: **10**

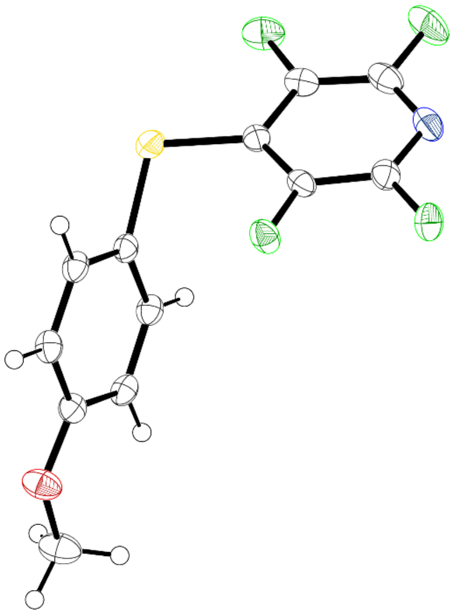

Empirical formula	$C_{12}H_7F_4NOS$
Formula weight	289.25
Temperature/K	120.00
Crystal system	orthorhombic
Space group	$P2_12_12_1$
a/Å	5.1181(3)
b/Å	12.5033(7)
c/Å	18.2341(10)
α/°	90
β/°	90
γ/°	90
Volume/Å^3	1166.86(11)
Z	4
ρ_{calc}/cm^3	1.646
μ/mm^{-1}	0.320
F(000)	584.0
Crystal size/mm^3	0.499 × 0.172 × 0.056
Radiation	Mo Kα (λ = 0.71073)
2Θ range for data collection/°	3.95 to 62.04
Index ranges	-7 ≤ h ≤ 7, -18 ≤ k ≤ 18, -26 ≤ l ≤ 26
Reflections collected	36486
Independent reflections	3731 [R_{int} = 0.0801, R_{sigma} = 0.0423]
Data/restraints/parameters	3731/0/173
Goodness-of-fit on F^2	1.075
Final R indexes [I>=2σ (I)]	R_1 = 0.0438, wR_2 = 0.0877
Final R indexes [all data]	R_1 = 0.0534, wR_2 = 0.0912
Largest diff. peak/hole / e Å^{-3}	0.23/-0.23
Flack parameter	0.02(6)
CCDC Number	2392096

Crystal structure: **11**

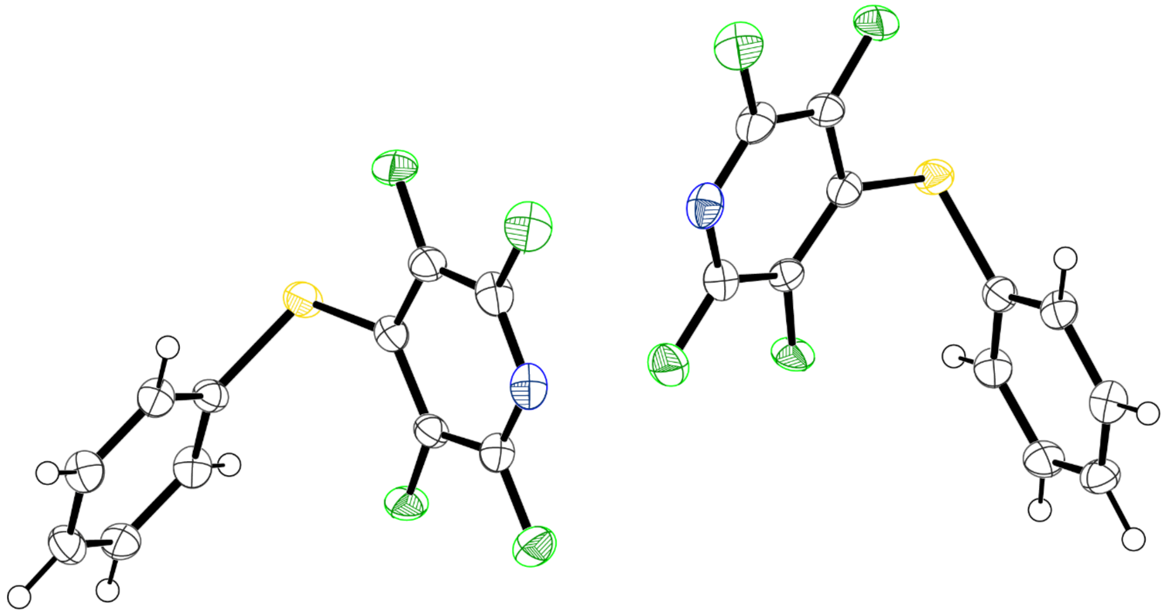

Empirical formula	$C_{11}H_5NF_4S$
Formula weight	259.22
Temperature/K	120
Crystal system	monoclinic
Space group	$P2_1$
a/Å	12.5441(12)
b/Å	5.1068(5)
c/Å	16.2407(16)
α/°	90
β/°	97.427(3)
γ/°	90
Volume/Å^3	1031.65(17)
Z	4
ρ_{calc}/cm^3	1.669
μ/mm^{-1}	0.344
F(000)	520.0
Crystal size/mm^3	0.57 × 0.128 × 0.072
Radiation	MoKα (λ = 0.71073)
2Θ range for data collection/°	4.388 to 56.02
Index ranges	-16 ≤ h ≤ 16, -6 ≤ k ≤ 6, -21 ≤ l ≤ 21
Reflections collected	21905
Independent reflections	4959 [R_{int} = 0.0605, R_{sigma} = 0.0459]
Data/restraints/parameters	4959/1/307
Goodness-of-fit on F^2	1.079
Final R indexes [I>=2σ (I)]	R_1 = 0.0471, wR_2 = 0.1175
Final R indexes [all data]	R_1 = 0.0500, wR_2 = 0.1198
Largest diff. peak/hole / e Å^{-3}	0.46/-0.48
Flack parameter	-0.03(6)
CCDC Number	2392097

Crystal structure: **12**

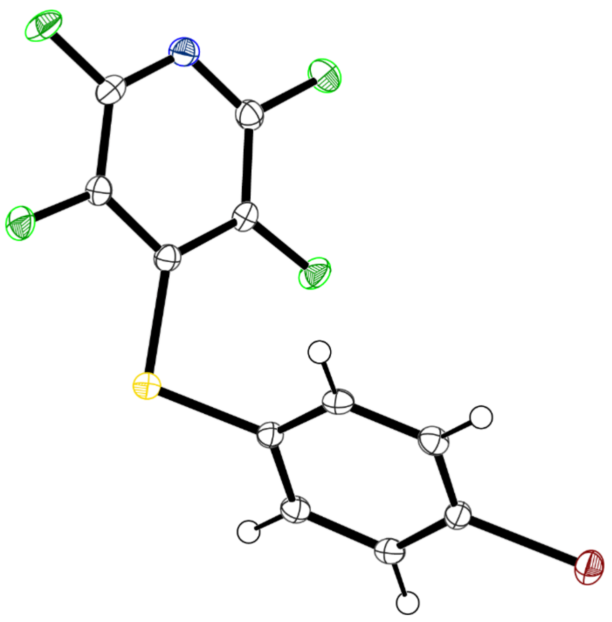

Empirical formula	$C_{11}H_4BrF_4NS$
Formula weight	338.12
Temperature/K	120.00
Crystal system	monoclinic
Space group	$P2_1/c$
a/Å	7.5339(3)
b/Å	6.1598(3)
c/Å	23.6919(11)
α/°	90
β/°	96.1396(18)
γ/°	90
Volume/Å^3	1093.17(9)
Z	4
ρ_{calc}/cm^3	2.054
μ/mm^{-1}	3.983
F(000)	656.0
Crystal size/mm^3	0.11 × 0.05 × 0.01
Radiation	Mo Kα (λ = 0.71073)
2Θ range for data collection/°	5.438 to 59.988
Index ranges	-10 ≤ h ≤ 10, -8 ≤ k ≤ 8, -33 ≤ l ≤ 33
Reflections collected	31779
Independent reflections	3192 [R_{int} = 0.0476, R_{sigma} = 0.0249]
Data/restraints/parameters	3192/0/163
Goodness-of-fit on F^2	1.056
Final R indexes [I>=2σ (I)]	R_1 = 0.0295, wR_2 = 0.0652
Final R indexes [all data]	R_1 = 0.0374, wR_2 = 0.0680
Largest diff. peak/hole / e Å^{-3}	0.43/-0.49
CCDC Number	2392098

Crystal structure: **13**

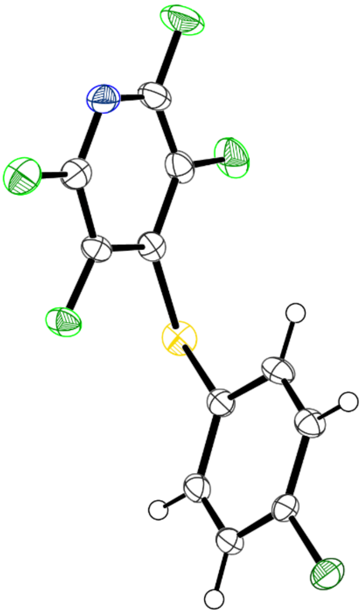

Empirical formula	$C_{11}H_4ClF_4NS$
Formula weight	293.66
Temperature/K	120.00
Crystal system	orthorhombic
Space group	$Pca2_1$
a/Å	7.3137(2)
b/Å	10.9595(3)
c/Å	13.8535(3)
α/°	90
β/°	90
γ/°	90
Volume/Å^3	1110.42(5)
Z	4
ρ_{calc}/cm^3	1.757
μ/mm^{-1}	0.564
F(000)	584.0
Crystal size/mm^3	0.33 × 0.29 × 0.09
Radiation	Mo Kα (λ = 0.71073)
2Θ range for data collection/°	4.74 to 61.978
Index ranges	-10 ≤ h ≤ 10, -15 ≤ k ≤ 15, -20 ≤ l ≤ 20
Reflections collected	35065
Independent reflections	3509 [R_{int} = 0.0428, R_{sigma} = 0.0217]
Data/restraints/parameters	3509/1/164
Goodness-of-fit on F^2	1.125
Final R indexes [I>=2σ (I)]	R_1 = 0.0288, wR_2 = 0.0727
Final R indexes [all data]	R_1 = 0.0293, wR_2 = 0.0730
Largest diff. peak/hole / e Å^{-3}	0.27/-0.20
Flack parameter	0.01(3)
CCDC Number	2392099

Crystal structure: **14**

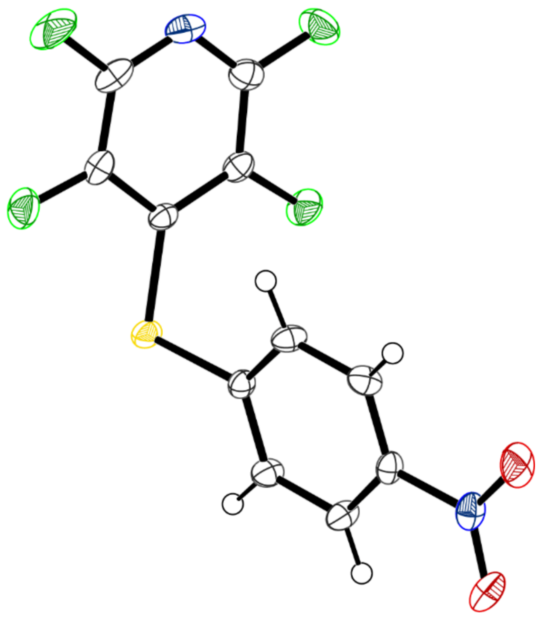

Empirical formula	$C_{11}H_4F_4N_2O_2S$
Formula weight	304.22
Temperature/K	120.00
Crystal system	monoclinic
Space group	Pc
a/Å	5.7048(3)
b/Å	12.7901(7)
c/Å	7.8026(4)
α/°	90
β/°	90.181(2)
γ/°	90
Volume/Å^3	569.31(5)
Z	2
ρ_{calc}/cm^3	1.775
μ/mm^{-1}	0.341
F(000)	304.0
Crystal size/mm^3	0.19 × 0.09 × 0.02
Radiation	Mo Kα (λ = 0.71073)
2Θ range for data collection/°	6.116 to 59.978
Index ranges	-8 ≤ h ≤ 8, -17 ≤ k ≤ 17, -10 ≤ l ≤ 10
Reflections collected	18922
Independent reflections	3308 [R_{int} = 0.0342, R_{sigma} = 0.0257]
Data/restraints/parameters	3308/2/197
Goodness-of-fit on F^2	1.097
Final R indexes [I>=2σ (I)]	R_1 = 0.0338, wR_2 = 0.0742
Final R indexes [all data]	R_1 = 0.0354, wR_2 = 0.0749
Largest diff. peak/hole / e Å^{-3}	0.25/-0.19
Flack parameter	-0.03(4)
CCDC Number	2392100

Crystal structure: **15**

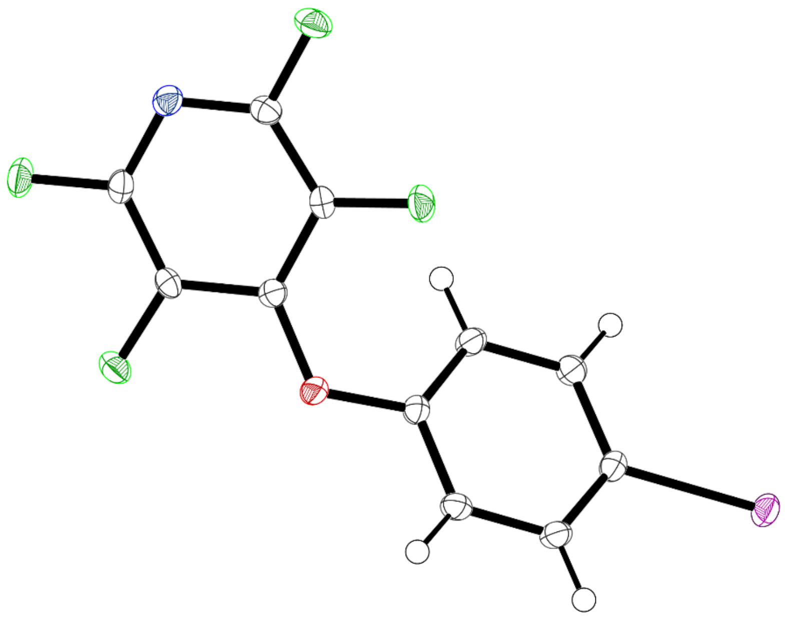

Empirical formula	$C_{11}H_4F_4INO$
Formula weight	369.05
Temperature/K	120.00
Crystal system	monoclinic
Space group	$P2_1/n$
a/Å	13.7192(7)
b/Å	5.8431(3)
c/Å	15.2620(8)
α/°	90
β/°	115.101(2)
γ/°	90
Volume/Å^3	1107.90(10)
Z	4
ρ_{calc}g/cm^3	2.213
μ/mm^{-1}	2.931
F(000)	696.0
Crystal size/mm^3	0.17 × 0.09 × 0.08
Radiation	MoKα (λ = 0.71073)
2Θ range for data collection/°	5.258 to 59.982
Index ranges	$-19 \leq h \leq 19$, $-8 \leq k \leq 8$, $-21 \leq l \leq 21$
Reflections collected	17915
Independent reflections	3210 [R_{int} = 0.0261, R_{sigma} = 0.0184]
Data/restraints/parameters	3210/0/163
Goodness-of-fit on F^2	1.120
Final R indexes [I>=2σ (I)]	R_1 = 0.0171, wR_2 = 0.0376
Final R indexes [all data]	R_1 = 0.0190, wR_2 = 0.0383
Largest diff. peak/hole / e Å^{-3}	0.44/-0.54
CCDC Number	2392103

Crystal structure: **16**

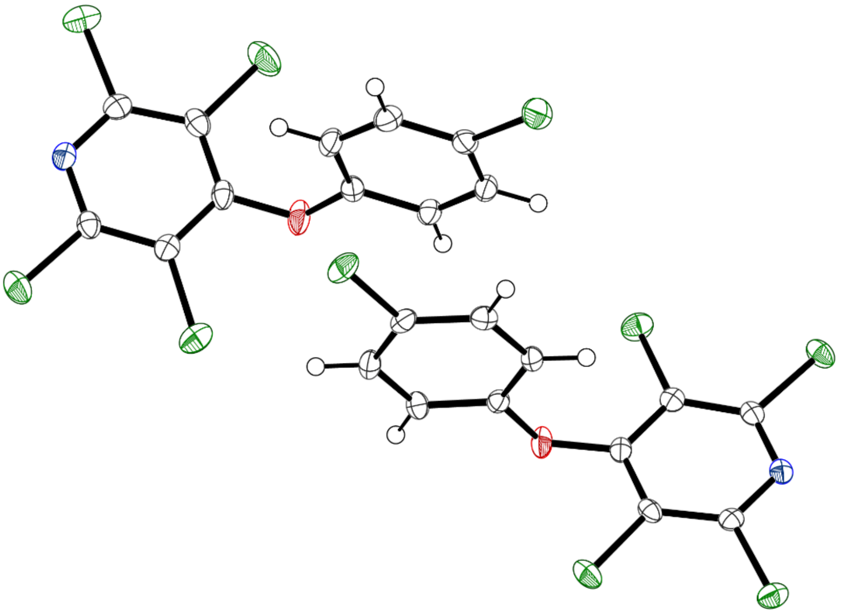

Empirical formula	$C_{11}H_4Cl_5NO$
Formula weight	343.40
Temperature/K	120.00
Crystal system	monoclinic
Space group	$P2_1/c$
a/Å	21.5601(9)
b/Å	6.8474(3)
c/Å	17.8817(8)
α/°	90
β/°	103.405(2)
γ/°	90
Volume/Å^3	2567.96(19)
Z	8
ρ_{calc}/cm^3	1.776
μ/mm^{-1}	1.113
F(000)	1360.0
Crystal size/mm^3	0.23 × 0.19 × 0.19
Radiation	MoKα (λ = 0.71073)
2Θ range for data collection/°	4.636 to 59.994
Index ranges	-30 ≤ h ≤ 30, -9 ≤ k ≤ 9, -25 ≤ l ≤ 25
Reflections collected	42676
Independent reflections	7462 [R_{int} = 0.0305, R_{sigma} = 0.0230]
Data/restraints/parameters	7462/0/325
Goodness-of-fit on F^2	1.074
Final R indexes [I>=2σ (I)]	R_1 = 0.0314, wR_2 = 0.0643
Final R indexes [all data]	R_1 = 0.0380, wR_2 = 0.0672
Largest diff. peak/hole / e Å^{-3}	0.42/-0.34
CCDC Number	2392101

Crystal structure: **17**

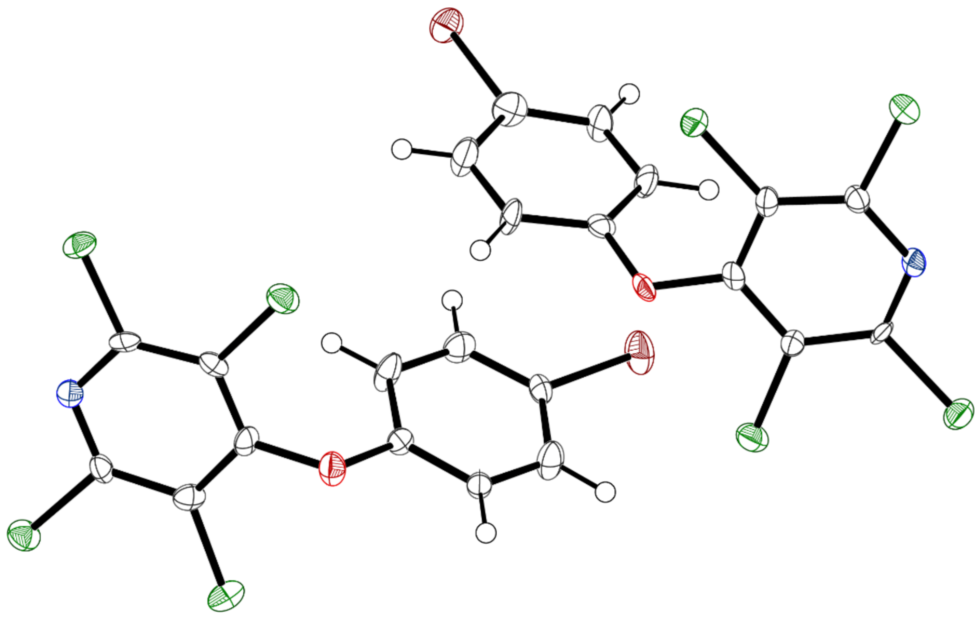

Empirical formula	$C_{11}H_4BrCl_4NO$
Formula weight	387.86
Temperature/K	120.00
Crystal system	triclinic
Space group	P-1
a/Å	6.7111(4)
b/Å	8.4325(5)
c/Å	23.0864(13)
α/°	85.0300(18)
β/°	89.023(2)
γ/°	89.1030(19)
Volume/Å^3	1301.26(13)
Z	4
ρ_{calc}/cm^3	1.980
μ/mm^{-1}	3.963
F(000)	752.0
Crystal size/mm^3	0.09 × 0.06 × 0.02
Radiation	Mo Kα (λ = 0.71073)
2Θ range for data collection/°	4.85 to 58.998
Index ranges	-9 ≤ h ≤ 9, -11 ≤ k ≤ 11, 0 ≤ l ≤ 31
Reflections collected	5724
Independent reflections	5724 [R_{int} = 0.0403, R_{sigma} = 0.0487]
Data/restraints/parameters	5724/0/325
Goodness-of-fit on F^2	1.132
Final R indexes [I>=2σ (I)]	R_1 = 0.0640, wR_2 = 0.1592
Final R indexes [all data]	R_1 = 0.0737, wR_2 = 0.1641
Largest diff. peak/hole / e Å^{-3}	0.71/-1.14
CCDC Number	2392102

Crystal structure: **18**

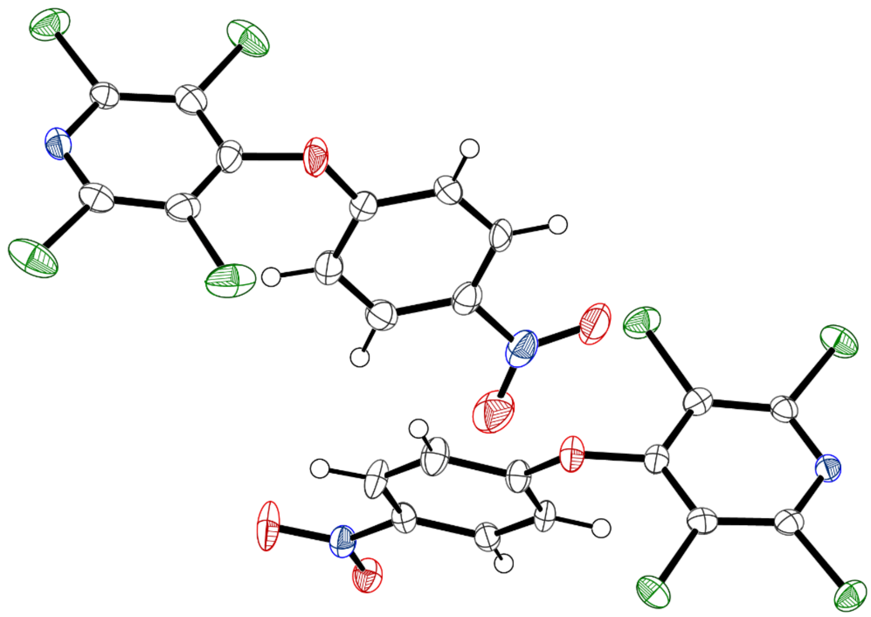

Empirical formula	$C_{11}H_4Cl_4N_2O_3$
Formula weight	353.96
Temperature/K	120.00
Crystal system	triclinic
Space group	P-1
a/Å	7.2430(4)
b/Å	8.9058(5)
c/Å	22.1478(12)
α/°	85.007(2)
β/°	88.544(2)
γ/°	68.139(2)
Volume/Å^3	1320.83(13)
Z	4
ρ_{calc}/cm^3	1.780
μ/mm^{-1}	0.902
F(000)	704.0
Crystal size/mm^3	0.09 × 0.08 × 0.07
Radiation	MoKα (λ = 0.71073)
2Θ range for data collection/°	4.946 to 59.998
Index ranges	-9 ≤ h ≤ 10, -12 ≤ k ≤ 12, -31 ≤ l ≤ 31
Reflections collected	23006
Independent reflections	7672 [R_{int} = 0.0626, R_{sigma} = 0.0937]
Data/restraints/parameters	7672/0/361
Goodness-of-fit on F^2	1.051
Final R indexes [I>=2σ (I)]	R_1 = 0.0708, wR_2 = 0.1339
Final R indexes [all data]	R_1 = 0.1228, wR_2 = 0.1545
Largest diff. peak/hole / e Å^{-3}	0.40/-0.53
CCDC Number	2392104

Crystal structure: **19**

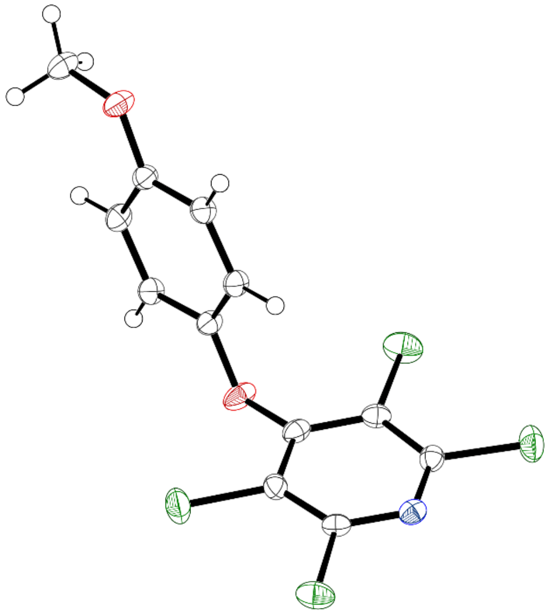

Empirical formula	$C_{12}H_7Cl_4NO_2$
Formula weight	338.99
Temperature/K	120.00
Crystal system	monoclinic
Space group	$P2_1/c$
a/Å	6.7939(2)
b/Å	21.8618(7)
c/Å	8.9954(3)
α/°	90
β/°	96.7060(10)
γ/°	90
Volume/Å^3	1326.92(7)
Z	4
ρ_{calc}g/cm^3	1.697
μ/mm^{-1}	0.886
F(000)	680.0
Crystal size/mm^3	0.16 × 0.12 × 0.11
Radiation	MoKα (λ = 0.71073)
2Θ range for data collection/°	4.926 to 59.98
Index ranges	-9 ≤ h ≤ 9, -30 ≤ k ≤ 30, -12 ≤ l ≤ 12
Reflections collected	22731
Independent reflections	3863 [R_{int} = 0.0350, R_{sigma} = 0.0262]
Data/restraints/parameters	3863/0/173
Goodness-of-fit on F^2	1.049
Final R indexes [I>=2σ (I)]	R_1 = 0.0306, wR_2 = 0.0617
Final R indexes [all data]	R_1 = 0.0411, wR_2 = 0.0663
CCDC Number	2392105

Crystal structure: **20**

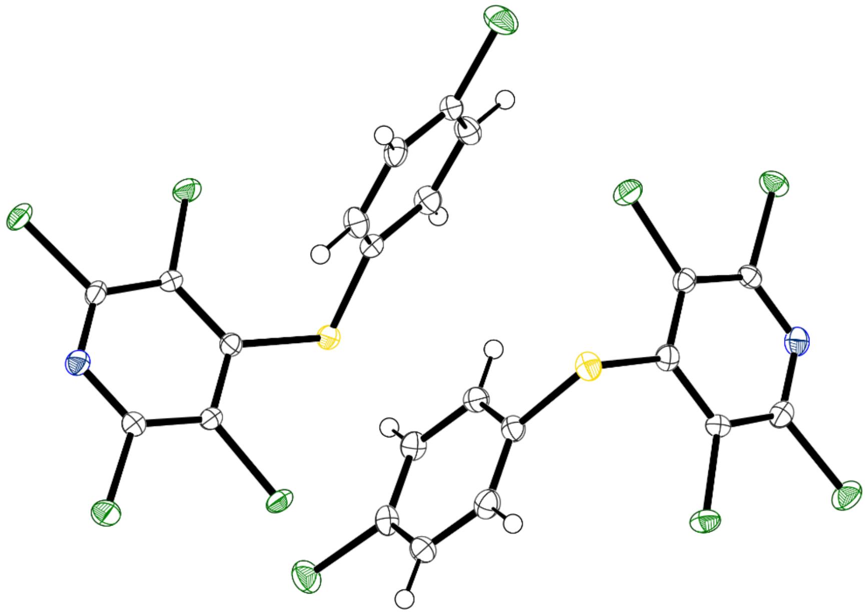

Empirical formula	$C_{11}H_4Cl_5NS$
Formula weight	359.46
Temperature/K	120.00
Crystal system	monoclinic
Space group	$P2_1/c$
a/Å	22.3918(10)
b/Å	7.0830(3)
c/Å	17.1257(8)
α/°	90
β/°	105.912(2)
γ/°	90
Volume/Å^3	2612.1(2)
Z	8
ρ_{calc}/cm^3	1.828
μ/mm^{-1}	1.247
F(000)	1424.0
Crystal size/mm^3	0.15 × 0.12 × 0.11
Radiation	MoKα (λ = 0.71073)
2Θ range for data collection/°	4.788 to 58.998
Index ranges	-31 ≤ h ≤ 31, -9 ≤ k ≤ 9, -23 ≤ l ≤ 23
Reflections collected	42120
Independent reflections	7267 [R_{int} = 0.0558, R_{sigma} = 0.0445]
Data/restraints/parameters	7267/0/325
Goodness-of-fit on F^2	1.072
Final R indexes [I>=2σ (I)]	R_1 = 0.0605, wR_2 = 0.1453
Final R indexes [all data]	R_1 = 0.0784, wR_2 = 0.1561
Largest diff. peak/hole / e Å^{-3}	2.27/-0.67
CCDC Number	2392106

Crystal structure: **21**

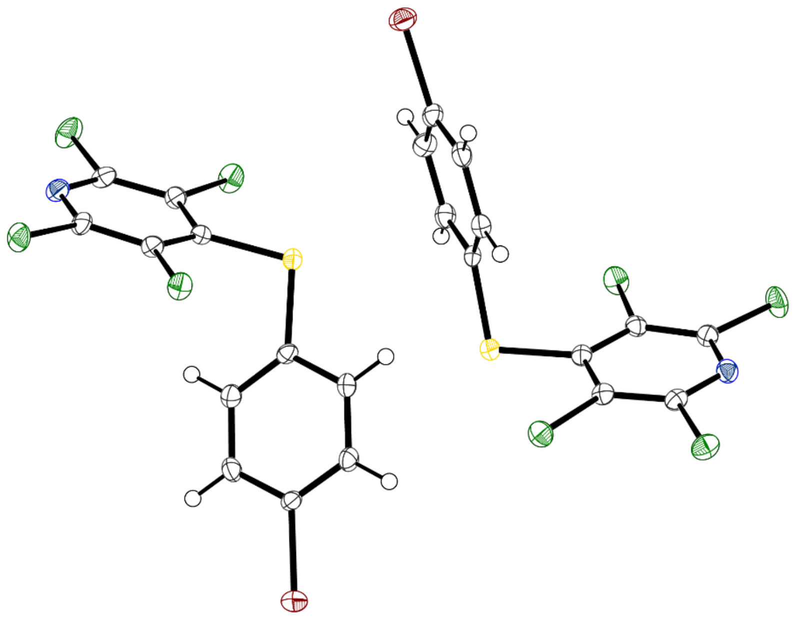

Empirical formula	$C_{11}H_4BrCl_4NS$
Formula weight	403.92
Temperature/K	120.00
Crystal system	monoclinic
Space group	$P2_1/c$
a/Å	21.9683(7)
b/Å	7.1152(2)
c/Å	17.1369(6)
α/°	90
β/°	96.4090(10)
γ/°	90
Volume/Å^3	2661.91(15)
Z	8
ρ_{calc}/cm^3	2.016
μ/mm^{-1}	4.025
F(000)	1568.0
Crystal size/mm^3	0.11 × 0.1 × 0.06
Radiation	MoKα (λ = 0.71073)
2Θ range for data collection/°	4.784 to 59.996
Index ranges	-30 ≤ h ≤ 30, -10 ≤ k ≤ 10, -24 ≤ l ≤ 24
Reflections collected	44716
Independent reflections	7735 [R_{int} = 0.0571, R_{sigma} = 0.0490]
Data/restraints/parameters	7735/0/325
Goodness-of-fit on F^2	0.831
Final R indexes [I>=2σ (I)]	R_1 = 0.0399, wR_2 = 0.1097
Final R indexes [all data]	R_1 = 0.0619, wR_2 = 0.1263
Largest diff. peak/hole / e Å^{-3}	0.94/-1.02
CCDC Number	2392107

Crystal structure: **22**

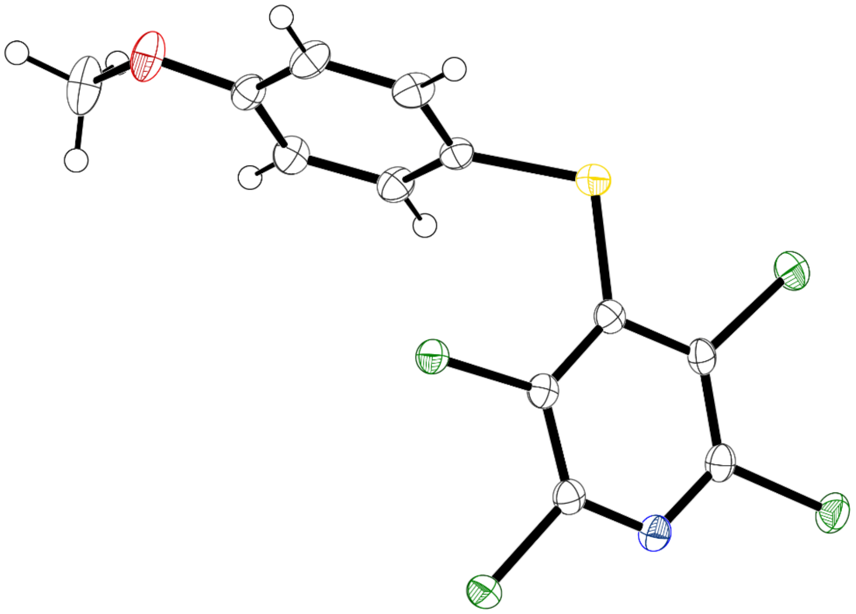

Empirical formula	$C_{12}H_7Cl_4NOS$
Formula weight	355.05
Temperature/K	120.00
Crystal system	orthorhombic
Space group	$P2_12_12_1$
a/Å	5.6490(2)
b/Å	11.5028(5)
c/Å	20.9664(9)
α/°	90
β/°	90
γ/°	90
Volume/Å^3	1362.38(10)
Z	4
ρ_{calc}/cm^3	1.731
μ/mm^{-1}	1.010
F(000)	712.0
Crystal size/mm^3	0.24 × 0.09 × 0.08
Radiation	MoKα (λ = 0.71073)
2Θ range for data collection/°	5.258 to 59.998
Index ranges	-7 ≤ h ≤ 7, -16 ≤ k ≤ 16, -29 ≤ l ≤ 29
Reflections collected	23608
Independent reflections	3958 [R_{int} = 0.0606, R_{sigma} = 0.0498]
Data/restraints/parameters	3958/0/173
Goodness-of-fit on F^2	1.020
Final R indexes [I>=2σ (I)]	R_1 = 0.0351, wR_2 = 0.0661
Final R indexes [all data]	R_1 = 0.0464, wR_2 = 0.0708
Largest diff. peak/hole / e Å^{-3}	0.33/-0.29
Flack parameter	0.05(4)
CCDC Number	2392108

Crystal structure: **23**

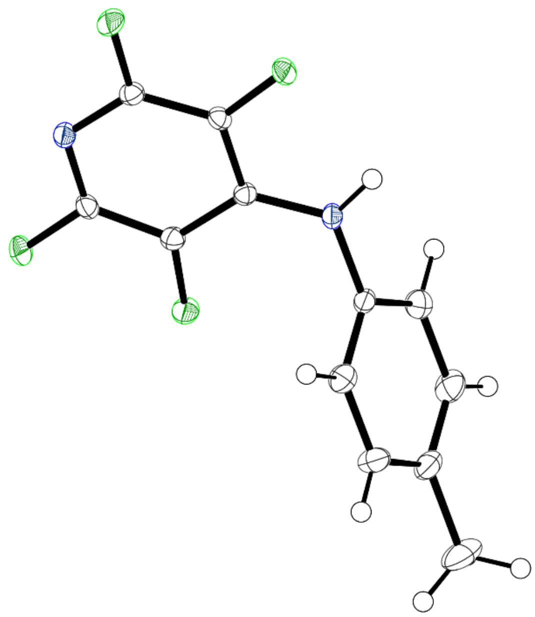

Empirical formula	$C_{12}H_8F_4N_2$
Formula weight	256.20
Temperature/K	120.00
Crystal system	orthorhombic
Space group	Pbca
a/Å	12.9519(4)
b/Å	6.4097(2)
c/Å	25.6755(8)
α/°	90
β/°	90
γ/°	90
Volume/Å^3	2131.52(11)
Z	8
ρ_{calc}/cm^3	1.597
μ/mm^{-1}	0.146
F(000)	1040.0
Crystal size/mm^3	0.21 × 0.16 × 0.08
Radiation	Mo Kα (λ = 0.71073)
2Θ range for data collection/°	4.468 to 60
Index ranges	-18 ≤ h ≤ 18, -9 ≤ k ≤ 9, -36 ≤ l ≤ 36
Reflections collected	46746
Independent reflections	3100 [R_{int} = 0.0451, R_{sigma} = 0.0175]
Data/restraints/parameters	3100/0/195
Goodness-of-fit on F^2	1.190
Final R indexes [I>=2σ (I)]	R_1 = 0.0535, wR_2 = 0.1159
Final R indexes [all data]	R_1 = 0.0588, wR_2 = 0.1186
Largest diff. peak/hole / e Å^{-3}	0.42/-0.27
CCDC Number	2392109

Crystal structure: **24**

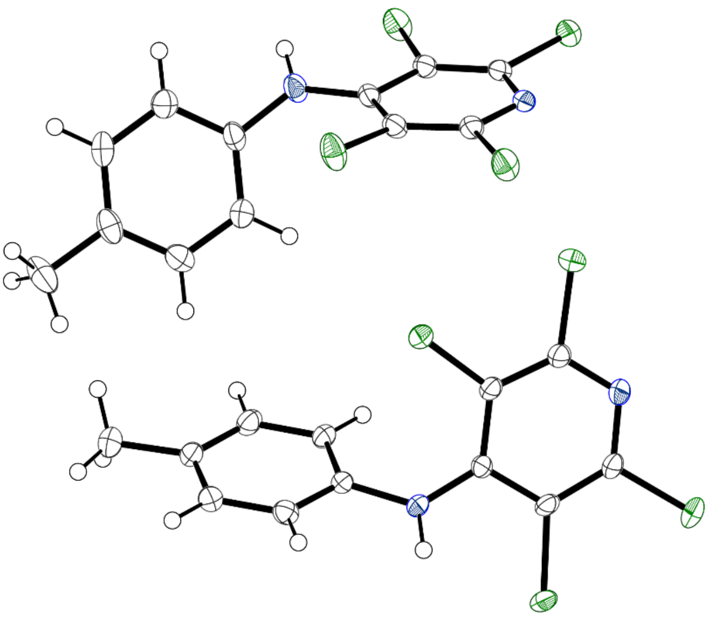

Empirical formula	$C_{12}H_8Cl_4N_2$
Formula weight	322.00
Temperature/K	120.00
Crystal system	triclinic
Space group	P-1
a/Å	9.7059(7)
b/Å	10.6772(7)
c/Å	13.8788(9)
α/°	105.635(3)
β/°	90.491(3)
γ/°	107.122(2)
Volume/Å^3	1317.63(16)
Z	4
ρ_{calc}g/cm^3	1.623
μ/mm^{-1}	0.879
F(000)	648.0
Crystal size/mm^3	0.23 × 0.13 × 0.1
Radiation	MoKα (λ = 0.71073)
2Θ range for data collection/°	4.164 to 59.998
Index ranges	-13 ≤ h ≤ 13, -15 ≤ k ≤ 14, -19 ≤ l ≤ 19
Reflections collected	22710
Independent reflections	7645 [R_{int} = 0.0508, R_{sigma} = 0.0606]
Data/restraints/parameters	7645/0/335
Goodness-of-fit on F^2	1.038
Final R indexes [I>=2σ (I)]	R_1 = 0.0526, wR_2 = 0.1129
Final R indexes [all data]	R_1 = 0.0741, wR_2 = 0.1234
Largest diff. peak/hole / e Å^{-3}	0.49/-0.55
CCDC Number	2392110

Crystal structure: **25**

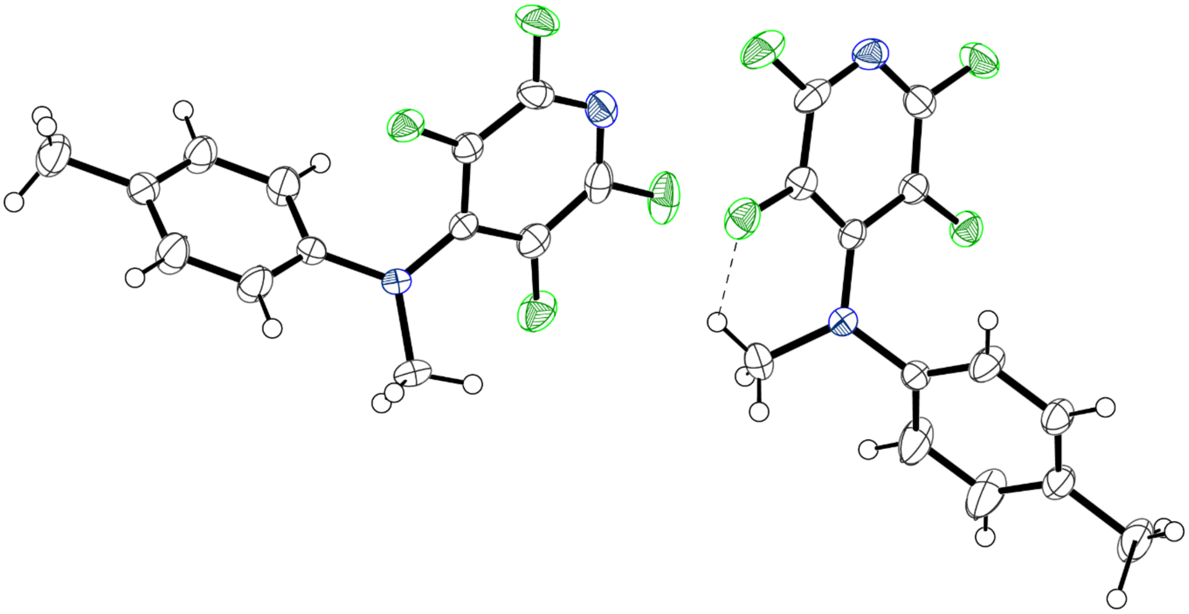

Empirical formula	$C_{13}H_{10}F_4N_2$
Formula weight	270.23
Temperature/K	120.00
Crystal system	monoclinic
Space group	$P2_1/c$
a/Å	29.157(3)
b/Å	4.9301(5)
c/Å	16.9166(18)
α/°	90
β/°	102.455(4)
γ/°	90
Volume/Å^3	2374.5(4)
Z	8
ρ_{calc}g/cm^3	1.512
μ/mm^{-1}	0.135
F(000)	1104.0
Crystal size/mm^3	0.81 × 0.09 × 0.06
Radiation	MoKα (λ = 0.71073)
2Θ range for data collection/°	4.292 to 51.984
Index ranges	-35 ≤ h ≤ 35, -6 ≤ k ≤ 6, -20 ≤ l ≤ 20
Reflections collected	29615
Independent reflections	4648 [R_{int} = 0.0815, R_{sigma} = 0.0571]
Data/restraints/parameters	4648/534/347
Goodness-of-fit on F^2	1.049
Final R indexes [I>=2σ (I)]	R_1 = 0.0612, wR_2 = 0.1204
Final R indexes [all data]	R_1 = 0.0931, wR_2 = 0.1350
Largest diff. peak/hole / e Å^{-3}	0.27/-0.29
CCDC Number	2392111

Crystal structure: **26**

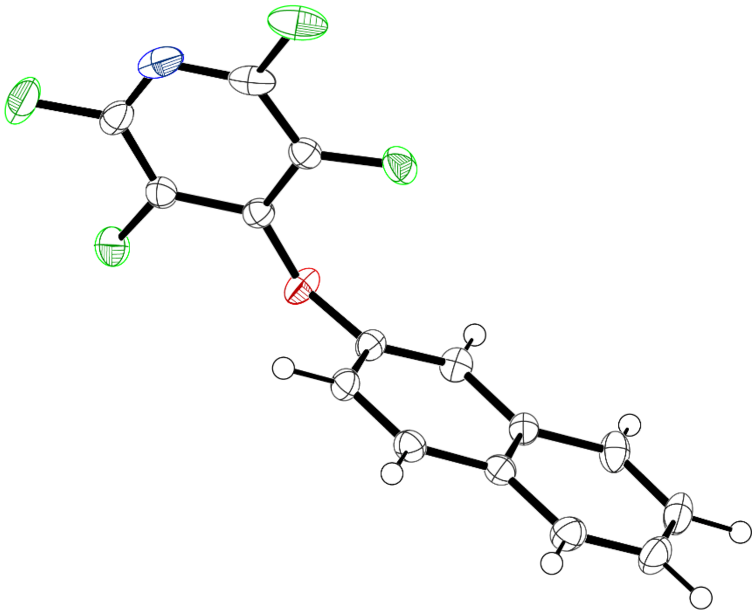

Empirical formula	$C_{15}H_7F_4NO$
Formula weight	293.22
Temperature/K	120.0
Crystal system	orthorhombic
Space group	$P2_12_12_1$
a/Å	5.4538(2)
b/Å	9.2473(3)
c/Å	24.0778(8)
α/°	90
β/°	90
γ/°	90
Volume/Å^3	1214.31(7)
Z	4
ρ_{calc}/cm^3	1.604
μ/mm^{-1}	0.143
F(000)	592.0
Crystal size/mm^3	0.38 × 0.21 × 0.17
Radiation	MoKα (λ = 0.71073)
2Θ range for data collection/°	4.718 to 57.982
Index ranges	-7 ≤ h ≤ 7, -12 ≤ k ≤ 12, -32 ≤ l ≤ 32
Reflections collected	18778
Independent reflections	3234 [R_{int} = 0.0309, R_{sigma} = 0.0241]
Data/restraints/parameters	3234/0/218
Goodness-of-fit on F^2	1.051
Final R indexes [I>=2σ (I)]	R_1 = 0.0327, wR_2 = 0.0734
Final R indexes [all data]	R_1 = 0.0418, wR_2 = 0.0767
Largest diff. peak/hole / e Å^{-3}	0.22/-0.18
Flack parameter	0.35(18)
CCDC Number	2392112

Crystal structure: **27** (JUTCAF)[1a]

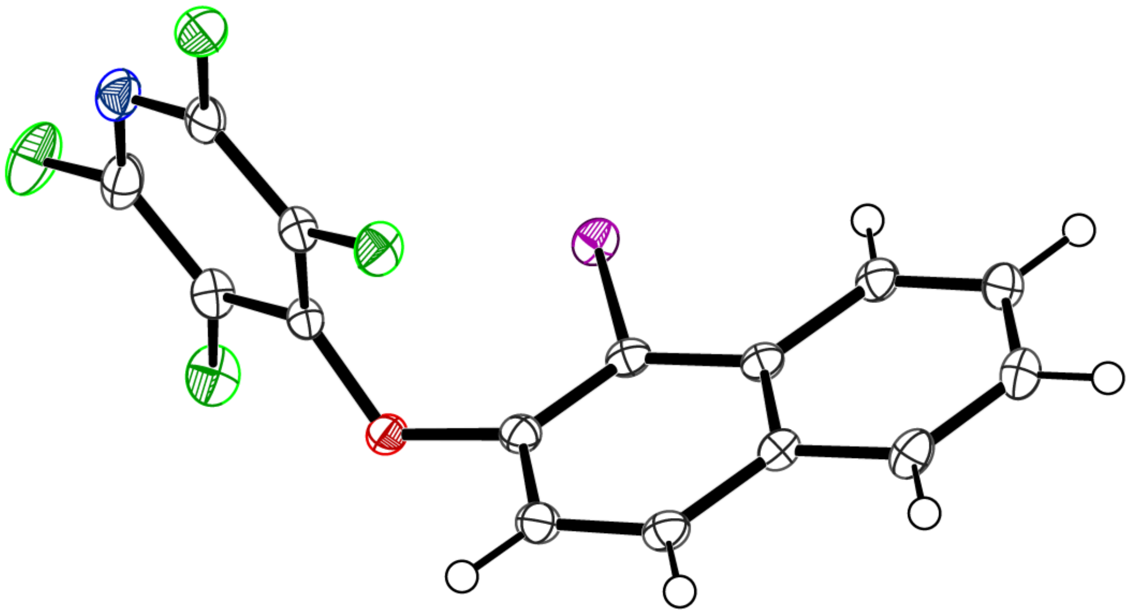

Empirical formula	$C_{15}H_6F_4INO$
Formula weight	419.11
Temperature/K	120.0
Crystal system	monoclinic
Space group	$P2_1$
a/Å	4.6677(3)
b/Å	11.0185(7)
c/Å	13.0170(8)
α/°	90
β/°	90.865(2)
γ/°	90
Volume/Å^3	669.40(7)
Z	2
ρ_{calc}/cm^3	2.079
μ/mm^{-1}	2.439
F(000)	400.0
Crystal size/mm^3	0.23 × 0.1 × 0.06
Radiation	MoKα (λ = 0.71073)
2Θ range for data collection/°	6.26 to 59.99
Index ranges	-6 ≤ h ≤ 6, -15 ≤ k ≤ 15, -18 ≤ l ≤ 18
Reflections collected	14627
Independent reflections	3908 [R_{int} = 0.0261, R_{sigma} = 0.0228]
Data/restraints/parameters	3908/1/199
Goodness-of-fit on F^2	$C_{15}H_6F_4INO$
Final R indexes [I>=2σ (I)]	419.11
Final R indexes [all data]	120.0
Largest diff. peak/hole / e Å^{-3}	monoclinic

Crystal structure: **28**

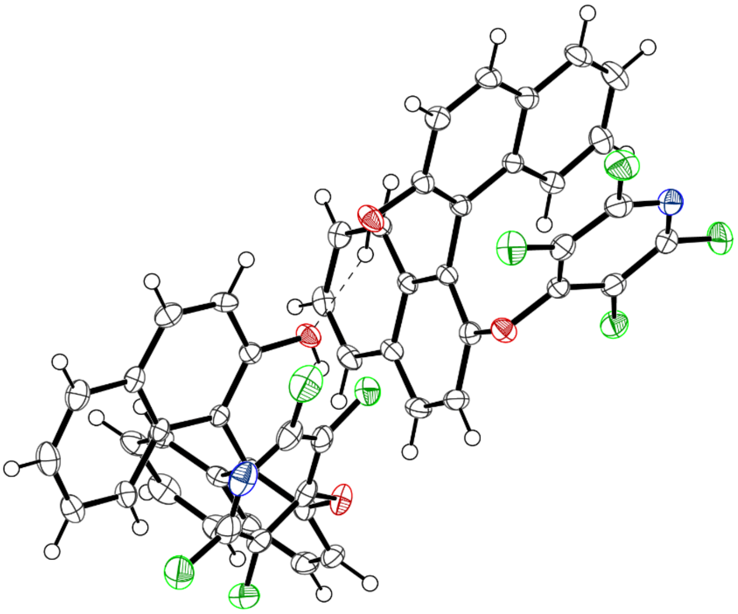

Empirical formula	$C_{25}H_{13}F_4NO_2$
Formula weight	435.36
Temperature/K	296(2)
Crystal system	triclinic
Space group	P-1
a/Å	10.7725(2)
b/Å	13.1234(3)
c/Å	15.0726(3)
α/°	106.6400(10)
β/°	101.9180(10)
γ/°	101.6390(10)
Volume/Å^3	1918.69(7)
Z	4
ρ_{calc}/cm^3	1.507
μ/mm^{-1}	0.122
F(000)	888.0
Crystal size/mm^3	0.14 × 0.17 × 0.19
Radiation	MoKα (λ = 0.71073)
2Θ range for data collection/°	3.636 to 59.998
Index ranges	-15 ≤ h ≤ 15, -18 ≤ k ≤ 18, -21 ≤ l ≤ 21
Reflections collected	33532
Independent reflections	11124 [R_{int} = 0.0390, R_{sigma} = 0.0564]
Data/restraints/parameters	11124/0/579
Goodness-of-fit on F^2	1.046
Final R indexes [I>=2σ (I)]	R1 = 0.0567, wR2 = 0.1402
Final R indexes [all data]	R1 = 0.0903, wR2 = 0.1596
Largest diff. peak/hole / e Å^{-3}	0.43/-0.31
CCDC Number	2392113

Crystal structure: **29** (FISJOJ)[1b]

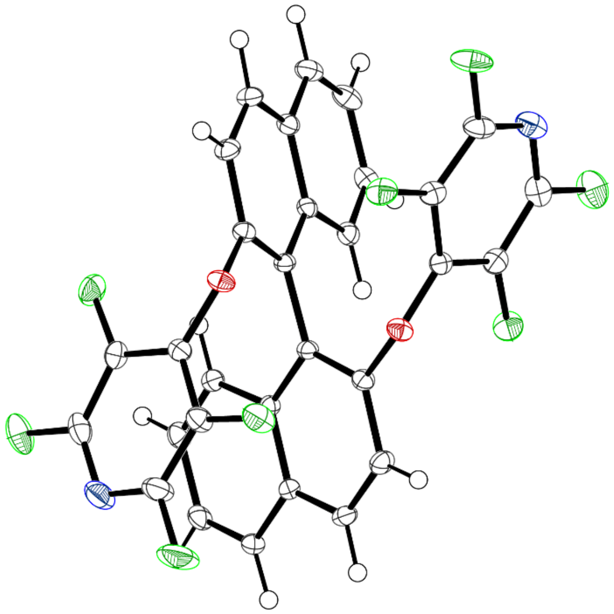

Empirical formula	$C_{30}H_{12}F_8N_2O_2$
Formula weight	584.42
Temperature/K	120.0
Crystal system	monoclinic
Space group	$P2_1/c$
a/Å	13.2804(5)
b/Å	8.7572(4)
c/Å	21.1478(8)
α/°	90
β/°	106.2235(15)
γ/°	90
Volume/Å^3	2361.53(17)
Z	4
ρ_{calc}/cm^3	1.644
μ/mm^{-1}	0.147
F(000)	1176.0
Crystal size/mm^3	0.35 × 0.28 × 0.14
Radiation	MoKα (λ = 0.71073)
2Θ range for data collection/°	4.374 to 58.996
Index ranges	-18 ≤ h ≤ 18, -12 ≤ k ≤ 12, -29 ≤ l ≤ 29
Reflections collected	49176
Independent reflections	6565 [R_{int} = 0.0333, R_{sigma} = 0.0211]
Data/restraints/parameters	6565/0/427
Goodness-of-fit on F^2	1.047
Final R indexes [I>=2σ (I)]	R_1 = 0.0384, wR_2 = 0.0953
Final R indexes [all data]	R_1 = 0.0507, wR_2 = 0.1017
Largest diff. peak/hole / e Å^{-3}	0.38/-0.25

Crystal structure: **30**

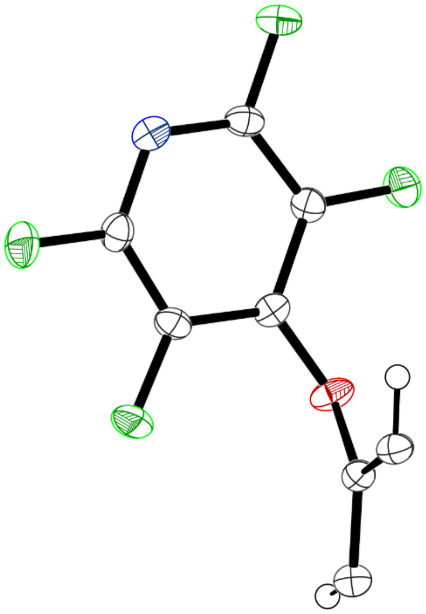

Empirical formula	$C_{16}H_4F_8N_2O_2$
Formula weight	408.21
Temperature/K	120.00
Crystal system	monoclinic
Space group	$P2_1/c$
a/Å	5.1719(5)
b/Å	9.2296(8)
c/Å	15.5250(13)
α/°	90
β/°	94.978(4)
γ/°	90
Volume/Å^3	738.28(11)
Z	2
ρ_{calc}g/cm^3	1.836
μ/mm^{-1}	0.190
F(000)	404.0
Crystal size/mm^3	0.57 × 0.12 × 0.07
Radiation	MoKα (λ = 0.71073)
2Θ range for data collection/°	5.14 to 59.996
Index ranges	-7 ≤ h ≤ 7, -12 ≤ k ≤ 12, -21 ≤ l ≤ 21
Reflections collected	11849
Independent reflections	2153 [R_{int} = 0.0669, R_{sigma} = 0.0515]
Data/restraints/parameters	2153/0/128
Goodness-of-fit on F^2	1.037
Final R indexes [I>=2σ (I)]	R_1 = 0.0448, wR_2 = 0.1077
Final R indexes [all data]	R_1 = 0.0621, wR_2 = 0.1183
Largest diff. peak/hole / e Å^{-3}	0.45/-0.27
CCDC Number	2392114

Crystal structure: **31**

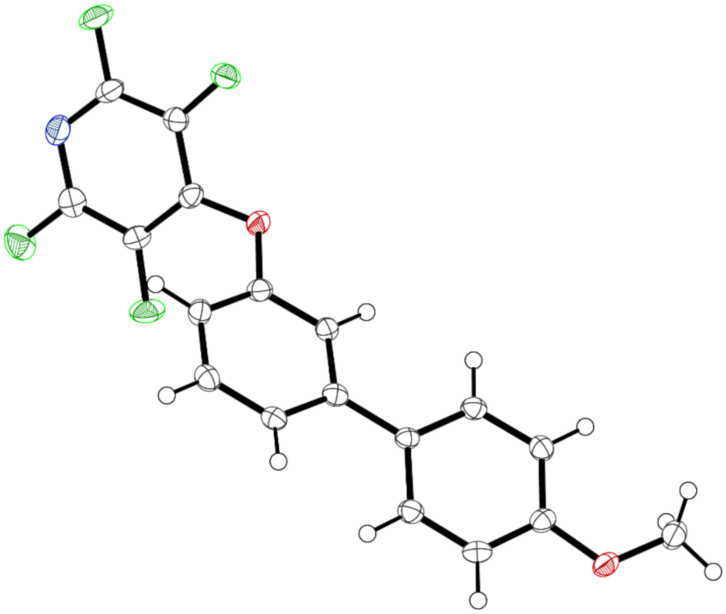

Empirical formula	$C_{18}H_{11}F_4NO_2$
Formula weight	349.28
Temperature/K	120.00
Crystal system	monoclinic
Space group	Cc
a/Å	30.6153(14)
b/Å	7.6257(3)
c/Å	6.3650(3)
α/°	90
β/°	98.846(2)
γ/°	90
Volume/Å^3	1468.32(11)
Z	4
ρ_{calc}/cm^3	1.580
μ/mm^{-1}	0.137
F(000)	712.0
Crystal size/mm^3	0.26 × 0.09 × 0.07
Radiation	MoKα (λ = 0.71073)
2Θ range for data collection/°	5.386 to 59.994
Index ranges	-42 ≤ h ≤ 42, -10 ≤ k ≤ 10, -8 ≤ l ≤ 8
Reflections collected	12144
Independent reflections	4242 [R_{int} = 0.0396, R_{sigma} = 0.0517]
Data/restraints/parameters	4242/2/227
Goodness-of-fit on F^2	1.010
Final R indexes [I>=2σ (I)]	R1 = 0.0458, wR2 = 0.0972
Final R indexes [all data]	R1 = 0.0647, wR2 = 0.1067
Largest diff. peak/hole / e Å^{-3}	0.25/-0.24
Flack parameter	-0.3(4)
CCDC Number	2392115

Crystal structure: **32**

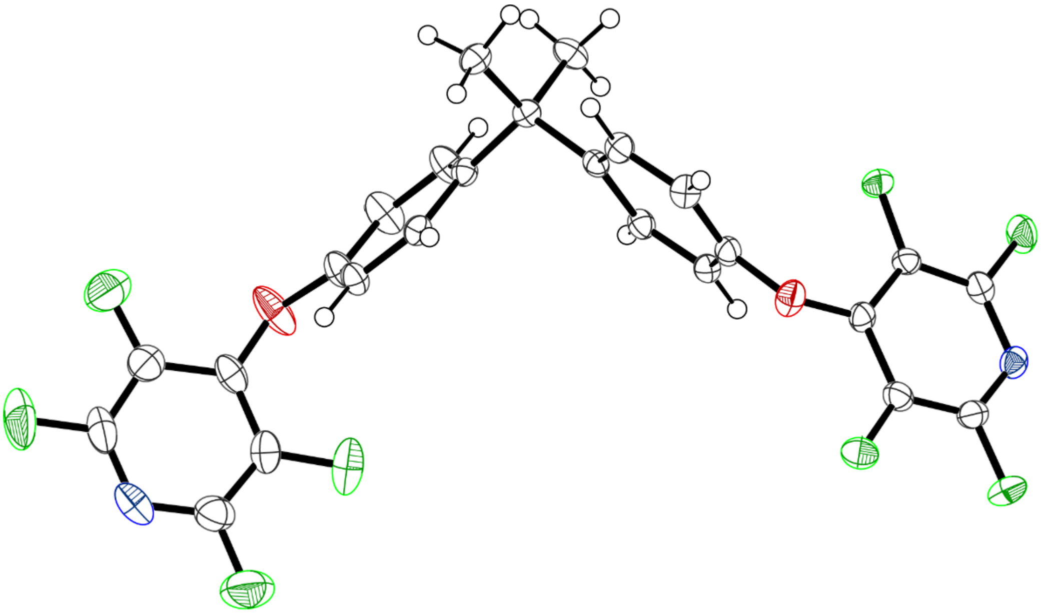

Empirical formula	$C_{25}H_{14}F_8N_2O_2$
Formula weight	526.38
Temperature/K	120.00
Crystal system	orthorhombic
Space group	Pbca
a/Å	8.6878(4)
b/Å	10.9872(5)
c/Å	46.4292(19)
α/°	90
β/°	90
γ/°	90
Volume/Å^3	4431.9(3)
Z	8
ρ_{calc}g/cm^3	1.578
μ/mm^{-1}	0.147
F(000)	2128.0
Crystal size/mm^3	0.24 × 0.15 × 0.12
Radiation	MoKα (λ = 0.71073)
2Θ range for data collection/°	5.006 to 60
Index ranges	-12 ≤ h ≤ 12, -15 ≤ k ≤ 15, -65 ≤ l ≤ 65
Reflections collected	72251
Independent reflections	6452 [R_{int} = 0.0583, R_{sigma} = 0.0336]
Data/restraints/parameters	6452/0/336
Goodness-of-fit on F^2	1.120
Final R indexes [I>=2σ (I)]	R_1 = 0.0627, wR_2 = 0.1129
Final R indexes [all data]	R_1 = 0.0865, wR_2 = 0.1218
Largest diff. peak/hole / e Å^{-3}	0.25/-0.32
CCDC Number	2392116

Crystal structure: **33**

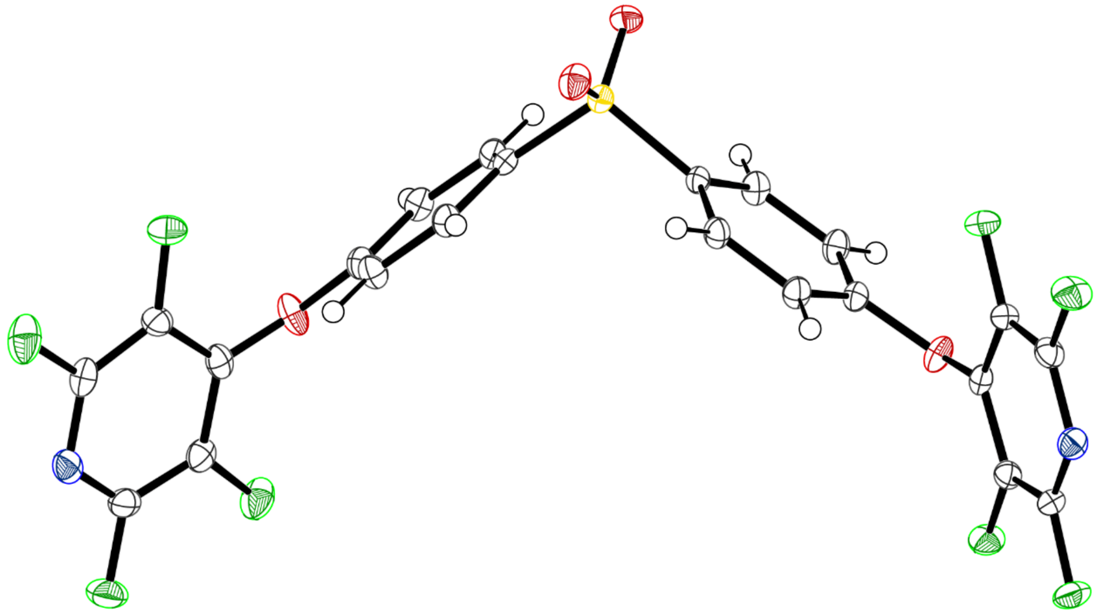

Empirical formula	$C_{22}H_8F_8N_2O_4S$
Formula weight	548.36
Temperature/K	120.00
Crystal system	monoclinic
Space group	C2
a/Å	20.2129(8)
b/Å	5.8939(2)
c/Å	18.8358(8)
α/°	90
β/°	116.5550(10)
γ/°	90
Volume/Å^3	2007.24(14)
Z	4
ρ_{calc}g/cm^3	1.815
μ/mm^{-1}	0.273
F(000)	1096.0
Crystal size/mm^3	0.34 × 0.16 × 0.15
Radiation	MoKα (λ = 0.71073)
2Θ range for data collection/°	4.506 to 59.99
Index ranges	-28 ≤ h ≤ 28, -8 ≤ k ≤ 8, -26 ≤ l ≤ 26
Reflections collected	17226
Independent reflections	5836 [R_{int} = 0.0533, R_{sigma} = 0.0573]
Data/restraints/parameters	5836/1/334
Goodness-of-fit on F^2	1.044
Final R indexes [I>=2σ (I)]	R_1 = 0.0376, wR_2 = 0.0819
Final R indexes [all data]	R_1 = 0.0434, wR_2 = 0.0849
Largest diff. peak/hole / e Å^{-3}	0.29/-0.37
Flack parameter	0.12(4)
CCDC Number	2392117

Crystal structure: **34** (JUTBUY)[1a]

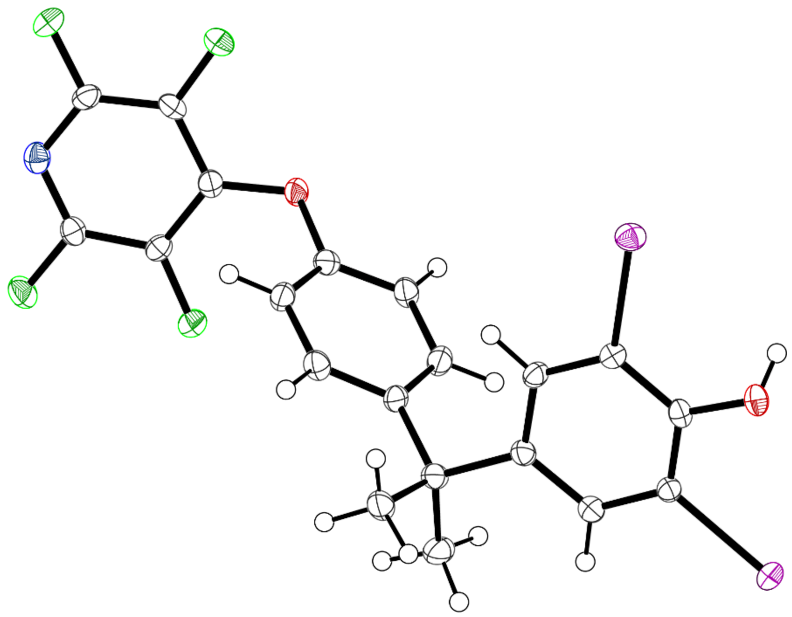

Empirical formula	$C_{20}H_{13}F_4I_2NO_2$
Formula weight	629.11
Temperature/K	120.0
Crystal system	monoclinic
Space group	$P2_1/c$
a/Å	12.3523(3)
b/Å	12.7890(3)
c/Å	12.8327(3)
α/°	90
β/°	94.170(2)
γ/°	90
Volume/Å^3	2021.86(9)
Z	4
ρ_{calc}/cm^3	2.067
μ/mm^{-1}	3.163
F(000)	1192.0
Crystal size/mm^3	0.38 × 0.17 × 0.15
Radiation	MoKα (λ = 0.71073)
2Θ range for data collection/°	4.59 to 58.994
Index ranges	-16 ≤ h ≤ 17, -17 ≤ k ≤ 17, -17 ≤ l ≤ 17
Reflections collected	20412
Independent reflections	5635 [R_{int} = 0.0394, R_{sigma} = 0.0384]
Data/restraints/parameters	5635/0/264
Goodness-of-fit on F^2	1.024
Final R indexes [I>=2σ (I)]	R_1 = 0.0294, wR_2 = 0.0642
Final R indexes [all data]	R_1 = 0.0426, wR_2 = 0.0711
Largest diff. peak/hole / e Å^{-3}	0.81/-0.90

Crystal structure: **35**

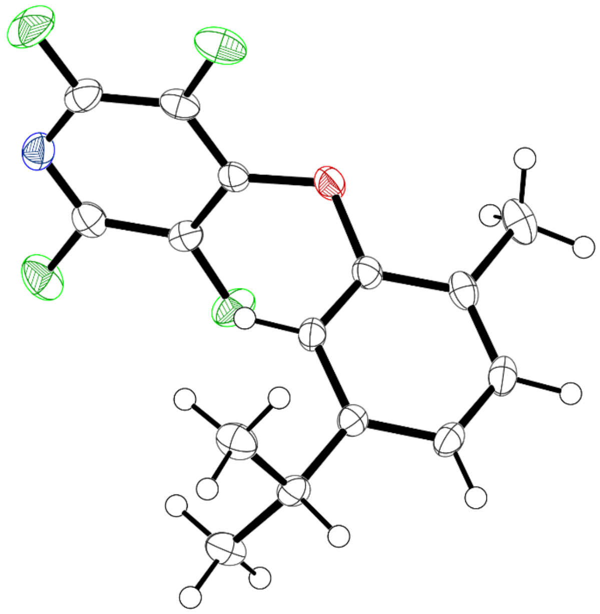

Empirical formula	$C_{15}H_{13}F_4NO$
Formula weight	299.26
Temperature/K	120.00
Crystal system	monoclinic
Space group	$P2_1/n$
a/Å	9.4929(3)
b/Å	8.8670(3)
c/Å	16.6490(5)
α/°	90
β/°	102.6977(13)
γ/°	90
Volume/Å^3	1367.13(8)
Z	4
ρ_{calc}/cm^3	1.454
μ/mm^{-1}	0.128
F(000)	616.0
Crystal size/mm^3	0.38 × 0.19 × 0.08
Radiation	Mo Kα (λ = 0.71073)
2Θ range for data collection/°	5.522 to 59.998
Index ranges	-13 ≤ h ≤ 13, -12 ≤ k ≤ 12, -23 ≤ l ≤ 23
Reflections collected	31720
Independent reflections	3980 [R_{int} = 0.0413, R_{sigma} = 0.0242]
Data/restraints/parameters	3980/0/242
Goodness-of-fit on F^2	1.060
Final R indexes [I>=2σ (I)]	R_1 = 0.0469, wR_2 = 0.1090
Final R indexes [all data]	R_1 = 0.0567, wR_2 = 0.1146
Largest diff. peak/hole / e Å^{-3}	0.43/-0.32
CCDC Number	2392118

Crystal structure: **36**

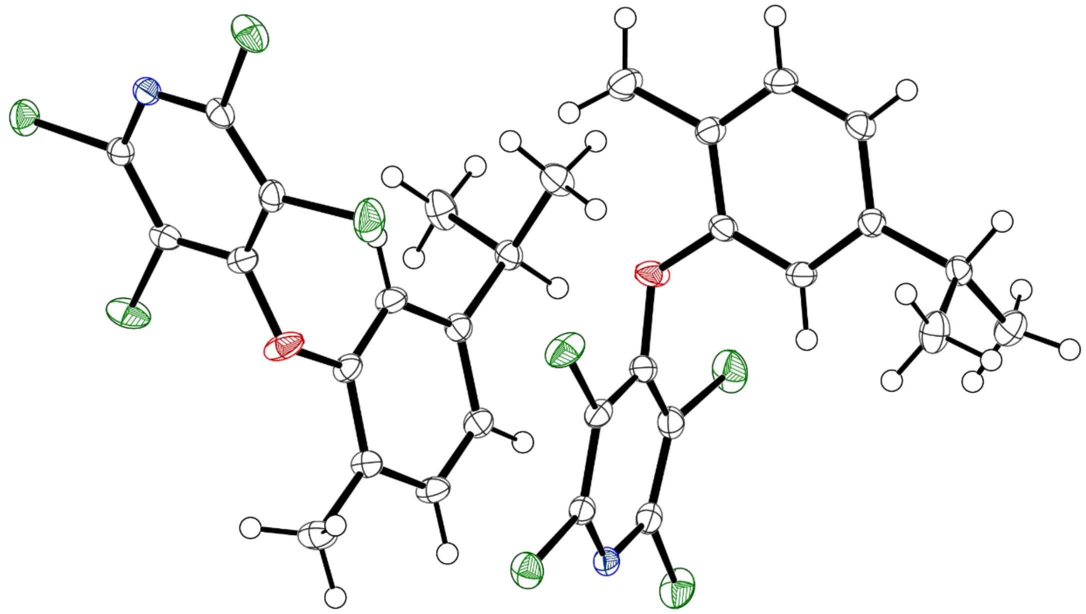

Empirical formula	$C_{15}H_{13}Cl_4NO$
Formula weight	365.06
Temperature/K	120.00
Crystal system	monoclinic
Space group	$P2_1/c$
a/Å	8.8146(3)
b/Å	10.3821(3)
c/Å	17.3296(6)
α/°	90
β/°	96.9790(10)
γ/°	90
Volume/Å^3	1574.15(9)
Z	4
ρ_{calc}g/cm^3	1.540
μ/mm^{-1}	0.748
F(000)	744.0
Crystal size/mm^3	0.525 × 0.279 × 0.192
Radiation	MoKα (λ = 0.71073)
2Θ range for data collection/°	4.582 to 60.068
Index ranges	-12 ≤ h ≤ 12, -14 ≤ k ≤ 14, -24 ≤ l ≤ 24
Reflections collected	26550
Independent reflections	4600 [R_{int} = 0.0240, R_{sigma} = 0.0167]
Data/restraints/parameters	4600/0/193
Goodness-of-fit on F^2	1.053
Final R indexes [I>=2σ (I)]	R_1 = 0.0260, wR_2 = 0.0664
Final R indexes [all data]	R_1 = 0.0295, wR_2 = 0.0683
Largest diff. peak/hole / e Å^{-3}	0.39/-0.26
CCDC Number	2392119

Crystal structure: **37**

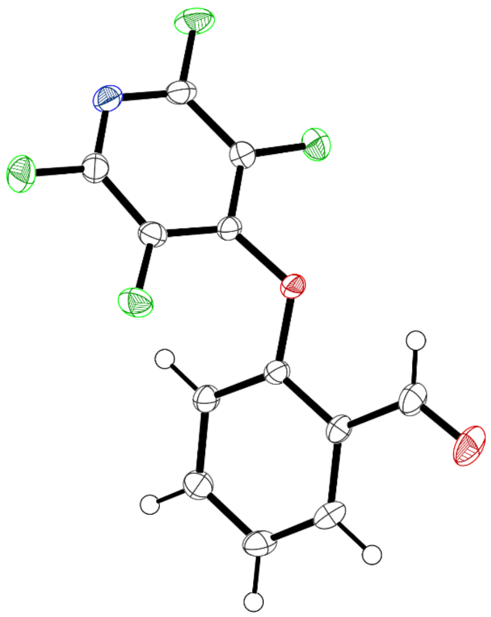

Empirical formula	$C_{12}H_5F_4NO_2$
Formula weight	271.17
Temperature/K	120.00
Crystal system	triclinic
Space group	P-1
a/Å	7.4513(2)
b/Å	7.9087(2)
c/Å	9.6886(3)
α/°	75.6080(10)
β/°	75.5120(10)
γ/°	86.7280(10)
Volume/Å^3	535.44(3)
Z	2
ρ_{calc}g/cm^3	1.682
μ/mm^{-1}	0.161
F(000)	272.0
Crystal size/mm^3	0.47 × 0.41 × 0.21
Radiation	MoKα (λ = 0.71073)
2Θ range for data collection/°	4.476 to 59.992
Index ranges	-10 ≤ h ≤ 10, -11 ≤ k ≤ 11, -13 ≤ l ≤ 13
Reflections collected	9013
Independent reflections	3092 [R_{int} = 0.0257, R_{sigma} = 0.0273]
Data/restraints/parameters	3092/0/172
Goodness-of-fit on F^2	1.035
Final R indexes [I>=2σ (I)]	R_1 = 0.0382, wR_2 = 0.0955
Final R indexes [all data]	R_1 = 0.0435, wR_2 = 0.0989
Largest diff. peak/hole / e Å^{-3}	0.38/-0.20
CCDC Number	2392120

Crystal structure: **38**

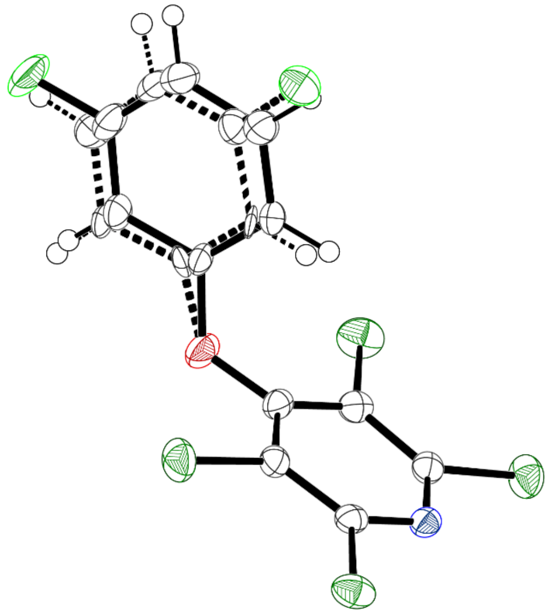

Empirical formula	$C_{11}H_4Cl_4FNO$
Formula weight	326.95
Temperature/K	120.00
Crystal system	triclinic
Space group	P-1
a/Å	6.8399(2)
b/Å	9.0246(2)
c/Å	10.6704(3)
α/°	100.2700(10)
β/°	105.4190(10)
γ/°	99.8220(10)
Volume/Å^3	608.05(3)
Z	2
ρ_{calc}/cm^3	1.786
μ/mm^{-1}	0.969
F(000)	324.0
Crystal size/mm^3	0.311 × 0.23 × 0.152
Radiation	Mo Kα (λ = 0.71073)
2Θ range for data collection/°	4.074 to 66.194
Index ranges	-10 ≤ h ≤ 10, -13 ≤ k ≤ 13, -16 ≤ l ≤ 16
Reflections collected	25078
Independent reflections	4616 [R_{int} = 0.0323, R_{sigma} = 0.0246]
Data/restraints/parameters	4616/501/227
Goodness-of-fit on F^2	1.025
Final R indexes [I>=2σ (I)]	R_1 = 0.0335, wR_2 = 0.0813
Final R indexes [all data]	R_1 = 0.0474, wR_2 = 0.0883
Largest diff. peak/hole / e Å^{-3}	0.45/-0.32
CCDC Number	2392121

Crystal structure: **39**

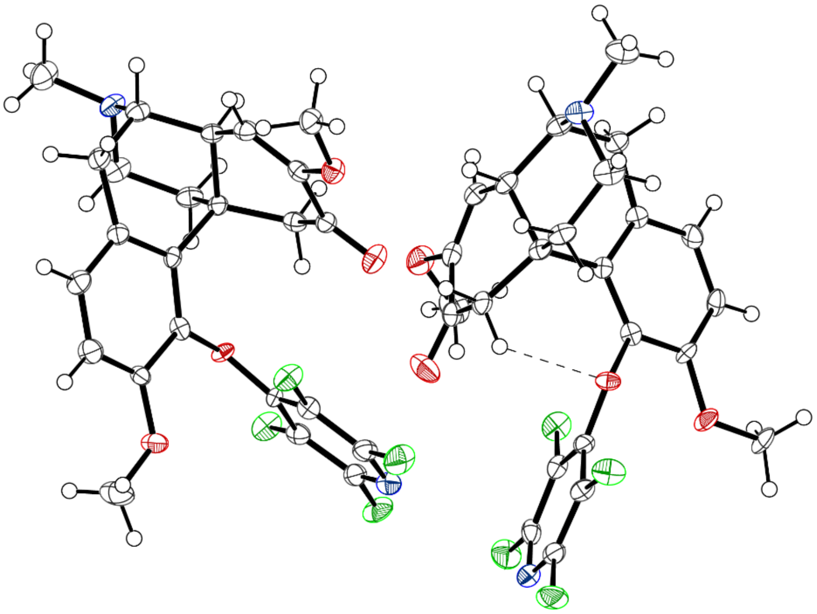

Empirical formula	$C_{24}H_{22}F_4N_2O_4$
Formula weight	478.43
Temperature/K	120.00
Crystal system	monoclinic
Space group	$P2_1$
a/Å	10.3202(11)
b/Å	13.7561(14)
c/Å	15.4577(16)
α/°	90
β/°	98.611(4)
γ/°	90
Volume/Å^3	2169.7(4)
Z	4
ρ_{calc}g/cm^3	1.465
μ/mm^{-1}	0.123
F(000)	992.0
Crystal size/mm^3	0.16 × 0.14 × 0.08
Radiation	MoKα (λ = 0.71073)
2Θ range for data collection/°	3.992 to 59.99
Index ranges	-14 ≤ h ≤ 14, -19 ≤ k ≤ 19, -21 ≤ l ≤ 21
Reflections collected	37925
Independent reflections	12590 [R_{int} = 0.0866, R_{sigma} = 0.1236]
Data/restraints/parameters	12590/1/619
Goodness-of-fit on F^2	1.050
Final R indexes [I>=2σ (I)]	R_1 = 0.0765, wR_2 = 0.1347
Final R indexes [all data]	R_1 = 0.1282, wR_2 = 0.1566
Largest diff. peak/hole / e Å^{-3}	0.31/-0.34
Flack parameter	0.2(5)
CCDC Number	2392122

Crystal structure: **40**

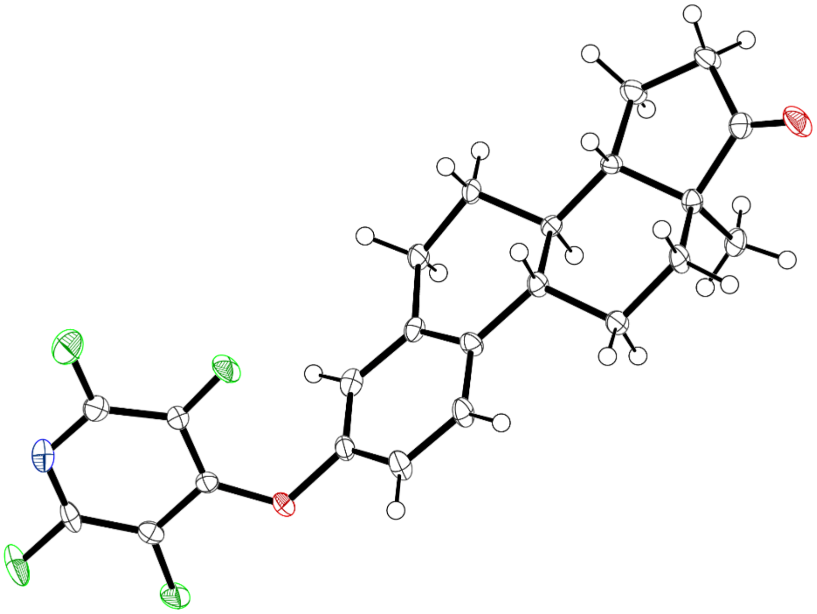

Empirical formula	$C_{23}H_{21}F_4NO_2$
Formula weight	419.41
Temperature/K	296.15
Crystal system	monoclinic
Space group	$P2_1$
a/Å	7.1096(6)
b/Å	7.9622(7)
c/Å	17.1109(16)
α/°	90
β/°	98.347(3)
γ/°	90
Volume/Å^3	958.35(15)
Z	2
ρ_{calc}g/cm^3	1.453
μ/mm^{-1}	0.119
F(000)	436.0
Crystal size/mm^3	0.32 × 0.27 × 0.24
Radiation	MoKα (λ = 0.71073)
2Θ range for data collection/°	4.812 to 60
Index ranges	$-10 \le h \le 9$, $-11 \le k \le 11$, $0 \le l \le 24$
Reflections collected	5482
Independent reflections	5482 [R_{int} = ?, R_{sigma} = 0.0454]
Data/restraints/parameters	5482/1/273
Goodness-of-fit on F^2	1.114
Final R indexes [I>=2σ (I)]	R_1 = 0.0633, wR_2 = 0.1578
Final R indexes [all data]	R_1 = 0.0653, wR_2 = 0.1590
Largest diff. peak/hole / e Å^{-3}	0.44/-0.34
Flack parameter	0.0(3)
CCDC Number	2392123

Crystal structure: **41**

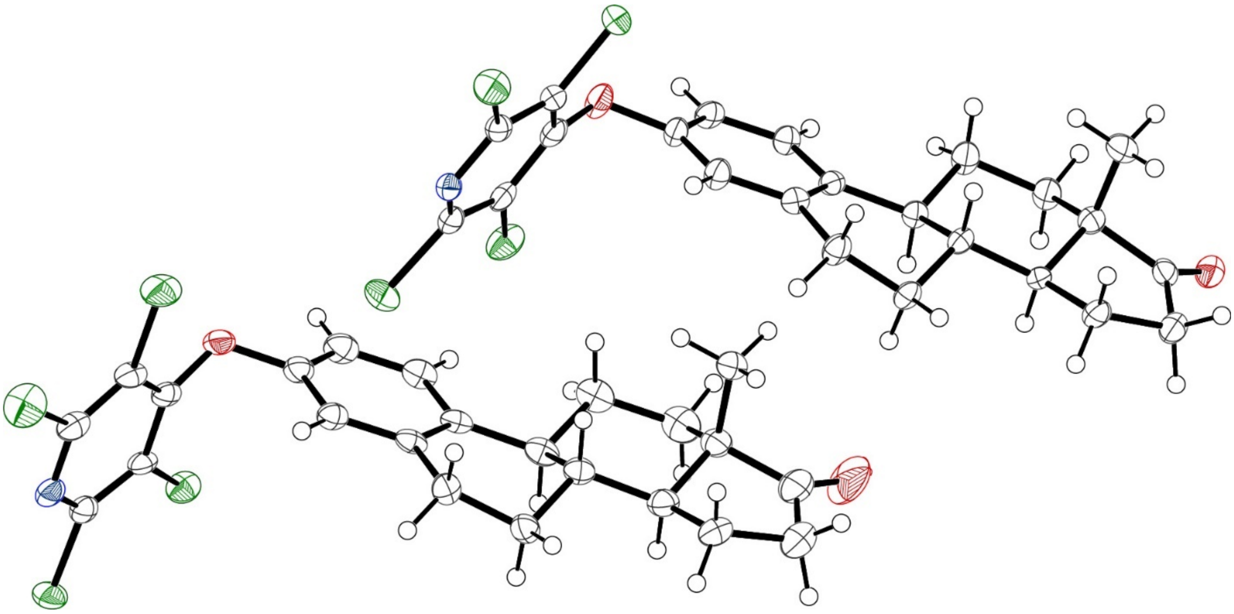

Empirical formula	$C_{48}H_{46}Cl_8N_2O_5$
Formula weight	1014.531
Temperature/K	120.00
Crystal system	orthorhombic
Space group	$P2_12_12_1$
a/Å	7.8593(3)
b/Å	12.9213(4)
c/Å	44.5444(15)
α/°	90
β/°	90
γ/°	90
Volume/Å^3	4523.6(3)
Z	4
ρ_{calc}g/cm^3	1.490
μ/mm^{-1}	0.549
F(000)	2101.7
Crystal size/mm^3	0.121 × 0.096 × 0.065
Radiation	MoKα (λ = 0.71073)
2Θ range for data collection/°	3.64 to 55
Index ranges	-11 ≤ h ≤ 11, -18 ≤ k ≤ 19, -65 ≤ l ≤ 65
Reflections collected	86112
Independent reflections	10394 [R_{int} = 0.1243, R_{sigma} = 0.1311]
Data/restraints/parameters	10394/522/543
Goodness-of-fit on F^2	1.062
Final R indexes [I>=2σ (I)]	R_1 = 0.0635, wR_2 = 0.1067
Final R indexes [all data]	R_1 = 0.0965, wR_2 = 0.1197
Largest diff. peak/hole / e Å^{-3}	0.61/-0.60
Flack parameter	0.01(3)
CCDC Number	2392124

Crystal structure: **42**

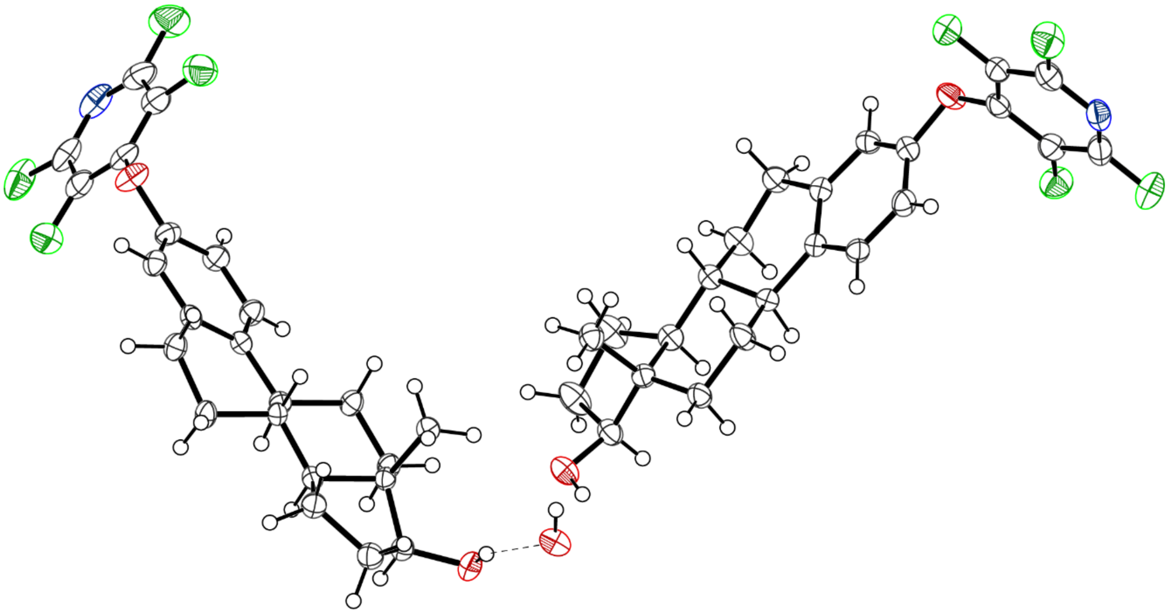

Empirical formula	$C_{92}H_{94}F_{16}N_4O_9$
Formula weight	1703.71
Temperature/K	120.00
Crystal system	monoclinic
Space group	C2
a/Å	34.3327(13)
b/Å	6.6021(3)
c/Å	23.8537(9)
α/°	90
β/°	132.8876(16)
γ/°	90
Volume/Å^3	3961.6(3)
Z	2
ρ_{calc}/cm^3	1.428
μ/mm^{-1}	0.996
F(000)	1780.0
Crystal size/mm^3	0.36 × 0.08 × 0.01
Radiation	Mo Kα (λ = 1.54178)
2Θ range for data collection/°	5.056 to 144.898
Index ranges	-42 ≤ h ≤ 42, -7 ≤ k ≤ 8, -29 ≤ l ≤ 29
Reflections collected	24165
Independent reflections	7239 [R_{int} = 0.0573, R_{sigma} = 0.0579]
Data/restraints/parameters	7239/1/734
Goodness-of-fit on F^2	1.027
Final R indexes [I>=2σ (I)]	R_1 = 0.0477, wR_2 = 0.1153
Final R indexes [all data]	R_1 = 0.0579, wR_2 = 0.1217
Largest diff. peak/hole / e Å^{-3}	0.25/-0.25
Flack parameter	-0.14(16)
CCDC Number	2392125
Notes	*Asymmetric unit exists as a half hydrate.*

Crystal structure: **43**

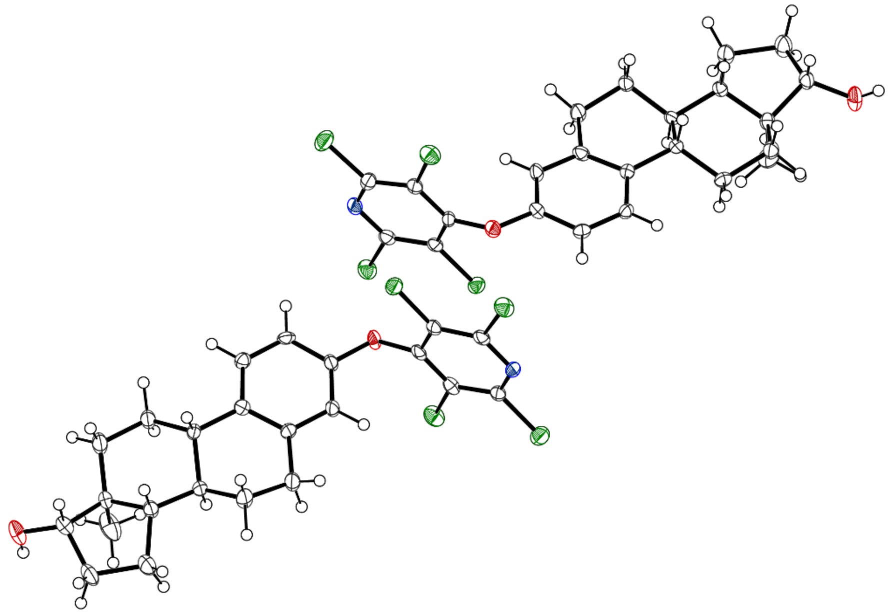

Empirical formula	$C_{23}H_{23}Cl_4NO_2$
Formula weight	487.22
Temperature/K	120.00
Crystal system	monoclinic
Space group	$P2_1$
a/Å	8.8203(3)
b/Å	8.1152(2)
c/Å	30.0843(10)
α/°	90
β/°	91.5990(10)
γ/°	90
Volume/Å^3	2152.55(12)
Z	4
ρ_{calc}g/cm^3	1.503
μ/mm^{-1}	0.571
F(000)	1008.0
Crystal size/mm^3	0.182 × 0.089 × 0.028
Radiation	Mo Kα (λ = 0.71073)
2Θ range for data collection/°	4.064 to 61.092
Index ranges	-12 ≤ h ≤ 12, -11 ≤ k ≤ 11, -42 ≤ l ≤ 42
Reflections collected	65705
Independent reflections	12928 [R_{int} = 0.0645, R_{sigma} = 0.0608]
Data/restraints/parameters	12928/1/551
Goodness-of-fit on F^2	1.101
Final R indexes [I>=2σ (I)]	R_1 = 0.0591, wR_2 = 0.0973
Final R indexes [all data]	R_1 = 0.0770, wR_2 = 0.1027
Largest diff. peak/hole / e Å^{-3}	0.41/-0.47
Flack parameter	-0.02(2)
CCDC Number	2392126

Computational details

Generation of interacting pairs

Each interacting pair of molecules from the crystal structure of a compound with the hydrogens normalized was generated from Mercury[6] as a mol2 file and converted to a Gaussian 16 input file in GaussView.[7]

Generation of output files

The Gaussian 16 package[8] was used to generate wavefunction files at the computationally-intensive and benchmark-accurate model chemistry MP2[9]/6-311G(d,p) for quantum theory of atoms in molecules (QTAIM) and non-covalent interactions-reduced density gradient (NCI-RDG) analyses and natural bond orbital (NBO) output files for NBO analyses.

Intermolecular interaction energies

The difference between the interacting pair total electronic energy and the sum of the total electronic energies of the two individual molecules was determined as the intermolecular interaction energy of the specific interacting pair (Tables SX). The computed MP2/6-311G(d,p) energies are based on gas-phase environments so favor non-polar pairs over polar pairs resulting in increased interaction energies for non-polar pairs and decreased interaction energies for polar pairs. Dipole moments for single molecules and interacting pairs are thus listed in the tables to aid our interpretation of the intermolecular interaction energy values.

QTAIM

QTAIM analyses for all pairs of molecules containing intermolecular interactions in the crystal structures were carried out with the wavefunction file .wfn [at MP2/6-311G(d,p)], obtained from Gaussian 16 as input in the AIMALL package[10] and selected parameters like Laplacian ($\nabla^2\rho$) and bond dissociation energy (D.Ev) at the bond critical points (BCPs) were calculated.

NCI-RDG

NCI-RDG analyses for all pairs of molecules were performed using AIMALL with the wavefunction file .wfx [at MP2/6-311G(d,p)] obtained from Gaussian 16 to generate the reduced density gradient (RDG) and ρ*sign(λ_2) cube files for isosurface figures.

NBO

NBO analyses were carried out on all pairs of molecules at MP2/6-311G(d,p) using NBO 3.1 by E. D. Glendening, A. E. Reed, J. E. Carpenter, and F. Weinhold[11] in Gaussian 16. The orbital donor-acceptor intermolecular interactions within pairs are assessed by second-order perturbation theory where the stabilization energies E(2) of delocalizations from the filled NBOs to the unoccupied/unfilled non-Lewis NBOs are estimated.

Table S3. Total electronic energies, intermolecular interaction energies and dipole moments of individual molecules and interacting pairs for tetrafluoropyridyl ethers **1**, **5**–**9**. Two independent molecules in a crystal structure are denoted as molecules A and B here.

Molecule/Pair	Total Energy a.u.	Intermolecular Interaction Energy kJ mol^{-1}	Dipole moment Debye
**1**			
Molecule	-988.53838		4.13
Pair 1	-1977.09565	49.58	7.81
Pair 2	-1977.08986	34.39	3.99
Pair 3	-1977.08708	27.10	5.14
Pair 4	-1977.07907	6.06	7.09
**5**			
Molecule	-1063.59041		3.71
Pair 1	-2127.20025	51.01	7.10
Pair 2	-2127.19679	41.92	5.42
Pair 3	-2127.19434	35.48	0.41
Pair 4	-2127.19213	29.68	6.53
Pair 5	-2127.18377	7.73	5.10
**6**			
Molecule	-949.33973		3.83
Pair 1	-1898.69519	41.30	7.25
Pair 2	-1898.69440	39.22	2.86
Pair 3	-1898.68831	23.24	3.96
Pair 4	-1898.68798	22.37	3.06
Pair 5	-1898.68286	8.91	6.69
**7**			
Molecule	-3521.24224		1.96
Pair 1	-7042.49904	38.24	3.59
Pair 2	-7042.49595	30.14	0.83
Pair 3	-7042.49571	29.50	0.00
Pair 4	-7042.49395	24.88	0.80
Pair 5	-7042.48860	10.84	0.00
Pair 6	-7042.48788	8.94	4.60
**8**			
Molecule	-1408.39484		1.88
Pair 1	-2816.80381	37.10	3.40
Pair 2	-2816.80100	29.71	0.87
Pair 3	-2816.80034	27.98	0.00
Pair 4	-2816.79930	25.24	0.87
Pair 5	-2816.79345	9.87	0.00
Pair 6	-2816.79242	7.17	4.20
**9**			
Molecule A	-1153.42816		2.75
Molecule B	-1153.42622		2.68
Pair 1	-2306.87477	53.54	5.24
Pair 2	-2306.87243	42.32	0.00
Pair 3	-2306.86480	32.45	2.82
Pair 4	-2306.86589	30.22	5.45
Pair 5	-2306.86525	28.56	2.55
Pair 6	-2306.86374	24.60	5.16
Pair 7	-2306.85926	17.89	5.12

Table S4. Total electronic energies, intermolecular interaction energies and dipole moments of individual molecules and interacting pairs for tetrafluoropyridyl thioethers **2**, **10–14**. Crystal structures that contain two independent molecules are listed as molecules A and B here.

Molecule/Pair	Total Energy a.u.	Intermolecular Interaction Energy kJ mol^{-1}	Dipole moment Debye
**2**			
Molecule A	-1311.14878		4.38
Molecule B	-1311.14719		4.17
Pair 1	-2622.31845	54.88	8.38
Pair 2	-2622.31457	53.01	7.98
Pair 3	-2622.30930	30.85	0.77
Pair 4	-2622.30877	29.45	0.96
Pair 5	-2622.30571	25.59	5.44
Pair 6	-2622.30141	18.46	0.88
**10**			
Molecule	-1386.20129		4.79
Pair 1	-2772.42285	53.22	8.98
Pair 2	-2772.41697	37.76	6.60
Pair 3	-2772.41581	34.74	0.79
Pair 4	-2772.41210	24.99	7.52
Pair 5	-2772.40355	2.54	0.36
**11**			
Molecule A	-1271.94940		3.92
Molecule B	-1271.94934		3.97
Pair 1	-2543.91617	45.63	7.57
Pair 2	-2543.91603	45.58	7.64
Pair 3	-2543.91038	30.74	0.60
Pair 4	-2543.90947	28.04	0.58
Pair 5	-2543.90488	16.14	4.49
**12**			
Molecule	-3843.85339		2.68
Pair 1	-7687.72953	59.71	0.00
Pair 2	-7687.72408	45.41	0.00
Pair 3	-7687.72159	38.88	4.96
Pair 4	-7687.71724	27.44	1.93
Pair 5	-7687.71438	19.93	0.00
Pair 6	-7687.71135	11.98	5.18
Pair 7	-7687.70881	5.32	0.00
**13**			
Molecule	-1731.00622		2.35
Pair 1	-3462.02594	35.45	3.96
Pair 2	-3462.02536	33.95	2.86
Pair 3	-3462.02457	31.86	2.18
Pair 4	-3462.01843	15.75	4.80
Pair 5	-3462.01342	2.59	1.01
**14**			
Molecule	-1476.03843		3.86
Pair 1	-2952.09506	47.81	7.61
Pair 2	-2952.08900	31.90	7.28
Pair 3	-2952.08675	25.99	6.76
Pair 4	-2952.08612	24.33	8.25
Pair 5	-2952.08482	20.92	8.79
Pair 6	-2952.08361	17.76	7.33

Table S5. Total electronic energies, intermolecular interaction energies and dipole moments of individual molecules and interacting pairs for tetrachloropyridyl ethers **3**, **16–17**, **18–19**. Crystal structures that contain two independent molecules are listed as molecules A and B here.

Molecule/Pair	Total Energy a.u.	Intermolecular Interaction Energy kJ mol^{-1}	Dipole moment Debye
**3**			
Molecule	-2428.47997		3.84
Pair 1	-4856.99152	82.92	0.00
Pair 2	-4856.97292	34.09	0.00
Pair 3	-4856.96990	26.17	0.00
Pair 4	-4856.96980	25.90	0.00
Pair 5	-4856.96496	13.19	7.46
Pair 6	-4856.96171	4.65	0.00
**16**			
Molecule A	-2848.33727		1.53
Molecule B	-2848.33687		1.49
Pair 1	-5696.70871	91.80	0.00
Pair 2	-5696.70627	85.38	0.00
Pair 3	-5696.69290	50.28	0.00
Pair 4	-5696.69259	49.47	0.00
Pair 5	-5696.68972	40.90	0.22
Pair 6	-5696.68162	20.67	2.87
Pair 7	-5696.67847	12.40	2.94
Pair 8	-5696.67545	3.43	0.38
**17**			
Molecule A	-4961.18126		1.86
Molecule B	-4961.17969		1.68
Pair 1	-9922.39692	90.34	0.00
Pair 2	-9922.39275	87.60	0.00
Pair 3	-9922.37804	48.97	0.00
Pair 4	-9922.38078	47.95	0.00
Pair 5	-9922.37260	30.59	0.80
Pair 6	-9922.36951	18.37	3.51
Pair 7	-9922.36237	7.85	3.27
Pair 8	-9922.36523	7.12	3.63
**18**			
Molecule A	-2593.37326		3.10
Molecule B	-2593.37124		2.95
Pair 1	-5186.78052	89.27	0.00
Pair 2	-5186.77535	86.34	0.00
Pair 3	-5186.76753	55.17	0.00
Pair 4	-5186.75747	34.05	4.96
Pair 5	-5186.75482	32.43	0.00
Pair 6	-5186.75693	27.33	0.00
Pair 7	-5186.74693	11.71	5.71
**19**			
Molecule	-2503.53258		4.44
Pair 1	-5007.09711	83.91	0.00
Pair 2	-5007.08363	48.52	0.00
Pair 3	-5007.07628	29.23	0.00

Pair 4	-5007.07400	23.22	6.83
Pair 5	-5007.07385	22.85	8.58
Pair 6	-5007.06960	11.69	0.00

Table S6. Total electronic energies, intermolecular interaction energies and dipole moments of individual molecules and interacting pairs for tetrachloropyridyl thioethers **4**, **20–22**. Crystal structures that contain two independent molecules are listed as molecules A and B here.

Molecule/Pair	Total Energy a.u.	Intermolecular Interaction Energy kJ mol^{-1}	Dipole moment Debye
**4**			
Molecule	-2751.08427		4.32
Pair 1	-5502.19844	78.51	0.00
Pair 2	-5502.18489	42.94	0.00
Pair 3	-5502.17836	25.78	0.00
Pair 4	-5502.17541	18.04	0.00
Pair 5	-5502.17307	11.89	8.37
Pair 6	-5502.17149	7.73	8.39
Pair 7	-5502.17048	5.10	9.00
**20**			
Molecule A	-3170.94192		1.53
Molecule B	-3170.94166		1.49
Pair 1	-6341.91705	87.22	0.00
Pair 2	-6341.91468	82.36	0.00
Pair 3	-6341.89661	33.54	0.71
Pair 4	-6341.89530	30.11	4.10
Pair 5	-6341.88600	5.68	4.33
Pair 6	-6341.88536	4.01	4.22
Pair 7	-6341.88524	3.70	0.27
**21**			
Molecule A	-5283.78905		2.15
Molecule B	-5283.78872		1.68
Pair 1	-10567.61104	86.49	0.00
Pair 2	-10567.60165	63.58	0.66
Pair 3	-10567.60154	61.53	0.00
Pair 4	-10567.59125	35.39	0.79
Pair 5	-10567.58952	30.86	0.46
Pair 6	-10567.58521	20.43	4.18
Pair 7	-10567.58066	8.47	4.19
Pair 8	-10567.58081	8.00	0.70
Pair 9	-10567.58098	7.57	4.10
Pair 10	-10567.57937	4.22	4.18
**22**			
Molecule	-2826.13395		4.96
Pair 1	-5652.28773	52.05	5.94
Pair 2	-5652.28739	51.16	9.20
Pair 3	-5652.28013	32.08	6.96
Pair 4	-5652.27910	29.39	1.53

QTAIM, NCI-RDG and NBO Analyses
Interacting pairs in compounds 1-4

Compound **1** pair 1

Total interaction energy = 49.6 kJ mol^{-1}

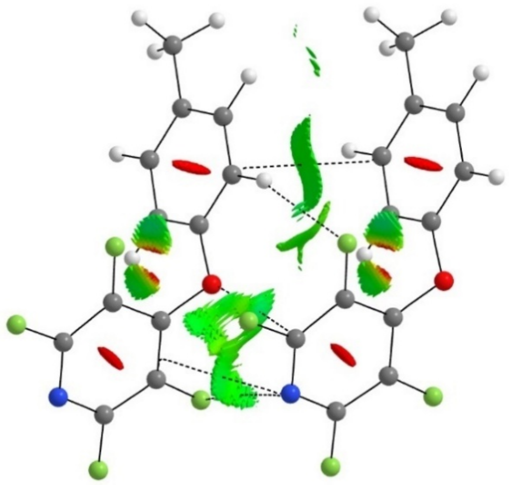

Figure S117. Bond critical paths (BCP) as dashed lines and reduced density gradient (RDG) isosurface (isovalue 0.5 mapped on a Blue-Green-Red color scale with values -0.02 < ρ*signλ$_2$ < 0.02 a.u.) in pair.

Table S7. Distances, dissociation energies and Laplacian of electron density (∇^{2}ρ) of bond critical paths (BCP) identified by QTAIM analysis.

BCP	BCP Distance Å	BCP Dissociation Energy kJ mol^{-1}	∇^{2}ρ e bohr^{-3}
O...C	2.972	7.7	0.0324
F...N	3.290	4.0	0.0183
C...N	3.440	3.3	0.0147
H...F	2.798	3.0	0.0146
C...C	3.499	2.9	0.0142

Table S8. Donor contribution and stabilization energies in Lewis donor-acceptor orbital interactions from NBO analysis. BD = bond, LP = lone pair.

Donor	D-A Stabilization Energy kJ mol^{-1}
BD C-C	9.5
LP O	4.1
BD N-C	2.1
LP F	1.1
BD O-C	0.6
BD F-C	0.5
LP N	0.3

Compound **1** pair 2

Total interaction energy = 34.4 kJ mol^{-1}

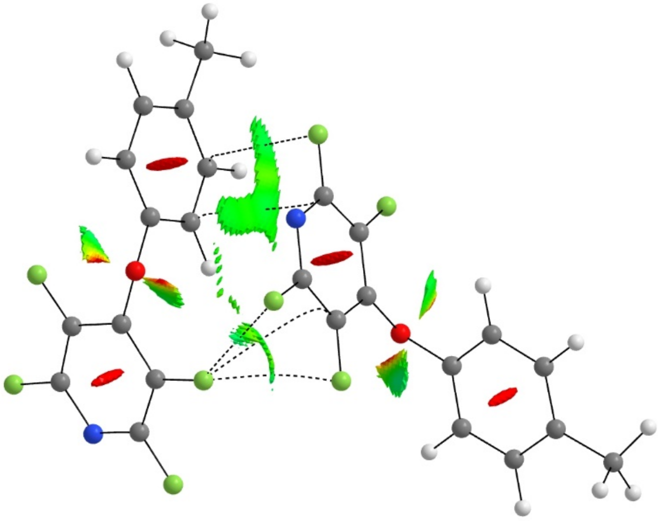

Figure S118. Bond critical paths (BCP) as dashed lines and reduced density gradient (RDG) isosurface (isovalue 0.5 mapped on a B-G-R color scale with values -0.02 < $\rho*\text{sign}\lambda_2$ < 0.02 a.u.) in pair.

Table S9. Distances, dissociation energies and Laplacian of electron density ($\nabla^2\rho$) of bond critical paths (BCP) identified by QTAIM analysis.

BCP	BCP Distance Å	BCP Dissociation Energy kJ mol^{-1}	$\nabla^2\rho$ e bohr^{-3}
F...F	2.940	6.9	0.0298
F...F	2.939	6.9	0.0299
F...C	3.096	5.4	0.0261
F...C	3.394	4.5	0.0197
C...C	3.489	3.7	0.0175

Table S10. Donor contribution and stabilization energies in Lewis donor-acceptor orbital interactions from NBO analysis. BD = bond, LP = lone pair.

Donor	D-A Stabilization Energy kJ mol^{-1}
BD C-C	8.0
LP F	3.3
BD N-C	1.7
BD F-C	0.4

Compound **1** pair 3

Total interaction energy = 27.1 kJ mol^{-1}

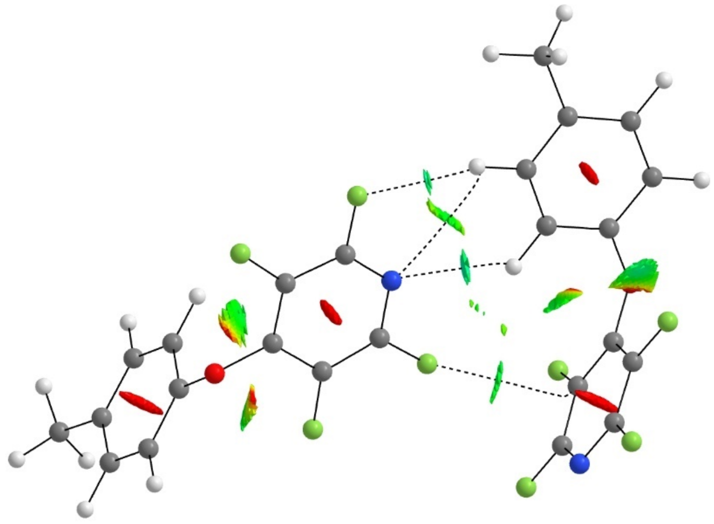

Figure S119. Bond critical paths (BCP) as dashed lines and reduced density gradient (RDG) isosurface (isovalue 0.5 mapped on a B-G-R color scale with values -0.02 < ρ*signλ$_2$ < 0.02 a.u.) in pair.

Table S11. Distances, dissociation energies and Laplacian of electron density ($\nabla^2\rho$) of bond critical paths (BCP) identified by QTAIM analysis.

BCP	BCP Distance Å	BCP Dissociation Energy kJ mol^{-1}	$\nabla^2\rho$ e bohr^{-3}
N...H	2.560	6.9	0.0301
H...F	2.583	5.2	0.0218
F...C	3.171	4.4	0.0208
N...H	3.018	3.2	0.0153

Table S12. Donor contribution and stabilization energies in Lewis donor-acceptor orbital interactions from NBO analysis. BD = bond, LP = lone pair.

Donor	D-A Stabilization Energy kJ mol^{-1}
LP N	3.8
LP F	3.6
BD C-H	0.9
BD N-C	0.8
BD C-C	0.6
BD F-C	0.3

Compound **1** pair 4

Total interaction energy = 6.1 kJ mol^{-1}

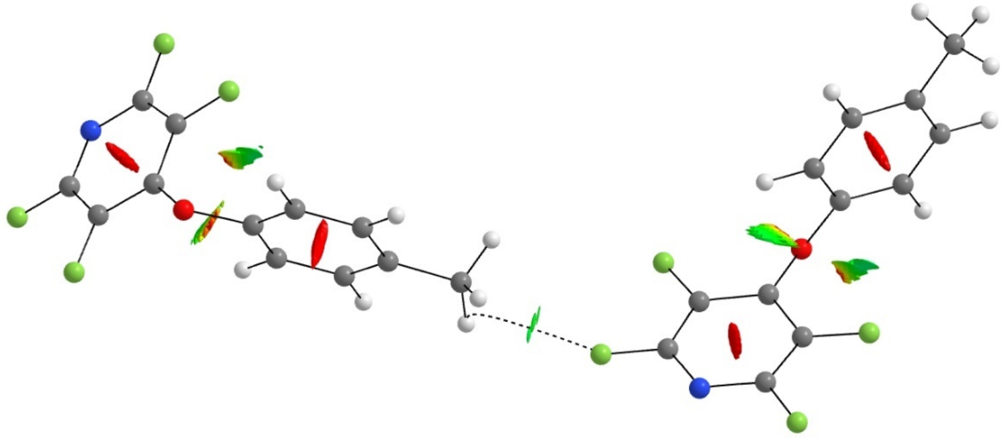

Figure S120. Bond critical paths (BCP) as dashed lines and reduced density gradient (RDG) isosurface (isovalue 0.5 mapped on a B-G-R color scale with values -0.02 < ρ*signλ_2 < 0.02 a.u.) in pair.

Table S13. Distances, dissociation energies and Laplacian of electron density ($\nabla^2\rho$) of bond critical paths (BCP) identified by QTAIM analysis.

BCP	BCP Distance Å	BCP Dissociation Energy kJ mol^{-1}	$\nabla^2\rho$ e bohr^{-3}
H…F	2.748	4.8	0.0236

Table S14. Donor contribution and stabilization energies in Lewis donor-acceptor orbital interactions from NBO analysis. BD = bond, LP = lone pair.

Donor	D-A Stabilization Energy kJ mol^{-1}
LP F	1.3
BD C-H	0.3

Compound **2** pair 1

Total interaction energy = 54.9 kJ mol^{-1}

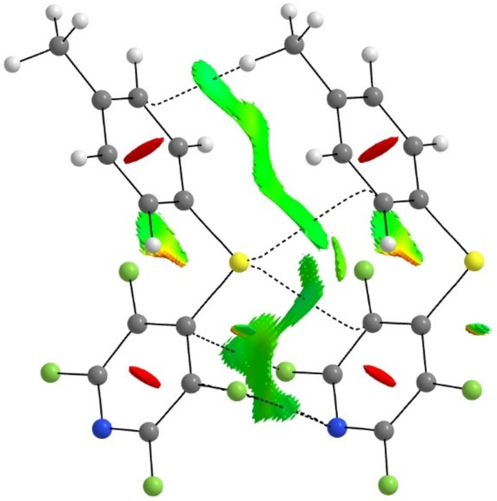

Figure S121. Bond critical paths (BCP) as dashed lines and reduced density gradient (RDG) isosurface (isovalue 0.5 mapped on a Blue-Green-Red color scale with values -0.02 < ρ*signλ_2 < 0.02 a.u.) in pair.

Table S15. Distances, dissociation energies and Laplacian of electron density ($\nabla^2\rho$) of bond critical paths (BCP) identified by QTAIM analysis.

BCP	BCP Distance Å	BCP Dissociation Energy kJ mol^{-1}	$\nabla^2\rho$ e bohr^{-3}
C...H	2.821	4.6	0.0193
S...C	3.482	4.4	0.0208
S...C	3.453	4.2	0.0214
F...N	3.277	4.0	0.0178
F...C	3.300	3.8	0.0178
C...N	3.516	2.6	0.0125

Table S16. Donor contribution and stabilization energies in Lewis donor-acceptor orbital interactions from NBO analysis. 3C = three center bond, BD = bond, LP = lone pair.

Donor	D-A Stabilization Energy kJ mol^{-1}
BD C-C	11.7
3C C-C-C	4.8
LP S	4.6
BD S-C	1.9
BD C-H	0.8
3C C-C-N	0.7
BD F-C	0.7
BD N-C	0.3
LP F	0.3

Compound **2** pair 2

Total interaction energy = 53.0 kJ mol^{-1}

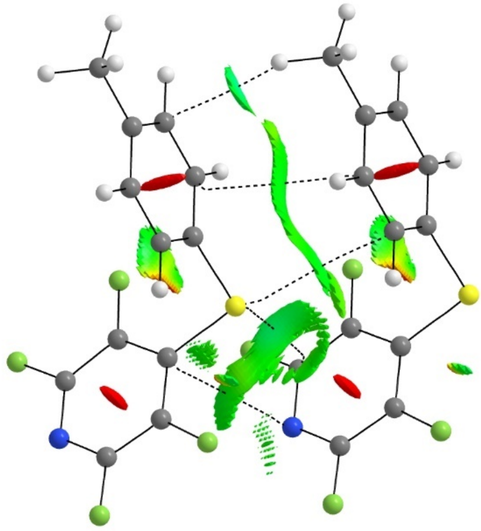

Figure S122. Bond critical paths (BCP) as dashed lines and reduced density gradient (RDG) isosurface (isovalue 0.5 mapped on a Blue-Green-Red color scale with values -0.02 < ρ*signλ_2 < 0.02 a.u.) in pair.

Table S17. Distances, dissociation energies and Laplacian of electron density ($\nabla^2\rho$) of bond critical paths (BCP) identified by QTAIM analysis.

BCP	BCP Distance Å	BCP Dissociation Energy kJ mol^{-1}	$\nabla^2\rho$ e $bohr^{-3}$
S...C	3.344	5.4	0.0272
C...H	2.665	5.2	0.0220
C...N	3.460	3.3	0.0146
S...C	3.602	2.9	0.0149
C...C	3.581	2.6	0.0118

Table S18. Donor contribution and stabilization energies in Lewis donor-acceptor orbital interactions from NBO analysis. BD = bond, LP = lone pair.

Donor	D-A Stabilization Energy kJ mol^{-1}
BD C-C	18.8
BD C-H	2.6
BD N-C	2.6
BD S-C	1.9
LP S	1.6

Compound **2** pair 3

Total interaction energy = 30.9 kJ mol^{-1}

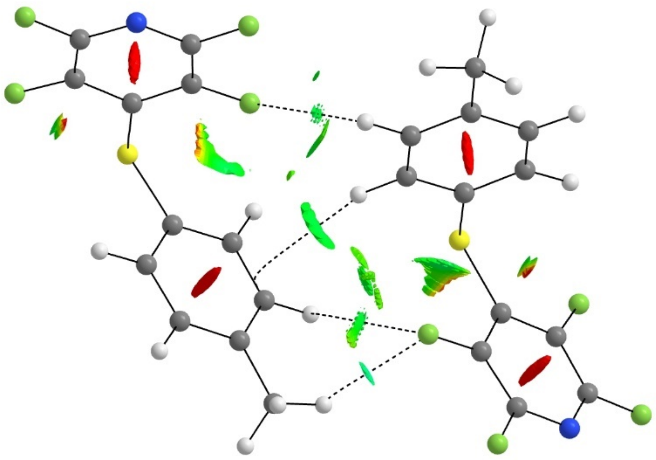

Figure S123. Bond critical paths (BCP) as dashed lines and reduced density gradient (RDG) isosurface (isovalue 0.5 mapped on a Blue-Green-Red color scale with values -0.02 < ρ*signλ_2 < 0.02 a.u.) in pair.

Table S19. Distances, dissociation energies and Laplacian of electron density ($\nabla^2\rho$) of bond critical paths (BCP) identified by QTAIM analysis.

BCP	BCP Distance Å	BCP Dissociation Energy kJ mol^{-1}	$\nabla^2\rho$ e bohr^{-3}
F...H	2.541	6.5	0.0263
F...H	2.807	3.6	0.0177
C...H	2.922	3.2	0.0139
F...H	2.897	3.0	0.0159

Table S20. Donor contribution and stabilization energies in Lewis donor-acceptor orbital interactions from NBO analysis. 3C = three center bond, BD = bond, LP = lone pair.

Donor	D-A Stabilization Energy kJ mol^{-1}
BD C-C	2.3
3C C-C-C	1.2
LP F	0.9
BD C-H	0.9
LP S	0.5
BD S-C	0.3

Compound **2** pair 4

Total interaction energy = 29.5 kJ mol^{-1}

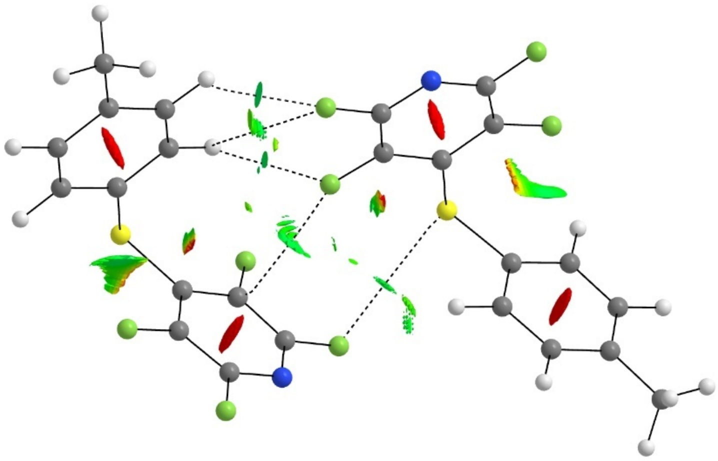

Figure S124. Bond critical paths (BCP) as dashed lines and reduced density gradient (RDG) isosurface (isovalue 0.5 mapped on a Blue-Green-Red color scale with values -0.02 < ρ*signλ_2 < 0.02 a.u.) in pair.

Table S21. Distances, dissociation energies and Laplacian of electron density ($\nabla^2\rho$) of bond critical paths (BCP) identified by QTAIM analysis.

BCP	BCP Distance Å	BCP Dissociation Energy kJ mol^{-1}	$\nabla^2\rho$ e bohr^{-3}
F…H	2.600	5.3	0.0237
F…C	3.103	4.8	0.0221
F…H	2.653	4.4	0.0190
F…H	2.839	3.4	0.0179
S…F	3.607	2.8	0.0156

Table S22. Donor contribution and stabilization energies in Lewis donor-acceptor orbital interactions from NBO analysis. BD = bond, LP = lone pair.

Donor	D-A Stabilization Energy kJ mol^{-1}
LP F	3.0
LP S	0.3

Compound **2** pair 5

Total interaction energy = 25.6 kJ mol^{-1}

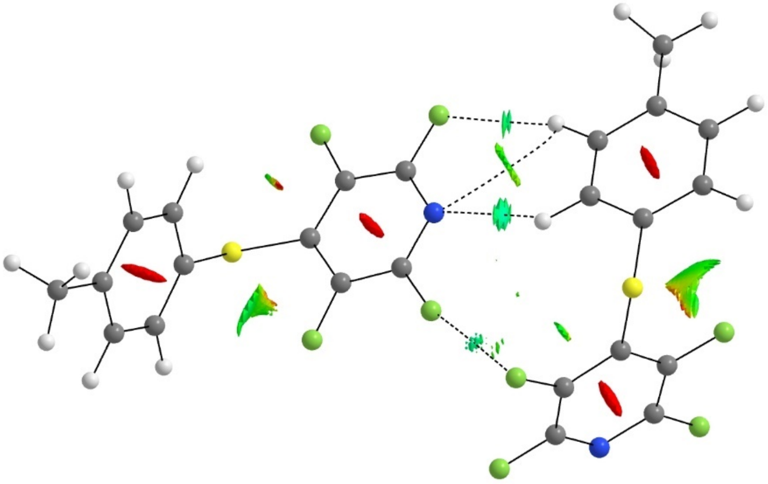

Figure S125. Bond critical paths (BCP) as dashed lines and reduced density gradient (RDG) isosurface (isovalue 0.5 mapped on a Blue-Green-Red color scale with values -0.02 < ρ*signλ_2 < 0.02 a.u.) in pair.

Table S23. Distances, dissociation energies and Laplacian of electron density ($\nabla^2\rho$) of bond critical paths (BCP) identified by QTAIM analysis.

BCP	BCP Distance Å	BCP Dissociation Energy kJ mol^{-1}	$\nabla^2\rho$ e bohr^{-3}
F…F	2.851	8.1	0.0330
N…H	2.640	5.8	0.0253
F…H	2.611	4.8	0.0206
N…H	3.064	2.9	0.0137

Table S24. Donor contribution and stabilization energies in Lewis donor-acceptor orbital interactions from NBO analysis. BD = bond, LP = lone pair.

Donor	D-A Stabilization Energy kJ mol^{-1}
LP F	2.9
LP N	2.6
BD C-C	0.5
BD C-H	0.5
BD N-C	0.4

Compound **2** pair 6

Total interaction energy = 18.5 kJ mol^{-1}

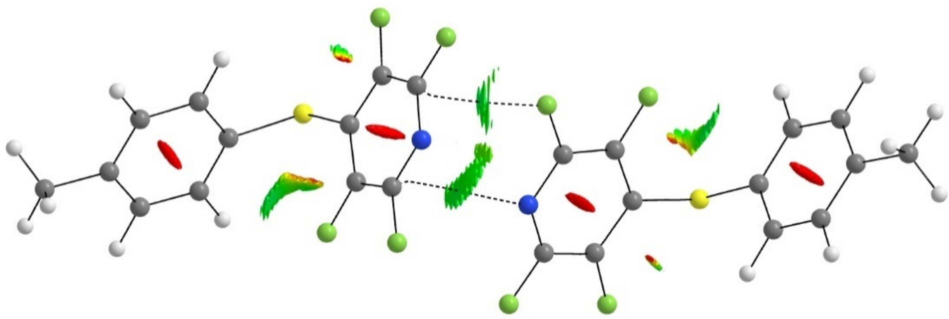

Figure S126. Bond critical paths (BCP) as dashed lines and reduced density gradient (RDG) isosurface (isovalue 0.5 mapped on a Blue-Green-Red color scale with values -0.02 < ρ*signλ$_2$ < 0.02 a.u.) in pair.

Table S25. Distances, dissociation energies and Laplacian of electron density ($\nabla^2\rho$) of bond critical paths (BCP) identified by QTAIM analysis.

BCP	BCP Distance Å	BCP Dissociation Energy kJ mol^{-1}	$\nabla^2\rho$ e bohr^{-3}
F…C	2.982	6.5	0.0294
N…C	3.391	3.4	0.0167

Table S26. Donor contribution and stabilization energies in Lewis donor-acceptor (D-A) orbital interactions from NBO analysis. BD = bond, LP = lone pair.

Donor	D-A Stabilization Energy kJ mol^{-1}
LP F	2.8
BD N-C	0.9
LP N	0.8
BD C-C	0.7

Compound **3** pair 1

Total interaction energy = 82.9 kJ mol^{-1}

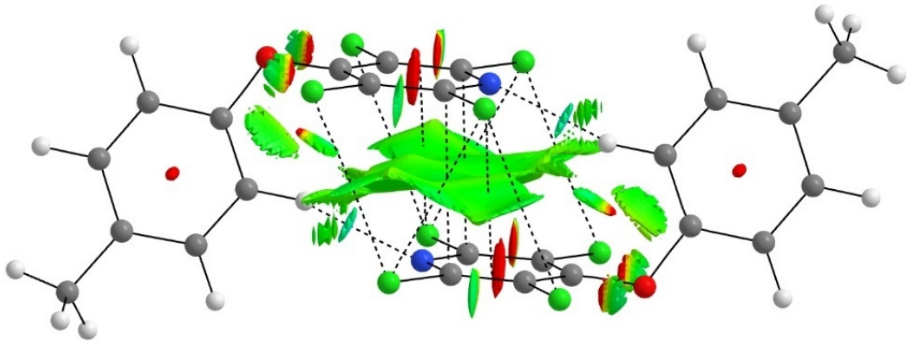

Figure S127. Bond critical paths (BCP) as dashed lines and reduced density gradient (RDG) isosurface (isovalue 0.5 mapped on a Blue-Green-Red color scale with values -0.02 < ρ*signλ$_2$ < 0.02 a.u.) in pair.

Table S27. Distances, dissociation energies and Laplacian of electron density ($\nabla^2\rho$) of bond critical paths (BCP) identified by QTAIM analysis.

BCP	BCP Distance Å	BCP Dissociation Energy kJ mol^{-1}	$\nabla^2\rho$ e bohr^{-3}
N...H	2.416	8.6	0.0378
N...H	2.416	8.6	0.0378
Cl...Cl	3.699	3.0	0.0185
Cl...Cl	3.699	3.0	0.0185
Cl...mid C-C	3.709	3.0	0.0154
Cl...mid C-C	3.709	3.0	0.0154
Cl...Cl	3.759	2.6	0.0165
Cl...Cl	3.759	2.6	0.0165
Cl...Cl	3.796	2.4	0.0151
Cl...Cl	3.796	2.4	0.0151

Table S28. Donor contribution and stabilization energies in Lewis donor-acceptor (D-A) orbital interactions from NBO analysis. BD = bond, LP = lone pair.

Donor	D-A Stabilization Energy kJ mol^{-1}
LP N	18.1
BD C-C	10.9
BD Cl-C	7.5
BD N-C	6.7
LP Cl	2.8
BD C-H	2.6

Compound **3** pair 2

Total interaction energy = 34.1 kJ mol^{-1}

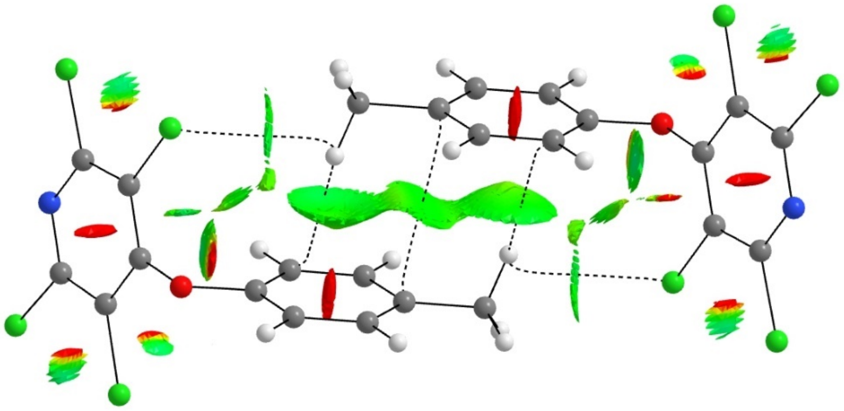

Figure S128. Bond critical paths (BCP) as dashed lines and reduced density gradient (RDG) isosurface (isovalue 0.5 mapped on a Blue-Green-Red color scale with values $-0.02 < \rho * \text{sign}\lambda_2 < 0.02$ a.u.) in pair.

Table S29. Distances, dissociation energies and Laplacian of electron density ($\nabla^2\rho$) of bond critical paths (BCP) identified by QTAIM analysis.

BCP	BCP Distance Å	BCP Dissociation Energy kJ mol^{-1}	$\nabla^2\rho$ e bohr^{-3}
C…H	2.880	4.4	0.0180
C…H	2.880	4.4	0.0180
Cl…H	3.134	3.2	0.0187
Cl…H	3.134	3.2	0.0187
C…C	3.733	2.4	0.0102

Table S30. Donor contribution and stabilization energies in Lewis donor-acceptor (D-A) orbital interactions from NBO analysis. BD = bond, LP = lone pair.

Donor	D-A Stabilization Energy kJ mol^{-1}
BD C-C	13.7
LP Cl	2.5
BD C-H	0.4

Compound **3** pair 3

Total interaction energy = 26.2 kJ mol^{-1}

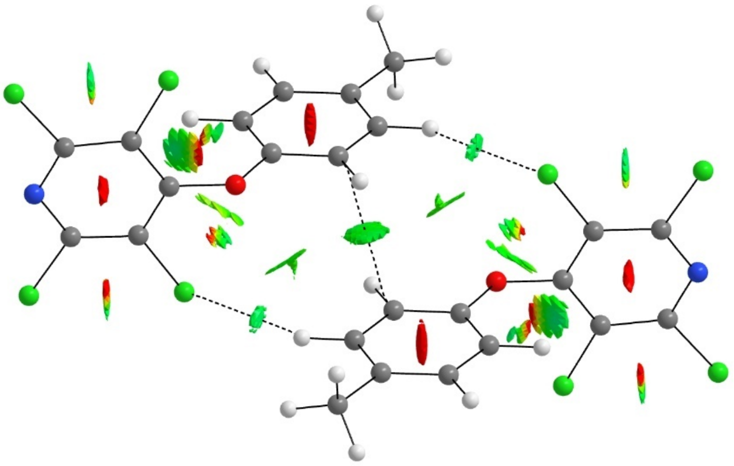

Figure S129. Bond critical paths (BCP) as dashed lines and reduced density gradient (RDG) isosurface (isovalue 0.5 mapped on a Blue-Green-Red color scale with values -0.02 < ρ*signλ_2 < 0.02 a.u.) in pair.

Table S31. Distances, dissociation energies and Laplacian of electron density ($\nabla^2\rho$) of bond critical paths (BCP) identified by QTAIM analysis.

BCP	BCP Distance Å	BCP Dissociation Energy kJ mol^{-1}	$\nabla^2\rho$ e bohr^{-3}
C...C	3.357	4.2	0.0227
Cl...H	2.973	3.3	0.0171
Cl...H	2.973	3.3	0.0171

Table S32. Donor contribution and stabilization energies in Lewis donor-acceptor (D-A) orbital interactions from NBO analysis. BD = bond, LP = lone pair.

Donor	D-A Stabilization Energy kJ mol^{-1}
BD C-C	6.6
LP Cl	3.5
BD C-H	2.5
BD Cl-C	0.4

Compound **3** pair 4

Total interaction energy = 25.9 kJ mol^{-1}

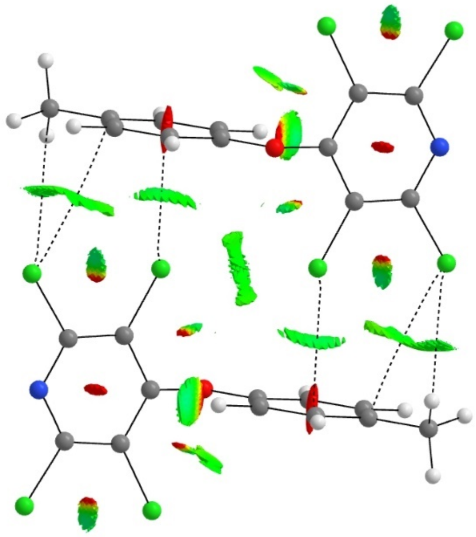

Figure S130. Bond critical paths (BCP) as dashed lines and reduced density gradient (RDG) isosurface (isovalue 0.5 mapped on a Blue-Green-Red color scale with values -0.02 < ρ*signλ_2 < 0.02 a.u.) in pair.

Table S33. Distances, dissociation energies and Laplacian of electron density ($\nabla^2\rho$) of bond critical paths (BCP) identified by QTAIM analysis.

BCP	BCP Distance Å	BCP Dissociation Energy kJ mol^{-1}	$\nabla^2\rho$ e bohr^{-3}
Cl…C	3.395	4.2	0.0217
Cl…H	3.350	2.1	0.0134
Cl…H	3.350	2.1	0.0134
Cl…C	3.843	1.9	0.0099
Cl…C	3.843	1.9	0.0099

Table S34. Donor contribution and stabilization energies in Lewis donor-acceptor (D-A) orbital interactions from NBO analysis. BD = bond, LP = lone pair.

Donor	D-A Stabilization Energy kJ mol^{-1}
LP Cl	4.2
BD C-C	2.3
BD C-H	1.1

Compound **3** pair 5

Total interaction energy = 13.2 kJ mol^{-1}

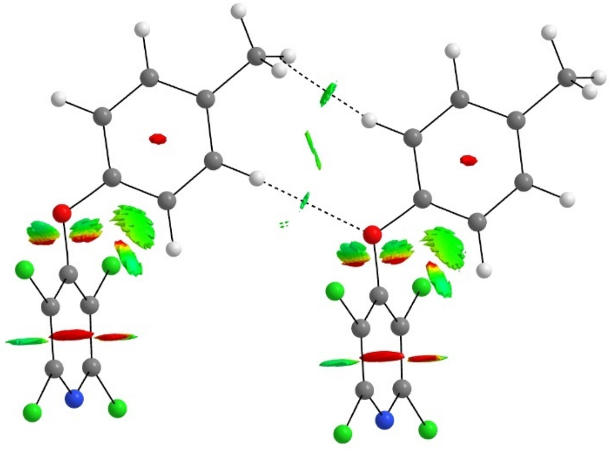

Figure S131. Bond critical paths (BCP) as dashed lines and reduced density gradient (RDG) isosurface (isovalue 0.5 mapped on a Blue-Green-Red color scale with values -0.02 < ρ*signλ_2 < 0.02 a.u.) in pair.

Table S35. Distances, dissociation energies and Laplacian of electron density ($\nabla^2\rho$) of bond critical paths (BCP) identified by QTAIM analysis.

BCP	BCP Distance Å	BCP Dissociation Energy kJ mol^{-1}	$\nabla^2\rho$ e $bohr^{-3}$
H...H	2.410	3.7	0.0183
O...H	2.863	3.2	0.0136

Table S36. Donor contribution and stabilization energies in Lewis donor-acceptor (D-A) orbital interactions from NBO analysis. BD = bond, LP = lone pair.

Donor	D-A Stabilization Energy kJ mol^{-1}
BD C-H	1.8
LP O	0.7

Compound **3** pair 6

Total interaction energy = 4.7 kJ mol^{-1}

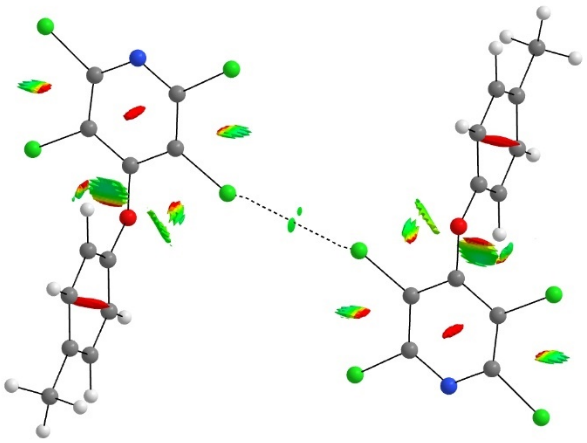

Figure S132. Bond critical paths (BCP) as dashed lines and reduced density gradient (RDG) isosurface (isovalue 0.5 mapped on a Blue-Green-Red color scale with values -0.02 < ρ*signλ$_2$ < 0.02 a.u.) in pair.

Table S37. Distances, dissociation energies and Laplacian of electron density ($\nabla^2\rho$) of bond critical paths (BCP) identified by QTAIM analysis.

BCP	BCP Distance Å	BCP Dissociation Energy kJ mol^{-1}	$\nabla^2\rho$ e bohr^{-3}
Cl…Cl	3.590	2.6	0.0178

Table S38. Donor contribution and stabilization energies in Lewis donor-acceptor (D-A) orbital interactions from NBO analysis. BD = bond, LP = lone pair.

Donor	D-A Stabilization Energy kJ mol^{-1}
LP Cl	2.8
BD Cl-C	0.6

Compound **4** pairs

Within in all pairs of compound **4**, there is an intramolecular BCP.

Table S39. Distance, dissociation energy and Laplacian of electron density ($\nabla^2\rho$) of intramolecular bond critical path (BCP) identified by QTAIM analysis for compound **4**.

BCP	BCP Distance Å	BCP Dissociation Energy kJ mol^{-1}	$\nabla^2\rho$ e $bohr^{-3}$
Intramolecular Cl…C	3.088	8.6	0.0454

Compound **4** pair 1

Total interaction energy = 78.5 kJ mol^{-1}

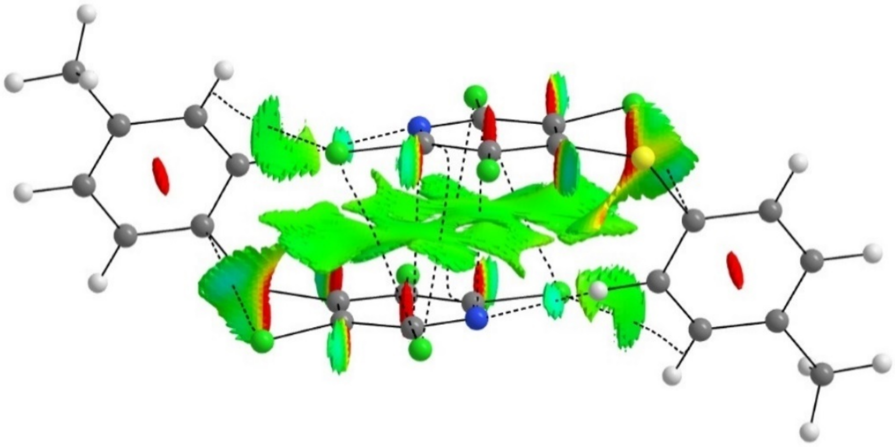

Figure S133. Bond critical paths (BCP) as dashed lines and reduced density gradient (RDG) isosurface (isovalue 0.5 mapped on a Blue-Green-Red color scale with values -0.02 < ρ*signλ_2 < 0.02 a.u.) in pair.

Table S40. Distances, dissociation energies and Laplacian of electron density ($\nabla^2\rho$) of bond critical paths (BCP) identified by QTAIM analysis.

BCP	BCP Distance Å	BCP Dissociation Energy kJ mol^{-1}	$\nabla^2\rho$ e bohr^{-3}
N…H	2.624	6.1	0.0255
N…H	2.624	6.1	0.0255
N…C	3.546	3.0	0.0132
N…C	3.546	3.0	0.0132
Cl…mid C-H	3.400	2.8	0.0165
Cl…mid C-H	3.400	2.8	0.0165
Cl…C	3.699	2.8	0.0143
Cl…C	3.699	2.8	0.0143
Cl…Cl	3.823	2.4	0.0144
Cl…Cl	3.823	2.4	0.0144

Table S41. Donor contribution and stabilization energies in Lewis donor-acceptor (D-A) orbital interactions from NBO analysis. BD = bond, LP = lone pair.

Donor	D-A Stabilization Energy kJ mol^{-1}
LP N	5.5
BD Cl-C	3.4
LP Cl	3.1
BD N-C	2.9
BD C-C	2.5
BD C-H	1.7

Compound **4** pair 2

Total interaction energy = 42.9 kJ mol^{-1}

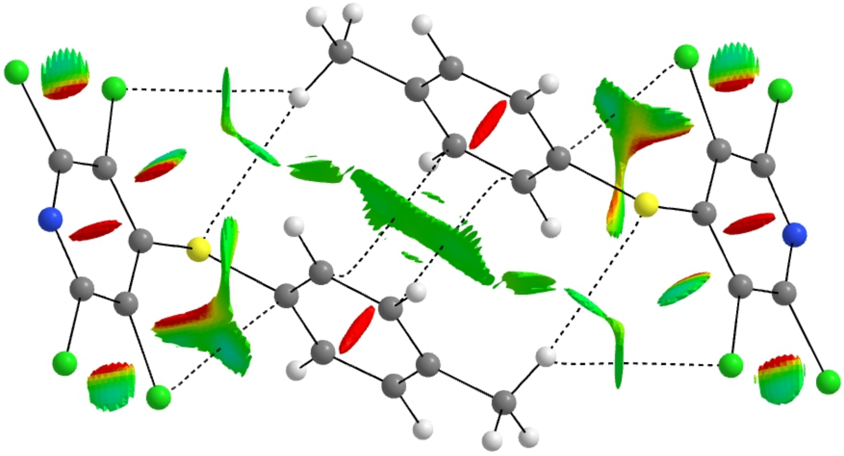

Figure S134. Bond critical paths (BCP) as dashed lines and reduced density gradient (RDG) isosurface (isovalue 0.5 mapped on a Blue-Green-Red color scale with values -0.02 < ρ*signλ$_2$ < 0.02 a.u.) in pair.

Table S42. Distances, dissociation energies and Laplacian of electron density ($\nabla^2\rho$) of bond critical paths (BCP) identified by QTAIM analysis.

BCP	BCP Distance Å	BCP Dissociation Energy kJ mol^{-1}	$\nabla^2\rho$ e bohr^{-3}
S…H	3.089	3.4	0.0158
S…H	3.089	3.4	0.0158
C…C	3.489	3.4	0.0151
C…C	3.489	3.4	0.0151
Cl…H	3.160	2.6	0.0141
Cl…H	3.160	2.6	0.0141

Table S43. Donor contribution and stabilization energies in Lewis donor-acceptor (D-A) orbital interactions from NBO analysis. BD = bond, LP = lone pair.

Donor	D-A Stabilization Energy kJ mol^{-1}
BD C-C	13.6
LP S	4.9
BD S-C	2.4
BD C-H	0.6
LP Cl	0.5
BD Cl-C	0.4

Compound **4** pair 3

Total interaction energy = 25.8 kJ mol^{-1}

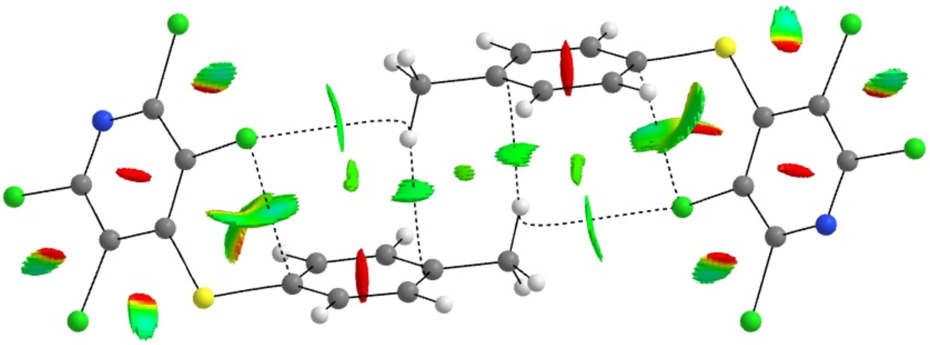

Figure S135. Bond critical paths (BCP) as dashed lines and reduced density gradient (RDG) isosurface (isovalue 0.5 mapped on a Blue-Green-Red color scale with values -0.02 < ρ*signλ$_2$ < 0.02 a.u.) in pair.

Table S44. Distances, dissociation energies and Laplacian of electron density ($\nabla^2\rho$) of bond critical paths (BCP) identified by QTAIM analysis.

BCP	BCP Distance Å	BCP Dissociation Energy kJ mol^{-1}	$\nabla^2\rho$ e bohr^{-3}
C…H	2.951	3.6	0.0143
C…H	2.951	3.6	0.0143
Cl…H	3.170	2.9	0.0179
Cl…H	3.170	2.9	0.0179

Table S45. Donor contribution and stabilization energies in Lewis donor-acceptor (D-A) orbital interactions from NBO analysis. BD = bond, LP = lone pair.

Donor	D-A Stabilization Energy kJ mol^{-1}
BD C-C	7.5
LP Cl	2.4
BD C-H	0.5
BD Cl-C	0.5

Compound **4** pair 4

Total interaction energy = 18.0 kJ mol^{-1}

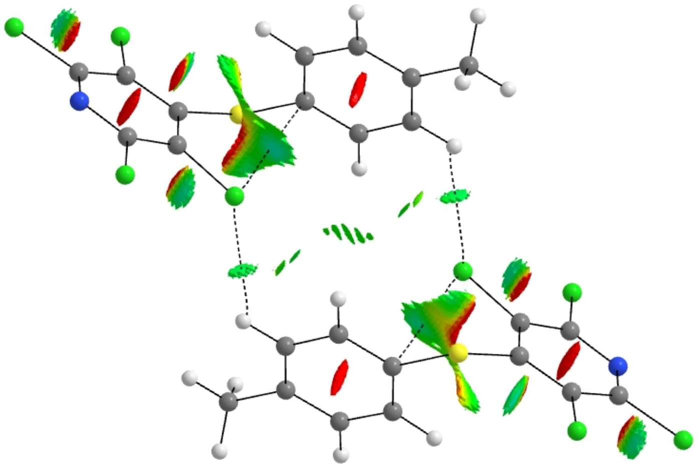

Figure S136. Bond critical paths (BCP) as dashed lines and reduced density gradient (RDG) isosurface (isovalue 0.5 mapped on a Blue-Green-Red color scale with values -0.02 < ρ*signλ$_2$ < 0.02 a.u.) in pair.

Table S46. Distances, dissociation energies and Laplacian of electron density ($\nabla^2\rho$) of bond critical paths (BCP) identified by QTAIM analysis.

BCP	BCP Distance Å	BCP Dissociation Energy kJ mol^{-1}	$\nabla^2\rho$ e bohr^{-3}
Cl...H	3.018	3.3	0.0177
Cl...H	3.018	3.3	0.0177

Table S47. Donor contribution and stabilization energies in Lewis donor-acceptor (D-A) orbital interactions from NBO analysis. BD = bond, LP = lone pair.

Donor	D-A Stabilization Energy kJ mol^{-1}
LP Cl	1.8
BD Cl-C	1.3
BD C-H	0.6

Compound **4** pair 5

Total interaction energy = 11.9 kJ mol^{-1}

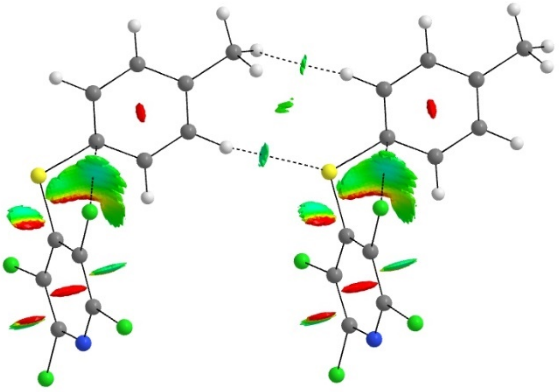

Figure S137. Bond critical paths (BCP) as dashed lines and reduced density gradient (RDG) isosurface (isovalue 0.5 mapped on a Blue-Green-Red color scale with values -0.02 < ρ*signλ_2 < 0.02 a.u.) in pair.

Table S48. Distances, dissociation energies and Laplacian of electron density ($\nabla^2\rho$) of bond critical paths (BCP) identified by QTAIM analysis.

BCP	BCP Distance Å	BCP Dissociation Energy kJ mol^{-1}	$\nabla^2\rho$ e $bohr^{-3}$
S…H	2.863	4.9	0.0223
H…H	2.422	3.3	0.0147

Table S49. Donor contribution and stabilization energies in Lewis donor-acceptor (D-A) orbital interactions from NBO analysis. BD = bond, LP = lone pair.

Donor	D-A Stabilization Energy kJ mol^{-1}
LP S	5.5
BD C-H	1.7

Compound **4** pair 6

Total interaction energy = 7.7 kJ mol^{-1}

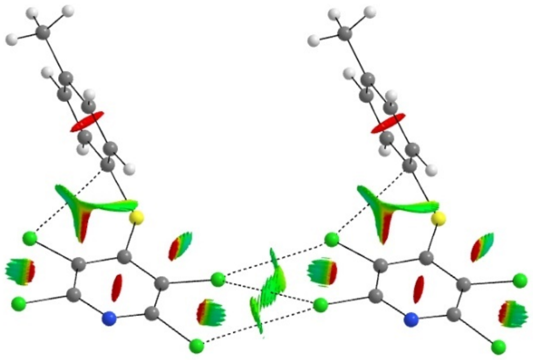

Figure S138. Bond critical paths (BCP) as dashed lines and reduced density gradient (RDG) isosurface (isovalue 0.5 mapped on a Blue-Green-Red color scale with values -0.02 < ρ*signλ_2 < 0.02 a.u.) in pair.

Table S50. Distances, dissociation energies and Laplacian of electron density ($\nabla^2\rho$) of bond critical paths (BCP) identified by QTAIM analysis.

BCP	BCP Distance Å	BCP Dissociation Energy kJ mol^{-1}	$\nabla^2\rho$ e bohr^{-3}
Cl…Cl	3.407	5.2	0.0302
Cl…Cl	3.736	2.5	0.0170
Cl…Cl	3.685	2.4	0.0167

Table S51. Donor contribution and stabilization energies in Lewis donor-acceptor (D-A) orbital interactions from NBO analysis. BD = bond, LP = lone pair.

Donor	D-A Stabilization Energy kJ mol^{-1}
LP Cl	7.2
BD Cl-C	0.7

Compound **4** pair 7

Total interaction energy = 5.1 kJ mol^{-1}

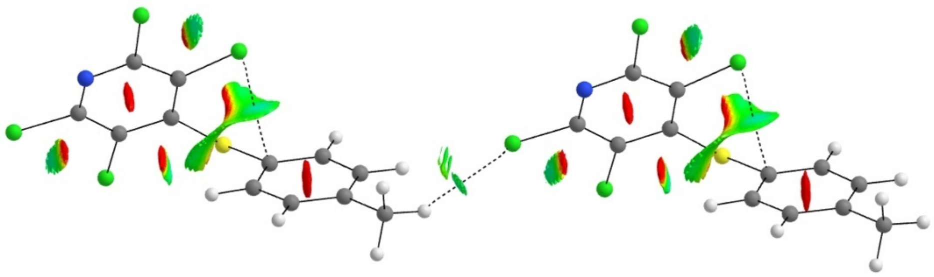

Figure S139. Bond critical paths (BCP) as dashed lines and reduced density gradient (RDG) isosurface (isovalue 0.5 mapped on a Blue-Green-Red color scale with values -0.02 < ρ*signλ_2 < 0.02 a.u.) in pair.

Table S52. Distances, dissociation energies and Laplacian of electron density ($\nabla^2\rho$) of bond critical paths (BCP) identified by QTAIM analysis.

BCP	BCP Distance Å	BCP Dissociation Energy kJ mol^{-1}	$\nabla^2\rho$ e $bohr^{-3}$
Cl...H	2.889	4.0	0.0200

Table S53. Donor contribution and stabilization energies in Lewis donor-acceptor (D-A) orbital interactions from NBO analysis. BD = bond, LP = lone pair.

Donor	D-A Stabilization Energy kJ mol^{-1}
BD C-H	1.9
LP Cl	1.2
BD Cl-C	1.1

Pairs with F…F Interactions

Compound **5** pair 2 containing F…F interactions

Total interaction energy = 41.9 kJ mol^{-1}

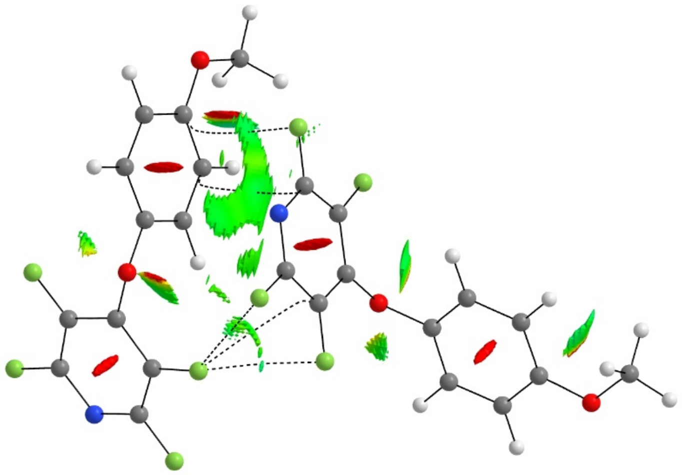

Figure S140. Bond critical paths (BCP) as dashed lines and reduced density gradient (RDG) isosurface (isovalue 0.5 mapped on a Blue-Green-Red color scale with values -0.02 < ρ*signλ_2 < 0.02 a.u.) in pair.

Table S54. Distances, dissociation energies and Laplacian of electron densities ($\nabla^2\rho$) of F…F bond critical paths (BCP) identified by QTAIM analysis and corresponding donor and acceptor contributions and stabilization energies in Lewis donor-acceptor orbital interactions from NBO analysis. LP = lone pair, BD* = anti-bonding.

Interaction	Distance Å	BCP Dissociation Energy kJ mol^{-1}	$\nabla^2\rho$ e $bohr^{-3}$	Donor	Acceptor	D-A Stabilization Energy kJ mol^{-1}
F…F	2.895	7.7	0.0315	LP F	BD* C-C	0.79
F…F	3.046	5.2	0.0241	LP F	BD* C-C	0.42

Compound **1** pair 2 containing F…F interactions

Total interaction energy = 34.4 kJ mol^{-1}

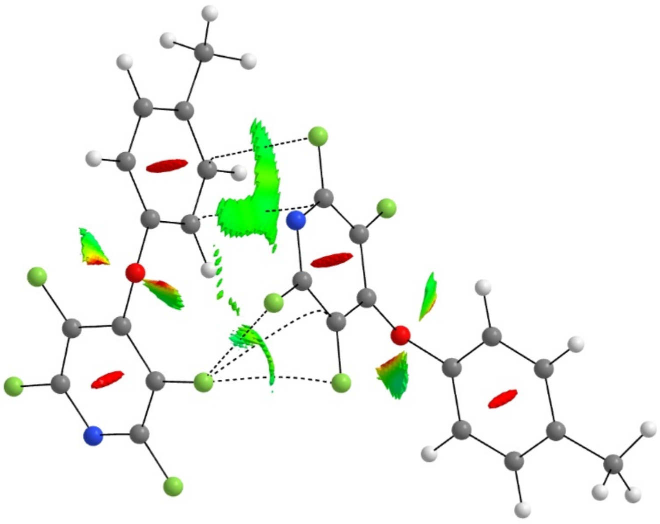

Figure S141. Bond critical paths (BCP) as dashed lines and reduced density gradient (RDG) isosurface (isovalue 0.5 mapped on a Blue-Green-Red color scale with values -0.02 < ρ*signλ$_2$ < 0.02 a.u.) in pair.

Table S55. Distances, dissociation energies and Laplacian of electron densities ($\nabla^2\rho$) of F…F bond critical paths (BCP) identified by QTAIM analysis and corresponding donor and acceptor contributions and stabilization energies in Lewis donor-acceptor orbital interactions from NBO analysis. LP = lone pair, BD* = anti-bonding.

Interaction	Distance Å	BCP Dissociation Energy kJ mol^{-1}	$\nabla^2\rho$ e bohr^{-3}	Donor	Acceptor	D-A Stabilization Energy kJ mol^{-1}
F…F	2.940	6.9	0.0298	LP F	BD* C-C	0.79
F…F	2.939	6.9	0.0299	LP F	BD* C-C	0.67

Compound **6** pair 2 containing F…F interaction

Total interaction energy = 39.2 kJ mol^{-1}

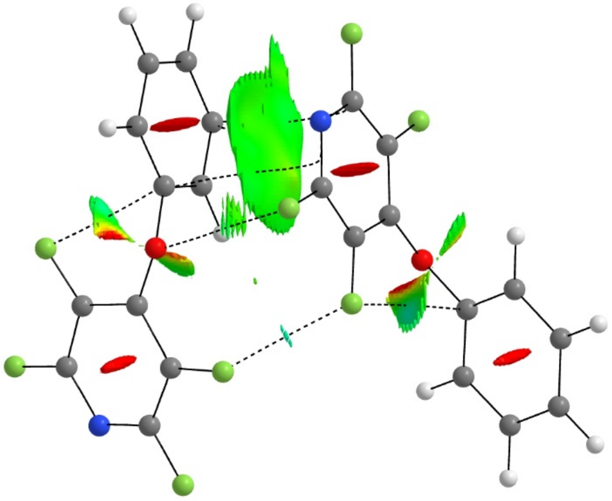

Figure S142. Bond critical paths (BCP) as dashed lines and reduced density gradient (RDG) isosurface (isovalue 0.5 mapped on a Blue-Green-Red color scale with values -0.02 < ρ*signλ_2 < 0.02 a.u.) in pair.

Table S56. Distance, dissociation energy and Laplacian of electron density ($\nabla^2\rho$) of F…F bond critical path (BCP) identified by QTAIM analysis and corresponding donor and acceptor contributions and stabilization energy in Lewis donor-acceptor orbital interaction from NBO analysis. LP = lone pair, BD* = anti-bonding.

Interaction	Distance Å	BCP Dissociation Energy kJ mol^{-1}	$\nabla^2\rho$ e bohr^{-3}	Donor	Acceptor	D-A Stabilization Energy kJ mol^{-1}
F…F	2.816	4.4	0.0200	LP F	BD* C-C	0.50

Compound **8** pair 1 containing F…F interactions

Total interaction energy = 37.1 kJ mol^{-1}

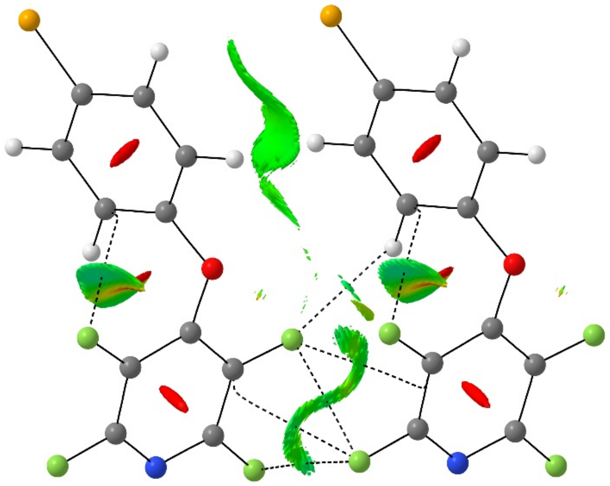

Figure S143. Bond critical paths (BCP) as dashed lines and reduced density gradient (RDG) isosurface (isovalue 0.5 mapped on a Blue-Green-Red color scale with values -0.02 < ρ*signλ_2 < 0.02 a.u.) in pair.

Table S57. Distances, dissociation energies and Laplacian of electron densities ($\nabla^2\rho$) of F…F bond critical paths (BCP) identified by QTAIM analysis and corresponding donor and acceptor contributions and stabilization energies in Lewis donor-acceptor orbital interactions from NBO analysis. LP = lone pair, BD* = anti-bonding.

Interaction	Distance Å	BCP Dissociation Energy kJ mol^{-1}	$\nabla^2\rho$ e $bohr^{-3}$	Donor	Acceptor	D-A Stabilization Energy kJ mol^{-1}
F…F	2.940	7.9	0.0327	LP F	BD* C-C	0.79
F…F	2.981	6.6	0.0287	LP F	BD* C-C	0.56
F…F	3.015	-	-	LP F	BD* C-C	0.67

Compound **8** pair 4 containing F…F interaction

Total interaction energy = 25.2 kJ mol^{-1}

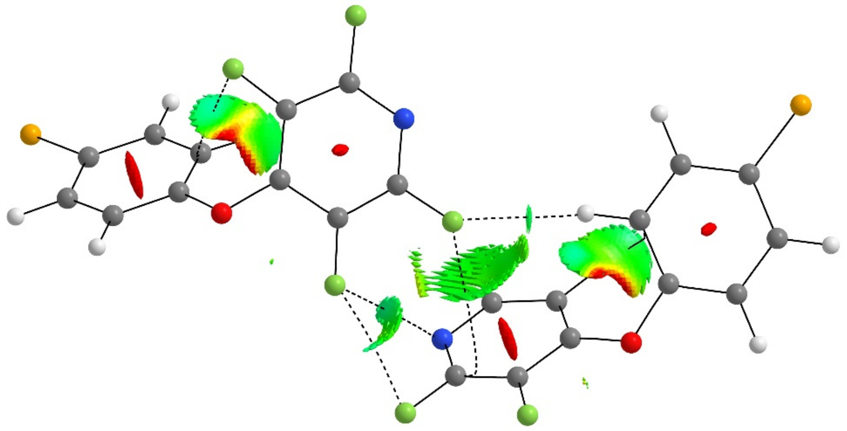

Figure S144. Bond critical paths (BCP) as dashed lines and reduced density gradient (RDG) isosurface (isovalue 0.5 mapped on a Blue-Green-Red color scale with values -0.02 < ρ*signλ_2 < 0.02 a.u.) in pair.

Table S58. Distance, dissociation energy and Laplacian of electron density ($\nabla^2\rho$) of F…F bond critical path (BCP) identified by QTAIM analysis and corresponding donor and acceptor contributions and stabilization energy in Lewis donor-acceptor orbital interaction from NBO analysis. LP = lone pair, BD* = anti-bonding.

Interaction	Distance Å	BCP Dissociation Energy kJ mol^{-1}	$\nabla^2\rho$ e bohr^{-3}	Donor	Acceptor	D-A Stabilization Energy kJ mol^{-1}
F…F	2.902	7.4	0.0316	LP F	BD* C-C	0.75

Compound **7** pair 1 containing F…F interactions

Total interaction energy = 38.2 kJ mol^{-1}

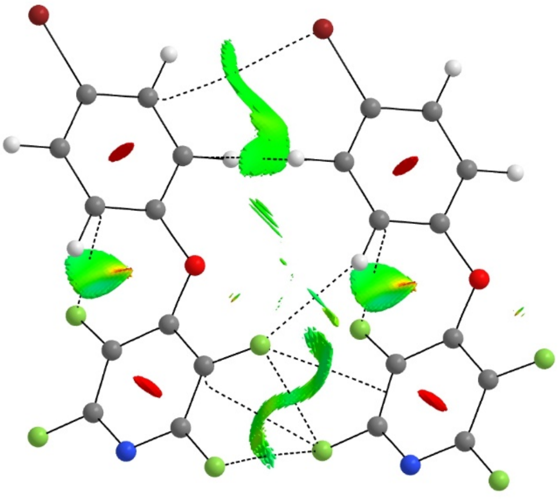

Figure S145. Bond critical paths (BCP) as dashed lines and reduced density gradient (RDG) isosurface (isovalue 0.5 mapped on a Blue-Green-Red color scale with values -0.02 < ρ*signλ$_2$ < 0.02 a.u.) in pair.

Table S59. Distances, dissociation energies and Laplacian of electron densities ($\nabla^2\rho$) of F…F bond critical paths (BCP) identified by QTAIM analysis and corresponding donor and acceptor contributions and stabilization energies in Lewis donor-acceptor orbital interactions from NBO analysis. LP = lone pair, BD* = anti-bonding.

Interaction	Distance Å	BCP Dissociation Energy kJ mol^{-1}	$\nabla^2\rho$ e bohr^{-3}	Donor	Acceptor	D-A Stabilization Energy kJ mol^{-1}
F…F	2.917	8.5	0.0341	LP F	BD* C-C	0.71
F…F	2.999	6.4	0.0258	LP F	BD* C-C	0.54
F…F	3.009	-	-	LP F	BD* C-C	0.67

Compound **7** pair 4 containing F…F interaction

Total interaction energy = 24.9 kJ mol^{-1}

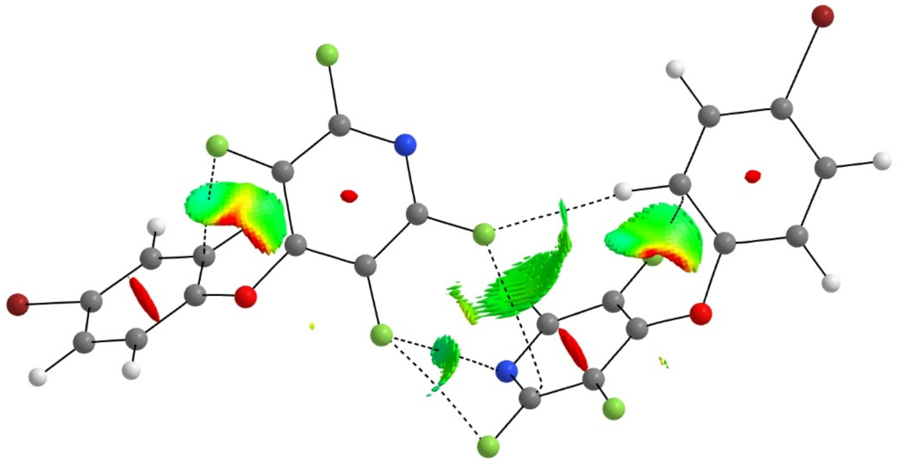

Figure S146. Bond critical paths (BCP) as dashed lines and reduced density gradient (RDG) isosurface (isovalue 0.5 mapped on a Blue-Green-Red color scale with values -0.02 < ρ*signλ$_2$ < 0.02 a.u.) in pair.

Table S60. Distance, dissociation energy and Laplacian of electron density ($\nabla^2\rho$) of F…F bond critical path (BCP) identified by QTAIM analysis and corresponding donor and acceptor contributions and stabilization energy in Lewis donor-acceptor orbital interaction from NBO analysis. LP = lone pair, BD* = anti-bonding.

Interaction	Distance Å	BCP Dissociation Energy kJ mol^{-1}	$\nabla^2\rho$ e bohr^{-3}	Donor	Acceptor	D-A Stabilization Energy kJ mol^{-1}
F…F	2.886	7.8	0.0325	LP F	BD* C-C	0.79

Compound **9** pair 6 containing F...F interactions

Total interaction energy = 24.6 kJ mol^{-1}

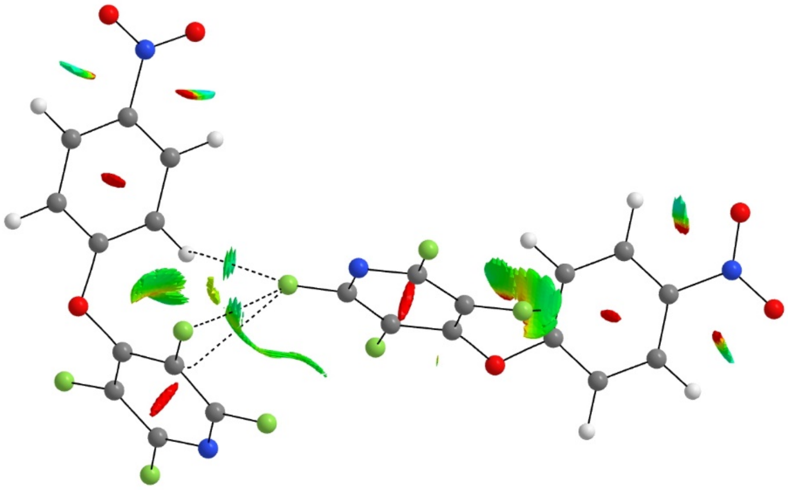

Figure S147. Bond critical paths (BCP) as dashed lines and reduced density gradient (RDG) isosurface (isovalue 0.5 mapped on a Blue-Green-Red color scale with values -0.02 < ρ*signλ_2 < 0.02 a.u.) in pair.

Table S61. Distances, dissociation energy and Laplacian of electron density ($\nabla^2\rho$) of F...F bond critical path (BCP) identified by QTAIM analysis and corresponding donor and acceptor contributions and stabilization energies in Lewis donor-acceptor orbital interactions from NBO analysis. LP = lone pair, BD* = anti-bonding.

Interaction	Distance Å	BCP Dissociation Energy kJ mol^{-1}	$\nabla^2\rho$ e bohr^{-3}	Donor	Acceptor	D-A Stabilization Energy kJ mol^{-1}
F...F	2.836	9.9	0.0397	LP F	BD* C-C	1.38
F...F	3.036	-	-	LP F	BD* C-C	0.54

Compound **10** pair 5 containing F…F interaction

Total interaction energy = 2.5 kJ mol^{-1}

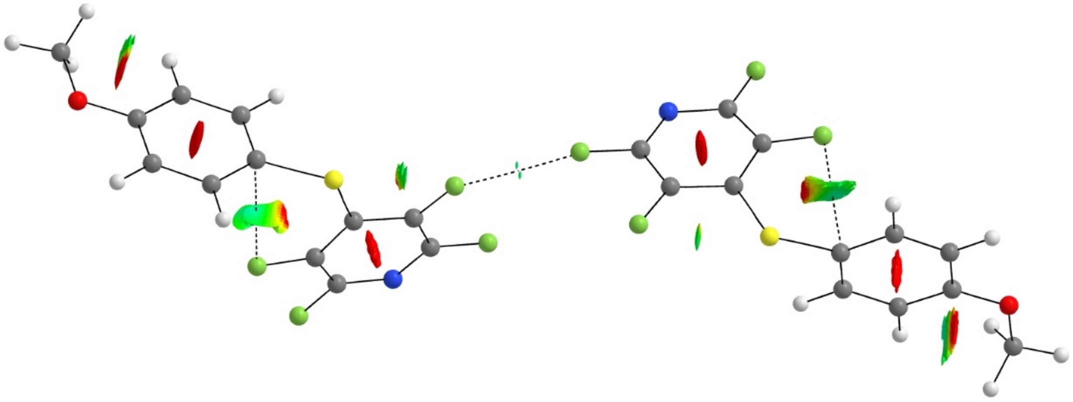

Figure S148. Bond critical paths (BCP) as dashed lines and reduced density gradient (RDG) isosurface (isovalue 0.5 mapped on a Blue-Green-Red color scale with values -0.02 < ρ*signλ$_2$ < 0.02 a.u.) in pair.

Table S62. Distance, dissociation energy and Laplacian of electron density ($\nabla^2\rho$) of F…F bond critical path (BCP) identified by QTAIM analysis. No donor-acceptor interaction found with NBO analysis.

Interaction	Distance Å	BCP Dissociation Energy kJ mol^{-1}	$\nabla^2\rho$ e bohr^{-3}	Donor	Acceptor	D-A Stabilization Energy kJ mol^{-1}
F…F	2.853	7.1	0.0305	-	-	-

Compound **2** pair 5 containing F…F interaction

Total interaction energy = 25.6 kJ mol^{-1}

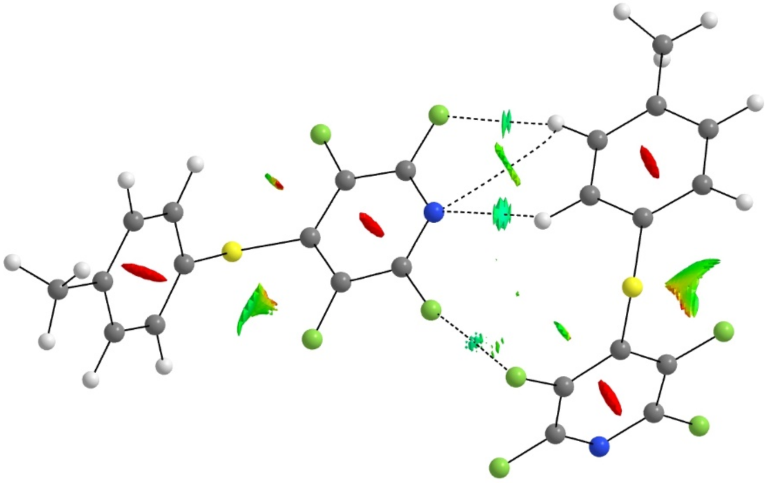

Figure S149. Bond critical paths (BCP) as dashed lines and reduced density gradient (RDG) isosurface (isovalue 0.5 mapped on a Blue-Green-Red color scale with values -0.02 < ρ*signλ_2 < 0.02 a.u.) in pair.

Table S63. Distance, dissociation energy and Laplacian of electron density ($\nabla^2\rho$) of F…F bond critical path (BCP) identified by QTAIM analysis and corresponding donor and acceptor contributions and stabilization energy in Lewis donor-acceptor orbital interaction from NBO analysis. LP = lone pair, BD* = anti-bonding.

Interaction	Distance Å	BCP Dissociation Energy kJ mol^{-1}	$\nabla^2\rho$ e bohr^{-3}	Donor	Acceptor	D-A Stabilization Energy kJ mol^{-1}
F…F	2.851	8.1	0.0330	LP F	BD* C-C	0.88

Compound **11** pair 3 containing F…F interaction

Total interaction energy = 30.7 kJ mol^{-1}

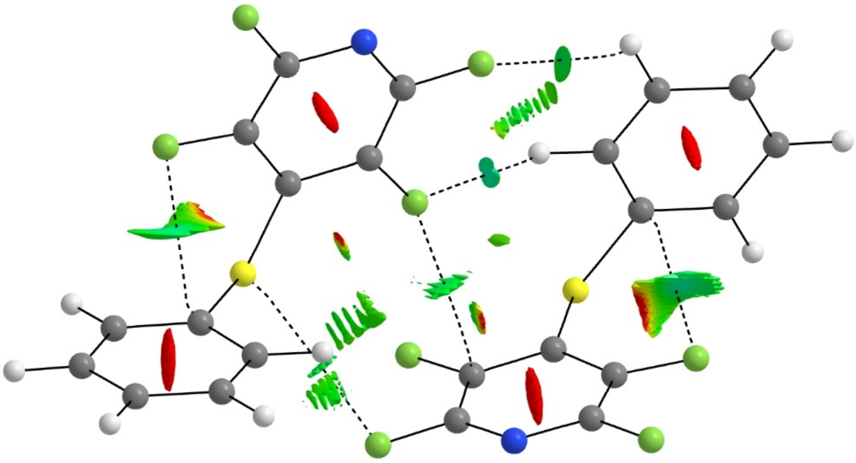

Figure S150. Bond critical paths (BCP) as dashed lines and reduced density gradient (RDG) isosurface (isovalue 0.5 mapped on a Blue-Green-Red color scale with values -0.02 < ρ*signλ$_2$ < 0.02 a.u.) in pair.

Table S64. Distance, stabilization energy in Lewis donor-acceptor orbital interaction from NBO analysis. LP = lone pair, BD* = anti-bonding. BCP for F…F not located.

Interaction	Distance Å	BCP Dissociation Energy kJ mol^{-1}	$\nabla^2\rho$ e bohr^{-3}	Donor	Acceptor	D-A Stabilization Energy kJ mol^{-1}
F…F	3.202	-	-	LP F	BD* C-C	0.29

Compound **13** pair 1 containing F…F interaction

Total interaction energy = 35.5 kJ mol^{-1}

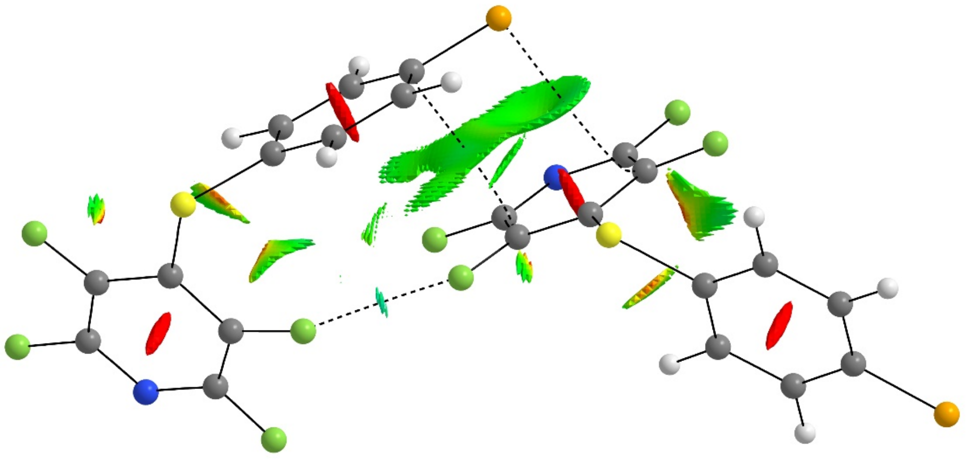

Figure S151. Bond critical paths (BCP) as dashed lines and reduced density gradient (RDG) isosurface (isovalue 0.5 mapped on a Blue-Green-Red color scale with values -0.02 < ρ*signλ$_2$ < 0.02 a.u.) in pair.

Table S65. Distance, dissociation energy and Laplacian of electron density ($\nabla^2\rho$) of F…F bond critical path (BCP) identified by QTAIM analysis and corresponding donor and acceptor contributions and stabilization energy in Lewis donor-acceptor orbital interaction from NBO analysis. LP = lone pair, BD* = anti-bonding.

Interaction	Distance Å	BCP Dissociation Energy kJ mol^{-1}	$\nabla^2\rho$ e bohr^{-3}	Donor	Acceptor	D-A Stabilization Energy kJ mol^{-1}
F…F	2.780	9.3	0.369	LP F	BD* C-C	0.71

Compound **12** pair 3 containing F…F interactions

Total interaction energy = 38.9 kJ mol^{-1}

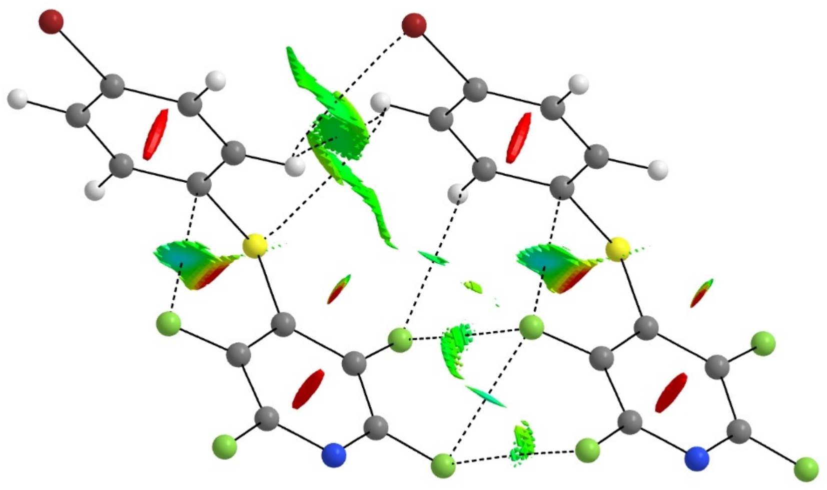

Figure S152. Bond critical paths (BCP) as dashed lines and reduced density gradient (RDG) isosurface (isovalue 0.5 mapped on a Blue-Green-Red color scale with values -0.02 < ρ*signλ$_2$ < 0.02 a.u.) in pair.

Table S66. Distances, dissociation energies and Laplacian of electron densities ($\nabla^2\rho$) of F…F bond critical paths (BCP) identified by QTAIM analysis and corresponding donor and acceptor contributions and stabilization energies in Lewis donor-acceptor orbital interactions from NBO analysis. LP = lone pair, BD* = anti-bonding.

Interaction	Distance Å	BCP Dissociation Energy kJ mol^{-1}	$\nabla^2\rho$ e bohr^{-3}	Donor	Acceptor	D-A Stabilization Energy kJ mol^{-1}
F…F	2.830	10.2	0.0384	LP F	BD* C-C	1.34
F…F	3.044	5.6	0.0255	LP F	BD* C-C	0.25
F…F	3.131	4.2	0.0210	-	-	-

Compound **12** pair 5 containing F…F interactions

Total interaction energy = 19.9 kJ mol^{-1}

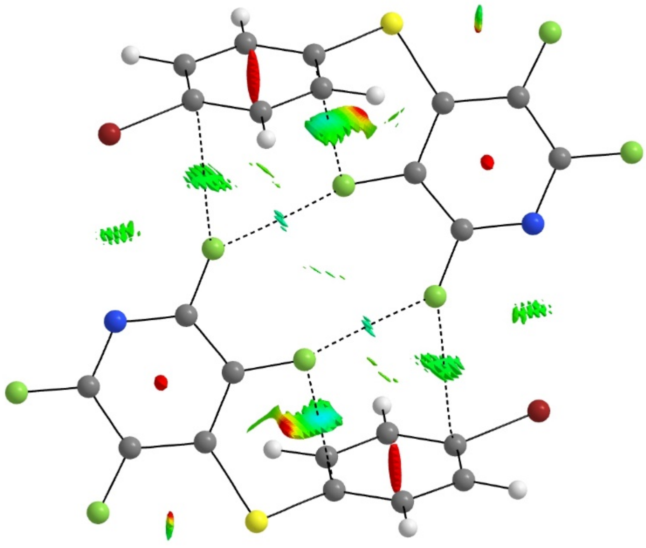

Figure S153. Bond critical paths (BCP) as dashed lines and reduced density gradient (RDG) isosurface (isovalue 0.5 mapped on a Blue-Green-Red color scale with values -0.02 < ρ*signλ$_2$ < 0.02 a.u.) in pair.

Table S67. Distances, dissociation energies and Laplacian of electron densities ($\nabla^2\rho$) of F…F bond critical paths (BCP) identified by QTAIM analysis and corresponding donor and acceptor contributions and stabilization energies in Lewis donor-acceptor orbital interactions from NBO analysis. LP = lone pair, BD* = anti-bonding.

Interaction	Distance Å	BCP Dissociation Energy kJ mol^{-1}	$\nabla^2\rho$ e bohr^{-3}	Donor	Acceptor	D-A Stabilization Energy kJ mol^{-1}
F…F	2.819	8.5	0.0342	LP F	BD* C-C	0.21
F…F	2.819	8.5	0.0342	LP F	BD* C-C	0.21

Compound **12** pair 7 containing F…F interactions

Total interaction energy = 5.3 kJ mol^{-1}

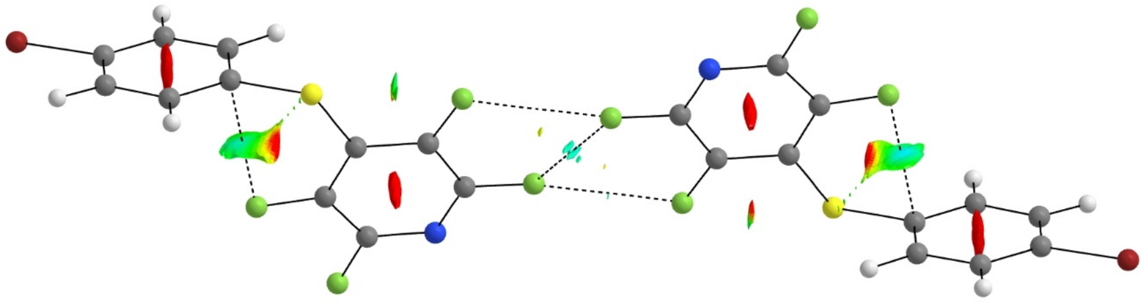

Figure S154. Bond critical paths (BCP) as dashed lines and reduced density gradient (RDG) isosurface (isovalue 0.5 mapped on a Blue-Green-Red color scale with values -0.02 < ρ*signλ_2 < 0.02 a.u.) in pair.

Table S68. Distances, dissociation energies and Laplacian of electron densities ($\nabla^2\rho$) of F…F bond critical paths (BCP) identified by QTAIM analysis and corresponding donor and acceptor contributions and stabilization energies in Lewis donor-acceptor orbital interactions from NBO analysis. LP = lone pair, BD* = anti-bonding.

Interaction	Distance Å	BCP Dissociation Energy kJ mol^{-1}	$\nabla^2\rho$ e bohr^{-3}	Donor	Acceptor	D-A Stabilization Energy kJ mol^{-1}
F…F	2.816	9.5	0.0371	LP F	BD* C-C	0.42
F…F	2.899	6.8	0.0292	LP F	BD* C-C	0.25
F…F	2.899	6.8	0.0291	LP F	BD* C-C	0.25

Compound **14** pair 1 containing F...F interactions

Total interaction energy = 47.8 kJ mol^{-1}

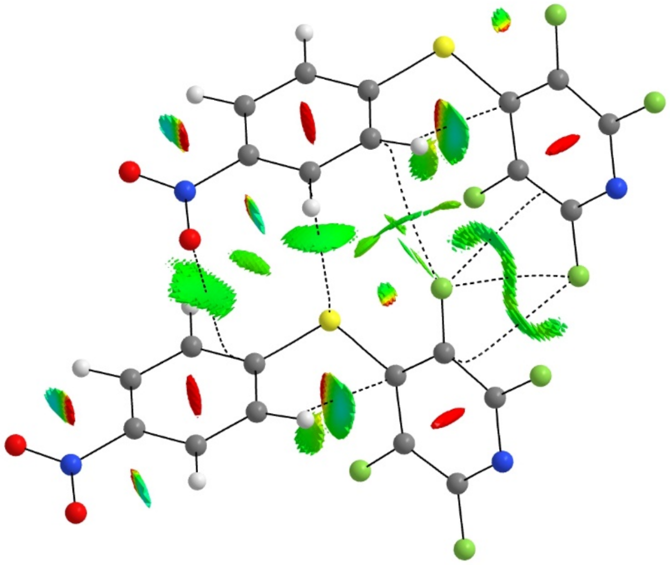

Figure S155. Bond critical paths (BCP) as dashed lines and reduced density gradient (RDG) isosurface (isovalue 0.5 mapped on a Blue-Green-Red color scale with values $-0.02 < \rho*\text{sign}\lambda_2 < 0.02$ a.u.) in pair.

Table S69. Distances, dissociation energy and Laplacian of electron densities ($\nabla^2\rho$) of F...F bond critical path (BCP) identified by QTAIM analysis and corresponding donor and acceptor contributions and stabilization energies in Lewis donor-acceptor orbital interactions from NBO analysis. LP = lone pair, BD* = anti-bonding.

Interaction	Distance Å	BCP Dissociation Energy kJ mol^{-1}	$\nabla^2\rho$ e $bohr^{-3}$	Donor	Acceptor	D-A Stabilization Energy kJ mol^{-1}
F...F	3.012	6.6	0.0295	LP F	BD* C-C	0.42
F...F	3.054	-	-	LP F	BD* C-C	0.29
F...F	3.083	-	-	LP F	BD* C-C	0.29

Compound **14** pair 2 containing F…F interaction

Total interaction energy = 31.9 kJ mol^{-1}

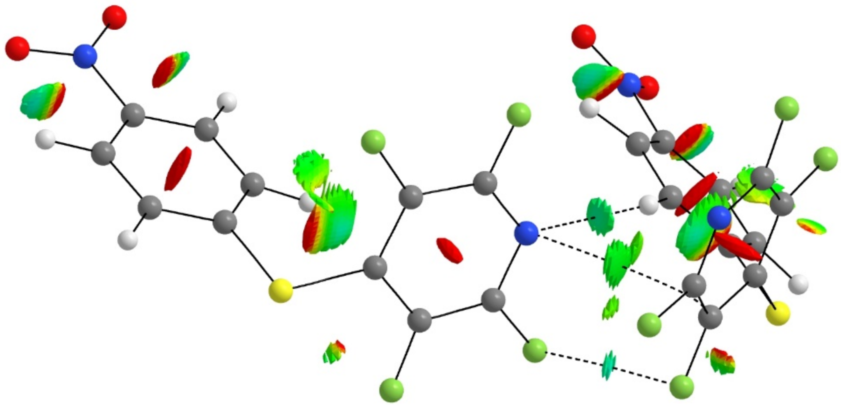

Figure S156. Bond critical paths (BCP) as dashed lines and reduced density gradient (RDG) isosurface (isovalue 0.5 mapped on a Blue-Green-Red color scale with values -0.02 < ρ*signλ_2 < 0.02 a.u.) in pair.

Table S70. Distance, dissociation energy and Laplacian of electron density ($\nabla^2\rho$) of F…F bond critical path (BCP) identified by QTAIM analysis and corresponding donor and acceptor contributions and stabilization energy in Lewis donor-acceptor orbital interaction from NBO analysis. LP = lone pair, BD* = anti-bonding.

Interaction	Distance Å	BCP Dissociation Energy kJ mol^{-1}	$\nabla^2\rho$ e bohr^{-3}	Donor	Acceptor	D-A Stabilization Energy kJ mol^{-1}
F…F	2.858	9.3	0.348	LP F	BD* C-C	0.79

Intramolecular interactions

Table S80. Distance, dissociation energy and Laplacian of electron density ($\nabla^2\rho$) of intramolecular bond critical path (BCP) identified by QTAIM analysis. Donor contribution and stabilization energies in Lewis donor-acceptor (D-A) orbital intramolecular interactions from NBO analysis. BD* = antibonding, LP = lone pair, BD = bond, 3C* 3-center C-C-C antibonding. Two independent molecules in a crystal structure are denoted as molecules A and B here.

Molecule	BCP	BCP Distance Å	BCP Dissociation Energy kJ mol^{-1}	$\nabla^2\rho$ e bohr^{-3}	D-A type	D-A Stabilization Energy kJ mol^{-1}
TFP ethers	-	-	-	-	-	-
**1**	-	-	-	-	-	-
**5**	-	-	-	-	-	-
**6**	F…C	2.814	12.3	0.0478	LP F…BD* C-C	2.6
**7**	F…C	2.869	11.0	0.0451	-	-
**8**	F…C	2.876	10.9	0.0450	-	-
**9A**	F…C	2.983	8.3	0.0377	-	-
**9B**	-	-	-	-	-	-
**10**	F…C	2.829	12.4	0.0456	-	-
TFP thioethers	-	-	-	-	-	-
**2A**	-	-	-	-	-	-
**2B**	-	-	-	-	-	-
**11A**	F…C	2.890	11.0	0.0431	-	-
**11B**	-	-	-	-	-	-
**12**	F…C	2.765	14.0	0.0511	LP F…BD* C-C	2.5
**13**	-	-	-	-	-	-
**14**	C…H	2.494	12.0	0.0524	BD C-H….3C*	2.8
**19**	-	-	-	-	-	-
TCP ethers	-	-	-	-	-	-
**3**	-	-	-	-	-	-
**16A**	-	-	-	-	-	-
**16B**	-	-	-	-	-	-
**17A**	-	-	-	-	-	-
**17B**	-	-	-	-	-	-
**18A**	-	-	-	-	-	-
**18B**	-	-	-	-	-	-
TCP thioethers	-	-	-	-	-	-
**4**	Cl…C	3.088	8.6	0.0454	LP Cl…BD* C-C	2.8
**20A**	-	-	-	-	-	-
**20B**	-	-	-	-	-	-
**21A**	-	-	-	-	-	-
**21B**	-	-	-	-	-	-
**22**	Cl…C	3.029	12.3	0.0492	LP Cl…BD* C-C	5.3

Figure S157. Intramolecular interactions in independent molecules. Dash lines show BCP identified by QTAIM analysis. Red circles highlight favorable NCI-RDG intramolecular interactions (Blue = strong and blue green = weak).

TFP ethers

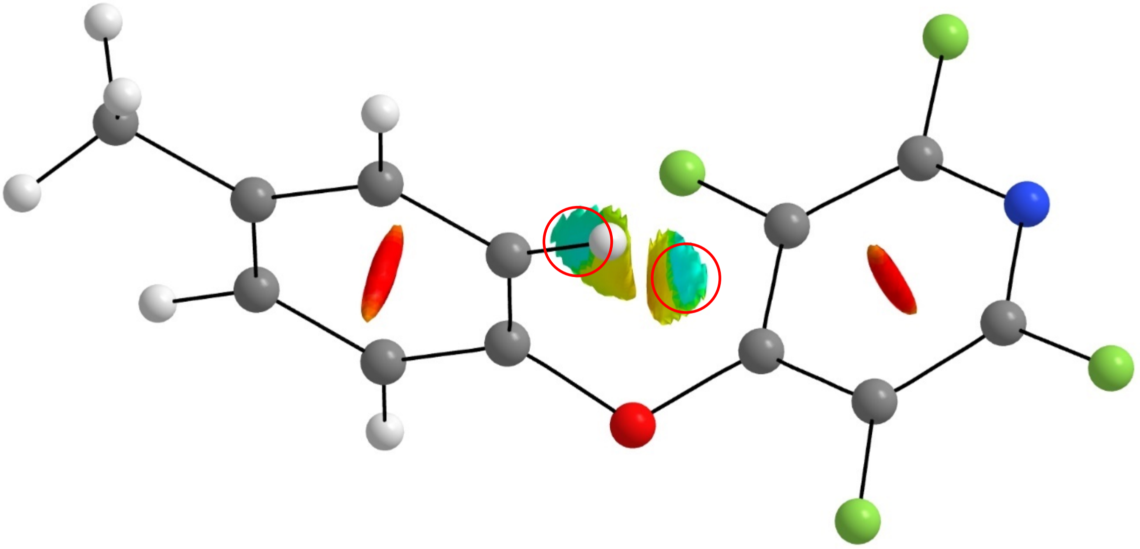

1

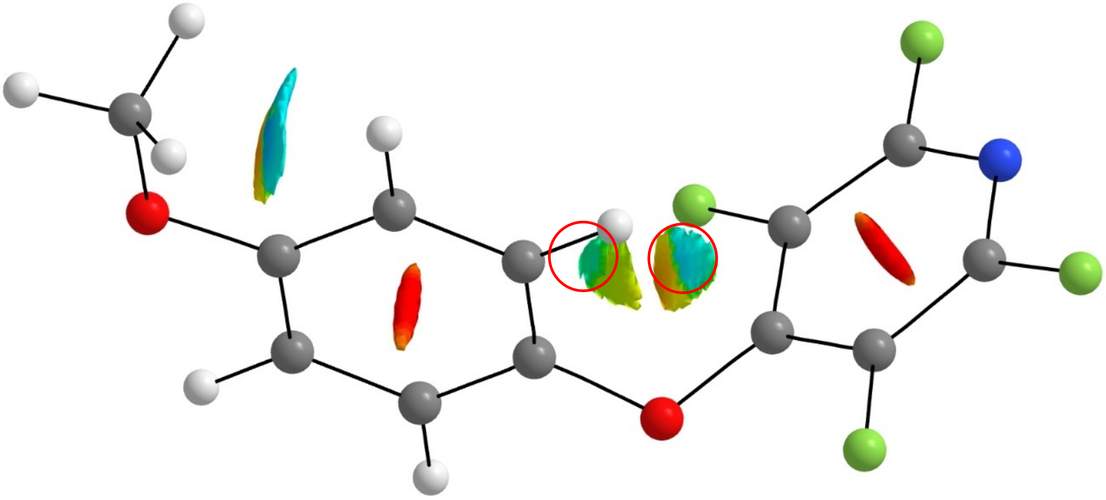

5

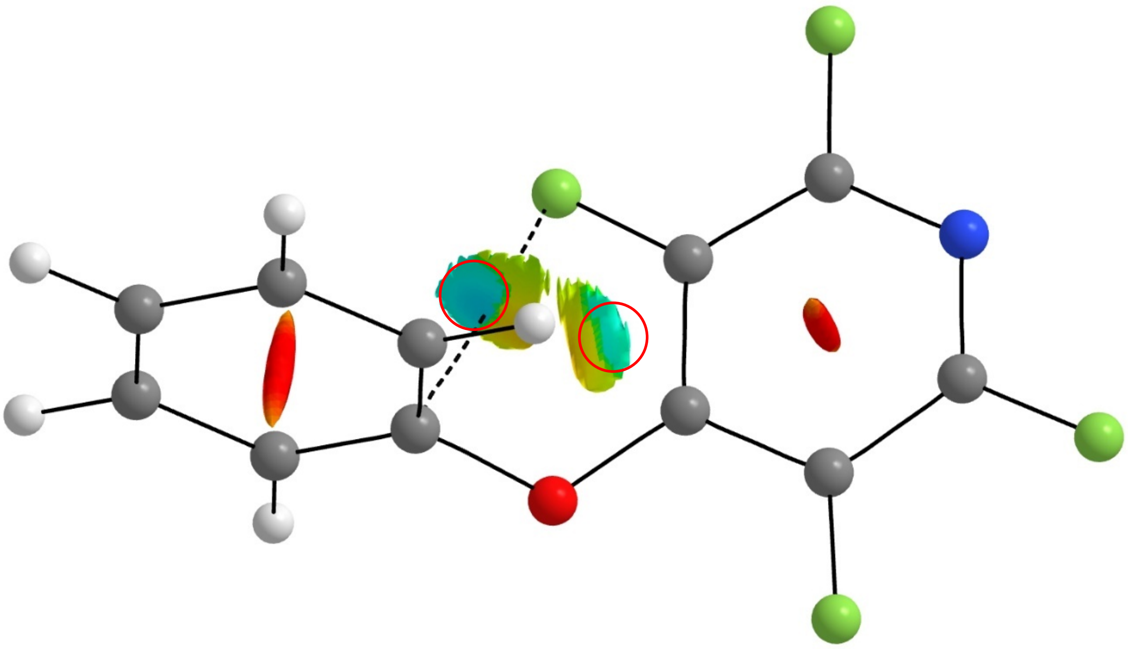

6

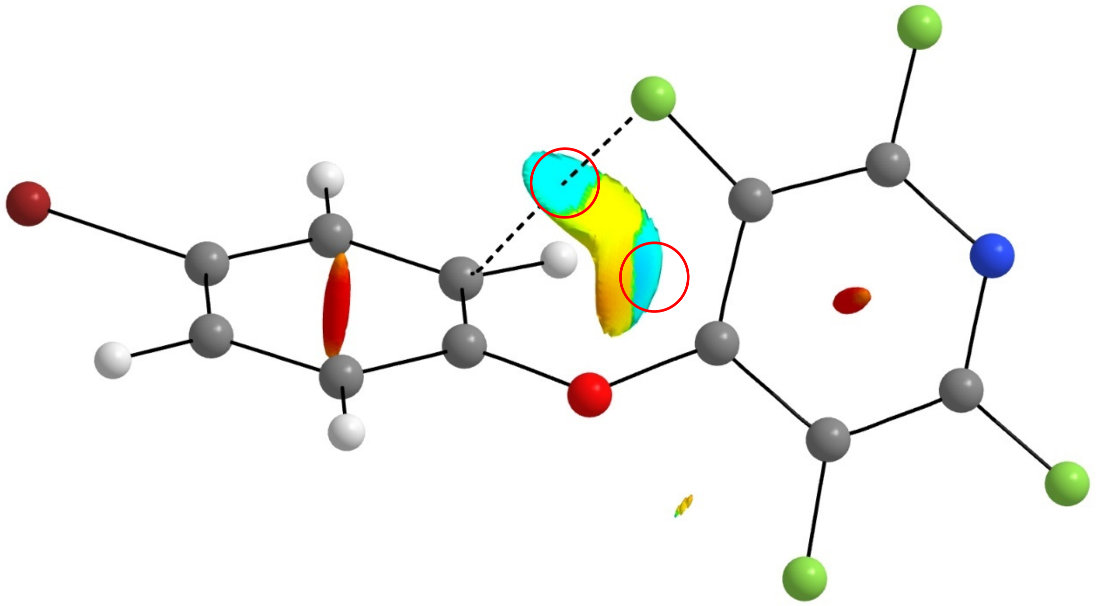

7

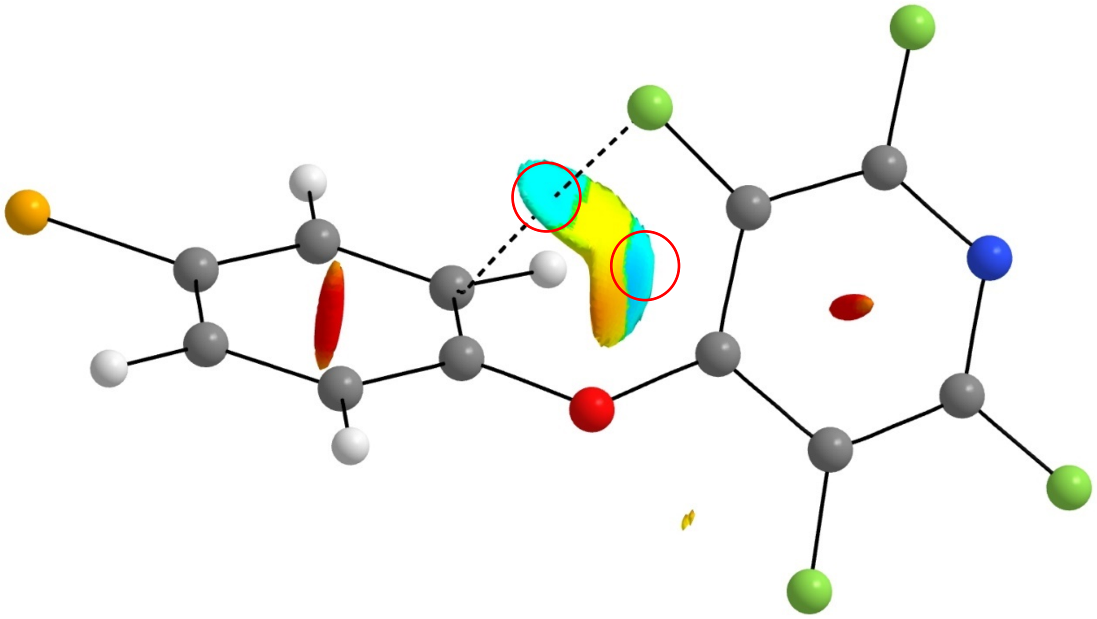

8

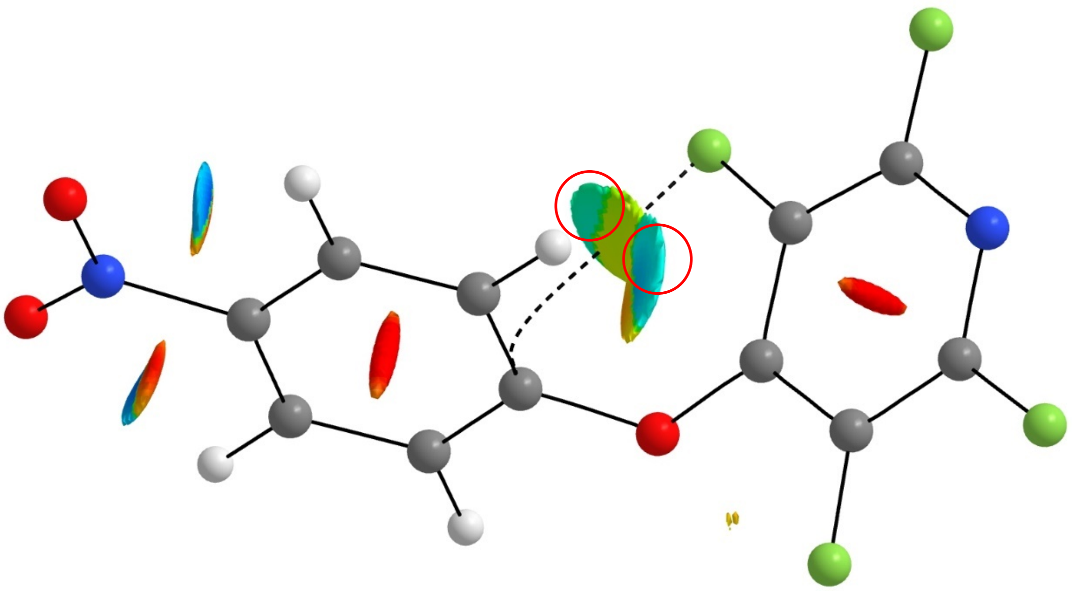

9A

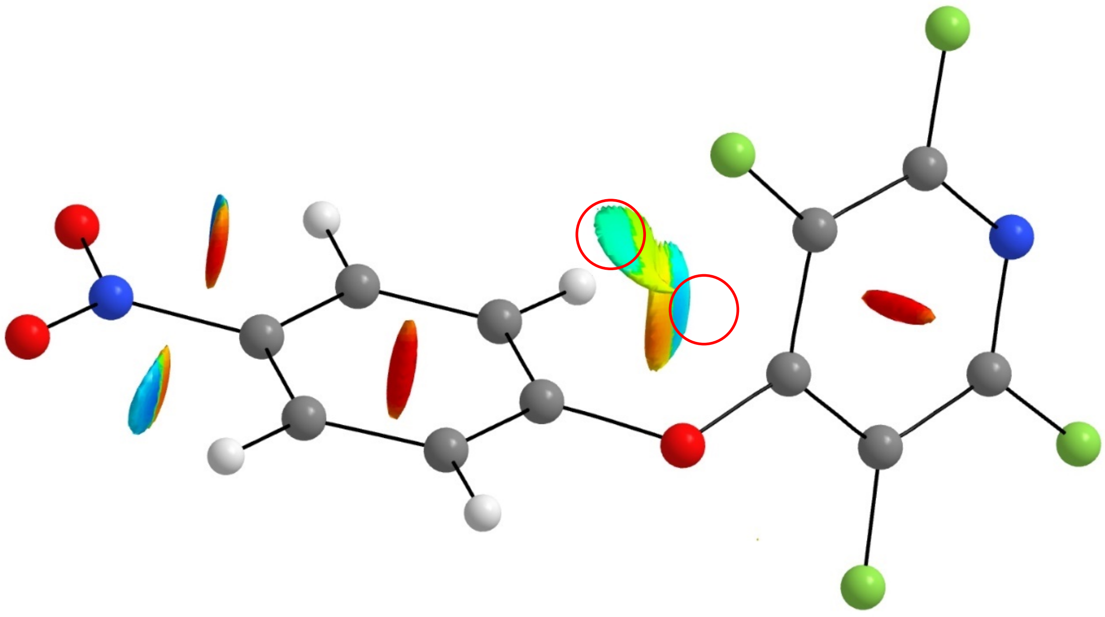

9B

TFP thioethers

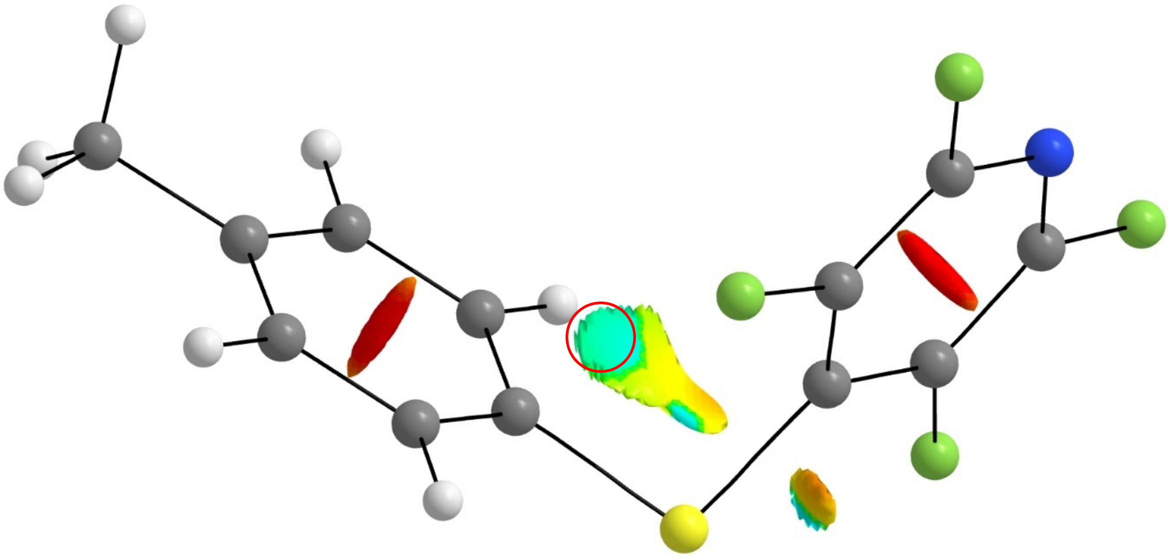

2A

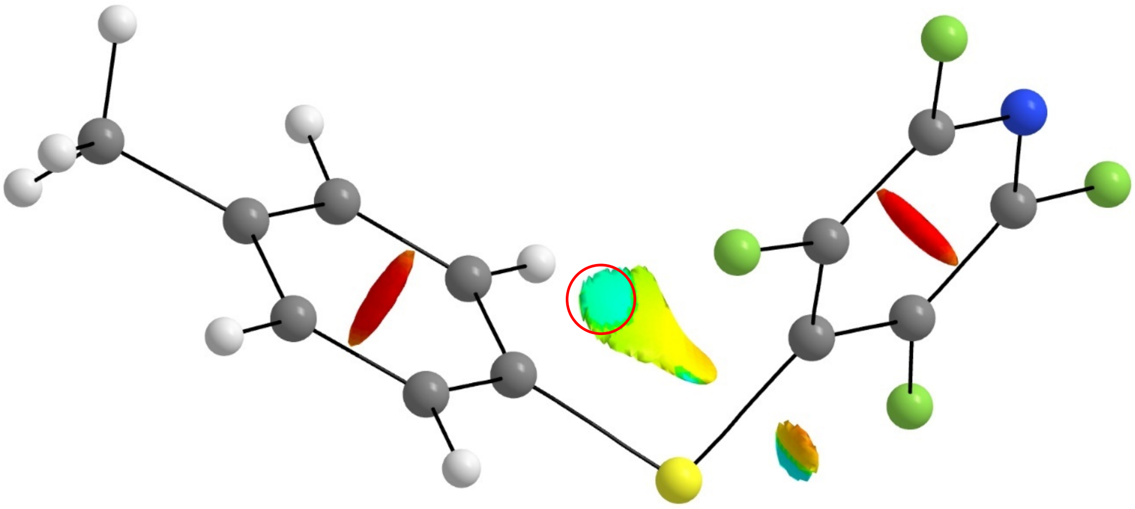

2B

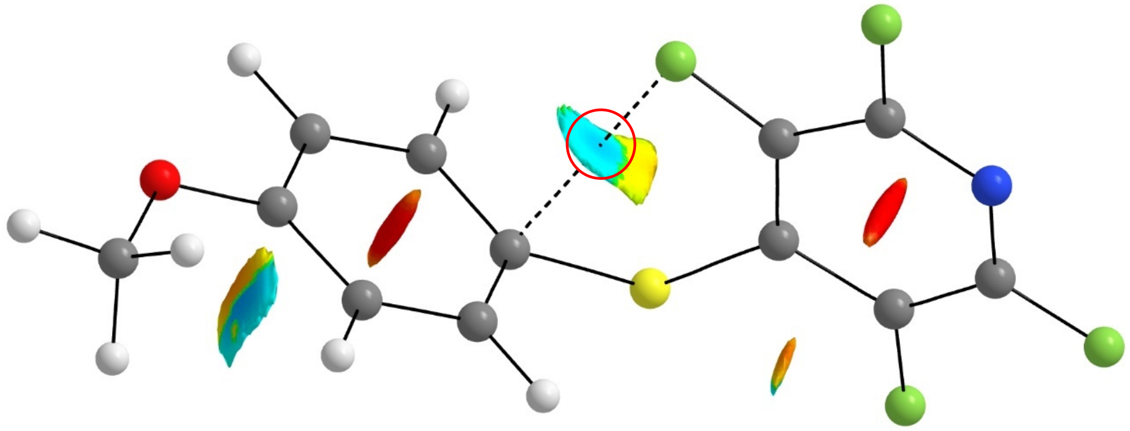

10

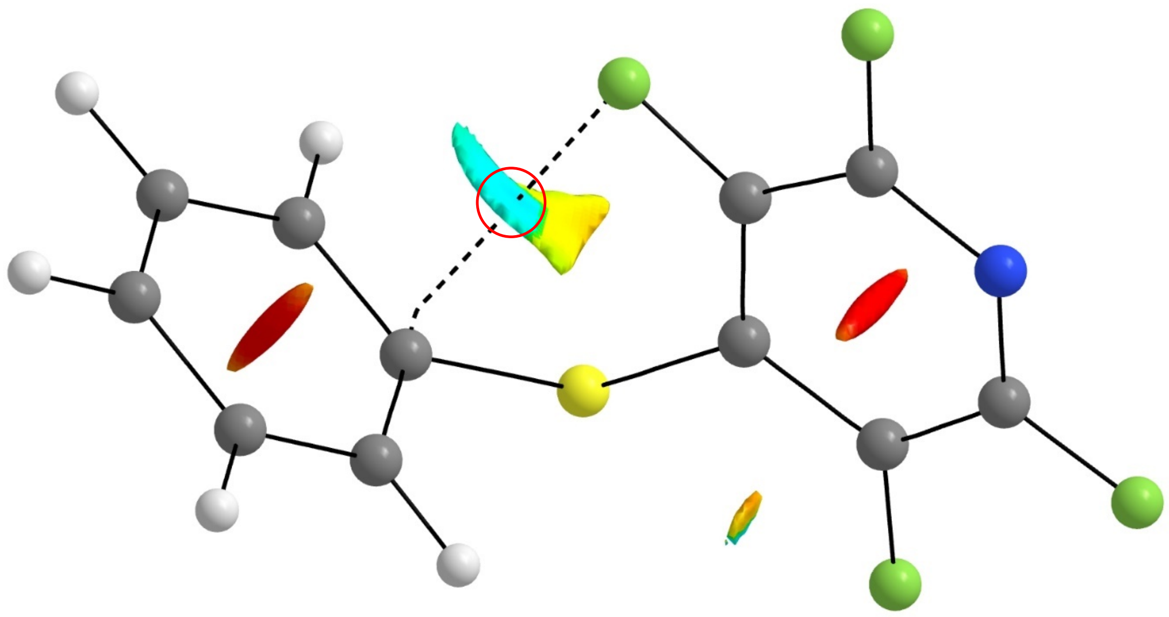

11A

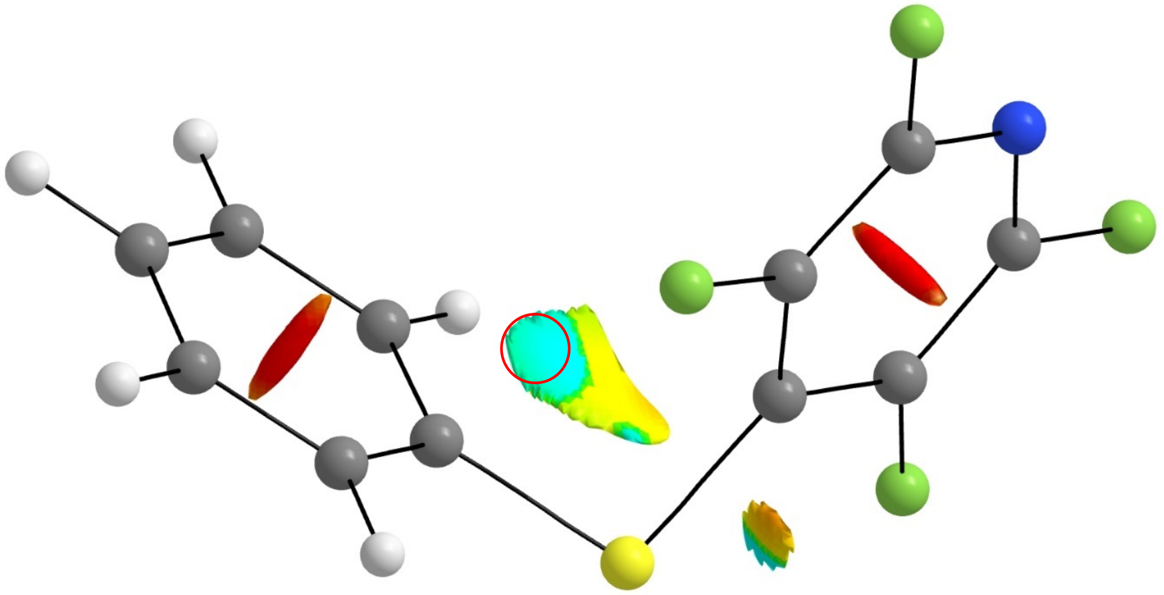

11B

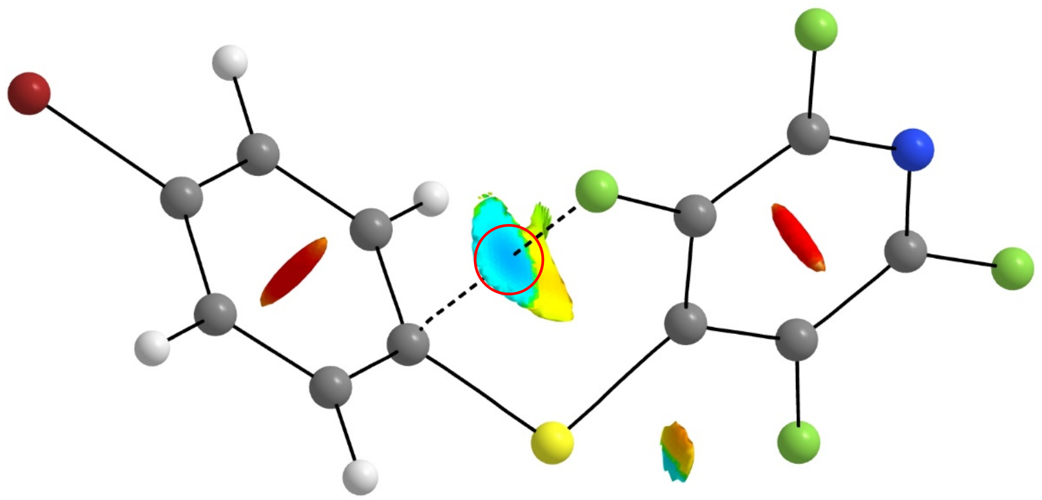

12

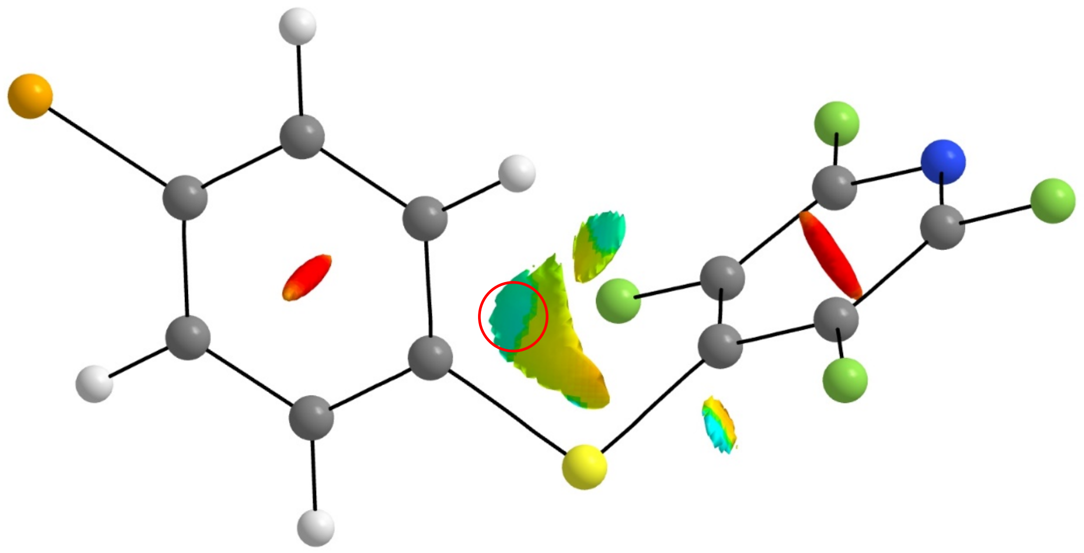

13

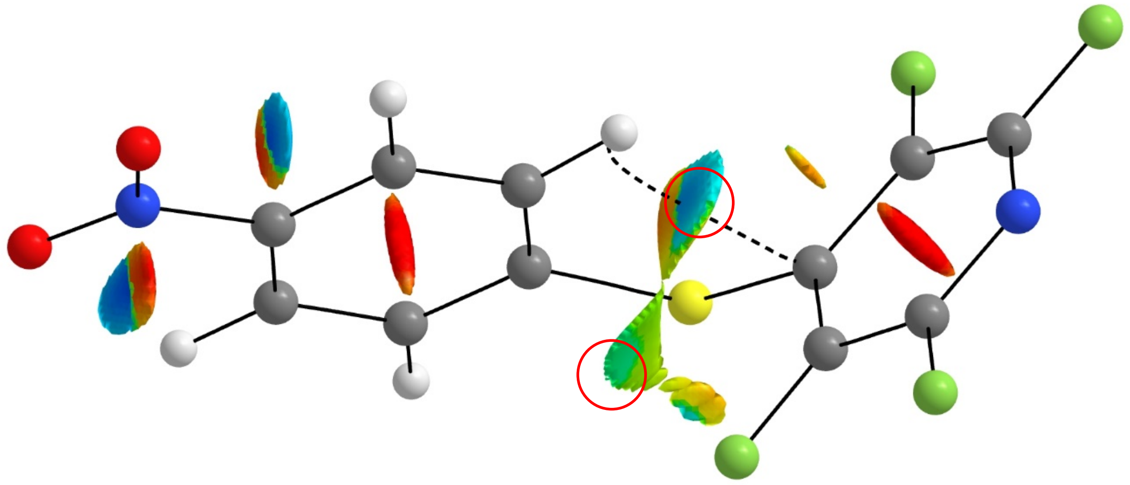

14

TCP ethers

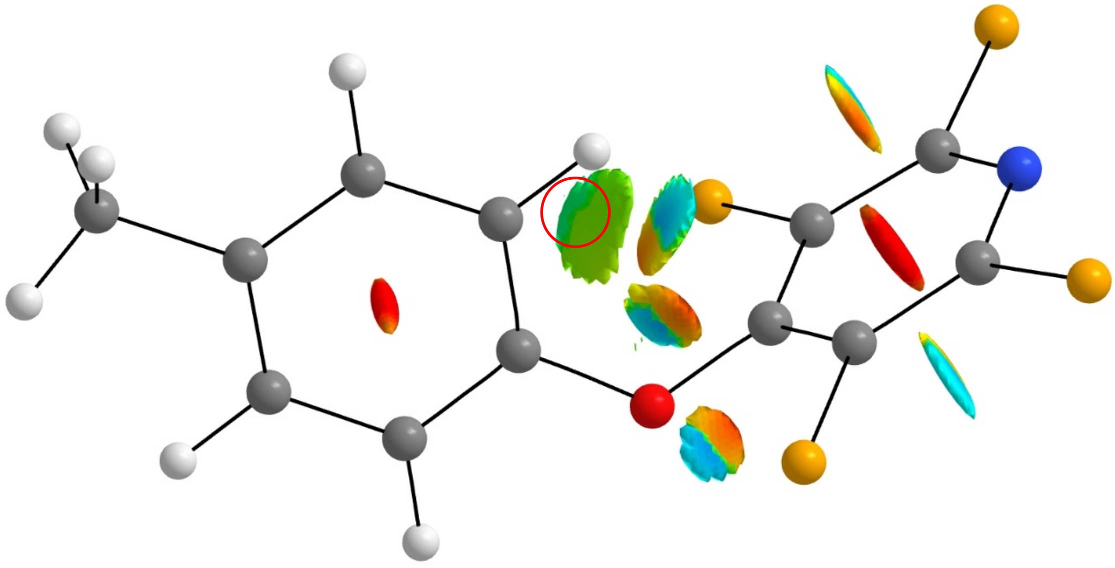

3

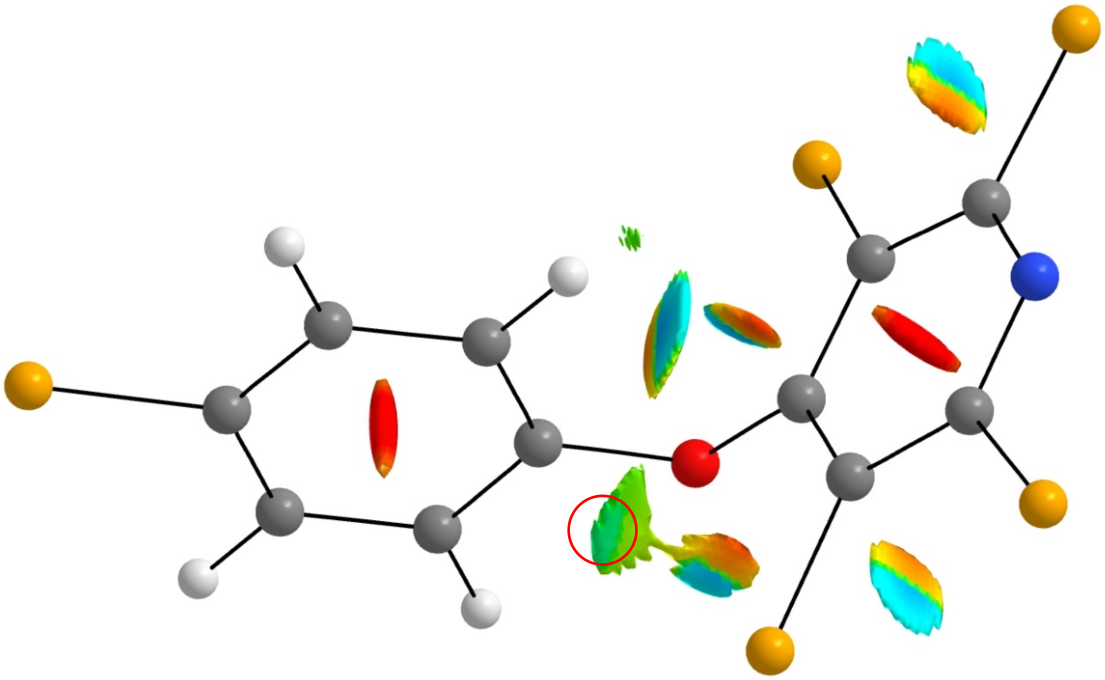

16A

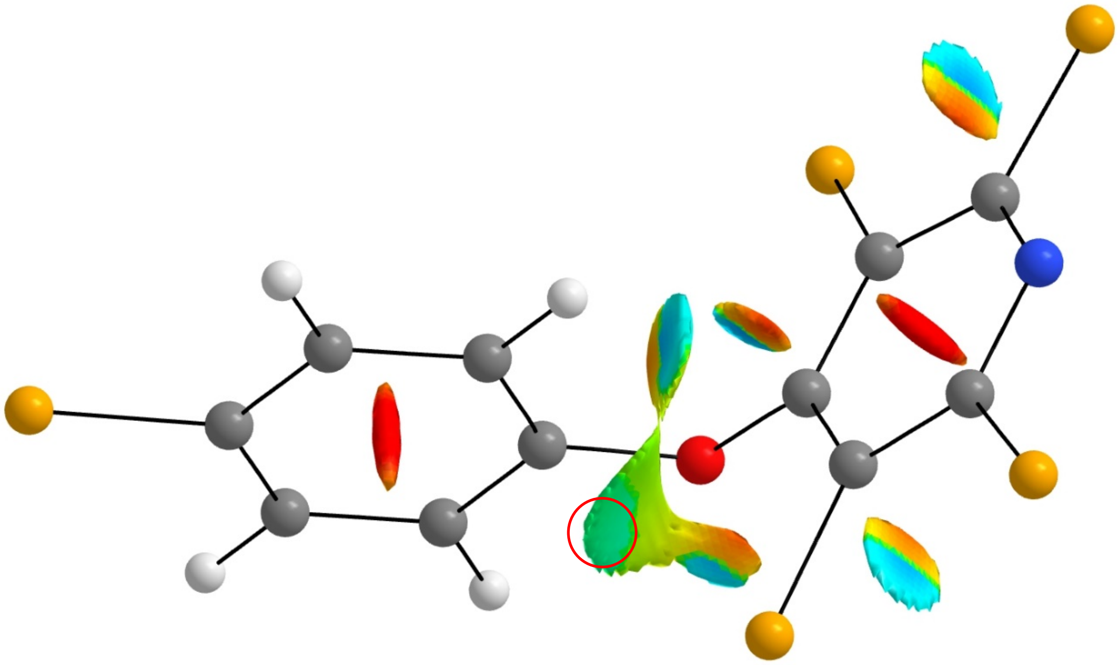

16B

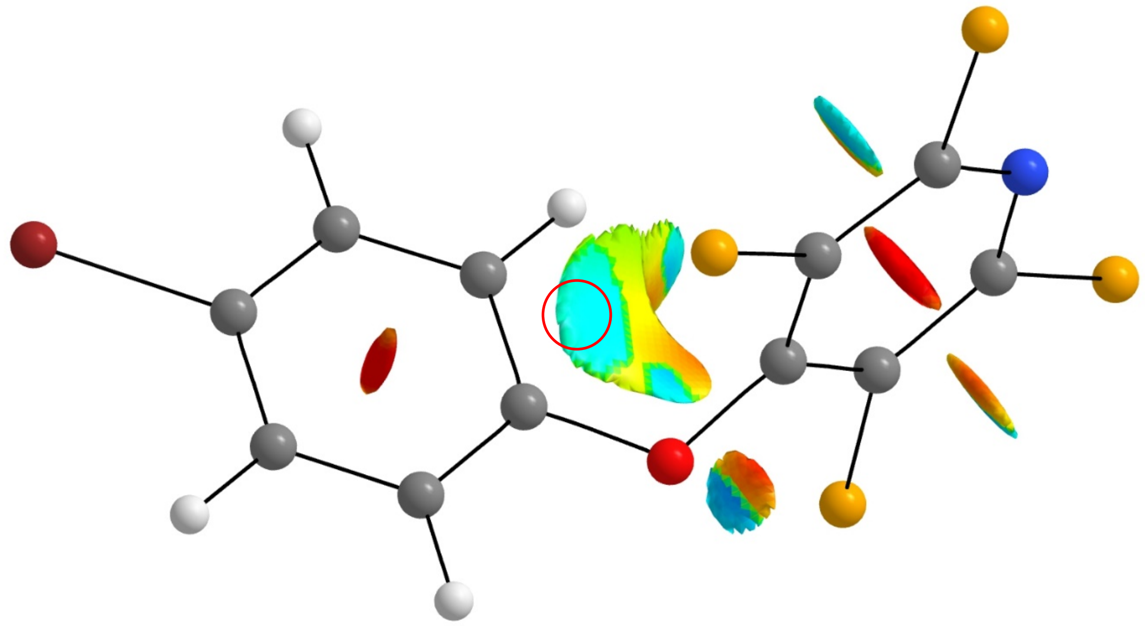

17A

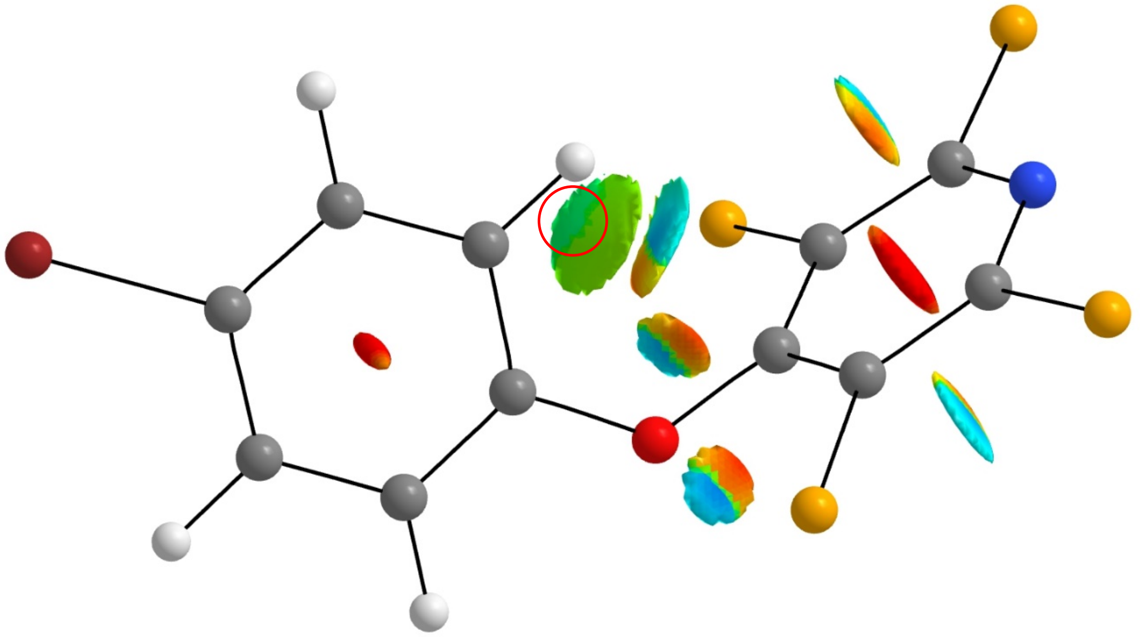

17B

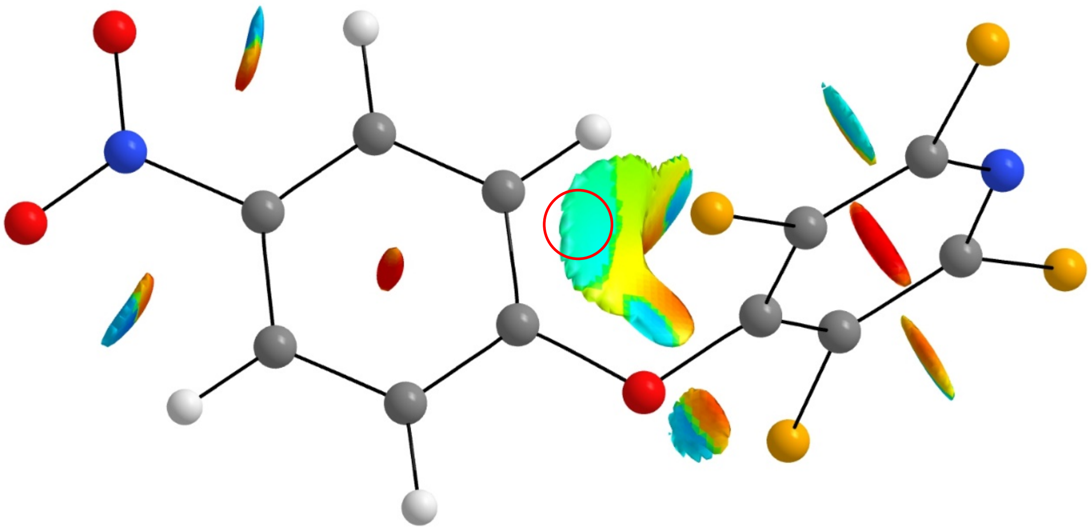

18A

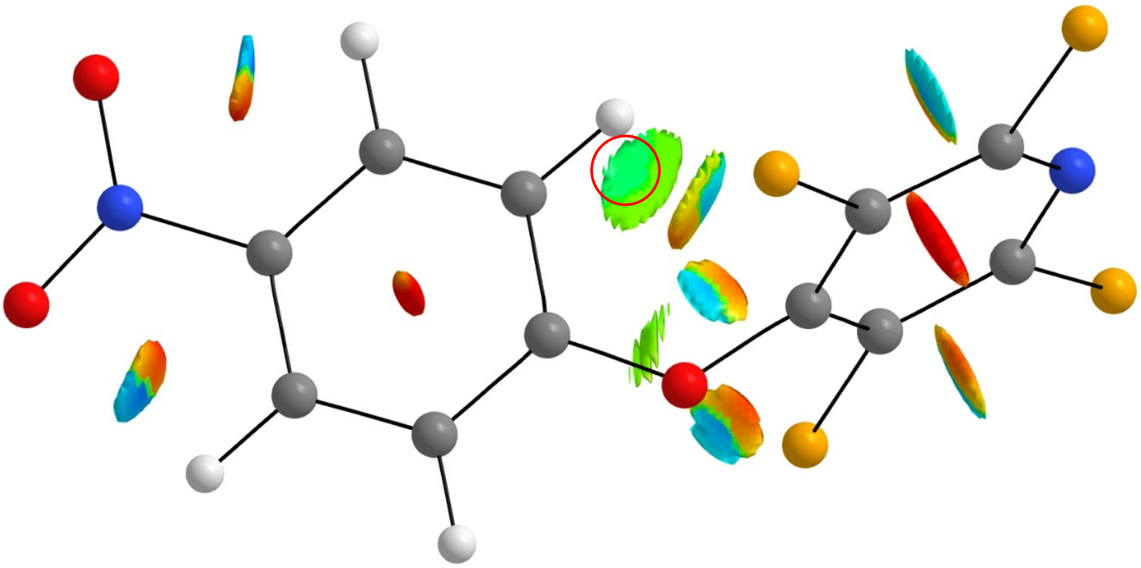

18B

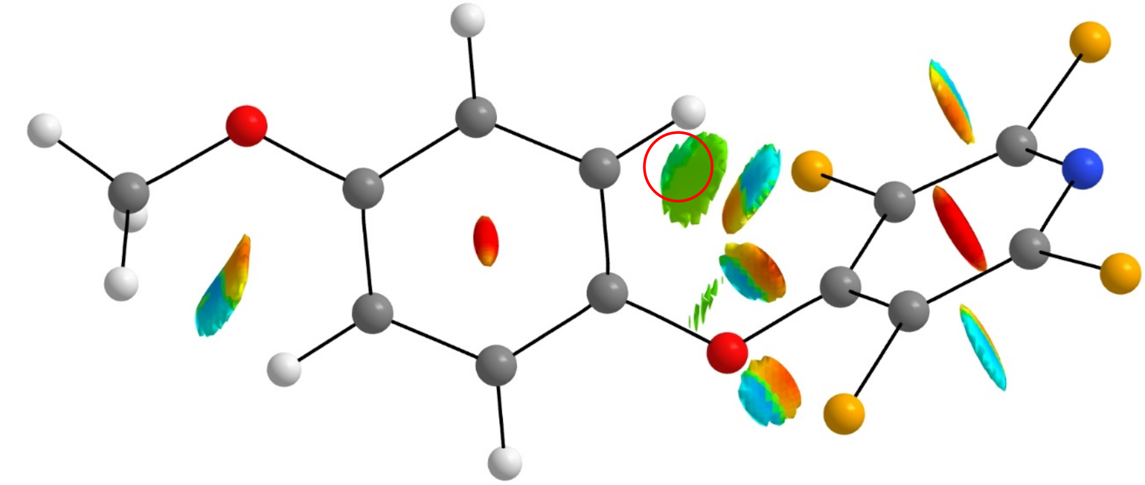

19

TCP thioethers

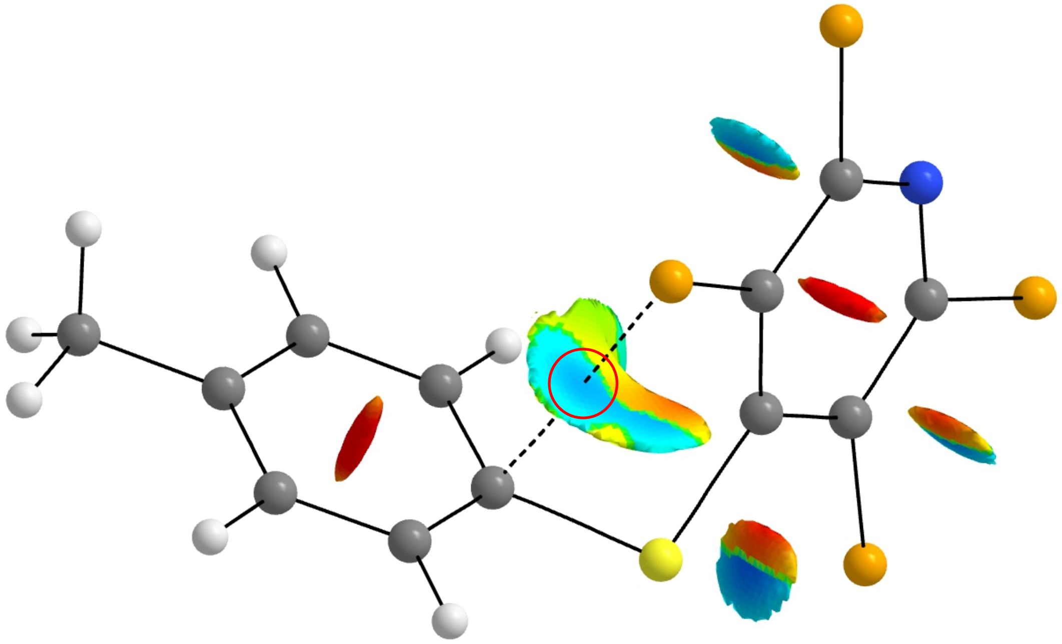

4

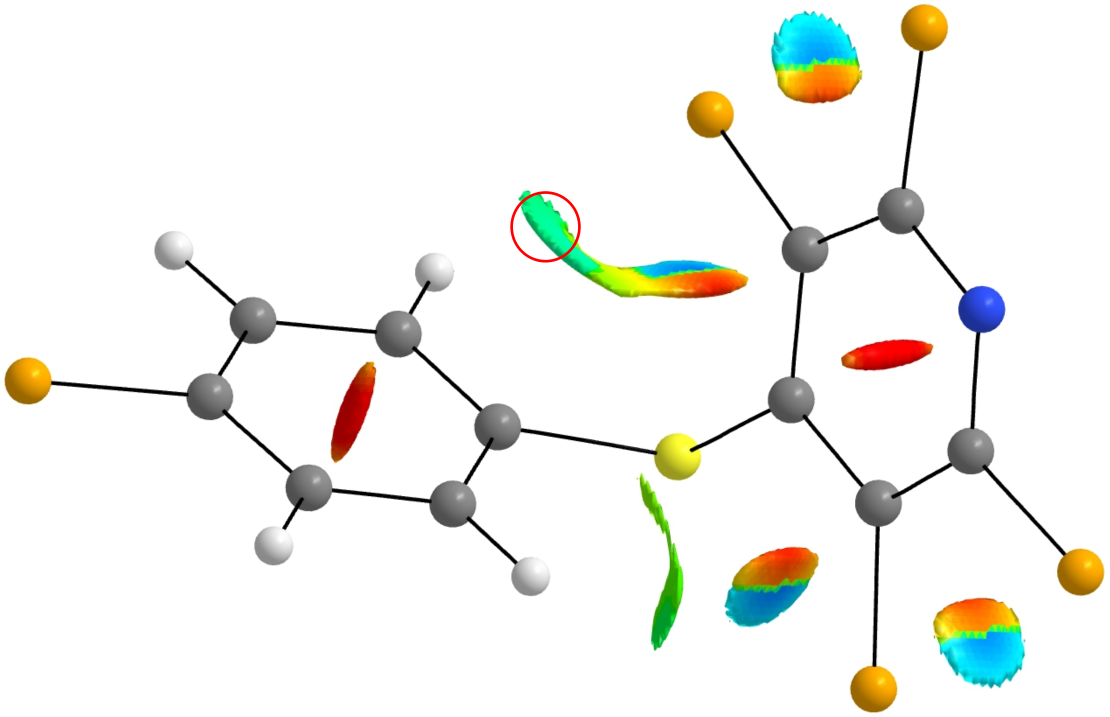

20A

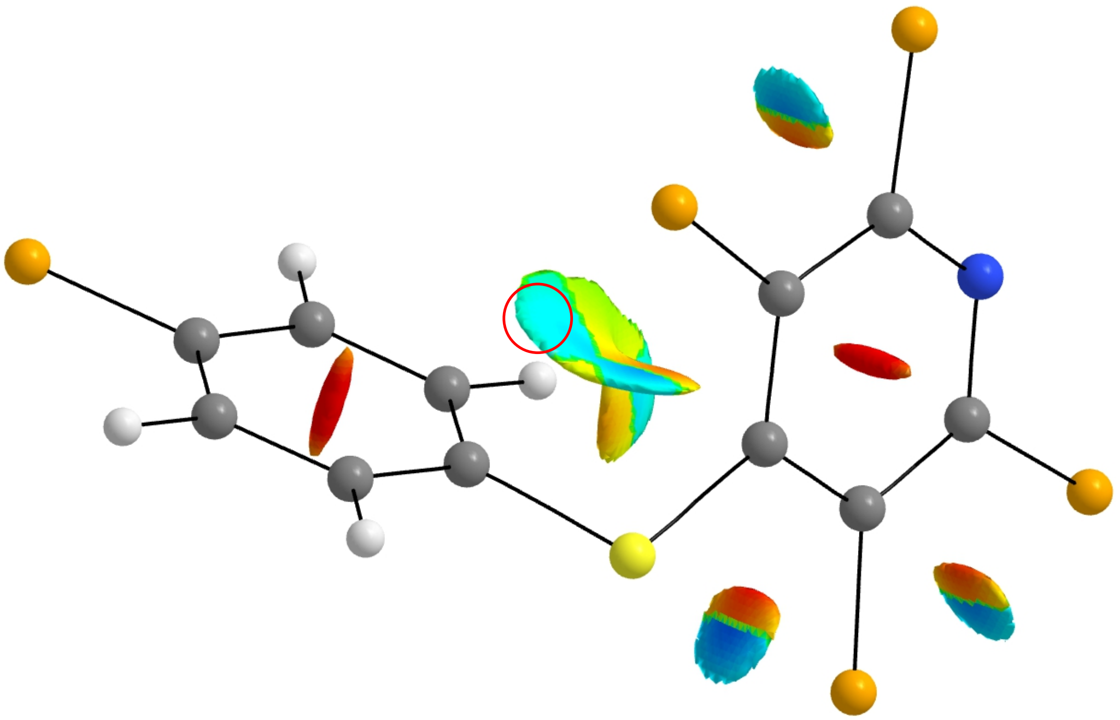

20B

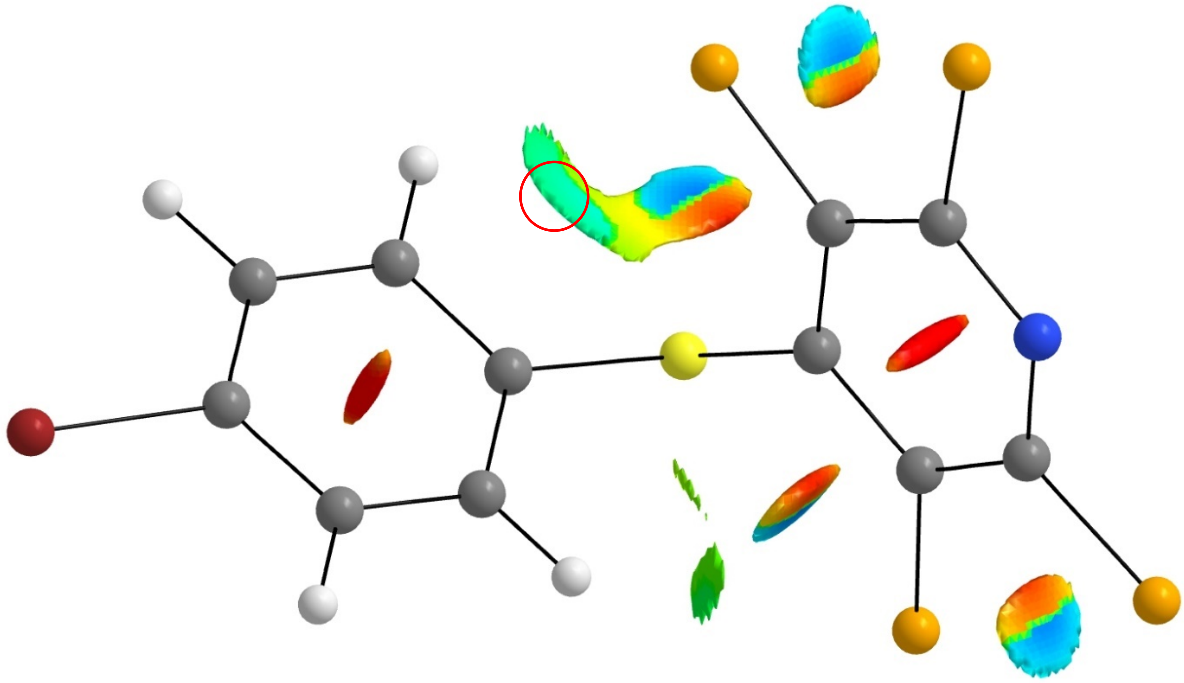

21A

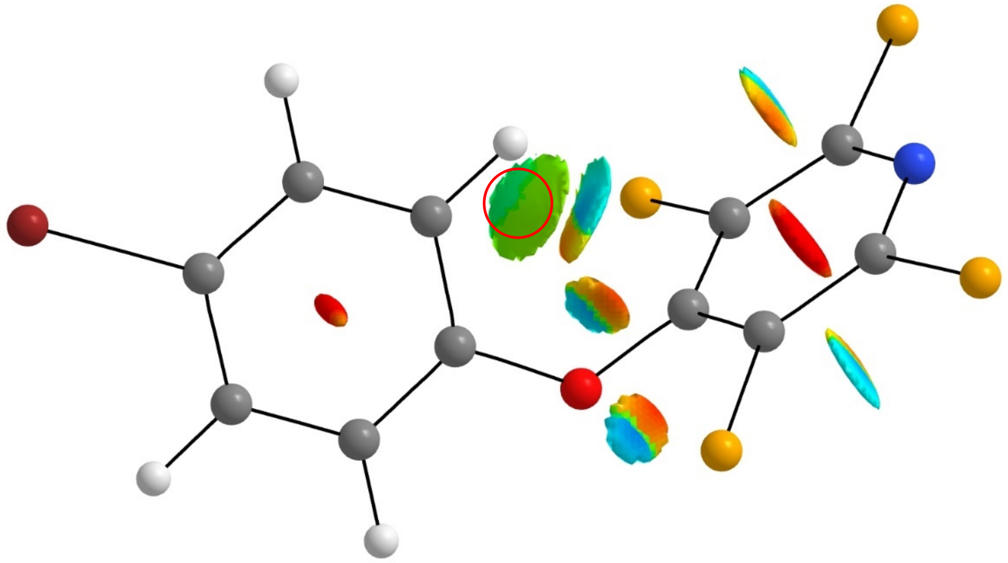

21B

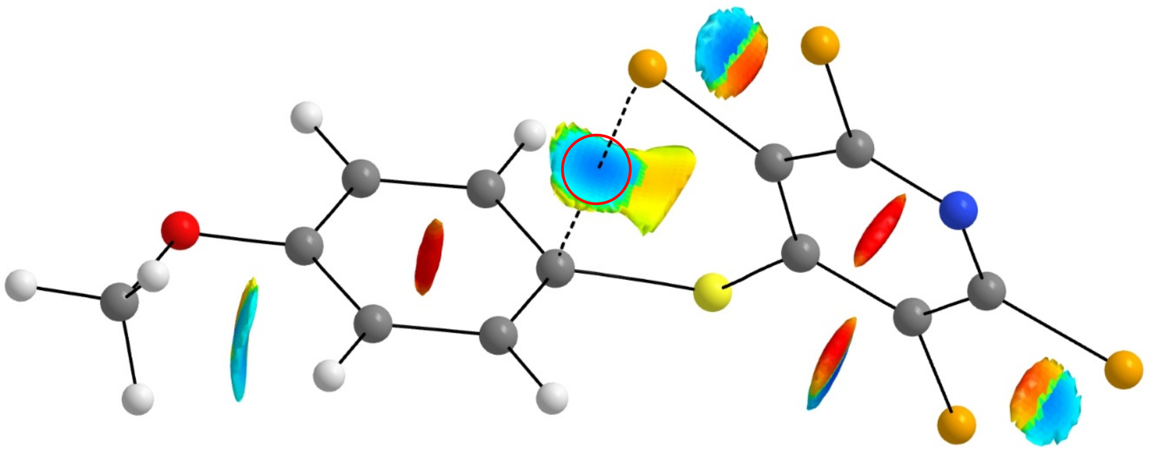

22

References

[1] a) W. D. G. Brittain, S. L. Cobb, *J. Org. Chem* **2020**, *85*, 6862-6871; b) W. D. G. Brittain, S. L. Cobb, *Org. Biomol. Chem* **2019**, *17*, 2110-2115; c) R. Gautam, I. Geniza, S. T. Iacono, C. M. Friesen, A. R. Jennings, *Molecules* **2022**, *27*, 1616.

[2] Peloquin, A. J.; Corley, C. A.; Adas, S. K.; Balaich, G. J.; Iacono, S. T. Crystal structures and Hirshfeld surface analysis of a series of 4-O-arylperfluoropyridines. Acta Crystallogr., Sect. E 2019, 75, 1102−1107.

[3] O. V Dolomanov, L. J. Bourhis, J. A. Howard and H. Puschmann, J. Appl. Cryst., 2009, 42, 339–341.

[4] C. B. Hübschle, G. M. Sheldrick and B. Dittrich, J. Appl. Cryst., 2011, 44, 1281–1284.

[5] G. M. Sheldrick, Acta Cryst., 2015, A71, 3–8.

[6] C. F. Macrae, I. Sovago, S. J. Cottrell, P. T. A. Galek, P. McCabe, E. Pidcock, M. Platings, G. P. Shields, J. S. Stevens, M. Towler, and P. A. Wood, J. Appl. Cryst., 53, 226-235. Mercury Version 4.0.

[7] R. Dennington, T. Keith and J. Millam, Semichem Inc., Shawnee Mission, KS, 2019. GaussView, Version 6

[8] [M. J. Frisch, G. W. Trucks, H. B. Schlegel, G. E. Scuseria, M. A. Robb, J. R. Cheeseman, G. Scalmani, V. Barone, G. A. Petersson, H. Nakatsuji, X. Li, M. Caricato, A. V. Marenich, J. Bloino, B. G. Janesko, R. Gomperts, B. Mennucci, H. P. Hratchian, J. V. Ortiz, A. F. Izmaylov, J. L. Sonnenberg, D. Williams-Young, F. Ding, F. Lipparini, F. Egidi, J. Goings, B. Peng, A. Petrone, T. Henderson, D. Ranasinghe, V. G. Zakrzewski, J. Gao, N. Rega, G. Zheng, W. Liang, M. Hada, M. Ehara, K. Toyota, R. Fukuda, J. Hasegawa, M. Ishida, T. Nakajima, Y. Honda, O. Kitao, H. Nakai, T. Vreven, K. Throssell, J. A. Montgomery, Jr., J. E. Peralta, F. Ogliaro, M. J. Bearpark, J. J. Heyd, E. N. Brothers, K. N. Kudin, V. N. Staroverov, T. A. Keith, R. Kobayashi, J. Normand, K. Raghavachari, A. P. Rendell, J. C. Burant, S. S. Iyengar, J. Tomasi, M. Cossi, J. M. Millam, M. Klene, C. Adamo, R. Cammi, J. W. Ochterski, R. L. Martin, K. Morokuma, O. Farkas, J. B. Foresman, and D. J. Fox, Gaussian 16, Revision B.01, Gaussian, Inc., Wallingford CT, 2016.

[9] M. C. Madhusudhanan, H. Balan, D. B. Werz, K. M. Sureshan, *Angew. Chem. Int. Ed.* **2021**, *60*, 22797-22803.

[10] T. A. Keith, AIMALL, version 19.10.12, TK Gristmill Software, Overland Park, KS, USA, 2019, http://aim.tkgristmill.com.

[11] J. P. Foster and F. Weinhold, Natural hybrid orbitals, J. Am. Chem. Soc., 102 (1980) 7211-18. A. E. Reed and F. Weinhold, Natural bond orbital analysis of near-Hartree-Fock water dimer, J. Chem. Phys., 78 (1983) 4066-73.
